# Supplementary material for: A novel Drosophila injury model reveals severed axons are cleared through a Draper/MMP-1 signaling cascade
Source: eLife. 2017 Aug 21;6:e23611. doi: 10.7554/eLife.23611 (PMC5565368; doi:10.7554/eLife.23611)
Supplement: Table 1—source code 3. — DOI: http://dx.doi.org/10.7554/eLife.23611.015 [file elife-23611-table1-code3.zip › Table 1 - Source Data 9 - human_annokey_report.html]

 Polarized endocytosis of the keratinocyte growth factor receptor in migrating cells: role of SRC-signaling and cortactin. Cell   migration   is a physiological process that requires endocytic trafficking and polarization of adhesion molecules and receptor tyrosine kinases (RTKs) to the leading edge. Many growth factors are able to induce motility by binding to specific RTK on target cells. Among them, keratinocyte growth factor (KGF or FGF7) and fibroblast growth factor 10 (FGF10), members of the FGF family, are motogenic for keratinocytes, and exert their action by binding to the keratinocyte growth factor receptor (KGFR), a splicing variant of FGFR2, exclusively expressed on epithelial cells. Here we analyzed the possible role of cortactin, an F-actin binding protein which is tyrosine phosphorylated by Src and is involved in KGFR-mediated cell   migration  , in the KGFR endocytosis and polarization to the leading edge of migrating cells upon ligand-induced stimulation. Biochemical phosphorylation study revealed that both KGF and FGF10 were able to induce tyrosine phosphorylation of Src and in turn of cortactin, as demonstrated by using the specific pharmacological Src-inhibitor SU6656, although FGF10 effect was delayed with respect to that promoted by KGF. Immunofluorescence analysis demonstrated the polarized localization of KGFR upon ligand stimulation to the leading edge of migrating keratinocytes, process that was regulated by Src. Moreover, we showed that the colocalization of cortactin with KGFR at the plasma membrane protrusions and on early endosomes after KGF and FGF10 treatment was Src-dependent. Further, by using a RNA interference approach through microinjection, we showed that cortactin is required for KGFR endocytosis and that the clathrin-dependent internalization of the receptor is a critical event for its polarization. Finally, KGFR expression and polarization enhanced cell   migration   in a scratch assay. Our results indicate that both Src and cortactin play a key role in the KGFR endocytosis and polarization at the leading edge of migrating keratinocytes, supporting the crucial involvement of RTK trafficking in cell motility. 
 The melanoma-associated antigen A3 mediates fibronectin-controlled cancer progression and metastasis. Tumor cells frequently exhibit decreased adhesiveness due to failure to deposit stromal fibronectin (FN), permitting more rapid proliferation,   migration  , invasion, and metastasis. Although up-regulation of FN has been noted in gene profiles of carcinomas compared with normal tissue, reduced FN expression has been described at the peripheral margins of invading tumors. In this study, we investigate the role of FN in cancer behavior. Using human thyroid carcinoma cells with stably down-regulated FN, we performed gene profiling and created an orthotopic mouse model. We stably overexpressed the FN target, MAGE A3, which has also been identified as a target of the breast cancer risk factor fibroblast growth factor receptor 2, and examined the functional effects in vitro and in vivo in a flank model and an orthotopic model of thyroid cancer. Mouse xenografts showed significantly enhanced tumor growth as well as larger and more numerous lung metastases in response to FN silencing. Gene profiling identified the melanoma-associated antigen (MAGE A3) as significantly up-regulated in response to FN silencing. Forced expression of MAGE A3 resulted in p21 down-regulation, accelerated cell cycle progression, increased cell   migration   rate, and invasion in vitro and in vivo in an orthotopic mouse model where microcomputed tomography confirmed lung metastases that recapitulate the progression of human thyroid cancer. We conclude that MAGE A3 is a functional integrator of diverse signals, including FGFR2 and FN, to modulate cancer progression. 
 Quantification of shape and cell polarity reveals a novel mechanism underlying malformations resulting from related FGF mutations during facial morphogenesis. Fibroblast growth factor (FGF) signaling mutations are a frequent contributor to craniofacial malformations including midfacial anomalies and craniosynostosis. FGF signaling has been shown to control cellular mechanisms that contribute to facial morphogenesis and growth such as proliferation, survival,   migration   and differentiation. We hypothesized that FGF signaling not only controls the magnitude of growth during facial morphogenesis but also regulates the direction of growth via cell polarity. To test this idea, we infected migrating neural crest cells of chicken embryos with  replication-competent avian sarcoma virus expressing either FgfR2(C278F), a receptor mutation found in Crouzon syndrome or the ligand Fgf8. Treated embryos exhibited craniofacial malformations resembling facial dysmorphologies in craniosynostosis syndrome. Consistent with our hypothesis, ectopic activation of FGF signaling resulted in decreased cell proliferation, increased expression of the Sprouty class of FGF signaling inhibitors, and repressed phosphorylation of ERK/MAPK. Furthermore, quantification of cell polarity in facial mesenchymal cells showed that while orientation of the Golgi body matches the direction of facial prominence outgrowth in normal cells, in FGF-treated embryos this direction is randomized, consistent with aberrant growth that we observed. Together, these data demonstrate that FGF signaling regulates cell proliferation and cell polarity and that these cell processes contribute to facial morphogenesis. 
  GeneRIFs  
 Results show that ESRP1 regulates the expression pattern of FGFR-2 isoforms, attenuates cell growth,   migration  , invasion and metastasis, and is a favorable prognostic factor in pancreatic ductal adenocarcinoma. 
  ECM  PubMed  
 Heparanase cleavage of perlecan heparan sulfate modulates FGF10 activity during ex vivo submandibular gland branching morphogenesis. Heparan sulfate proteoglycans are essential for biological processes regulated by fibroblast growth factors (FGFs). Heparan sulfate (HS) regulates the activity of FGFs by acting as a coreceptor at the cell surface, enhancing FGF-FGFR affinity, and being a storage reservoir for FGFs in the extracellular matrix (  ECM  ). Here we demonstrate a critical role for heparanase during mouse submandibular gland (SMG) branching morphogenesis. Heparanase, an endoglycosidase, colocalized with perlecan in the basement membrane and in epithelial clefts of SMGs. Inhibition of heparanase activity in organ culture decreased branching morphogenesis, and this inhibition was rescued specifically by FGF10 and not by other FGFs. By contrast, exogenous heparanase increased SMG branching and MAPK signaling and, surprisingly, when isolated epithelia were cultured in a three-dimensional   ECM   with FGF10, it increased the number of lateral branches and end buds. In a solid-phase binding assay, an FGF10-FGFR2b complex was released from the   ECM   by heparanase. In addition, surface plasmon resonance (SPR) analysis showed that FGF10 and the FGF10-FGFR2b complex bound to purified perlecan HS and could be released by heparanase. We used the FGF10-FGFR2b complex as a probe for HS in SMGs, and it colocalized with perlecan in the basement membrane and partly colocalized with syndecan 1 in the epithelium, and binding was reduced by treatment with heparanase. In summary, our results show heparanase releases FGF10 from perlecan HS in the basement membrane, increasing MAPK signaling, epithelial clefting, and lateral branch formation, which results in increased branching morphogenesis. 
 P253R fibroblast growth factor receptor-2 mutation induces RUNX2 transcript variants and calvarial osteoblast differentiation. Unregulated fibroblast growth factor 2 (FGF2) signaling caused by mutations in the fibroblast growth factor receptor (FGFR2) leads to human craniosynostosis such as the Apert syndrome. In an in vitro control model of calvarial osteoblasts from Apert patients carrying the FGFR2 P253R mutation, we studied the changes in cellular phenotype and evaluated the effects of FGF2. Compared with wild-type controls, osteocalcin mRNA was down-regulated in Apert osteoblasts, Runt-related transcription factor-2 (RUNX2) mRNA was differentially spliced, and FGF2 secretion was greater. Total protein synthesis, fibronectin and type I collagen secretion were up-regulated, while protease and glycosidase activities and matrix metalloproteinase-13 (MMP-13) transcription were decreased, suggesting an altered   ECM   turnover. Adding FGF2 increased protease and glycosidase activities and down-regulated fibronectin and type I collagen secretion in Apert osteoblasts. High affinity FGF2 receptors were up-regulated in Apert osteoblasts and analysis of signal transduction showed elevated levels of Grb2 tyrosine phosphorylation and the Grb2-p85 beta association, which FGF2 stimulation strongly reduced. All together these findings suggest increased constitutive receptor activity in Apert mutant osteoblasts and an autocrine loop involving the FGF2 pathway in modulation of Apert osteoblast behavior. 
  [Ee]xtracellular [Mm]atrix  PubMed  
 Integrin activation is required for VEGF and FGF receptor protein presence on human microvascular endothelial cells. Endothelial cell proliferation and migration is initiated by growth factors including FGF and VEGF that bind to specific transmembrane receptor tyrosine kinases. Mechanisms that regulate in vivo expression of fibroblast growth factor receptors (FGFR) and vascular endothelial growth factor receptors (VEGFR) are not well understood. Since it is well known that different matrices influence the proliferation and migration of endothelial cells in culture, we hypothesized that changes in the   extracellular matrix   environment can regulate growth factor receptors on endothelial cells. We cultured human microvascular endothelial cells on different matrices (vitronectin, laminin, fibronectin, fibrin, and collagen IV) and examined for the presence of growth factor receptors (FGFR-1, FGFR-2, VEGFR-1, and VEGFR-2). We show that vitronectin increased the presence of all four growth factor receptors and most notably, VEGFR-1. In contrast, fibrin decreased all four receptors, especially FGFR-1 and FGFR-2. Inhibiting phosphotyrosine signaling abolished immunostaining for all four receptors, regardless of the matrix, but was not dependent on activating the Fyn-Shc pathway. Cells plated on vitronectin in the presence of blocking antibodies to integrins alphavbeta3 and alphavbeta5 similarly decreased presence of these growth factor receptors. Our data suggests a possible mechanism of how matrix-integrin interactions regulate endothelial cell responsiveness to growth factors and anchorage-dependent cell growth. 
 Integrin activation is required for VEGF and FGF receptor protein presence on human microvascular endothelial cells. Endothelial cell proliferation and migration is initiated by growth factors including FGF and VEGF that bind to specific transmembrane receptor tyrosine kinases. Mechanisms that regulate in vivo expression of fibroblast growth factor receptors (FGFR) and vascular endothelial growth factor receptors (VEGFR) are not well understood. Since it is well known that different matrices influence the proliferation and migration of endothelial cells in culture, we hypothesized that changes in the   extracellular matrix   environment can regulate growth factor receptors on endothelial cells. We cultured human microvascular endothelial cells on different matrices (vitronectin, laminin, fibronectin, fibrin, and collagen IV) and examined for the presence of growth factor receptors (FGFR-1, FGFR-2, VEGFR-1, and VEGFR-2). We show that vitronectin increased the presence of all four growth factor receptors and most notably, VEGFR-1. In contrast, fibrin decreased all four receptors, especially FGFR-1 and FGFR-2. Inhibiting phosphotyrosine signaling abolished immunostaining for all four receptors, regardless of the matrix, but was not dependent on activating the Fyn-Shc pathway. Cells plated on vitronectin in the presence of blocking antibodies to integrins alphavbeta3 and alphavbeta5 similarly decreased presence of these growth factor receptors. Our data suggests a possible mechanism of how matrix-integrin interactions regulate endothelial cell responsiveness to growth factors and anchorage-dependent cell growth. 
 Heparanase cleavage of perlecan heparan sulfate modulates FGF10 activity during ex vivo submandibular gland branching morphogenesis. Heparan sulfate proteoglycans are essential for biological processes regulated by fibroblast growth factors (FGFs). Heparan sulfate (HS) regulates the activity of FGFs by acting as a coreceptor at the cell surface, enhancing FGF-FGFR affinity, and being a storage reservoir for FGFs in the   extracellular matrix   (ECM). Here we demonstrate a critical role for heparanase during mouse submandibular gland (SMG) branching morphogenesis. Heparanase, an endoglycosidase, colocalized with perlecan in the basement membrane and in epithelial clefts of SMGs. Inhibition of heparanase activity in organ culture decreased branching morphogenesis, and this inhibition was rescued specifically by FGF10 and not by other FGFs. By contrast, exogenous heparanase increased SMG branching and MAPK signaling and, surprisingly, when isolated epithelia were cultured in a three-dimensional ECM with FGF10, it increased the number of lateral branches and end buds. In a solid-phase binding assay, an FGF10-FGFR2b complex was released from the ECM by heparanase. In addition, surface plasmon resonance (SPR) analysis showed that FGF10 and the FGF10-FGFR2b complex bound to purified perlecan HS and could be released by heparanase. We used the FGF10-FGFR2b complex as a probe for HS in SMGs, and it colocalized with perlecan in the basement membrane and partly colocalized with syndecan 1 in the epithelium, and binding was reduced by treatment with heparanase. In summary, our results show heparanase releases FGF10 from perlecan HS in the basement membrane, increasing MAPK signaling, epithelial clefting, and lateral branch formation, which results in increased branching morphogenesis. 
 FGF2 effects in periosteal fibroblasts bearing the FGFR2 receptor Pro253 Arg mutation. A growing number of mutations mapped in the receptor gene for fibroblast growth factor have been implicated in several cranial development disorders including the Apert and Crouzon syndromes. The present paper investigated cellular mechanisms underlying Apert phenotype, by analyzing the effects of FGF2 in primary cultures of Apert periosteal fibroblasts carrying the FGFR2 Pro253Arg mutation.FGF2 administration significantly decreased   extracellular matrix   production in mutant cells by stimulating degradative enzymatic activities. Gene expression analysis revealed that decorin and biglycan, two proteoglycans involved in collagen fibrillogenesis, were more expressed in mutant cells and down-regulated by FGF2. FGF2 receptor binding showed little differences in high affinity receptor counts between mutant and wild-type cells, while we showed for the first time that low affinity receptors are significantly fewer in mutant cells. Differences were found in Crouzon syndrome, where both high and low affinity receptor counts were up-regulated.The different mutation and low affinity receptor regulation in mutant receptors support the hypothesis that the impact on the activity of the ligand-receptor complex could allow distinct modes of FGF2 activation in Apert and Crouzon syndromes, which interfere with the FGFR2 signalling cascade. 
 Fibroblast growth factor receptor 2-positive fibroblasts provide a suitable microenvironment for tumor development and progression in esophageal carcinoma. Tumor fibroblasts (TF) have been suggested to play an essential role in the complex process of tumor-stroma interactions and tumorigenesis. The aim of the present study was to investigate the specific role of TF in the esophageal cancer microenvironment.An Affymetrix expression microarray was used to compare gene expression profiles between six pairs of TFs and normal fibroblasts from esophageal squamous cell carcinoma (ESCC). Differentially expressed genes were identified, and a subset was evaluated by quantitative real-time PCR and immunohistochemistry.About 43% (126 of 292) of known deregulated genes in TFs were associated with cell proliferation,   extracellular matrix   remodeling, and immune response. Up-regulation of fibroblast growth factor receptor 2 (FGFR2), which showed the most significant change, was detected in all six tested TFs compared with their paired normal fibroblasts. A further study found that FGFR2-positive fibroblasts were only observed inside the tumor tissues and not in tumor-surrounding stromal tissues, suggesting that FGFR2 could be used as a TF-specific marker in ESCC. Moreover, the conditioned medium from TFs was found to be able to promote ESCC tumor cell growth, migration, and invasion in vitro.Our study provides new candidate genes for the esophageal cancer microenvironment. Based on our results, we hypothesize that FGFR2(+)-TFs might provide cancer cells with a suitable microenvironment via secretion of proteins that could promote cancer development and progression through stimulation of cancer cell proliferation, induction of angiogenesis, inhibition of cell adhesion, enhancement of cell mobility, and promotion of the epithelial-mesenchymal transition. 
 Apert p.Ser252Trp mutation in FGFR2 alters osteogenic potential and gene expression of cranial periosteal cells. Apert syndrome (AS), a severe form of craniosynostosis, is caused by dominant gain-of-function mutations in FGFR2. Because the periosteum contribution to AS cranial pathophysiology is unknown, we tested the osteogenic potential of AS periosteal cells (p.Ser252Trp mutation) and observed that these cells are more committed toward the osteoblast lineage. To delineate the gene expression profile involved in this abnormal behavior, we performed a global gene expression analysis of coronal suture periosteal cells from seven AS patients (p.Ser252Trp), and matched controls. We identified 263 genes with significantly altered expression in AS samples (118 upregulated, 145 downregulated; SNR >or= |0.4|, P <or= 0.05). Several upregulated genes are involved in positive regulation of cell proliferation and nucleotide metabolism, whereas several downregulated genes are involved in inhibition of cell proliferation, gene expression regulation, cell adhesion, and   extracellular matrix   organization, and in PIK3-MAPK cascades. AS expression profile was confirmed through real-time PCR of a selected set of genes using RNAs from AS and control cells as well as from control cells treated with high FGF2 concentration, and through the analysis of genes involved in FGF-FGFR signaling. Our results allowed us to: (a) suggest that AS periosteal cells present enhanced osteogenic potential, (b) unravel a specific gene expression signature characteristic of AS periosteal cells which may be associated with their osteogenic commitment, (c) identify a set of novel genes involved in the pathophysiology of AS or other craniosynostotic conditions, and (d) suggest for the first time that the periosteum might be involved in the pathophysiology of AS. 
 Characterization of recombinant human fibroblast growth factor (FGF)-10 reveals functional similarities with keratinocyte growth factor (FGF-7). A newly identified member of the fibroblast growth factor (FGF) family, designated FGF-10, is expressed during development and preferentially in adult lung. The predicted FGF-10 protein is most related to keratinocyte growth factor (KGF, or FGF-7). The latter is unique among FGFs in that it binds and signals only through the FGF receptor (FGFR2b) isoform KGF receptor (KGFR) expressed specifically by epithelial cells. In order to examine the biological and biochemical properties of human FGF-10, we isolated the cDNA and expressed its encoded protein in bacteria. The recombinant protein (rFGF-10) was a potent mitogen for Balb/MK mouse epidermal keratinocytes with activity detectable at 0.1 nM and maximal at around 5 nM. Within this concentration range, FGF-10 did not stimulate DNA synthesis in NIH/3T3 mouse fibroblasts. rFGF-10 bound the KGFR with high affinity comparable to that of KGF, and did not bind detectably to either the FGFR1c (Flg) or FGFR2c (Bek) receptor isoforms. The mitogenic activity of FGF-10 could be distinguished from that of KGF by its different sensitivity to heparin and lack of neutralization by a KGF monoclonal antibody. These results indicate that FGF-10 and KGF have similar receptor binding properties and target cell specificities, but are differentially regulated by components of the   extracellular matrix  . 
  Component  
   extracellular matrix   
  [Ii]nvasive  PubMed  
 Fibroblast growth factor receptor (FGFR) gene amplifications are rare events in bladder cancer. Activating point mutations and protein overexpression of fibroblast growth factor receptors (FGFRs), especially FGFR3, are frequent events in bladder cancer. Little is known about gene amplifications, therefore we characterized amplification of FGFR1-3 by fluorescence in-situ hybridization (FISH).Tumours of 153 patients (n = 65 pTa low-grade, n = 15 pTa high-grade, n = 37 pT1, n = 20 pT2, n = 10 pT3, n = 6 pT4) were analysed by FISH for FGFR1-3 copy numbers and screened for FGFR3 mutations and immunohistochemical protein expression. Amplifications of FGFR1 were found in 1.6% (two of 122), FGFR2 in 0.8% (one of 121) and FGFR3 in 3.4% (five of 145). All amplifications were high-level amplifications, not overlapping with polysomy. Amplifications were found in papillar  y/papill  ary-invasive tumour parts, and predominantly in tumours with enhanced Ki67 index (>10%), aberrant CK20 expression, and low p53 expression. All FGFR3-amplified samples showed concomitant FGFR3 mutations and FGFR3 protein overexpression. FGFR amplifications were not associated significantly with gender, age, grade or stage in statistical analyses.FGFR amplifications are rare events in bladder cancer, with FGFR3 amplification being the most prevalent (3.4% of cases). Concomitant FGFR3 mutations and protein overexpression indicate that FGFR3-mediated signalling in these tumours would probably be highly active. This patient subgroup may be particularly suited to FGFR-targeted pharmacotherapy. 
 The influence of genetic variation in 30 selected genes on the clinical characteristics of early onset breast cancer. Common variants that alter breast cancer risk are being discovered. Here, we determine how these variants influence breast cancer prognosis, risk and tumour characteristics.We selected 1,001 women with early onset nonfamilial   invasive   breast cancer from the Prospective study of Outcomes in Sporadic versus Hereditary breast cancer (POSH) cohort and genotyped 206 single nucleotide polymorphisms (SNPs) across 30 candidate genes. After quality control, 899 cases and 133 SNPs remained. Survival analyses were used to identify SNPs associated with prognosis and determine their interdependency with recognized prognostic factors. To identify SNPs that alter breast cancer risk, association tests were used to compare cases with controls from the Wellcome Trust Case Control Consortium. To search for SNPs affecting tumour biology, cases were stratified into subgroups according to oestrogen receptor (ER) status and grade and tested for association.We confirmed previous associations between increased breast cancer risk and SNPs in CASP8, TOX3 (previously known as TNRC9) and ESR1. Analysis of prognosis identified eight SNPs in six genes (MAP3K1, DAPK1, LSP1, MMP7, TOX3 and ESR1) and one region without genes on 8q24 that are associated with survival. For MMP7, TOX3 and MAP3K1 the effects on survival are independent of the main recognized clinical prognostic factors. The SNP in 8q24 is more weakly associated with independent effects on survival. Once grade and pathological nodal status (pN stage) were taken into account, SNPs in ESR1 and LSP1 showed no independent survival difference, whereas the effects of the DAPK1 SNP were removed when correcting for ER status. Interestingly, effects on survival for SNPs in ESR1 were most significant when only ER-positive tumours were examined. Stratifying POSH cases by tumour characteristics identified SNPs in FGFR2 and TOX3 associated with ER-positive disease and SNPs in ATM associated with ER-negative disease.We have demonstrated that several SNPs are associated with survival. In some cases this appears to be due to an effect on tumour characteristics known to have a bearing on prognosis; in other cases the effect appears to be independent of these prognostic factors. These findings require validation by further studies in similar patient groups. 
 Expression of basic fibroblast growth factor, its receptors and syndecans in bladder cancer. Basic fibroblast growth factor (bFGF) is a heparin-binding cationic protein involved in a variety of pathological conditions including angiogenesis and solid tumour growth. The basic fibroblast growth factor receptor (FGFR) family comprises at least 4 high affinity tyrosine kinase receptors that require syndecans for their function. Mounting evidence indicates that syndecans, that bind both bFGF and their FGFRs, will act as stimulators, whereas syndecans that only bind bFGF will act as inhibitors of signaling by sequestering the growth factor. Recent findings have highlighted the importance of syndecans in urological cancers. The aim of this study is to investigate the expression of bFGF, its receptors (R1 and R2) and syndecans (1-4) in   invasive   urothelial carcinoma and normal-looking urothelium by Western blotting, RT-PCR, and immunohistochemistry analyses. Interestingly, bFGF, FGFR1 and FGFR2 protein levels statistically increased in bladder cancer tissues. mRNA of FGFR1 and syndecans (1-4), showed a statistically significant increase while an mRNA increase in the other molecules analysed was not significant. bFGF, its receptors and syndecan immunostaining were mainly present in the urothelium both in normal-looking tissues and urothelial neoplastic cells. In conclusion, our data report that the bFGF, FGFR and syndecan expressions are altered in bladder tumours. 
 The breast cancer susceptibility gene product fibroblast growth factor receptor 2 serves as a scaffold for regulation of NF-κB signaling. Fibroblast growth factor (FGF) receptor 2 (FGFR2) has been identified in genome-wide association studies to be associated with increased breast cancer risk; however, its mechanism of action remains unclear. Here we show that the two major FGFR2 alternatively spliced isoforms, FGFR2-IIIb and FGFR2-IIIc, interact with IκB kinase β and its downstream target, NF-κB. FGFR2 inhibits nuclear RelA/p65 NF-κB translocation and activity and reduces expression of dependent transcripts, including interleukin-6. These interactions result in diminished STAT3 phosphorylation and reduced breast cancer cell growth, motility,   and inv  asiveness. FGFR2 also arrests the epithelial cell-to-mesenchymal cell transition (EMT), resulting in attenuated neoplastic growth in orthotopic xenografts of breast cancer cells. Our studies provide strong evidence for the protective effects of FGFR2 on tumor progression. We propose that FGFR2 serves as a scaffold for multiple components of the NF-κB signaling complex. Through these interactions, FGFR2 isoforms can respond to tissue-specific FGF signals to modulate epithelial cell-stromal cell communications in cancer progression. 
 Fibroblast growth factor receptor (FGFR) gene amplifications are rare events in bladder cancer. Activating point mutations and protein overexpression of fibroblast growth factor receptors (FGFRs), especially FGFR3, are frequent events in bladder cancer. Little is known about gene amplifications, therefore we characterized amplification of FGFR1-3 by fluorescence in-situ hybridization (FISH).Tumours of 153 patients (n = 65 pTa low-grade, n = 15 pTa high-grade, n = 37 pT1, n = 20 pT2, n = 10 pT3, n = 6 pT4) were analysed by FISH for FGFR1-3 copy numbers and screened for FGFR3 mutations and immunohistochemical protein expression. Amplifications of FGFR1 were found in 1.6% (two of 122), FGFR2 in 0.8% (one of 121) and FGFR3 in 3.4% (five of 145). All amplifications were high-level amplifications, not overlapping with polysomy. Amplifications were found in papillar  y/papill  ary-invasive tumour parts, and predominantly in tumours with enhanced Ki67 index (>10%), aberrant CK20 expression, and low p53 expression. All FGFR3-amplified samples showed concomitant FGFR3 mutations and FGFR3 protein overexpression. FGFR amplifications were not associated significantly with gender, age, grade or stage in statistical analyses.FGFR amplifications are rare events in bladder cancer, with FGFR3 amplification being the most prevalent (3.4% of cases). Concomitant FGFR3 mutations and protein overexpression indicate that FGFR3-mediated signalling in these tumours would probably be highly active. This patient subgroup may be particularly suited to FGFR-targeted pharmacotherapy. 
 The breast cancer susceptibility gene product fibroblast growth factor receptor 2 serves as a scaffold for regulation of NF-κB signaling. Fibroblast growth factor (FGF) receptor 2 (FGFR2) has been identified in genome-wide association studies to be associated with increased breast cancer risk; however, its mechanism of action remains unclear. Here we show that the two major FGFR2 alternatively spliced isoforms, FGFR2-IIIb and FGFR2-IIIc, interact with IκB kinase β and its downstream target, NF-κB. FGFR2 inhibits nuclear RelA/p65 NF-κB translocation and activity and reduces expression of dependent transcripts, including interleukin-6. These interactions result in diminished STAT3 phosphorylation and reduced breast cancer cell growth, motility,   and inv  asiveness. FGFR2 also arrests the epithelial cell-to-mesenchymal cell transition (EMT), resulting in attenuated neoplastic growth in orthotopic xenografts of breast cancer cells. Our studies provide strong evidence for the protective effects of FGFR2 on tumor progression. We propose that FGFR2 serves as a scaffold for multiple components of the NF-κB signaling complex. Through these interactions, FGFR2 isoforms can respond to tissue-specific FGF signals to modulate epithelial cell-stromal cell communications in cancer progression. 
 Expression of basic fibroblast growth factor, its receptors and syndecans in bladder cancer. Basic fibroblast growth factor (bFGF) is a heparin-binding cationic protein involved in a variety of pathological conditions including angiogenesis and solid tumour growth. The basic fibroblast growth factor receptor (FGFR) family comprises at least 4 high affinity tyrosine kinase receptors that require syndecans for their function. Mounting evidence indicates that syndecans, that bind both bFGF and their FGFRs, will act as stimulators, whereas syndecans that only bind bFGF will act as inhibitors of signaling by sequestering the growth factor. Recent findings have highlighted the importance of syndecans in urological cancers. The aim of this study is to investigate the expression of bFGF, its receptors (R1 and R2) and syndecans (1-4) in   invasive   urothelial carcinoma and normal-looking urothelium by Western blotting, RT-PCR, and immunohistochemistry analyses. Interestingly, bFGF, FGFR1 and FGFR2 protein levels statistically increased in bladder cancer tissues. mRNA of FGFR1 and syndecans (1-4), showed a statistically significant increase while an mRNA increase in the other molecules analysed was not significant. bFGF, its receptors and syndecan immunostaining were mainly present in the urothelium both in normal-looking tissues and urothelial neoplastic cells. In conclusion, our data report that the bFGF, FGFR and syndecan expressions are altered in bladder tumours. 
 The influence of genetic variation in 30 selected genes on the clinical characteristics of early onset breast cancer. Common variants that alter breast cancer risk are being discovered. Here, we determine how these variants influence breast cancer prognosis, risk and tumour characteristics.We selected 1,001 women with early onset nonfamilial   invasive   breast cancer from the Prospective study of Outcomes in Sporadic versus Hereditary breast cancer (POSH) cohort and genotyped 206 single nucleotide polymorphisms (SNPs) across 30 candidate genes. After quality control, 899 cases and 133 SNPs remained. Survival analyses were used to identify SNPs associated with prognosis and determine their interdependency with recognized prognostic factors. To identify SNPs that alter breast cancer risk, association tests were used to compare cases with controls from the Wellcome Trust Case Control Consortium. To search for SNPs affecting tumour biology, cases were stratified into subgroups according to oestrogen receptor (ER) status and grade and tested for association.We confirmed previous associations between increased breast cancer risk and SNPs in CASP8, TOX3 (previously known as TNRC9) and ESR1. Analysis of prognosis identified eight SNPs in six genes (MAP3K1, DAPK1, LSP1, MMP7, TOX3 and ESR1) and one region without genes on 8q24 that are associated with survival. For MMP7, TOX3 and MAP3K1 the effects on survival are independent of the main recognized clinical prognostic factors. The SNP in 8q24 is more weakly associated with independent effects on survival. Once grade and pathological nodal status (pN stage) were taken into account, SNPs in ESR1 and LSP1 showed no independent survival difference, whereas the effects of the DAPK1 SNP were removed when correcting for ER status. Interestingly, effects on survival for SNPs in ESR1 were most significant when only ER-positive tumours were examined. Stratifying POSH cases by tumour characteristics identified SNPs in FGFR2 and TOX3 associated with ER-positive disease and SNPs in ATM associated with ER-negative disease.We have demonstrated that several SNPs are associated with survival. In some cases this appears to be due to an effect on tumour characteristics known to have a bearing on prognosis; in other cases the effect appears to be independent of these prognostic factors. These findings require validation by further studies in similar patient groups. 
 Assessing interactions between the associations of fibroblast growth factor receptor 2 common genetic variants and hormone receptor status with breast cancer risk. Fibroblast growth factor receptor 2 (FGFR2) is a member of a receptor tyrosine kinase gene superfamily, involved in cell growth,   invasive  ness, motility, and angiogenesis, which has attracted considerable attention as a candidate gene for breast cancer (BC) since it was first identified through genome-wide association approach. In the past few years, the relationship between FGFR2 and BC has been reported in various ethnic groups; however, these studies have yielded contradictory results. To investigate this inconsistency, we performed a meta-analysis of 37 studies involving a total of 288,142 subjects for rs2981582, rs1219648, and rs2420946 polymorphism of the FGFR2 gene to evaluate the effect of FGFR2 on genetic susceptibility for BC. Overall, significantly increased BC risk was associated with these polymorphisms when all studies were pooled into the meta-analysis. In addition, our data indicate that FGFR2 is involved in BC susceptibility and confer its effect primarily in estrogen receptor-positive and progesterone receptor-positive tumors. When stratified by ethnicity, significantly increased risks were found in Caucasian and East Asian populations. However, no significant associations were detected among African descent populations. There was strong evidence of heterogeneity (P < 0.05), which largely disappeared after stratification by ethnicity. This meta-analysis demonstrated that FGFR2 polymorphism is a risk factor associated with increased BC susceptibility, but these associations vary in different ethnic populations. 
 FGFR2 signaling underlies p63 oncogenic function in squamous cell carcinoma. Oncogenic transcription factors drive many human cancers, yet identifying and therapeutically targeting the resulting deregulated pathways has proven difficult. Squamous cell carcinoma (SCC) is a common and lethal human cancer, and relatively little progress has been made in improving outcomes for SCC due to a poor understanding of its underlying molecular pathogenesis. While SCCs typically lack somatic oncogene-activating mutations, they exhibit frequent overexpression of the p53-related transcription factor p63. We developed an in vivo murine tumor model to investigate the function and key transcriptional programs of p63 in SCC. Here, we show that established SCCs are exquisitely dependent on p63, as acute genetic ablation of p63 in advanced,   invasive   SCC induced rapid and dramatic apoptosis and tumor regression. In vivo genome-wide gene expression analysis identified a tumor-survival program involving p63-regulated FGFR2 signaling that was activated by ligand emanating from abundant tumor-associated stroma. Correspondingly, we demonstrate the therapeutic efficacy of extinguishing this signaling axis in endogenous SCCs using the clinical FGFR2 inhibitor AZD4547. Collectively, these results reveal an unanticipated role for p63-driven paracrine FGFR2 signaling as an addicting pathway in human cancer and suggest a new approach for the treatment of SCC. 
 Variation in the FGFR2 gene and the effects of postmenopausal hormone therapy on   invasive   breast cancer. Breast cancer concern is a major reason for the recent marked reduction in use of postmenopausal hormone therapy, although equally effective means of controlling menopausal symptoms are lacking. Single nucleotide polymorphisms (SNP) in the fibroblast growth factor receptor 2 (FGFR2) gene are substantially associated with postmenopausal breast cancer risk and could influence hormone therapy effects.We interrogated eight SNPs in intron 2 of the FGFR2 gene for 2,166   invasive   breast cancer cases from the Women's Health Initiative clinical trial and one-to-one matched controls to confirm an association with breast cancer risk. We used case-only analyses to examine the dependence of estrogen plus progestin and estrogen-alone odds ratios on SNP genotype.Seven FGFR2 SNPs, including six in a single linkage disequilibrium region, were found to associate strongly (P < 10(-7)) with breast cancer risk. SNP rs3750817 (minor allele T with frequency 0.39) had an estimated per-minor-allele odds ratio of 0.78, and was not in such strong linkage disequilibrium with the other SNPs. The genotype of this SNP related significantly (P < 0.05) to hormone therapy odds ratios. For estrogen plus progestin, the odds ratios (95% confidence intervals) at 0, 1, and 2 minor SNP alleles were 1.52 (1.14-2.02), 1.33 (1.01-1.75), and 0.69 (0.41-1.17), whereas the corresponding values for estrogen alone were 0.74 (0.51-1.09), 0.99 (0.68-1.44), and 0.34 (0.15-0.76).Postmenopausal women having TT genotype for SNP rs3750817 have a reduced breast cancer risk and seem to experience comparatively favorable effects of postmenopausal hormone therapy. 
 Decreased expression of keratinocyte growth factor receptor in a subset of human transitional cell bladder carcinomas. Growth factors and growth factor receptors are involved in tumor progression. The fibroblast growth factor receptor 2 gene encodes distinct isoforms. The isoforms which bind KGF (keratinocyte growth factor or FGF-7) are called KGF-R or FGFR2b. KGF-R is expressed in different epithelia and is involved in the control of epithelial-mesenchymal interactions. Expression of KGF-R mRNA was examined in normal human bladder and transitional cell carcinoma of the bladder (TCC) by semi-quantitative RT-PCR using TFIID and GAPDH as internal standards. In normal bladder, the KGF-R mRNA was detected in the urothelium but not in the underlying stroma. In TCCs, the level of KGF-R mRNA was generally either normal or low. Eighteen out of 54 TCCs had a KGF-R mRNA level below 30% of that found in normal urothelium. This decrease in KGF-R mRNA was not accompanied by an increase in BEK (FGFR2c) mRNA, the other major splice variant of the fibroblast growth factor receptor 2 gene. Expression of the KGF-R was also monitored by immunohistochemistry using a functional KGF-immunoglobulin chimera. The receptor was uniformly expressed throughout the normal urothelium except for the umbrella cells. Immunoreactivity for KGF-R was found to be negative in tumors with low levels of KGF-R mRNA, while the peritumoral normal urothelium was positive. Among patients with muscle   invasive   tumors, those exhibiting a low level of KGF-R mRNA had a significantly higher proportion of cancer deaths. Our results suggest that decreased expression of KGF-R can be considered as a marker of tumor progression in muscle   invasive   TCCs. 
 Interaction between FGFR-2, STAT5, and progesterone receptors in breast cancer. Fibroblast growth factor (FGF) receptor 2 (FGFR-2) polymorphisms have been associated with an increase in estrogen receptor and progesterone receptor (PR)-positive breast cancer risk; however, a clear mechanistic association between FGFR-2 and steroid hormone receptors remains elusive. In previous works, we have shown a cross talk between FGF2 and progestins in mouse mammary carcinomas. To investigate the mechanisms underlying these interactions and to validate our findings in a human setting, we have used T47D human breast cancer cells and human cancer tissue samples. We showed that medroxyprogesterone acetate (MPA) and FGF2 induced cell proliferation and activation of ERK, AKT, and STAT5 in T47D and in murine C4-HI cells. Nuclear interaction between PR, FGFR-2, and STAT5 after MPA and FGF2 treatment was also showed by confocal microscopy and immunoprecipitation. This effect was associated with increased transcription of PRE and/or GAS reporter genes, and of PR/STAT5-regulated genes and proteins. Two antiprogestins and the FGFR inhibitor PD173074, specifically blocked the effects induced by FGF2 or MPA respectively. The presence of PR/FGFR-2/STAT5 complexes bound to the PRE probe was corroborated by using NoShift transcription and chromatin immunoprecipitation of the MYC promoter. Additionally, we showed that T47D cells stably transfected with constitutively active FGFR-2 gave rise to   invasive   carcinomas when transplanted into NOD/SCID mice. Nuclear colocalization between PR and FGFR-2/STAT5 was also observed in human breast cancer tissues. This study represents the first demonstration of a nuclear interaction between FGFR-2 and STAT5, as PR coactivators at the DNA progesterone responsive elements, suggesting that FGFRs are valid therapeutic targets for human breast cancer treatment. 
 Novel breast cancer risk alleles and endometrial cancer risk. Genome-wide association studies have identified several novel risk alleles for breast cancer. We hypothesized that genetic variants that are associated with breast cancer, a hormone-related disease, would also be associated with endometrial cancer, another hormone-related disease. We conducted a case-control study nested within the Nurses' Health Study and the Women's Health Study to investigate the associations between these 7 newly identified risk alleles for breast cancer and endometrial cancer risk using 692   invasive   endometrial cancer cases and 1,723 matched controls. We used conditional logistic regression to calculate odds ratios (ORs) and 95% confidence intervals (CIs) to assess the risk of endometrial cancer. In contrast to the breast cancer findings, we did not observe an increased risk of endometrial cancer. We observed an inverse association among rs2981582 (FGFR2) variant carriers [OR= 0.75 (95% CI: 0.60-0.95)]. We also observed a nonsignificant inverse association with rs889312 (MAP3K1) variant carriers [OR = 0.85 (95% CI: 0.68-1.05)] and rs1219648 (FGFR2) variant carriers [OR= 0.86 (95% CI: 0.69-1.06) and endometrial cancer risk. We did not observe associations with the other single nucleotide polymorphisms (SNPs) and endometrial cancer risk. Replication studies investigating these polymorphisms and endometrial cancer risk are warranted. However, our findings do suggest the potential importance of biological differences between endometrial and breast cancer with respect to the genes identified in the scans. The molecular mechanisms underlying these differences remain to be defined. 
 Involvement of fibroblast growth factor receptor 2 isoform switching in mammary oncogenesis. We identified the IIIb C2 epithelial cell-specific splice variant of fibroblast growth factor receptor 2 (FGFR2 IIIb C2) receptor tyrosine kinase in a screen for activated oncogenes expressed in T-47D human breast carcinoma cells. We found FGFR2 IIIb C2 expression in breast carcinoma cell lines and, additionally, expression of the mesenchymal-specific FGFR2 IIIc splice variant in   invasive   breast carcinomas. FGFR2 IIIc expression was associated with loss of epithelial markers and gain of mesenchymal markers. Although FGFR2 IIIb is expressed in epithelial cells, previous studies on FGFR2 IIIb transformation have focused on NIH 3T3 fibroblasts. Therefore, we compared the transforming activities of FGFR2 IIIb C2 in RIE-1 intestinal cells and several mammary epithelial cells. FGFR2 IIIb C2 caused growth transformation of epithelial cells but morphologic transformation of only NIH 3T3 cells. FGFR2 IIIb C2-transformed NIH 3T3, but not RIE-1 cells, showed persistent activation of Ras and increased cyclin D1 protein expression. NIH 3T3 but not RIE-1 cells express keratinocyte growth factor, a ligand for FGFR2 IIIb C2. Ectopic treatment with keratinocyte growth factor caused FGFR2 IIIb C2-dependent morphologic transformation of RIE-1 cells, as well as cyclin D1 up-regulation, indicating that both ligand-independent and stromal cell-derived, ligand-dependent mechanisms contribute to RIE-1 cell transformation. Our results support cell context distinct mechanisms of FGFR2 IIIb C2 transformation. 
 Expression of fibroblast growth factor receptor 2 IIIc in human uterine cervical intraepithelial neoplasia and cervical cancer. Fibroblast growth factor receptors (FGFRs) 1-3 have IIIb and IIIc isoforms, and we reported that FGFR2 IIIb is highly expressed in cervical keratinizing squamous cell carcinoma (SCC). In this study, we determined the expression and roles of FGFR2 IIIc in cervical intraepithelial neoplasia (CIN) and cervical cancer. In CINs 1 and 2, FGFR2 IIIc was found to be localized at the basal to lower two-thirds of the squamous epithelium, whereas it was localized in most of the squamous epithelium, except for the superficial layer in CIN 3. In situ hybridization (ISH) analysis showed that the expression patterns of FGFR2 IIIc mRNA are similar to those of FGFR2 IIIc protein in CINs. The FGFR2 IIIc protein was detected in all   invasive   cervical cancer patients (29 cases) and its mRNA was found to be strongly expressed in the   invasive   front of cancer cell nests. FGFR2 IIIc cDNA was stably transfected into CaSki cells, which are derived from a cervical SCC. The growth rates of the CaSki cells were higher than those of Mock cells in vitro, and the CaSki cells tended to form larger subcutaneous tumors in nude mice. These findings suggest that FGFR2 IIIc plays important roles in carcinogenesis and growth of cervical cancer cells. Anti-FGFR2 IIIc therapies may represent therapeutic strategies for inhibiting the growth of CIN and cervical cancer. 
 A genome-wide association study identifies alleles in FGFR2 associated with risk of sporadic postmenopausal breast cancer. We conducted a genome-wide association study (GWAS) of breast cancer by genotyping 528,173 SNPs in 1,145 postmenopausal women of European ancestry with   invasive   breast cancer and 1,142 controls. We identified four SNPs in intron 2 of FGFR2 (which encodes a receptor tyrosine kinase and is amplified or overexpressed in some breast cancers) that were highly associated with breast cancer and confirmed this association in 1,776 affected individuals and 2,072 controls from three additional studies. Across the four studies, the association with all four SNPs was highly statistically significant (P(trend) for the most strongly associated SNP (rs1219648) = 1.1 x 10(-10); population attributable risk = 16%). Four SNPs at other loci most strongly associated with breast cancer in the initial GWAS were not associated in the replication studies. Our summary results from the GWAS are available online in a form that should speed the identification of additional risk loci. 
 C1GALT1 overexpression promotes the   invasive   behavior of colon cancer cells through modifying O-glycosylation of FGFR2. Core 1 β1,3-galactosyltransferase (C1GALT1) transfers galactose (Gal) to N-acetylgalactosamine (GalNAc) to form Galβ1,3GalNAc (T antigen). Aberrant O-glycans, such as T antigen, are commonly found in colorectal cancer. However, the role of C1GALT1 in colorectal cancer remains unclear. Here we showed that C1GALT1 was frequently overexpressed in colorectal tumors and is associated with poor survival. C1GALT1 overexpression promoted cell survival, migration, invasion, and sphere formation as well as tumor growth and metastasis of colon cancer cells. Conversely, knockdown of C1GALT1 with small interference (si) RNA was sufficient to suppress these malignant phenotypes in vitro and in vivo. Moreover, we are the first to show that fibroblast growth factor receptor (FGFR) 2 carried O-glycans in colon cancer cells. Mechanistic investigations showed that C1GALT1 modified the O-glycans on FGFR2 and enhanced bFGF-triggered activation of FGFR2 as well as increased bFGF-mediated malignant phenotypes. In addition, BGJ398, a selective inhibitor of FGFR, blocked the effects of C1GALT1. These findings suggest that C1GALT1 overexpression modifies O-glycans on FGFR2 and enhances its phosphorylation to promote th  e invasi  ve behavior and cancer stem-like property in colon cancer cells, indicating a critical role of O-glycosylation in the pathogenesis of colorectal cancer. 
 Increased expression of fibroblastic growth factor receptor 2 is correlated with poor prognosis in patients with breast cancer. Although there is growing evidence supporting the hypothesis that fibroblast growth factor receptor 2 (FGFR2) is one of the few candidate genes linked with breast cancer susceptibility, the precise role of FGFR2 protein expression in breast cancer is still unknown. Our study examines FGFR2 protein expression in breast cancer and determines its associations with clinicopathological features and survival.Specimens from 125   invasive   ductal carcinoma grade 2 (IDC2) breast cancer patients were investigated by immunohistochemistry for FGFR2 protein expression. Associations between the expression of FGFR2 and various clinicopathological features as well as survival status were studied.Cytoplasmic and nuclear FGFR2 were expressed in 64.8% and 56.8% of breast cancer patients, respectively. Cytoplasmic FGFR2 expression was significantly associated with tumor size and TNM stage. Furthermore, patients with high expression levels of cytoplasmic and nuclear FGFR2 showed much lower overall survival (OS) and disease-free survival (DFS) rates than those patients with low FGFR2 expression. Cytoplasmic FGFR2 expression and lymph node metastasis were independent prognostic factors for both DFS and OS by multivariate analysis.High FGFR2 expression is correlated with poor OS and DFS in breast cancer patients. It could be a biomarker for poor prognosis. 
 Heterogeneity of breast cancer associations with five susceptibility loci by clinical and pathological characteristics. A three-stage genome-wide association study recently identified single nucleotide polymorphisms (SNPs) in five loci (fibroblast growth receptor 2 (FGFR2), trinucleotide repeat containing 9 (TNRC9), mitogen-activated protein kinase 3 K1 (MAP3K1), 8q24, and lymphocyte-specific protein 1 (LSP1)) associated with breast cancer risk. We investigated whether the associations between these SNPs and breast cancer risk varied by clinically important tumor characteristics in up to 23,039   invasive   breast cancer cases and 26,273 controls from 20 studies. We also evaluated their influence on overall survival in 13,527 cases from 13 studies. All participants were of European or Asian origin. rs2981582 in FGFR2 was more strongly related to ER-positive (per-allele OR (95%CI) = 1.31 (1.27-1.36)) than ER-negative (1.08 (1.03-1.14)) disease (P for heterogeneity = 10(-13)). This SNP was also more strongly related to PR-positive, low grade and node positive tumors (P = 10(-5), 10(-8), 0.013, respectively). The association for rs13281615 in 8q24 was stronger for ER-positive, PR-positive, and low grade tumors (P = 0.001, 0.011 and 10(-4), respectively). The differences in the associations between SNPs in FGFR2 and 8q24 and risk by ER and grade remained significant after permutation adjustment for multiple comparisons and after adjustment for other tumor characteristics. Three SNPs (rs2981582, rs3803662, and rs889312) showed weak but significant associations with ER-negative disease, the strongest association being for rs3803662 in TNRC9 (1.14 (1.09-1.21)). rs13281615 in 8q24 was associated with an improvement in survival after diagnosis (per-allele HR = 0.90 (0.83-0.97). The association was attenuated and non-significant after adjusting for known prognostic factors. Our findings show that common genetic variants influence the pathological subtype of breast cancer and provide further support for the hypothesis that ER-positive and ER-negative disease are biologically distinct. Understanding the etiologic heterogeneity of breast cancer may ultimately result in improvements in prevention, early detection, and treatment. 
 Quantitative assessment of the effect of FGFR2 gene polymorphism on the risk of breast cancer. Fibroblast growth factor receptor 2 is a tyrosine kinase receptor that is a member of the family of individually distinct fibroblast growth factor receptors involved in cell proliferation,   invasive  ness, motility, and angiogenesis. Genome-wide association studies have identified FGFR2 as a breast cancer (BC) susceptibility gene in populations of European and Asian descent. After that, a number of studies reported that the rs2981582, rs1219648, and rs2420946 polymorphism in FGFR2 has been implicated in BC risk. However, studies on the association between these polymorphism and BC remain conflicting. To derive a more precise estimation of the relationship, a meta-analysis of 46,747 cases and 87,342 controls from 16 published case-control studies was performed. Overall, significantly elevated BC risk was associated with rs2981582, rs1219648, and rs2420946 risk allele when all studies were pooled into the meta-analysis. Significant results were also observed in heterozygous and homozygous when compared with wild genotype for these polymorphisms. In the subgroup analysis by ethnicity, source of controls, significantly increased risks were found for these polymorphisms in all genetic model. In conclusion, this meta-analysis suggests that rs2981582, rs1219648, and rs2420946 polymorphisms in FGFR2 are associated with elevated BC risk. 
 Competition between Grb2 and Plcγ1 for FGFR2 regulates basal phospholipase activity and invasion. FGFR2-expressing human cancer cells with low concentrations of the adaptor protein Grb2 show high prevalence for metastatic outcome. In nonstimulated cells, the SH3 domain (and not the SH2 domains) of Plcγ1 directly competes for a binding site at the very C terminus of FGFR2 with the C-terminal SH3 domain of Grb2. Reduction of Grb2 concentration permits Plcγ1 access to the receptor. Recruitment of Plcγ1 in this way is sufficient to upregulate phospholipase activity. This results in elevated phosphatidylinositol 4,5-bisphosphate turnover and intracellular calcium levels, thus leading to increased cell motility and promotion of c  ell-inva  sive behavior in the absence of extracellular receptor stimulation. Therefore, metastatic outcome can be dictated by the constitutive competition between Grb2 and Plcγ1 for the phosphorylation-independent binding site on FGFR2. 
 Genetic variants in fibroblast growth factor receptor 2 (FGFR2) contribute to susceptibility of breast cancer in Chinese women. Fibroblast growth factor receptor 2 (FGFR2) belongs to the FGFR family, which plays an important role in cell growth,   invasive  ness, motility and angiogenesis. In human breast cancer, expression of FGFR2 is estrogen receptor (ER)-dependent and correlates with a lower rate of apoptosis. Recently, whole-genome association studies have identified several single-nucleotide polymorphisms (SNPs) of FGFR2 as novel breast cancer susceptibility loci. In the present study of 1049 breast cancer patients and 1073 cancer-free controls, we assessed whether polymorphisms of FGFR2 are associated with breast cancer risk in Chinese women and whether these associations are stronger in women with a reproductive history suggestive of greater exposure to endogenous estrogens. We genotyped three FGFR2 polymorphisms (rs2981582C/T, rs1219648A/G and rs2420946C/T) using the SNPstream 12-plex platform. Each of the three SNPs was significantly associated with increased breast cancer risk in a dose-dependent manner. Compared with women with 0-2 risk loci, those with 3 risk loci had a 1.36-fold increased odds of breast cancer (95% confidence interval = 1.13-1.62, P = 0.001). In stratified analyses, associations between the presence of 3 risk loci and breast cancer were stronger among women with ER- and/or progesterone receptor-positive cancers, premenopausal women and women with an older age at first live birth. Furthermore, there was a significant additive interaction between risk genotypes and menopausal status (P for multiplication interaction/additive interaction: 0.083/0.037). These findings indicate that genetic variants in FGFR2 may contribute to breast cancer occurrence in Chinese women, possibly through pathways related to estrogen and/or progesterone. 
 Genetic variants associated with breast cancer risk for Ashkenazi Jewish women with strong family histories but no identifiable BRCA1/2 mutation. The ability to establish genetic risk models is critical for early identification and optimal treatment of breast cancer. For such a model to gain clinical utility, more variants must be identified beyond those discovered in previous genome-wide association studies (GWAS). This is especially true for women at high risk because of family history, but without BRCA1/2 mutations. This study incorporates three datasets in a GWAS analysis of women with Ashkenazi Jewish (AJ) homogeneous ancestry. Two independent discovery cohorts comprised 239 and 238 AJ women with   invasive   breast cancer or pre  invasive   ductal carcinoma in situ and strong family histories of breast cancer, but lacking the three BRCA1/2 founder mutations, along with 294 and 230 AJ controls, respectively. An independent, third cohort of 203 AJ cases with familial breast cancer history and 263 healthy controls of AJ women was used for validation. A total of 19 SNPs were identified as associated with familial breast cancer risk in AJ women. Among these SNPs, 13 were identified from a panel of 109 discovery SNPs, including an FGFR2 haplotype. In addition, six previously identified breast cancer GWAS SNPs were confirmed in this population. Seven of the 19 markers were significant in a multivariate predictive model of familial breast cancer in AJ women, three novel SNPs [rs17663555(5q13.2), rs566164(6q21), and rs11075884(16q22.2)], the FGFR2 haplotype, and three previously published SNPs [rs13387042(2q35), rs2046210(ESR1), and rs3112612(TOX3)], yielding moderate predictive power with an area under the curve (AUC) of the ROC (receiver-operator characteristic curve) of 0.74. Population-specific genetic variants in addition to variants shared with populations of European ancestry may improve breast cancer risk prediction among AJ women from high-risk families without founder BRCA1/2 mutations. 
 A combined analysis of genome-wide association studies in breast cancer. In an attempt to identify common disease susceptibility alleles for breast cancer, we performed a combined analysis of three genome-wide association studies (GWAS), involving 2,702 women of European ancestry with   invasive   breast cancer and 5,726 controls. Tests for association were performed for 285,984 SNPs. Evidence for association with SNPs in genes in specific pathways was assessed using a permutation-based approach. We confirmed associations with loci reported by previous GWAS on 1p11.2, 2q35, 3p, 5p12, 8q24, 10q23.13, 14q24.1 and 16q. Six SNPs with the strongest signals of association with breast cancer, and which have not been reported previously, were typed in two further studies; however, none of the associations could be confirmed. Suggestive evidence for an excess of associations was found for genes involved in the regulation of actin cytoskeleton, glycan degradation, alpha-linolenic acid metabolism, circadian rhythm, hematopoietic cell lineage and drug metabolism. Androgen and oestrogen metabolism, a pathway previously found to be associated with the development of postmenopausal breast cancer, was marginally significant (P = 0.051 [unadjusted]). These results suggest that further analysis of SNPs in these pathways may identify associations that would be difficult to detect through agnostic single SNP analyses. More effort focused in these aspects of oncology can potentially open up promising avenues for the understanding of breast cancer and its prevention. 
 Quantification of alternatively spliced FGFR2 RNAs using the RNA   invasive   cleavage assay. The regulated splicing of fibroblast growth factor receptor-2 (FGFR2) transcripts leads to tissue-specific expression of distinct receptor isoforms. These isoforms contain two different versions of the ligand binding Ig-like domain III, which are encoded by exon IIIb or exon IIIc. The mutually exclusive use of exon IIIb and exon IIIc can be recapitulated in tissue culture using DT3 and AT3 rat prostate carcinoma cells. We used this well-characterized system to evaluate the precision and accuracy of the RNA   invasive   cleavage assay to specifically measure FGFR2 alternative splicing outcomes. Experiments presented here demonstrated that the RNA   invasive   cleavage assay could specifically detect isoforms with discrimination levels that ranged from 1 in 5 x 10(3) to 1 in 10(5). Moreover the assay could detect close to 0.01 amole of FGFR2 RNAs. The assay detected the expected levels of transcripts containing either exon IIIb or IIIc, but, surprisingly, it detected high levels of IIIb-IIIc double inclusion transcripts. This finding, which has important implications for the role of exon silencing and of mRNA surveillance mechanisms, had been missed by RT-PCR. Additionally, we used the RNA   invasive   cleavage assay to demonstrate a novel function for the regulatory element IAS2 in repressing exon IIIc inclusion. We also show here that purification of RNA is not necessary for the   invasive   cleavage assay, because crude cell lysates could be used to accurately measure alternative transcripts. The data presented here indicate that the RNA   invasive   cleavage assay is an important addition to the repertoire of techniques available for the study of alternative splicing. 
 Polymorphisms in second intron of the FGFR2 gene are associated with the risk of early-onset breast cancer in Chinese Han women. Fibroblast growth factor receptor 2 (FGFR2) plays an important role in tumor cell growth,   invasive  ness, motility, and angiogenesis. Several single-nucleotide polymorphisms (SNPs) in the second intron of the FGFR2 gene are associated with the risk of breast cancer. In this study, we determined whether these SNPs of the FGFR2 gene are associated with early onset of non-familial breast cancer in a Chinese Han population. Recruited were 118 female breast cancer patients who were less than or equal to 35 years of age and without a family history of breast cancer, and 104 age-matched healthy controls. Six SNPs of the second intron of the FGFR2 gene, including rs2981428C/A (i.e., a change at this particular site from nucleotide C to A), rs11200014G/A, rs2981579C/T, rs1219648A/G, rs2420946C/T, and rs2981582C/T, were detected using matrix-assisted laser desorption/ionization mass spectrometry. The data showed that the homozygotes at each minor allele, rs11200014 (AA), rs1219648 (GG), rs2420946 (TT), and rs2981582 (TT), were significantly associated with an increased risk of early-onset non-familial breast cancer. The haplotype containing rs11200014A, rs1219648G, rs2420946T and rs2981582T also exhibited a significantly higher distribution in patients compared to controls (OR=1.784, 95% CI=1.161-2.744). In stratified analyses, each of the above four SNPs conferred a significantly greater risk of estrogen receptor-positive breast cancer, compared to estrogen receptor-negative breast cancer that is more resistant to treatment. Our data demonstrate that these four SNPs of the FGFR2 gene are associated with the risk of breast cancer at a young age in Chinese Han women. 
 FGFR2 isoforms support epithelial-stromal interactions in thyroid cancer progression. Alternate splicing yields two distinct isoforms of the fibroblast growth factor (FGF) receptor FGFR2-IIIb and FGFR2-IIIc varying their extracellular structure in human thyroid cancer, in which FGFR expression is commonly dysregulated. In this study, we characterized the function of these variants in modulating thyroid cancer behavior. Enforced expression of either FGFR2-IIIb or FGFR2-IIIc in thyroid epithelial cancer cells reduced expression of fibronectin, MAGE-A3 and MMP9, while increasing p21 and enhancing Rb dephosphorylation. Consistent with these tumor-suppressive properties, FGFR2-IIIb and FGFR2-IIIc each diminished   invasive   behavior in vitro and reduced tumor growth and metastasis in vivo. Notably, these effects contrasted with those produced by expression of these FGFR isoforms in fibroblasts, in which they both stimulated cell growth. Moreover, in xenograft tumors generated by coimplantation of epithelial and fibroblast cells expressing that same isoform, there was no significant effect on tumor progression. Conversely, FGFR2-IIIb expression in epithelial cells yielded higher FGF4/FGF7 expression that, in the presence of FGFR2-IIIc-expressing fibroblasts, enhanced tumor progression. Together, our findings highlight the importance of cellular context in assigning growth properties to growth factor receptor isoforms. More specifically, they show how alternative splicing of FGFR2 yields heteroisoforms critical to the growth-promoting actions of FGFs that exert distinct epithelial-stromal effects in thyroid cancer. 
 Overexpressed fibroblast growth factor receptor 2 in the   invasive   front of colorectal cancer: a potential therapeutic target in colorectal cancer. Fibroblast growth factor receptor 2 (FGFR2) is considered a novel therapeutic target for various cancer. We used a silencing strategy to clarify the effect of reduced FGFR2 expression in human colorectal cancer (CRC) cells. The   invasive   front of cancer cells exhibited stronger FGFR2 expression than the surface area of the cancers. FGFR2 shRNA-transfected LoVo cells inhibited cell migration, invasion and tumor growth in vitro and in vivo. Thus, FGFR2 plays important roles in CRC progression in association with tumor cell migration, invasion and growth, and FGFR2 might be a novel therapeutic target for CRC. 
 A multistage genome-wide association study in breast cancer identifies two new risk alleles at 1p11.2 and 14q24.1 (RAD51L1). We conducted a three-stage genome-wide association study (GWAS) of breast cancer in 9,770 cases and 10,799 controls in the Cancer Genetic Markers of Susceptibility (CGEMS) initiative. In stage 1, we genotyped 528,173 SNPs in 1,145 cases of   invasive   breast cancer and 1,142 controls. In stage 2, we analyzed 24,909 top SNPs in 4,547 cases and 4,434 controls. In stage 3, we investigated 21 loci in 4,078 cases and 5,223 controls. Two new loci achieved genome-wide significance. A pericentromeric SNP on chromosome 1p11.2 (rs11249433; P = 6.74 x 10(-10) adjusted genotype test, 2 degrees of freedom) resides in a large linkage disequilibrium block neighboring NOTCH2 and FCGR1B; this signal was stronger for estrogen-receptor-positive tumors. A second SNP on chromosome 14q24.1 (rs999737; P = 1.74 x 10(-7)) localizes to RAD51L1, a gene in the homologous recombination DNA repair pathway. We also confirmed associations with loci on chromosomes 2q35, 5p12, 5q11.2, 8q24, 10q26 and 16q12.1. 
 Transforming potential of alternatively spliced variants of fibroblast growth factor receptor 2 in human mammary epithelial cells. A breast cancer cell line developed in our laboratory (SUM-52PE) has a 12-fold amplification and high-level overexpression of the oncogene fibroblast growth factor receptor 2 (FGFR2). Previously, nine different alternatively spliced FGFR2 variants were isolated from this cell line. Overexpression of two variants that differ only in their carboxyl termini (C1 and C3) has been successfully accomplished in the immortalized human mammary epithelial cell line H16N2. FGFR2 expression led to the activation of the mitogen-activated protein kinase and phosphatidylinositol 3-kinase signaling cascades. Phosphorylation of the adapter protein FGF receptor substrate 2 is much more robust in the cells expressing the C3 variant of FGFR2 compared with the C1 variant. H16N2 cells expressing the full-length FGFR2 with the C1 or C3 carboxyl terminus were tested for their ability to grow under epidermal growth factor (EGF)-independent conditions, in soft agar, and for their ability to invade naturally occurring basement membranes and compared with the parental SUM-52PE cell line. All three cell lines grew under EGF-independent conditions and all were inhibited by the FGFR family specific inhibitor PD173074. The full-length FGFR2-C1 and FGFR2-C3 variants grew robustly in soft agar similar to the parental cell line SUM-52PE. However, cells expressing the C3 variant formed large colonies in agar in both insulin-free and EGF-free medium, whereas the cells expressing the C1 variant required insulin for growth. Soft agar growth was also inhibited by PD173074. Because SUM-52PE was developed from a metastatic breast carcinoma, the FGFR2-overexpressing cell lines were assessed for their ability to invade sea urchin embryo cell membranes. H16N2 cells expressing the C1 carboxyl terminus failed to invade sea urchin embryo cell membranes. By contrast, FGFR2-C3-expressing cells were as   invasive   as the SUM-52 breast cancer cells and erbB-2-overexpressing H16N2 cells. These results indicate that FGFR2 is a transforming oncogene in human mammary epithelial cells when expressed to levels similar to that found in breast cancer cells with FGFR2 gene amplification. Furthermore, the results suggest that different splice variants have differing transforming activities and that signaling from variants expressing the C3 carboxyl terminus results in more autonomous signaling, cell growth, and invasion. 
 The significance of fibroblast growth factor receptor 2 expression in differentiation of hepatocellular carcinoma. Fibroblast growth factor receptors (FGFRs) have been reported to be involved in the progression of many cancers. The aim of this study is to clarify the significance of FGFR2 expression in the differentiation of hepatocellular carcinoma (HCC). One nodule-in-nodule HCC sample was obtained from a patient to analyze the different expression in well- to moderately differentiated HCC and poorly differentiated HCC using microarray technique. The expression of FGFR2 in 46 patients with surgically resected HCC was immunohistochemically examined and analyzed in relation to their clinicopathological factors. Fgfr2 was 4.7 times up-regulated in poorly differentiated HCC from a nodule-in-nodule sample. The high expression group was 16 cases and the low expression group was 30 cases. The high FGFR2 expression correlated significantly with a poor histological differentiation, a higher incidence of portal vein and a high level of alpha-fetoprotein. The overall survival rates and the disease-free survival rates in high expression were significantly worse than those in low. In conclusion, a high FGFR2 expression plays an important role in poor differentiation, portal vein invasion, high alpha-fetoprotein production, and poor prognosis. These data suggest that FGFR2 may be a potentially useful biological marker of tumor   invasive  ness in HCC as well as a novel molecular target for HCC. 
 Variation in the FGFR2 gene and the effect of a low-fat dietary pattern on   invasive   breast cancer. The Women's Health Initiative dietary modification (DM) trial provided suggestive evidence of a benefit of a low-fat dietary pattern on breast cancer risk, with stronger evidence among women whose baseline diet was high in fat. Single nucleotide polymorphisms (SNP) in the FGFR2 gene relate strongly to breast cancer risk and could influence intervention effects.All 48,835 trial participants were postmenopausal and ages 50 to 79 years at enrollment (1993-1998). We interrogated eight SNPs in intron 2 of the FGFR2 gene for 1,676 women who developed breast cancer during trial follow-up (1993-2005). Case-only analyses were used to estimate odds ratios for the DM intervention in relation to SNP genotype.Odds ratios for the DM intervention did not vary significantly with the genotype for any of the eight FGFR2 SNPs (P > or = 0.18). However, odds ratios varied (P < 0.05) with the genotype of six of these SNPs, among women having baseline percent of energy from fat in the upper quartile (> or =36.8%). This variation is most evident for SNP rs3750817, with odds ratios for the DM intervention at 0, 1, and 2 minor SNP alleles of 1.06 [95% confidence intervals (95% CI), 0.80-1.41], 0.53 (95% CI, 0.38-0.74), and 0.62 (95% CI, 0.33-1.15). The nominal significance level for this interaction is P = 0.005, and P = 0.03 following multiple testing adjustment, with most evidence deriving from hormone receptor-positive tumors.  Invasive   breast cancer odds ratios for a low-fat dietary pattern, among women whose usual diets are high in fat, seem to vary with SNP rs3750817 in the FGFR2 gene. 
 Inhibition of fibroblast growth factor receptor signaling impairs metastasis of hepatocellular carcinoma. The molecular mechanism underlying metastasis of hepatocellular carcinoma (HCC) remains elusive. Here, we showed that matrix metalloproteinase (MMP) 7 and MMP26 levels are significantly higher in the resected HCC than in the adjacent healthy hepatic cells from the patients. Moreover, a strong correlation of the levels of MMP7 or MMP26 with the phosphorylated fibroblast growth factor receptor 2 (FGFR2) was detected. To prove a causal link between the activation of FGFR signaling pathway and expression of MMP7 and MMP26, we used two human HCC lines, HepG2 and HuH-7, to study the underlying molecular basis. We found that FGF1-induced FGFR2 phosphorylation in either line resulted in significant activation of MMP7 and MMP26 and consequently an increase in cancer   invasive  ness. Inhibition of FGFR2 phosphorylation in HCC abolished FGF1-stimulated MMP7 and MMP26 expression, suggesting that activation of the FGFR signaling pathway in HCC may promote cancer metastasis by inducing MMP7 and MMP26 expression. To define the signal transduction cascades downstream of FGFR2 activation for MMP7 and MMP26 activation, we applied specific inhibitors for phosphatidylinositol-3 kinase (PI3K), extracellular signal-related kinase/mitogen-activated protein kinase (ERK/MAPK), and Jun N-terminal kinase (JNK), respectively, to the FGF1-stimulated HCC cells. We found that only inhibition of JNK significantly decreased the activation of MMP26 in response to FGF1 stimulation, and only inhibition of PI3K significantly decreased the activation of MMP7 in response to FGF1 stimulation, suggesting that the activation of the FGFR2 signaling may activate PI3K to activate MMP7 and activate JNK to activate MMP26, in HCC. Our study thus highlights the FGFR2 signaling pathway and MMP7 and MMP26 as novel therapeutic targets for HCC. 
 FGFR2 alterations in endometrial carcinoma. Fibroblast growth factor receptor 2 (FGFR2) is a tyrosine kinase receptor involved in many biological processes such as embryogenesis, adult tissue homeostasis and cell proliferation. Mutations in FGFR2 have been reported in up to 10-12% of endometrial carcinomas identical to those found in congenital craniofacial disorders. Inhibition of FGFR2 could be a new therapeutic target in endometrial carcinoma. FGFR2 immunostaining was assessed in three tissue microarrays: one constructed from paraffin-embedded blocks of 60 samples of normal endometrium in different phases of menstrual cycle, and two tissue microarrays containing endometrial carcinoma samples (95 and 62 cases). FGFR2 expression was correlated with stage, histological type and grade as well as with immunostaining of PTEN, RASSF1A, estrogen and progesterone receptors, KI67, Cyclin D1, STAT-3 and SPRY2. FGFR2 mutations were assessed by PCR and direct sequencing, with DNA obtained from 31 paraffin-embedded endometrial carcinoma samples. In normal endometrium, FGFR2 expression was higher in the secretory than in the proliferative phase (P=0.001), with an inverse correlation with Ki67 (P=0.00032), suggesting a tumor-suppressor role for FGFR2 in normal endometrium. Cytoplasmic expression of FGFR2 was higher in endometrial carcinoma when compared with the atrophic endometrium from the same patients (P=0.0283), but was lower in comparison with normal endometrium from women in the menstrual cycle. Interestingly, nuclear staining was observed in some cases, and it was less frequent in endometrial carcinoma when compared with the adjacent atrophic endometrium (P=0.0465). There were no statistical differences when comparing superficial and myo  invasive   endometrial carcinoma samples. Endometrioid endometrial carcinomas showed higher expression of FGFR2 than nonendometrioid endometrial carcinomas (fold change 2.56; P=0.0015). Grade III endometrioid endometrial carcinomas showed decreased FGFR2 expression when compared with grade II endometrioid endometrial carcinomas (P=0.0055). No differences were found regarding pathological stage. Two missense mutations of FGFR2 gene were detected in exons 6 and 11 (S252W and N549K, respectively; 6.45%). Results support the hypothesis that FGFR2 has a dual role in the endometrium, by inhibiting cell proliferation in normal endometrium during the menstrual cycle, but acting as an oncogene in endometrial carcinoma. 
 Kinase domain activation of FGFR2 yields high-grade lung adenocarcinoma sensitive to a Pan-FGFR inhibitor in a mouse model of NSCLC. Somatic mutations in FGFR2 are present in 4% to 5% of patients diagnosed with non-small cell lung cancer (NSCLC). Amplification and mutations in FGFR genes have been identified in patients with NSCLCs, and clinical trials are testing the efficacy of anti-FGFR therapies. FGFR2 and other FGFR kinase family gene alterations have been found in both lung squamous cell carcinoma and lung adenocarcinoma, although mouse models of FGFR-driven lung cancers have not been reported. Here, we generated a genetically engineered mouse model (GEMM) of NSCLC driven by a kinase domain mutation in FGFR2. Combined with p53 ablation, primary grade 3/4 adenocarcinoma was induced in the lung epithelial compartment exhibiting locally   invasive   and pleiotropic tendencies largely made up of multinucleated cells. Tumors were acutely sensitive to pan-FGFR inhibition. This is the first FGFR2-driven lung cancer GEMM, which can be applied across different cancer indications in a preclinical setting. 
 Genetic variants of fibroblast growth factor receptor 2 (FGFR2) are associated with breast cancer risk in Chinese women of the Han nationality. Fibroblast growth factor receptor 2 (FGFR2), a recently described risk factor for breast cancer, plays important roles in cell growth,   invasive  ness, motility, and angiogenesis. In attempt to investigate whether FGFR2 polymorphisms are associated with a risk of breast cancer in Chinese women of the Han nationality, we genotyped single-nucleotide polymorphisms (SNPs) of seven FGFR2 sites (rs2981582, rs17102287, rs17542768, rs10510097, rs11200012, rs3750817, rs2981578) in 816 women including 388 breast cancer patients and 428 healthy controls via the polymerase chain reaction single-strand conformation polymorphism procedure as well as sequence detection. Our results suggest that the A allele and AA genotype of SNP rs2981578 appear to be protective factors associated with breast cancer, while the CT genotype of SNP rs3750817 is a putative risk factor. 
 FGFR2 variants and breast cancer risk: fine-scale mapping using African American studies and analysis of chromatin conformation. Genome-wide association studies have identified FGFR2 as a breast cancer (BC) susceptibility gene in populations of European and Asian descent, but a causative variant has not yet been conclusively identified. We hypothesized that the weaker linkage disequilibrium across this associated region in populations of African ancestry might help refine the set of candidate-causal single nucleotide polymorphisms (SNPs) previously identified by our group. Eight candidate-causal SNPs were evaluated in 1253 African American   invasive   BC cases and 1245 controls. A significant association with BC risk was found with SNP rs2981578 (unadjusted per-allele odds ratio = 1.20, 95% confidence interval 1.03-1.41, P(trend) = 0.02), with the odds ratio estimate similar to that reported in European and Asian subjects. To extend the fine-mapping, genotype data from the African American studies were analyzed jointly with data from European (n = 7196 cases, 7275 controls) and Asian (n = 3901 cases, 3205 controls) studies. In the combined analysis, SNP rs2981578 was the most strongly associated. Five other SNPs were too strongly correlated to be excluded at a likelihood ratio of < 1/100 relative to rs2981578. Analysis of DNase I hypersensitive sites indicated that only two of these map to highly accessible chromatin, one of which, SNP rs2981578, has previously been implicated in up-regulating FGFR2 expression. Our results demonstrate that the association of SNPs in FGFR2 with BC risk extends to women of African American ethnicity, and illustrate the utility of combining association analysis in datasets of diverse ethnic groups with functional experiments to identify disease susceptibility variants. 
 FGFR2, HER2 and cMet in gastric adenocarcinoma: detection, prognostic significance and assessment of downstream pathway activation. Receptor tyrosine kinase pathways are potential therapeutic targets in gastric adenocarcinoma patients. We evaluated HER2 and cMet protein expression, and FGFR2 gene amplification to assess their prognostic significance, and downstream mediators pS6 and pERK for their potential utility as pharmacodynamic biomarkers in patients with gastric adenocarcinoma. Tissue microarrays were constructed from resection samples of 184 patients who underwent surgery for gastric/gastro-oesophageal junction adenocarcinoma. Tissue cores were obtained from the tumour body (TB), luminal surface (LS) and   invasive   edge (IE), and immunohistochemical and fluorescence in situ hybridisation (FGFR2) analysis was performed. FGFR2 amplification was identified in 2 % of cases and associated with worse survival (P = 0.005). HER2 overexpression was observed in 10 % of cases and associated with increased survival (P = 0.041). cMet overexpression was observed in 4 % of cases and associated with worse survival (P < 0.001). On multivariate analysis, only cMet retained significance (P = 0.006). pS6 and pERK expression were observed in 73 % and 30 % of tumours, respectively, with no association with survival. HER2 (P = 0.004) and pERK (P = 0.001) expression differed between tumour regions with HER2 expression increased in the LS compared with the TB and IE. These findings confirm subpopulations in gastric adenocarcinoma with poor outcome that may benefit from specific therapeutic strategies. However, we found heterogeneous HER2, pS6 and pERK overexpression, which presents challenges for their use as predictive biomarkers in gastric biopsies. The potential downstream pharmacodynamic markers pS6 and pERK were expressed across tumour regions, providing evidence that resections and biopsies would yield comparative results in clinical trials. 
  GeneRIFs  
 C1GALT1 overexpression modifies O-glycans on FGFR2 and enhances its phosphorylation to promote the   invasive   behavior and cancer stem-like property in colon cancer cells. 
 FGFR2 may be a potentially useful biological marker of tumor   invasive  ness in hepatocellular carcinoma as well as a novel molecular traget for this disease. 
   Invasive   breast cancer odds ratios for a low-fat dietary pattern, among women whose usual diets are high in fat, seem to vary with SNP rs3750817 in the FGFR2 gene 
 An FGFR2 splice variant is expressed in breast carcinoma cell lines, with a specific splice variant expressesed in   invasive   breast carcinomas. 
  [Mm]etastasis  PubMed  
 Long-Pentraxin 3 Derivative as a Small-Molecule FGF Trap for Cancer Therapy. The fibroblast growth factor (FGF)/FGF receptor (FGFR) system plays a crucial role in cancer by affecting tumor growth, angiogenesis, drug resistance, and escape from anti-angiogenic anti-vascular endothelial growth factor therapy. The soluble pattern recognition receptor long-pentraxin 3 (PTX3) acts as a multi-FGF antagonist. Here we demonstrate that human PTX3 overexpression in transgenic mice driven by the Tie2 promoter inhibits tumor growth, angiogenesis, and   metastasis   in heterotopic, orthotopic, and autochthonous FGF-dependent tumor models. Using pharmacophore modeling of the interaction of a minimal PTX3-derived FGF-binding pentapeptide with FGF2, we identified a small-molecule chemical (NSC12) that acts as an extracellular FGF trap with significant implications in cancer therapy. 
 The VEGFR2, COX-2 and MMP-2 polymorphisms are associated with clinical outcome of patients with inoperable non-small cell lung cancer. Certain common inherited variations in genes involved in tumor angiogenesis, progression and   metastasis   may contribute to cancer therapy outcome and prognosis by altering the gene expression and protein activity. In this report, we examined the effect of functional polymorphisms in MMP-1, MMP-2, MMP-3, VEGF, VEGFR2, FGFR4 and COX-2 genes on overall (OS) and progression-free survival (PFS) of 350 Caucasian patients with inoperable non-small cell lung cancer (NSCLC). The results of multivariate analysis indicated that VEGFR2 -906C and COX-2 -1195G alleles were strongly associated with poor OS and PFS (p = 0.002 and 0.015, respectively, for OS; p = 0.009 and 0.015, respectively, for PFS), while MMP-2 -1306 T allele carriers had significantly reduced PFS (p = 0.010). Moreover, an increased risk of death and progression was significantly associated with the number of adverse alleles for VEGFR2/COX-2 (p = 0.0005 for OS and 0.0006 for PFS in >1 adverse allele carriers) and VEGFR2/COX-2/MMP-2 combinations (p = 0.0003 for OS and 0.0001 for PFS in patients with >2 adverse alleles). Finally, VEGFR2 TC/CC, COX-2 AG/GG and MMP-2 CT/TT genotypes as well as "at risk" allele combinations were identified as independent predictors of unfavorable OS and PFS in the group. In conclusion, the data suggest that selected VEGFR2, COX-2 and MMP-2 polymorphisms may be potential prognostic markers in unresectable NSCLC treated with radiotherapy with or without chemotherapy, although further validation studies are warranted to confirm our observations. 
 Targeting FGFR Pathway in Human Hepatocellular Carcinoma: Expressing pFGFR and pMET for Antitumor Activity. The MET receptor tyrosine kinase, the receptor for hepatocyte growth factor (HGF), has been implicated in cancer growth, invasion, migration, angiogenesis, and   metastasis   in a broad variety of human cancers, including human hepatocellular carcinoma (HCC). Recently, MET was suggested to be a potential target for the personalized treatment of HCC with an active HGF-MET signaling pathway. However, the mechanisms of resistance to MET inhibitors need to be elucidated to provide effective treatment. Here, we show that HCC cells exhibit different sensitivities to the MET inhibitor PHA665752, depending on the phosphorylation status of FGFR. Treatment of cells expressing both phospho-FGFR and phospho-MET with the inhibitor PHA665752 did not cause growth inhibition and cell death, whereas treatment with AZD4547, a pan-FGFR inhibitor, resulted in decreased colony formation and cleavage of caspase-3. Moreover, silencing of endogenous FGFR1 and FGFR2 by RNAi of HCC cells expressing phospho-FGFR, phospho-FGFR2, and phospho-MET overcame the resistance to PHA665752 treatment. Treatment of primary cancer cells from patients with HCC expressing both phospho-FGFR and phospho-MET with PHA665752 did not induce cell death, whereas AZD4547 treatment induced cell death through the cleavage of caspase-3. In addition, treatment of cells resistant to PHA665752 with AZD4547 abrogated the activation of downstream effectors of cell growth, proliferation, and survival. On the basis of these results, we conclude that the FGFR pathway is critical for HCC survival, and that targeting this pathway with AZD4547 may be beneficial for the treatment of patients with HCC-expressing phospho-FGFR and phospho-MET. 
 Targeting FGFR Pathway in Human Hepatocellular Carcinoma: Expressing pFGFR and pMET for Antitumor Activity. The MET receptor tyrosine kinase, the receptor for hepatocyte growth factor (HGF), has been implicated in cancer growth, invasion, migration, angiogenesis, and   metastasis   in a broad variety of human cancers, including human hepatocellular carcinoma (HCC). Recently, MET was suggested to be a potential target for the personalized treatment of HCC with an active HGF-MET signaling pathway. However, the mechanisms of resistance to MET inhibitors need to be elucidated to provide effective treatment. Here, we show that HCC cells exhibit different sensitivities to the MET inhibitor PHA665752, depending on the phosphorylation status of FGFR. Treatment of cells expressing both phospho-FGFR and phospho-MET with the inhibitor PHA665752 did not cause growth inhibition and cell death, whereas treatment with AZD4547, a pan-FGFR inhibitor, resulted in decreased colony formation and cleavage of caspase-3. Moreover, silencing of endogenous FGFR1 and FGFR2 by RNAi of HCC cells expressing phospho-FGFR, phospho-FGFR2, and phospho-MET overcame the resistance to PHA665752 treatment. Treatment of primary cancer cells from patients with HCC expressing both phospho-FGFR and phospho-MET with PHA665752 did not induce cell death, whereas AZD4547 treatment induced cell death through the cleavage of caspase-3. In addition, treatment of cells resistant to PHA665752 with AZD4547 abrogated the activation of downstream effectors of cell growth, proliferation, and survival. On the basis of these results, we conclude that the FGFR pathway is critical for HCC survival, and that targeting this pathway with AZD4547 may be beneficial for the treatment of patients with HCC-expressing phospho-FGFR and phospho-MET. 
 The VEGFR2, COX-2 and MMP-2 polymorphisms are associated with clinical outcome of patients with inoperable non-small cell lung cancer. Certain common inherited variations in genes involved in tumor angiogenesis, progression and   metastasis   may contribute to cancer therapy outcome and prognosis by altering the gene expression and protein activity. In this report, we examined the effect of functional polymorphisms in MMP-1, MMP-2, MMP-3, VEGF, VEGFR2, FGFR4 and COX-2 genes on overall (OS) and progression-free survival (PFS) of 350 Caucasian patients with inoperable non-small cell lung cancer (NSCLC). The results of multivariate analysis indicated that VEGFR2 -906C and COX-2 -1195G alleles were strongly associated with poor OS and PFS (p = 0.002 and 0.015, respectively, for OS; p = 0.009 and 0.015, respectively, for PFS), while MMP-2 -1306 T allele carriers had significantly reduced PFS (p = 0.010). Moreover, an increased risk of death and progression was significantly associated with the number of adverse alleles for VEGFR2/COX-2 (p = 0.0005 for OS and 0.0006 for PFS in >1 adverse allele carriers) and VEGFR2/COX-2/MMP-2 combinations (p = 0.0003 for OS and 0.0001 for PFS in patients with >2 adverse alleles). Finally, VEGFR2 TC/CC, COX-2 AG/GG and MMP-2 CT/TT genotypes as well as "at risk" allele combinations were identified as independent predictors of unfavorable OS and PFS in the group. In conclusion, the data suggest that selected VEGFR2, COX-2 and MMP-2 polymorphisms may be potential prognostic markers in unresectable NSCLC treated with radiotherapy with or without chemotherapy, although further validation studies are warranted to confirm our observations. 
 Epithelial splicing regulatory protein 1 is a favorable prognostic factor in pancreatic cancer that attenuates pancreatic metastases. Epithelial splicing regulatory protein 1 (ESRP1) binds the FGFR-2 auxiliary cis-element ISE/ISS-3, located in the intron between exon IIIb and IIIc, and primarily promotes FGFR-2 IIIb expression. Here we assessed the role of ESRP1 in pancreatic ductal adenocarcinoma (PDAC). Immunohistochemical analysis was performed using anti-ESRP1, FGFR-2 IIIb and FGFR-2 IIIc antibodies in 123 PDAC cases. ESRP1 expression vector and small interference RNA (siRNA) targeting ESRP1 were transfected into human PDAC cells, and cell growth, migration and invasion were analyzed. In vivo heterotopic and orthotopic implantations using ESRP1 overexpression clones were performed and effects on pancreatic tumor volumes and hepatic and pulmonary metastases determined. ESRP1 immunoreactivity was strong in the nuclei of cancer cells in well-to-moderately differentiated PDACs but weak in poorly differentiated cancers. Well-to-moderately differentiated cancers also exhibited high FGFR-2 IIIb and low FGFR-2 IIIc expression, whereas this ratio was reversed in the poorly differentiated cancers. Increased ESRP1 expression was associated with longer survival in comparison with low ESRP1 expression, and PANC-1 cells engineered to express ESRP1 exhibited increased FGFR-2 IIIb expression and decreased migration and invasion in vitro, whereas ESRP1 siRNA-transfected KLM-1 cells exhibited increased FGFR-2 IIIc expression and increased cell growth, migration and invasion. In vivo, ESRP1-overexpressing clones formed significantly fewer liver metastases as compared with control clones. ESRP1 regulates the expression pattern of FGFR-2 isoforms, attenuates cell growth, migration, invasion and   metastasis  , and is a favorable prognostic factor in PDAC. Therefore, devising mechanisms to upregulate ESRP1 may exert a beneficial therapeutic effect in PDAC. 
 Inhibition of fibroblast growth factor receptor 2 attenuates proliferation and invasion of pancreatic cancer. The alternative splicing of the extracellular domain of fibroblast growth factor receptor (FGFR)-2 generates the IIIb and IIIc isoforms. Expression of FGFR-2 IIIb correlates with vascular endothelial growth factor-A (VEGF-A) expression and venous invasion of pancreatic ductal adenocarcinoma (PDAC). By contrast, FGFR-2 IIIc expression correlates with faster development of liver   metastasis   after surgery, and increased proliferation rates and invasion of the cancer. In this study, we analyzed the expression and roles of total FGFR-2 (both isoforms) to determine the effectiveness of FGFR-2-targeting therapy for PDAC. Immunohistochemically, FGFR-2 was highly expressed in 25/48 (52.1%) PDAC cases, and correlated with advanced stage cancer. In FISH analysis, FGFR2 was amplified in 3/7 PDAC cell lines. We stably transfected an FGFR-2 shRNA targeting the IIIb and IIIc isoforms into FGFR2-amplified PDAC cells. The proliferation rates, migration, and invasion of FGFR-2-shRNA-transfected cells were lower than those of control cells in vitro. In response to FGF-2, FGFR-2-shRNA-transfected cells showed decreased phosphorylation of ERK compared with control cells. The FGFR-2-shRNA-transfected cells also expressed lower levels of vascular endothelial growth factor-A than control cells, and formed smaller s.c. tumors in nude mice. These findings suggest that FGFR-2 is a therapeutic target for inhibition in PDAC. 
 Altered expression of fibroblast growth factor receptor 2 isoform IIIc: relevance to endometrioid adenocarcinoma carcinogenesis and histological differentiation. Fibroblast growth factor receptor 2 (FGFR2) is activated in many cancers and considered as a potential therapeutic molecular target including for endometrial endometrioid carcinoma (EEC). Overexpression of FGFR2 isoform IIIc (FGFR2IIIc) has been shown to be associated with carcinogenesis in various cancers, but its expression in EEC has not been reported yet to the best of our knowledge. In this study, we identified roles for FGFR2IIIc in EEC carcinogenesis and demonstrated its diagnostic and prognostic values in EEC. FGFR2IIIc expression was compared between 10 normal endometrium, 10 atypical endometrial hyperplasias, and 47 EEC specimens using immunohistochemistry and quantitative real-time PCR. Atypical hyperplasia, Grade 1 (G1), and Grade 2 (G2) differentiated EEC tissues showed significantly higher FGFR2IIIc expression than normal endometrium tissue. However, as compared to G1 and G2 EECs, Grade 3 (G3) differentiated EEC tissue showed lower FGFR2IIIc expression (P<0.05). There was no significant association between FGFR2IIIc expression and patient age, lymph node   metastasis  , and EEC stage. These results suggest that altered FGFR2IIIc expression plays an important role in EEC carcinogenesis and may occur in precancerous tissues. However, FGFR2IIIc appears to be not related to EEC progression. Some G3 EECs may develop through different carcinogenic processes than G1 and G2 EECs. 
 Fibroblast growth factor receptor 2 IIIc as a therapeutic target for colorectal cancer cells. A high percentage of colorectal carcinomas overexpress a lot of growth factors and their receptors, including fibroblast growth factor (FGF) and FGF receptor (FGFR). We previously reported that FGFR2 overexpression was associated with distant   metastasis   and that FGFR2 inhibition suppressed cell growth, migration, and invasion. The FGFR2 splicing isoform FGFR2IIIb is associated with well-differentiated histologic type, tumor angiogenesis, and adhesion to extracellular matrices. Another isoform, FGFR2IIIc, correlates with the aggressiveness of various types of cancer. In the present study, we examined the expression and roles of FGFR2IIIc in colorectal carcinoma to determine the effectiveness of FGFR2IIIc-targeting therapy. In normal colorectal tissues, FGFR2IIIc expression was weakly detected in superficial colorectal epithelial cells and was not detected in proliferative zone cells. FGFR2IIIc-positive cells were detected by immunohistochemistry in the following lesions, listed in the order of increasing percentage: hyperplastic polyps < low-grade adenomas < high-grade adenomas < carcinomas. FGFR2IIIc immunoreactivity was expressed in 27% of colorectal carcinoma cases, and this expression correlated with distant   metastasis   and poor prognosis. FGFR2IIIc-transfected colorectal carcinoma cells showed increased cell growth, soft agar colony formation, migration, and invasion, as well as decreased adhesion to extracellular matrices. Furthermore, FGFR2IIIc-transfected colorectal carcinoma cells formed larger tumors in subcutaneous tissues and the cecum of nude mice. Fully human anti-FGFR2IIIc monoclonal antibody inhibited the growth and migration of colorectal carcinoma cells through alterations in cell migration, cell death, and development-related genes. In conclusion, FGFR2IIIc plays an important role in colorectal carcinogenesis and tumor progression. Monoclonal antibody against FGFR2IIIc has promising potential in colorectal carcinoma therapy. 
 Conditional activation of fibroblast growth factor receptor (FGFR) 1, but not FGFR2, in prostate cancer cells leads to increased osteopontin induction, extracellular signal-regulated kinase activation, and in vivo proliferation. Changes in the fibroblast growth factor receptor (FGFR) axis are often associated with prostate cancer (CaP) progression. We have used chemically induced dimerization (CID) to elucidate the individual contributions of FGFR1 and FGFR2 to tumor etiology. Novel CaP cell lines stably expressing CID/AP20187-inducible FGFR1 (iFGFR1) and iFGFR2 were made using the tumorigenic transgenic adenocarcinoma of the murine prostate (TRAMP)-derived clone, TRAMP-C2N (C2N), to generate C2N.iFGFR1 or C2N.iFGFR2 cells. To test the effects of iFGFR activation on tumor growth, mice bearing s.c. C2N.iFGFR1- or C2N.iFGFR2-derived tumors were treated biweekly with CID. Activation of iFGFR1 led to rapid tumor growth as a result of increased proliferation. In contrast, expression of iFGFR2 inhibited tumor growth. Furthermore, we have ascertained that FGFR1 activation appears to be most important during the early stages of tumor development, but once established, tumors become rapidly CID independent. In these C2N-based lines, quantitative signaling differences were seen between the two receptors, with iFGFR1 leading to more robust extracellular signal-regulated kinase activation. Additionally, activation of iFGFR1, but not iFGFR2, led to strong up-regulation of osteopontin, a secreted glycoprotein involved in integrin activation and associated with CaP progression and   metastasis  . These studies support the hypothesis that observed changes in the FGFR axis in mammals during CaP progression are causally important. 
 NGF/γ-IFN inhibits androgen-independent prostate cancer and reverses androgen receptor function through downregulation of FGFR2 and decrease in cancer stem cells. Androgen-independent prostate cancer (AIPC) is difficult to treat. Present study is to explore the inhibitory effect of a cytokine environment on AIPC and its mechanism. We utilized nerve growth factor (NGF)/γ-interferon (γ-IFN) to change the cytokine environment. Animal models and 2 androgen receptor (AR)-negative prostate cancer cell lines were used to evaluate the effect of NGF/γ-IFN. Flow cytometry, immunocytochemistry, western blotting, Tunel assay, colony formation efficiency, gene microarray, and in vivo bioluminescence were used to discern the mechanisms within NGF/γ-IFN that effect the environment. In vitro, NGF/γ-IFN effectively inhibited the proliferation of AIPC cell lines and promoted the apoptosis of the cancer cells. In vivo, NGF/γ-IFN suppressed the grow  th and met  astasis of a tumor mass that arose from the AIPC cell line. After NGF/γ-IFN treatment, the AR-negative cell lines re-expressed AR and were then able to respond to the androgen. Contrary to expectations, the proliferation of cells was inhibited after dihydrotestosterone was added, and the results indicated that NGF/γ-IFN decreased the proportion of cancer stem cells. NGF/γ-IFN worked mainly through the downregulation of fibroblast growth factor receptor 2. 
 C1GALT1 overexpression promotes the invasive behavior of colon cancer cells through modifying O-glycosylation of FGFR2. Core 1 β1,3-galactosyltransferase (C1GALT1) transfers galactose (Gal) to N-acetylgalactosamine (GalNAc) to form Galβ1,3GalNAc (T antigen). Aberrant O-glycans, such as T antigen, are commonly found in colorectal cancer. However, the role of C1GALT1 in colorectal cancer remains unclear. Here we showed that C1GALT1 was frequently overexpressed in colorectal tumors and is associated with poor survival. C1GALT1 overexpression promoted cell survival, migration, invasion, and sphere formation as well as tumor growth an  d metastas  is of colon cancer cells. Conversely, knockdown of C1GALT1 with small interference (si) RNA was sufficient to suppress these malignant phenotypes in vitro and in vivo. Moreover, we are the first to show that fibroblast growth factor receptor (FGFR) 2 carried O-glycans in colon cancer cells. Mechanistic investigations showed that C1GALT1 modified the O-glycans on FGFR2 and enhanced bFGF-triggered activation of FGFR2 as well as increased bFGF-mediated malignant phenotypes. In addition, BGJ398, a selective inhibitor of FGFR, blocked the effects of C1GALT1. These findings suggest that C1GALT1 overexpression modifies O-glycans on FGFR2 and enhances its phosphorylation to promote the invasive behavior and cancer stem-like property in colon cancer cells, indicating a critical role of O-glycosylation in the pathogenesis of colorectal cancer. 
 Increased expression of fibroblastic growth factor receptor 2 is correlated with poor prognosis in patients with breast cancer. Although there is growing evidence supporting the hypothesis that fibroblast growth factor receptor 2 (FGFR2) is one of the few candidate genes linked with breast cancer susceptibility, the precise role of FGFR2 protein expression in breast cancer is still unknown. Our study examines FGFR2 protein expression in breast cancer and determines its associations with clinicopathological features and survival.Specimens from 125 invasive ductal carcinoma grade 2 (IDC2) breast cancer patients were investigated by immunohistochemistry for FGFR2 protein expression. Associations between the expression of FGFR2 and various clinicopathological features as well as survival status were studied.Cytoplasmic and nuclear FGFR2 were expressed in 64.8% and 56.8% of breast cancer patients, respectively. Cytoplasmic FGFR2 expression was significantly associated with tumor size and TNM stage. Furthermore, patients with high expression levels of cytoplasmic and nuclear FGFR2 showed much lower overall survival (OS) and disease-free survival (DFS) rates than those patients with low FGFR2 expression. Cytoplasmic FGFR2 expression and lymph node   metastasis   were independent prognostic factors for both DFS and OS by multivariate analysis.High FGFR2 expression is correlated with poor OS and DFS in breast cancer patients. It could be a biomarker for poor prognosis. 
 Anti-PlGF inhibits growth of VEGF(R)-inhibitor-resistant tumors without affecting healthy vessels. Novel antiangiogenic strategies with complementary mechanisms are needed to maximize efficacy and minimize resistance to current angiogenesis inhibitors. We explored the therapeutic potential and mechanisms of alphaPlGF, an antibody against placental growth factor (PlGF), a VEGF homolog, which regulates the angiogenic switch in disease, but not in health. alphaPlGF inhibited growth and   metastasis   of various tumors, including those resistant to VEGF(R) inhibitors (VEGF(R)Is), and enhanced the efficacy of chemotherapy and VEGF(R)Is. alphaPlGF inhibited angiogenesis, lymphangiogenesis, and tumor cell motility. Distinct from VEGF(R)Is, alphaPlGF prevented infiltration of angiogenic macrophages and severe tumor hypoxia, and thus, did not switch on the angiogenic rescue program responsible for resistance to VEGF(R)Is. Moreover, it did not cause or enhance VEGF(R)I-related side effects. The efficacy and safety of alphaPlGF, its pleiotropic and complementary mechanism to VEGF(R)Is, and the negligible induction of an angiogenic rescue program suggest that alphaPlGF may constitute a novel approach for cancer treatment. 
 Fibroblast growth factor receptor 2 gene amplification status and its clinicopathologic significance in gastric carcinoma. Fibroblast growth factor receptor 2 is a member of receptor tyrosine kinase family, and fibroblast growth factor receptor 2 gene amplification or missense mutation has been observed in various human cancers, including gastric carcinoma. Recent studies have shown that anti-fibroblast growth factor receptor 2 agents inhibit tumor progression in various human cancers, such as endometrial carcinoma and gastric carcinoma, which remains one of the most frequent causes of cancer-related death worldwide. We considered that knowledge of the status of fibroblast growth factor receptor 2 gene amplification in gastric carcinoma might aid in targeted cancer therapy. In this study, fibroblast growth factor receptor 2 amplification status was evaluated by fluorescence in situ hybridization in 313 surgically resected gastric carcinoma tissues, and the results were validated by quantitative real-time polymerase chain reaction. In addition, potential associations between clinicopathologic parameters and the presence of fibroblast growth factor receptor 2 amplification were investigated, and survival analysis was performed. Of the 313 cases, 14 (4.5%) showed fibroblast growth factor receptor 2 amplification by fluorescence in situ hybridization. Fibroblast growth factor receptor 2 amplification was found to be associated with a higher pT stage (P = .023), higher pN stage (P = .038), and distant   metastasis   (P = .009) and to be significantly associated with lower cancer-specific survival by univariate analysis (P = .012). Gastric carcinoma with fibroblast growth factor receptor 2 amplification was found to be associated with advanced disease and a poor prognosis. We believe that the determination of fibroblast growth factor receptor 2 amplification status could allow the identification of a subset of cancers sensitive to targeted fibroblast growth factor receptor 2 inhibitor-based therapy. 
 Peritoneal metastatic lesions of gastric cancer exhibit low expression of human epidermal growth factor receptor 2. The prognosis of gastric cancer patients with peritoneal   metastasis   is very poor. Recent findings suggest that use of trastuzumab, a monoclonal antibody-based agent that targets human epidermal growth factor receptor 2 (HER2), may improve the prognosis of gastric cancer patients with HER2 overexpression and/or gene amplification. However, whether these mechanisms of HER2 upregulation are present in gastric cancer patients with peritoneal   metastasis   is unclear. The status of HER2 expression in a cohort of samples obtained from 35 gastric cancer patients with peritoneal   metastasis   was investigated using immunohistochemistry and fluorescence in situ hybridization. In 18 cases, we also investigated the influence of induction chemotherapy on HER2 overexpression. The frequency of HER2 overexpression and gene amplification was 2.9 % (1/35) in peritoneal metastatic lesions. There was concurrence in HER2 status in the samples examined prior to and following induction of chemotherapy. Most samples from the gastric cancer patients with peritoneal   metastasi  s did not show HER2 amplification and/or overexpression. Although our study size was small, these results suggest that trastuzumab, which is critically dependent on HER2 expression, might not be an effective agent for these patients. Consequently, other therapeutic approaches for these patients must be developed. 
 Risk of aggressive breast cancer in women of Han nationality carrying TGFB1 rs1982073 C allele and FGFR2 rs1219648 G allele in North China. The high morbidity and aggressive behavior of breast cancer are associated with genetic variations. Single nucleotide polymorphisms (SNPs) in many genes have been demonstrated as a risk factor for breast cancer. In this study, we evaluated the association of the SNP rs1982073 (Leu10Pro) in transforming growth factor-beta 1 (TGFB1) gene and the SNP rs1219648 in fibroblast growth factor receptor 2 (FGFR2) gene with the risk and aggressiveness of breast cancer among women of Han nationality in North China. TaqMan allelic discrimination assay was performed to genotype rs1982073 and rs1219648 in 447 breast cancer cases and 406 age-matched healthy controls. The results imply that genotype frequencies of rs1982073 and rs1219648 were not significantly different between breast cancer patients and healthy controls. However, for TGFB1 SNP rs1982073, the C-carriers (T/C+C/C genotype) were more likely to bear tumors of greater aggressiveness (histological grade III) than T/T-carriers (grade I-II) (OR = 3.45, 95% CI: 1.48-8.00, P = 0.004). For FGFR2 SNP rs1219648, G-carriers (A/G+G/G genotype) were more significantly linked to tumors with lymph node   metastasis   than those with A/A genotype (lymph node negative) (OR = 1.69, 95% CI: 1.11-2.58, P = 0.016). Therefore, the SNPs of TGFB1 rs1982073 and FGFR2 rs1219648 may contribute to the identification of patients with more aggressive breast cancers among Han women in North China. 
 FGFR2 isoforms support epithelial-stromal interactions in thyroid cancer progression. Alternate splicing yields two distinct isoforms of the fibroblast growth factor (FGF) receptor FGFR2-IIIb and FGFR2-IIIc varying their extracellular structure in human thyroid cancer, in which FGFR expression is commonly dysregulated. In this study, we characterized the function of these variants in modulating thyroid cancer behavior. Enforced expression of either FGFR2-IIIb or FGFR2-IIIc in thyroid epithelial cancer cells reduced expression of fibronectin, MAGE-A3 and MMP9, while increasing p21 and enhancing Rb dephosphorylation. Consistent with these tumor-suppressive properties, FGFR2-IIIb and FGFR2-IIIc each diminished invasive behavior in vitro and reduced tumor growth and   metastasis   in vivo. Notably, these effects contrasted with those produced by expression of these FGFR isoforms in fibroblasts, in which they both stimulated cell growth. Moreover, in xenograft tumors generated by coimplantation of epithelial and fibroblast cells expressing that same isoform, there was no significant effect on tumor progression. Conversely, FGFR2-IIIb expression in epithelial cells yielded higher FGF4/FGF7 expression that, in the presence of FGFR2-IIIc-expressing fibroblasts, enhanced tumor progression. Together, our findings highlight the importance of cellular context in assigning growth properties to growth factor receptor isoforms. More specifically, they show how alternative splicing of FGFR2 yields heteroisoforms critical to the growth-promoting actions of FGFs that exert distinct epithelial-stromal effects in thyroid cancer. 
 One patient, two lesions, two oncogenic drivers of gastric cancer. Deep-sequencing of a primary tumor and   metastasis   from a single patient, and functional validation in culture, reveals that TGFBR2 and FGFR2 act as drivers of gastric cancer. 
 Enhanced expression of fibroblast growth factor receptor 2 IIIc promotes human pancreatic cancer cell proliferation. In pancreatic ductal adenocarcinoma (PDAC), the fibroblast growth factor receptor 1 (FGFR-1) IIIb isoform correlates with the inhibition of cancer cell proliferation, migration, and invasion, whereas FGFR-1 IIIc enhances cancer cell proliferation. The FGFR-2 IIIb isoform is expressed in PDAC, and its expression correlates with increased venous invasion. We examined the role of FGFR-2 IIIc in PDAC. FGFR-2 IIIc was expressed in all six pancreatic cancer cell lines examined and was highest in PANC-1 cells. FGFR-2 IIIc was abundant in the cancer cells from 83 of 117 PDAC cases, which correlated with decreased duration to development of liver   metastasis   after surgery. FGFR-2 IIIc-transfected cells exhibited increased proliferation in vitro and formed larger subcutaneous and orthotopic tumors, the latter producing more liver metastases. Moreover, FGF-2 exerted a more rapid stimulatory effect on the levels of phosphorylated extracellular signal-regulated kinase (p-ERK) in FGFR-2 IIIc stably transfected PANC-1 cells, compared with control cells. FGFR-2 IIIc-transfected cells also formed more spheres and contained more side population cells. Suppression of FGFR-2 IIIc expression inhibited the proliferation of PANC-1 cells, whereas an anti-FGFR-2 IIIc antibody inhibited the proliferation and migration of PANC-1 cells. Thus, high FGFR-2 IIIc levels in PDAC contribute to disease aggressiveness and confer to pancreatic cancer cells features suggestive of cancer stem cells, indicating that FGFR-2 IIIc may be a novel and important therapeutic target in PDAC. 
 Microarray analysis identifies a death-from-cancer signature predicting therapy failure in patients with multiple types of cancer. Activation in transformed cells of normal stem cells' self-renewal pathways might contribute to the survival life cycle of cancer stem cells and promote tumor progression. The BMI-1 oncogene-driven gene expression pathway is essential for the self-renewal of hematopoietic and neural stem cells. We applied a mouse/human comparative translational genomics approach to identify an 11-gene signature that consistently displays a stem cell-resembling expression profile in distant metastatic lesions as revealed by the analysis of metastases and primary tumors from a transgenic mouse model of prostate cancer and cancer patients. To further validate these results, we examined the prognostic power of the 11-gene signature in several independent therapy-outcome sets of clinical samples obtained from 1,153 cancer patients diagnosed with 11 different types of cancer, including 5 epithelial malignancies (prostate, breast, lung, ovarian, and bladder cancers) and 5 nonepithelial malignancies (lymphoma, mesothelioma, medulloblastoma, glioma, and acute myeloid leukemia). Kaplan-Meier analysis demonstrated that a stem cell-like expression profile of the 11-gene signature in primary tumors is a consistent powerful predictor of a short interval to disease recurrence, distant   metastasis  , and death after therapy in cancer patients diagnosed with 11 distinct types of cancer. These data suggest the presence of a conserved BMI-1-driven pathway, which is similarly engaged in both normal stem cells and a highly malignant subset of human cancers diagnosed in a wide range of organs and uniformly exhibiting a marked propensity toward metastatic dissemination as well as a high probability of unfavorable therapy outcome. 
 Inhibition of fibroblast growth factor receptor signaling impairs   metastasis   of hepatocellular carcinoma. The molecular mechanism underlying   metastasis   of hepatocellular carcinoma (HCC) remains elusive. Here, we showed that matrix metalloproteinase (MMP) 7 and MMP26 levels are significantly higher in the resected HCC than in the adjacent healthy hepatic cells from the patients. Moreover, a strong correlation of the levels of MMP7 or MMP26 with the phosphorylated fibroblast growth factor receptor 2 (FGFR2) was detected. To prove a causal link between the activation of FGFR signaling pathway and expression of MMP7 and MMP26, we used two human HCC lines, HepG2 and HuH-7, to study the underlying molecular basis. We found that FGF1-induced FGFR2 phosphorylation in either line resulted in significant activation of MMP7 and MMP26 and consequently an increase in cancer invasiveness. Inhibition of FGFR2 phosphorylation in HCC abolished FGF1-stimulated MMP7 and MMP26 expression, suggesting that activation of the FGFR signaling pathway in HCC may promote cancer   metastasis   by inducing MMP7 and MMP26 expression. To define the signal transduction cascades downstream of FGFR2 activation for MMP7 and MMP26 activation, we applied specific inhibitors for phosphatidylinositol-3 kinase (PI3K), extracellular signal-related kinase/mitogen-activated protein kinase (ERK/MAPK), and Jun N-terminal kinase (JNK), respectively, to the FGF1-stimulated HCC cells. We found that only inhibition of JNK significantly decreased the activation of MMP26 in response to FGF1 stimulation, and only inhibition of PI3K significantly decreased the activation of MMP7 in response to FGF1 stimulation, suggesting that the activation of the FGFR2 signaling may activate PI3K to activate MMP7 and activate JNK to activate MMP26, in HCC. Our study thus highlights the FGFR2 signaling pathway and MMP7 and MMP26 as novel therapeutic targets for HCC. 
 FGFR2 intronic SNPs and breast cancer risk: associations with tumor characteristics and interactions with exogenous exposures and other known breast cancer risk factors. Recent genome-wide association studies have revealed several new candidate genes for breast cancer, including fibroblast growth factor receptor 2 (FGFR2) gene. The associations were also replicated in several other independent studies. The next important step is to study whether these common variants interact with known breast cancer risk factors, exogenous exposures and tumor characteristics. In a population-based case-control study of 1,170 breast cancer cases and 2,115 controls, we examined genetic associations of four intronic FGFR2 single-nucleotide polymorphisms (SNPs) and breast tumor characteristics and assessed the potential interactions with smoking, alcohol consumption, adiposity and known breast cancer risk factors. FGFR2 variants were significantly associated with breast cancer risk regardless of estrogen and progesterone receptor status,   metastasis  , lymph node involvement and histologic and nuclear grade. The FGFR2-breast cancer association was modified by smoking status, with increased risk for former and current smokers compared to never smokers; former/current smokers carrying two copies of the rs1219648 minor allele were at highest risk with a crude OR (95% confidence interval) of 2.11 (1.52-2.92) compared to never smokers with no rs1219648 variant alleles. Our study found no evidence for either modification of FGFR2 and breast cancer by alcohol intake or adiposity, even when analyses were stratified by menopausal status. Although these results require further replication, they may provide new insight into the possible new exposures that may interact with FGFR2 susceptibility alleles. 
 The melanoma-associated antigen A3 mediates fibronectin-controlled cancer progression and   metastasis  . Tumor cells frequently exhibit decreased adhesiveness due to failure to deposit stromal fibronectin (FN), permitting more rapid proliferation, migration, invasion, and   metastasis  . Although up-regulation of FN has been noted in gene profiles of carcinomas compared with normal tissue, reduced FN expression has been described at the peripheral margins of invading tumors. In this study, we investigate the role of FN in cancer behavior. Using human thyroid carcinoma cells with stably down-regulated FN, we performed gene profiling and created an orthotopic mouse model. We stably overexpressed the FN target, MAGE A3, which has also been identified as a target of the breast cancer risk factor fibroblast growth factor receptor 2, and examined the functional effects in vitro and in vivo in a flank model and an orthotopic model of thyroid cancer. Mouse xenografts showed significantly enhanced tumor growth as well as larger and more numerous lung metastases in response to FN silencing. Gene profiling identified the melanoma-associated antigen (MAGE A3) as significantly up-regulated in response to FN silencing. Forced expression of MAGE A3 resulted in p21 down-regulation, accelerated cell cycle progression, increased cell migration rate, and invasion in vitro and in vivo in an orthotopic mouse model where microcomputed tomography confirmed lung metastases that recapitulate the progression of human thyroid cancer. We conclude that MAGE A3 is a functional integrator of diverse signals, including FGFR2 and FN, to modulate cancer progression. 
 Fibroblast growth factor receptor 2 overexpression is predictive of poor prognosis in rectal cancer patients receiving neoadjuvant chemoradiotherapy. Neoadjuvant concurrent chemoradiotherapy (CCRT) followed by surgery is an increasingly used therapeutic strategy for advanced rectal cancer, but risk stratification and final outcomes remain suboptimal. Recently, the oncogenic role of the fibroblast growth factor/fibroblast growth factor receptor (FGFR) signalling pathway has been recognised; however, its clinical significance in rectal cancer has not been elucidated. In this study, we identify and validate targetable drivers associated with the FGFR signalling pathway in rectal cancer patients treated with CCRT.Using a published transcriptome of rectal cancers, we found FGFR2 gene significantly predicted response to CCRT. The expression levels of FGFR2, using immunohistochemistry assays, were further evaluated in 172 rectal cancer specimens that had not received any treatment. Expression levels of FGFR2 were statistically correlated with major clinicopathological features and clinical survival in this valid cohort.High expression of FGFR2 was significantly related to advanced pretreatment tumour (p=0.022) and nodal status (p=0.026), post-treatment tumour (p<0.001) and nodal status (p=0.004), and inferior tumour regression grade (p<0.001). In survival analyses, high expression of FGFR2 was significantly associated with shorter local recurrence-free survival (p=0.0001),   metastasis  -free survival (MeFS; p=0.0003) and disease-specific survival (DSS; p<0.0001). Notably, high expression of FGFR2 was independently predictive of worse outcomes for MeFS (p=0.002, HR=5.387) and DSS (p=0.004, HR=4.997).High expression of FGFR2 is correlated with advanced tumour stage, poor therapeutic response and worse survival in rectal cancer patients receiving neoadjuvant CCRT. These findings indicate that FGFR2 is a prognostic factor for treating rectal cancer. 
  GeneRIFs  
 Deep-sequencing of a primary tumor and   metastasis   from a single patient, and functional validation in culture, reveals that FGFR2 and TGFBR2 act as drivers of gastric cancer in primary and   metastasis   respectively. 
 High FGFR2 phosphorylation is associated with   metastasis   of hepatocellular carcinoma. 
 Results show that ESRP1 regulates the expression pattern of FGFR-2 isoforms, attenuates cell growth, migration, invasion and   metastasis  , and is a favorable prognostic factor in pancreatic ductal adenocarcinoma. 
 Most samples from the gastric cancer patients with peritoneal   metastasis   did not show human epidermal growth factor receptor 2 (HER2) amplification and/or overexpression. 
  [Cc]ell [Ii]nvasion  GeneRIFs  
 FGFR2 plays an important role in colorectal cancer progression in association with tumor   cell invasion   and growth, and FGFR2 might be a novel therapeutic target for CRC 
   
  
 TIE1 
 
  NCBI Gene  
  GeneRIF  
  Pubmed  
 
 
  Search Term  Rank  Fields  
  [Mm]igration  6  Process(1); PubMed(2); Conserved Domains(1)  
  [Ee]xtracellular [Mm]atrix  8  PubMed(1)  
  [Mm]etastasis  12  PubMed(1)  
 
 
 hide/show details for TIE1 
  [Mm]igration  Process  
 negative regulation of cell   migration   
  PubMed  
 Tie1-Tie2 interactions mediate functional differences between angiopoietin ligands. The Tie family of endothelial-specific receptor tyrosine kinases is essential for cell proliferation,   migration  , and survival during angiogenesis. Despite considerable similarity, experiments with Tie1- or Tie2-deficient mice highlight distinct functions for these receptors in vivo. The Tie2 receptor is further unique with respect to its structurally homologous ligands. Angiopoietin-2 and -3 can function as agonists or antagonists; angiopoietin-1 and -4 are constitutive agonists. To address the role of Tie1 in angiopoietin-mediated Tie2 signaling and determine the basis for the behavior of the individual angiopoietins, we used an in vivo FRET-based proximity assay to monitor Tie1 and -2 localization and association. We provide evidence for Tie1-Tie2 complex formation on the cell surface and identify molecular surface areas essential for receptor-receptor recognition. We further demonstrate that the Tie1-Tie2 interactions are dynamic, inhibitory, and differentially modulated by angiopoietin-1 and -2. Based on the available data, we propose a unified model for angiopoietin-induced Tie2 signaling. 
 Tie1-Tie2 interactions mediate functional differences between angiopoietin ligands. The Tie family of endothelial-specific receptor tyrosine kinases is essential for cell proliferation,   migration  , and survival during angiogenesis. Despite considerable similarity, experiments with Tie1- or Tie2-deficient mice highlight distinct functions for these receptors in vivo. The Tie2 receptor is further unique with respect to its structurally homologous ligands. Angiopoietin-2 and -3 can function as agonists or antagonists; angiopoietin-1 and -4 are constitutive agonists. To address the role of Tie1 in angiopoietin-mediated Tie2 signaling and determine the basis for the behavior of the individual angiopoietins, we used an in vivo FRET-based proximity assay to monitor Tie1 and -2 localization and association. We provide evidence for Tie1-Tie2 complex formation on the cell surface and identify molecular surface areas essential for receptor-receptor recognition. We further demonstrate that the Tie1-Tie2 interactions are dynamic, inhibitory, and differentially modulated by angiopoietin-1 and -2. Based on the available data, we propose a unified model for angiopoietin-induced Tie2 signaling. 
  Conserved Domains  
 cd00055: EGF_Lam; Laminin-type epidermal growth factor-like domain; laminins are the major noncollagenous components of basement membranes that mediate cell adhesion, growth   migration  , and differentiation; the laminin-type epidermal growth factor-like module occurs in ... 
  [Ee]xtracellular [Mm]atrix  PubMed  
 A secreted protein microarray platform for extracellular protein interaction discovery. Characterization of the extracellular protein interactome has lagged far behind that of intracellular proteins, where mass spectrometry and yeast two-hybrid technologies have excelled. Improved methods for identifying receptor-ligand and   extracellular matrix   protein interactions will greatly accelerate biological discovery in cell signaling and cellular communication. These technologies must be able to identify low-affinity binding events that are often observed between membrane-bound coreceptor molecules during cell-cell or cell-  extracellular matrix   contact. Here we demonstrate that functional protein microarrays are particularly well-suited for high-throughput screening of extracellular protein interactions. To evaluate the performance of the platform, we screened a set of 89 immunoglobulin (Ig)-type receptors against a highly diverse extracellular protein microarray with 686 genes represented. To enhance detection of low-affinity interactions, we developed a rapid method to assemble bait Fc fusion proteins into multivalent complexes using protein A microbeads. Based on these screens, we developed a statistical methodology for hit calling and identification of nonspecific interactions on protein microarrays. We found that the Ig receptor interactions identified using our methodology are highly specific and display minimal off-target binding, resulting in a 70% true-positive to false-positive hit ratio. We anticipate that these methods will be useful for a wide variety of functional protein microarray users. 
  [Mm]etastasis  PubMed  
 Vasculogenic mimicry and pseudo-comedo formation in breast cancer. Tumors require a blood supply for growth and hematogenous metastases. Until recently, most research in this area has focused on the role of angiogenesis, the recruitment of new vessels into a tumor from preexisting vessels. Previously, in a study of breast cancer (IBC), in which we used established inflammatory breast cancer (IBC) xenografts (WIBC-9) originating from a patient with IBC (Shirakawa et al., Cancer Res 2001:61:445-451), we reported observing vasculogenic mimicry (VM), a condition in which bloodstreams within cancer tissue are not accompanied by a lining of endothelial cells (ECs) (Shirakawa et al., Cancer Res 2002:62:560-566). In the present study, we examined 331 surgically resected breast cancer specimens for evidence of VM, using immunohistochemistry and laser-captured microdissection (LCM) followed by nested reverse transcriptase polymerase chain reaction (RT-PCR). Surprisingly, 7.9% (26 specimens) of the 331 specimens exhibited evidence of VM. Of these 26 VM specimens, 84.6% (22 specimens) exhibited pseudo-comedo formation. RT-PCR analysis of 8 microdissected typical VM specimens revealed expression of Tie-2, Flt-1, thrombin receptor and CD31 in 63, 50, 0 and 0% of specimens, respectively. In contrast, results of RT-PCR analysis of 8 specimens from non-VM tumors were negative for expression of these genes. The 26 VM cases tended to have a higher percentage of hematogenous recurrence (p = 0.059) and a lower percentage of 5-year survival (p = 0.071) than the 305 non-VM cases. However, there were no significant differences in tumor size, lymph node   metastasis  , estrogen receptors or progesterone receptors between the 2 groups (p > 0.1). Our results suggest that the existence of VM increases the likelihood of hematogenous metastases and is in inverse proportion to prognosis. 
   
  
 KIT 
 
  NCBI Gene  
  GeneRIF  
  Pubmed  
 
 
  Search Term  Rank  Fields  
  [Mm]igration  6  Process(4); PubMed(22)  
  ECM  7  PubMed(1)  
  [Ee]xtracellular [Mm]atrix  8  PubMed(4)  
  [Ii]nvasive  10  PubMed(22); GeneRIFs(1)  
  [Mm]etastasize  11  PubMed(4)  
  [Mm]etastasis  12  PubMed(58); GeneRIFs(8)  
  [Cc]ell [Ii]nvasion  13  PubMed(3); GeneRIFs(1)  
 
 
 hide/show details for KIT 
  [Mm]igration  Process  
 germ cell   migration   
 hematopoietic stem cell   migration   
 melanocyte   migration   
 positive regulation of cell   migration   
  PubMed  
 STAT protein recruitment and activation in c-Kit deletion mutants. Stem cell factor (SCF) and its tyrosine kinase receptor, c-Kit, play a crucial role in regulating   migration   and proliferation of melanoblasts, germ cells, and hemopoietic cell progenitors by activating a number of intracellular signaling molecules. Here we report that SCF stimulation of myeloid cells or fibroblasts ectopically expressing c-Kit induces physical association with and tyrosine phosphorylation of three signal transducers and activators of transcription (STATs) as follows: STAT1alpha, STAT5A, and STAT5B. Other STAT proteins are not recruited upon SCF stimulation. Recruitment of STATs leads to their dimerization, nuclear translocation, and binding to specific promoter-responsive elements. Whereas STAT1alpha, possibly in the form of homodimers, binds to the sis-inducible DNA element, STAT5 proteins, either as STAT5A/STAT5B or STAT5/STAT1alpha heterodimers, bind to the prolactin-inducible element of the beta-casein promoter. The tyrosine kinase activity of Kit appears essential for STAT activation since a kinase-defective mutant lacking a kinase insert domain was inactive in STAT signaling. However, another mutant that lacked the carboxyl-terminal region retained STAT1alpha activation and nuclear translocation but was unable to fully activate STAT5 proteins, although it mediated their transient phosphorylation. These results indicate that different intracellular domains of c-Kit are involved in activation of the various STAT proteins. 
 Stem cell factor induces phosphatidylinositol 3'-kinase-dependent Lyn/Tec/Dok-1 complex formation in hematopoietic cells. Stem cell factor (SCF) has an important role in the proliferation, differentiation, survival, and   migration   of hematopoietic cells. SCF exerts its effects by binding to cKit, a receptor with intrinsic tyrosine kinase activity. Activation of phosphatidylinositol 3'-kinase (PI3-K) by cKit was previously shown to contribute to many SCF-induced cellular responses. Therefore, PI3-K-dependent signaling pathways activated by SCF were investigated. The PI3-K-dependent activation and phosphorylation of the tyrosine kinase Tec and the adapter molecule p62Dok-1 are reported. The study shows that Tec and Dok-1 form a stable complex with Lyn and 2 unidentified phosphoproteins of 56 and 140 kd. Both the Tec homology and the SH2 domain of Tec were identified as being required for the interaction with Dok-1, whereas 2 domains in Dok-1 appeared to mediate the association with Tec. In addition, Tec and Lyn were shown to phosphorylate Dok-1, whereas phosphorylated Dok-1 was demonstrated to bind to the SH2 domains of several signaling molecules activated by SCF, including Abl, CrkL, SHIP, and PLCgamma-1, but not those of Vav and Shc. These findings suggest that p62Dok-1 may function as an important scaffold molecule in cKit-mediated signaling. 
 Repression of c-Kit by p53 is mediated by miR-34 and is associated with reduced chemoresistance,   migration   and stemness. The c-Kit receptor tyrosine kinase is commonly over-expressed in different types of cancer. p53 activation is known to result in the down-regulation of c-Kit. However, the underlying mechanism has remained unknown. Here, we show that the p53-induced miR-34 microRNA family mediates repression of c-Kit by p53 via a conserved seed-matching sequence in the c-Kit 3'-UTR. Ectopic miR-34a resulted in a decrease in Erk signaling and transformation, which was dependent on the down-regulation of c-Kit expression. Furthermore, ectopic expression of c-Kit conferred resistance of colorectal cancer (CRC) cells to treatment with 5-fluorouracil (5-FU), whereas ectopic miR-34a sensitized the cells to 5-FU. After stimulation with c-Kit ligand/stem cell factor (SCF) Colo320 CRC cells displayed increased   migration  /invasion, whereas ectopic miR-34a inhibited SCF-induced   migration  /invasion. Activation of a conditional c-Kit allele induced several stemness markers in DLD-1 CRC cells. In primary CRC samples elevated c-Kit expression also showed a positive correlation with markers of stemness, such as Lgr5, CD44, OLFM4, BMI-1 and β-catenin. On the contrary, activation of a conditional miR-34a allele in DLD-1 cells diminished the expression of c-Kit and several stemness markers (CD44, Lgr5 and BMI-1) and suppressed sphere formation. MiR-34a also suppressed enhanced sphere-formation after exposure to SCF. Taken together, our data establish c-Kit as a new direct target of miR-34 and demonstrate that this regulation interferes with several c-Kit-mediated effects on cancer cells. Therefore, this regulation may be potentially relevant for future diagnostic and therapeutic approaches. 
 Germ cell specific expression of c-kit in the human fetal gonad. The proto-oncogene receptor, c-kit, and its ligand have been demonstrated to be essential to the processes of germ cell   migration  , proliferation and survival in the rodent. The aim of the present study was to investigate the expression of c-kit mRNA and protein in human fetal ovary and testis across the gestational period 13-21 weeks. In the ovary, this crucial period of development spans the transition from oogonial replication by mitosis to primordial follicle formation. In the testis, germ cells (gonocytes) are mitotically active. Expression of c-kit mRNA was demonstrated by reverse transcription-polymerase chain reaction (RT-PCR) in both ovary and testis at all gestational ages examined. Testicular germ cell specific expression of c-kit mRNA was confirmed by RT-PCR using specific cell types recovered by laser capture microscopy. The expression of c-kit protein by both male and female germ cells was demonstrated by immunohistochemistry at all gestational ages examined, and was confirmed by immunoblotting. In both, c-kit was localized to the cell membrane except in oocytes within primordial follicles where it was localized to the cytoplasm. These data demonstrate that the expression of c-kit mRNA and protein is germ cell specific in human fetal gonads and are consistent with an important role for the c-kit/kit ligand signalling system in germ cell proliferation and survival in the developing human gonad. 
 Piebaldism and neurofibromatosis type 1: family report. Piebaldism is a rare disorder present at birth and inherited as an autosomal dominant trait. It results from a mutation in the c-kit proto-oncogene and is associated with a defect in the   migration   and differentiation of melanoblasts from the neural crest. Clinical manifestations and phenotypic severity strongly correlates with the site of mutation within the KIT gene. Here we report a 3-year-old boy and his 33-year-old father with leukoderma and poliosis associated with clinical criteria for Neurofibromatosis type 1. Genetic study of both revealed a p.Gly610Asp mutation in the KIT gene. This familiar mutation has not yet been reported in the literature. There are rare reports of piebaldism in association with neurofibromatosis type I. 
 Bone-induced c-kit expression in prostate cancer: a driver of intraosseous tumor growth. Loss of BRCA2 function stimulates prostate cancer (PCa) cell invasion and is associated with more aggressive and metastatic tumors in PCa patients. Concurrently, the receptor tyrosine kinase c-kit is highly expressed in skeletal metastases of PCa patients and induced in PCa cells placed into the bone microenvironment in experimental models. However, the precise requirement of c-kit for intraosseous growth of PCa and its relation to BRCA2 expression remain unexplored. Here, we show that c-kit expression promotes   migration   and invasion of PCa cells. Alongside, we found that c-kit expression in PCa cells parallels BRCA2 downregulation. Gene rescue experiments with human BRCA2 transgene in c-kit-transfected PCa cells resulted in reduction of c-kit protein expression and   migration   and invasion, suggesting a functional significance of BRCA2 downregulation by c-kit. The inverse association between c-kit and BRCA2 gene expressions in PCa cells was confirmed using laser capture microdissection in experimental intraosseous tumors and bone metastases of PCa patients. Inhibition of bone-induced c-kit expression in PCa cells transduced with lentiviral short hairpin RNA reduced intraosseous tumor incidence and growth. Overall, our results provide evidence of a novel pathway that links bone-induced c-kit expression in PCa cells to BRCA2 downregulation and supports bone metastasis. 
 The MAPK pathway functions as a redundant survival signal that reinforces the PI3K cascade in c-Kit mutant melanoma. Stimulation of the c-Kit receptor tyrosine kinase has a critical role in the development and   migration   of melanocytes, and oncogenic c-Kit mutants contribute to the progression of some melanomas. c-Kit signalling activates the mitogen-activated protein kinase (MAPK) and phosphatidylinositol 3-kinase (PI3K) pathways and their relative contribution to the activities of oncogenic and ligand-dependent c-Kit remains uncertain. We show that PI3K is a major regulator of MAPK activation in response to c-Kit activity and the dominant effector of c-Kit-driven melanocyte proliferation and melanoma survival. Nevertheless, inhibition of the PI3K pathway in c-Kit mutant melanoma cells did not replicate the apoptotic efficacy of the c-Kit inhibitor, imatinib mesylate. Instead, the simultaneous suppression of the PI3K and MAPK pathways promoted a strong synergistic apoptotic effect. These data indicate that MAPK functions as a redundant survival signal that reinforces the PI3K cascade in c-Kit mutant melanoma. Thus, the concurrent inhibition of PI3K and MAPK signalling is required to suppress oncogenic c-Kit activity and may provide an effective therapeutic strategy in c-Kit mutant melanomas. 
 Identification of the c-kit gene mutations in biopsy tissues of mammary gland carcinoma tumor. C-kit gene is a transmembrane tyrosine kinase that acts as type III receptor for mast cell growth factor and cellular   migration  , proliferation, survival of melanoblasts, haematopoietic progenitors and primordial germ cells. Apart from the scant information about the pathologies associated with loss-of-function mutations, few reports have proposed role of the c-kit gene in case of carcinogenesis. Apparently, in breast cancer the involvement of c-kit gene mutations has been considered as a rare phenomenon. Thus, we designed our study with aim to investigate the c-kit gene mutation in breast cancer, and their correlation with clinico-pathological findings. We performed mutational analysis of the c-kit gene in 58 cases of malignant breast cancer. With the aim to ascertain the variety of mutations at exon 8, 9, 11, 13, 15 and 17 of c-kit gene in breast cancer, we have done PCR-SSCP followed by DNA sequencing. In breast cancer the c-kit gene mutation rates were 3.44% (02/58) in exon 8, 5.17% (3/58) in exon 9, 5.17% (3/58) in exon 11, 3.44% (2/580 in exon 13, 3.44% (2/58) in exon 15 and 5.17% (3/58) in exon 17, respectively. The overall c-kit mutation frequency in exons 8, 9, 11, 13, 15 and 17 was determined to be 25.86% (15/58). Our study indicates to specify the role of c-kit proto-oncogene mutation in breast cancer. The result signifies that c-kit gene plays a poor role in prognosis of ductal and lobular carcinoma. 
 Induction of microRNA-221 by platelet-derived growth factor signaling is critical for modulation of vascular smooth muscle phenotype. The platelet-derived growth factor (PDGF) signaling pathway is a critical regulator of animal development and homeostasis. Activation of the PDGF pathway leads to neointimal proliferative responses to artery injury; it promotes a switch of vascular smooth muscle cells (vSMC) to a less contractile phenotype by inhibiting the SMC-specific gene expression and increasing the rate of proliferation and   migration  . The molecular mechanism for these pleiotropic effects of PDGFs has not been fully described. Here, we identify the microRNA-221 (miR-221), a small noncoding RNA, as a modulator of the phenotypic change of vSMCs in response to PDGF signaling. We demonstrate that miR-221 is transcriptionally induced upon PDGF treatment in primary vSMCs, leading to down-regulation of the targets c-Kit and p27Kip1. Down-regulation of p27Kip1 by miR-221 is critical for PDGF-mediated induction of cell proliferation. Additionally, decreased c-Kit causes inhibition of SMC-specific contractile gene transcription by reducing the expression of Myocardin (Myocd), a potent SMC-specific nuclear coactivator. Our study demonstrates that PDGF signaling, by modulating the expression of miR-221, regulates two critical determinants of the vSMC phenotype; they are SMC gene expression and cell proliferation. 
 Screening of the c-kit gene missense mutation in invasive ductal carcinoma of breast among north Indian population. Breast cancer is one of the most frequently diagnosed cancers and the leading cause of cancer deaths among females across the world, accounting for 23 % (1.38 million) of total new cancer cases and 14 % (0.45 million) of the total cancer deaths in 2008. c-kit is expressed in mast cell growth factor, cellular   migration  , proliferation, melanoblasts, haematopoietic progenitors and germ cells. We have designed our study with aim to explore the c-kit gene mutations in invasive ductal carcinoma (IDC) breast. To ascertain the range of mutations in exon 11, 13 and 17 of c-kit gene in 53 cases of IDC breast, we carried out PCR-SSCP followed by DNA sequencing. The mutation frequency of c-kit gene in exon 11, 13 and 17 were 9.43 % (5/53), 1.88 % (1/53) and 3.77 % (2/53), respectively. During our mutational analysis, we have detected five missense mutations in exon 11 (Pro551Leu, Glu562Val, Leu576Phe, His580Tyr and Phe584Leu), one missense mutation in exon 13 (Ser639Pro) and two missense mutations in exon 17 (Arg796Gly and Asn822Ser). It seems that c-kit mutations might participate in breast cancer pathogenesis and may be utilized as predictive marker, since the loss of c-kit positivity is generally linked with different types of breast cancer. Further molecular studies are necessary to validate the association of c-kit gene mutation in IDC breast pathogenesis. 
 Stem cell factor/c-Kit signalling in normal and androgenetic alopecia hair follicles. Androgens stimulate many hair follicles to alter hair colour and size via the hair growth cycle; in androgenetic alopecia tiny, pale hairs gradually replace large, pigmented ones. Since stem cell factor (SCF) is important in embryonic melanocyte   migration   and maintaining adult rodent pigmentation, we investigated SCF/c-Kit signalling in human hair follicles to determine whether this was altered in androgenetic alopecia. Quantitative immunohistochemistry detected three melanocyte-lineage markers and c-Kit in four focus areas: the epidermis, infundibulum, hair bulb (where pigment is formed) and mid-follicle outer root sheath (ORS). Colocalisation confirmed melanocyte c-Kit expression; cultured follicular melanocytes also exhibited c-Kit. Few ORS cells expressed differentiated melanocyte markers or c-Kit, but NKI/beteb antibody, which also recognises early melanocyte-lineage antigens, identified fourfold more cells, confirmed by colocalisation. Occasional similar bulbar cells were seen. Melanocyte distribution, concentration and c-Kit expression were unaltered in balding follicles. Androgenetic alopecia cultured dermal papilla cells secreted less SCF, measured by ELISA, than normal cells. This identifies three types of melanocyte-lineage cells in human follicles. The c-Kit expression by dendritic, pigmenting, bulbar melanocytes and rounded, differentiated, non-pigmenting ORS melanocytes implicate SCF in maintaining pigmentation and   migration   into regenerating hair bulbs. Less differentiated, c-Kit-independent cells in the mid-follicle ORS stem cell niche and occasionally in the bulb, presumably a local reserve for long scalp hair growth, implicate other factors in activating stem cells. Androgens appear to reduce alopecia hair colour by inhibiting dermal papilla SCF production, impeding bulbar melanocyte pigmentation. These results may facilitate new treatments for hair colour changes in hirsutism, alopecia or greying. 
 CD117-positive cells in adult human heart are localized in the subepicardium, and their activation is associated with laminin-1 and alpha6 integrin expression. CD117-positive cells contributing to cardiac cell turnover in normal and pathological conditions have recently been described in adult human heart. Since the precise spatial and temporal expression of extracellular matrix proteins and their receptors is critical for organ formation, we compared the distribution of cardiac primitive CD117-positive cells in the human adult normal and pathological hearts with ischemic cardiomyopathy, with respect to localization and expression of laminin and integrin isoforms. In the pathological hearts, CD117-positive cells were significantly more numerous than in the normal hearts. They were localized mainly in the atria and were up to 38-fold more numerous in the subepicardium than in the myocardium. Compared with normal hearts, most CD117-positive cells in the subepicardium of pathological hearts were alpha(6) integrin-positive. Laminin-1, typical of developing heart, was found predominantly in the subepicardium of adult heart. Immunoblotting revealed its highest expression in the normal atrium and pathological left ventricle. Both laminin isoforms reduced apoptosis and increased proliferation and   migration   of CD117-positive cells in vitro with respect to control, but the effects of laminin-1 significantly outweighed those of laminin-2. Signaling mediated by alpha(6) integrin was implicated in the   migration   and protection from apoptosis, as documented by transfection with specific small interfering RNA. These data reveal that the increase in the number of cardiac CD117-positive cells and the expression of laminin-1 are observed in ischemic cardiomyopathy. Subepicardial localization of CD117-positive cells and expression of laminin-1 and alpha(6) integrin subunits may all correspond to the activation of regeneration involving an epithelial-mesenchymal transition recently described in adult heart. 
 The usefulness of c-Kit in the immunohistochemical assessment of melanocytic lesions. C-Kit (CD117), the receptor for the stem cell factor, a growth factor for melanocyte   migration   and proliferation, has shown differential immunostaining in various benign and malignant melanocytic lesions. The purpose of this study is to compare c-Kit immunostaining in benign nevi and in primary and metastatic malignant melanomas, to determine whether c-Kit can aid in the differential diagnosis of these lesions. c-Kit immunostaining was performed in 60 cases of pigmented lesions, including 39 benign nevi (5 blue nevi, 5 intradermal nevi, 3 junctional nevi, 15 cases of primary compound nevus, 11 cases of Spitz nevus), 18 cases of primary malignant melanoma and 3 cases of metastatic melanoma. The vast majority of nevi and melanomas examined in this study were positive for c-Kit, with minimal differences between benign and malignant lesions. C-Kit cytoplasmatic immunoreactivity in the intraepidermal proliferating nevus cells, was detected in benign pigmented lesions as well as in malignant melanoma, increasing with the age of patients (P=0.007) in both groups. The patient's age at presentation appeared to be the variable able to cluster benign and malignant pigmented lesions. The percentage of c-Kit positive intraepidermal nevus cells was better associated with age despite other variables (P=0.014). The intensity and percentage of c-Kit positivity in the proliferating nevus cells in the dermis was significantly increased in malignant melanocytic lesions (P=0.015 and P=0.008) compared to benign lesions (compound melanocytic nevi, Spitz nevi, intradermal nevi, blue nevi). Immunostaning for c-Kit in metastatic melanomas was negative. Interestingly in two cases of melanoma occurring on a pre-existent nevus, the melanoma tumor cells showed strong cytoplasmatic and membranous positivity for c-kit, in contrast with the absence of any immunoreactivity in pre-existent intradermal nevus cells. C-Kit does not appear to be a strong immunohistochemical marker for distinguishing melanoma from melanocytic nevi, if we consider c-Kit expression in intraepidermal proliferating cells. The c-Kit expression in proliferating melanocytes in the dermis could help in the differential diagnosis between a superficial spreading melanoma (with dermis invasion) and a compound nevus or an intradermal nevus. Finally, c-Kit could be a good diagnostic tool for distinguishing benign compound nevi from malignant melanocytic lesions with dermis invasion and to differentiate metastatic melanoma from primary melanoma. 
 [Hereditary hypomelanocytoses: the role of PAX3, SOX10, MITF, SNAI2, KIT, EDN3 and EDNRB genes]. Hypo- and hyperpigmentation disorders are the most severe dermatological diseases observed in patients from all over the world. These disorders can be divided into melanoses connected with disorders of melanocyte function and melanocytoses connected with melanocyte development. The article presents some hereditary hypomelanocytoses, which are caused by abnormal melanoblast development,   migration   and proliferation as well as by abnormal melanocyte viability and proliferation. These disorders are represented by Waardenburg syndrome, piebaldism and Tietz syndrome, and are caused by different mutations of various or the same genes. The types of mutations comprise missense and nonsense mutations, frameshifts (in-frame insertions or deletions), truncating variations, splice alterations and non-stop mutations. It has been demonstrated that mutations of the same gene may cause different hypopigmentation syndromes that may have similar phenotypes. For example, mutations of the MITF gene cause Waardenburg syndrome type 2A as well as Tietz syndrome. It has also been demonstrated that mutations of different genes may cause an identical syndrome. For example, mutations of MITF, SNAI2 and SOX10 genes are observed in Waardenburg syndrome type II and mutations of EDNRB, EDN3 and SOX10 genes are responsible for Waardenburg syndrome type IV. In turn, mutation of the KIT gene and/or heterozygous deletion of the SNAI2 gene result in piebaldism disease. The knowledge of the exact mechanisms of pigmentary disorders may be useful in the development of new therapeutic approaches to their treatment. 
 Structural and functional analysis of KIT gene encoding receptor tyrosine kinase and its interaction with sunitinib and HDAC inhibitors: an in silico approach. KIT is a growth factor receptor, important for normal germ cell   migration   and development. The malfunction of KIT gene results in constitutive activation of the tyrosine kinase activity of c-KIT which is believed to be the major oncogenic event in stomach, small intestine mastocytosis, acute leukemias, melanomas and colon tumors. The genetics of these diseases could be better understood by knowing the functional relevance of their SNP variation. In this study, a computational analysis to detect the most deleterious nonsynonymous SNPs of KIT gene was performed and investigated its binding affinity to native and predicted mutant protein structure (D816V) with sunitinib and HDAC (Trichostatin A and Panobinostat) inhibitors was investigated. Out of 1,288 SNPs retrieved from dbSNP database against KIT gene, 11 non-synonymous SNPs were detected to be damaging and deleterious by SIFT, PolyPhen and I-Mutant2.0 servers. Further, we modeled the mutant protein based on the deleterious nsSNP (rs121913507) and showed that the mutation from Aspartic acid to Valine at 816 position exhibit greatest impact on stability. The RMSD values of mutant and native structures are found to be 0.40 and 1.9 A, respectively. Furthermore, the binding affinity of sunitinib and HDAC inhibitors were compared with native and mutant protein. In this regard, it was found that trichostatin A has a high binding efficacy towards the mutant protein with a binding energy of -35.274 kcal mol(-1), as compared to the native structure which has a binding energy of -25.996 kcal mol(-1). Also, the FastSNP tool suggested that 3 SNPs found to affect protein splicing site and splicing regulation. From present results, it was clear that the non-synonymous SNP rs121913507 (D816V) could be the most deleterious SNP for KIT gene and HDAC inhibitors can serve as a best drug for the mutant protein. 
 The CD20 homologue MS4A4 directs trafficking of KIT toward clathrin-independent endocytosis pathways and thus regulates receptor signaling and recycling. MS4A family members differentially regulate the cell cycle, and aberrant, or loss of, expression of MS4A family proteins has been observed in colon and lung cancer. However, the precise functions of MS4A family proteins and their mechanistic interactions remain unsolved. Here we report that MS4A4 facilitates trafficking of the receptor tyrosine kinase KIT through endocytic recycling rather than degradation pathways by a mechanism that involves recruitment of KIT to caveolin-1-enriched microdomains. Silencing of MS4A4 in human mast cells altered ligand-induced KIT endocytosis pathways and reduced receptor recycling to the cell surface, thus promoting KIT signaling in the endosomes while reducing that in the plasma membrane, as exemplified by Akt and PLCγ1 phosphorylation, respectively. The altered endocytic trafficking of KIT also resulted in an increase in SCF-induced mast cell proliferation and   migratio  n, which may reflect altered signaling in these cells. Our data reveal a novel function for MS4A family proteins in regulating trafficking and signaling, which could have implications in both proliferative and immunological diseases. 
 Increased Kit/SCF receptor induced mitogenicity but abolished cell motility after inhibition of protein kinase C. The product of the c-kit proto-oncogene, denoted Kit/SCF-R, encodes a tyrosine kinase receptor for stem cell factor (SCF). Kit/SCF-R induces proliferation, differentiation or   migration   of cells within the hematopoietic, gametogenic and melanogenic lineages at different developmental stages. We report here that protein kinase C (PKC) mediates phosphorylation of Kit/SCF-R on serine residues in response to SCF or PMA in intact cells. The phosphorylation inhibits SCF-induced tyrosine autophosphorylation of Kit/SCF-R. In vitro studies showed that PKC phosphorylated the Kit/SCF-R directly on serine residues and inhibited autophosphorylation of Kit/SCF-R, as well as its kinase activity towards an exogenous substrate. The PKC-induced phosphorylation did not affect Kit/SCF-R ligand binding affinity. Inhibition of PKC led to increased SCF-induced tyrosine autophosphorylation, as well as increased SCF-induced mitogenicity. In contrast, PKC was necessary for SCF-induced motility responses, including actin reorganization and chemotaxis. Our data suggest that PKC is involved in a negative feedback loop which regulates the Kit/SCF-R and that the activity of PKC determines whether the effect of SCF will be preferentially mitogenic or motogenic. 
 Masitinib (AB1010), a potent and selective tyrosine kinase inhibitor targeting KIT. The stem cell factor receptor, KIT, is a target for the treatment of cancer, mastocytosis, and inflammatory diseases. Here, we characterise the in vitro and in vivo profiles of masitinib (AB1010), a novel phenylaminothiazole-type tyrosine kinase inhibitor that targets KIT.In vitro, masitinib had greater activity and selectivity against KIT than imatinib, inhibiting recombinant human wild-type KIT with an half inhibitory concentration (IC(50)) of 200+/-40 nM and blocking stem cell factor-induced proliferation and KIT tyrosine phosphorylation with an IC(50) of 150+/-80 nM in Ba/F3 cells expressing human or mouse wild-type KIT. Masitinib also potently inhibited recombinant PDGFR and the intracellular kinase Lyn, and to a lesser extent, fibroblast growth factor receptor 3. In contrast, masitinib demonstrated weak inhibition of ABL and c-Fms and was inactive against a variety of other tyrosine and serine/threonine kinases. This highly selective nature of masitinib suggests that it will exhibit a better safety profile than other tyrosine kinase inhibitors; indeed, masitinib-induced cardiotoxicity or genotoxicity has not been observed in animal studies. Molecular modelling and kinetic analysis suggest a different mode of binding than imatinib, and masitinib more strongly inhibited degranulation, cytokine production, and bone marrow mast cell   migration   than imatinib. Furthermore, masitinib potently inhibited human and murine KIT with activating mutations in the juxtamembrane domain. In vivo, masitinib blocked tumour growth in mice with subcutaneous grafts of Ba/F3 cells expressing a juxtamembrane KIT mutant.Masitinib is a potent and selective tyrosine kinase inhibitor targeting KIT that is active, orally bioavailable in vivo, and has low toxicity. 
 The phosphatidyl inositol 3-kinase pathway is central to the pathogenesis of Kit-activated melanoma. Mouse Kit L575P, the ortholog of human KIT L576P, a common KIT mutation found in human melanoma was expressed in an immortalized but non-transformed mouse Ink4a-Arf-deficient melanocyte cell line. The resultant Ink4a-Arf-deficient Kit L575P-expressing melanocytes exhibited increased proliferation, the ability to grow in soft agar, and increased   migration  . When these cells were injected subcutaneously into NOD/SCID/gamma(c) mice, melanomas arose in 5 of 7 (71%) mice. One of seven mice (14%) injected with these cells developed metastatic disease. Evaluation of signal transduction pathways downstream of constitutively activated Kit L575P revealed striking activation of the phosphatidyl inositol 3-kinase (PI3K) pathway. Inhibition of the PI3K pathway pharmacologically or genetically abolished the transformation phenotypes gained by the L575P single mutant. These studies validate this Kit L575P-activated model of melanoma and establish the PI3K pathway as a dominant signaling pathway downstream of Kit in melanoma. 
 Novel mutations of the KIT (mast/stem cell growth factor receptor) proto-oncogene in human piebaldism. Piebaldism is an autosomal dominant genetic disorder of pigmentation characterized by congenital patches of white skin and hair that lack melanocytes. Piebaldism results from mutations of the KIT proto-oncogene, which encodes the cellular receptor transmembrane tyrosine kinase for mast/stem cell growth factor. Here we describe two novel KIT mutations associated with human piebaldism. These amino acid substitutions, located in the most highly conserved sections of the KIT kinase domain, would be expected to dominant-negatively inhibit KIT-dependent signal transduction, resulting in aberrant melanocyte proliferation or   migration   during embryologic development. 
 Analysis of p53, K-ras, c-kit, and beta-catenin gene mutations in sinonasal NK/T cell lymphoma in northeast district of China. Recently we reported the different frequencies of p53 and c-kit gene mutations among sinonasal NK/T cell lymphoma (NKTCL) in Korea, north China (Beijing), and Japan, suggesting some racial, environmental, or life-style differences as a possible cause of nasal tumorigenesis. In this study, gene mutations in p53, c-kit, K-ras, and beta-catenin gene were analyzed by polymerase chain reaction (PCR)-single strand conformation polymorphism (SSCP) followed by direct sequencing in 20 cases of sinonasal NKTCL from northeast China (Shen Yang). Age of patients ranged from 5 to 63 (median, 40.0) years. p53 gene mutations were found in eight of 20 cases (40%), with exon 4 involvement in 10% of cases. The majority was missense mutations and G:C to A:T transition was predominant. The frequency of the c-kit and K-ras gene mutations was low (5%), while that of the beta-catenin gene was six of 20 cases (30%). From these findings, it is concluded that nasal NKTCL in northeast China shared common features with that in Korea in the younger onset of disease compared to that in Japan and lower frequency of p53 gene mutations with infrequent exon 4 involvement compared to that in Japan and north China. These differences might be caused by   migration   of susceptible populations or some environmental confounding factors. 
 Soluble c-kit proteins and antireceptor monoclonal antibodies confine the binding site of the stem cell factor. The binding of the stem cell factor (SCF) to the c-kit-encoded receptor tyrosine kinase stimulates a variety of biochemical responses that culminate in cellular proliferation,   migration  , or survival. The extracellular domain of p145kit consists of five immunoglobulin-like domains. To confine the ligand binding site to this portion of the receptor we generated a panel of murine monoclonal antibodies (mAbs) to the Kit protein and identified two mAbs that efficiently displaced receptor-bound SCF and also inhibited proliferation of SCF-dependent human megakaryocytes. To map the epitopes of these mAbs we constructed and expressed soluble portions of the extracellular domain of Kit, which included either the two amino-terminal Ig-like domains (denoted Kit 1-2), three Ig-like domains (Kit 1-2-3), or the entire extracellular portion (Kit-X). All three recombinant proteins were recognized by the ligand inhibitory mAbs, suggesting that the SCF binding site resides in the amino-terminal half of the ecto-domain. Consistent with this conclusion, all of the soluble proteins inhibited SCF binding to Kit-expressing cells, and they also underwent specific covalent cross-linking to the radiolabeled ligand. However, whereas Kit 1-2-3 and Kit-X displayed comparable ligand affinities, deletion of the third Ig-like domain, in Kit 1-2, involved significant reduction in SCF binding. Hence, the binding site of SCF probably includes Ig-like domains 1 and 2, but structural determinants distal to this portion may also participate in ligand recognition. 
  ECM  PubMed  
 Adult human retinal Müller glia display distinct peripheral and macular expression of CD117 and CD44 stem cell-associated proteins. Experimental evidence suggests human Müller glia exhibit neural progenitor properties in vitro. CD117 and CD44 are known to be expressed by stem cells, the survival of which appears to depend critically on interactions with hyaluronan-rich extracellular matrix   (E  CM). Here, we characterise Müller glia expression of CD117 and CD44 in normal adult human retina and describe how it correlates with hyaluronan distribution in ocul  ar   ECM. By using chromogen-based immunohistochemistry, CD117 expression was found in entire Müller glia cytoplasm spanning from inner to outer limiting membrane in both peripheral retina (PR) and macular retina (MR), mirroring expression of the established Müller glia marker vimentin. Unlike vimentin, CD117 was also strongly expressed by Müller glia nuclei. Relative to total inner nuclear layer (INL) nuclei, more CD117+ Müller glia nuclei were seen in PR than MR. By contrast, CD44 expression was found predominantly in Müller glia apical processes of PR; no expression was found in MR. Astral blue staining demonstrated the presence of hyaluronan in cortical vitreous and the interphotoreceptor matrix (IPM) in both MR and PR. Our findings demonstrate that: (i) both CD117 and CD44 are expressed by human adult Müller glia; (ii) CD117 is a robust nuclear and cytoplasmic immunohistochemical marker of Müller glia; and (iii) that while CD117 is expressed by the entire Müller glia in both PR and MR, CD44 is only expressed by Müller glia apices in PR. Since the apices of Müller glia are in direct contact with the hyaluronan-rich IPM, the Müller glia-IPM interface in PR is likely a favourable region for supporting progenitor or stem cell-like signalling. These observations provide novel insights into potential stem-cell favouring microenvironments in mature human retina. 
  [Ee]xtracellular [Mm]atrix  PubMed  
 Adult human retinal Müller glia display distinct peripheral and macular expression of CD117 and CD44 stem cell-associated proteins. Experimental evidence suggests human Müller glia exhibit neural progenitor properties in vitro. CD117 and CD44 are known to be expressed by stem cells, the survival of which appears to depend critically on interactions with hyaluronan-ric  h extracellular matr  ix (ECM). Here, we characterise Müller glia expression of CD117 and CD44 in normal adult human retina and describe how it correlates with hyaluronan distribution in ocular ECM. By using chromogen-based immunohistochemistry, CD117 expression was found in entire Müller glia cytoplasm spanning from inner to outer limiting membrane in both peripheral retina (PR) and macular retina (MR), mirroring expression of the established Müller glia marker vimentin. Unlike vimentin, CD117 was also strongly expressed by Müller glia nuclei. Relative to total inner nuclear layer (INL) nuclei, more CD117+ Müller glia nuclei were seen in PR than MR. By contrast, CD44 expression was found predominantly in Müller glia apical processes of PR; no expression was found in MR. Astral blue staining demonstrated the presence of hyaluronan in cortical vitreous and the interphotoreceptor matrix (IPM) in both MR and PR. Our findings demonstrate that: (i) both CD117 and CD44 are expressed by human adult Müller glia; (ii) CD117 is a robust nuclear and cytoplasmic immunohistochemical marker of Müller glia; and (iii) that while CD117 is expressed by the entire Müller glia in both PR and MR, CD44 is only expressed by Müller glia apices in PR. Since the apices of Müller glia are in direct contact with the hyaluronan-rich IPM, the Müller glia-IPM interface in PR is likely a favourable region for supporting progenitor or stem cell-like signalling. These observations provide novel insights into potential stem-cell favouring microenvironments in mature human retina. 
 CD117-positive cells in adult human heart are localized in the subepicardium, and their activation is associated with laminin-1 and alpha6 integrin expression. CD117-positive cells contributing to cardiac cell turnover in normal and pathological conditions have recently been described in adult human heart. Since the precise spatial and temporal expression of   extracellular matrix   proteins and their receptors is critical for organ formation, we compared the distribution of cardiac primitive CD117-positive cells in the human adult normal and pathological hearts with ischemic cardiomyopathy, with respect to localization and expression of laminin and integrin isoforms. In the pathological hearts, CD117-positive cells were significantly more numerous than in the normal hearts. They were localized mainly in the atria and were up to 38-fold more numerous in the subepicardium than in the myocardium. Compared with normal hearts, most CD117-positive cells in the subepicardium of pathological hearts were alpha(6) integrin-positive. Laminin-1, typical of developing heart, was found predominantly in the subepicardium of adult heart. Immunoblotting revealed its highest expression in the normal atrium and pathological left ventricle. Both laminin isoforms reduced apoptosis and increased proliferation and migration of CD117-positive cells in vitro with respect to control, but the effects of laminin-1 significantly outweighed those of laminin-2. Signaling mediated by alpha(6) integrin was implicated in the migration and protection from apoptosis, as documented by transfection with specific small interfering RNA. These data reveal that the increase in the number of cardiac CD117-positive cells and the expression of laminin-1 are observed in ischemic cardiomyopathy. Subepicardial localization of CD117-positive cells and expression of laminin-1 and alpha(6) integrin subunits may all correspond to the activation of regeneration involving an epithelial-mesenchymal transition recently described in adult heart. 
 Oncogenic KIT-containing exosomes increase gastrointestinal stromal tumor cell invasion. During tumor development, constant interplay occurs between tumor cells and surrounding stromal cells. We report evidence that gastrointestinal stromal tumor (GIST) cells invade the interstitial stroma through the release of the oncogenic protein tyrosine kinase (KIT)-containing exosomes, which triggers the phenotypic conversion of progenitor smooth muscle cells to tumor-promoting cells. These recipient cells display morphologic changes and acquire tumor-associated phenotypes, including enhanced adhesion to   extracellular matrix   proteins, activation of intracellular pathways downstream of KIT, expression of Interstitial Cell of Cajal-like markers, and release of various matrix metalloproteinases (MMPs), particularly MMP1. This report shows stimulation of MMP1 production by stromal cells via uptake of tumor-derived exosomes, which leads to tumor cell invasion. Exosomes derived from GIST patients but not healthy donors show enhanced MMP1 secretion by smooth muscle cells and tumor cell invasion, whereas selective blocking of exosome-mediated MMP1 secretion decreases tumor invasiveness. Our study indicates that exosome release and subsequent MMP1 induction creates a positive feedback mechanism established between tumor and stromal cells that drives GIST development and offers unique insights for potential therapeutic strategies to block GIST progression and metastatic spread. 
 Epithelial-mesenchymal transition of epicardial mesothelium is a source of cardiac CD117-positive stem cells in adult human heart. Epithelial-mesenchymal transition is implicated in the remodelling of tissues during development and in the adult life. In the heart, it gives origin to progenitors of fibroblasts, coronary endothelium, smooth muscle cells, and cardiomyocytes. Moreover, epicardially-derived cells determine myocardial wall thickness and Purkinje fibre network. Recently, the presence of numerous cardiac stem cells in the subepicardium of the adult human heart has been described and the hypothesis that epicardially-derived cells can contribute to the population of cardiac stem cells in the adult heart has been advanced. In an effort to test this hypothesis and establish a possible link between epicardium, epicardially-derived cells and cardiac stem cells in the adult human heart we have examined epicardial mesothelial cells in the normal and pathological adult human heart with ischemic cardiomyopathy in vivo and we have induced and documented their epithelial-mesenchymal transition in vitro. Noticeably, epicardial cells were missing from the surface of pathological hearts and the cells with the expression of epithelial and mesenchymal markers populated thick subepicardial space. When the fragments of epicardium from the normal hearts were cultured on the specific substrate formed by   extracellular matrix   derived from cardiac fibroblasts, we obtained the outgrowth of the epithelial sheet with the mRNA and protein expression characteristic of epicardium. TGFβ induced cellular and molecular changes typical of epithelial-mesenchymal transition. Moreover, the epicardially-derived cells expressed CD117 antigen. Thus, this study provides evidence that cardiac stem cells can originate from epithelial-mesenchymal transition of the epicardial cells in the adult human heart. 
  [Ii]nvasive  PubMed  
 Urinary bladder urothelial carcinoma with expression of KIT and PDGFRA and showing diverse differentiations into plasmacytoid, clear cell, acantholytic, nested, and spindle variants, and into adenocarcinoma, signet-ring cell carcinoma, small cell carcinoma, large cell carcinoma, and pleomorphic carcinoma. Various tumors can arise in the urinary bladder (UB); most common is urothelial carcinoma (UC). UC of the UB have many variants. Other types of carcinomas such as adenocarcinoma (AC) and small cell carcinoma (SmCC) can occur in UB carcinomas. Expression of KIT and PDGFRA has not been reported. A 66-year-old man admitted to our hospital because of hematuria. Cystoscopy revealed papillary   invasive   tumor and a transurethral bladder tumorectomy (TUR-BT) was performed. The TUR-BT showed UC, AC, SmCC, large cell carcinoma (LCC), and pleomorphic carcinoma (PC). The UC component showed plasmacytoid, spindle, nested, clear cell, acantholytic variants. The AC element showed tubular adenocarcinoma and signet-ring cell carcinoma (Sig). Immunohistochemically, all of these subtypes were positive for cytokeratin (CK) AE1/3, CK CAM5.2, CK34BE12, CK5, CK6, CK7, CK8, CK18, CK19, CK20, EMA, CEA, p63, CA19-9, p53 (positive 45%), MUC1, NSE, NCAM, KIT, PDGFRA, and Ki-67 (87%). They were negative for vimentin, chromogranin, synaptophysin, S100 protein, CD34, CD14, α-smooth muscle actin, CD31, caldesmon, CD138, CD45, κ-chain, λ-chain, MUC2, MUC5AC and MUC6. Mucin histochemistry revealed mucins in AC element including Sig. A molecular genetic analysis using PCR-direct sequencing method identified no mutations of KIT (exons 9, 11, 13, and 17) and PDGFRA (exons 12 and 18) genes. The carcinoma was highly aggressive and invaded into muscular layer. The nuclear grade was very high, and there were numerous lymphovascular permeations were seen. The surface showed carcinoma in situ involving von-Brunn's nests. This case shows that carcinoma of UB can show diverse differentiations into numerous histological types and variants, and can express KIT and PDGFRA. The both genes showed no mutations in the present case. 
 Expression of C-KIT, CD24, CD44s, and COX2 in benign and non-  invasive   apocrine lesions of the breast. Benign apocrine metaplasia (AM) of the adult breast is a very common, but enigmatic lesion. It has been speculated that AM might be a precursor of malignancy or an indicator of a susceptibility of the breast tissue to develop neoplasia, mainly based on comparing the frequency of AM in breast cancer and non-breast cancer patients [1]. Studies using comparative genomic hybridization have supported this by showing similar molecular alterations in benign and malignant apocrine lesions [2]. Few studies, however, have compared expression of biomarkers involved in tumor progression in AM and progressively more advanced atypical apocrine lesions. The expression of C-KIT, COX2, CD24, and CD44s was evaluated by immunohistochemistry in formalin-fixed, paraffin-embedded material of 9 AM, 20 apocrine ductal intraepithelial neoplasia (DIN1c-3) and 40 atypical apocrine lesions (not qualifying for DIN1c-3) and compared to expression of the same biomarkers in adjacent normal ductal epithelium. Of the 66 apocrine lesions, 62 (94 %) did not express C-KIT compared to 4/63 (6 %) of the normal glands (Fisher's exact, p < 0.001). COX2 was expressed in a significantly higher proportion of apocrine lesions than of normal glands (49 vs. 14 %, p < 0.001), and the number of apocrine lesions positive for CD24 was found to be higher with increasing aggressiveness of the lesions (Spearman, p < 0.001). In conclusion,   benign a  nd non-invasive proliferative apocrine lesions of the breast display immuno-phenotypical characteristics previously ascribed mainly to malignant transformation. This could lend support to the theory that AM is an early step towards malignant transformation, albeit associated with slow progression to carcinoma. 
 Low incidence of KIT gene mutations and no PDGFRA gene mutations in primary cutaneous melanoma: an immunohistochemical and molecular genetic study of Japanese cases. Although several studies have reported the incidence of KIT and PDGFRA gene mutations in cutaneous melanomas of Caucasians, only one report is available for Mongoloids, including Japanese. In Japan, melanoma is an uncommon neoplasm compared with countries with Caucasian populations.In this study, protein expression and gene mutations of these two genes were investigated by immunohistochemistry and PCR-direct sequencing in 12 primary cutaneous   invasive   melanomas of Japanese patients. Acral melanoma was detected in 2 cases, chronically sun-damaged skin melanoma in 3 cases, and non-chronically sun-damaged skin melanoma in 7 cases. Genetic analysis was performed in exons 9, 11, 13, and 17 of the KIT gene and in exons 12 and 18 of the PDGFRA gene.Point mutations of the KIT gene were recognized in only one (8%) case, codon 559 of exon 11 (GTT → GCT). This is a melanoma of the sole (acral melanoma). No mutations of the PDGFRA gene were recognized. At the protein level, 11 case (92%) showed membranous expression of KIT; the expression did not correlate with melanoma depth. KIT expression was stronger in the peripheral in situ or mini  mal inva  sive areas than the cent  ral inva  sive areas. Membranous PDGFRA expression was found in all cases (100%).In this study, only 8% of melanomas had the KIT mutation in this Japanese population. The finding of a mutation in one of two acral melanomas suggests that KIT inhibitors, for example imatinib, which are already used in clinics for treatment of GIST, can also be used for melanoma. 
 Expression of C-KIT, CD24, CD44s, and COX2 in benign and non-  invasive   apocrine lesions of the breast. Benign apocrine metaplasia (AM) of the adult breast is a very common, but enigmatic lesion. It has been speculated that AM might be a precursor of malignancy or an indicator of a susceptibility of the breast tissue to develop neoplasia, mainly based on comparing the frequency of AM in breast cancer and non-breast cancer patients [1]. Studies using comparative genomic hybridization have supported this by showing similar molecular alterations in benign and malignant apocrine lesions [2]. Few studies, however, have compared expression of biomarkers involved in tumor progression in AM and progressively more advanced atypical apocrine lesions. The expression of C-KIT, COX2, CD24, and CD44s was evaluated by immunohistochemistry in formalin-fixed, paraffin-embedded material of 9 AM, 20 apocrine ductal intraepithelial neoplasia (DIN1c-3) and 40 atypical apocrine lesions (not qualifying for DIN1c-3) and compared to expression of the same biomarkers in adjacent normal ductal epithelium. Of the 66 apocrine lesions, 62 (94 %) did not express C-KIT compared to 4/63 (6 %) of the normal glands (Fisher's exact, p < 0.001). COX2 was expressed in a significantly higher proportion of apocrine lesions than of normal glands (49 vs. 14 %, p < 0.001), and the number of apocrine lesions positive for CD24 was found to be higher with increasing aggressiveness of the lesions (Spearman, p < 0.001). In conclusion,   benign a  nd non-invasive proliferative apocrine lesions of the breast display immuno-phenotypical characteristics previously ascribed mainly to malignant transformation. This could lend support to the theory that AM is an early step towards malignant transformation, albeit associated with slow progression to carcinoma. 
 Urinary bladder urothelial carcinoma with expression of KIT and PDGFRA and showing diverse differentiations into plasmacytoid, clear cell, acantholytic, nested, and spindle variants, and into adenocarcinoma, signet-ring cell carcinoma, small cell carcinoma, large cell carcinoma, and pleomorphic carcinoma. Various tumors can arise in the urinary bladder (UB); most common is urothelial carcinoma (UC). UC of the UB have many variants. Other types of carcinomas such as adenocarcinoma (AC) and small cell carcinoma (SmCC) can occur in UB carcinomas. Expression of KIT and PDGFRA has not been reported. A 66-year-old man admitted to our hospital because of hematuria. Cystoscopy revealed papillary   invasive   tumor and a transurethral bladder tumorectomy (TUR-BT) was performed. The TUR-BT showed UC, AC, SmCC, large cell carcinoma (LCC), and pleomorphic carcinoma (PC). The UC component showed plasmacytoid, spindle, nested, clear cell, acantholytic variants. The AC element showed tubular adenocarcinoma and signet-ring cell carcinoma (Sig). Immunohistochemically, all of these subtypes were positive for cytokeratin (CK) AE1/3, CK CAM5.2, CK34BE12, CK5, CK6, CK7, CK8, CK18, CK19, CK20, EMA, CEA, p63, CA19-9, p53 (positive 45%), MUC1, NSE, NCAM, KIT, PDGFRA, and Ki-67 (87%). They were negative for vimentin, chromogranin, synaptophysin, S100 protein, CD34, CD14, α-smooth muscle actin, CD31, caldesmon, CD138, CD45, κ-chain, λ-chain, MUC2, MUC5AC and MUC6. Mucin histochemistry revealed mucins in AC element including Sig. A molecular genetic analysis using PCR-direct sequencing method identified no mutations of KIT (exons 9, 11, 13, and 17) and PDGFRA (exons 12 and 18) genes. The carcinoma was highly aggressive and invaded into muscular layer. The nuclear grade was very high, and there were numerous lymphovascular permeations were seen. The surface showed carcinoma in situ involving von-Brunn's nests. This case shows that carcinoma of UB can show diverse differentiations into numerous histological types and variants, and can express KIT and PDGFRA. The both genes showed no mutations in the present case. 
 Low incidence of KIT gene mutations and no PDGFRA gene mutations in primary cutaneous melanoma: an immunohistochemical and molecular genetic study of Japanese cases. Although several studies have reported the incidence of KIT and PDGFRA gene mutations in cutaneous melanomas of Caucasians, only one report is available for Mongoloids, including Japanese. In Japan, melanoma is an uncommon neoplasm compared with countries with Caucasian populations.In this study, protein expression and gene mutations of these two genes were investigated by immunohistochemistry and PCR-direct sequencing in 12 primary cutaneous   invasive   melanomas of Japanese patients. Acral melanoma was detected in 2 cases, chronically sun-damaged skin melanoma in 3 cases, and non-chronically sun-damaged skin melanoma in 7 cases. Genetic analysis was performed in exons 9, 11, 13, and 17 of the KIT gene and in exons 12 and 18 of the PDGFRA gene.Point mutations of the KIT gene were recognized in only one (8%) case, codon 559 of exon 11 (GTT → GCT). This is a melanoma of the sole (acral melanoma). No mutations of the PDGFRA gene were recognized. At the protein level, 11 case (92%) showed membranous expression of KIT; the expression did not correlate with melanoma depth. KIT expression was stronger in the peripheral in situ or mini  mal inva  sive areas than the cent  ral inva  sive areas. Membranous PDGFRA expression was found in all cases (100%).In this study, only 8% of melanomas had the KIT mutation in this Japanese population. The finding of a mutation in one of two acral melanomas suggests that KIT inhibitors, for example imatinib, which are already used in clinics for treatment of GIST, can also be used for melanoma. 
 Biofunctional characteristics of in situ and   invasive   breast carcinoma. The increasing use of breast-conserving surgery makes it essential to identify biofunctional profiles responsible for the progression of in situ to   invasive   carcinomas to facilitate the detection of lesions that are most likely to relapse or progress and, thus, to be able to offer patients tailored treatment options. Our objective was to analyse and compare biofunctional profiles in ductal carcinomas in situ (DCIS) and   invasive   ductal carcinomas (IDC). We also aimed to identify markers in tumor and normal surrounding tissues that may be predictive of locoregional recurrence in patients with DCIS.Biofunctional parameters including mitotic activity, estrogen receptor, progesterone receptor, microvessel density (MVD), c-kit and p27 expression were evaluated in 829 in situ and   invasive   carcinomas. The impact of the biomarker profiles of DCIS, IDC and normal surrounding tissues on loco-regional recurrence was analyzed.A progressive increase in cell proliferation and a concomitant decrease in steroid hormone receptor-positive lesions was observed during the transition from in situ to   invasive   carcinomas, as also within each subgroup as grade increased. Conversely, p27 expression and MVD dramatically decreased during the transition from in situ to   invasive   carcinomas. Finally, we found that a low c-kit expression was indicative of IDC relapse.Cell proliferation, hormonal and differentiation characteristics differed in DCIS with respect to IDC, and the main variation in the transition between the two histologic lesions was the decrease in p27 expression and MVD. 
 C-kit expression in human osteosarcoma and in vitro assays. Biologic agents targeting oncogenes have encourage researchs trying to correlate the role of tyrosine kinase in the pathogenesis of tumours. Osteosarcoma is a high grade aggressive neoplasm with poor survival. Our aim was to investigate c-kit immunoexpression, its prognostic relevance for patients with osteosarcoma, and the effect of imatinib mesylate (STI571) on proliferation and invasion of the human osteosarcoma cell line.A retrospective immunohistochemical study was performed on archival formalin-fixed paraffin-embedded specimens from 52 patients with high-grade primary osteosarcoma of extremities treated at the Pediatric Oncology Institute (IOP, GRAAC) and archived in the Department of Pathology, Federal University of São Paulo. Only pre-chemotherapy specimens were analyzed. Strongly stained cytoplasm and membrane cells were taken as positive. Human osteosarcoma cells from line MG-63 were incubated and the inhibitory effect of imatinib mesylate (STI571) on cell proliferation and invasion was studied. In 24 cases (46.15%), c-kit was expressed by the cells and c-kit-positive tumors exhibited lower necrosis post-chemotherapy. No correlation was found between c-kit expression and overall and disease-free survival. Imatinib mesylate decreased the rates of cell growth of osteosarcoma cells in low doses and invasion in high doses C-kit-positive tumors had worse response to chemotherapy and imatinib mesylate can play a role in blocking or decreasing the rate of growth of osteosarcoma cells, but not the   invasiv  e capacity of these neoplastic cells. These data suggested that imatinib mesylate could be a therapeutic target of strategies against osteosarcoma tumors. Further studies are necessary to confirm this indication. 
 C-kit protein expression correlated with activating mutations in KIT gene in oral mucosal melanoma. C-kit is a trans-membrane receptor tyrosine kinase (RTK) encoded by the proto-oncogene KIT located at 4q11-12. Gain-of-function mutations arising to c-kit activation independent of its ligand were observed in various tumors related to germ cells, mast cells, and interstitial cells of Cajal. C-kit also participates in melanocyte development; hence, its involvement in oral mucosal melanoma (OMM) tumorigenesis was investigated. Immunohistochemistry and mutation analysis were performed using 18 cases of human primary OMM. Results revealed 16 cases positive to c-kit protein. Atypical melanocytes expressed c-kit. All in situ components expressed c-kit, but only four cases exhibited intense expression in the   invasive   component. Missense mutations were observed in four cases, and two of those correlated with increased protein expression. C-kit expression in atypical melanocytes suggests the role of c-kit in the early stage of OMM tumorigenesis. C-kit protein expression correlated with activating mutations indicating the pertinent role of the proto-oncogene KIT in the tumorigenesis of OMM. 
 Screening of the c-kit gene missense mutation in   invasive   ductal carcinoma of breast among north Indian population. Breast cancer is one of the most frequently diagnosed cancers and the leading cause of cancer deaths among females across the world, accounting for 23 % (1.38 million) of total new cancer cases and 14 % (0.45 million) of the total cancer deaths in 2008. c-kit is expressed in mast cell growth factor, cellular migration, proliferation, melanoblasts, haematopoietic progenitors and germ cells. We have designed our study with aim to explore the c-kit gene mutations in   invasive   ductal carcinoma (IDC) breast. To ascertain the range of mutations in exon 11, 13 and 17 of c-kit gene in 53 cases of IDC breast, we carried out PCR-SSCP followed by DNA sequencing. The mutation frequency of c-kit gene in exon 11, 13 and 17 were 9.43 % (5/53), 1.88 % (1/53) and 3.77 % (2/53), respectively. During our mutational analysis, we have detected five missense mutations in exon 11 (Pro551Leu, Glu562Val, Leu576Phe, His580Tyr and Phe584Leu), one missense mutation in exon 13 (Ser639Pro) and two missense mutations in exon 17 (Arg796Gly and Asn822Ser). It seems that c-kit mutations might participate in breast cancer pathogenesis and may be utilized as predictive marker, since the loss of c-kit positivity is generally linked with different types of breast cancer. Further molecular studies are necessary to validate the association of c-kit gene mutation in IDC breast pathogenesis. 
 Mastocytosis in mice expressing human Kit receptor with the activating Asp816Val mutation. Mastocytosis is a rare neoplastic disease characterized by a pathologic accumulation of tissue mast cells (MCs). Mastocytosis is often associated with a somatic point mutation in the Kit protooncogene leading to an Asp/Val substitution at position 816 in the kinase domain of this receptor. The contribution of this mutation to mastocytosis development remains unclear. In addition, the clinical heterogeneity presented by mastocytosis patients carrying the same mutation is unexplained. We report that a disease with striking similarities to human mastocytosis develops spontaneously in transgenic mice expressing the human Asp816Val mutant Kit protooncogene specifically in MCs. This disease is characterized by clinical signs ranging from a localized and indolent MC hyperplasia to an   invasive   MC tumor. In addition, bone marrow-derived MCs from transgenic animals can be maintained in culture for >24 mo and acquire growth factor independency for proliferation. These results demonstrate a causal link in vivo between the Asp816Val Kit mutation and MC neoplasia and suggest a basis for the clinical heterogeneity of human mastocytosis. 
 Stem cell factor/c-kit signaling enhances invasion of pancreatic cancer cells via HIF-1α under normoxic condition. The SCF/c-kit signaling plays an important role in invasion of c-kit-expressing tumor cells, however, the molecular mechanisms have not been studied yet. Using a pancreatic cancer model, we demonstrate that SCF/c-kit binding up-regulates the expression of invasion-related genes through the accumulation of HIF-1α. Furthermore, the expression of HIF-1α induced by SCF is not dependent on the oxygen level, but rather on both the PI3K/Akt and Ras/MEK/ERK signaling pathways. In conclusion, under normoxic conditions, SCF/c-kit binding increases expression of HIF-1α through the PI3K/Akt and Ras/MEK/ERK pathways, and the accumulation of HIF-1α up-regulates expression of invasion-related genes that augment   the inv  asiveness of pancreatic cancer, a fatal cancer. Therefore, our results suggest that the inhibition of both c-kit and HIF-1α may be an effective strategy for pancreatic cancer therapy. 
 KIT and RAS signalling pathways in testicular germ cell tumours: new data and a review of the literature. Testicular germ cell tumours (TGCTs) are the leading cause of cancer deaths in young male Caucasians. Identifying changes in DNA copy number can pinpoint genes involved in tumour development. We defined the smallest overlapping regions of imbalance in TGCTs using array comparative genomic hybridization analysis. Novel regions, or regions which refined those previously reported, were identified. The expression profile of genes from 12p, which is invariably gained in TGCTs, and amplicons defined at 12p11.2-12.1 and 4q12, suggest KRAS and KIT involvement in TGCT and seminoma development, respectively. Amplification of these genes was not found in intratubular germ cell neoplasia adjacent to   invasive   disease showing these changes, suggesting their involvement in tumour progression. Activating mutations of RAS genes (KRAS or NRAS) and overexpression of KRAS were mutually exclusive events. These, correlations between the expression levels of KIT, KRAS and GRB7 (which encodes an adapter molecule known to interact with the KIT tyrosine kinase receptor) and other reported evidence reviewed here, are consistent with a role for activation of KIT and RAS signalling in TGCT development. In order to assess a role for KIT in seminomas, we modulated the level of KIT expression in TCam-2, a seminoma cell line. The likely seminomatous origin of this cell line was supported by demonstrating KIT and OCT3/4 overexpression and gain of 12p material. Reducing the expression of KIT in TCam-2 through RNA inhibition resulted in decreased cell viability. Further understanding of KIT and RAS signalling in TGCTs may lead to novel therapeutic approaches for these tumours. 
 A clinicopathological review of 33 patients with vulvar melanoma identifies c-KIT as a prognostic marker. Vulvar melanoma is the second most common vulvar cancer. Patients with vulvar melanoma usually present with the disease at a late stage and have a poor prognosis. The prognostic predictors reported in the literature are not unequivocal and the role of lichen sclerosus and c-KIT mutations in the aetiology of vulvar melanoma is unclear. Breslow staging currently seems to be the most adequate predictor of prognosis. We thus performed a clinicopathological and literature review to identify suitable predictors of prognosis and survival and investigated the expression of c-KIT (by immunohistochemistry) in patients with vulvar melanoma (n=33) from the Gynaecological Cancer Centres of the Royal Hospital for Women (Sydney, Australia) and John Hunter Hospital (Newcastle, Australia). Our series of 33 patients fitted the expected clinical profile of older women: delayed presentation, high stage, limited response to treatment and poor prognosis. We identified 3 patients (9.1%) with lichen sclerosus associated with melanoma in situ, although no lichen sclerosus was found in the areas of   invasive   melanoma. No patient had vulvar nevi. We identified a) Breslow's depth, b) an absence of any of the pathological risk factors, such as satellitosis, in-transit metastasis, lymphovascular space invasion (LVSI) and dermal mitosis, c) removal of inguino-femoral lymph nodes, d) lateral margin of >1 cm, and e) c-KIT expression as valuable prognostic predictors for disease-free survival. We conclude that c-KIT expression is, apart from Breslow's depth, another valuable predictor of prognosis and survival. Lichen sclerosus may be associated with vulvar melanoma. 
 C-KIT expression in ductal carcinoma in situ of the breast: co-expression with HER-2/neu. The proto-oncogene c-KIT (CD117) is highly expressed in normal breast epithelium and is decreased in   invasive   breast cancer. In this study, we analyzed the protein expression and the mutational status of c-KIT in ductal carcinoma in situ (DCIS) of the breast and correlated these findings with nuclear grade, architectural pattern, and expression of HER-2, estrogen receptor (ER)-alpha, and progesterone receptor (PR). C-KIT, HER-2, ER, and PR expression were analyzed immunohistochemically in 106 cases of paraffin-embedded DCIS (85 pure DCIS and 21 DCIS with concurrent carcinoma). Direct sequencing of exons 9 and 11 of the c-KIT gene was performed to analyze the hot spot mutational regions in representative cases. C-KIT expression was found in 55 (52.8%) of all DCIS, correlating with high nuclear grade (P < .0001), comedonecrosis (P < .0001), and solid growth pattern (P = .001). Furthermore, c-KIT expression was strongly associated with HER-2 positivity (P < .0001) and was significantly lower in ER- or PR-positive cases (P = .001 and P = .006, respectively). C-KIT expression alone or co-expression with HER-2 in pure DCIS did not differ significantly from DCIS with   invasive   component (P = .09). Mutational analysis in 6 c-KIT-positive DCIS revealed no activating mutations in exons 9 or 11. Our findings suggest that the expression of c-KIT protein might define a subset of poorly differentiated, HER-2-positive DCIS with decreased expression of steroid hormone receptors, comedonecrosis, and a solid growth pattern. The implications of c-KIT and HER-2 co-expression for breast carcinogenesis should be further evaluated. 
 Oncogenic KIT-containing exosomes increase gastrointestinal stromal tumor cell invasion. During tumor development, constant interplay occurs between tumor cells and surrounding stromal cells. We report evidence that gastrointestinal stromal tumor (GIST) cells invade the interstitial stroma through the release of the oncogenic protein tyrosine kinase (KIT)-containing exosomes, which triggers the phenotypic conversion of progenitor smooth muscle cells to tumor-promoting cells. These recipient cells display morphologic changes and acquire tumor-associated phenotypes, including enhanced adhesion to extracellular matrix proteins, activation of intracellular pathways downstream of KIT, expression of Interstitial Cell of Cajal-like markers, and release of various matrix metalloproteinases (MMPs), particularly MMP1. This report shows stimulation of MMP1 production by stromal cells via uptake of tumor-derived exosomes, which leads to tumor cell invasion. Exosomes derived from GIST patients but not healthy donors show enhanced MMP1 secretion by smooth muscle cells and tumor cell invasion, whereas selective blocking of exosome-mediated MMP1 secretion decreases tumor   invasive  ness. Our study indicates that exosome release and subsequent MMP1 induction creates a positive feedback mechanism established between tumor and stromal cells that drives GIST development and offers unique insights for potential therapeutic strategies to block GIST progression and metastatic spread. 
 Acquired resistance to imatinib and secondary KIT exon 13 mutation in gastrointestinal stromal tumour. Gastrointestinal stromal tumours (GISTs) are the most common mesenchymal tumours of the gastrointestinal tract. Most of them have an activating mutation of KIT or PDGFRalpha tyrosine-kinase receptors. Imatinib is a selective tyrosine-kinase inhibitor of ABL, KIT and PDGFR, and provides a clinical benefit in about 85% of patients with advanced GIST. Unfortunately, secondary resistance following initial responses occurs in most of the cases, and molecular mechanisms are poorly understood. We sequenced KIT and PGDFRalpha exons from one patient with GIST before and after the development of imatinib resistance. We identified, in addition to a primary mutation in exon 9, a secondary mutation in KIT exon 13 (first kinase domain) in the resistant sample. We demonstrate for the first time the feasibility of sequencing such samples removed by non-surgical biopsies during imatinib therapy. Such a approach, far less   invasive   than surgery and combined with sequencing, will likely help in better tailoring the treatment of advanced GISTs and understanding the mechanisms of resistance and response to imatinib. 
 C-kit signaling promotes proliferation and invasion of colorectal mucinous adenocarcinoma in a murine model. It was reported that the receptor tyrosine kinase (RTK) family often highly expressed in several mucinous carcinomas. In the present study, we established a murine model of colorectal mucinous adenocardinoma (CRMAC) by treating C57 mice [both wild type (WT) and loss-of-function c-kit mutant type (Wads-/-)] with AOM+DSS for 37 weeks and found that c-kit, a member of RTK family, clearly enhanced the tumor cell proliferation by decreasing p53 and increasing cyclin D1 through AKT pathway. Significantly, c-kit strongly promoted tumor cell   invasive  ness by increasing ETV4, which induced MMP7 expression and epithelial-mesenchymal transition (EMT) via ERK pathway. In vitro up- or down-regulating c-kit activation in human colorectal cancer HCT-116 cells further consolidated these results. In conclusion, our data suggested that the c-kit signaling obviously promoted proliferation and invasion of CRMAC. Therefore, targeting the c-kit signaling and its downstream molecules might provide the potential strategies for treatment of patients suffering from CRMAC in the future. 
 Oncogene abnormalities in a series of primary melanomas of the sinonasal tract: NRAS mutations and cyclin D1 amplification are more frequent than KIT or BRAF mutations. Primary malignant melanoma of sinonasal tract is a rare but severe form of melanoma. We retrospectively analyzed 17 cases and focused on the histologic presentation and the expression of c-Kit, epidermal growth factor receptor (EGFR), cyclin D1/Bcl-1, PS100, and HMB45 and searched for BRAF, NRAS, and KIT mutations that are known to be associated with melanoma subtypes, together with amplifications of KIT, cyclin D1, cyclin-dependent kinase 4, MDM2, and microphthalmia-associated transcription factor using quantitative polymerase chain reaction. In most cases (78%), an in situ component was evidenced.   Invasive   components were composed of diffuse areas of rhabdoid, epithelioid, or spindle cells and, in most cases, lacked inflammatory reaction, suggesting that an immune escape phenomenon probably develops when the disease progresses. EGFR was rarely and weakly expressed in the in situ component of 2 cases. None of the investigated case showed BRAF V600E, but 1 had a D594G mutation. NRAS mutations in exon 2 (G12D or G12A) were found in 3 cases (18%), and a KIT mutation in exon 11 (L576P), in 1, whereas c-Kit was expressed at the protein level in half of the cases. Amplifications of cyclin D1 were evidenced in 5 cases, confirmed in 3 by fluorescence in situ hybridization, but this was not always correlated with protein expression, found in 8 patients (62.5%), 3 having no significant amplification. In conclusion, primary malignant melanoma of sinonasal tract is not associated with BRAF V600E mutations. Instead, NRAS or KIT mutations and cyclin D1 amplification can be found in a proportion of cases, suggesting that primary malignant melanoma of sinonasal tract is heterogeneous at the molecular level and should not be sensitive to therapeutic approaches aiming at BRAF. 
 Analysis of c-kit protein expression in pancreatic neoplasms and its implication for prognosis. To evaluate the expression and role of c-kit protein in the neoplasia of pancreatic neoplasms and its relationship to prognosis in   invasive   ductal carcinoma (IDC) of the pancreas.The immunohistochemical expression of c-kit protein was studied in normal pancreatic tissues, intraductal papillary-mucinous neoplasms of the pancreas (IPMN), mucinous cystic neoplasm of the pancreas (MCN), and IDC of the pancreas. The immunoreactive score (IRS) of c-kit protein expression was examined in normal pancreatic ductal cells, neoplastic cells, and pancreatic endocrine cells.The IRS values of c-kit protein expression in various neoplastic cells were significantly higher than those in normal ductal cells. No significant difference was seen between IRS values of c-kit protein expression in various pancreatic neoplastic cells of IDC of the pancreas, IPMN and MCN. No significant difference was seen between IRS values of c-kit expression in endocrine cells among various pancreatic tissues. Furthermore, a survival analysis in patients with IDC of the pancreas showed an obvious trend toward decreased survival in patients with c-kit-positive cancer, and c-kit protein expression did not correlate with any clinicopathological factors in IDC of the pancreas.c-kit protein expression may play an important role in neoplasia of pancreatic neoplasms. In view of the result that c-kit protein expression was found in 8% of IDC of the pancreas, a clinical trial for STI-571 (Glivec) against pancreatic cancer may be warranted for selected pancreatic cancer patients with c-kit protein expression. 
 c-Kit is suppressed in human colon cancer tissue and contributes to L1-mediated metastasis. The transmembrane neural cell adhesion receptor L1 is a Wnt/β-catenin target gene expressed in many tumor types. In human colorectal cancer, L1 localizes preferentially to the   invasiv  e front of tumors and when overexpressed in colorectal cancer cells, it facilitates their metastasis to the liver. In this study, we investigated genes that are regulated in human colorectal cancer and by the L1-NF-κB pathway that has been implicated in liver metastasis. c-Kit was the most highly suppressed gene in both colorectal cancer tissue and the L1-NF-κB pathway. c-Kit suppression that resulted from L1-mediated signaling relied upon NF-κB, which directly inhibited the transcription of SP1, a major activator of the c-Kit gene promoter. Reconstituting c-Kit expression in L1-transfected cells blocked the biological effects conferred by L1 overexpression in driving motility and liver metastasis. We found that c-Kit expression in colorectal cancer cells is associated with a more pronounced epithelial morphology, along with increased expression of E-cadherin and decreased expression of Slug. Although c-Kit overexpression inhibited the motility and metastasis of L1-expressing colorectal cancer cells, it enhanced colorectal cancer cell proliferation and tumorigenesis, arguing that separate pathways mediate tumorigenicity and metastasis by c-Kit. Our findings provide insights into how colorectal cancer metastasizes to the liver, the most common site of dissemination in this cancer. 
 Correlation between KIT expression and KIT mutation in melanoma: a study of 173 cases with emphasis on the acral-lentiginous/mucosal type. The role of immunohistochemistry in the assessment of KIT status in melanomas, especially acral lentiginous/mucosal, is not well established. Although the reported prevalence of KIT mutations in acral lentiginous/mucosal melanomas is relatively low, detection of mutations in KIT can have profound therapeutic implications. We evaluated the efficacy of immunohistochemistry to predict mutations in KIT. One hundred seventy-three tumors, comprising primary and metastatic melanomas (141 acral lentiginous/mucosal, 5 nodular, 4 lentigo maligna, 3 superficial spreading, 2 uveal, 1 melanoma of soft parts, 8 metastases from unclassified primaries, and 9 metastases from unknown primaries) were studied. Immunohistochemical expression of KIT using an anti-CD117 antibody and KIT mutational analysis by gene sequencing of exons 11, 13, and 17 were performed. Eighty-one percent of acral lentiginous/mucosal melanomas, primary and metastatic, showed KIT expression by at least 5% of the tumor cells. The overall frequency of activating KIT gene mutations in acral lentiginous/mucosal melanomas was 15% (14 out of 91 cases), being the L576P mutation in exon 11 the most frequently detected (4 of 14 cases). Cases showing less than 10% positive tumor cells were negative for KIT mutations. Eighty-two percent (12 of 14) of cases positive for KIT mutation showed KIT expression in more than 50% of the cells. An association between immunohistochemical expression of KIT and mutation status was found (P=0.007). Immunohistochemical expression of KIT in less than 10% of the cells of the   invasive   component of acral lentiginous/mucosal melanomas appears to be a strong negative predictor of KIT mutation and therefore can potentially be used to triage cases for additional KIT genotyping. 
  GeneRIFs  
 Data indicate that p27 expression and microvessel density (MVD) dramatically decreased during the transition from in situ to   invasive   ductal carcinomas (IDC), and a low c-kit expression was indicative of IDC relapse. 
  [Mm]etastasize  PubMed  
 Female adnexal tumor of probable wolffian origin: morphological, immunohistochemical, and ultrastructural study with c-kit gene analysis. Female adnexal tumors of probable wolffian origin (FATWO) are rare neoplasms believed to originate from mesonephric (wolffian) remnants. Rarity and variable location of FATWO make the diagnosis difficult. Although most cases follow a benign clinical course, approximately 10% of them either recur or   metastasize   and are thought to be resistant to chemoradiation therapy. In 2004, imatinib therapy, a tyrosine kinase inhibitor known to be effective against gastrointestinal stromal tumors, was reported to be effective also in a case of KIT-positive FATWO. However, c-kit gene mutations in FATWO have never been studied. Herein is reported the case of a 50-year-old Japanese woman with FATWO arising in the right paratubal site. The tumor had typical characteristics of FATWO in both morphology and immunohistochemistry. KIT protein was diffusely and weakly expressed, but DNA analysis revealed no mutational change in exon 9 or 11 of the c-kit gene. It is believed that accumulation of such genetic data of FATWO are essential from a therapeutic standpoint, although the present case had no mutation. In addition, the cytological features of this rare tumor are presented, which have not been described previously. 
 Increased c-kit (CD117) expression in malignant mammary phyllodes tumors. Mammary phyllodes tumors are uncommon stromal neoplasms, and are divided into benign, borderline and malignant groups basing on histologic criteria. While benign phyllodes tumors may recur, borderline phyllodes tumors show higher propensity to recur locally and rarely   metastasize  , and malignant phyllodes tumors show even higher chances of local recurrences or distant metastases. c-kit is a proto-oncogene that encodes a tyrosine kinase receptor (CD117) and is a marker for gastrointestinal stromal tumors (GIST). With the advent of therapeutic agent targeted at this receptor for GIST, we investigated 179 phyllodes tumors (101 benign, 50 borderline, 28 malignant) for c-kit expression using immunohistochemistry. The staining was compared to the degree of malignancy, and to the degree of stromal cellularity, mitotic activity, nuclear pleomorphism and stromal overgrowth. The overall positive rate for c-kit was 29% (52/179) and 17% (17/101), 24% (12/50) and 46% (13/28), respectively, for benign, borderline malignant and frank malignant phyllodes and the differences between all categories were significant (chi2=13.844, P=0.001). In mammary phyllodes tumors, there was increasing c-kit expression with increasing degree of malignancy, up to 46% in malignant cases. This provides strong evidence that c-kit receptor mediated tyrosine kinase involvement in the pathogenesis of phyllodes tumors, and the therapeutic agent, STI571, Glivec, may be a potentially useful drug for its management. 
 Gastrointestinal stromal tumors with KIT exon 9 mutations: Update on genotype-phenotype correlation and validation of a high-resolution melting assay for mutational testing. KIT exon 9 mutations in gastrointestinal stromal tumors (GISTs) are highly relevant and have direct therapeutic implications. In this context, we established and validated a fast and sensitive high-resolution melting assay. Analyzing 126 primary and 18 metastatic KIT exon 9-mutated cases from our registry, we demonstrate that the mutational spectrum of exon 9 is broader than previously thought and describe 3 novel mutations. Including these cases and the common p.A502_Y503dup mutation, we provide a comprehensive list of all known KIT exon 9 mutations according to the Human Genome Variation Society nomenclature. Two of the newly described mutations were associated with an aggressive phenotype and tumor progression while being treated with 400 mg imatinib, indicating that also GIST with rare exon 9 mutations could be treated with increased imatinib dosage. On the basis of >1500 GISTs from our registry, we have determined the frequency of KIT exon 9 mutations to be 9.2% among all GISTs and 22.5% among small-bowel cases. We describe for the first time that nearly 20% of exon 9-mutated GIST occur in the stomach or rectum. Furthermore, we provide first evidence that exon 9-mutated GISTs   metastasize   significantly more often to the peritoneum than to the liver. Performing extensive statistical analyses on data from our registry and from the literature, we demonstrate that KIT exon 9 mutations are neither associated with intermediate-risk/high-risk status nor overrepresented among metastatic lesions. Thus, we conclude that exon 9 mutations per se do not have prognostic relevance. 
 c-Kit is suppressed in human colon cancer tissue and contributes to L1-mediated metastasis. The transmembrane neural cell adhesion receptor L1 is a Wnt/β-catenin target gene expressed in many tumor types. In human colorectal cancer, L1 localizes preferentially to the invasive front of tumors and when overexpressed in colorectal cancer cells, it facilitates their metastasis to the liver. In this study, we investigated genes that are regulated in human colorectal cancer and by the L1-NF-κB pathway that has been implicated in liver metastasis. c-Kit was the most highly suppressed gene in both colorectal cancer tissue and the L1-NF-κB pathway. c-Kit suppression that resulted from L1-mediated signaling relied upon NF-κB, which directly inhibited the transcription of SP1, a major activator of the c-Kit gene promoter. Reconstituting c-Kit expression in L1-transfected cells blocked the biological effects conferred by L1 overexpression in driving motility and liver metastasis. We found that c-Kit expression in colorectal cancer cells is associated with a more pronounced epithelial morphology, along with increased expression of E-cadherin and decreased expression of Slug. Although c-Kit overexpression inhibited the motility and metastasis of L1-expressing colorectal cancer cells, it enhanced colorectal cancer cell proliferation and tumorigenesis, arguing that separate pathways mediate tumorigenicity and metastasis by c-Kit. Our findings provide insights into how colorectal can  cer metasta  sizes to the liver, the most common site of dissemination in this cancer. 
  [Mm]etastasis  PubMed  
 Deletion of the KIT gene is associated with liver   metastasis   and poor prognosis in patients with gastrointestinal stromal tumor in the stomach. The goal of this study was to investigate the association of mutations in the KIT gene and the platelet-derived growth factor receptor alpha (PDGFRA) gene with clinicopathological features of patients with gastrointestinal stromal tumor (GIST) localized in the stomach. We evaluated 56 gastric GISTs for KIT and PDGFRA mutations. DNA was extracted from paraffin-embedded tumor specimens, and exons 9, 11, 13 and 17 of the KIT gene and exons 12 and 18 of the PDGFRA gene were amplified by polymerase chain reaction and sequenced. The genetic features were then compared with the clinicopathological features. Immunohistochemistry was performed for KIT, CD34, Ki-67 (as a marker of cell proliferation) and CD31 (as a marker of microvessel density), and apoptosis was assessed by in situ DNA nick-end labeling. Thirty-four (61%) of the 56 GISTs had a mutation in exon 11 of KIT, and 2 (4%) had a mutation in exon 13 of KIT. Deletions in exon 11 of KIT were the most common mutation encountered in the present study. No mutations were found in exon 9 or 17 of KIT. Six of the 20 GISTs lacking KIT mutations had a mutation in exon 18 of PDGFRA, and 1 had a mutation in exon 12 of PDGFRA. The KIT mutation-positive GISTs showed more frequent liver metastases and higher mortality than KIT mutation-negative GISTs. Our data indicate that KIT mutations, especially deletions in exon 11, are markers of poor prognosis for gastric GISTs. 
 Deletions affecting codons 557-558 of the c-KIT gene indicate a poor prognosis in patients with completely resected gastrointestinal stromal tumors: a study by the Spanish Group for Sarcoma Research (GEIS). To explore the prognostic value of mutations in c-KIT and PDGFR-alpha genes with respect to relapse-free survival (RFS) in patients with gastrointestinal stromal tumors (GIST). We have investigated the prognostic relevance of the type and position of the mutations, in addition to other clinicopathologic factors, in a large series of patients with GIST.For this study, 162 patients were selected according to the following criteria: completely resected tumors with negative margins attended between 1994 and 2001; no   metastasis   at diagnosis; tumor larger than 2 cm, c-KIT-positive immunostaining; and no other primary tumors.The median follow-up was 42 months for patients free of recurrence. Mutations were detected in 96 tumors (60%): 82 cases involving c-KIT and 14 cases involving PDFGR-alpha. Univariate analysis demonstrated the following as poor prognostic factors for RFS: tumors larger than 10 cm (P < .0001); mitotic count higher than 10 mitoses per 50 high-power fields (P < .0001); high risk index (P < .0001); intestinal GIST location (P = .0041); high cellularity (P < .0001); tumor necrosis (P < .0001); deletions affecting exon 11 (P = .0007); and deletions affecting codons 557 to 558 (P < .0001). After the multivariate analysis, only the high risk index (relative risk [RR], 12.36), high cellularity (RR, 3.97), and deletions affecting codons 557 to 558 of c-KIT (RR, 2.57) corresponded to independent prognostic factors for RFS in GIST patients.Deletions affecting codons 557 to 558 are relevant for the prognosis of RFS in GIST patients. This critical genetic alteration should be considered to be a new prognostic stratification variable for randomized trials exploring imatinib mesylate in the adjuvant setting in GIST patients. 
 Amelanotic malignant melanoma of the esophagus: report of two cases with immunohistochemical and molecular genetic study of KIT and PDGFRA. The author reports herein two cases of amelanotic malignant melanoma of the esophagus. Case 1 is an 87-year-old woman who was admitted to our hospital because of nausea and vomiting. Endoscopic examination revealed an ulcerated tumor of the distal esophagus, and a biopsy was taken. The biopsy showed malignant polygonal and spindle cells. No melanin pigment was recognized. Immunohistochemically, the tumor cells were positive for melanosome (HMB45), S100 protein, KIT and Platelet derived growth factor receptor-alpha (PDGFRA). The patient was treated by chemotherapy and radiation, but died of systemic   metastasis   12 mo after the presentation. Case 2 is a 56-year-old man presenting with dysphagia. Endoscopic examination revealed a polypoid tumor in the middle esophagus, and a biopsy was obtained. The biopsy showed malignant spindle cells without melanin pigment. Immunohistochemically, the tumor cells were positively labeled for melanosome, S100 protein, KIT and PDGFRA. The patient refused operation, and was treated by palliative chemotherapy and radiation. He died of   metastasis   7 mo after the admission. In both cases, molecular genetic analyses of KIT gene (exons 9, 11, 13 and 17) and PDGFRA gene (exons 12 and 18) were performed by the PCR direct sequencing method, which showed no mutations of KIT and PDGFRA genes. This is the first report of esophageal malignant melanoma with an examination of the expression of KIT and PDGFRA and the mutational status of KIT and PDGFRA genes. 
 Detection of mutations in the BRAF gene in patients with KIT and PDGFRA wild-type gastrointestinal stromal tumors. Gastrointestinal stromal tumors (GISTs) are characterized by mutations in exons 9, 11, 13, and 17 of KIT or exons 12, 14, and 18 of PDGFRA gene. However, approximately 10 to 15 % of GISTs lack the mutations in KIT and PDGFRA, and these are referred to as wild-type GISTs which are less sensitive to tyrosine-kinase inhibitors. The aim of this study was to detect BRAF mutations in patients with wild-type GISTs. We applied a sensitive allele-specific PCR, which was optimized using the V600E mutation-harboring cell line RKO, followed by verification of the results by dideoxy sequencing. We selected 149 GIST patients without detectable mutations in KIT and PDGFRA genes from the Slovak national GIST register and analyzed biopsy specimens for the presence of BRAF mutations in exon 15. We identified nine patients with the V600E mutation. The BRAF-driven GISTs were primary gastric (n = 3), small intestinal (n = 3), colon (n = 1), and of uncertain origin (n = 1). We also included   a liver m  etastasis of a patient with a simultaneous KIT exon 11-mutated intra-a  bdominal m  etastasis. We conclude that genome analysis of wild-type GISTs for mutations should include the BRAF gene, as its mutation status contributes to understanding of pathogenesis and might be important for decisions on therapy. 
 Prognostic value of KIT/PDGFRA mutations in gastrointestinal stromal tumors: a meta-analysis. The postulated relationship between KIT/PDGFRA mutations and their prognostic value in gastrointestinal stromal tumors (GISTs) has generated intense attention during the past decade, despite the fact that a great deal of studies have been conducted on this subject. To provide a strong quantitative estimate of this postulated relationship, we carried out a meta-analysis which combined, compared, and summarized the results of existing relevant studies.Studies were identified by searching databases and reviewing citations in relevant articles. Of 48 potentially relevant studies, we combined individual patient data from 18 studies which involved 1,487 patients with GISTs, by which we made a comparison between the positive KIT mutation subgroup and the negative KIT mutation subgroup (PDGFRA mutation and wild type). We tabulated and analyzed the patient characteristics from each study, including general information such as age and gender, histopathological parameters, and clinical follow-up outcomes.KIT mutations, compared with PDGFRA mutations and wild type, showed a marked increased risk not only for tumor size (>5 cm) but also for higher mitotic activity (>5), suggesting that KIT mutations significantly correlated with the National Comprehensive Cancer Network (NCCN) high risk or National Institutes of Health (NIH) high risk (1.74 (95% CI, 1.20 to 2.53) and 2.00 (95% CI, 1.08 to 3.68), respectively). Moreover, higher recurrence and   metastasis   was observed in GISTs with KIT mutations, revealing its closer correlation with clinical malignant risk (P<0.001 for each, with odds ratio (OR) of 2.06 (95%, 1.37 to 3.11) and 2.77 (95%, 1.64 to 4.67), respectively). High risk or malignant GISTs with KIT mutations had a significantly poorer prognosis, as measured by 3-year overall survival, compared to those with PDGFRA mutations and wild type (0.47 (95% CI, 0.25 to 0.90)).KIT mutations, compared with PDGFRA mutations and wild type, represent a poorer prognostic marker in high risk or malignant GISTs. 
 Expression of platelet-derived growth factor-alpha receptor and c-kit in giant cell tumours of bone. Platelet-derived growth factor-alpha receptor (PDGFRA) and c-kit are tyrosine kinase receptors expressed in many neoplasms. We investigated protein expression in 34 giant cell tumours of bone and two specimens of lung metastases.The expression of PDGFRA and c-kit was analysed by immunohistochemistry. Additionally, capillary sequencing of genomic DNA was performed to search for mutations in therapeutically relevant exons 12 and 18 of the PDGFRA gene and exons 9 and 11 of the c-kit gene.PDGFRA expression was detected in all specimens and all cellular components such as reactive osteoclast-like giant cells and neoplastic stromal cells. C-kit expression was found in six cases of giant cell tumour and one specimen of lung   metastasis   in all cellular components ranging from 5 to 50% (mean 4.8%, SD 12.4). A further 13 cases showed labelling of osteoclast-like giant cells. DNA sequence of exons 12 and 18 of the PDGFRA gene and exons 9 and 11 of the c-kit gene were not altered in these tumours.PDGFRA and c-kit play a functional role in the growth of giant cell tumours of bone but genetic changes of therapeutically relevant exons of the PDGFRA gene and the c-kit gene are not seen in these tumours. 
 The analysis of status and clinical implication of KIT and PDGFRA mutations in gastrointestinal stromal tumor (GIST). To analyze the frequency and spectrum of KIT and platelet-derived growth factor receptor-alpha (PDGFRA) gene in a large series of study and to explore the clinical implication of mutations in the gastrointestinal stromal tumors (GISTs) in China.A total of 141 GISTs were subject to mutation analysis of KIT (exons 9, 11, 13, and 17) and PDGFRA (exons 12 and 18) using PCR amplification and direct sequencing. Clinicopathologic characteristics were correlated to gene mutations.Of the 141 tumors studied, approximately 76.6% had KIT gene mutations, 2.8% had PDGFRA gene mutations and 20.6% had a wild-type gene of KIT and PDGFRA. Among those with KIT gene mutations, 70.2% occurred in exon 11, 5.7% in exon 9, 0.7% in exon 13, and no mutation was detected in exons 17. The most frequent sites of mutation were in exon 11 and the mutations clustered in the classic "hot spot" at the 5'end of the exon mostly heterogeneous and the second "hot spot" were internal tandem duplications (ITD) at the 3'end of the exon. The overall mutation rate was significantly lower in GISTs originated from colorectum or extra-gastrointestinal tract (chi(2) = 6.728, P = 0.009; chi(2) = 4.059, P = 0.044), however, mutation rate on exon 11 was significantly increased in gastric stromal tumor (chi(2) = 5.713, P = 0.017; chi(2) = 4.341, P = 0.037). There were no significant differences in terms of age, gender, tumor size, mitotic counts, grade of malignant potential and liver   metastasis   in patients with or without gene mutations.KIT and PDGFRA gene were frequently found in patients with GISTs. Gene mutation rate varies in originated organ. 
 KIT and PDGFRA mutations and PDGFRA immunostaining in gastrointestinal stromal tumors. In the present study, we investigated the association of PDGFRA and KIT mutations as well as PDGFRA immunohistochemical expression with clinicopathologic features and prognosis in a series of gastrointestinal stromal tumors (GISTs). Tumor DNA from 40 GISTs was sequenced for the presence of mutations in KIT exons 9, 11, 13 and 17, and in PDGFRA exons 12 and 18. Tissue sections were stained with polyclonal anti-PDGFRA antibody. KIT mutations occurred in 26 cases. There were 13 deletions, 6 substitutions, 3 deletion-substitutions, 3 duplications and 1 insertion. Tumors with KIT deletions/insertion were large with a high mitotic index (MI), and were associated with a high rate of symptoms at diagnosis, invasion into adjacent organs, distant   metastasis  , relapse and a short disease-free survival (DFS). PDGFRA mutations occurred in 6 gastric GISTs. There were 4 deletions and 2 substitutions. Tumors with PDGFRA mutations were small, with a low MI and Ki67 score, and were associated with a very low rate of symptoms at diagnosis, invasion into adjacent organs and distant   metastasis  . PDGFRA immunopositivity was found in 23 cases: a peculiar 'dotlike' staining was found in 5 out of 6 PDGFRA mutated cases. Patients with positive PDGFRA immunostaining had a longer DFS than those with negative staining. Our data confirm that the type of KIT mutation is associated with various clinicopathologic features of GISTs, and indicate that PDGFRA mutations are associated with rather indolent tumors. PDGFRA immunopositivity reflects PDGFRA mutational status and is associated with a favorable outcome. 
 Fluorescence in situ hybridization analysis and immunophenotyping of c-Kit/PDGFRA and Bcl-2 expression in gastrointestinal stromal tumors. To investigate c-Kit/PDGFRA genomic alterations, KIT-PDGFRA coexpression in gastrointestinal stromal tumors (GISTs) and the role of Bcl-2.We analyzed 70 primary tumors, 6 recurrences, 4 metastases and 1 recurrence plus   metastasis  , all molecularly characterized. Alterations in gene copy number were detected by fluorescence in situ hybridization (FISH) and expression of KIT, PDGFRA and Bcl-2 by immunohistochemistry.c-Kit/PDGFRA gene alterations affected 38% of all cases and 39% of primary tumors, with major changes accounting for 15% in both all the cases and primary tumors. Cytoplasmic KIT/PDGFRA coexpression was present in 96.5% of the c-Kit-mutated cases, 100% of the wt c-Kit/PDGFRA cases and 66.6% of the PDGFRA-mutated cases. Bcl-2 immunoreactivity was present in 70% of cases, with expression levels of +++ in 29%, ++ in 38% and + in 33%.FISH confirmed cytogenetic alterations in about 40% of primary GISTs at the onset. The high rate of c-Kit/PDGFRA coexpression suggests that both receptors are involved in oncogenicity and may affect imatinib efficacy. The assumption that Bcl-2 expression is supported by the KIT pathway and that its imatinib-mediated down-regulation contributes to autophagic cell death, although attractive, needs to be further confirmed. 
 Potential role of imatinib mesylate (Gleevec, STI-571) in the treatment of vestibular schwannoma. To determine the expression of the tyrosine kinases platelet-derived growth factor receptor (PDGFR) and c-Kit in vestibular schwannoma (VS) and to determine the potential role of imatinib mesylate (Gleevec) in regulating the growth and cell death of this tumor.Protein tyrosine kinases are transmembrane tyrosine kinase receptors that transduce signals from inside and outside the cell and function as relay points for signaling pathways. They have a key role in numerous processes that affect cell proliferation, tumorigenesis, cancer invasion,   metastasis  , and modulation of apoptosis. A few of these kinases have been demonstrated to be overexpressed and dysregulated in many carcinomas, sarcomas, and benign tumors.Immunohistochemical staining was used to investigate the expression of PDGFR and c-Kit in archived acoustic neuroma tissue. Clinical data including size of tumors, age, sex, and symptoms were correlated with kinase expression, whereas Western blot analysis and immunofluorescence were performed to demonstrate the expression and localization of PDGFR and c-Kit in HEI193, an immortalized VS cell line. Clonogenic survival assays were performed to assess proliferation inhibition by Gleevec. Gleevec's effect on the cell cycle profile also was investigated via flow cytometry analysis.Expression of PDGFR in the formalin-fixed VS tumor tissue was observed in 23 (67.5%) of the 34 samples. C-kit was expressed in 18 (52.9%) of the 34 samples. Western blot analysis demonstrates positive expression of c-Kit and PDGFR-Q in HEI193 and a primary VS culture. Western blot analysis showed downregulation of phospho-c-kit and phospho-PDGFR-Q with 5 and 10 uM Gleevec. Immunofluorescent staining of this cell line also reveals that PDGFR-β is localized primarily in the cytoplasm, whereas c-Kit is both nuclear and cytoplasmic. Cell cycle analysis of HEI193 96 hours after incubation with Gleevec indicates a dose-dependent increase in G1 from 61.6% to 70.7% and 74% at 5 and 10 uM of Gleevec, respectively. Colony formation assays demonstrate dose-dependent growth inhibition by Gleevec, in the HEI193 cell line as well as in a VS cell culture derived from a fresh tumor.The expression of PDGFR-Q and c-Kit in VS tissue may indicate novel molecular targets involved in the development of this tumor. Direct inhibition of these molecules by Gleevec may have relevant therapeutic applications. 
 Detection of mutations in the BRAF gene in patients with KIT and PDGFRA wild-type gastrointestinal stromal tumors. Gastrointestinal stromal tumors (GISTs) are characterized by mutations in exons 9, 11, 13, and 17 of KIT or exons 12, 14, and 18 of PDGFRA gene. However, approximately 10 to 15 % of GISTs lack the mutations in KIT and PDGFRA, and these are referred to as wild-type GISTs which are less sensitive to tyrosine-kinase inhibitors. The aim of this study was to detect BRAF mutations in patients with wild-type GISTs. We applied a sensitive allele-specific PCR, which was optimized using the V600E mutation-harboring cell line RKO, followed by verification of the results by dideoxy sequencing. We selected 149 GIST patients without detectable mutations in KIT and PDGFRA genes from the Slovak national GIST register and analyzed biopsy specimens for the presence of BRAF mutations in exon 15. We identified nine patients with the V600E mutation. The BRAF-driven GISTs were primary gastric (n = 3), small intestinal (n = 3), colon (n = 1), and of uncertain origin (n = 1). We also included   a liver m  etastasis of a patient with a simultaneous KIT exon 11-mutated intra-a  bdominal m  etastasis. We conclude that genome analysis of wild-type GISTs for mutations should include the BRAF gene, as its mutation status contributes to understanding of pathogenesis and might be important for decisions on therapy. 
 Prognostic value of KIT/PDGFRA mutations in gastrointestinal stromal tumors: a meta-analysis. The postulated relationship between KIT/PDGFRA mutations and their prognostic value in gastrointestinal stromal tumors (GISTs) has generated intense attention during the past decade, despite the fact that a great deal of studies have been conducted on this subject. To provide a strong quantitative estimate of this postulated relationship, we carried out a meta-analysis which combined, compared, and summarized the results of existing relevant studies.Studies were identified by searching databases and reviewing citations in relevant articles. Of 48 potentially relevant studies, we combined individual patient data from 18 studies which involved 1,487 patients with GISTs, by which we made a comparison between the positive KIT mutation subgroup and the negative KIT mutation subgroup (PDGFRA mutation and wild type). We tabulated and analyzed the patient characteristics from each study, including general information such as age and gender, histopathological parameters, and clinical follow-up outcomes.KIT mutations, compared with PDGFRA mutations and wild type, showed a marked increased risk not only for tumor size (>5 cm) but also for higher mitotic activity (>5), suggesting that KIT mutations significantly correlated with the National Comprehensive Cancer Network (NCCN) high risk or National Institutes of Health (NIH) high risk (1.74 (95% CI, 1.20 to 2.53) and 2.00 (95% CI, 1.08 to 3.68), respectively). Moreover, higher recurrence and   metastasis   was observed in GISTs with KIT mutations, revealing its closer correlation with clinical malignant risk (P<0.001 for each, with odds ratio (OR) of 2.06 (95%, 1.37 to 3.11) and 2.77 (95%, 1.64 to 4.67), respectively). High risk or malignant GISTs with KIT mutations had a significantly poorer prognosis, as measured by 3-year overall survival, compared to those with PDGFRA mutations and wild type (0.47 (95% CI, 0.25 to 0.90)).KIT mutations, compared with PDGFRA mutations and wild type, represent a poorer prognostic marker in high risk or malignant GISTs. 
 KIT and PDGFRA mutations and PDGFRA immunostaining in gastrointestinal stromal tumors. In the present study, we investigated the association of PDGFRA and KIT mutations as well as PDGFRA immunohistochemical expression with clinicopathologic features and prognosis in a series of gastrointestinal stromal tumors (GISTs). Tumor DNA from 40 GISTs was sequenced for the presence of mutations in KIT exons 9, 11, 13 and 17, and in PDGFRA exons 12 and 18. Tissue sections were stained with polyclonal anti-PDGFRA antibody. KIT mutations occurred in 26 cases. There were 13 deletions, 6 substitutions, 3 deletion-substitutions, 3 duplications and 1 insertion. Tumors with KIT deletions/insertion were large with a high mitotic index (MI), and were associated with a high rate of symptoms at diagnosis, invasion into adjacent organs, distant   metastasis  , relapse and a short disease-free survival (DFS). PDGFRA mutations occurred in 6 gastric GISTs. There were 4 deletions and 2 substitutions. Tumors with PDGFRA mutations were small, with a low MI and Ki67 score, and were associated with a very low rate of symptoms at diagnosis, invasion into adjacent organs and distant   metastasis  . PDGFRA immunopositivity was found in 23 cases: a peculiar 'dotlike' staining was found in 5 out of 6 PDGFRA mutated cases. Patients with positive PDGFRA immunostaining had a longer DFS than those with negative staining. Our data confirm that the type of KIT mutation is associated with various clinicopathologic features of GISTs, and indicate that PDGFRA mutations are associated with rather indolent tumors. PDGFRA immunopositivity reflects PDGFRA mutational status and is associated with a favorable outcome. 
 Fluorescence in situ hybridization analysis and immunophenotyping of c-Kit/PDGFRA and Bcl-2 expression in gastrointestinal stromal tumors. To investigate c-Kit/PDGFRA genomic alterations, KIT-PDGFRA coexpression in gastrointestinal stromal tumors (GISTs) and the role of Bcl-2.We analyzed 70 primary tumors, 6 recurrences, 4 metastases and 1 recurrence plus   metastasis  , all molecularly characterized. Alterations in gene copy number were detected by fluorescence in situ hybridization (FISH) and expression of KIT, PDGFRA and Bcl-2 by immunohistochemistry.c-Kit/PDGFRA gene alterations affected 38% of all cases and 39% of primary tumors, with major changes accounting for 15% in both all the cases and primary tumors. Cytoplasmic KIT/PDGFRA coexpression was present in 96.5% of the c-Kit-mutated cases, 100% of the wt c-Kit/PDGFRA cases and 66.6% of the PDGFRA-mutated cases. Bcl-2 immunoreactivity was present in 70% of cases, with expression levels of +++ in 29%, ++ in 38% and + in 33%.FISH confirmed cytogenetic alterations in about 40% of primary GISTs at the onset. The high rate of c-Kit/PDGFRA coexpression suggests that both receptors are involved in oncogenicity and may affect imatinib efficacy. The assumption that Bcl-2 expression is supported by the KIT pathway and that its imatinib-mediated down-regulation contributes to autophagic cell death, although attractive, needs to be further confirmed. 
 Potential role of imatinib mesylate (Gleevec, STI-571) in the treatment of vestibular schwannoma. To determine the expression of the tyrosine kinases platelet-derived growth factor receptor (PDGFR) and c-Kit in vestibular schwannoma (VS) and to determine the potential role of imatinib mesylate (Gleevec) in regulating the growth and cell death of this tumor.Protein tyrosine kinases are transmembrane tyrosine kinase receptors that transduce signals from inside and outside the cell and function as relay points for signaling pathways. They have a key role in numerous processes that affect cell proliferation, tumorigenesis, cancer invasion,   metastasis  , and modulation of apoptosis. A few of these kinases have been demonstrated to be overexpressed and dysregulated in many carcinomas, sarcomas, and benign tumors.Immunohistochemical staining was used to investigate the expression of PDGFR and c-Kit in archived acoustic neuroma tissue. Clinical data including size of tumors, age, sex, and symptoms were correlated with kinase expression, whereas Western blot analysis and immunofluorescence were performed to demonstrate the expression and localization of PDGFR and c-Kit in HEI193, an immortalized VS cell line. Clonogenic survival assays were performed to assess proliferation inhibition by Gleevec. Gleevec's effect on the cell cycle profile also was investigated via flow cytometry analysis.Expression of PDGFR in the formalin-fixed VS tumor tissue was observed in 23 (67.5%) of the 34 samples. C-kit was expressed in 18 (52.9%) of the 34 samples. Western blot analysis demonstrates positive expression of c-Kit and PDGFR-Q in HEI193 and a primary VS culture. Western blot analysis showed downregulation of phospho-c-kit and phospho-PDGFR-Q with 5 and 10 uM Gleevec. Immunofluorescent staining of this cell line also reveals that PDGFR-β is localized primarily in the cytoplasm, whereas c-Kit is both nuclear and cytoplasmic. Cell cycle analysis of HEI193 96 hours after incubation with Gleevec indicates a dose-dependent increase in G1 from 61.6% to 70.7% and 74% at 5 and 10 uM of Gleevec, respectively. Colony formation assays demonstrate dose-dependent growth inhibition by Gleevec, in the HEI193 cell line as well as in a VS cell culture derived from a fresh tumor.The expression of PDGFR-Q and c-Kit in VS tissue may indicate novel molecular targets involved in the development of this tumor. Direct inhibition of these molecules by Gleevec may have relevant therapeutic applications. 
 Expression of platelet-derived growth factor-alpha receptor and c-kit in giant cell tumours of bone. Platelet-derived growth factor-alpha receptor (PDGFRA) and c-kit are tyrosine kinase receptors expressed in many neoplasms. We investigated protein expression in 34 giant cell tumours of bone and two specimens of lung metastases.The expression of PDGFRA and c-kit was analysed by immunohistochemistry. Additionally, capillary sequencing of genomic DNA was performed to search for mutations in therapeutically relevant exons 12 and 18 of the PDGFRA gene and exons 9 and 11 of the c-kit gene.PDGFRA expression was detected in all specimens and all cellular components such as reactive osteoclast-like giant cells and neoplastic stromal cells. C-kit expression was found in six cases of giant cell tumour and one specimen of lung   metastasis   in all cellular components ranging from 5 to 50% (mean 4.8%, SD 12.4). A further 13 cases showed labelling of osteoclast-like giant cells. DNA sequence of exons 12 and 18 of the PDGFRA gene and exons 9 and 11 of the c-kit gene were not altered in these tumours.PDGFRA and c-kit play a functional role in the growth of giant cell tumours of bone but genetic changes of therapeutically relevant exons of the PDGFRA gene and the c-kit gene are not seen in these tumours. 
 Amelanotic malignant melanoma of the esophagus: report of two cases with immunohistochemical and molecular genetic study of KIT and PDGFRA. The author reports herein two cases of amelanotic malignant melanoma of the esophagus. Case 1 is an 87-year-old woman who was admitted to our hospital because of nausea and vomiting. Endoscopic examination revealed an ulcerated tumor of the distal esophagus, and a biopsy was taken. The biopsy showed malignant polygonal and spindle cells. No melanin pigment was recognized. Immunohistochemically, the tumor cells were positive for melanosome (HMB45), S100 protein, KIT and Platelet derived growth factor receptor-alpha (PDGFRA). The patient was treated by chemotherapy and radiation, but died of systemic   metastasis   12 mo after the presentation. Case 2 is a 56-year-old man presenting with dysphagia. Endoscopic examination revealed a polypoid tumor in the middle esophagus, and a biopsy was obtained. The biopsy showed malignant spindle cells without melanin pigment. Immunohistochemically, the tumor cells were positively labeled for melanosome, S100 protein, KIT and PDGFRA. The patient refused operation, and was treated by palliative chemotherapy and radiation. He died of   metastasis   7 mo after the admission. In both cases, molecular genetic analyses of KIT gene (exons 9, 11, 13 and 17) and PDGFRA gene (exons 12 and 18) were performed by the PCR direct sequencing method, which showed no mutations of KIT and PDGFRA genes. This is the first report of esophageal malignant melanoma with an examination of the expression of KIT and PDGFRA and the mutational status of KIT and PDGFRA genes. 
 The analysis of status and clinical implication of KIT and PDGFRA mutations in gastrointestinal stromal tumor (GIST). To analyze the frequency and spectrum of KIT and platelet-derived growth factor receptor-alpha (PDGFRA) gene in a large series of study and to explore the clinical implication of mutations in the gastrointestinal stromal tumors (GISTs) in China.A total of 141 GISTs were subject to mutation analysis of KIT (exons 9, 11, 13, and 17) and PDGFRA (exons 12 and 18) using PCR amplification and direct sequencing. Clinicopathologic characteristics were correlated to gene mutations.Of the 141 tumors studied, approximately 76.6% had KIT gene mutations, 2.8% had PDGFRA gene mutations and 20.6% had a wild-type gene of KIT and PDGFRA. Among those with KIT gene mutations, 70.2% occurred in exon 11, 5.7% in exon 9, 0.7% in exon 13, and no mutation was detected in exons 17. The most frequent sites of mutation were in exon 11 and the mutations clustered in the classic "hot spot" at the 5'end of the exon mostly heterogeneous and the second "hot spot" were internal tandem duplications (ITD) at the 3'end of the exon. The overall mutation rate was significantly lower in GISTs originated from colorectum or extra-gastrointestinal tract (chi(2) = 6.728, P = 0.009; chi(2) = 4.059, P = 0.044), however, mutation rate on exon 11 was significantly increased in gastric stromal tumor (chi(2) = 5.713, P = 0.017; chi(2) = 4.341, P = 0.037). There were no significant differences in terms of age, gender, tumor size, mitotic counts, grade of malignant potential and liver   metastasis   in patients with or without gene mutations.KIT and PDGFRA gene were frequently found in patients with GISTs. Gene mutation rate varies in originated organ. 
 Deletions affecting codons 557-558 of the c-KIT gene indicate a poor prognosis in patients with completely resected gastrointestinal stromal tumors: a study by the Spanish Group for Sarcoma Research (GEIS). To explore the prognostic value of mutations in c-KIT and PDGFR-alpha genes with respect to relapse-free survival (RFS) in patients with gastrointestinal stromal tumors (GIST). We have investigated the prognostic relevance of the type and position of the mutations, in addition to other clinicopathologic factors, in a large series of patients with GIST.For this study, 162 patients were selected according to the following criteria: completely resected tumors with negative margins attended between 1994 and 2001; no   metastasis   at diagnosis; tumor larger than 2 cm, c-KIT-positive immunostaining; and no other primary tumors.The median follow-up was 42 months for patients free of recurrence. Mutations were detected in 96 tumors (60%): 82 cases involving c-KIT and 14 cases involving PDFGR-alpha. Univariate analysis demonstrated the following as poor prognostic factors for RFS: tumors larger than 10 cm (P < .0001); mitotic count higher than 10 mitoses per 50 high-power fields (P < .0001); high risk index (P < .0001); intestinal GIST location (P = .0041); high cellularity (P < .0001); tumor necrosis (P < .0001); deletions affecting exon 11 (P = .0007); and deletions affecting codons 557 to 558 (P < .0001). After the multivariate analysis, only the high risk index (relative risk [RR], 12.36), high cellularity (RR, 3.97), and deletions affecting codons 557 to 558 of c-KIT (RR, 2.57) corresponded to independent prognostic factors for RFS in GIST patients.Deletions affecting codons 557 to 558 are relevant for the prognosis of RFS in GIST patients. This critical genetic alteration should be considered to be a new prognostic stratification variable for randomized trials exploring imatinib mesylate in the adjuvant setting in GIST patients. 
 Deletion of the KIT gene is associated with liver   metastasis   and poor prognosis in patients with gastrointestinal stromal tumor in the stomach. The goal of this study was to investigate the association of mutations in the KIT gene and the platelet-derived growth factor receptor alpha (PDGFRA) gene with clinicopathological features of patients with gastrointestinal stromal tumor (GIST) localized in the stomach. We evaluated 56 gastric GISTs for KIT and PDGFRA mutations. DNA was extracted from paraffin-embedded tumor specimens, and exons 9, 11, 13 and 17 of the KIT gene and exons 12 and 18 of the PDGFRA gene were amplified by polymerase chain reaction and sequenced. The genetic features were then compared with the clinicopathological features. Immunohistochemistry was performed for KIT, CD34, Ki-67 (as a marker of cell proliferation) and CD31 (as a marker of microvessel density), and apoptosis was assessed by in situ DNA nick-end labeling. Thirty-four (61%) of the 56 GISTs had a mutation in exon 11 of KIT, and 2 (4%) had a mutation in exon 13 of KIT. Deletions in exon 11 of KIT were the most common mutation encountered in the present study. No mutations were found in exon 9 or 17 of KIT. Six of the 20 GISTs lacking KIT mutations had a mutation in exon 18 of PDGFRA, and 1 had a mutation in exon 12 of PDGFRA. The KIT mutation-positive GISTs showed more frequent liver metastases and higher mortality than KIT mutation-negative GISTs. Our data indicate that KIT mutations, especially deletions in exon 11, are markers of poor prognosis for gastric GISTs. 
 Expression of CD133 and CD117 in 64 Serous Ovarian Cancer Cases. The cancer stem cells (CSCs) represent a minority of tumor cells that are able to proliferate and self-renew and might be responsible for tumor initiation and maintenance. The CD133 and CD117 are the most commonly used markers for the putative CSCs, especially for the ovarian CSCs, but its clinical significance remains uncertain. The aim of this study was to compare the immunohistochemical expression of CD133 and CD117 in 64 primary ovarian high grade serous carcinoma and peritoneal   metastasis  , and to examine their potential clinical role. CD133 expression was mainly seen in the apical/endoluminal cell surface of tumor cells and was found in 61% of the carcinoma samples and 41% of the   metastasis  . The median of CD133 positive cells in tumors was 1 (0.1-7)%, and in metastases was 0.6 (0.1-6)%. CD117 expression appeared as a cytoplasmic and/or membranous stain and was found in 81% of the carcinoma samples and 77% of the   metastasis  . The median of CD117 positive cells in tumors was 1 (0.1-8)%, and in metastases was 0.1 (0.1-7)%. Multivariate analysis has shown that patients with high CD133 expression in tumor cells have significantly shorter disease free survival and overall survival (p=0.025 and p=0.014, respectively). Patients with high CD117 expression in tumor cells have significantly shorter disease free survival (p=0.031). Cox's proportional hazards model identified expression of CD133 protein in tumor as an independent prognostic factor. Our study indicates that the immunohistochemical assessment of CD133 and CD117 expression may have potential clinical value in predicting disease progression and prognosis in the high grade serous ovarian cancer. CD133 proved to be an independent prognostic factor in the high grade serous ovarian cancer patients. 
 Expression of c-Kit, p-ERK and cyclin D1 in malignant melanoma: an immunohistochemical study and analysis of prognostic value. The mitogen-activated protein kinase (MAPK) signaling pathway is one of the major cascades that are crucial for the initiation and progression of melanoma; however, the influence of these signaling molecules on patient survival has not been clarified.The purpose of this study was to analyze the protein expression of MAPK signaling molecules in melanoma, and to correlate the expression status with clinicopathologic parameters.Expression of c-Kit, phosphorylated ERK (p-ERK), and cyclin D1 was examined by immunohistochemistry in 78 primary melanomas, 24 metastatic lesions, and in 42 benign nevi. The following clinicopathologic variables were evaluated: age, gender, histologic type, tumor site, Breslow thickness, Clark's level, ulceration, and survival period. Statistical analyses were performed for assessment of associations and melanoma-specific survival.The expression of c-Kit, p-ERK, and cyclin D1 was significantly higher in primary melanomas than in nevi. c-Kit immunoreactivity was highest in thin (Tis-pT2) melanomas, and showed a significant reduction with tumor progression and   metastasis  . The expression of p-ERK was high in all stages of melanoma. Cyclin D1 positivity increased significantly according to tumor progression, but decreased in metastases. A significant correlation between p-ERK and cyclin D1 expression was observed. Survival analysis failed to detect any trends towards shorter or longer survival among patients expressing either c-Kit, p-ERK or cyclin D1.The expression of c-Kit, p-ERK, and cyclin D1 might help to differentiate thin melanoma from melanocytic nevus, but it appears to lack prognostic potential. 
 Merkel cell carcinoma: correlation of KIT expression with survival and evaluation of KIT gene mutational status. Merkel cell carcinoma is one of the most aggressive primary cutaneous malignancies. Because some Merkel cell carcinomas express the receptor tyrosine kinase KIT, we aimed to evaluate the correlation of KIT expression with the outcome and the presence of activating mutations in the KIT gene in Merkel cell carcinoma. A total of 49 tumors from 40 patients with a diagnosis of Merkel cell carcinoma were identified, of which 30 cases from 21 patients were used in the study. KIT expression was assessed by immunohistochemistry on formalin-fixed, paraffin-embedded material. Cases were divided into low expressors (0-1+ staining intensity) and high expressors (2-3+ staining intensity). Direct sequencing of exons 9, 11, 13, 17, and 18 of the KIT gene spanning the extracellular, juxtamembrane, and tyrosine kinase domains was performed for cases with high KIT expression. Thirty tumors from 21 patients were analyzed for KIT expression. High KIT expression was seen in 67% of the patients. Five-year survival rates in tumors expressing high versus low levels of KIT were 0% versus 57.8%, respectively; however, this dramatic difference did not reach statistical significance (P = .07). A total of 4 point mutations were identified in 18 tumors analyzed. Two of these were silent mutations involving exons 17 and 18, and 2 involved intron 16-17. Two of the identified mutations may represent novel polymorphisms. Our work suggests a correlation between KIT expression and a worse prognosis in Merkel cell carcinoma patients, raising the possibility of an active role of this receptor in tumor progression and   metastasis  . However, we did not identify KIT activating mutations in any of the tumors analyzed. 
 Effect of loss of heterozygosity of the c-kit gene on prognosis after hepatectomy for metastatic liver gastrointestinal stromal tumors. The authors have previously reported that loss of heterozygosity (LOH) of the c-kit gene could be responsible for the gain in high proliferative activity in some gastrointestinal stromal tumors (GIST), resulting in enhanced metastatic potential. In the present study, an attempt was made to identify the factors that might predict the postoperative prognosis of patients with metastatic liver GIST. The clinicopathologic or genetic features of resected liver GIST in 14 patients who had undergone a hepatectomy for metachronous liver metastases and who had not received adjuvant imatinib treatment were examined. LOH of the c-kit gene was observed in seven of 12 metastatic liver GIST (58.3%), of which DNA suitable for testing could be extracted. Ten patients had recurrence after hepatectomy and four had none. The median post-recurrent disease-free survival (PRDFS) after hepatectomy was 27.5 months (range 8-104). The tumor-specific PRDFS was examined using clinicopathologic features, c-kit mutation and LOH of the c-kit gene. No single clinicopathologic or genetic finding was significantly associated with PRDFS. However, patients with 'Ki67 labeling index <5% and LOH(-)' had a significantly longer PRDFS than those with 'Ki67 >/=5% or LOH(+)' (P = 0.032), and there was no correlation between the presence of LOH of the c-kit gene and the Ki67 labeling index. LOH of the c-kit gene in metastatic liver seems to be a common event, and LOH of the c-kit gene in resected liver GIST may be a helpful factor in the prediction of the post-recurrent prognosis of patients with liver   metastasis  . 
 KIT-negative gastrointestinal stromal tumor of the abdominal soft tissue: a clinicopathologic and genetic study of 10 cases. Gastrointestinal stromal tumor (GIST) typically occurs in the gastrointestinal (GI) tract, and expresses KIT protein that is associated with KIT or platelet-derived growth factor receptor-α (PDGFRA) gene mutation. Extragastrointestinal stromal tumors (EGISTs) are a minor subset of GIST that occurs in the soft tissue outside the GI tract, and in very rare cases, these tumors can be KIT negative. We examined the clinicopathologic and molecular characteristics of 10 cases of KIT-negative EGIST by using immunohistochemical staining and gene mutation analysis. The tumors occurred in the omentum (n=5), mesentery (n=2), retroperitoneum (n=1), pelvic cavity (n=1), and not otherwise specified regions of the abdominal cavity (n=1). They ranged from 4 to 33 cm (median, 15 cm) in maximum diameter with relatively low mitotic counts (median, 3.5 per 50 high-power fields). Morphologically, most cases were of epithelioid cell (n=9) or mixed epithelioid and spindle cell (n=1) type, accompanied by variable amounts of myxoid stroma. By immunohistochemical staining, the tumors were positive for CD34 (80%), protein kinase C (PKC) θ (90%), and discovered on GIST-1 (DOG1) (90%), but were negative for KIT (0%). The majority of the examined cases (7 of 9 cases; 78%) had PDGFRA mutations in exon 12 (n=1) or exon 18 (n=6). One case (11%) had a mutation in KIT exon 11, and the remaining 1 had no mutation in either KIT or PDGFRA. Distan  t metastas  is and local recurrence occurred in 1 (10%) and 2 (20%) patients, respectively, and adverse outcome was correlated with larger (>10 cm) tumor size and high mitotic counts (>5/50 high-power fields). Therefore, KIT-negative EGISTs can be characterized by preferential omental origin, epithelioid cell type, low mitotic activity, and mutation of the PDGFRA gene, and these features are similar to those of KIT-negative gastric GISTs. As KIT-negative EGISTs should be considered to be a potential abdominal soft tissue neoplasm, immunohistochemical staining panel and molecular analysis are necessary not only to confirm the diagnosis but also to determine the therapeutic strategy. 
 Proposed histopathologic grading system derived from a study of KIT and CK19 expression in pancreatic endocrine neoplasm. Predicting the biologic behavior of pancreatic endocrine neoplasm in the absence of local invasion or   metastasis   is difficult. We recently proposed an immunohistochemical classification system based on a combination of KIT and CK19 expression: low risk (KIT-/CK19-), intermediate risk (KIT-/CK19+), and high risk (KIT+/CK19+). In the current study, we sought histopathologic features that correlated with the immunohistochemical categories and that could be used in predicting tumor behavior. We assessed histologic findings of 97 pancreatic endocrine neoplasm that had been classified into 3 risk groups based on KIT/CK19 expression. Clinicopathologic associations with prognosis were evaluated using Cox proportional hazards regression models. Tumor size, mitoses, infiltrative border, extrapancreatic extension, perineural invasion, and presence of amyloid were significantly different among the 3 risk groups, and tumor necrosis approached statistical significance as well (P = .089). A scoring system using mitoses, necrosis, and tumor border was developed as follows: mitosis: 0 (0/50 high-power field), 1 (1-3/50 high-power field), and 2 (≥4/50 high-power field); necrosis: 0 (absent) and 1 (present); tumor border: 0 (noninfiltrating) and 1 (infiltrating), giving a possible histology score of 0 to 4. Although there was an overall difference in disease-specific survival among the 4 histology scores (P < .001), there was not a statistically significant difference between patients with scores of 0 and 1 or between patients with scores of 3 and 4. Therefore, a 3-tied grading system was developed by combining score 0 and 1 tumors as grade 1, score 2 tumors as grade 2, and score 3 and 4 tumors as grade 3. There was a significant difference in survival, tumo  r metastas  is, tumor recurrence, and functioning status among the 3 grades. By analyzing histopathologic features in KIT/CK19 risk groups, we developed a 3-tiered histopathologic grading system using reproducible parameters (mitoses, tumor necrosis, and infiltrative border) that are easy to apply in practice. 
 Bone-induced c-kit expression in prostate cancer: a driver of intraosseous tumor growth. Loss of BRCA2 function stimulates prostate cancer (PCa) cell invasion and is associated with more aggressive and metastatic tumors in PCa patients. Concurrently, the receptor tyrosine kinase c-kit is highly expressed in skeletal metastases of PCa patients and induced in PCa cells placed into the bone microenvironment in experimental models. However, the precise requirement of c-kit for intraosseous growth of PCa and its relation to BRCA2 expression remain unexplored. Here, we show that c-kit expression promotes migration and invasion of PCa cells. Alongside, we found that c-kit expression in PCa cells parallels BRCA2 downregulation. Gene rescue experiments with human BRCA2 transgene in c-kit-transfected PCa cells resulted in reduction of c-kit protein expression and migration and invasion, suggesting a functional significance of BRCA2 downregulation by c-kit. The inverse association between c-kit and BRCA2 gene expressions in PCa cells was confirmed using laser capture microdissection in experimental intraosseous tumors and bone metastases of PCa patients. Inhibition of bone-induced c-kit expression in PCa cells transduced with lentiviral short hairpin RNA reduced intraosseous tumor incidence and growth. Overall, our results provide evidence of a novel pathway that links bone-induced c-kit expression in PCa cells to BRCA2 downregulation and supports bone   metastasis  . 
 A subset of colorectal carcinomas express c-KIT protein independently of BRAF and/or KRAS activation. c-KIT is a tyrosine kinase receptor found to be overexpressed in several tumours, namely, GISTs, breast, lung, prostate, ovarian and colorectal carcinomas (CRC). We aimed at determining the frequency of c-KIT expression and mutations in a series of 109 CRC cases (73 primary tumours and 36 lymph node metastases) characterised for KRAS and BRAF mutations. We also aimed at analysing the cellular effects of STI571/Gleevec in CRC-derived cell lines displaying c-KIT expression and KRAS or BRAF mutations. By immunohistochemistry, we found c-KIT overexpression in 15% (11/73) of primary tumours and in 14% (5/36) of   metastasis  ; however, cases showing overexpression did not show c-kit mutations in hotspot regions. The majority (64%) of primary tumours with c-KIT overexpression had mutations at KRAS-BRAF genes. The same was true for 60% of the metastases. We treated CRC cell lines with STI571/Gleevec and verified that it inhibits proliferation and induces apoptosis in all cell lines. In conclusion, overexpression of c-KIT is observed in a subset of primary and CRC metastases in the absence of c-kit mutations. STI571/Gleevec increases apoptosis in CRC cell lines independently of its genetic profile, suggesting that STI571/Gleevec is likely to be an alternative drug for the clinical trials of CRC. 
 Diagnosis and treatment of KIT-mutant metastatic melanoma. A 52-year-old man has unresectable locally recurrent melanoma of the left foot (Fig 1) and pulmonary metastases. Nine months before this presentation, he underwent a wide local excision and sentinel node biopsy for an acral melanoma on his left heel. Pathology disclosed Breslow thickness of 4.8 mm, Clark level IV, and tumor ulceration with a mitotic rate of 37 mitoses/mm(2). Both sentinel nodes in the left groin were positive for melanoma cells, which expressed S100, HMB45, and melan A. At subsequent left inguinal dissection, seven more nodes showed no additional nodal metastases. Within 3 months of his original surgery, the patient developed a local recurrence in the foot, and over the subsequent 6 months, he underwent serial local excisions and topical diphencyprone treatment. A recent staging scan showed at least 20 foci of in-transit disease in the left lower leg and foot, as well as a solitary lung   metastasis   (12 mm). His Eastern Cooperative Oncology Group performance status is 1, with no significant comorbidities. High-resolution melt followed by sequencing of an in-transit   metastasis   showed there is no BRAF exon 15 mutation. However, Sanger sequencing of KIT exons 9, 11, 13, and 17, performed as screening for a clinical trial enrolling patients with metastatic acral and mucosal melanomas, showed an exon 13 K642E mutation. 
 Gastrointestinal stromal tumors with exon 8 c-kit gene mutation might occur at extragastric sites and have   metastasis  -prone nature. Gastrointestinal stromal tumors (GISTs) are the most common mesenchymal tumors of the human gut. Most sporadic GISTs have somatic gain-of-function mutations of the c-kit gene. The mutations are frequently found at exon 11, sometimes at exon 9 and rarely at exon 13 or 17. Recently, exon 8 c-kit gene mutations were reported in very minor proportion of sporadic GISTs. We also found 3 GISTs with exon 8 c-kit gene mutations in approximately 1,000 sporadic GISTs examined. In the present report, we showed the clinicopathological data of those GISTs. One case had a deletion of codon 419 of aspartate, and 2 cases had a substitution of 3 amino acids of codon 417 to codon 419 to tyrosine. The former was the same mutation recently reported in 2 GIST cases, but the latter has not been reported in any GISTs. All three cases occurred at extragastric sites and two of three showed distant   metastasis  . Since the remaining case was regarded as high risk for recurrence, imatinib adjuvant treatment has been done without evidence of   metastasis  . Our results confirmed the idea that exon 8 mutations are minor but actually existing abnormalities in sporadic GISTs, and suggested that such GISTs have a feature of extragastric development and a   metastasis  -prone nature. Since the exon 8 mutations appeared to be really sensitive to imatinib as shown in the present case study, accurate genotyping including exon 8 of the c-kit gene is necessary in GISTs to predict response to imatinib in both the unresectable/metastatic and adjuvant settings. 
 KIT is an independent prognostic marker for pancreatic endocrine tumors: a finding derived from analysis of islet cell differentiation markers. Prediction of the biologic behavior of pancreatic endocrine tumor (PET) without local invasion or   metastasis   is often difficult. The 2004 World Health Organization (WHO) classification uses size, angioinvasion, mitotic activity, and Ki-67 index as prognostic criteria. Recently, cytokeratin 19 (CK19) was shown to be another prognostic marker, but the mechanism by which CK19 predicts prognosis is unknown. As CK19 is the first cytokeratin expressed in all epithelial cells in fetal pancreas, we sought to test expression of other markers of islet cell differentiation including KIT, Pdx-1, Pax4, and Pax6 in PET and correlation of these markers with clinical behavior. Clinical information and histology was reviewed in 97 PETs. All tumors were classified according to WHO criteria and a tumor, node, and metastases stage system. Immunohistochemistry was performed using antibodies to Ki-67, KIT, CK19, Pdx-1, Pax4, and Pax6. Associations of clinicopathologic and immunohistochemical features with prognosis were evaluated using Cox proportional hazards regression models. WHO and tumor, node, and metastases classifications, mitotic counts and Ki-67 labeling, infiltrative border, necrosis, perineural invasion, extrapancreatic extension, tumor size, and positive CK19 and KIT expression were significantly associated with death from disease in a univariate setting. In multivariate analysis, only WHO criteria and KIT expression were shown to be independent. An immunohistochemical classification system was derived from a combination of KIT and CK19 expression: low risk (KIT-/CK19-), intermediate risk (KIT-/CK19+), and high risk (KIT+/CK19+). Survival, metastases, and recurrence of PET were significantly different among the 3 groups. These results indicate that KIT is a new and independent prognostic marker for PETs. The classification system derived from KIT and CK19 was able to predict clinical behavior of PET. 
 Postsurgical radiation therapy for gastric carcinosarcoma with c-kit expression: a case report. Gastric carcinosarcomas are rare morphologically biphasic tumors, consisting of carcinoma and sarcoma components, with a poor clinical course. Here we report the case of a 70-year-old man with advanced Borrmann type III carcinosarcoma arising from the upper body of the stomach with extensive lymph node   metastasis   who underwent a total, but palliative, gastrectomy. Histology showed the tumor consisted of a biphasic structure of tubular adenocarcinoma and spindle cell sarcoma. Immunohistochemistry revealed sarcoma cells expressing c-kit (CD117) and CD34, which are criteria for gastrointestinal stromal tumors. Nine months after the surgical operation, tumor metastases had extended to the hepatohilar, retroperitoneal and mediastinal lymph nodes. Radiation therapy of 50 Gy markedly decreased the size of each of these nodes and reduced the risk of respiratory complications and jaundice. However, the patient died of respiratory failure due to bronchopneumonia with multiple lung metastases 22 mo after resection. Autopsy revealed severe necrosis in most of the lymph nodes with tumor metastases. Radiation therapy combined with gastrectomy should be considered to improve survival in patients with gastric carcinosarcomas that express c-kit. 
 Reappraisal of KIT mutation in adenoid cystic carcinomas of the salivary gland. While overexpression of KIT protein has been well documented in adenoid cystic carcinomas (ACCs), mutation of KIT gene has been a controversial issue. We wanted to evaluate clinical value of the KIT mutation and protein expression in ACC.We analyzed 33 cases of ACC. Gene mutations in KIT exons 9, 11, 13, and 17 were analyzed using paraffin-embedded tissue, and two different sets of primers with direct sequencing after polymerase chain reaction (PCR) for exon 9, 11, 13, and 17, and cloning of PCR products for exon 11. KIT protein expression was assessed by immunohistochemistry. The correlation between clinicopathological findings and these biomarkers was analyzed.No KIT mutation was observed in all of the 33 cases. With one primer set, KIT mutation was found in nine of 33 cases (27.3%). However, these mutations were not reproducible in the experiment using another primer set. KIT protein overexpression was detected in 22 of 33 patients (66.7%). KIT protein expression was not statistically correlated with either clinicopathological factors or survival. Patients with   metastasis   showed a tendency of longer progression-free survival (P = 0.052) and overall survival (P = 0.080) when the tumor overexpressed KIT protein.This study supports that mutational study using paraffin-embedded tissue should be interpreted with great caution. KIT gene mutation is very rare in ACC, and gene mutation is not the cause of protein overexpression. KIT protein expression may have a potential value for better prognostic factor in patients with   metastasis  . 
 Expression of CK19 and KIT in resectable pancreatic neuroendocrine tumors. The objectives of this study were to validate the immunohistochemical expression patterns of CK19 and KIT in primary pancreatic neuroendocrine tumors (pNETs) and to verify the potential biomarkers that can be used to predict the clinical behaviors and postoperative outcomes. The immunohistochemical expressions of CK19 and KIT were determined in normal pancreatic islets and resectable pNETs. Associations of the immunohistochemical features with the clinicopathologic features and prognosis were evaluated. All 20 samples from the normal control group were negative for both KIT and CK19 in normal pancreatic islets. Positive rates for KIT and CK19 in pNETs were 49.5 % (45/91) and 70.0 % (70/100), respectively. The percentages of G1, G2, and G3 tumors were 54.9, 42.9, and 2.2 %, respectively. Ki-67 index was significantly higher in the KIT-positive subgroup than in the KIT-negative subgroup (p < 0.05); however, no statistically significant difference of the Ki-67 index was found between the CK19-positive and the CK19-negative subgroups (p = 0.656). The positive CK19 expression was significantly associated with non-functioning tumors, regional lymph nodes metastases, and advanced tumor node   metastasis   (TNM) stage (p < 0.05). Meanwhile, the positive KIT expression was significantly associated with advanced TNM grade (p < 0.05). In univariate analysis, the overall survival in patients with positive CK19 expression was significantly lower than that in patients with CK19-negative expression (p < 0.05). Also, patients with negative KIT expression showed a tendency of longer survival duration compared with those with positive KIT expression (p = 0.188). The high-risk subgroup (2.1 ± 2.9) might have a higher Ki-67 index than the low-risk subgroup (1.0 ± 1.7, p = 0.208). There was a significant difference in functioning status among the three risk levels (p < 0.05). Pairwise comparison prompted that patients at high risk were more prone to have regional lymph nodes metastases, distant metastases, and/or recurrences. In conclusion, the expressions of CK19 and KIT are associated with aggressive clinical behaviors in patients with resectable pNETs. CK19 and KIT may play a role in tumor progression and metastases. 
 CD117 immunoreactivity in atypical fibroxanthoma. Atypical fibroxanthoma (AFX) is a spindle cell neoplasm of the skin seen typically on sun-damaged skin of the elderly. Though described as a benign entity, local recurrence and distant   metastasis   have been reported. This study aims to investigate the potential pathogenic role of CD117, the c-kit receptor in AFX. CD117 was detected in 15 of the 16 cases (94%). The percentage of positive cells for CD117 expression among all tumors was approximately 30%. CD117 proved to be a very sensitive marker of AFX. This antibody may be a useful diagnostic adjunct in AFX. 
 CD117 expression in operable oesophageal squamous cell carcinomas predicts worse clinical outcome. To investigate the aberrant expression of CD117 in oesophageal squamous cell carcinoma (SCC) and its prognostic significance.Immunohistochemical staining for CD117 was performed on tissue microarray and routine tissue sections from 157 oesophageal SCC patients and 10 normal oesophageal epithelia adjacent to tumour. The positive rate of CD117 expression was 29.9% in oesophageal SCC tissues, whereas no CD117 expression was detected in the 10 normal oesophageal epithelia. CD117 expression was significantly associated with T stage (P < 0.001), distant   metastasis   (P = 0.015), lymph node   metastasis   (P = 0.019), and clinical stage (P = 0.021). Progression-free survival in the patients with CD117-positive tumours was shorter than that in the patients with CD117-negative tumours (P = 0.010). In univariate analyses, CD117 expression was the most significant factor for overall survival of oesophageal SCC patients (P < 0.001), followed by lymph node   metastasis   (P = 0.001), T stage (P = 0.002), clinical stage (P = 0.006), distant   metastasis   (P = 0.020), and histological grade (P = 0.027). Multivariate analyses verified that CD117 expression was an independent prognostic marker for oesophageal SCC patients (P = 0.002). In addition, CD117 expression predicted poorer survival in patients without distant metastases.CD117 expression in operable oesophageal SCC may be a valuable prognostic marker, and detection of its expression in clinical samples may be useful in defining a subclass of oesophageal SCCs with extremely poor clinical outcome, which may require a specially targeted treatment modality. 
 C-Kit SCF receptor (CD117) expression and KIT gene mutation in conjunctival pigmented lesions. To investigate the presence of KIT gene mutations and immunoreactivity in 85 conjunctival melanocytic tumours and to clarify the role of KIT as a potential therapeutic target in this group of patients.Eighty-five conjunctival pigmented tumours [27 melanomas, 12 primary acquired melanosis (PAMs) and 46 nevi] were immunostained for KIT. Intensity and pattern of expression were evaluated. Molecular analysis to identify KIT mutations was performed in 15 selected cases (tumour-rich areas >50%). KIT immunostaining score and pattern were statistically related to patients' age, sex, diagnostic category, presence of relapse, disease-free survival, presence of metastases,   metastasis  -free survival, limbal versus nonlimbal tumour location and thickness of melanomas.KIT stains were documented in 48% of melanomas, 50% of PAMs and 24% of nevi. The mean score of KIT staining in the melanomas/PAMs group was significantly different from nevi (p = 0.0076). No statistically significant differences were detected between either c-kit immunostaining score or pattern and each of the other clinico-pathologic parameters considered. No KIT gene mutations were detected in melanomas and nevi. A silent mutation/polymorphism in KIT exon 13 was found in one PAM.Despite the high level of KIT immunostains in PAMs and melanomas, this parameter seems not to be a good predictor of the presence of molecular mutations. KIT-activating mutations should be considered an uncommon event in this tumour. 
 Malignant Melanoma of Vulva and Vagina: A Histomorphological Review and Mutation Analysis--A Single-Center Study. The aim of this work was to determine molecular characteristics and specifically, the frequency of BRAF, C-KIT, and NRAS mutations in vulvar and vaginal melanomas.A retrospective review of all cases of vulvar and vaginal melanoma between 2002 and 2013 was performed. We reviewed the clinical and histological characteristics of all cases and performed genotyping studies on cases that had tissue available for the study, using next-generation sequencing.We identified 33 vulvar and 11 vaginal melanomas in women with mean ages 58 and 61 years, respectively. Next-generation sequencing analysis on 20 cases (15 vulvar and 5 vaginal) identified a BRAF mutation in 7.6%, C-KIT mutation in 27.6%, NRAS mutation in 27.6%, and TP53 mutation in 7.6% of the vulvar cases. We detected only a single TP53 mutation in the vaginal cases. We did not identify any statistically significant relationship between the mutation status and patients' outcome, depth of invasion, ulceration, stage at presentation, or lymph node   metastasis  .BRAF mutations are infrequent, whereas C-KIT and NRAS mutations are seen with higher frequency in vulvar melanomas than melanomas of other sites. These mutations can be considered as potential therapeutic targets in patients harboring them. Further studies are necessary to increase our understanding of mutational events occurring in melanoma of the lower female genital tract and their relationship with clinical parameters/outcome. 
 Hereditary gastrointestinal stromal tumors sharing the KIT Exon 17 germline mutation p.Asp820Tyr develop through different cytogenetic progression pathways. Hereditary gastrointestinal stromal tumor (GIST) syndrome is a rare autosomal dominant genetic disorder originated by germline mutations in the KIT or PDGFRA genes. We report the third family with hereditary predisposition to GIST due to the KIT Exon 17 germline mutation p.Asp820Tyr and characterize the cytogenetic progression pathways followed by different GIST sharing the same primary genetic event, using a combination of chromosome banding, comparative genomic hybridization (CGH), and fluorescence in situ hybridization (FISH) analyses. The missense mutation p.Asp820Tyr was detected in the proband's rectal and gastric GIST, as well as in his aunt's GIST epiplon   metastasis  . The mutation p.Asp820Tyr was subsequently also found in the proband's peripheral blood DNA, as well as in that of 4 of 10 relatives thus far analyzed. CGH analysis revealed loss of 14q and 15q in the proband's gastric lesion, whereas FISH analysis of the proband's rectal GIST did not detect loss of 14q and 15q, but instead loss of 4q and 22q and gain of 20q, indicating that the two tumors were independent GIST. Chromosome banding and CGH analyses of the aunt's GIST epiplon   metastasis   revealed multiple cytogenetic alterations, including 1p loss, but none in common with the two proband's GIST. We conclude that, although the patients share the same KIT Exon 17 germline mutation, the multiple GIST analyzed followed different pathogenetic progression pathways, each of which did not significantly differ from what has been described in sporadic GIST. 
 Recurrent inflammatory myofibroblastic tumors harboring PIK3CA and KIT mutations. Inflammatory myofibroblastic tumour (IMT) is a relatively rare soft tissue malignancy. It exhibits locally aggressive behavior with a tendency for local recurrence and rare   metastasis  , and rare recurrent IMTs may show histological progression. The genetic hallmark of IMT is ALK rearrangement from chromosome arm 2p, but gene mutations involved in IMT remain poorly understood. The aim of the present study was to perform a pairwise comparison of the gene mutations occurring in primary and recurrent IMT from the same patient. We conducted a high-throughput analysis of 238 known mutations of 19 oncogenes in pairwise comparison primary and recurrent samples from 2 patients of IMT using Sequenom MassARRAY technology. Our results revealed 2 mutations in 2 recurrent lesion samples, including one in exon 11 of the KIT gene, resulting in a T-C substitution at position 1727 (L576P), the recurrent sample underwent histologic progression with "pleomorphic undifferentiated sarcoma-like" transformation; the other mutation was in exon 19 of the phosphatidylinositol-4,5-bisphosphate 3-kinase, catalytic subunit alpha (PIK3CA) gene, resulting in a G-A substitution at position 1624 (E542K). Moreover, no any mutation was found in the primary lesion samples from 2 patients. Our findings suggest that variable genome changes might be present in IMT, especially during the progression from a primary tumour to recurrence. To the best of our knowledge, no such longitudinal study of IMT has been undertaken previously. 
 [Expression and mutation of c-kit gene in gastrointestinal stromal tumor]. To investigate the effect of the expression and mutation of c-kit gene and its relationship with clinical pathology and prognosis of gastrointestinal stromal tumor (GIST).Immunohistochemical and PCR-SSCP techniques were used to detect c-kit protein expression and c-kit gene exon 11 mutation in 82 patients with GIST.The positive c-kit protein expression and c-kit gene mutation rates were 97.6% (80/82) and 41.5% (34/82). Correlating the results of these two methods and clinicopathological factors, the c-kit expression and c-kit gene mutation rates were 95.0% (19/20) and 0 in benign GIST, and were 98.4% (61/62), 54.8% (34/62) in malignant GIST. Mutation positive GIST showed higher frequency of adjacent tissue invasion,   metastasis   and recurrence as compared with mutation negative ones.c-kit protein is an important diagnostic marker of gastrointestinal stromal tumor. c-kit gene mutation may play a significant role in the pathogenesis of GIST and also may be a prognostic marker. 
 Prognostic value of KIT mutation in gastrointestinal stromal tumors. To examine the prevalence and prognostic significance of C-kit gene mutation and analysis the correlation of C-kit gene mutation and the clinicalpathologic parameters of GISTs.Eighty-two GISTs were studied for the mutation of C-kit gene by PCR-SSCP, DNA sequence. Statistical comparison were used to analysis the correlation of C-kit gene mutation and clinicalpathology, clinical behavior, recurrence.(1) Mutation-positive and mutation-negative GISTs were 34 and 48,respectively; (2) Among these patients with C-kit mutation remained a significantly poor prognosis associated with 59% 3-year survival compared to those whose tumors did not; (3) Tumor size, PCNA index, mitotic cell number, presence of necrosis, microscopic invasion to adjacent tissues, recurrence and distant   metastasis   among mutation-positive and mutation-negative GISTs were significantly different.C-kit mutation is a undoubtedly pivotal event in GIST and may be associated with poor prognosis. Evaluation of C-kit gene mutation may have both prognosis and therapeutic significances. 
 A clinicopathological review of 33 patients with vulvar melanoma identifies c-KIT as a prognostic marker. Vulvar melanoma is the second most common vulvar cancer. Patients with vulvar melanoma usually present with the disease at a late stage and have a poor prognosis. The prognostic predictors reported in the literature are not unequivocal and the role of lichen sclerosus and c-KIT mutations in the aetiology of vulvar melanoma is unclear. Breslow staging currently seems to be the most adequate predictor of prognosis. We thus performed a clinicopathological and literature review to identify suitable predictors of prognosis and survival and investigated the expression of c-KIT (by immunohistochemistry) in patients with vulvar melanoma (n=33) from the Gynaecological Cancer Centres of the Royal Hospital for Women (Sydney, Australia) and John Hunter Hospital (Newcastle, Australia). Our series of 33 patients fitted the expected clinical profile of older women: delayed presentation, high stage, limited response to treatment and poor prognosis. We identified 3 patients (9.1%) with lichen sclerosus associated with melanoma in situ, although no lichen sclerosus was found in the areas of invasive melanoma. No patient had vulvar nevi. We identified a) Breslow's depth, b) an absence of any of the pathological risk factors, such as satellitosis, in-transit   metastasis  , lymphovascular space invasion (LVSI) and dermal mitosis, c) removal of inguino-femoral lymph nodes, d) lateral margin of >1 cm, and e) c-KIT expression as valuable prognostic predictors for disease-free survival. We conclude that c-KIT expression is, apart from Breslow's depth, another valuable predictor of prognosis and survival. Lichen sclerosus may be associated with vulvar melanoma. 
 Molecular portraits of heterogeneity related to cancer stem cells in human ovarian cancer. The aim of this study was to investigate molecular portraits of heterogeneity related to cancer stem cells (CSCs) in human ovarian cancer and to access the value in diagnosis and treatment.Sixty specimens were collected in both cytoreductive and re-cytoreductive surgeries of 20 serous papillary ovarian adenocarcinoma cases. Expression density and distribution of 3 CSC markers (CD44, CD133, and CD117) and 3 stemness proteins (Bmi1, Nestin, and Oct3/4) were analyzed by immunohistochemical staining. Pairwise comparisons were performed among their expression in primary,   metastasis  , and relapsing tumors.Some molecules presented different localization in 1 tissue, like CD133 and CD117, and all but Oct3/4 expressed differentially in different specimens of 1 case. Compared to primary or metastatic cancers, recurrent cancers show higher expression of CD133, CD117, and Bmi1, as well as higher histological grades.Our study indicated that there exist extratumoral and intratumoral heterogeneity in ovarian epithelial cancers related to CSCs. And this is worth further studying. 
 Identification of c-kit gene mutations in primary adenoid cystic carcinoma of the salivary gland. The CD117 (KIT) protein is overexpressed in many human neoplasms including adenoid cystic carcinoma of salivary glands. To evaluate the function of c-kit-activating mutations in adenoid cystic carcinoma of the salivary gland, we studied 14 cases (13 primary, 1 cervical lymph node   metastasis  ) from our institution. KIT protein expression was evaluated by immunohistochemistry using formalin-fixed paraffin-embedded tissue. Mutational analyses of c-kit extracellular (exon 9), juxtamembrane (exon 11) and tyrosine kinase domains (exons 13 and 17) were performed by polymerase chain reaction, clonal selection and DNA sequencing. All 14 cases demonstrated strong KIT expression by immunohistochemistry. Molecular analysis was successful in 8 of 14 cases, and c-kit missense point mutations were detected in seven of eight cases (88%) including seven in exon 11, two in exon 9, two in exon 13 and two in exon 17. Eight silent point mutations were detected in five cases. Two cases contained missense mutations in more than one exon. Different mutations were found in the primary tumor and the cervical lymph node   metastasis   of one patient. Point mutations in domains similar to those described in gastrointestinal stromal tumors were detected, including Pro551Leu and Lys558Glu (5' end of exon 11), Leu576Phe (3' end of exon 11), Val643Ala (exon 13) and Asn822Ser (exon 17). Additional novel point mutations in exons 9, 11, 13 and 17 were also identified. This study is the first to report c-kit gene mutations in primary adenoid cystic carcinoma of the salivary gland. Identification of such potential gain-of-function mutations in exon 11, and less frequently in exons 9, 13 and 17, suggests that KIT may be involved in the pathogenesis of adenoid cystic carcinoma of salivary glands. Our study raises a prospect of correlation of c-kit mutation and a potential treatment of adenoid cystic carcinoma with tyrosine kinase inhibitor (imatinib). 
 Coexpression of fragile histidine triad and c-kit is relevant for prediction of survival in patients with small cell lung cancer. In a retrospective analysis of 195 patients with small cell lung cancer (SCLC), we examined the prognostic value of a coexpression of fragile histidine triad (FHIT) protein and c-kit on patient's survival.As assessed by immunohistochemistry using formalin-fixed, paraffin-embedded tissue sections, tumors of 195 patients with SCLC were evaluated for FHIT and c-kit coexpression.Coexpression of FHIT and c-kit was observed in 53.3%; a positive expression of either FHIT or c-kit was found in 40.5%. Complete lack of FHIT and c-kit (6.2%) was associated with a significantly shorter survival time for the patients with a mean of 122 +/- 45 days compared with 468 +/- 89 days for patients with lung cancer coexpressing FHIT and c-kit (P = 0.0011). The proportion of FHIT- and c-kit-positive cells within a tumor was also related to survival time. Patients with tumors with a proportion between 0% to 25% of FHIT- and c-kit-positive cells had the worst survival of 157 +/- 34 days compared with 496 +/- 95 days for patients showing >25% FHIT- and c-kit-positive cells (P = 0.0002). Further, variables associated with shorter survival times were low performance status, elevated lactate dehydrogenase level, and advanced tumor stage according to tumor-node-  metastasis   classification. Multivariate analysis using Cox regression model, including 11 variables, confirmed the prognostic significance of a combined expression of FHIT and c-kit next to tumor stage, performance status, and lactate dehydrogenase level.Differential FHIT and c-kit expression was of prognostic relevance for survival in patients with SCLC and therefore provide useful variables for therapeutic decisions. 
 c-kit expression in primary and metastatic merkel cell carcinoma. Merkel cell carcinoma (MCC) is an aggressive neuroendocrine tumor of the skin that is associated with a high incidence of recurrence and   metastasis  . The therapeutic arsenal for this malignancy is limited and once it spreads, there is no effective treatment. c-kit expression has been demonstrated previously in primary MCCs thus raising the possibility of treating MCCs with imatinib mesylate, the tyrosine kinase inhibitor that has shown promise in the management of c-kit expressing tumors. In this study we examine 25 additional primary MCCs and also 6 of their lymph node metastases. Formalin-fixed, paraffin-embedded tissues were stained immunohistochemically with an antibody directed against the KIT receptor. Percentage and intensity of staining were analyzed semiquantitatively using a three-tiered system. Twenty-one of the 25 (84%) primary tumors stained positively for KIT, of which 14 (67%) showed widespread positivity. Five of the 6 lymph nodes (83%) were similarly positive. High mitotic rate and vascular invasion in the primary tumors tended to be associated with prominent staining in the lymph node metastases. No association was found between c-kit expression and outcome. We confirm that the majority of primary MCCs express c-kit and further find that metastases are positive for the KIT receptor as well. Thus, c-kit expression may be an early event in the transformation of MCC, but not a marker for tumor progression. 
 Long non-coding RNA HOTAIR modulates c-KIT expression through sponging miR-193a in acute myeloid leukemia. HOTAIR is significantly overexpressed in various cancers and facilitates tumor invasion and   metastasis  . However, whether HOTAIR plays oncogenic roles in acute myeloid leukemia (AML) is still unknown. Here, we report that HOTAIR expression was obviously increased in leukemic cell lines and primary AML blasts. Clinically, AML patients with higher HOTAIR predicted worse clinical outcome compared with those with lower HOTAIR. Importantly, HOTAIR knockdown by small hairpin RNA inhibited cell growth, induced apoptosis, and decreased number of colony formation. Finally, HOTAIR modulated c-KIT expression by competitively binding miR-193a. Collectively, our data suggest that HOTAIR plays an important oncogenic role in AML and might serve as a marker for AML prognosis and a potential target for therapeutic intervention. 
 KIT exon 10 variant (c.1621 A > C) single nucleotide polymorphism as predictor of GIST patient outcome. Tumor genotype plays a crucial role in clinical management of GIST. Whether genetic polymorphism of KIT may influence GIST patient outcome is unclear.We investigated the biological and clinical significance of the presence of KIT exon 10 variant (c.1621 A > C), KIT (L541), in a transfected cell line (3 T3 L541) and in two retrospectively collected series of 109 GIST patients in total. The control group consisted of 60 healthy donors collected at the French department of blood transfusion.In the 3 T3 L541 cell line, KIT(L541) protein exhibited a spontaneous phosphorylation status comparable to that of wild-type KIT but displayed a phosphorylation pattern of AKT and ERK1/2 that was found similar to that of the classical mutated forms of the KIT receptor. Of 109 patients enrolled in this retrospective translational research study, 24 (22%) harboured KIT (L541), similarly to the control group of healthy donors (n = 10 of 60, 17%). A higher prevalence of the variant KIT (L541) was observed in patients with metastatic status at diagnosis (KIT (L541) correlated nine of 22 versus 15 of 87, p = 0.02). In addition, patients with KIT (L541) and localized GIST had a higher rate of relapse at 5 years and lower relapse free survival at 5 years in univariate, as well as in multivariate analysis. Response rate and duration of response to imatinib was similar in KIT (L541) and KIT (M541) patients.KIT (L541) genotype is associated with a higher risk of   metastasis   at diagnosis and a higher risk of relapse in GIST patients. 
 BRAF, KIT and NRAS mutations and expression of c-KIT, phosphorylated extracellular signal-regulated kinase and phosphorylated AKT in Japanese melanoma patients. To clarify the status of gene mutation and activation of growth signal in melanoma of Japanese patients in vivo, we analyzed the mutation of BRAF exon 15, NRAS exon 2, and KIT exons 9, 11, 13, 17 and 18 in melanoma cells obtained by laser capture microdissection, and performed direct sequencing in 20 cases of acral lentiginous melanoma (ALM) and 17 cases of superficial spreading melanoma (SSM). In the study of the mutation of BRAF, pyrosequencing was also done. To examine the cell proliferation signaling, immunohistochemistry for phosphorylated extracellular signal-regulated kinase (pERK), phosphorylated AKT (phosphorylated AKT) and c-KIT was done. The mutation of BRAF p.V600E was detected in 13 cases of ALM (65.0%) and 12 cases of SSM (70.6%). No NRAS mutation was found in all cases. The mutation in exons 9, 11, and 18 of KIT was detected in nine cases. The mutation of BRAF and KIT showed no correlation with clinical stage, lymph node   metastasis  , tumor thickness, ulceration and histology. pERK and pAKT was observed in small population of melanoma cells and there was no correlation with gene mutation. Our results indicate that the mutations of BRAF and KIT exist in Japanese melanoma patients, however, the cell growth signaling may be regulated by not only these mutated genes, but by other unknown regulatory factors, which may affect the prognosis of melanoma. 
 Expression of survivin, CD117, and C-erbB-2 in neuroendocrine carcinoma of the uterine cervix. To evaluate the expression and prognostic significance of survivin, CD117, and C-erbB-2 in neuroendocrine carcinoma of the uterine cervix.Immunohistochemical stains of survivin, CD117, and C-erbB-2 were evaluated in 100 cases of cervical neuroendocrine carcinoma. The findings were correlated with clinicopathologic variables and disease-free survival.Expressions of survivin, CD117, and C-erbB-2 were detected in 27.0%, 12.0%, and 2.0% of the cases, respectively. Survivin-positive patients had a significantly younger mean age than the survivin-negative group (P=0.033). In early-stage cases, tumor recurrence was significantly associated with lymph node   metastasis   (P=0.005), depth of invasion (P=0.028), and the presence of lymphovascular space invasion (P=0.031) but not with the expression of survivin or CD117. Subgroup analysis in early-stage cases without lymph node   metastasis   (n=32) showed that only survivin expression had a significant association with decreased disease-free survival (P=0.041).Survivin expression may be a prognostic indicator for survival in early-stage neuroendocrine carcinoma of the uterine cervix without lymph node   metastasis  . Adjuvant survivin-targeted therapy may have potential benefit in patients with this tumor. 
 [Prognostic significance of clinicopathologic parameters in gastrointestinal stromal tumor: a study of 156 cases]. To evaluate the prognostic significance of various clinicopathologic parameters in gastrointestinal stromal tumor (GIST), and to study the frequency of c-kit exon 11 mutations in this tumor.One hundred and fifty-six cases of gastric or small intestinal GIST were retrieved from the archival files of the Department of Pathology, Chinese PLA General Hospital. The clinical features, site of occurrence, tumor diameter, mitotic index, coagulative tumor necrosis, and risk grade were studied and analyzed statistically. Tumor DNA was extracted and c-kit exon 11 was amplified. Upon detection by denaturing high-performance liquid chromatography, the amplified exon 11 was sequenced.For the 83 cases of gastric GIST studied, the mean age of patients was 55.4 years. Follow-up information was available in 62 cases, with 17 cases having local recurrence or distant   metastasis  . The 5-year survival rate was 66.5% +/- 17.1%. For the 73 cases of small intestinal GIST studied, the mean age of patients was 50.6 years. Follow-up information was available in 43 cases, with 22 cases having local recurrence or distant   metastasis  . The 5-year survival rate was 61.8% +/- 18.3%. In general, for gastric GIST, age younger than 50 years (P = 0.046), advanced clinical stage (P = 0.0001), large tumor size (P = 0.0001), high mitotic index (P = 0.0001), presence of coagulative tumor necrosis (P = 0.0001), and high risk grade (P = 0.004) were associated with lower survival rate. COX hazard proportional model revealed that advanced clinical stage (P = 0.001), large tumor size (P = 0.001), high mitotic index (P = 0.002) and high risk grade (P = 0.018) indicated worse prognosi. For small intestinal GIST, advanced clinical stage (P = 0.010) and presence of coagulative tumor necrosis (P = 0.036) were associated with lower survival rate. Advanced clinical stage was an independent prognostic factor. A total of 25 cases harbored c-kit mutations. The frequency of c-kit mutations was 32% and 22.5% for gastric and small intestinal GIST respectively. For gastric GIST, c-kit mutations occurred mainly in patients older than 50 years. In contrast, c-kit mutations in small intestinal GIST occurred in the age group of 40 to 49 years.For gastric GIST, advanced clinical stage, tumor diameter, mitotic index and risk grade are the main prognostic indicators. For small intestinal GIST, advanced clinical stage and presence of coagulative tumor necrosis indicate poor prognosis. In general, small intestinal GIST is more frequently associated with   metastasis   and tumor relapse than gastric GIST. The occurrence of c-kit mutations also correlates with age of patients. 
 Modulation of c-kit expression in pancreatic adenocarcinoma: a novel stem cell marker responsible for the progression of the disease. Pancreatic ductal adenocarcinoma (PDAC) is one of the most lethal cancers because of late symptoms and resistance to chemotherapy and radiation therapy. We have investigated the appearance of c-kit, a stem cell marker, in both normal adult pancreatic tissue and in cancerous tissue. Apart from some very pale staining of islets of Langerhans, normal pancreas was devoid of staining with antibodies to c-kit. In contrast, in cancerous tissue that still preserves the overall integrity of the pancreatic tissue, there was a clear labeling in islets of Langerhans, which seemed to be co-localized with insulin containing β cells. In other cases, where the pancreatic tissue was completely deteriorated, intensive labeling was clearly evident in remnants of both the exocrine and the endocrine tissues. The duct cells of the adenocarcinoma were moderately but clearly labeled with antibodies to c-kit. In contrast, in   metastasi  s of PDAC, very intensive labeling of c-kit was evident. The location of KRAS, which is strongly associated with PDAC, was also analyzed at the initial stages of the disease, when islets of Langerhans still preserve their integrity to a large extent. KRAS was found exclusively in islets of Langerhans and overlapped in its location with insulin and c-kit expressing cells. It is suggested that the modulation of the expression of c-kit, visualized by antibodies to the oncogene molecule, may play an important role in the formation and progression of PDAC. The absence of c-kit in normal pancreas and its appearance in PDAC is probably due to a mutational event, which probably allows conversion of the β cells into cancer stem cells (CSC). Co-expression of both c-kit and KRAS, typical markers for CSC with overlapping with insulin in islets of Langerhans, strongly support the notion that β-cells play a central role in the development of PDAC. The use of specific drugs that can attenuate the kinase activity of c-kit or target KRAS expressing cancer cells should be tested in order to attenuate the progression of this lethal disease. 
 C-kit induces epithelial-mesenchymal transition and contributes to salivary adenoid cystic cancer progression. Epithelial-mesenchymal transition (EMT) is associated with salivary adenoid cystic cancer (ACC) progression and   metastasis  . Here, we report that ectopic overexpression of c-kit in ACC cell lines is sufficient for acquisition of mesenchymal traits, enhanced cell invasion, along with stem cell properties defined by the presence of a CD133+/CD44+ cell subpopulation. c-kit positively regulated expression of known EMT inducers, also activating TGF-β to contribute to EMT. c-kit itself was induced by TGF-β in ACC cell lines and required for TGF-β-induced EMT. Xenograft experiments showed that c-kit cooperated with oncogenic Ras to promote tumorigenesis in vivo. Finally, in human specimens of ACC, we found that c-kit was abnormally overexpressed and correlated with the prognosis of ACC. Our findings define an important function for c-kit in ACC progression by orchestrating EMT, and they implicate this gene product as a marker of poor prognosis in this disease. 
 Prognostic significance of c-KIT in vulvar cancer: bringing this molecular marker from bench to bedside. Vulvar carcinomas are rare tumors, and there is limited data regarding molecular alterations. To our knowledge there are no published studies on c-KIT and squamous cell carcinomas of the vulva (VSCC). Although there are a significant number of other tumor types which express c-KIT, there remains controversy as to its relationship to patient outcome. Thus, we wished to investigate such controversial findings to determine the prognostic importance of c-KIT by evaluating its protein and mRNA expression in VSCCs, correlating these findings with clinicopathological features and Human Papillomavirus (HPV) infection.c-KIT expression was scored by immunohistochemistry (IHC) as positive or negative in 139 formalin-fixed paraffin-embedded (FFPE) cases of vulvar carcinomas arrayed in a tissue microarray (TMA) using the DAKO A4502 rabbit polyclonal c-KIT antibody (diluted 1:100). c-KIT mRNA was evaluated by qRT-PCR in 34 frozen samples from AC Camargo Hospital Biobank (17 tumoral and 17 non-tumoral samples) using TaqMan probes-Applied Biosystems [Hs00174029_m1]. HPV genotyping was assessed in 103 samples using Linear Array® HPV Genotyping Test kit (Roche Molecular Diagnostics, Basel, Switzerland). All results obtained were correlated with clinical and pathological data of the patients.c-KIT protein was positive by immunohistochemistry in 70.5% of the cases and this was associated with a higher global survival (p = 0.007), a higher recurrence-free survival (p < 0.0001), an absence of associated lesions (p = 0.001), lymph node   metastasi  s (p = 0.0053), and HPV infection (p = 0.034). Furthermore, c-KIT mRNA quantitation revealed higher levels of transcripts in normal samples compared to tumor samples (p = 0,0009).Our findings indicate that those vulvar tumors staining positively for c-KIT present better prognosis. Thus, positivity of c-KIT as evaluated by IHC may be a good predictor for use of more conservative surgery techniques and lymph node dissection in vulvar cancer. So part of the essence of our study is to see the possibility of translating our current results from the bench to the bedside. This will help provide patients a more appropriate, less mutilating treatment, in order to keep the maximum physical and psychic quality as possible to these women. 
 [Effect of c-kit mutation on the prognosis of gastrointestinal stromal tumors: a meta-analysis]. To investigate the effect of c-kit mutation on the prognosis of gastrointestinal stromal tumors.A search of studies in PubMed and MedLine (from 1999 to 2008) was performed to assess the effect of c-kit mutation on the prognosis of gastrointestinal stromal tumors. The articles were retrieved with the entries of "gastrointestinal stromal tumors", "imatinib", "c-kit" and "mutation". A meta-analysis was performed to assess the data included.A total of 15 articles were collected in this analysis. No significant differences was found in incidence of mitoses (> 5/50 HPF) between the patients with wild type c-kit (wild type group) and the ones with mutated c-kit (mutation group) (P = 0.710); tumor recurrence and   metastasis   rate after surgery was significant higher in the mutation group than that in wild type group (P = 0.010); as for imatinib response with different c-kit mutation types, the results showed the incidence of clinical response (complete response + partial response) was significantly higher in mutation group than that in wild type group (P = 0.009), but the imatinib resistance rate was lower in mutation group (P = 0.000); three studies provided data for imatinib resistance with c-kit second mutations, the results showed the second mutations mainly focus on exon 13, 14, 17.C-kit mutation is related closely with the incidence of recurrence and   metastasis   in GIST after surgery. The mutations of c-kit influences the therapeutic effects of imatinib. 
 C-kit Mutations in Endometrial Cancer: Correlation with Tumor Histologic Type. Endometrial cancer is the fourth most common cancer among women in developed countries. Affected patients may benefit from systemic chemotherapy, alone or in combination with targeted therapies if the disease is clinically diagnosed prior to expansion and   metastasis   to other organs.The aim of this study was to evaluate the prognostic role of c-kit mutations and comparision with tumor type and grade in human uterine endometrial carcinomas.Seventy five patients with endometrial carcinoma and seventy five normal controls were studied for possible mutations in exon 17 of the c-kit gene using single strand conformational polymorphisms and sequencing.c-kit mutation in exon 17 appeared to be significantly different between endometrial carcinoma and normal endometrium. The pattern and frequency of the mutations was also shown to be different between tumors from different stages. 
 c-Kit is suppressed in human colon cancer tissue and contributes to L1-mediated   metastasis  . The transmembrane neural cell adhesion receptor L1 is a Wnt/β-catenin target gene expressed in many tumor types. In human colorectal cancer, L1 localizes preferentially to the invasive front of tumors and when overexpressed in colorectal cancer cells, it facilitates their   metastasi  s to the liver. In this study, we investigated genes that are regulated in human colorectal cancer and by the L1-NF-κB pathway that has been implicated in live  r metastas  is. c-Kit was the most highly suppressed gene in both colorectal cancer tissue and the L1-NF-κB pathway. c-Kit suppression that resulted from L1-mediated signaling relied upon NF-κB, which directly inhibited the transcription of SP1, a major activator of the c-Kit gene promoter. Reconstituting c-Kit expression in L1-transfected cells blocked the biological effects conferred by L1 overexpression in driving motility and li  ver metast  asis. We found that c-Kit expression in colorectal cancer cells is associated with a more pronounced epithelial morphology, along with increased expression of E-cadherin and decreased expression of Slug. Although c-Kit overexpression inhibited the motility   and metast  asis of L1-expressing colorectal cancer cells, it enhanced colorectal cancer cell proliferation and tumorigenesis, arguing that separate pathways mediate tumorigenicity   and metast  asis by c-Kit. Our findings provide insights into how colorectal cancer metastasizes to the liver, the most common site of dissemination in this cancer. 
  GeneRIFs  
 KIT (L541) single nucleotide polymorphism is associated with a higher risk of   metastasis   at diagnosis and a higher risk of relapse in GIST patients. 
 study reported 3 cases of sporadic gastrointestinal stromal tumors (GISTs) with exon 8 c-kit gene mutations; results confirmed exon 8 mutations are minor but actually existing abnormalities in sporadic GISTs and suggested such GISTs have a feature of extragastric development and   metastasis  -prone nature 
 Kit mutations are associated with recurrence and   metastasis   in gastrointestinal stromal tumors. 
 Bone-induced c-kit expression in prostate cancer cells leads to BRCA2 downregulation and supports bone   metastasis  . 
 c-Kit is suppressed in human colon cancer tissue and contributes to L1-mediated   metastasis  . 
 Correlation sduggested between KIT expression and a worse prognosis in Merkel cell carcinoma patients, raising the possibility of an active role of this receptor in tumor progression and   metastasis  . 
 C-kit mutation is closely related with the incidence of recurrence and   metastasis   in gastrointestinal stromal tumors. 
 Loss of heterozygosity of the c-kit gene in resected liver Gastrointestinal Stromal Neoplasms may be a helpful factor in the prediction of the prognosis of patients with liver   metastasis  . 
  [Cc]ell [Ii]nvasion  PubMed  
 Bone-induced c-kit expression in prostate cancer: a driver of intraosseous tumor growth. Loss of BRCA2 function stimulates prostate cancer (PCa)   cell invasion   and is associated with more aggressive and metastatic tumors in PCa patients. Concurrently, the receptor tyrosine kinase c-kit is highly expressed in skeletal metastases of PCa patients and induced in PCa cells placed into the bone microenvironment in experimental models. However, the precise requirement of c-kit for intraosseous growth of PCa and its relation to BRCA2 expression remain unexplored. Here, we show that c-kit expression promotes migration and invasion of PCa cells. Alongside, we found that c-kit expression in PCa cells parallels BRCA2 downregulation. Gene rescue experiments with human BRCA2 transgene in c-kit-transfected PCa cells resulted in reduction of c-kit protein expression and migration and invasion, suggesting a functional significance of BRCA2 downregulation by c-kit. The inverse association between c-kit and BRCA2 gene expressions in PCa cells was confirmed using laser capture microdissection in experimental intraosseous tumors and bone metastases of PCa patients. Inhibition of bone-induced c-kit expression in PCa cells transduced with lentiviral short hairpin RNA reduced intraosseous tumor incidence and growth. Overall, our results provide evidence of a novel pathway that links bone-induced c-kit expression in PCa cells to BRCA2 downregulation and supports bone metastasis. 
 Oncogenic KIT-containing exosomes increase gastrointestinal stromal tumor   cell invasion  . During tumor development, constant interplay occurs between tumor cells and surrounding stromal cells. We report evidence that gastrointestinal stromal tumor (GIST) cells invade the interstitial stroma through the release of the oncogenic protein tyrosine kinase (KIT)-containing exosomes, which triggers the phenotypic conversion of progenitor smooth muscle cells to tumor-promoting cells. These recipient cells display morphologic changes and acquire tumor-associated phenotypes, including enhanced adhesion to extracellular matrix proteins, activation of intracellular pathways downstream of KIT, expression of Interstitial Cell of Cajal-like markers, and release of various matrix metalloproteinases (MMPs), particularly MMP1. This report shows stimulation of MMP1 production by stromal cells via uptake of tumor-derived exosomes, which leads to tumor   cell invasion  . Exosomes derived from GIST patients but not healthy donors show enhanced MMP1 secretion by smooth muscle cells and tumor   cell invasion  , whereas selective blocking of exosome-mediated MMP1 secretion decreases tumor invasiveness. Our study indicates that exosome release and subsequent MMP1 induction creates a positive feedback mechanism established between tumor and stromal cells that drives GIST development and offers unique insights for potential therapeutic strategies to block GIST progression and metastatic spread. 
 C-kit induces epithelial-mesenchymal transition and contributes to salivary adenoid cystic cancer progression. Epithelial-mesenchymal transition (EMT) is associated with salivary adenoid cystic cancer (ACC) progression and metastasis. Here, we report that ectopic overexpression of c-kit in ACC cell lines is sufficient for acquisition of mesenchymal traits, enhanced   cell invasion  , along with stem cell properties defined by the presence of a CD133+/CD44+ cell subpopulation. c-kit positively regulated expression of known EMT inducers, also activating TGF-β to contribute to EMT. c-kit itself was induced by TGF-β in ACC cell lines and required for TGF-β-induced EMT. Xenograft experiments showed that c-kit cooperated with oncogenic Ras to promote tumorigenesis in vivo. Finally, in human specimens of ACC, we found that c-kit was abnormally overexpressed and correlated with the prognosis of ACC. Our findings define an important function for c-kit in ACC progression by orchestrating EMT, and they implicate this gene product as a marker of poor prognosis in this disease. 
  GeneRIFs  
 Oncogenic KIT-containing exosomes increase gastrointestinal stromal tumor   cell invasion  . 
   
  
 FGFRL1 
 
  NCBI Gene  
  GeneRIF  
  Pubmed  
 
 
  Search Term  Rank  Fields  
  [Mm]igration  6  PubMed(1)  
 
 
 hide/show details for FGFRL1 
  [Mm]igration  PubMed  
 Examination of FGFRL1 as a candidate gene for diaphragmatic defects at chromosome 4p16.3 shows that Fgfrl1 null mice have reduced expression of Tpm3, sarcomere genes and Lrtm1 in the diaphragm. Fgfrl1 (also known as Fgfr5; OMIM 605830) homozygous null mice have thin, amuscular diaphragms and die at birth because of diaphragm hypoplasia. FGFRL1 is located at 4p16.3, and this chromosome region can be deleted in patients with congenital diaphragmatic hernia (CDH). We examined FGFRL1 as a candidate gene for the diaphragmatic defects associated with 4p16.3 deletions and re-sequenced this gene in 54 patients with CDH. We confirmed six known coding single nucleotide polymorphisms (SNPs): c.209G > A (p.Pro20Pro), c.977G > A (p.Pro276Pro), c.1040T > C (p.Asp297Asp), c.1234C > A (p.Pro362Gln), c.1420G > T (p.Arg424Leu), and c.1540C > T (p.Pro464Leu), but we did not identify any gene mutations. We genotyped additional CDH patients for four of these six SNPs, including the three non-synonymous SNPs, to make a total of 200 chromosomes, and found that the allele frequency for the four SNPs, did not differ significantly between patients and normal controls (p > or = 0.05). We then used Affymetrix Genechip Mouse Gene 1.0 ST arrays and found eight genes with significantly reduced expression levels in the diaphragms of Fgfrl1 homozygous null mice when compared with wildtype mice-Tpm3, Fgfrl1 (p = 0.004), Myl2, Lrtm1, Myh4, Myl3, Myh7 and Hephl1. Lrtm1 is closely related to Slit3, a protein associated with herniation of the central tendon of the diaphragm in mice. The Slit proteins are known to regulate axon branching and cell   migration  , and inhibition of Slit3 reduces cell motility and decreases the expression of Rac and Cdc42, two genes that are essential for myoblast fusion. Further studies to determine if Lrtm1 has a similar function to Slit3 and if reduced Fgfrl1 expression can cause diaphragm hypoplasia through a mechanism involving decreased myoblast motility and/or myoblast fusion, seem indicated. 
   
  
 PDGFRB 
 
  NCBI Gene  
  GeneRIF  
  Pubmed  
 
 
  Search Term  Rank  Fields  
  [Mm]igration  6  Process(8); PubMed(58); GeneRIFs(4)  
  ECM  7  PubMed(6)  
  [Ee]xtracellular [Mm]atrix  8  PubMed(9)  
  [Ii]nvasive  10  PubMed(5); GeneRIFs(1)  
  [Mm]etastasize  11  PubMed(1); GeneRIFs(1)  
  [Mm]etastasis  12  PubMed(21); GeneRIFs(3)  
  [Cc]ell [Ii]nvasion  13  PubMed(3)  
 
 
 hide/show details for PDGFRB 
  [Mm]igration  Process  
 cell   migration   
 cell   migration   involved in coronary angiogenesis 
 cell   migration   involved in vasculogenesis 
 metanephric mesenchymal cell   migration   
 positive regulation of cell   migration   
 positive regulation of metanephric mesenchymal cell   migration   by platelet-derived growth factor receptor-beta signaling pathway 
 positive regulation of smooth muscle cell   migration   
 positive regulation of smooth muscle cell   migration   
  PubMed  
 Signal transduction via platelet-derived growth factor receptors. Platelet-derived growth factor (PDGF) exerts its stimulatory effects on cell growth and motility by binding to two related protein tyrosine kinase receptors. Ligand binding induces receptor dimerization and autophosphorylation, allowing binding and activation of cytoplasmic SH2-domain containing signal transduction molecules. Thereby, a number of different signaling pathways are initiated leading to cell growth, actin reorganization   migration   and differentiation. Recent observations suggest that extensive cross-talk occurs between different signaling pathways, and that stimulatory signals are modulated by inhibitory signals arising in parallel. 
 Mesenchymal stem cell   migration   is regulated by fibronectin through α5β1-integrin-mediated activation of PDGFR-β and potentiation of growth factor signals. Ce  ll migrat  ion during vascular remodelling is regulated by crosstalk between growth factor receptors and integrin receptors, which together coordinate cytoskeletal and motogenic changes. Here, we report extracellular matrix (ECM)-directed crosstalk between platelet-derived growth factor receptor (PDGFR)-β and α5β1-integrin, which control  s the mig  ration of mesenchymal stem (stromal) cells (MSCs). Cell adhesion to fibronectin induced α5β1-integrin-dependent phosphorylation of PDGFR-β in the absence of growth factor stimulation. Phosphorylated PDGFR-β co-immunoprecipitated with α5-integrin and colocalised with α5β1-integrin in the transient tidemarks of focal adhesions. Adhesion to fibronectin also strongly potentiated PDGF-BB-induced PDGFR-β phosphorylation and focal adhesion kinase (FAK) activity, in an α5β1-integrin-dependent manner. PDGFR-β-induced phosphoinositide 3-kinase (PI3K) and Akt activity, actin reorgan  isation a  nd cell migration were all regulated by fibronectin and α5β1-integrin. This synergistic relationship between α5β1-integrin and PDGFR-β is a fundamenta  l determi  nant of cell migration. Thus, fibronectin-rich matrices can prime PDGFR-β to recruit mesenchymal cells at sites of vascular remodelling. 
 Structural determinants in the platelet-derived growth factor alpha-receptor implicated in modulation of chemotaxis. Activation of the platelet-derived growth factor (PDGF) beta-receptor leads to cell growth and chemotaxis. The PDGF alpha-receptor also mediates a mitogenic signal, but fails to induce cell   migration   in certain cell types. To examine this difference in signal transduction, a series of point-mutated PDGF alpha-receptors were analyzed. Porcine aortic endothelial cells expressing mutant PDGF alpha-receptors, in which tyrosine residues 768, 993, or 1018 were changed to phenylalanine residues migrated toward PDGF, whereas wild-type alpha-receptors and mutant alpha-receptors changed at tyrosine residues 720, 944, or 988 failed to migrate. All mutant receptors were mitogenically active and their capacity to activate phosphatidylinositol 3'-kinase and phospholipase C-gamma was not different from that of the wild-type receptor. Tyr-768 was found to be phosphorylated in PDGF-stimulated cells; in the Y768F mutant, there was a considerable increase in phosphorylation of Ser-767. Tyr-993 was not phosphorylated, but mutation of this tyrosine residue to a phenylalanine residue resulted in increased efficiency of phosphorylation on Tyr-988. Tyr-1018 is known to be an autophosphorylation site. Phosphorylated Tyr-768 and Tyr-1018 may bind signal transduction molecules involved in negative modulation of the chemotactic signaling capacity, whereas phosphorylated Tyr-988 may mediate increased chemotaxis. Thus our data indicate that the PDGF alpha-receptor has an intrinsic ability to transduce a chemotactic signal, and that this signal is counteracted by overriding negative signals. 
 PDGFs and their receptors. The platelet-derived growth factor (PDGF)/PDGFR receptor (PDGFR) family is essential for a vast array of physiological processes such as   migration   and proliferation of percityes that contribute to the formation and proper function of blood vessels. While ligand-dependent de-repression of the PDGFR's kinase activity is the major mode by which the PDGFR is activated, there are additional mechanisms to activate PDGFRs. Deregulated PDGFR activity contributes to various pathological conditions, and hence the PDGF/PDGFR family members are viable therapeutic targets. An increased appreciation of which PDGFR contributes to pathology, biomarkers that indicate the amplitude and mode of activation, and receptor-specific antagonists are necessary for the development of next-generation therapies that target the PDGF/PDGFR family. 
 Combined anti-PDGFRalpha and PDGFRbeta targeting in non-small cell lung cancer. Activation of the platelet-derived growth factor (PDGF)-receptors is critically involved into various stromal cell functions including recruitment of stromal cells and vascular endothelial growth factor (VEGF) induction in tumor and perivascular cells. To evaluate the effects of combined PDGFRalpha and -beta inhibition in a non-small cell lung cancer model, we stably transfected A549 lung cancer cells with the PDGF-A mutant PDGF-0. PDGF-0 has been generated by substituting amino acids in the binding region of PDGF-A with the corresponding VEGF-A region, leading to a decreased receptor-binding affinity and activation. Compared with control vector transfected cells, transfection with PDGF-0 had no impact on monolayer growth and apoptosis in vitro, but significantly impaired the number of colony formation in soft agar. After subcutaneous injections, all mice developed tumors within 5 days. While control vector transfected A549 cells were characterized by constant tumor growth, PDGF-0 transfected A549 revealed a reduced tumor mass (p < 0.001) with no further growth beyond 14 days (2 months observation time) and complete regressions in 7 of 13 cases. Immunohistochemical analyses revealed that PDGF-0 transfected tumors demonstrated decreased recruitment of periendothelial cells, while the tumor invasion zone was similar to control vector transfectants. Similarly, conditioned medium from PDGF-0 transfected cells induced significantly less   migration   of smooth muscle cells and fibroblasts in vitro. Interestingly, in PDGF-0 transfectants, neither total vessel count nor VEGF expression were significantly altered. These studies demonstrate that combined inhibition of PDGFRalpha and -beta results in markedly decreased tumor growth in vivo because of impaired recruitment of periendothelial cells. 
 A new look at platelet-derived growth factor in renal disease. The PDGF system, comprising four isoforms (PDGF-A, -B, -C, and -D) and two receptor chains (PDGFR-alpha and -beta), plays important roles in wound healing, atherosclerosis, fibrosis, and malignancy. Components of the system are expressed constitutively or inducibly in most renal cells. They regulate a multitude of pathophysiologic events, ranging from cell proliferation and   migration   to extracellular matrix accumulation, production of pro- and anti-inflammatory mediators, tissue permeability, and regulation of hemodynamics. Genetic deletion of PDGF-B or PDGFR-beta results in an absent glomerular mesangium, whereas PDGF-C and PDGFR-alpha contribute to the formation of the renal cortical interstitium. Almost all experimental and human renal diseases are characterized by altered expression of components of the PDGF system. Infusion or systemic overexpression of PDGF-B or -D induces prominent mesangioproliferative changes and renal fibrosis. Intervention studies identified PDGF-C as a mediator of renal interstitial fibrosis and PDGF-B and -D as key factors involved in mesangioproliferative disease and renal interstitial fibrosis. These data establish PDGF as one of the best characterized growth factors in renal disease and the most potent stimulus of mesangial cell proliferation currently identified. Accordingly, targeted intervention against the various PDGF isoforms offers a promising novel therapeutic approach to renal disease. 
 MicroRNA-34A inhibits the growth, invasion and metastasis of gastric cancer by targeting PDGFR and MET expression. Within the family of RTKs (receptor tyrosine kinases), PDGFR (platelet-derived growth factor receptor) has been implicated in carcinogenesis and tumour development. miRNAs (microRNAs), which can target the mRNAs (messenger RNAs) of cancer-associated genes, are abnormally expressed in various cancers. In this study, our aim was to identify the miRNAs that target PDGFR-α/β and to study the functions of these miRNAs. miR-34a was predicted to target PDGFR, and luciferase reporter assays showed that miR-34a could directly target PDGFR. Meanwhile, we found that miR-34a was down-regulated in gastric cancer tissues and was associated with metastasis. Our findings showed that miR-34a could inhibit gastric cancer cel  l migrati  on, invasion and proliferation, but these tumourigenic properties were only partially restored when PDGFR-α/β was overexpressed. In subsequent experiments, we found that the overexpression of both PDGFR and MET could completely restore the gastric cancer tumourigenic properties. Moreover, the cancer-associated cell signalling pathway was studied, and we found that miR-34a could inhibit Akt [PKB (protein kinase B)] phosphorylation, which was restored by the overexpression of both PDGFR and MET. In conclusion, miR-34a may act as a potential tumour suppressor in gastric cancer and is associated with the mechanisms of gastric cancer metastasis; miR-34a can inhibit gastric cancer tumourigenesis by targeting PDGFR and MET through the PI3K (phosphoinositide 3-kinase)/Akt pathway. 
 Analysis of the myosin-II-responsive focal adhesion proteome reveals a role for β-Pix in negative regulation of focal adhesion maturation. Focal adhesions undergo myosin-II-mediated maturation wherein they grow and change composition to modulate integrin signalling for cell   migratio  n, growth and differentiation. To determine how focal adhesion composition is affected by myosin II activity, we performed proteomic analysis of isolated focal adhesions and compared protein abundance in focal adhesions from cells with and without myosin II inhibition. We identified 905 focal adhesion proteins, 459 of which changed in abundance with myosin II inhibition, defining the myosin-II-responsive focal adhesion proteome. The abundance of 73% of the proteins in the myosin-II-responsive focal adhesion proteome was enhanced by contractility, including proteins involved in Rho-mediated focal adhesion maturation and endocytosis- and calpain-dependent focal adhesion disassembly. During myosin II inhibition, 27% of proteins in the myosin-II-responsive focal adhesion proteome, including proteins involved in Rac-mediated lamellipodial protrusion, were enriched in focal adhesions, establishing that focal adhesion protein recruitment is also negatively regulated by contractility. We focused on the Rac guanine nucleotide exchange factor β-Pix, documenting its role in the negative regulation of focal adhesion maturation and the promotion of lamellipodial protrusion and focal adhesion turnover to drive cel  l migrati  on. 
 Human cytomegalovirus downregulates expression of receptors for platelet-derived growth factor by smooth muscle cells. Infection by human cytomegalovirus (HCMV) is associated with the development of vascular diseases and may cause severe brain damage in infected fetuses. Platelet-derived growth factor receptors alpha and beta (PDGFR-alpha and -beta) control important cellular processes associated with atherosclerosis and fetal development. In the present investigation, our goal was to determine whether infection by HCMV can influence the expression of PDGFR-alpha and -beta in human smooth muscle cells (SMCs). In connection with HCMV infection in vitro the levels of PDGFR-alpha and -beta at the cell surface and in the total cellular protein of SMCs were reduced in parallel with decreases in the levels of the corresponding mRNAs. These effects were dependent on immediate-early (IE) or early (E) HCMV gene products, since inhibition of late genes did not prevent HCMV from affecting the expression of PDGFR-alpha and -beta. The downregulation of PDGFR caused by HCMV was dose dependent. Furthermore, confocal microscopy revealed that the localization of PDGFR-beta was altered in HCMV-infected cells, in which this protein colocalized with proteins associated with endosomes (Rab4 and -5) and lysosomes (Lamp1 and -2), indicating entrance into pathways for protein degradation. Altogether these observations indicate that an IE and/or E HCMV protein(s) downregulates the expression of PDGFR-alpha and -beta in SMCs. This phenomenon may disrupt cellular processes of importance in connection with cellular differentiation,   migration  , and/or proliferation. These observations may explain why congenital infection with HCMV can cause fetal brain damage. 
 Platelet-derived growth factor receptor signaling is not involved in osteogenic differentiation of human mesenchymal stem cells. Platelet-derived growth factor (PDGF) receptor signaling plays an important role in the regulation of proliferation and   migration   of skeletal cells such as osteoblasts or mesenchymal stem cells (MSCs). However, involvement of these receptors in the process of osteoblastic differentiation of MSCs is still a matter of debate. The aim of our study was to examine the role of PDGF receptor signaling in osteogenic differentiation of human MSCs. For this purpose, we performed PDGF receptor stimulation as well as inhibition experiments. Inhibition experiments were carried out with Tyrphostin AG1296, a potent and specific inhibitor of PDGF receptor activity. As expected, Tyrphostin AG1296 treatment caused a concentration-dependent decrease in fetal calf serum and PDGF-BB-induced proliferation of MSCs and effectively inhibited PDGF-BB-induced phosphorylation of extracellular-regulated kinase 1/2. However, PDGF receptor inhibition had no significant effect on osteoblastic differentiation of MSCs, as evaluated histochemically by von Kossa, Alizarin-Red, and osteocalcin stainings. Moreover, mineralized matrix production, as assayed by quantitative Ca(2+)-measurements, was also not modulated by Tyrphostin AG1296 treatment. These results were noticeable irrespective of whether MSCs were grown under nonosteogenic or osteogenic differentiation conditions. Similarly, PDGF-BB treatment of MSCs in receptor stimulation experiments also failed to modulate mineralization. However, expression of alkaline phosphatase was suppressed by Tyrphostin AG1296 treatment at later stages of osteogenesis but not in the early stages, as assessed by enzyme activity and mRNA expression assays. Expression of other osteogenic marker genes such as osteocalcin, runt-related transcription factor 2, osteopontin, collagen type I, and bone sialoprotein was almost unaffected in our perturbation studies. From these experiments, we conclude that PDGF receptor signaling sustains proliferation without affecting osteogenic differentiation of MSCs. 
 Vascular endothelial growth factor can signal through platelet-derived growth factor receptors. Vascular endothelial growth factor (VEGF-A) is a crucial stimulator of vascular cell   migration   and proliferation. Using bone marrow-derived human adult mesenchymal stem cells (MSCs) that did not express VEGF receptors, we provide evidence that VEGF-A can stimulate platelet-derived growth factor receptors (PDGFRs), thereby regulating MSC   migration   and proliferation. VEGF-A binds to both PDGFRalpha and PDGFRbeta and induces tyrosine phosphorylation that, when inhibited, results in attenuation of VEGF-A-induced MSC   migration   and proliferation. This mechanism was also shown to mediate human dermal fibroblast (HDF)   migration  . VEGF-A/PDGFR signaling has the potential to regulate vascular cell recruitment and proliferation during tissue regeneration and disease. 
 PTEN tumor suppressor associates with NHERF proteins to attenuate PDGF receptor signaling. PTEN, a tumor suppressor frequently inactivated in many human cancers, directly antagonizes the activity of phosphatidylinositol-3-OH kinase (PI3K) by dephosphorylating phosphoinositides. We show here that PTEN interacts directly with the NHERF1 and NHERF2 (Na+/H+ exchanger regulatory factor) homologous adaptor proteins through the PDZ motif of PTEN and the PDZ1 domain of NHERF1 or both PDZ domains of NHERF2. NHERFs were shown to interact directly with platelet-derived growth factor receptor (PDGFR), and we demonstrate the assembly of a ternary complex between PTEN, NHERFs and PDGFR. The activation of the PI3K pathway after PDGFR stimulation was prolonged in NHERF1(-/-) mouse embryonic fibroblasts as compared to wild-type cells, consistent with defective PTEN recruitment to PDGFR in the absence of NHERF1. Depletion of NHERF2 by small interfering RNA similarly increased PI3K signaling. Phenotypically, the loss of NHERF1 enhanced the PDGF-induced cytoskeletal rearrangements and chemotactic   migration   of the cells. These data indicate that, in normal cells, NHERF proteins recruit PTEN to PDGFR to restrict the activation of the PI3K. 
 Analysis of protein-protein interactions in cross-talk pathways reveals CRKL protein as a novel prognostic marker in hepatocellular carcinoma. Deciphering the network of signaling pathways in cancer via protein-protein interactions (PPIs) at the cellular level is a promising approach but remains incomplete. We used an in situ proximity ligation assay to identify and quantify 67 endogenous PPIs among 21 interlinked pathways in two hepatocellular carcinoma (HCC) cells, Huh7 (minimally migratory cells) and Mahlavu (highly migratory cells). We then applied a differential network biology analysis and determined that the novel interaction, CRKL-FLT1, has a high centrality ranking, and the expression of this interaction is strongly correlated with the migratory ability of HCC and other cancer cell lines. Knockdown of CRKL and FLT1 in HCC cells leads to a decrease in cell   migration   via ERK signaling and the epithelial-mesenchymal transition process. Our immunohistochemical analysis shows high expression levels of the CRKL and CRKL-FLT1 pair that strongly correlate with reduced disease-free and overall survival in HCC patient samples, and a multivariate analysis further established CRKL and the CRKL-FLT1 as novel prognosis markers. This study demonstrated that functional exploration of a disease network with interlinked pathways via PPIs can be used to discover novel biomarkers. 
 Combined vascular endothelial growth factor and platelet-derived growth factor inhibition in rat cardiac allografts: beneficial effects on inflammation and smooth muscle cell proliferation. Perivascular inflammation and subsequent smooth muscle cell (SMC) proliferation are central in the development of cardiac allograft arteriosclerosis. We examined the effect of combined inhibition of proinflammatory vascular endothelial growth factor (VEGF) and SMC mitogen platelet-derived growth factor (PDGF) in rat cardiac allografts.Heterotopic cardiac transplantations were performed between fully major histocompatibility mismatched rat strains receiving cyclosporine A immunosuppression. In situ hybridization and immunohistochemistry were performed to examine VEGF and PDGF ligand and receptor (R) expression. Protein tyrosine kinase inhibitors PTK787 and imatinib were used to inhibit VEGFR and PDGFR activity, respectively. Rat coronary artery SMC   migration   and proliferation assays were used to examine the effect of VEGF and PDGF and tyrosine kinase inhibitors in vitro.Both ligand and receptor expression of VEGF and PDGF were detected in chronically rejecting allografts. In vitro, PDGF-BB mediated rat coronary artery SMC   migration   and proliferation was completely inhibited with imatinib and partially with PTK787. In vivo, combined treatment with PTK787 and imatinib significantly reduced the formation of neointimal lesions in arteries of cardiac allografts at 8 weeks, producing a greater effect than either drug alone. PTK787, in contrast with imatinib, reduced the number of ED1 macrophages and PDGF-B immunoreactivity in the allografts at 4 weeks.Blocking VEGF and PDGF receptor signaling in cardiac allografts has distinctive effects on inflammation and SMC proliferation, suggesting that targeting both inflammation and pathologic vascular remodeling may be needed to inhibit cardiac allograft arteriosclerosis. 
 PDGFs and their receptors. The platelet-derived growth factor (PDGF)/PDGFR receptor (PDGFR) family is essential for a vast array of physiological processes such as   migration   and proliferation of percityes that contribute to the formation and proper function of blood vessels. While ligand-dependent de-repression of the PDGFR's kinase activity is the major mode by which the PDGFR is activated, there are additional mechanisms to activate PDGFRs. Deregulated PDGFR activity contributes to various pathological conditions, and hence the PDGF/PDGFR family members are viable therapeutic targets. An increased appreciation of which PDGFR contributes to pathology, biomarkers that indicate the amplitude and mode of activation, and receptor-specific antagonists are necessary for the development of next-generation therapies that target the PDGF/PDGFR family. 
 MicroRNA-34A inhibits the growth, invasion and metastasis of gastric cancer by targeting PDGFR and MET expression. Within the family of RTKs (receptor tyrosine kinases), PDGFR (platelet-derived growth factor receptor) has been implicated in carcinogenesis and tumour development. miRNAs (microRNAs), which can target the mRNAs (messenger RNAs) of cancer-associated genes, are abnormally expressed in various cancers. In this study, our aim was to identify the miRNAs that target PDGFR-α/β and to study the functions of these miRNAs. miR-34a was predicted to target PDGFR, and luciferase reporter assays showed that miR-34a could directly target PDGFR. Meanwhile, we found that miR-34a was down-regulated in gastric cancer tissues and was associated with metastasis. Our findings showed that miR-34a could inhibit gastric cancer cel  l migrati  on, invasion and proliferation, but these tumourigenic properties were only partially restored when PDGFR-α/β was overexpressed. In subsequent experiments, we found that the overexpression of both PDGFR and MET could completely restore the gastric cancer tumourigenic properties. Moreover, the cancer-associated cell signalling pathway was studied, and we found that miR-34a could inhibit Akt [PKB (protein kinase B)] phosphorylation, which was restored by the overexpression of both PDGFR and MET. In conclusion, miR-34a may act as a potential tumour suppressor in gastric cancer and is associated with the mechanisms of gastric cancer metastasis; miR-34a can inhibit gastric cancer tumourigenesis by targeting PDGFR and MET through the PI3K (phosphoinositide 3-kinase)/Akt pathway. 
 Mesenchymal stem cell   migration   is regulated by fibronectin through α5β1-integrin-mediated activation of PDGFR-β and potentiation of growth factor signals. Ce  ll migrat  ion during vascular remodelling is regulated by crosstalk between growth factor receptors and integrin receptors, which together coordinate cytoskeletal and motogenic changes. Here, we report extracellular matrix (ECM)-directed crosstalk between platelet-derived growth factor receptor (PDGFR)-β and α5β1-integrin, which control  s the mig  ration of mesenchymal stem (stromal) cells (MSCs). Cell adhesion to fibronectin induced α5β1-integrin-dependent phosphorylation of PDGFR-β in the absence of growth factor stimulation. Phosphorylated PDGFR-β co-immunoprecipitated with α5-integrin and colocalised with α5β1-integrin in the transient tidemarks of focal adhesions. Adhesion to fibronectin also strongly potentiated PDGF-BB-induced PDGFR-β phosphorylation and focal adhesion kinase (FAK) activity, in an α5β1-integrin-dependent manner. PDGFR-β-induced phosphoinositide 3-kinase (PI3K) and Akt activity, actin reorgan  isation a  nd cell migration were all regulated by fibronectin and α5β1-integrin. This synergistic relationship between α5β1-integrin and PDGFR-β is a fundamenta  l determi  nant of cell migration. Thus, fibronectin-rich matrices can prime PDGFR-β to recruit mesenchymal cells at sites of vascular remodelling. 
 Platelet-derived growth factor receptor signaling is not involved in osteogenic differentiation of human mesenchymal stem cells. Platelet-derived growth factor (PDGF) receptor signaling plays an important role in the regulation of proliferation and   migration   of skeletal cells such as osteoblasts or mesenchymal stem cells (MSCs). However, involvement of these receptors in the process of osteoblastic differentiation of MSCs is still a matter of debate. The aim of our study was to examine the role of PDGF receptor signaling in osteogenic differentiation of human MSCs. For this purpose, we performed PDGF receptor stimulation as well as inhibition experiments. Inhibition experiments were carried out with Tyrphostin AG1296, a potent and specific inhibitor of PDGF receptor activity. As expected, Tyrphostin AG1296 treatment caused a concentration-dependent decrease in fetal calf serum and PDGF-BB-induced proliferation of MSCs and effectively inhibited PDGF-BB-induced phosphorylation of extracellular-regulated kinase 1/2. However, PDGF receptor inhibition had no significant effect on osteoblastic differentiation of MSCs, as evaluated histochemically by von Kossa, Alizarin-Red, and osteocalcin stainings. Moreover, mineralized matrix production, as assayed by quantitative Ca(2+)-measurements, was also not modulated by Tyrphostin AG1296 treatment. These results were noticeable irrespective of whether MSCs were grown under nonosteogenic or osteogenic differentiation conditions. Similarly, PDGF-BB treatment of MSCs in receptor stimulation experiments also failed to modulate mineralization. However, expression of alkaline phosphatase was suppressed by Tyrphostin AG1296 treatment at later stages of osteogenesis but not in the early stages, as assessed by enzyme activity and mRNA expression assays. Expression of other osteogenic marker genes such as osteocalcin, runt-related transcription factor 2, osteopontin, collagen type I, and bone sialoprotein was almost unaffected in our perturbation studies. From these experiments, we conclude that PDGF receptor signaling sustains proliferation without affecting osteogenic differentiation of MSCs. 
 Combined anti-PDGFRalpha and PDGFRbeta targeting in non-small cell lung cancer. Activation of the platelet-derived growth factor (PDGF)-receptors is critically involved into various stromal cell functions including recruitment of stromal cells and vascular endothelial growth factor (VEGF) induction in tumor and perivascular cells. To evaluate the effects of combined PDGFRalpha and -beta inhibition in a non-small cell lung cancer model, we stably transfected A549 lung cancer cells with the PDGF-A mutant PDGF-0. PDGF-0 has been generated by substituting amino acids in the binding region of PDGF-A with the corresponding VEGF-A region, leading to a decreased receptor-binding affinity and activation. Compared with control vector transfected cells, transfection with PDGF-0 had no impact on monolayer growth and apoptosis in vitro, but significantly impaired the number of colony formation in soft agar. After subcutaneous injections, all mice developed tumors within 5 days. While control vector transfected A549 cells were characterized by constant tumor growth, PDGF-0 transfected A549 revealed a reduced tumor mass (p < 0.001) with no further growth beyond 14 days (2 months observation time) and complete regressions in 7 of 13 cases. Immunohistochemical analyses revealed that PDGF-0 transfected tumors demonstrated decreased recruitment of periendothelial cells, while the tumor invasion zone was similar to control vector transfectants. Similarly, conditioned medium from PDGF-0 transfected cells induced significantly less   migration   of smooth muscle cells and fibroblasts in vitro. Interestingly, in PDGF-0 transfectants, neither total vessel count nor VEGF expression were significantly altered. These studies demonstrate that combined inhibition of PDGFRalpha and -beta results in markedly decreased tumor growth in vivo because of impaired recruitment of periendothelial cells. 
 A new look at platelet-derived growth factor in renal disease. The PDGF system, comprising four isoforms (PDGF-A, -B, -C, and -D) and two receptor chains (PDGFR-alpha and -beta), plays important roles in wound healing, atherosclerosis, fibrosis, and malignancy. Components of the system are expressed constitutively or inducibly in most renal cells. They regulate a multitude of pathophysiologic events, ranging from cell proliferation and   migration   to extracellular matrix accumulation, production of pro- and anti-inflammatory mediators, tissue permeability, and regulation of hemodynamics. Genetic deletion of PDGF-B or PDGFR-beta results in an absent glomerular mesangium, whereas PDGF-C and PDGFR-alpha contribute to the formation of the renal cortical interstitium. Almost all experimental and human renal diseases are characterized by altered expression of components of the PDGF system. Infusion or systemic overexpression of PDGF-B or -D induces prominent mesangioproliferative changes and renal fibrosis. Intervention studies identified PDGF-C as a mediator of renal interstitial fibrosis and PDGF-B and -D as key factors involved in mesangioproliferative disease and renal interstitial fibrosis. These data establish PDGF as one of the best characterized growth factors in renal disease and the most potent stimulus of mesangial cell proliferation currently identified. Accordingly, targeted intervention against the various PDGF isoforms offers a promising novel therapeutic approach to renal disease. 
 Human cytomegalovirus downregulates expression of receptors for platelet-derived growth factor by smooth muscle cells. Infection by human cytomegalovirus (HCMV) is associated with the development of vascular diseases and may cause severe brain damage in infected fetuses. Platelet-derived growth factor receptors alpha and beta (PDGFR-alpha and -beta) control important cellular processes associated with atherosclerosis and fetal development. In the present investigation, our goal was to determine whether infection by HCMV can influence the expression of PDGFR-alpha and -beta in human smooth muscle cells (SMCs). In connection with HCMV infection in vitro the levels of PDGFR-alpha and -beta at the cell surface and in the total cellular protein of SMCs were reduced in parallel with decreases in the levels of the corresponding mRNAs. These effects were dependent on immediate-early (IE) or early (E) HCMV gene products, since inhibition of late genes did not prevent HCMV from affecting the expression of PDGFR-alpha and -beta. The downregulation of PDGFR caused by HCMV was dose dependent. Furthermore, confocal microscopy revealed that the localization of PDGFR-beta was altered in HCMV-infected cells, in which this protein colocalized with proteins associated with endosomes (Rab4 and -5) and lysosomes (Lamp1 and -2), indicating entrance into pathways for protein degradation. Altogether these observations indicate that an IE and/or E HCMV protein(s) downregulates the expression of PDGFR-alpha and -beta in SMCs. This phenomenon may disrupt cellular processes of importance in connection with cellular differentiation,   migration  , and/or proliferation. These observations may explain why congenital infection with HCMV can cause fetal brain damage. 
 Combined vascular endothelial growth factor and platelet-derived growth factor inhibition in rat cardiac allografts: beneficial effects on inflammation and smooth muscle cell proliferation. Perivascular inflammation and subsequent smooth muscle cell (SMC) proliferation are central in the development of cardiac allograft arteriosclerosis. We examined the effect of combined inhibition of proinflammatory vascular endothelial growth factor (VEGF) and SMC mitogen platelet-derived growth factor (PDGF) in rat cardiac allografts.Heterotopic cardiac transplantations were performed between fully major histocompatibility mismatched rat strains receiving cyclosporine A immunosuppression. In situ hybridization and immunohistochemistry were performed to examine VEGF and PDGF ligand and receptor (R) expression. Protein tyrosine kinase inhibitors PTK787 and imatinib were used to inhibit VEGFR and PDGFR activity, respectively. Rat coronary artery SMC   migration   and proliferation assays were used to examine the effect of VEGF and PDGF and tyrosine kinase inhibitors in vitro.Both ligand and receptor expression of VEGF and PDGF were detected in chronically rejecting allografts. In vitro, PDGF-BB mediated rat coronary artery SMC   migration   and proliferation was completely inhibited with imatinib and partially with PTK787. In vivo, combined treatment with PTK787 and imatinib significantly reduced the formation of neointimal lesions in arteries of cardiac allografts at 8 weeks, producing a greater effect than either drug alone. PTK787, in contrast with imatinib, reduced the number of ED1 macrophages and PDGF-B immunoreactivity in the allografts at 4 weeks.Blocking VEGF and PDGF receptor signaling in cardiac allografts has distinctive effects on inflammation and SMC proliferation, suggesting that targeting both inflammation and pathologic vascular remodeling may be needed to inhibit cardiac allograft arteriosclerosis. 
 Regulation of platelet-derived growth factor receptor function by integrin-associated cell surface transglutaminase. A functional collaboration between growth factor receptors such as platelet derived growth factor receptor (PDGFR) and integrins is required for effective signal transduction in response to soluble growth factors. However, the mechanisms of synergistic PDGFR/integrin signaling remain poorly understood. Our previous work showed that cell surface tissue transglutaminase (tTG) induces clustering of integrins and amplifies integrin signaling by acting as an integrin binding adhesion co-receptor for fibronectin. Here we report that in fibroblasts tTG enhances PDGFR-integrin association by interacting with PDGFR and bridging the two receptors on the cell surface. The interaction between tTG and PDGFR reduces cellular levels of the receptor by accelerating its turnover. Moreover, the association of PDGFR with tTG causes receptor clustering, increases PDGF binding, promotes adhesion-mediated and growth factor-induced PDGFR activation, and up-regulates downstream signaling. Importantly, tTG is required for efficient PDGF-dependent proliferation and   migration   of fibroblasts. These results reveal a previously unrecognized role for cell surface tTG in the regulation of the joint PDGFR/integrin signaling and PDGFR-dependent cell responses. 
 Breast cancer-derived K172N, D301V mutations abolish Na+/H+ exchanger regulatory factor 1 inhibition of platelet-derived growth factor receptor signaling. Na(+)/H(+) exchanger regulatory factor 1 (NHERF1) is a scaffold protein known to interact with a number of cancer-related proteins. nherf1 Mutations (K172N and D301V) were recently identified in breast cancer cells. To investigate the functional properties of NHERF1, wild-type and cancer-derived nherf1 mutations were stably expressed in SKMES-1 cells respectively. NHERF1-wt overexpression suppressed the cellular malignant phenotypes, including proliferation,   migration  , and invasion. nherf1 Mutations (K172N and D301V) caused complete or partial loss of NHERF1 functions by affecting the PTEN/NHERF1/PDGFRβ complex formation, inactivating NHERF1 inhibition of PDGF-induced AKT and ERK activation, and attenuating the tumor-suppressor effects of NHERF1-wt. These results further demonstrated the functional consequences of breast cancer-derived nherf1 mutations (K172N and D301V), and suggested the causal role of NHERF1 in tumor development and progression. 
 PDGF-D/PDGF-ββ receptor-regulated chemotaxis of malignant mesothelioma cells. Our earlier study suggested that platelet-derived growth factor (PDGF)- ββ receptor regulates chemotaxis of human malignant mesothelioma cells such as MSTO-211H, NCIH-2052, NCIH-2452, and NCIH-28 cells, but not non-malignant Met5A cells. The present study was designed to gain further insight into the PDGF-ββ receptor signals underlying the chemotaxis.PDGF-D secreted from cells, activation of Akt and ERK, and   cell mig  ration were monitored for cells with and without knocking-down PDGF-ββ receptor.FBS significantly stimulated PDGF-D secretion from malignant mesothelioma cells, but not Met5A cells. PDGF-D activated Akt and ERK in both the non-malignant and malignant cells. PDGF-D significantly faci  litated m  igration of malignant mesothelioma cells, but not Met5A cells, with the extent varying among the cell types. The facilitatory action of PDGF-D was clearly prevented by knocking-down PDGF-ββ receptor or inhibitors of PI3 kinase, PDK1, Akt, Rac1, ROCK, and MEK.The results of the present study indicate that PDGF-D promotes malignant mesothelioma cell chemotaxis through PDGF-ββ receptor signaling pathways along a PI3 kinase/PDK1/Akt/Rac1/ROCK axis and relevant to ERK activation. 
 Regulation of chemotaxis by the cytoplasmic domain of tissue factor. We previously demonstrated that FVIIa bound to tissue factor (TF) induces a hyperchemotactic response towards PDGF-BB. The aim of the present study was to investigate the role of the cytoplasmic domain of TF in cell   migration  . Porcine aortic endothelial (PAE) cells expressing human PDGF beta-receptors (PAE/PDGFRbeta) were transfected for expression of human wildtype TF (PAE/PDGFRbeta,TF), a construct lacking the cytoplasmic domain (PAE/PDGFRbeta,TFDeltacyto), a construct with alanine replacement of serine 258 (PAE/PDGFRbeta,TFS258A), or a construct with alanine replacement of serine 253, 258 and 263 in the cytoplasmic domain (PAE/PDGFRbeta,TF3SA). All stably transfected cell lines expressed functional TF. Chemotaxis was analyzed in a modified Boyden chamber assay. PAE/PDGFRbeta,TF cells stimulated with FVIIa migrated towards a 100-fold lower concentration of PDGF-BB than in the absence of FVIIa, however, hyperchemotaxis was not seen in PAE/PDGFRbeta,TFDeltacyto cells. PAE/PDGFRbeta/TFS258A and PAE/PDGFRbeta,TF3SA cells responded to low levels of PDGF-BB, but migrated a significantly shorter distance than PAE/PDGFRbeta,TF cells. Thus, hyperchemotaxis towards PDGF-BB is likely to depend in part on phosphorylation of the cytoplasmic domain of TF. We conclude that the cytoplasmic domain of TF plays a pivotal role in modulating cellular   migration   response. Our results suggest that the FVIIa/TF complex mediates intracellular signaling by alternative signal transduction pathway(s). 
 Regulation of PDGF signalling and vascular remodelling by peroxiredoxin II. Platelet-derived growth factor (PDGF) is a potent mitogenic and migratory factor that regulates the tyrosine phosphorylation of a variety of signalling proteins via intracellular production of H2O2 (refs 1, 2-3). Mammalian 2-Cys peroxiredoxin type II (Prx II; gene symbol Prdx2) is a cellular peroxidase that eliminates endogenous H2O2 produced in response to growth factors such as PDGF and epidermal growth factor; however, its involvement in growth factor signalling is largely unknown. Here we show that Prx II is a negative regulator of PDGF signalling. Prx II deficiency results in increased production of H2O2, enhanced activation of PDGF receptor (PDGFR) and phospholipase Cgamma1, and subsequently increased cell proliferation and   migration   in response to PDGF. These responses are suppressed by expression of wild-type Prx II, but not an inactive mutant. Notably, Prx II is recruited to PDGFR upon PDGF stimulation, and suppresses protein tyrosine phosphatase inactivation. Prx II also leads to the suppression of PDGFR activation in primary culture and a murine restenosis model, including PDGF-dependent neointimal thickening of vascular smooth muscle cells. These results demonstrate a localized role for endogenous H2O2 in PDGF signalling, and indicate a biological function of Prx II in cardiovascular disease. 
 Platelet-derived growth factor β-receptor, transforming growth factor β type I receptor, and CD44 protein modulate each other's signaling and stability. Growth factors, such as platelet-derived growth factor BB (PDGF-BB) and transforming growth factor β (TGFβ), are key regulators of cellular functions, including proliferati  on, migra  tion, and differentiation. Growth factor signaling is modulated by context-dependent cross-talk between different signaling pathways. We demonstrate in this study that PDGF-BB induces phosphorylation of Smad2, a downstream mediator of the canonical TGFβ pathway, in primary dermal fibroblasts. The PDGF-BB-mediated Smad2 phosphorylation was dependent on the kinase activities of both TGFβ type I receptor (TβRI) and PDGF β-receptor (PDGFRβ), and it was prevented by inhibitory antibodies against TGFβ. Inhibition of the activity of the TβRI kinase greatly reduced the PDGF-BB  -dependen  t migration in dermal fibroblasts. Moreover, we demonstrate that the receptors for PDGF-BB and TGFβ interact physically in primary dermal fibroblasts and that stimulation with PDGF-BB induces internalization not only of PDGFRβ but also of TβRI. In addition, silencing of PDGFRβ by siRNA decreased the stability of TβRI and delayed TGFβ-induced signaling. We further show that the hyaluronan receptor CD44 interacts with both PDGFRβ and TβRI. Depletion of CD44 by siRNA increased signaling via PDGFRβ and TβRI by stabilizing the receptor proteins. Our data suggest that cross-talk between PDGFRβ and TβRI occurs in dermal fibroblasts and that CD44 negatively modulates signaling via these receptors. 
 Functional cross talk between CXCR4 and PDGFR on glioblastoma cells is essential for   migration  . Glioblastoma (GBM) is the most common and aggressive form of brain tumor, characterized by high migratory behavior and infiltration in brain parenchyma which render classic therapeutic approach ineffective. The migratory behaviour of GBM cells could be conditioned by a number of tissue- and glioma-derived cytokines and growth factors. Although the pro-migratory action of CXCL12 on GBM cells in vitro and in vivo is recognized, the molecular mechanisms involved are not clearly identified. In fact the signaling pathways involved in the pro-migratory action of CXCL12 may differ in individual glioblastoma and integrate with those resulting from abnormal expression and activation of growth factor receptors. In this study we investigated whether some of the receptor tyrosine kinases commonly expressed in GBM cells could cooperate with CXCL12/CXCR4 in their migratory behavior. Our results show a functional cross-talk between CXCR4 and PDGFR which appears to be essential for GBM chemotaxis. 
 Structure of Full-Length Human PDGFRβ Bound to Its Activating Ligand PDGF-B as Determined by Negative-Stain Electron Microscopy. Members of the receptor tyrosine kinases (RTKs) regulate important cellular functions such as cell growth and   migratio  n, which are key steps in angiogenesis, in organ morphogenesis and in the unregulated states, cancer formation. One long-standing puzzle regarding RTKs centers on how the extracellular domain (ECD), which detects and binds to growth factors, is coupled with the intracellular domain kinase activation. While extensive structural works on the soluble portions of RTKs have provided critical insights into RTK structures and functions, lack of a full-length receptor structure has hindered a comprehensive overview of RTK activation. In this study, we successfully purified and determined a 27-Å-resolution structure of PDGFRβ [a full-length human platelet-derived growth factor receptor], in complex with its ligand PDGF-B. In the ligand-stimulated complex, two PDGFRβs assemble into a dimer via an extensive interface essentially running along the full-length of the receptor, suggesting that the membrane-proximal region, the transmembrane helix and the kinase domain of PDGFRβ are involved in dimerization. Major structural differences are seen between the full-length and soluble ECD structures, rationalizing previous experimental data on how membrane-proximal domains modulate receptor ligand-binding affinity and dimerization efficiency. Also, in contrast to the 2-fold symmetry of the ECD, the intracellular kinase domains adopt an asymmetric dimer arrangement, in agreement with prior observations for the closely related KIT receptor. In essence, the structure provides a first glimpse into how platelet-derived growth factor receptor ECD, upon ligand stimulation, is coupled to its intracellular domain kinase activation. 
 PDGFRβ expression and function in fibroblasts derived from pluripotent cells is linked to DNA demethylation. Platelet-derived growth factor receptor-beta (PDGFRβ) is required for the development of mesenchymal cell types, and plays a diverse role in the function of fibroblasts in tissue homeostasis and regeneration. In this study, we characterized the expression of PDGFRβ in fibroblasts derived from human embryonic stem cells (ESCs) and induced pluripotent stem cells (iPSCs), and showed that this expression is important for cellular functions such   as migrat  ion, extracellular matrix production and assembly in 3D self-assembled tissues. To determine potential regulatory regions predictive of expression of PDGFRβ following differentiation from ESCs and iPSCs, we analyzed the DNA methylation status of a region of the PDGFRB promoter that contains multiple CpG sites, before and after differentiation. We demonstrated that this promoter region is extensively demethylated following differentiation, and represents a developmentally regulated, differentially methylated region linked to PDGFRβ expression. Understanding the epigenetic regulation of genes such as PDGFRB, and identifying sites of active DNA demethylation, is essential for future applications of iPSC-derived fibroblasts for regenerative medicine. 
 Endothelial progenitor cells induce a phenotype shift in differentiated endothelial cells towards PDGF/PDGFRβ axis-mediated angiogenesis. Endothelial Progenitor Cells (EPC) support neovascularization and regeneration of injured endothelium both by providing a proliferative cell pool capable of differentiation into mature vascular endothelial cells and by secretion of angiogenic growth factors.The aim of this study was to investigate the role of PDGF-BB and PDGFRβ in EPC-mediated angiogenesis of differentiated endothelial cells.Conditioned medium from human EPC (EPC-CM) cultured in hypoxic conditions contained substantially higher levels of PDGF-BB as compared to normoxic conditions (P<0.01). EPC-CM increased proliferation (1.39-fold; P<0.001) an  d migrati  on (2.13-fold; P<0.001) of isolated human umbilical vein endothelial cells (HUVEC), as well as sprouting of vascular structures from ex vivo cultured aortic rings (2.78-fold increase; P = 0.01). The capacity of EPC-CM to modulate the PDGFRβ expression in HUVEC was assessed by western blot and RT-PCR. All the pro-angiogenic effects of EPC-CM on HUVEC could be partially inhibited by inactivation of PDGFRβ (P<0.01). EPC-CM triggered a distinct up-regulation of PDGFRβ (2.5±0.5; P<0.05) and its phosphorylation (3.6±0.6; P<0.05) in HUVEC. This was not observed after exposure of HUVEC to recombinant human PDGF-BB alone.These data indicate that EPC-CM sensitize endothelial cells and induce a pro-angiogenic phenotype including the up-regulation of PDGFRβ, thereby turning the PDGF/PDGFRβ signaling-axis into a critical element of EPC-induced endothelial angiogenesis. This finding may be utilized to enhance EPC-based therapy of ischemic tissue in future. 
 E7080, a multi-targeted tyrosine kinase inhibitor suppresses tumor cell   migration   and invasion. E7080 is an orally active multi-targeted kinase inhibitor whose targets include vascular endothelial growth factor receptors (VEGFR), fibroblast growth factor receptor (FGFR) and platelet derived growth factor receptors (PDGFR). It has been shown to inhibit tumor angiogenesis by targeting endothelial cells. A number of the targets of E7080 are also expressed on tumor cells and here we have looked at the direct effects of E7080 on tumor cell behavior.Using a panel of human tumor cell lines we determined the effect of E7080 on cell proliferation,   migration   and invasion. Inhibition of FGFR and PDGFR signaling in the cells was measured.E7080 had little effect on tumor cell proliferation. However, it blocked   migration   and invasion at concentrations that inhibited FGFR and PDGFR signaling. Knock-down of PDGFR-β in U2OS osteosarcoma cells also inhibited cell   migratio  n which, could not be further inhibited in the presence of E7080. Furthermore, E7080 could not inhibit the   migratio  n of a PDGFR negative cell line.E7080 does not significantly affect tumor cell proliferation but can inhibit their   migratio  n and invasion at concentrations that both inhibit its known targets and are achievable clinically. 
 Expression of platelet-derived growth factor receptor β is maintained by Prox1 in lymphatic endothelial cells and is required for tumor lymphangiogenesis. The lymphatic system plays important roles not only in the physiological processes, such as maintenance of tissue fluid homeostasis, but also in pathological processes including the lymph node metastasis of tumor cells. Therefore, understanding of the molecular mechanisms underlying lymphatic vessel formation is crucial. Previous studies have shown that proliferation and   migratio  n of lymphatic endothelial cells (LECs) are activated by multiple types of signals mediated by tyrosine kinase receptors such as vascular endothelial growth factor receptor (VEGFR) 3. Although signals mediated by platelet-derived growth factor receptor β (PDGFRβ) have been implicated in lymphangiogenesis, the mechanisms explaining how PDGFRβ expression is maintained in LECs remain to be fully elucidated. In the present study, we show that PDGFRβ expression in LECs is maintained by Prox1 transcription factor. Knockdown of Prox1 expression in human dermal LECs decreased the expression of PDGFRβ, leading to the lo  wered mig  ration of human dermal LECs towards PDGF-BB. Furthermore, we found that PDGF signals play important roles in inflammatory lymphangiogenesis in a chronic aseptic peritonitis model. Intraperitoneal administration of imatinib, a potent inhibitor of PDGFRβ, and transduction of PDGFRβ/Fc chimeric protein by an adenoviral system both reduced inflammatory lymphangiogenesis induced by thioglycollate in mice. We also found that the expression of PDGFRβ/Fc reduced tumor lymphangiogenesis in a BxPC3 human pancreatic cancer xenograft model. These findings suggest that PDGFRβ is one of the key mediators of lymphatic vessel formation acting downstream of Prox1. 
 FAK integrates growth-factor and integrin signals to promote cell   migration  . Here we show that cells lacking focal adhesion kinase (FAK) are refractory to motility signals from platelet-derived and epidermal growth factors (PDGF and EGF respectively), and that stable re-expression of FAK rescues these defects. FAK associates with activated PDGF- and EGF-receptor (PDGFR and EGFR) signalling complexes, and expression of the band-4.1-like domain at the FAK amino terminus is sufficient to mediate an interaction with activated EGFR. However, efficient EGF-stimulated cell   migration   also requires FAK to be targeted, by its carboxy-terminal domain, to sites of integrin-receptor clustering. Although the kinase activity of FAK is not needed to promote PDGF- or EGF-stimulated cell motility, kinase-inactive FAK is transphosphorylated at the indispensable Src-kinase-binding site, FAK Y397, after EGF stimulation of cells. Our results establish that FAK is an important receptor-proximal link between growth-factor-receptor and integrin signalling pathways. 
 C-reactive protein and vein graft disease: evidence for a direct effect on smooth muscle cell phenotype via modulation of PDGF receptor-beta. Plasma C-reactive protein (CRP) concentration is a biomarker of systemic atherosclerosis and may also be associated with vein graft disease. It remains unclear whether CRP is also an important modulator of biological events in the vessel wall. We hypothesized that CRP influences vein graft healing by stimulating smooth muscle cells (SMCs) to undergo a phenotypic switch. Distribution of CRP was examined by immunohistochemistry in prebypass human saphenous veins (HSVs, n = 21) and failing vein grafts (n = 18, 25-4,400 days postoperatively). Quiescent HSV SMCs were stimulated with human CRP (5-50 microg/ml). SMC   migration   was assessed in modified Boyden chambers with platelet-derived growth factor (PDGF)-BB (5-10 ng/ml) as the chemoattractant. SMC viability and proliferation were assessed by trypan blue exclusion and reduction of Alamar Blue substrate, respectively. Expression of PDGF ligand and receptor (PDGFR) genes was examined at RNA and protein levels after 24-72 h of CRP exposure. CRP staining was present in 13 of 18 diseased vein grafts, where it localized to the deep media and adventitia, but it was minimally detectable in most prebypass veins. SMCs pretreated with CRP demonstrated a dose-dependent increase in   migration   to PDGF-BB (P = 0.02), which was inhibited by a PDGF-neutralizing antibody. SMCs treated with CRP showed a dose-dependent increase in PDGFRbeta expression and phosphorylation after 24-48 h. Exogenous CRP had no effect on SMC viability or proliferation. These data suggest that CRP is detectable within the wall of most diseased vein grafts, where it may exert local effects. Clinically relevant levels of CRP can stimulate SMC   migration   by a mechanism that may involve upregulation and activation of PDGFRbeta. 
 Site-selective regulation of platelet-derived growth factor beta receptor tyrosine phosphorylation by T-cell protein tyrosine phosphatase. The platelet-derived growth factor (PDGF) beta receptor mediates mitogenic and chemotactic signals. Like other tyrosine kinase receptors, the PDGF beta receptor is negatively regulated by protein tyrosine phosphatases (PTPs). To explore whether T-cell PTP (TC-PTP) negatively regulates the PDGF beta receptor, we compared PDGF beta receptor tyrosine phosphorylation in wild-type and TC-PTP knockout (ko) mouse embryos. PDGF beta receptors were hyperphosphorylated in TC-PTP ko embryos. Fivefold-higher ligand-induced receptor phosphorylation was observed in TC-PTP ko mouse embryo fibroblasts (MEFs) as well. Reexpression of TC-PTP partly abolished this difference. As determined with site-specific phosphotyrosine antibodies, the extent of hyperphosphorylation varied among different autophosphorylation sites. The phospholipase Cgamma1 binding site Y1021, previously implicated in chemotaxis, displayed the largest increase in phosphorylation. The increase in Y1021 phosphorylation was accompanied by increased phospholipase Cgamma1 activity and migratory hyperresponsiveness to PDGF. PDGF beta receptor tyrosine phosphorylation in PTP-1B ko MEFs but not in PTPepsilon ko MEFs was also higher than that in control cells. This increase occurred with a site distribution different from that seen after TC-PTP depletion. PDGF-induced   migration   was not increased in PTP-1B ko cells. In summary, our findings identify TC-PTP as a previously unrecognized negative regulator of PDGF beta receptor signaling and support the general notion that PTPs display site selectivity in their action on tyrosine kinase receptors. 
 Role of platelet-derived growth factor/platelet-derived growth factor receptor axis in the trafficking of circulating fibrocytes in pulmonary fibrosis. Circulating fibrocytes have been reported to migrate into the injured lungs, and contribute to fibrogenesis via CXCL12-CXCR4 axis. In contrast, we report that imatinib mesylate prevented bleomycin (BLM)-induced pulmonary fibrosis in mice by inhibiting platelet-derived growth factor receptor (PDGFR), even when it was administered only in the early phase. The goal of this study was to test the hypothesis that platelet-derived growth factor (PDGF) might directly contribute to the   migration   of fibrocytes to the injured lungs. PDGFR expression in fibrocytes was examined by flow cytometry and RT-PCR. The   migration   of fibrocytes was evaluated by using a chemotaxis assay for human fibrocytes isolated from peripheral blood. The numbers of fibrocytes triple-stained for CD45, collagen-1, and CXCR4 were also examined in lung digests of BLM-treated mice. PDGFR mRNA levels in fibrocytes isolated from patients with idiopathic pulmonary fibrosis were investigated by real-time PCR. Fibrocytes expressed both PDGFR-α and -β, and migrated in response to PDGFs. PDGFR inhibitors (imatinib, PDGFR-blocking antibodies) suppressed fibrocyt  e migrati  on in vitro, and reduced the number of fibrocytes in the lungs of BLM-treated mice. PDGF-BB was a stronger chemoattractant than the other PDGFs in vitro, and anti-PDGFR-β-blocking antibody decreased the numbers of fibrocytes in the lungs compared with anti-PDGFR-α antibody in vivo. Marked expression of PDGFR-β was observed in fibrocytes from patients with idiopathic pulmonary fibrosis compared with healthy subjects. These results suggest that PDGF directly functions as a strong chemoattractant for fibrocytes. In particular, the PDGF-BB-PDGFR-β biological axis might play a critical role in fibr  ocyte mig  ration into the fibrotic lungs. 
 Differential tumorigenic potential and matriptase activation between PDGF B versus PDGF D in prostate cancer. The platelet-derived growth factors (PDGF A, B, C, and D) and their receptors (α-PDGFR and β-PDGFR) play an indispensible role in physiologic and pathologic conditions, including tumorigenesis. The transformative β-PDGFR is overexpressed and activated during prostate cancer progression, but the identification and functional significance of its complementary ligand have not been elucidated. This study examined potential oncogenic functions of β-PDGFR ligands PDGF B and PDGF D, using nonmalignant prostate epithelial cells engineered to overexpress these ligands. In our models, PDGF D induced c  ell migra  tion and invasion more effectively than PDGF B in vitro. Importantly, PDGF D supported prostate epithelial cell tumorigenesis in vivo and showed increased tumor angiogenesis compared with PDGF B. Autocrine signaling analysis of the mitogen-activated protein kinase and phosphoinositide 3-kinase pathways found PDGF D-specific activation of the c-jun-NH2-kinase (JNK) signaling cascade. Using short hairpin RNA and pharmacologic inhibitors, we showed that PDGFD-mediated phenotypic transformation is β-PDGFR and JNK dependent. Importantly, we made a novel finding of PDGF D-specific increase in the shedding and activation of the serine protease matriptase in prostate epithelial cells. Our study, for the first time to our knowledge, showed ligand-specific β-PDGFR signaling as well as PDGF D-specific regulation of matriptase activity and its spatial distribution through shedding. Taken together with our previous finding that matriptase is a proteolytic activator of PDGF D, this study provides a molecular insight into signal amplification of the proteolytic network and PDGF signaling loop during cancer progression. 
 Tissue transglutaminase promotes PDGF/PDGFR-mediated signaling and responses in vascular smooth muscle cells. Although the pivotal role of platelet derived growth factor (PDGF)-mediated signaling in vascular diseases was demonstrated, the pathophysiological mechanisms driving its over-activation remain incompletely understood. Tissue transglutaminase (tTG) is a multifunctional protein expressed in the vasculature, including smooth muscle cells (SMCs), and implicated in several vascular pathologies. The goal of this study is to define the regulation of PDGF-BB/PDGFRβ-induced signaling pathways and cell responses by tTG in vascular SMCs. We find that in human aortic SMCs, shRNA-mediated depletion and over-expression of tTG reveals its ability to down-regulate PDGFRβ levels and induce receptor clustering. In these cells, tTG specifically amplifies the activation of PDGFRβ and its multiple downstream signaling targets in response to PDGF-BB. Furthermore, tTG promotes dedifferentiation and increases survival, proliferation, a  nd migrat  ion of human aortic SMCs mediated by this growth factor. Finally, PDGF-BB stimulates tTG expression in human aortic SMCs in culture and in the blood vessels in response to injury. Together, our results show that tTG in vascular SMCs acts as a principal enhancer within the PDGF-BB/PDGFRβ signaling axis involved in phenotypic modulation of these cells, thereby suggesting a novel role for this protein in the progression of vascular diseases. 
 P-Rex1 cooperates with PDGFRβ to drive cellular   migratio  n in 3D microenvironments. Expression of the Rac-guanine nucleotide exchange factor (RacGEF), P-Rex1 is a key determinant of progression to metastasis in a number of human cancers. In accordance with this proposed role in cancer cell invasion and metastasis, we find that ectopic expression of P-Rex1 in an immortalised human fibroblast cell line is sufficient to drive multiple migratory and invasive phenotypes. The invasive phenotype is greatly enhanced by the presence of a gradient of serum or platelet-derived growth factor, and is dependent upon the expression of functional PDGF receptor β. Consistently, the invasiveness of WM852 melanoma cells, which endogenously express P-Rex1 and PDGFRβ, is opposed by siRNA of either of these proteins. Furthermore, the current model of P-Rex1 activation is advanced through demonstration of P-Rex1 and PDGFRβ as components of the same macromolecular complex. These data suggest that P-Rex1 has an influence on physiological migratory processes, such as invasion of cancer cells, both through effects upon classical Rac1-driven motility and a novel association with RTK signalling complexes. 
 Differential effects of red and white wines on inhibition of the platelet-derived growth factor receptor: impact of the mash fermentation. Moderate wine consumption is associated with a significant reduction of cardiovascular mortality. The molecular basis of this phenomenon remains unknown. Platelet-derived growth factor (PDGF) is an important contributor to atherogenesis. We investigated the effects of selected red and white wines on PDGF receptor (PDGFR) signalling in rat and human vascular smooth muscle cells (VSMCs).All red wines concentration dependently inhibited the ligand-induced tyrosine phosphorylation of the PDGFR, downstream signalling events such as mitogen activated protein (MAP) kinase activation (Erk 1/2) and induction of immediate early genes (Egr-1, c-fos), and PDGF-induced cellular responses, whereas all white wines had no effect. At concentrations achieved after wine consumption in humans, all red wines completely abolished PDGF-dependent VSMC proliferation and   migration  . Red wines also inhibited PDGFR phosphorylation in vascular tissue, and in human coronary smooth muscle cells. Quantitative analyses of all tested wines and of samples collected at various time points (Days 0-16) of the 'mash fermentation', which is only performed for red wine, revealed that flavonoids of the catechin family, which potently inhibit PDGFR signalling, are extracted from grape seeds and skins during this process and therefore accumulate specifically in red wine. The accumulation of flavonoids correlated with the inhibitory potency of red wines on PDGFR signalling. Furthermore, this procedure could be imitated by incubation of wines with shredded grape seeds, and flavonoid-enriched white wine inhibited the PDGFR as potently as red wines.Only red wines abrogate a critical pathogenic mechanism during atherogenesis, PDGFR signalling, in VSMCs. This effect is mediated by non-alcoholic constituents, which accumulate during the mash fermentation. Our findings offer a molecular explanation for the vasoprotective effects particularly of red wine. Therefore, future epidemiological studies should consider differential protective effects of red and white wine in vivo. 
 Beta-platelet-derived growth factor receptor mediates motility and growth of Ewing's sarcoma cells. The Ewing's sarcoma family of tumors (ESFT) contain a translocation, t(11;22), which results in the novel oncogenic fusion protein EWS/FLI1. Platelet-derived growth factors (PDGF) and their receptors (PDGFR) are involved in the induction and proliferation of numerous solid tumors and are the potential candidates for novel targeted antitumor therapy. Since a relation was reported between PDGF-C and EWS/FLI1, we sought to characterize the PDGF signaling pathway in ESFT. Eight out of nine ESFT cell lines were found to express significant levels of beta-PDGFR. Interestingly, none of the tested cell lines expressed alpha-PDGFR, which is the receptor isotype required for PDGF-C binding. By immunohistochemical staining 47 of 52 (90.4%) archival tumor samples from patients with ESFT were positive for beta-PDGFR. ESFT cell lines were treated with PDGF-AA or PDGF-BB ligands to evaluate downstream signaling. Autophosphorylation of beta-PDGFR and tyrosine phosphorylation of PLC-gamma, PI3Kp85 and Shc were detected only in PDGF-BB-stimulated cells that express beta-PDGFR. Receptor function was further evaluated using chemotaxis assays that showed TC-32 cell   migration   towards PDGF-BB. A specific PDGFR kinase inhibitor AG1295 blocked beta-PDGFR activation, downstream signaling, growth in cell culture and chemotaxis of TC-32 cells. AG1295 also delayed tumor formation and prolonged survival in an ESFT animal model. We conclude that ESFT express beta-PDGFR and that this is a functional and potentially crucial signaling pathway. Therefore, beta-PDGFRs may provide a novel therapeutic target in ESFT that can be utilized to design better treatment modalities. 
 Radiation-induced senescence in securin-deficient cancer cells promotes cell invasion involving the IL-6/STAT3 and PDGF-BB/PDGFR pathways. Securin overexpression correlates with poor prognosis in various tumours. We have previously shown that securin depletion promotes radiation-induced senescence and enhances radiosensitivity in human cancer cells. However, the underlying molecular mechanisms and the paracrine effects remain unknown. In this study, we showed that radiation induced senescence in securin-deficient human breast cancer cells involving the ATM/Chk2 and p38 pathways. Conditioned medium (CM) from senescent cells promoted the invasion and   migration   of non-irradiated cancer and endothelial cells. Cytokine assay analysis showed the up-regulation of various senescence-associated secretory phenotypes (SASPs). The IL-6/STAT3 signalling loop and platelet-derived growth factor-BB (PDGF-BB)/PDGF receptor (PDGFR) pathway were important for CM-induced cell   migration   and invasion. Furthermore, CM promoted angiogenesis in the chicken chorioallantoic membrane though the induction of IL-6/STAT3- and PDGF-BB/PDGFR-dependent endothelial cell invasion. Taken together, our results provide the molecular mechanisms for radiation-induced senescence in securin-deficient human breast cancer cells and for the SASP responses. 
 Platelet-derived growth factor receptor beta and vascular endothelial growth factor receptor 2 bind to the beta 3 integrin through its extracellular domain. Integrin-mediated cell attachment and growth factor stimulation often act synergistically on cell proliferation, differentiation,   migration  , and survival. Some of these synergistic effects depend on the physical interaction of integrins with growth factor receptors. Here we examine the nature of the physical interaction between the alpha(v)beta(3) integrin and two receptor tyrosine kinases (RTKs), the platelet-derived growth factor receptor beta (PDGF-Rbeta) and the vascular endothelial growth factor receptor 2 (VEGF-R2, also known as KDR and flk-1). Both of these RTKs associate with the alpha(v)beta(3) integrin but do not associate with beta(1) integrins. Furthermore, growth factor stimulation of these RTKs promotes increased cell proliferation and   migration   when cells are attached to the alpha(v)beta(3) ligand, vitronectin. We show that alpha(v)beta(3) in which the beta(3) cytoplasmic domain is deleted or replaced with the beta(1) cytoplasmic domain coimmunoprecipitates with PDGF-Rbeta and VEGF-R2. The beta(3) extracellular domain alone was sufficient for the PDGF-Rbeta association whereas the VEGF-R2 association required the presence of the alpha(v) subunit. Activation of the RTKs by their ligands was not required for them to associate with the integrin. Cell   migration   to PDGF was enhanced in the cells transfected with the chimeric subunit containing the beta(3) extracellular domain but not when that domain came from the beta(1) subunit. These results show that the interactions that lead to the association of the alpha(v)beta(3) integrin with PDGF-Rbeta and VEGF-R2 and enhancement of RTK activity take place outside the cell. 
 Disruption of platelet-derived growth factor-dependent phosphatidylinositol 3-kinase and phospholipase Cγ 1 activity abolishes vascular smooth muscle cell proliferation and   migratio  n and attenuates neointima formation in vivo. We tested the hypothesis whether selective blunting of platelet-derived growth factor (PDGF)-dependent vascular smooth muscle cell (VSMC) proliferation and   migratio  n is sufficient to prevent neointima formation after vascular injury.To prevent neointima formation and stent thrombosis after coronary interventions, it is essential to inhibit VSMC proliferation and   migratio  n without harming endothelial cell function. The role of PDGF-a potent mitogen and chemoattractant for VSMC that does not affect endothelial cells-for neointima formation remains controversial.To decipher the signaling pathways that control PDGF beta receptor (βPDGFR)-driven VSMC proliferation an  d migrati  on, we characterized 2 panels of chimeric CSF1R/βPDGFR mutants in which the binding sites for βPDGFR-associated signaling molecules (Src, phosphatidylinositol 3-kinase [PI3K], GTPase activating protein of ras, SHP-2, phospholipase Cγ 1 [PLCγ]) were individually mutated. Based on in vitro results, the importance of PDGF-initiated signals for neointima formation was investigated in genetically modified mice.Our results indicate that the chemotactic response to PDGF requires the activation of Src, PI3K, and PLCγ, whereas PDGF-dependent cell cycle progression is exclusively mediated by PI3K and PLCγ. These 2 signaling molecules contribute to signal relay of the βPDGFR by differentially regulating cyclin D1 and p27(kip1). Blunting of βPDGFR-induced PI3K and PLCγ signaling by a combination mutant (F3) completely abolished the mitogenic and chemotactic response to PDGF. Disruption of PDGF-dependent PI3K and PLCγ signaling in mice expressing the F3 receptor led to a profound reduction of neointima formation after balloon injury.Signaling by the activated βPDGFR, particularly through PI3K and PLCγ, is crucial for neointima formation after vascular injury. Disruption of these specific signaling pathways is sufficient to attenuate pathogenic processes such as vascular remodeling in vivo. 
 PDGFRβ Is a Novel Marker of Stromal Activation in Oral Squamous Cell Carcinomas. Carcinoma associated fibroblasts (CAFs) form the main constituents of tumor stroma and play an important role in tumor growth and invasion. The presence of CAFs is a strong predictor of poor prognosis of head and neck squamous cell carcinoma. Despite significant progress in determining the role of CAFs in tumor progression, the mechanisms contributing to their activation remain poorly characterized, in part due to fibroblast heterogeneity and the scarcity of reliable fibroblast surface markers. To search for such markers in oral squamous cell carcinoma (OSCC), we applied a novel approach that uses RNA-sequencing data derived from the cancer genome atlas (TCGA). Specifically, our strategy allowed for an unbiased identification of genes whose expression was closely associated with a set of bona fide stroma-specific transcripts, namely the interstitial collagens COL1A1, COL1A2, and COL3A1. Among the top hits were genes involved in cellular matrix remodeling and tumor invasion and   migratio  n, including platelet-derived growth factor receptor beta (PDGFRβ), which was found to be the highest-ranking receptor protein genome-wide. Similar analyses performed on ten additional TCGA cancer datasets revealed that other tumor types shared CAF markers with OSCC, including PDGFRβ, which was found to significantly correlate with the reference collagen expression in ten of the 11 cancer types tested. Subsequent immunostaining of OSCC specimens demonstrated that PDGFRβ was abundantly expressed in stromal fibroblasts of all tested cases (12/12), while it was absent in tumor cells, with greater specificity than other known markers such as alpha smooth muscle actin or podoplanin (3/11). Overall, this study identified PDGFRβ as a novel marker of stromal activation in OSCC, and further characterized a list of promising candidate CAF markers that may be relevant to other carcinomas. Our novel approach provides for a fast and accurate method to identify CAF markers without the need for large-scale immunostaining experiments. 
 Inhibition of receptor signaling and of glioblastoma-derived tumor growth by a novel PDGFRβ aptamer. Platelet-derived growth factor receptor β (PDGFRβ) is a cell-surface tyrosine kinase receptor implicated in several cellular processes including proliferatio  n, migrat  ion, and angiogenesis. It represents a compelling therapeutic target in many human tumors, including glioma. A number of tyrosine kinase inhibitors under development as antitumor agents have been found to inhibit PDGFRβ. However, they are not selective as they present multiple tyrosine kinase targets. Here, we report a novel PDGFRβ-specific antagonist represented by a nuclease-resistant RNA-aptamer, named Gint4.T. This aptamer is able to specifically bind to the human PDGFRβ ectodomain (Kd: 9.6 nmol/l) causing a strong inhibition of ligand-dependent receptor activation and of downstream signaling in cell lines and primary cultures of human glioblastoma cells. Moreover, Gint4.T aptamer drastically inhibi  ts cell m  igration and proliferation, induces differentiation, and blocks tumor growth in vivo. In addition, Gint4.T aptamer prevents PDGFRβ heterodimerization with and resultant transactivation of epidermal growth factor receptor. As a result, the combination of Gint4.T and an epidermal growth factor receptor-targeted aptamer is better at slowing tumor growth than either single aptamer alone. These findings reveal Gint4.T as a PDGFRβ-drug candidate with translational potential. 
 Validation of PDGFRbeta and c-Src tyrosine kinases as tumor/vessel targets in patients with multiple myeloma: preclinical efficacy of the novel, orally available inhibitor dasatinib. Inhibition of multiple myeloma (MM) plasma cells in their permissive bone marrow microenvironment represents an attractive strategy for blocking the tumor/vessel growth associated with the disease progression. However, target specificity is an essential aim of this approach. Here, we identified platelet-derived growth factor (PDGF)-receptor beta (PDGFRbeta) and pp60c-Src as shared constitutively activated tyrosine-kinases (TKs) in plasma cells and endothelial cells (ECs) isolated from MM patients (MMECs). Our cellular and molecular dissection showed that the PDGF-BB/PDGFRbeta kinase axis promoted MM tumor growth and vessel sprouting by activating ERK1/2, AKT, and the transcription of MMEC-released proangiogenic factors, such as vascular endothelial growth factor (VEGF) and interleukin-8 (IL-8). Interestingly, pp60c-Src TK-activity was selectively induced by VEGF in MM tumor and ECs, and the use of small-interfering (si)RNAs validated pp60c-Src as a key signaling effector of VEGF loop required for MMEC survival,   migration  , and angiogenesis. We also assessed the antitumor/vessel activity of dasatinib, a novel orally bioactive PDGFRbeta/Src TK-inhibitor that significantly delayed MM tumor growth and angiogenesis in vivo, showing a synergistic cytotoxicity with conventional and novel antimyeloma drugs (ie, melphalan, prednisone, bor-tezomib, and thalidomide). Overall data highlight the biologic and therapeutic relevance of the combined targeting of PDGFRbeta/c-Src TKs in MM, providing a framework for future clinical trials. 
 Primary familial brain calcification: update on molecular genetics. Primary familial brain calcification is a neuropsychiatric disorder with calcium deposits in the brain, especially in basal ganglia, cerebellum and subcortical white matter. The disease is characterized by a clinical heterogeneity, with a various combination of symptoms that include movement disorders and psychiatric disturbances; asymptomatic patients have been also reported. To date, three causative genes have been found: SLC20A2, PDGFRB and PDGFB. SLC20A2 gene codes for the 'sodium-dependent phosphate transporter 2' (PiT-2), a cell membrane transporters of inorganic phosphate, involved in Pi uptake by cells and maintenance of Pi body levels. Over 40 pathogenic variants of SLC20A2 have been reported, affecting the regulation of Pi homeostasis. It was hypothesized that SLC20A2 mutations cause brain calcification most likely through haploinsufficiency. PDGFRB encodes for the platelet-derived growth factor receptor-β (PDGFRβ), a cell-surface tyrosine-kinase (RTK) receptor that regulates cell proliferation  , migrati  on, survival and differentiation. PDGFB encodes for the 'platelet-derived growth factor beta' (PDGFβ), the ligand of PDGFRβ. The loss of function of PDGFRβ and PDGFβ could lead to the impairment of the pericytes function and blood brain barrier integrity, causing vascular and perivascular calcium accumulation. SLC20A2 accounts for about 40 % of familial form and 14 % of sporadic cases, while PDGFRB and PDGFB mutations are likely rare. However, approximately 50 % of patients are not genetically defined and there should be at least another causative gene. 
 XBP 1-Deficiency Abrogates Neointimal Lesion of Injured Vessels Via Cross Talk With the PDGF Signaling. Smooth muscle cell (SMC)   migration   and proliferation play an essential role in neointimal formation after vascular injury. In this study, we intended to investigate whether the X-box-binding protein 1 (XBP1) was involved in these processes.In vivo studies on femoral artery injury models revealed that vascular injury triggered an immediate upregulation of XBP1 expression and splicing in vascular SMCs and that XBP1 deficiency in SMCs significantly abrogated neointimal formation in the injured vessels. In vitro studies indicated that platelet-derived growth factor-BB triggered XBP1 splicing in SMCs via the interaction between platelet-derived growth factor receptor β and the inositol-requiring enzyme 1α. The spliced XBP1 (XBP1s) increased SM  C migrati  on via PI3K/Akt activation and proliferation via downregulating calponin h1 (CNN1). XBP1s directed the transcription of mir-1274B that targeted CNN1 mRNA degradation. Proteomic analysis of culture media revealed that XBP1s decreased transforming growth factor (TGF)-β family proteins secretion via transcriptional suppression. TGF-β3 but not TGF-β1 or TGF-β2 attenuated XBP1s-induced CNN1 decrease and SMC proliferation.This study demonstrates for the first time that XBP1 is crucial for SMC proliferation via modulating the platelet-derived growth factor/TGF-β pathways, leading to neointimal formation. 
 Binding of Cbl to a phospholipase Cgamma1-docking site on platelet-derived growth factor receptor beta provides a dual mechanism of negative regulation. Ubiquitin conjugation to receptor tyrosine kinases is a critical biochemical step in attenuating their signaling through lysosomal degradation. Our previous studies have established Cbl as an E3 ubiquitin ligase for ubiquitinylation and degradation of platelet-derived growth factor receptor (PDGFR) alpha and PDGFRbeta. However, the role of endogenous Cbl in PDGFR regulation and the molecular mechanisms of this regulation remain unclear. Here, we demonstrate that endogenous Cbl is essential for ligand-induced ubiquitinylation and degradation of PDGFRbeta; this involves the Cbl TKB domain binding to PDGFRbeta phosphotyrosine 1021, a known phospholipase C (PLC) gamma1 SH2 domain-binding site. Lack of Cbl or ablation of the Cbl-binding site on PDGFRbeta impedes receptor sorting to the lysosome. Cbl-deficient cells also show more PDGF-induced PLCgamma1 association with PDGFRbeta and enhanced PLC-mediated cell   migration  . Thus, Cbl-dependent negative regulation of PDGFRbeta involves a dual mechanism that concurrently promotes ubiquitin-dependent lysosomal sorting of the receptor and competitively reduces the recruitment of a positive mediator of receptor signaling. 
 Endothelial and smooth muscle-derived neuropilin-like protein regulates platelet-derived growth factor signaling in human vascular smooth muscle cells by modulating receptor ubiquitination. Endothelial and smooth muscle cell-derived neuropilin-like protein (ESDN) is up-regulated in the neointima of remodeling arteries and modulates vascular smooth muscle cell (VSMC) growth. Platelet-derived growth factor (PDGF) is the prototypic growth factor for VSMCs and plays a key role in vascular remodeling. Here, we sought to further define ESDN function in primary human VSMCs. ESDN down-regulation by RNA interference significantly enhanced PDGF-induced VSMC DNA synthesis and   migration  . This was associated with increased ERK1/2, Src, and PDGF receptor (PDGFR)beta phosphorylation, without altering total PDGFRbeta expression levels. In binding assays, ESDN down-regulation significantly increased (125)I-PDGF maximum binding (B(max)) to PDGF receptors on VSMCs without altering the binding constant (K(d)), raising the possibility that ESDN regulates PDGFR processing. ESDN down-regulation significantly reduced ligand-induced PDGFRbeta ubiquitination. This was associated with a significant reduction in the expression level of c-Cbl, an E3 ubiquitin ligase that ubiquitinylates PDGFRbeta. Thus, ESDN modulates PDGF signaling in VSMCs via regulation of PDGFR surface levels. The ESDN effect is mediated, at least in part, through effects on PDGFRbeta ubiquitination. ESDN may serve as a target for regulating PDGFRbeta signaling in VSMCs. 
 The Involvement of PDGF-B/PDGFRβ Axis in the Resistance to Antiangiogenic and Antivascular Therapy in Renal Cancer. Studies developed in the field of platelet-derived growth factors/platelet-derived growth factor receptors (PDGFs/PDGFRs) inhibition have focused on the therapeutic effects on tumor cells, neglecting their potential effects on tumor blood vessels. We herein propose a differential and critic assessment of platelet-derived growth factor B (PDGF-B) and platelet-derived growth factor receptor β (PDGFRβ) in renal cell carcinoma, correlated with the four main vascular patterns previously reported by our team.PDGF-B and PDGFRβ were evaluated on 50 archival paraffin embedded specimens related to vascular endothelial growth factor (VEGF), its inhibitory isoform VEGF165b and vascular patterns.Our results support the involvement of VEGF165b in the phosphorylation of PDGFRβ with an inhibitory effect on endothelial proliferation   and migr  ation. The simultaneous action of PDGF-B/PDGFRβ and VEGF165b on the same type of receptor may explain the resistance to antiangiogenic therapy, which depends on the degree of modulation of PDGFRβ phosphorylation. 
 Altered   migration   and adhesion potential of pro-neurally converted human bone marrow stromal cells. Bone marrow (BM)-derived mesenchymal stromal cells (MSC) are promising candidate cells for the development of neuroregenerative therapies. We have previously introduced the pro-neural conversion of human MSC to neural stem cell-like cells (m-NSC) by culturing them in suspension culture under serum-free conditions.In the present study, we used a modified Boyden chamber assay to study the influence of various chemoattractants and extracellular matrix components on MSC and m-NSC   migration   in vitro. The underlying mechanisms were investigated further by applying real-time reverse transcriptase (RT)-polymerase chain reaction (PCR) and flow cytometry.The basal   migration   of m-NSC was significantly reduced compared with MSC (six versus 27 out of 10,000 cells migrated within 6 h). We evaluated the effects of bone morphogenic protein 2 (BMP2), insulin-like growth factor 1 (IGF1), platelet-derived growth factor bb (PDGFbb), vascular endothelial growth factor (VEGFa), and stromal cell-derived factor 1 (SDF1) on the   migration   potential of both cell types and PDGFbb proved to be the most potent stimulant of   migration   (235 versus 198 m-NSC or MSC migrated). Adhesion of m-NSC to the filter membrane was delayed and not affected by IGF1 or PDGFbb: 90% of MSC, but only 20% of m-NSC, adhered within 1 h, with 90% of m-NSC adhering within 3 h. However, real-time RT-PCR and flow cytometry revealed an up-regulation of the PDGF receptor B following conversion. Coating the membranes with collagen type I or hyaluronan also significantly influenced cell   migration  .We could identify major chemoattractive factors for m-NSC and gained partial insight into the complex processes involved in   migration   of neurally converted cells. 
 TF/FVIIa transactivate PDGFRbeta to regulate PDGF-BB-induced chemotaxis in different cell types: involvement of Src and PLC. We have previously reported the potentiation of PDGF-BB-induced chemotaxis of fibroblasts, vascular smooth muscle cells, and endothelial cells by FVIIa. Here we studied the role of TF/FVIIa and the induced signaling pathways in regulation of chemotaxis of human monocytes, fibroblasts, and porcine aorta endothelial cells.Human monocytes were obtained by using Ficoll-Paque gradient and the MACS system (for highly purified population), fibroblasts and PAE cells have been characterized previously. Inhibitors of selected signaling intermediates were used, and the effect of TF/FVIIa on the migratory response of all cells to chemotactic agents was analyzed. The induced signaling was studied by immunoprecipitation and Western blotting. TF/FVIIa complex selectively enhanced PDGF-BB-induced chemotaxis in a Src-family, PLC, and PAR-2-dependent manner. Using PAE cells we identified c-Src and c-Yes as the Src-family members activated by TF/FVIIa. We report for the first time the PAR-2 and Src family-dependent transactivation of PDGFRbeta by TF/FVIIa involving phosphorylation of a subset of PDGFRbeta tyrosines.The described transactivation is a likely mechanism of TF/FVIIa-mediated regulation of PDGF-BB-induced chemotaxis. Similar behavior of 3 principally different cell types in our experimental setup may reflect a general function of TF in regulation of cell   migration  . 
 The Telomeric Protein TRF2 Regulates Angiogenesis by Binding and Activating the PDGFRβ Promoter. Telomeric repeat binding factor 2 (TRF2), which plays a central role in telomere capping, is frequently increased in human tumors. We reveal here that TRF2 is expressed in the vasculature of most human cancer types, where it colocalizes with the Wilms' tumor suppressor WT1. We further show that TRF2 is a transcriptional target of WT1 and is required for proliferation,   migratio  n, and tube formation of endothelial cells. These angiogenic effects of TRF2 are uncoupled from its function in telomere capping. Instead, TRF2 binds and transactivates the promoter of the angiogenic tyrosine kinase platelet-derived growth factor receptor β (PDGFRβ). These findings reveal an unexpected role of TRF2 in neoangiogenesis and delineate a distinct function of TRF2 as a transcriptional regulator. 
 Dysfunctional, pro-inflammatory HDL directly upregulates monocyte PDGFRβ, chemotaxis and TNFα production. Accelerated atherosclerosis is a major co-morbid condition in autoimmune diseases. Monocytes are the main immune cell involved in atherosclerosis initiation. We hypothesized that dysfunctional, pro-inflammatory HDL (piHDL), which occurs in approximately half of SLE patients, might directly influence monocyte gene expression and function. SLE subjects were stratified into three groups: 1) carotid artery plaque+piHDL+,2) plaque-piHDL+,and 3) plaque-piHDL- (n=18/group). PDGFRβ was upregulated in primary monocytes from plaque+piHDL+patients and in THP-1 cells acutely treated in vitro with piHDL compared to normal HDL. THP-1 chemotaxis was enhanced after treatment with piHDL versus normal HDL. Abnorm  al migrat  ion was restored to normal levels by treatment with imatinib or an apoJ mimetic peptide. Increased piHDL-mediated TNFα protein levels were reduced with both inhibitors. Dysfunctional piHDL directly influences expression of a small number of transcripts and proteins, and piHDL inhibition through reducing piHDL oxidation or blocking PDGFRβ kinase activity restored normal monocyte chemotaxis. 
  GeneRIFs  
 The IL-6/STAT3 signalling loop and platelet-derived growth factor-BB (PDGF-BB)/PDGF receptor (PDGFR) pathway were important for CM-induced cell   migration   and invasion. 
 Data show that knock-down of PDGFR-beta by siRNA resulted in a significant inhibition of cell   migration   while treatment with scrambled siRNA sequences had no effect. 
 Mesenchymal stem cell   migration   is regulated by fibronectin through alpha5beta1-integrin-mediated activation of PDGFR-beta and potentiation of PDGF-BB signals. 
 C-reactive protein can stimulate smooth muscle cell   migration   by a mechanism that may involve upregulation and activation of platelet-derived growth factor receptor beta 
  ECM  PubMed  
 Mesenchymal stem cell migration is regulated by fibronectin through α5β1-integrin-mediated activation of PDGFR-β and potentiation of growth factor signals. Cell migration during vascular remodelling is regulated by crosstalk between growth factor receptors and integrin receptors, which together coordinate cytoskeletal and motogenic changes. Here, we report extracellular matri  x (  ECM)-directed crosstalk between platelet-derived growth factor receptor (PDGFR)-β and α5β1-integrin, which controls the migration of mesenchymal stem (stromal) cells (MSCs). Cell adhesion to fibronectin induced α5β1-integrin-dependent phosphorylation of PDGFR-β in the absence of growth factor stimulation. Phosphorylated PDGFR-β co-immunoprecipitated with α5-integrin and colocalised with α5β1-integrin in the transient tidemarks of focal adhesions. Adhesion to fibronectin also strongly potentiated PDGF-BB-induced PDGFR-β phosphorylation and focal adhesion kinase (FAK) activity, in an α5β1-integrin-dependent manner. PDGFR-β-induced phosphoinositide 3-kinase (PI3K) and Akt activity, actin reorganisation and cell migration were all regulated by fibronectin and α5β1-integrin. This synergistic relationship between α5β1-integrin and PDGFR-β is a fundamental determinant of cell migration. Thus, fibronectin-rich matrices can prime PDGFR-β to recruit mesenchymal cells at sites of vascular remodelling. 
 Sphingosine-1-phosphate induces a PDGFR-dependent cell detachment via inhibiting beta1 integrin in HEK293 cells. Several different types of interactions between sphingosine-1-phosphate (S1P) receptors and platelet-derived growth factor receptor (PDGFR) have been revealed recently. In this work, we used HEK293 cells to further investigate the potential crosstalk. Interestingly, we observed that S1P specifically induced a PDGFR-dependent cell detachment in HEK293 cells, which could be inhibited by AG1296, a specific inhibitor for PDGFR. EGFR on the other hand, did not have any effect on cell detachment. The detachment was extracellular matrix (  ECM  ) protein specific, suggesting the involvement of specific integrin molecules. When beta(1) integrin was engaged into an active state, S1P-induced cell detachment was blocked, suggesting that S1P induced an inside-out inhibitory effect on beta(1) integrin. G(i) protein and ERK activation were required for the cell detachment induced by S1P, suggesting an endogenous receptor for S1P is likely to be involved. 
 Hypoxia differentially regulates arterial and venous smooth muscle cell proliferation via PDGFR-β and VEGFR-2 expression. Despite intensive research studies, theories have yet to focus on the contribution of hypoxia to patency differences observed clinically between arterial vs. venous grafts. This study investigates the differential hypoxic response of smooth muscle cells (SMC) to hypoxia-derived endothelial cell (EC) growth factors. Initiation of SMC proliferation under hypoxia (<5% O(2)) occurred only after incubation with hypoxic endothelial cell-conditioned media (H  -EC  M). After the investigation of several possible growth factors in the H  -EC  M that may be responsible for SMC proliferation, the greatest difference was observed in vascular endothelial growth factor (VEGF-A) and platelet-derived growth factor homodimer B (PDGF-BB) expression. VEGF-A increased (2-fold) significantly (P < 0.05) in arterial-derived smooth muscle cells (ASMC) under hypoxia compared with venous-derived smooth muscle cells (VSMC), which showed no significant change. VSMC showed significant (P < 0.05) increase in VEGFR-2 expression under hypoxia compared with ASMC. Incubation with VEGFR-2-neutralizing antibody/PDGFR antagonist in VSMC before addition of H  -EC  M resulted in decreased proliferation. ASMC proliferation under hypoxia did not decrease during incubation with VEGFR-2-neutralizing antibody but did decrease upon PDGFR antagonist incubation. Current therapies focusing on treating intimal hyperplasia have negated the fact that combinational therapy might be required to combat induction of SMC proliferation. Clinically, therapy with PDGFR antagonists plus anti-VEGFR-2 may prove to be efficacious in managing SMC proliferation in venous-derived grafts. 
 Hypoxia differentially regulates arterial and venous smooth muscle cell proliferation via PDGFR-β and VEGFR-2 expression. Despite intensive research studies, theories have yet to focus on the contribution of hypoxia to patency differences observed clinically between arterial vs. venous grafts. This study investigates the differential hypoxic response of smooth muscle cells (SMC) to hypoxia-derived endothelial cell (EC) growth factors. Initiation of SMC proliferation under hypoxia (<5% O(2)) occurred only after incubation with hypoxic endothelial cell-conditioned media (H  -EC  M). After the investigation of several possible growth factors in the H  -EC  M that may be responsible for SMC proliferation, the greatest difference was observed in vascular endothelial growth factor (VEGF-A) and platelet-derived growth factor homodimer B (PDGF-BB) expression. VEGF-A increased (2-fold) significantly (P < 0.05) in arterial-derived smooth muscle cells (ASMC) under hypoxia compared with venous-derived smooth muscle cells (VSMC), which showed no significant change. VSMC showed significant (P < 0.05) increase in VEGFR-2 expression under hypoxia compared with ASMC. Incubation with VEGFR-2-neutralizing antibody/PDGFR antagonist in VSMC before addition of H  -EC  M resulted in decreased proliferation. ASMC proliferation under hypoxia did not decrease during incubation with VEGFR-2-neutralizing antibody but did decrease upon PDGFR antagonist incubation. Current therapies focusing on treating intimal hyperplasia have negated the fact that combinational therapy might be required to combat induction of SMC proliferation. Clinically, therapy with PDGFR antagonists plus anti-VEGFR-2 may prove to be efficacious in managing SMC proliferation in venous-derived grafts. 
 Mesenchymal stem cell migration is regulated by fibronectin through α5β1-integrin-mediated activation of PDGFR-β and potentiation of growth factor signals. Cell migration during vascular remodelling is regulated by crosstalk between growth factor receptors and integrin receptors, which together coordinate cytoskeletal and motogenic changes. Here, we report extracellular matri  x (  ECM)-directed crosstalk between platelet-derived growth factor receptor (PDGFR)-β and α5β1-integrin, which controls the migration of mesenchymal stem (stromal) cells (MSCs). Cell adhesion to fibronectin induced α5β1-integrin-dependent phosphorylation of PDGFR-β in the absence of growth factor stimulation. Phosphorylated PDGFR-β co-immunoprecipitated with α5-integrin and colocalised with α5β1-integrin in the transient tidemarks of focal adhesions. Adhesion to fibronectin also strongly potentiated PDGF-BB-induced PDGFR-β phosphorylation and focal adhesion kinase (FAK) activity, in an α5β1-integrin-dependent manner. PDGFR-β-induced phosphoinositide 3-kinase (PI3K) and Akt activity, actin reorganisation and cell migration were all regulated by fibronectin and α5β1-integrin. This synergistic relationship between α5β1-integrin and PDGFR-β is a fundamental determinant of cell migration. Thus, fibronectin-rich matrices can prime PDGFR-β to recruit mesenchymal cells at sites of vascular remodelling. 
 Sphingosine-1-phosphate induces a PDGFR-dependent cell detachment via inhibiting beta1 integrin in HEK293 cells. Several different types of interactions between sphingosine-1-phosphate (S1P) receptors and platelet-derived growth factor receptor (PDGFR) have been revealed recently. In this work, we used HEK293 cells to further investigate the potential crosstalk. Interestingly, we observed that S1P specifically induced a PDGFR-dependent cell detachment in HEK293 cells, which could be inhibited by AG1296, a specific inhibitor for PDGFR. EGFR on the other hand, did not have any effect on cell detachment. The detachment was extracellular matrix (  ECM  ) protein specific, suggesting the involvement of specific integrin molecules. When beta(1) integrin was engaged into an active state, S1P-induced cell detachment was blocked, suggesting that S1P induced an inside-out inhibitory effect on beta(1) integrin. G(i) protein and ERK activation were required for the cell detachment induced by S1P, suggesting an endogenous receptor for S1P is likely to be involved. 
  [Ee]xtracellular [Mm]atrix  PubMed  
 Mesenchymal stem cell migration is regulated by fibronectin through α5β1-integrin-mediated activation of PDGFR-β and potentiation of growth factor signals. Cell migration during vascular remodelling is regulated by crosstalk between growth factor receptors and integrin receptors, which together coordinate cytoskeletal and motogenic changes. Here, we repo  rt extracellular mat  rix (ECM)-directed crosstalk between platelet-derived growth factor receptor (PDGFR)-β and α5β1-integrin, which controls the migration of mesenchymal stem (stromal) cells (MSCs). Cell adhesion to fibronectin induced α5β1-integrin-dependent phosphorylation of PDGFR-β in the absence of growth factor stimulation. Phosphorylated PDGFR-β co-immunoprecipitated with α5-integrin and colocalised with α5β1-integrin in the transient tidemarks of focal adhesions. Adhesion to fibronectin also strongly potentiated PDGF-BB-induced PDGFR-β phosphorylation and focal adhesion kinase (FAK) activity, in an α5β1-integrin-dependent manner. PDGFR-β-induced phosphoinositide 3-kinase (PI3K) and Akt activity, actin reorganisation and cell migration were all regulated by fibronectin and α5β1-integrin. This synergistic relationship between α5β1-integrin and PDGFR-β is a fundamental determinant of cell migration. Thus, fibronectin-rich matrices can prime PDGFR-β to recruit mesenchymal cells at sites of vascular remodelling. 
 Sphingosine-1-phosphate induces a PDGFR-dependent cell detachment via inhibiting beta1 integrin in HEK293 cells. Several different types of interactions between sphingosine-1-phosphate (S1P) receptors and platelet-derived growth factor receptor (PDGFR) have been revealed recently. In this work, we used HEK293 cells to further investigate the potential crosstalk. Interestingly, we observed that S1P specifically induced a PDGFR-dependent cell detachment in HEK293 cells, which could be inhibited by AG1296, a specific inhibitor for PDGFR. EGFR on the other hand, did not have any effect on cell detachment. The detachment was   extracellular matrix   (ECM) protein specific, suggesting the involvement of specific integrin molecules. When beta(1) integrin was engaged into an active state, S1P-induced cell detachment was blocked, suggesting that S1P induced an inside-out inhibitory effect on beta(1) integrin. G(i) protein and ERK activation were required for the cell detachment induced by S1P, suggesting an endogenous receptor for S1P is likely to be involved. 
 A new look at platelet-derived growth factor in renal disease. The PDGF system, comprising four isoforms (PDGF-A, -B, -C, and -D) and two receptor chains (PDGFR-alpha and -beta), plays important roles in wound healing, atherosclerosis, fibrosis, and malignancy. Components of the system are expressed constitutively or inducibly in most renal cells. They regulate a multitude of pathophysiologic events, ranging from cell proliferation and migration to   extracellular matrix   accumulation, production of pro- and anti-inflammatory mediators, tissue permeability, and regulation of hemodynamics. Genetic deletion of PDGF-B or PDGFR-beta results in an absent glomerular mesangium, whereas PDGF-C and PDGFR-alpha contribute to the formation of the renal cortical interstitium. Almost all experimental and human renal diseases are characterized by altered expression of components of the PDGF system. Infusion or systemic overexpression of PDGF-B or -D induces prominent mesangioproliferative changes and renal fibrosis. Intervention studies identified PDGF-C as a mediator of renal interstitial fibrosis and PDGF-B and -D as key factors involved in mesangioproliferative disease and renal interstitial fibrosis. These data establish PDGF as one of the best characterized growth factors in renal disease and the most potent stimulus of mesangial cell proliferation currently identified. Accordingly, targeted intervention against the various PDGF isoforms offers a promising novel therapeutic approach to renal disease. 
 Mesenchymal stem cell migration is regulated by fibronectin through α5β1-integrin-mediated activation of PDGFR-β and potentiation of growth factor signals. Cell migration during vascular remodelling is regulated by crosstalk between growth factor receptors and integrin receptors, which together coordinate cytoskeletal and motogenic changes. Here, we repo  rt extracellular mat  rix (ECM)-directed crosstalk between platelet-derived growth factor receptor (PDGFR)-β and α5β1-integrin, which controls the migration of mesenchymal stem (stromal) cells (MSCs). Cell adhesion to fibronectin induced α5β1-integrin-dependent phosphorylation of PDGFR-β in the absence of growth factor stimulation. Phosphorylated PDGFR-β co-immunoprecipitated with α5-integrin and colocalised with α5β1-integrin in the transient tidemarks of focal adhesions. Adhesion to fibronectin also strongly potentiated PDGF-BB-induced PDGFR-β phosphorylation and focal adhesion kinase (FAK) activity, in an α5β1-integrin-dependent manner. PDGFR-β-induced phosphoinositide 3-kinase (PI3K) and Akt activity, actin reorganisation and cell migration were all regulated by fibronectin and α5β1-integrin. This synergistic relationship between α5β1-integrin and PDGFR-β is a fundamental determinant of cell migration. Thus, fibronectin-rich matrices can prime PDGFR-β to recruit mesenchymal cells at sites of vascular remodelling. 
 A new look at platelet-derived growth factor in renal disease. The PDGF system, comprising four isoforms (PDGF-A, -B, -C, and -D) and two receptor chains (PDGFR-alpha and -beta), plays important roles in wound healing, atherosclerosis, fibrosis, and malignancy. Components of the system are expressed constitutively or inducibly in most renal cells. They regulate a multitude of pathophysiologic events, ranging from cell proliferation and migration to   extracellular matrix   accumulation, production of pro- and anti-inflammatory mediators, tissue permeability, and regulation of hemodynamics. Genetic deletion of PDGF-B or PDGFR-beta results in an absent glomerular mesangium, whereas PDGF-C and PDGFR-alpha contribute to the formation of the renal cortical interstitium. Almost all experimental and human renal diseases are characterized by altered expression of components of the PDGF system. Infusion or systemic overexpression of PDGF-B or -D induces prominent mesangioproliferative changes and renal fibrosis. Intervention studies identified PDGF-C as a mediator of renal interstitial fibrosis and PDGF-B and -D as key factors involved in mesangioproliferative disease and renal interstitial fibrosis. These data establish PDGF as one of the best characterized growth factors in renal disease and the most potent stimulus of mesangial cell proliferation currently identified. Accordingly, targeted intervention against the various PDGF isoforms offers a promising novel therapeutic approach to renal disease. 
 Sphingosine-1-phosphate induces a PDGFR-dependent cell detachment via inhibiting beta1 integrin in HEK293 cells. Several different types of interactions between sphingosine-1-phosphate (S1P) receptors and platelet-derived growth factor receptor (PDGFR) have been revealed recently. In this work, we used HEK293 cells to further investigate the potential crosstalk. Interestingly, we observed that S1P specifically induced a PDGFR-dependent cell detachment in HEK293 cells, which could be inhibited by AG1296, a specific inhibitor for PDGFR. EGFR on the other hand, did not have any effect on cell detachment. The detachment was   extracellular matrix   (ECM) protein specific, suggesting the involvement of specific integrin molecules. When beta(1) integrin was engaged into an active state, S1P-induced cell detachment was blocked, suggesting that S1P induced an inside-out inhibitory effect on beta(1) integrin. G(i) protein and ERK activation were required for the cell detachment induced by S1P, suggesting an endogenous receptor for S1P is likely to be involved. 
 Thymosin beta4 upregulates the expression of hepatocyte growth factor and downregulates the expression of PDGF-beta receptor in human hepatic stellate cells. Hepatic stellate cells (HSCs) are the main producers of type I collagen in the liver, and therefore are responsible, in part, for the fibrous scar observed in cirrhotic livers. Although there is no approved treatment for this deadly disease, drugs inducing HSC apoptosis in animals (gliotoxin) and hepatocyte regeneration in man (hepatocyte growth factor [HGF]), have been used successfully in ameliorating liver fibrosis. In this communication we investigated whether thymosin beta(4) (Tbeta(4)), an actin-sequestering peptide that prevents scarring of the heart after a myocardial infarction and that prevents kidney fibrosis in animals, has the potential to be used to treat liver fibrosis. To this end we studied whether the administration of Tbeta(4) to HSCs could alter the expression of genes encoding for   extracellular matrix   components, as well as those required for differentiation of HSCs. Our preliminary findings show that Tbeta(4) had no effect on the expression of alpha2 (I) collagen, tissue inhibitor of metalloproteinases-1, and matrix metalloproteinase-2 mRNAs. However, it upregulated the expression of HGF and downregulated the expression of platelet-derived growth factor-beta receptor mRNAs in these cells. Overall, these findings suggest that Tbeta(4) has antifibrogenic potential. 
 PDGFRβ expression and function in fibroblasts derived from pluripotent cells is linked to DNA demethylation. Platelet-derived growth factor receptor-beta (PDGFRβ) is required for the development of mesenchymal cell types, and plays a diverse role in the function of fibroblasts in tissue homeostasis and regeneration. In this study, we characterized the expression of PDGFRβ in fibroblasts derived from human embryonic stem cells (ESCs) and induced pluripotent stem cells (iPSCs), and showed that this expression is important for cellular functions such as migratio  n, extracellular mat  rix production and assembly in 3D self-assembled tissues. To determine potential regulatory regions predictive of expression of PDGFRβ following differentiation from ESCs and iPSCs, we analyzed the DNA methylation status of a region of the PDGFRB promoter that contains multiple CpG sites, before and after differentiation. We demonstrated that this promoter region is extensively demethylated following differentiation, and represents a developmentally regulated, differentially methylated region linked to PDGFRβ expression. Understanding the epigenetic regulation of genes such as PDGFRB, and identifying sites of active DNA demethylation, is essential for future applications of iPSC-derived fibroblasts for regenerative medicine. 
 Altered migration and adhesion potential of pro-neurally converted human bone marrow stromal cells. Bone marrow (BM)-derived mesenchymal stromal cells (MSC) are promising candidate cells for the development of neuroregenerative therapies. We have previously introduced the pro-neural conversion of human MSC to neural stem cell-like cells (m-NSC) by culturing them in suspension culture under serum-free conditions.In the present study, we used a modified Boyden chamber assay to study the influence of various chemoattractants and   extracellular matrix   components on MSC and m-NSC migration in vitro. The underlying mechanisms were investigated further by applying real-time reverse transcriptase (RT)-polymerase chain reaction (PCR) and flow cytometry.The basal migration of m-NSC was significantly reduced compared with MSC (six versus 27 out of 10,000 cells migrated within 6 h). We evaluated the effects of bone morphogenic protein 2 (BMP2), insulin-like growth factor 1 (IGF1), platelet-derived growth factor bb (PDGFbb), vascular endothelial growth factor (VEGFa), and stromal cell-derived factor 1 (SDF1) on the migration potential of both cell types and PDGFbb proved to be the most potent stimulant of migration (235 versus 198 m-NSC or MSC migrated). Adhesion of m-NSC to the filter membrane was delayed and not affected by IGF1 or PDGFbb: 90% of MSC, but only 20% of m-NSC, adhered within 1 h, with 90% of m-NSC adhering within 3 h. However, real-time RT-PCR and flow cytometry revealed an up-regulation of the PDGF receptor B following conversion. Coating the membranes with collagen type I or hyaluronan also significantly influenced cell migration.We could identify major chemoattractive factors for m-NSC and gained partial insight into the complex processes involved in migration of neurally converted cells. 
  [Ii]nvasive  PubMed  
 Evaluation of candidate stromal epithelial cross-talk genes identifies association between risk of serous ovarian cancer and TERT, a cancer susceptibility "hot-spot". We hypothesized that variants in genes expressed as a consequence of interactions between ovarian cancer cells and the host micro-environment could contribute to cancer susceptibility. We therefore used a two-stage approach to evaluate common single nucleotide polymorphisms (SNPs) in 173 genes involved in stromal epithelial interactions in the Ovarian Cancer Association Consortium (OCAC). In the discovery stage, cases with epithelial ovarian cancer (n=675) and controls (n=1,162) were genotyped at 1,536 SNPs using an Illumina GoldenGate assay. Based on Positive Predictive Value estimates, three SNPs-PODXL rs1013368, ITGA6 rs13027811, and MMP3 rs522616-were selected for replication using TaqMan genotyping in up to 3,059 serous   invasive   cases and 8,905 controls from 16 OCAC case-control studies. An additional 18 SNPs with Pper-allele<0.05 in the discovery stage were selected for replication in a subset of five OCAC studies (n=1,233 serous   invasive   cases; n=3,364 controls). The discovery stage associations in PODXL, ITGA6, and MMP3 were attenuated in the larger replication set (adj. Pper-allele>or=0.5). However genotypes at TERT rs7726159 were associated with ovarian cancer risk in the smaller, five-study replication study (Pper-allele=0.03). Combined analysis of the discovery and replication sets for this TERT SNP showed an increased risk of serous ovarian cancer among non-Hispanic whites [adj. ORper-allele 1.14 (1.04-1.24) p=0.003]. Our study adds to the growing evidence that, like the 8q24 locus, the telomerase reverse transcriptase locus at 5p15.33, is a general cancer susceptibility locus. 
 Evaluation of candidate stromal epithelial cross-talk genes identifies association between risk of serous ovarian cancer and TERT, a cancer susceptibility "hot-spot". We hypothesized that variants in genes expressed as a consequence of interactions between ovarian cancer cells and the host micro-environment could contribute to cancer susceptibility. We therefore used a two-stage approach to evaluate common single nucleotide polymorphisms (SNPs) in 173 genes involved in stromal epithelial interactions in the Ovarian Cancer Association Consortium (OCAC). In the discovery stage, cases with epithelial ovarian cancer (n=675) and controls (n=1,162) were genotyped at 1,536 SNPs using an Illumina GoldenGate assay. Based on Positive Predictive Value estimates, three SNPs-PODXL rs1013368, ITGA6 rs13027811, and MMP3 rs522616-were selected for replication using TaqMan genotyping in up to 3,059 serous   invasive   cases and 8,905 controls from 16 OCAC case-control studies. An additional 18 SNPs with Pper-allele<0.05 in the discovery stage were selected for replication in a subset of five OCAC studies (n=1,233 serous   invasive   cases; n=3,364 controls). The discovery stage associations in PODXL, ITGA6, and MMP3 were attenuated in the larger replication set (adj. Pper-allele>or=0.5). However genotypes at TERT rs7726159 were associated with ovarian cancer risk in the smaller, five-study replication study (Pper-allele=0.03). Combined analysis of the discovery and replication sets for this TERT SNP showed an increased risk of serous ovarian cancer among non-Hispanic whites [adj. ORper-allele 1.14 (1.04-1.24) p=0.003]. Our study adds to the growing evidence that, like the 8q24 locus, the telomerase reverse transcriptase locus at 5p15.33, is a general cancer susceptibility locus. 
 Characteristics of the expression of KAI1/CD82 and PDGFRβ and their impact on glioma progression. The biological features of glioma cells may define their clinical outcome. Little is known about the interactions between KAI1/CD82 metastatic suppressor protein and PDGFRβ in gliomas. The aim of the study was to examine KAI1/CD82 and PDGFRβ expression in gliomas in order to find the impact of these proteins on progression of the tumors. PDGFRβ, KAI1/CD82 protein expression and mRNA of genes were evaluated on eighty four paraffin-embedded tissue of gliomas using immunohistochemical staining and RT-PCR analysis. The PDGFRβ expression was higher in IV/III than in I/II glioma grades (p = 0.0004). The level of mRNA PDGFRβ was associated with the degree of PDGFRβ immunoreactivity. Downregulation of KAI1/CD82 was associated with tumor malignancy (p = 0.007). The increased level of KAI1/CD82 gene expression (3-4-fold) was found in gliomas with strong KAI1/CD82 immunoreactivity. The parallel KAI1/CD82 and PDGFRβ expression was more significantly associated with cases in a group graded as III and IV than in a group graded as I/II (p = 0.002). We found that a loss of KAI1/CD82 and an increase in PDGFRβ expression in gliomas relate to a progressive tumor growth. The correlation between PDGFRβ and KAI1 expression in high grade gliomas suggests that a direct or indirect interaction between these proteins might have an impact on   cell mo  tility and invasive behavior of the tumor. 
 P-Rex1 cooperates with PDGFRβ to drive cellular migration in 3D microenvironments. Expression of the Rac-guanine nucleotide exchange factor (RacGEF), P-Rex1 is a key determinant of progression to metastasis in a number of human cancers. In accordance with this proposed role in cancer cell invasion and metastasis, we find that ectopic expression of P-Rex1 in an immortalised human fibroblast cell line is sufficient to drive multiple migratory and   invasiv  e phenotypes. The   invasiv  e phenotype is greatly enhanced by the presence of a gradient of serum or platelet-derived growth factor, and is dependent upon the expression of functional PDGF receptor β. Consistently, th  e invasi  veness of WM852 melanoma cells, which endogenously express P-Rex1 and PDGFRβ, is opposed by siRNA of either of these proteins. Furthermore, the current model of P-Rex1 activation is advanced through demonstration of P-Rex1 and PDGFRβ as components of the same macromolecular complex. These data suggest that P-Rex1 has an influence on physiological migratory processes, such as invasion of cancer cells, both through effects upon classical Rac1-driven motility and a novel association with RTK signalling complexes. 
 Mutant p53 drives pancreatic cancer metastasis through cell-autonomous PDGF receptor β signaling. Missense mutations in the p53 tumor suppressor inactivate its antiproliferative properties but can also promote metastasis through a gain-of-function activity. We show that sustained expression of mutant p53 is required to maintain the prometastatic phenotype of a murine model of pancreatic cancer, a highly metastatic disease that frequently displays p53 mutations. Transcriptional profiling and functional screening identified the platelet-derived growth factor receptor b (PDGFRb) as both necessary and sufficient to mediate these effects. Mutant p53 induced PDGFRb through a cell-autonomous mechanism involving inhibition of a p73/NF-Y complex that represses PDGFRb expression in p53-deficient, no  ninvasiv  e cells. Blocking PDGFRb signaling by RNA interference or by small molecule inhibitors prevented pancreatic cancer cell invasion in vitro and metastasis formation in vivo. Finally, high PDGFRb expression correlates with poor disease-free survival in pancreatic, colon, and ovarian cancer patients, implicating PDGFRb as a prognostic marker and possible target for attenuating metastasis in p53 mutant tumors. 
  GeneRIFs  
 PDGFRbeta   invasive   phenotype is greatly enhanced by the presence of a gradient of serum or platelet-derived growth factor, and is dependent upon the expression of functional PDGF receptor beta 
  [Mm]etastasize  PubMed  
 Bone-metastatic potential of human prostate cancer cells correlates with Akt/PKB activation by alpha platelet-derived growth factor receptor. Prostate adenocarcinoma   metastasize  s to the skeleton more frequently than any other organ. An underlying cause of this phenomenon may be the ability of bone-produced factors to specifically select disseminated prostate cancer cells that are susceptible to their trophic effects. Platelet-derived growth factor (PDGF), a potent mitogen for both normal and tumor cells, is produced in several tissues including bone, where it is synthesized by both osteoblasts and osteoclasts. Here, we show that PDGF causes a significantly stronger activation of the Akt/PKB survival pathway in bone-metastatic prostate cancer cells compared to nonmetastatic cells. Normal prostate epithelial cells and DU-145 prostate cells, originally derived from a brain metastasis, are not responsive to PDGF. In contrast, epidermal growth factor stimulates Akt to the same extent in all prostate cells tested. This difference in PDGF responsiveness depends on the higher expression of alpha-PDGFR in bone-metastatic compared to nonmetastatic prostate cells and the lack of alpha-PDGFR expression in normal and metastatic prostate cells derived from tissues other than bone. Thus, alpha-PDGFR expression might identify prostate cancer cells with the highest propensity to   metastasize   to the skeleton. 
  GeneRIFs  
 alpha-PDGFR expression might identify prostate cancer cells with the highest propensity to   metastasize   to the skeleton 
  [Mm]etastasis  PubMed  
 PDGFRalpha/beta expression correlates with the metastatic behavior of human colorectal cancer: a possible rationale for a molecular targeting strategy. As new multi-target tyrosine kinase inhibitors are emerging in the therapy of various malignancies, our aim was to define the co-expression pattern of receptor-tyrosine-kinase platelet-derived growth factor receptors alpha and beta (PDGFRalpha/beta) in human colorectal cancer. The co-expression pattern of PDGFRalpha/beta was analyzed by RT-PCR in 99 histologically confirmed human colorectal carcinomas and five colorectal cancer cell lines. In addition, immunohistochemical (IHC) staining was applied for confirmation of expression and analysis of receptor tyrosine kinase (RTK) localisation. The colorectal cancer cell lines that were analysed revealed varying expression intensities of PDGFRalpha and PDGFRbeta. The majority of human colorectal cancer specimens revealed a PDGFRalpha (83%) or PDGFRbeta (60%) expression. While PDGFRalpha showed a predominantly cytoplasmic staining in tumor cells as well as in stromal pericytes, PDGFRbeta was restricted to stromal pericytes only. Furthermore, PDGFRalpha expression significantly correlated with lymph node   metastasis   (P=0.0082) and advanced UICC stages III/IV (P=0.018) in older patients (P=0.043). PDGFRbeta expression only revealed a trend towards lymphatic dissemination (P=0.099). Co-expression of PDGFRalpha/beta occurred in 57% of the colorectal cancer samples, whereas another 29% of the samples depicted mono-expression of PDGFRalpha or PDGFRbeta. Notably, PDGFRalpha/beta expression significantly correlated with lymphatic   metastasis   (P=0.007) and advanced UICC stages III/IV (P=0.017) in older patients (P=0.03). In summary, our results revealed that PDGFRalpha/beta expression significantly correlates with lymphatic dissemination and therefore encourages application of PDGFRalpha/beta RTK-inhibitors within a combination therapy. 
 Expression of CAF-Related Proteins Is Associated with Histologic Grade of Breast Phyllodes Tumor. Purpose. The purpose of this study was to investigate the expression of cancer-associated fibroblast- (CAF-) related proteins and the implications in breast phyllodes tumor (PT). Methods. Tissue microarrays of 194 PT cases (151 benign PT, 27 borderline PT, and 16 malignant PT) were constructed. We performed immunohistochemical staining for CAF-related proteins (podoplanin, prolyl 4-hydroxylase, FAPα, S100A4, PDGFR α/β, and NG2) and analyzed the results according to clinicopathologic parameters. Results. Expression of PDGFRα and PDGFRβ in the stromal component increased with increasing histologic grade of PT (p = 0.003 and p = 0.034, resp.). Among clinicopathologic parameters, only expression of FAPα in stroma was associated with di  stant meta  stasis (p = 0.002). In univariate analysis, stromal expression of PDGFRα was associated with shorter overall survival (p = 0.002). In Cox multivariate analysis, stromal overgrowth and PDGFRα stromal positivity were associated with shorter overall survival (p = 0.006 and p = 0.050, resp.). Furthermore, expression of PDGFRβ in stroma was associated with shorter overall survival in patients with malignant PT (p = 0.041). Conclusion. Stromal expression of PDGFRα and PDGFRβ increased with increasing histologic grade of PT. In addition, PDGFR stromal positivity was associated with shorter overall survival. These results suggest that CAFs are associated with breast PT progression. 
 Anti-PlGF inhibits growth of VEGF(R)-inhibitor-resistant tumors without affecting healthy vessels. Novel antiangiogenic strategies with complementary mechanisms are needed to maximize efficacy and minimize resistance to current angiogenesis inhibitors. We explored the therapeutic potential and mechanisms of alphaPlGF, an antibody against placental growth factor (PlGF), a VEGF homolog, which regulates the angiogenic switch in disease, but not in health. alphaPlGF inhibited growth and   metastasis   of various tumors, including those resistant to VEGF(R) inhibitors (VEGF(R)Is), and enhanced the efficacy of chemotherapy and VEGF(R)Is. alphaPlGF inhibited angiogenesis, lymphangiogenesis, and tumor cell motility. Distinct from VEGF(R)Is, alphaPlGF prevented infiltration of angiogenic macrophages and severe tumor hypoxia, and thus, did not switch on the angiogenic rescue program responsible for resistance to VEGF(R)Is. Moreover, it did not cause or enhance VEGF(R)I-related side effects. The efficacy and safety of alphaPlGF, its pleiotropic and complementary mechanism to VEGF(R)Is, and the negligible induction of an angiogenic rescue program suggest that alphaPlGF may constitute a novel approach for cancer treatment. 
 PDGFRα and β play critical roles in mediating Foxq1-driven breast cancer stemness and chemoresistance. Many epithelial-mesenchymal transition (EMT)-promoting transcription factors have been implicated in tumorigenesis an  d metastas  is as well as chemoresistance of cancer. However, the underlying mechanisms mediating these processes are unclear. Here, we report that Foxq1, a forkhead box-containing transcription factor and EMT-inducing gene, promotes stemness traits and chemoresistance in mammary epithelial cells. Using an expression profiling assay, we identified Twist1, Zeb2, and PDGFRα and β as Foxq1 downstream targets. We further show that PDGFRα and β can be directly regulated by Foxq1 or indirectly regulated through the Foxq1/Twist1 axis. Knockdown of both PDGFRα and β results in more significant effects on reversing Foxq1-promoted oncogenesis in vitro and in vivo than knockdown of either PDGFRα or β alone. In addition, PDGFRβ is a more potent mediator of Foxq1-promoted stemness traits than PDGFRα. Finally, pharmacologic inhibition or gene silencing of PDGFRs sensitizes mammary epithelial cells to chemotherapeutic agents in vitro and in vivo. These findings collectively implicate PDGFRs as critical mediators of breast cancer oncogenesis and chemoresistance driven by Foxq1, with potential implications for developing novel therapeutic combinations to treat breast cancer. 
 MicroRNA-34A inhibits the growth, invasion and   metastasis   of gastric cancer by targeting PDGFR and MET expression. Within the family of RTKs (receptor tyrosine kinases), PDGFR (platelet-derived growth factor receptor) has been implicated in carcinogenesis and tumour development. miRNAs (microRNAs), which can target the mRNAs (messenger RNAs) of cancer-associated genes, are abnormally expressed in various cancers. In this study, our aim was to identify the miRNAs that target PDGFR-α/β and to study the functions of these miRNAs. miR-34a was predicted to target PDGFR, and luciferase reporter assays showed that miR-34a could directly target PDGFR. Meanwhile, we found that miR-34a was down-regulated in gastric cancer tissues and was associated wit  h metastas  is. Our findings showed that miR-34a could inhibit gastric cancer cell migration, invasion and proliferation, but these tumourigenic properties were only partially restored when PDGFR-α/β was overexpressed. In subsequent experiments, we found that the overexpression of both PDGFR and MET could completely restore the gastric cancer tumourigenic properties. Moreover, the cancer-associated cell signalling pathway was studied, and we found that miR-34a could inhibit Akt [PKB (protein kinase B)] phosphorylation, which was restored by the overexpression of both PDGFR and MET. In conclusion, miR-34a may act as a potential tumour suppressor in gastric cancer and is associated with the mechanisms of gastric can  cer metast  asis; miR-34a can inhibit gastric cancer tumourigenesis by targeting PDGFR and MET through the PI3K (phosphoinositide 3-kinase)/Akt pathway. 
 PDGF: the nuts and bolts of signalling toolbox. PDGF is a growth factor and is extensively involved in multi-dimensional cellular dynamics. It switches on a plethora of molecules other than its classical pathway. It is engaged in various transitions of development; however, if the unleashed potentials lead astray, it brings forth tumourigenesis. Conventionally, it has been assumed that the components of this signalling pathway show fidelity and act with a high degree of autonomy. However, as illustrated by the PDGF signal transduction, reinterpretation of recent data suggests that machinery is often shared between multiple pathways, and other components crosstalk to each other through multiple mechanisms. It is important to note that metastatic cascade is an intricate process that we have only begun to understand in recent years. Many of the early steps of this PDGF cascade are not readily targetable in the clinic. In this review, we will unravel the paradoxes with reference to mitrons and cellular plasticity and discuss how disruption of signalling cascade triggers cellular proliferation phase transition and   metastasis  . We will also focus on the therapeutic interventions to counteract resultant molecular disorders. 
 Use of signal specific receptor tyrosine kinase oncoproteins reveals that pathways downstream from Grb2 or Shc are sufficient for cell transformation and   metastasis  . Many human cancers have been associated with the deregulation of receptor tyrosine kinases (RTK). However, the individual contribution of receptor-associated signaling proteins in cellular transformation and   metastasis   is poorly understood. To examine the role of RTK activated signal transduction pathways to processes involved in cell transformation, we have exploited the oncogenic derivative of the Met RTK (Tpr-Met). Unlike other RTKs, twin tyrosine residues in the carboxy-terminal tail of the Met oncoprotein and receptor are required for all biological and transforming activities, and a mutant lacking these tyrosines is catalytically active but non transforming. Using this mutant we have inserted oligonucleotide cassettes, each encoding a binding site for a specific signaling protein derived from other RTKs. We have generated variant forms of the Tpr-Met oncoprotein with the ability to bind individually to the p85 subunit of PI3'K, PLCgamma, or to the Grb2 or Shc adaptor proteins. Variants that recruit the Shc or Grb2 adaptor proteins generated foci of morphologically transformed fibroblast cells and induced anchorage-independent growth, scattering of epithelial cells and experimental   metastasis  . In contrast, variants that bind and activate PI3'K or PLCgamma failed to generate readily detectable foci. Although cell lines expressing the PI3'K variant grew in soft-agar, these cells were non metastatic. Using this unique RTK oncoprotein model, we have established that Grb2 or Shc dependent signaling pathways are sufficient for cell transformation and metastatic spread. 
 PDGFRα/β and VEGFR2 polymorphisms in colorectal cancer: incidence and implications in clinical outcome. Angiogenesis plays an essential role in tumor growth an  d metastas  is, and is a major target in cancer therapy. VEGFR and PDGFR are key players involved in this process. The purpose of this study was to assess the incidence of genetic variants in these receptors and its potential clinical implications in colorectal cancer (CRC).VEGFR2, PDGFRα and PDGFRβ mutations were evaluated by sequencing their tyrosine kinase domains in 8 CRC cell lines and in 92 samples of patients with CRC. Correlations with clinicopathological features and survival were analyzed.Four SNPs were identified, three in PDGFRα [exon 12 (A12): c.1701A>G; exon 13 (A13): c.1809G>A; and exon 17 (A17): c.2439+58C>A] and one in PDGFRβ [exon 19 (B19): c.2601A>G]. SNP B19, identified in 58% of tumor samples and in 4 cell lines (LS174T, LS180, SW48, COLO205), was associated with higher PDGFR and pPDGFR protein levels. Consistent with this observation, 5-year survival was greater for patients with PDGFR B19 wild type tumors (AA) than for those harboring the G-allele genotype (GA or GG) (51% vs 17%; p=0.073). Multivariate analysis confirmed SNP B19 (p=0.029) was a significant prognostic factor for survival, independent of age (p=0.060) or TNM stage (p<0.001).PDGFRβ exon 19 c.2601A>G SNP is commonly encountered in CRC patients and is associated with increased pathway activation and poorer survival. Implications regarding its potential influence in response to PDGFR-targeted agents remain to be elucidated. 
 Expression of CAF-Related Proteins Is Associated with Histologic Grade of Breast Phyllodes Tumor. Purpose. The purpose of this study was to investigate the expression of cancer-associated fibroblast- (CAF-) related proteins and the implications in breast phyllodes tumor (PT). Methods. Tissue microarrays of 194 PT cases (151 benign PT, 27 borderline PT, and 16 malignant PT) were constructed. We performed immunohistochemical staining for CAF-related proteins (podoplanin, prolyl 4-hydroxylase, FAPα, S100A4, PDGFR α/β, and NG2) and analyzed the results according to clinicopathologic parameters. Results. Expression of PDGFRα and PDGFRβ in the stromal component increased with increasing histologic grade of PT (p = 0.003 and p = 0.034, resp.). Among clinicopathologic parameters, only expression of FAPα in stroma was associated with di  stant meta  stasis (p = 0.002). In univariate analysis, stromal expression of PDGFRα was associated with shorter overall survival (p = 0.002). In Cox multivariate analysis, stromal overgrowth and PDGFRα stromal positivity were associated with shorter overall survival (p = 0.006 and p = 0.050, resp.). Furthermore, expression of PDGFRβ in stroma was associated with shorter overall survival in patients with malignant PT (p = 0.041). Conclusion. Stromal expression of PDGFRα and PDGFRβ increased with increasing histologic grade of PT. In addition, PDGFR stromal positivity was associated with shorter overall survival. These results suggest that CAFs are associated with breast PT progression. 
 PDGFRα and β play critical roles in mediating Foxq1-driven breast cancer stemness and chemoresistance. Many epithelial-mesenchymal transition (EMT)-promoting transcription factors have been implicated in tumorigenesis an  d metastas  is as well as chemoresistance of cancer. However, the underlying mechanisms mediating these processes are unclear. Here, we report that Foxq1, a forkhead box-containing transcription factor and EMT-inducing gene, promotes stemness traits and chemoresistance in mammary epithelial cells. Using an expression profiling assay, we identified Twist1, Zeb2, and PDGFRα and β as Foxq1 downstream targets. We further show that PDGFRα and β can be directly regulated by Foxq1 or indirectly regulated through the Foxq1/Twist1 axis. Knockdown of both PDGFRα and β results in more significant effects on reversing Foxq1-promoted oncogenesis in vitro and in vivo than knockdown of either PDGFRα or β alone. In addition, PDGFRβ is a more potent mediator of Foxq1-promoted stemness traits than PDGFRα. Finally, pharmacologic inhibition or gene silencing of PDGFRs sensitizes mammary epithelial cells to chemotherapeutic agents in vitro and in vivo. These findings collectively implicate PDGFRs as critical mediators of breast cancer oncogenesis and chemoresistance driven by Foxq1, with potential implications for developing novel therapeutic combinations to treat breast cancer. 
 MicroRNA-34A inhibits the growth, invasion and   metastasis   of gastric cancer by targeting PDGFR and MET expression. Within the family of RTKs (receptor tyrosine kinases), PDGFR (platelet-derived growth factor receptor) has been implicated in carcinogenesis and tumour development. miRNAs (microRNAs), which can target the mRNAs (messenger RNAs) of cancer-associated genes, are abnormally expressed in various cancers. In this study, our aim was to identify the miRNAs that target PDGFR-α/β and to study the functions of these miRNAs. miR-34a was predicted to target PDGFR, and luciferase reporter assays showed that miR-34a could directly target PDGFR. Meanwhile, we found that miR-34a was down-regulated in gastric cancer tissues and was associated wit  h metastas  is. Our findings showed that miR-34a could inhibit gastric cancer cell migration, invasion and proliferation, but these tumourigenic properties were only partially restored when PDGFR-α/β was overexpressed. In subsequent experiments, we found that the overexpression of both PDGFR and MET could completely restore the gastric cancer tumourigenic properties. Moreover, the cancer-associated cell signalling pathway was studied, and we found that miR-34a could inhibit Akt [PKB (protein kinase B)] phosphorylation, which was restored by the overexpression of both PDGFR and MET. In conclusion, miR-34a may act as a potential tumour suppressor in gastric cancer and is associated with the mechanisms of gastric can  cer metast  asis; miR-34a can inhibit gastric cancer tumourigenesis by targeting PDGFR and MET through the PI3K (phosphoinositide 3-kinase)/Akt pathway. 
 PDGFRα/β and VEGFR2 polymorphisms in colorectal cancer: incidence and implications in clinical outcome. Angiogenesis plays an essential role in tumor growth an  d metastas  is, and is a major target in cancer therapy. VEGFR and PDGFR are key players involved in this process. The purpose of this study was to assess the incidence of genetic variants in these receptors and its potential clinical implications in colorectal cancer (CRC).VEGFR2, PDGFRα and PDGFRβ mutations were evaluated by sequencing their tyrosine kinase domains in 8 CRC cell lines and in 92 samples of patients with CRC. Correlations with clinicopathological features and survival were analyzed.Four SNPs were identified, three in PDGFRα [exon 12 (A12): c.1701A>G; exon 13 (A13): c.1809G>A; and exon 17 (A17): c.2439+58C>A] and one in PDGFRβ [exon 19 (B19): c.2601A>G]. SNP B19, identified in 58% of tumor samples and in 4 cell lines (LS174T, LS180, SW48, COLO205), was associated with higher PDGFR and pPDGFR protein levels. Consistent with this observation, 5-year survival was greater for patients with PDGFR B19 wild type tumors (AA) than for those harboring the G-allele genotype (GA or GG) (51% vs 17%; p=0.073). Multivariate analysis confirmed SNP B19 (p=0.029) was a significant prognostic factor for survival, independent of age (p=0.060) or TNM stage (p<0.001).PDGFRβ exon 19 c.2601A>G SNP is commonly encountered in CRC patients and is associated with increased pathway activation and poorer survival. Implications regarding its potential influence in response to PDGFR-targeted agents remain to be elucidated. 
 PDGF: the nuts and bolts of signalling toolbox. PDGF is a growth factor and is extensively involved in multi-dimensional cellular dynamics. It switches on a plethora of molecules other than its classical pathway. It is engaged in various transitions of development; however, if the unleashed potentials lead astray, it brings forth tumourigenesis. Conventionally, it has been assumed that the components of this signalling pathway show fidelity and act with a high degree of autonomy. However, as illustrated by the PDGF signal transduction, reinterpretation of recent data suggests that machinery is often shared between multiple pathways, and other components crosstalk to each other through multiple mechanisms. It is important to note that metastatic cascade is an intricate process that we have only begun to understand in recent years. Many of the early steps of this PDGF cascade are not readily targetable in the clinic. In this review, we will unravel the paradoxes with reference to mitrons and cellular plasticity and discuss how disruption of signalling cascade triggers cellular proliferation phase transition and   metastasis  . We will also focus on the therapeutic interventions to counteract resultant molecular disorders. 
 PDGFRalpha/beta expression correlates with the metastatic behavior of human colorectal cancer: a possible rationale for a molecular targeting strategy. As new multi-target tyrosine kinase inhibitors are emerging in the therapy of various malignancies, our aim was to define the co-expression pattern of receptor-tyrosine-kinase platelet-derived growth factor receptors alpha and beta (PDGFRalpha/beta) in human colorectal cancer. The co-expression pattern of PDGFRalpha/beta was analyzed by RT-PCR in 99 histologically confirmed human colorectal carcinomas and five colorectal cancer cell lines. In addition, immunohistochemical (IHC) staining was applied for confirmation of expression and analysis of receptor tyrosine kinase (RTK) localisation. The colorectal cancer cell lines that were analysed revealed varying expression intensities of PDGFRalpha and PDGFRbeta. The majority of human colorectal cancer specimens revealed a PDGFRalpha (83%) or PDGFRbeta (60%) expression. While PDGFRalpha showed a predominantly cytoplasmic staining in tumor cells as well as in stromal pericytes, PDGFRbeta was restricted to stromal pericytes only. Furthermore, PDGFRalpha expression significantly correlated with lymph node   metastasis   (P=0.0082) and advanced UICC stages III/IV (P=0.018) in older patients (P=0.043). PDGFRbeta expression only revealed a trend towards lymphatic dissemination (P=0.099). Co-expression of PDGFRalpha/beta occurred in 57% of the colorectal cancer samples, whereas another 29% of the samples depicted mono-expression of PDGFRalpha or PDGFRbeta. Notably, PDGFRalpha/beta expression significantly correlated with lymphatic   metastasis   (P=0.007) and advanced UICC stages III/IV (P=0.017) in older patients (P=0.03). In summary, our results revealed that PDGFRalpha/beta expression significantly correlates with lymphatic dissemination and therefore encourages application of PDGFRalpha/beta RTK-inhibitors within a combination therapy. 
 Bone-metastatic potential of human prostate cancer cells correlates with Akt/PKB activation by alpha platelet-derived growth factor receptor. Prostate adenocarcinoma metastasizes to the skeleton more frequently than any other organ. An underlying cause of this phenomenon may be the ability of bone-produced factors to specifically select disseminated prostate cancer cells that are susceptible to their trophic effects. Platelet-derived growth factor (PDGF), a potent mitogen for both normal and tumor cells, is produced in several tissues including bone, where it is synthesized by both osteoblasts and osteoclasts. Here, we show that PDGF causes a significantly stronger activation of the Akt/PKB survival pathway in bone-metastatic prostate cancer cells compared to nonmetastatic cells. Normal prostate epithelial cells and DU-145 prostate cells, originally derived from a brain   metastasis  , are not responsive to PDGF. In contrast, epidermal growth factor stimulates Akt to the same extent in all prostate cells tested. This difference in PDGF responsiveness depends on the higher expression of alpha-PDGFR in bone-metastatic compared to nonmetastatic prostate cells and the lack of alpha-PDGFR expression in normal and metastatic prostate cells derived from tissues other than bone. Thus, alpha-PDGFR expression might identify prostate cancer cells with the highest propensity to metastasize to the skeleton. 
 Expression of platelet-derived growth factor receptor β is maintained by Prox1 in lymphatic endothelial cells and is required for tumor lymphangiogenesis. The lymphatic system plays important roles not only in the physiological processes, such as maintenance of tissue fluid homeostasis, but also in pathological processes including the lymph node   metastasi  s of tumor cells. Therefore, understanding of the molecular mechanisms underlying lymphatic vessel formation is crucial. Previous studies have shown that proliferation and migration of lymphatic endothelial cells (LECs) are activated by multiple types of signals mediated by tyrosine kinase receptors such as vascular endothelial growth factor receptor (VEGFR) 3. Although signals mediated by platelet-derived growth factor receptor β (PDGFRβ) have been implicated in lymphangiogenesis, the mechanisms explaining how PDGFRβ expression is maintained in LECs remain to be fully elucidated. In the present study, we show that PDGFRβ expression in LECs is maintained by Prox1 transcription factor. Knockdown of Prox1 expression in human dermal LECs decreased the expression of PDGFRβ, leading to the lowered migration of human dermal LECs towards PDGF-BB. Furthermore, we found that PDGF signals play important roles in inflammatory lymphangiogenesis in a chronic aseptic peritonitis model. Intraperitoneal administration of imatinib, a potent inhibitor of PDGFRβ, and transduction of PDGFRβ/Fc chimeric protein by an adenoviral system both reduced inflammatory lymphangiogenesis induced by thioglycollate in mice. We also found that the expression of PDGFRβ/Fc reduced tumor lymphangiogenesis in a BxPC3 human pancreatic cancer xenograft model. These findings suggest that PDGFRβ is one of the key mediators of lymphatic vessel formation acting downstream of Prox1. 
 Expression of platelet-derived growth factor (PDGF)-B and PDGF-receptor β is associated with lymphatic   metastasi  s in human gastric carcinoma. Recent study of murine fibrosarcoma has revealed that platelet-derived growth factor (PDGF) plays a direct role in promoting lymphangiogenesis and metastatic spread to lymph nodes. Thus, we investigated the relation between PDGF and PDGF receptor (PDGF-R) expression and lymphatic   metastasi  s in human gastric carcinoma. We examined PDGF-B and PDGF-Rβ expression in four human gastric carcinoma cell lines (TMK-1, MKN-1, MKN-45, and KKLS) and in 38 surgical specimens of gastric carcinoma. PDGF-B and PDGF-Rβ expression was examined by immunofluorescence in surgical specimens and in human gastric carcinoma cells (TMK-1) implanted orthotopically in nude mice. Groups of mice (n = 10, each) received saline (control) or PDGF-R tyrosine kinase inhibitor imatinib. PDGF-B and PDGF-Rβ mRNA expression was significantly higher in patients with lymph n  ode metast  asis than in those without and was also significantly higher in diffuse-type carcinoma than in intestinal-type carcinoma. In surgical specimens, tumor cells expressed PDGF-B, but PDGF-Rβ was expressed predominantly by stromal cells. Under culture conditions, expression of PDGF-B mRNA was found in all of the gastric cell lines, albeit at different levels. In orthotopic TMK-1 tumors, cancer cells expressed PDGF-B but not PDGF-Rβ. PDGF-Rβ was expressed by stromal cells, including lymphatic endothelial cells. Four weeks of treatment with imatinib significantly decreased the area of lymphatic vessels. Our data indicate that secretion of PDGF-B by gastric carcinoma cells and expression of PDGF-Rβ by tumor-associated stromal cells are associated with ly  mphatic me  tastasis. Blockade of PDGF-R signaling pathways may inhibit lym  ph node me  tastasis of gastric carcinoma. 
 Clinicopathological significance of platelet-derived growth factor (PDGF)-B and vascular endothelial growth factor-A expression, PDGF receptor-β phosphorylation, and microvessel density in gastric cancer. Angiogenesis is important in the growth and   metastasi  s of various kinds of solid tumors, including gastric cancers. The angiogenic process is triggered by several key growth factors, including vascular endothelial growth factor (VEGF)-A and platelet-derived growth factor (PDGF)-B, that are secreted by tumors. Our aim was to define: i) the expression pattern of VEGF-A and PDGF-B in tumor cells and the activation of PDGF receptor (PDGFR)-β tyrosine kinase in stromal cells of human gastric adenocarcinomas; and ii) the relationship between VEGF-A and PDGF-B expression and microvessel density (MVD), to determine if there is a rationale for a new therapeutic strategy.A series of 109 gastric adenocarcinoma cases that had undergone surgical resection was examined immunohistochemically using antibodies against VEGF-A, PDGF-B, and CD34, followed by further examination of PDGFR-β phosphorylation by immunoblotting analysis.MVD was higher in diffuse-type than intestinal-type cancers (p < 0.001). VEGF-A overexpression correlated to PDGF-B overexpression in both the intestinal-type (p < 0.005) and diffuse-type (p < 0.0001) groups, indicating that VEGF-A and PDGF-B are secreted simultaneously in the same tumor, and may thus play important roles together in angiogenesis. However, several differences between intestinal-type and diffuse-type cancers were observed. In the diffuse-type cancer group, higher MVD was related to the PDGF-B proportion (p < 0.05) and VEGF-A overexpression (p < 0.05), but not to PDGF-B overexpression or the VEGF-A proportion. On the other hand, in the intestinal-type cancer group, higher MVD was correlated to overexpression (p < 0.005), intensity (p < 0.05), and proportion (p < 0.05) of PDGF-B, but not of VEGF-A. In addition, phosphorylation of PDGFR-β was correlated with depth of cancer invasion at statistically significant level.Our results indicate that PDGF-B, which is involved in the maintenance of microvessels, plays a more important role in angiogenesis in intestinal-type gastric carcinomas than VEGF-A, which plays a key role mainly in the initiation of new blood vessel formation. In contrast, VEGF-A has a critical role for angiogenesis more in diffuse-type cancers, but less in those of intestinal type. Thus, a therapy targeting the PDGF-B signaling pathway could be effective for intestinal-type gastric carcinoma, whereas targeting VEGF-A or both VEGF-A and PDGF-B signaling pathways could be effective for diffuse-type gastric carcinomas. 
 PDGFR signaling blockade in marrow stroma impairs lung cancer bone   metastasis  . Bone microenvironment and cell-cell interactions are crucial for the initiation and development of   metastasis  . By means of a pharmacologic approach, using the multitargeted tyrosine kinase inhibitor sunitinib, we tested the relevance of the platelet-derived growth factor receptor (PDGFR) axis in the bone marrow (BM) stromal compartment for the initiation and development of lung cancer   metastasis   to bone. PDGFRβ was found to be the main tyrosine kinase target of sunitinib expressed in BM stromal ST-2 and MC3T3-E1 preosteoblastic cells. In contrast, no expression of sunitinib-targeted receptors was found in A549M1 and low levels in H460M5 lung cancer metastatic cells. Incubation of ST-2 and human BM endothelial cells with sunitinib led to potent cell growth inhibition and induction of apoptosis in a dose-dependent manner. Similarly, sunitinib induced a robust proapoptotic effect in vivo on BM stromal PDGFRβ(+) cells and produced extensive disruption of tissue architecture and vessel leakage in the BM cavity. Pretreatment of ST-2 cells with sunitinib also hindered heterotypic adhesion to lung cancer cell lines. These effects were correlated with changes in cell-cell and cell-matrix molecules in both stromal and tumor cells. Pretreatment of mice with sunitinib before intracardiac inoculation of A549M1 or H460M5 cells caused marked inhibition of tumor cells homing to bone, whereas no effect was found when tumor cells were pretreated before inoculation. Treatment with sunitinib dramatically increased overall survival and prevented tumor colonization but not bone lesions, whereas combination with zoledronic acid resulted in marked reduction of osteolytic lesions and osseous tumor burden. Thus, disruption of the PDGFR axis in the BM stroma alters heterotypic tumor-stromal and tumor-matrix interactions, thereby preventing efficient engagement required for bone homing and osseous colonization. These results support the notion that concomitant targeting of the tumor and stromal compartment is a more effective approach for blocking bon  e metastas  is. 
 P-Rex1 cooperates with PDGFRβ to drive cellular migration in 3D microenvironments. Expression of the Rac-guanine nucleotide exchange factor (RacGEF), P-Rex1 is a key determinant of progression to   metastasi  s in a number of human cancers. In accordance with this proposed role in cancer cell invasion and   metastasi  s, we find that ectopic expression of P-Rex1 in an immortalised human fibroblast cell line is sufficient to drive multiple migratory and invasive phenotypes. The invasive phenotype is greatly enhanced by the presence of a gradient of serum or platelet-derived growth factor, and is dependent upon the expression of functional PDGF receptor β. Consistently, the invasiveness of WM852 melanoma cells, which endogenously express P-Rex1 and PDGFRβ, is opposed by siRNA of either of these proteins. Furthermore, the current model of P-Rex1 activation is advanced through demonstration of P-Rex1 and PDGFRβ as components of the same macromolecular complex. These data suggest that P-Rex1 has an influence on physiological migratory processes, such as invasion of cancer cells, both through effects upon classical Rac1-driven motility and a novel association with RTK signalling complexes. 
 Mutant p53 drives pancreatic cancer   metastasis   through cell-autonomous PDGF receptor β signaling. Missense mutations in the p53 tumor suppressor inactivate its antiproliferative properties but can also promote   metastasi  s through a gain-of-function activity. We show that sustained expression of mutant p53 is required to maintain the prometastatic phenotype of a murine model of pancreatic cancer, a highly metastatic disease that frequently displays p53 mutations. Transcriptional profiling and functional screening identified the platelet-derived growth factor receptor b (PDGFRb) as both necessary and sufficient to mediate these effects. Mutant p53 induced PDGFRb through a cell-autonomous mechanism involving inhibition of a p73/NF-Y complex that represses PDGFRb expression in p53-deficient, noninvasive cells. Blocking PDGFRb signaling by RNA interference or by small molecule inhibitors prevented pancreatic cancer cell invasion in vitro and   metastasi  s formation in vivo. Finally, high PDGFRb expression correlates with poor disease-free survival in pancreatic, colon, and ovarian cancer patients, implicating PDGFRb as a prognostic marker and possible target for attenuating   metastasi  s in p53 mutant tumors. 
  GeneRIFs  
 Study combined several orthogonal approaches and models to explore the basis whereby mutant p53 promotes invasion and   metastasis   in pancreatic ductal adenocarcinoma (PDAC)and identified the PDGFRb as necessary and sufficient to mediate the effects of mutant p53 on invasion and   metastasis   in both a murine model and human PDAC cells. 
 Increased expression of platelet-derived growth factor receptor-beta in pancreatic cancer cells is mediated by DNA binding and structural mutants of p53 and that this contributes to   metastasis   
 Data indicate that secretion of PDGF-B by gastric carcinoma cells and expression of PDGF-Rbeta by tumor-associated stromal cells are associated with lymphatic   metastasis  . 
  [Cc]ell [Ii]nvasion  PubMed  
 P-Rex1 cooperates with PDGFRβ to drive cellular migration in 3D microenvironments. Expression of the Rac-guanine nucleotide exchange factor (RacGEF), P-Rex1 is a key determinant of progression to metastasis in a number of human cancers. In accordance with this proposed role in cancer   cell invasio  n and metastasis, we find that ectopic expression of P-Rex1 in an immortalised human fibroblast cell line is sufficient to drive multiple migratory and invasive phenotypes. The invasive phenotype is greatly enhanced by the presence of a gradient of serum or platelet-derived growth factor, and is dependent upon the expression of functional PDGF receptor β. Consistently, the invasiveness of WM852 melanoma cells, which endogenously express P-Rex1 and PDGFRβ, is opposed by siRNA of either of these proteins. Furthermore, the current model of P-Rex1 activation is advanced through demonstration of P-Rex1 and PDGFRβ as components of the same macromolecular complex. These data suggest that P-Rex1 has an influence on physiological migratory processes, such as invasion of cancer cells, both through effects upon classical Rac1-driven motility and a novel association with RTK signalling complexes. 
 Radiation-induced senescence in securin-deficient cancer cells promotes   cell invasion   involving the IL-6/STAT3 and PDGF-BB/PDGFR pathways. Securin overexpression correlates with poor prognosis in various tumours. We have previously shown that securin depletion promotes radiation-induced senescence and enhances radiosensitivity in human cancer cells. However, the underlying molecular mechanisms and the paracrine effects remain unknown. In this study, we showed that radiation induced senescence in securin-deficient human breast cancer cells involving the ATM/Chk2 and p38 pathways. Conditioned medium (CM) from senescent cells promoted the invasion and migration of non-irradiated cancer and endothelial cells. Cytokine assay analysis showed the up-regulation of various senescence-associated secretory phenotypes (SASPs). The IL-6/STAT3 signalling loop and platelet-derived growth factor-BB (PDGF-BB)/PDGF receptor (PDGFR) pathway were important for CM-induced cell migration and invasion. Furthermore, CM promoted angiogenesis in the chicken chorioallantoic membrane though the induction of IL-6/STAT3- and PDGF-BB/PDGFR-dependent endothelial   cell invasion  . Taken together, our results provide the molecular mechanisms for radiation-induced senescence in securin-deficient human breast cancer cells and for the SASP responses. 
 Mutant p53 drives pancreatic cancer metastasis through cell-autonomous PDGF receptor β signaling. Missense mutations in the p53 tumor suppressor inactivate its antiproliferative properties but can also promote metastasis through a gain-of-function activity. We show that sustained expression of mutant p53 is required to maintain the prometastatic phenotype of a murine model of pancreatic cancer, a highly metastatic disease that frequently displays p53 mutations. Transcriptional profiling and functional screening identified the platelet-derived growth factor receptor b (PDGFRb) as both necessary and sufficient to mediate these effects. Mutant p53 induced PDGFRb through a cell-autonomous mechanism involving inhibition of a p73/NF-Y complex that represses PDGFRb expression in p53-deficient, noninvasive cells. Blocking PDGFRb signaling by RNA interference or by small molecule inhibitors prevented pancreatic cancer   cell invasio  n in vitro and metastasis formation in vivo. Finally, high PDGFRb expression correlates with poor disease-free survival in pancreatic, colon, and ovarian cancer patients, implicating PDGFRb as a prognostic marker and possible target for attenuating metastasis in p53 mutant tumors. 
   
  
 CSF1R 
 
  NCBI Gene  
  GeneRIF  
  Pubmed  
 
 
  Search Term  Rank  Fields  
  [Mm]igration  6  Process(1); PubMed(2)  
  [Ii]nvasive  10  PubMed(8)  
  [Mm]etastasis  12  PubMed(5)  
  [Cc]ell [Ii]nvasion  13  PubMed(1)  
 
 
 hide/show details for CSF1R 
  [Mm]igration  Process  
 positive regulation of cell   migration   
  PubMed  
 CSF1R signaling blockade stanches tumor-infiltrating myeloid cells and improves the efficacy of radiotherapy in prostate cancer. Radiotherapy is used to treat many types of cancer, but many treated patients relapse with local tumor recurrence. Tumor-infiltrating myeloid cells (TIM), including CD11b (ITGAM)(+)F4/80 (EMR1)+ tumor-associated macrophages (TAM), and CD11b(+)Gr-1 (LY6G)+ myeloid-derived suppressor cells (MDSC), respond to cancer-related stresses and play critical roles in promoting tumor angiogenesis, tissue remodeling, and immunosuppression. In this report, we used a prostate cancer model to investigate the effects of irradiation on TAMs and MDSCs in tumor-bearing animals. Unexpectedly, when primary tumor sites were irradiated, we observed a systemic increase of MDSCs in spleen, lung, lymph nodes, and peripheral blood. Cytokine analysis showed that the macrophage colony-stimulating factor CSF1 increased by two-fold in irradiated tumors. Enhanced macrophage   migration   induced by conditioned media from irradiated tumor cells was completely blocked by a selective inhibitor of CSF1R. These findings were confirmed in patients with prostate cancer, where serum levels of CSF1 increased after radiotherapy. Mechanistic investigations revealed the recruitment of the DNA damage-induced kinase ABL1 into cell nuclei where it bound the CSF1 gene promoter and enhanced CSF1 gene transcription. When added to radiotherapy, a selective inhibitor of CSF1R suppressed tumor growth more effectively than irradiation alone. Our results highlight the importance of CSF1/CSF1R signaling in the recruitment of TIMs that can limit the efficacy of radiotherapy. Furthermore, they suggest that CSF1 inhibitors should be evaluated in clinical trials in combination with radiotherapy as a strategy to improve outcomes. 
 Therapeutic applications of macrophage colony-stimulating factor-1 (CSF-1) and antagonists of CSF-1 receptor (CSF-1R) signaling. Macrophage-colony stimulating factor (CSF-1) signaling through its receptor (CSF-1R) promotes the differentiation of myeloid progenitors into heterogeneous populations of monocytes, macrophages, dendritic cells, and bone-resorbing osteoclasts. In the periphery, CSF-1 regulates the   migration  , proliferation, function, and survival of macrophages, which function at multiple levels within the innate and adaptive immune systems. Macrophage populations elicited by CSF-1 are associated with, and exacerbate, a broad spectrum of pathologies, including cancer, inflammation, and bone disease. Conversely, macrophages can also contribute to immunosuppression, disease resolution, and tissue repair. Recombinant CSF-1, antibodies against the ligand and the receptor, and specific inhibitors of CSF-1R kinase activity have been each been tested in a range of animal models and in some cases, in patients. This review examines the potential clinical uses of modulators of the CSF-1/CSF-1R system. We conclude that CSF-1 promotes a resident-type macrophage phenotype. As a treatment, CSF-1 has therapeutic potential in tissue repair. Conversely, inhibition of CSF-1R is unlikely to be effective in inflammatory disease but may have utility in cancer. 
  [Ii]nvasive  PubMed  
 Up-regulation of VEGF, c-fms and COX-2 expression correlates with severity of cervical cancer precursor (CIN) lesions and   invasive   disease. To describe the expression of vascular endothelial growth factor (VEGF), proto-oncogene macrophage colony-stimulating factor receptor (c-fms) and cyclooxygenase-2 (COX-2) in cervical carcinogenesis and to analyze the correlation of VEGF with c-fms and COX-2 expression.In this study, 26 cases of benign cervix, 28 low-grade cervical intraepithelial neoplasia (CIN; CIN 1), 30 high-grade CIN (CIN 2/3) and 28 squamous cervical carcinomas (SCC) were examined by immunohistochemistry (IHC) and analysis was performed separately for epithelium and stroma.Positive epithelial expressions in normal cervix, low-grade CIN, high-grade CIN and SCC were, respectively: VEGF - 11.5%, 39.3%, 53.3% and 75% (P<0.001); c-fms - 0%, 10.7%, 40% and 67.9% (P<0.001); COX-2 - 7.7%, 39.3%, 80% and 100% (P<0.001). Stromal VEGF expression was higher than epithelial expression in all CIN grades and was also associated with the lesion grade, while c-fms and COX-2 stromal expression was weak. VEGF expression was statistically correlated to c-fms and COX-2 expression in high-grade CIN (P=0.020 and P=0.027, respectively) and SCC (P=0.015 and P=0.005, respectively).On the basis of our findings, these factors may participate in the development and progression of CIN lesions, with possible interaction of c-fms and COX-2 on VEGF expression, and may be potential molecular targets for studies of cervical cancer prevention and treatment. 
 Genetic susceptibility to distinct bladder cancer subphenotypes. Clinical, pathologic, and molecular evidence indicate that bladder cancer is heterogeneous with pathologic/molecular features that define distinct subphenotypes with different prognoses. It is conceivable that specific patterns of genetic susceptibility are associated with particular subphenotypes.To examine evidence for the contribution of germline genetic variation to bladder cancer heterogeneity.The Spanish Bladder Cancer/EPICURO Study is a case-control study based in 18 hospitals located in five areas in Spain. Cases were patients with a newly diagnosed, histologically confirmed, urothelial cell carcinoma of the bladder from 1998 to 2001. Case diagnoses were reviewed and uniformly classified by pathologists following the World Health Organisation/International Society of Urological Pathology 1999 criteria. Controls were hospital-matched patients (n=1149).A total of 1526 candidate variants in 423 candidate genes were analysed. Three distinct subphenotypes were defined according to stage and grade: low-grade nonmuscle   invasive   (n=586), high-grade nonmuscle   invasive   (n=219), and muscle   invasive   (n=246). The association between each variant and subphenotype was assessed by polytomous risk models adjusting for potential confounders. Heterogeneity in genetic susceptibility among subphenotypes was also tested.Two established bladder cancer susceptibility genotypes, NAT2 slow-acetylation and GSTM1-null, exhibited similar associations among the subphenotypes, as did VEGF-rs25648, which was previously identified in our study. Other variants conferred risks for specific tumour subphenotypes such as PMS2-rs6463524 and CD4-rs3213427 (respective heterogeneity p values of 0.006 and 0.004), which were associated with muscle-  invasive   tumours (per-allele odds ratios [95% confidence interval] of 0.56 [0.41-0.77] and 0.71 [0.57-0.88], respectively) but not with non-muscle-  invasive   tumours. Heterogeneity p values were not robust in multiple testing according to their false-discovery rate.These exploratory analyses suggest that genetic susceptibility loci might be related to the molecular/pathologic diversity of bladder cancer. Validation through large-scale replication studies and the study of additional genes and single nucleotide polymorphisms are required. 
 Up-regulation of VEGF, c-fms and COX-2 expression correlates with severity of cervical cancer precursor (CIN) lesions and   invasive   disease. To describe the expression of vascular endothelial growth factor (VEGF), proto-oncogene macrophage colony-stimulating factor receptor (c-fms) and cyclooxygenase-2 (COX-2) in cervical carcinogenesis and to analyze the correlation of VEGF with c-fms and COX-2 expression.In this study, 26 cases of benign cervix, 28 low-grade cervical intraepithelial neoplasia (CIN; CIN 1), 30 high-grade CIN (CIN 2/3) and 28 squamous cervical carcinomas (SCC) were examined by immunohistochemistry (IHC) and analysis was performed separately for epithelium and stroma.Positive epithelial expressions in normal cervix, low-grade CIN, high-grade CIN and SCC were, respectively: VEGF - 11.5%, 39.3%, 53.3% and 75% (P<0.001); c-fms - 0%, 10.7%, 40% and 67.9% (P<0.001); COX-2 - 7.7%, 39.3%, 80% and 100% (P<0.001). Stromal VEGF expression was higher than epithelial expression in all CIN grades and was also associated with the lesion grade, while c-fms and COX-2 stromal expression was weak. VEGF expression was statistically correlated to c-fms and COX-2 expression in high-grade CIN (P=0.020 and P=0.027, respectively) and SCC (P=0.015 and P=0.005, respectively).On the basis of our findings, these factors may participate in the development and progression of CIN lesions, with possible interaction of c-fms and COX-2 on VEGF expression, and may be potential molecular targets for studies of cervical cancer prevention and treatment. 
 Genetic susceptibility to distinct bladder cancer subphenotypes. Clinical, pathologic, and molecular evidence indicate that bladder cancer is heterogeneous with pathologic/molecular features that define distinct subphenotypes with different prognoses. It is conceivable that specific patterns of genetic susceptibility are associated with particular subphenotypes.To examine evidence for the contribution of germline genetic variation to bladder cancer heterogeneity.The Spanish Bladder Cancer/EPICURO Study is a case-control study based in 18 hospitals located in five areas in Spain. Cases were patients with a newly diagnosed, histologically confirmed, urothelial cell carcinoma of the bladder from 1998 to 2001. Case diagnoses were reviewed and uniformly classified by pathologists following the World Health Organisation/International Society of Urological Pathology 1999 criteria. Controls were hospital-matched patients (n=1149).A total of 1526 candidate variants in 423 candidate genes were analysed. Three distinct subphenotypes were defined according to stage and grade: low-grade nonmuscle   invasive   (n=586), high-grade nonmuscle   invasive   (n=219), and muscle   invasive   (n=246). The association between each variant and subphenotype was assessed by polytomous risk models adjusting for potential confounders. Heterogeneity in genetic susceptibility among subphenotypes was also tested.Two established bladder cancer susceptibility genotypes, NAT2 slow-acetylation and GSTM1-null, exhibited similar associations among the subphenotypes, as did VEGF-rs25648, which was previously identified in our study. Other variants conferred risks for specific tumour subphenotypes such as PMS2-rs6463524 and CD4-rs3213427 (respective heterogeneity p values of 0.006 and 0.004), which were associated with muscle-  invasive   tumours (per-allele odds ratios [95% confidence interval] of 0.56 [0.41-0.77] and 0.71 [0.57-0.88], respectively) but not with non-muscle-  invasive   tumours. Heterogeneity p values were not robust in multiple testing according to their false-discovery rate.These exploratory analyses suggest that genetic susceptibility loci might be related to the molecular/pathologic diversity of bladder cancer. Validation through large-scale replication studies and the study of additional genes and single nucleotide polymorphisms are required. 
 Inhibition of the c-fms proto-oncogene autocrine loop and tumor phenotype in glucocorticoid stimulated human breast carcinoma cells. The c-fms proto-oncogene encoded CSF-1 receptor and its ligand represent a feedback loop, which in a paracrine manner, is well known to promote spread of breast cancers. The role of the autocrine feedback loop in promotion of breast tumor behavior, in particular in vitro, is less well understood. The physiologic stimulation of c-fms expression by glucocorticoids (GCs) in vitro and in vivo magnifies the tumor promoting effect seen in these cells from activated c-fms signaling by CSF-1. Targeted molecular therapy against c-fms could therefore abrogate both complementary feedback loops. Using breast cancer cells endogenously co-expressing receptor and ligand, we used complementary approaches to inhibit c-fms expression and function within this autocrine pathway in the context of GC stimulation. Silencing RNA (shRNA), antisense oligonucleotide therapy (AON), and inhibition of c-fms signaling, were all used to quantitate inhibition of GC-stimulated adhesion, motility, and invasion of human breast cancer cells in vitro. shRNA to c-fms downregulated GC-stimulated c-fms mRNA by fourfold over controls, correlating with over twofold reduction in cellular   invasive  ness. AON therapy was also able to inhibit GC stimulation of c-fms mRNA, and resulted in threefold less   invasive  ness and 1.5 to 2-fold reductions in adhesion and motility. Finally, the small-molecule c-fms inhibitor Ki20227 was able to decrease in a dose-response manner, breast cancer cell invasion by up to fourfold. Inhibition of this receptor/ligand pair may have clinical utility in inhibition of the autocrine as well as the known paracrine interactions in breast cancer, thus further supporting use of targeted therapies in this disease. 
 Autocrine CSF1R signaling mediates switching between invasion and proliferation downstream of TGFβ in claudin-low breast tumor cells. Patient data suggest that colony-stimulating factor-1 (CSF1) and its receptor (CSF1R) have critical roles during breast cancer progression. We have previously shown that in human breast tumors expressing both CSF1 and CSF1R, invasion in vivo is dependent both on a paracrine interaction with tumor-associated macrophages and an autocrine regulation of CSF1R in the tumor cells themselves. Although the role of the paracrine interaction between tumor cells and macrophages has been extensively studied, very little is known about the mechanism by which the autocrine CSF1R signaling contributes to tumor progression. We show here that breast cancer patients of the claudin-low subtype have significantly increased expression of CSF1R. Using a panel of breast cancer cell lines, we confirm that CSF1R expression is elevated and regulated by TGFβ specifically in claudin-low cell lines. Abrogation of autocrine CSF1R signaling in MDA-MB-231 xenografts (a claudin-low cell line) leads to increased tumor size by enhanced proliferation, but significantly reduced invasion, dissemination and metastasis. Indeed, we show that proliferation and invasion are oppositely regulated by CSF1R downstream of TGFβ only in claudin-low cell lines. Intravital multiphoton imaging revealed that inhibition of CSF1R in the tumor cells leads to decreased in vivo motility and a more cohesive morphology. We show that, both in vitro and in vivo, CSF1R inhibition results in a reversal of claudin-low marker expression by significant upregulation of luminal keratins and tight-junction proteins such as claudins. Finally, we show that artificial overexpression of claudins in MDA-MB-231 cells is sufficient to tip the cells from   an invas  ive state to a proliferative state. Our results suggest that autocrine CSF1R signaling is essential in maintaining low claudin expression and that it mediates a switch between the proliferative and t  he invas  ive state in claudin-low tumor cells downstream of TGFβ. 
 Autocrine CSF-1R activation promotes Src-dependent disruption of mammary epithelial architecture. Elevated coexpression of colony-stimulating factor receptor (CSF-1R) and its ligand, CSF-1, correlates with   invasive  ness and poor prognosis of a variety of epithelial tumors (Kacinski, B.M. 1995. Ann. Med. 27:79-85). Apart from recruitment of macrophages to the tumor site, the mechanisms by which CSF-1 may potentiate invasion are poorly understood. We show that autocrine CSF-1R activation induces hyperproliferation and a profound, progressive disruption of junctional integrity in acinar structures formed by human mammary epithelial cells in three-dimensional culture. Acini coexpressing receptor and ligand exhibit a dramatic relocalization of E-cadherin from the plasma membrane to punctate intracellular vesicles, accompanied by its loss from the Triton-insoluble fraction. Interfering with Src kinase activity, either by pharmacological inhibition or mutation of the Y561 docking site on CSF-1R, prevents E-cadherin translocation, suggesting that CSF-1R disrupts cell adhesion by uncoupling adherens junction complexes from the cytoskeleton and promoting cadherin internalization through a Src-dependent mechanism. These findings provide a mechanistic basis whereby CSF-1R could contribute to   invasive   progression in epithelial cancers. 
 Recognition of activated CSF-1 receptor in breast carcinomas by a tyrosine 723 phosphospecific antibody. The macrophage colony stimulating factor receptor (CSF-1R), the product of the c-fms proto-oncogene, plays an important role in regulating the normal proliferation and differentiation of macrophages and trophoblasts. However, the abnormal expression of CSF-1R transcripts and protein by human breast carcinomas has been shown to correlate with advanced stage and poor prognosis. Ligand activated CSF-1R dimers transphosphorylate several tyrosines in their cytoplasmic domains which provide recognition sites for various effector proteins in multiple signal transduction pathways. In cells transformed by the c-fms oncogene, one of the major CSF-1R phosphotyrosines, pTyr723 is important for phenotypic expression of anchorage-independent growth and metastasis. In order to investigate the relationship between receptor activation/phosphorylation and cellular phenotypes in vitro and in vivo, we prepared a CSF-1R phosphorylation-state specific antibody raised against a specific phosphopeptide of CSF-1R, which included phosphorylated tyrosine 723. On immunoblots of lysates from cells expressing CSF-1R, this antibody recognizes phosphorylated CSF-IR in CSF-1 stimulated cells but not in unstimulated cells. As an immunohistochemical reagent, this antibody stained 52% of   invasive   human breast tumors (72% of CSF-1R positive cases) in a sample of 114 cases and 38% of carcinoma in situ. This data represents the first direct evidence of in vivo phosphorylation of CSF-1R in human breast carcinomas. 
  [Mm]etastasis  PubMed  
 A unique anti-CD115 monoclonal antibody which inhibits osteolysis and skews human monocyte differentiation from M2-polarized macrophages toward dendritic cells. Cancer progression has been associated with the presence of tumor-associated M2-macrophages (M2-TAMs) able to inhibit anti-tumor immune responses. It is also often associated with   metastasis  -induced bone destruction mediated by osteoclasts. Both cell types are controlled by the CD115 (CSF-1R)/colony-stimulating factor-1 (CSF-1, M-CSF) pathway, making CD115 a promising target for cancer therapy. Anti-human CD115 monoclonal antibodies (mAbs) that inhibit the receptor function have been generated in a number of laboratories. These mAbs compete with CSF-1 binding to CD115, dramatically affecting monocyte survival and preventing osteoclast and macrophage differentiation, but they also block CD115/CSF-1 internalization and degradation, which could lead to potent rebound CSF-1 effects in patients after mAb treatment has ended. We thus generated and selected a non-ligand competitive anti-CD115 mAb that exerts only partial inhibitory effects on CD115 signaling without blocking the internalization or the degradation of the CD115/CSF-1 complex. This mAb, H27K15, affects monocyte survival only minimally, but downregulates osteoclast differentiation and activity. Importantly, it inhibits monocyte differentiation to CD163(+)CD64(+) M2-polarized suppressor macrophages, skewing their differentiation toward CD14(-)CD1a(+) dendritic cells (DCs). In line with this observation, H27K15 also drastically inhibits monocyte chemotactic protein-1 secretion and reduces interleukin-6 production; these two molecules are known to be involved in M2-macrophage recruitment. Thus, the non-depleting mAb H27K15 is a promising anti-tumor candidate, able to inhibit osteoclast differentiation, likely decreasing   metastasis  -induced osteolysis, and able to prevent M2 polarization of TAMs while inducing DCs, hence contributing to the creation of more efficient anti-tumor immune responses. 
 Autocrine CSF1R signaling mediates switching between invasion and proliferation downstream of TGFβ in claudin-low breast tumor cells. Patient data suggest that colony-stimulating factor-1 (CSF1) and its receptor (CSF1R) have critical roles during breast cancer progression. We have previously shown that in human breast tumors expressing both CSF1 and CSF1R, invasion in vivo is dependent both on a paracrine interaction with tumor-associated macrophages and an autocrine regulation of CSF1R in the tumor cells themselves. Although the role of the paracrine interaction between tumor cells and macrophages has been extensively studied, very little is known about the mechanism by which the autocrine CSF1R signaling contributes to tumor progression. We show here that breast cancer patients of the claudin-low subtype have significantly increased expression of CSF1R. Using a panel of breast cancer cell lines, we confirm that CSF1R expression is elevated and regulated by TGFβ specifically in claudin-low cell lines. Abrogation of autocrine CSF1R signaling in MDA-MB-231 xenografts (a claudin-low cell line) leads to increased tumor size by enhanced proliferation, but significantly reduced invasion, dissemination an  d metastas  is. Indeed, we show that proliferation and invasion are oppositely regulated by CSF1R downstream of TGFβ only in claudin-low cell lines. Intravital multiphoton imaging revealed that inhibition of CSF1R in the tumor cells leads to decreased in vivo motility and a more cohesive morphology. We show that, both in vitro and in vivo, CSF1R inhibition results in a reversal of claudin-low marker expression by significant upregulation of luminal keratins and tight-junction proteins such as claudins. Finally, we show that artificial overexpression of claudins in MDA-MB-231 cells is sufficient to tip the cells from an invasive state to a proliferative state. Our results suggest that autocrine CSF1R signaling is essential in maintaining low claudin expression and that it mediates a switch between the proliferative and the invasive state in claudin-low tumor cells downstream of TGFβ. 
 Regulation of non-AU-rich element containing c-fms proto-oncogene expression by HuR in breast cancer. The role of RNA-binding proteins in cancer biology is recognized increasingly. The nucleocytoplasmic shuttling and AU-rich RNA-binding protein HuR stabilizes several cancer-related target mRNAs. The proto-oncogene c-fms, whose 3'untranslated region (3'UTR) is not AU-rich, is associated with poor prognosis in breast cancer. Using a large breast-cancer tissue array (N=670), we found nuclear HuR expression to be associated with nodal   metastasis   and independently with poor survival (P=0.03, RR 1.45), as well as to be co-expressed with c-fms in the breast tumors (P=0.0007). We described c-fms mRNA as a direct target of HuR in vivo, and that HuR bound specifically to a 69-nt region containing 'CUU' motifs in 3'UTR c-fms RNA. Overexpressing or silencing HuR significantly up- or down-regulated c-fms RNA expression, respectively. We also found that known glucocorticoid stimulation of c-fms RNA and protein is largely dependent on the presence of HuR. HuR, by binding to the 69-nt wild type, but not mutant, c-fms sequence can regulate reporter gene expression post-transcriptionally. We are the first to describe that HuR can regulate gene expression by binding non-AU-rich sequences in 3'UTR c-fms RNA. Collectively, our findings suggest that HuR plays a supportive role for c-fms in breast cancer progression by binding a 69-nt element in its 3'UTR, thus regulating its expression. 
 High Expression of Colony-Stimulating Factor 1 Receptor Associates with Unfavorable Cancer-Specific Survival of Patients with Clear Cell Renal Cell Carcinoma. Colony-stimulating factor 1 receptor (CSF-1R), a single-pass type III transmembrane tyrosine-protein kinase, is mainly involved in inflammation and immune regulation to facilitate the progression of solid tumors. This study aimed to evaluate the impact of CSF-1R expression on clinical outcome of patients with clear cell renal cell carcinoma (ccRCC) after surgery.We retrospectively enrolled 268 patients with ccRCC undergoing nephrectomy between 2001 and 2004. Clinicopathologic features and cancer-specific survival (CSS) were collected. Western blot analysis was performed in the pairwise comparisons of CSF-1R expression in peritumor and tumor tissues of patients with ccRCC. Immunohistochemistry was conducted to determine CSF-1R expression level in tumor specimens. Survival analysis was performed by the Kaplan-Meier method. Cox regression models were used to evaluate the impact of prognostic factors on CSS. A concordance index was calculated to measure prognostic accuracy. A prognostic nomogram was constructed on the basis of the identified independent prognostic factors.CSF-1R expression in tumor tissues was higher than in peritumor tissues in 71.4% (5 of 7) patients. CSF-1R expression of tumor tissues was positively associated with   metastasis  , tumor, node,   metastasis   classification system (TNM) stage, Eastern Cooperative Oncology Group performance status score and poor CSS. CSF-1R expression was determined as an independent prognostic factor for CSS in patients with ccRCC. Furthermore, extension of the well-established prognostic models with CSF-1R expression presented significantly improved prognostic accuracy. An efficient prognostic nomogram was constructed on the basis of the independent prognostic factors.High CSF-1R expression is a potential independent adverse prognostic factor for CSS in patients with ccRCC. 
 Recognition of activated CSF-1 receptor in breast carcinomas by a tyrosine 723 phosphospecific antibody. The macrophage colony stimulating factor receptor (CSF-1R), the product of the c-fms proto-oncogene, plays an important role in regulating the normal proliferation and differentiation of macrophages and trophoblasts. However, the abnormal expression of CSF-1R transcripts and protein by human breast carcinomas has been shown to correlate with advanced stage and poor prognosis. Ligand activated CSF-1R dimers transphosphorylate several tyrosines in their cytoplasmic domains which provide recognition sites for various effector proteins in multiple signal transduction pathways. In cells transformed by the c-fms oncogene, one of the major CSF-1R phosphotyrosines, pTyr723 is important for phenotypic expression of anchorage-independent growth and   metastasis  . In order to investigate the relationship between receptor activation/phosphorylation and cellular phenotypes in vitro and in vivo, we prepared a CSF-1R phosphorylation-state specific antibody raised against a specific phosphopeptide of CSF-1R, which included phosphorylated tyrosine 723. On immunoblots of lysates from cells expressing CSF-1R, this antibody recognizes phosphorylated CSF-IR in CSF-1 stimulated cells but not in unstimulated cells. As an immunohistochemical reagent, this antibody stained 52% of invasive human breast tumors (72% of CSF-1R positive cases) in a sample of 114 cases and 38% of carcinoma in situ. This data represents the first direct evidence of in vivo phosphorylation of CSF-1R in human breast carcinomas. 
  [Cc]ell [Ii]nvasion  PubMed  
 Inhibition of the c-fms proto-oncogene autocrine loop and tumor phenotype in glucocorticoid stimulated human breast carcinoma cells. The c-fms proto-oncogene encoded CSF-1 receptor and its ligand represent a feedback loop, which in a paracrine manner, is well known to promote spread of breast cancers. The role of the autocrine feedback loop in promotion of breast tumor behavior, in particular in vitro, is less well understood. The physiologic stimulation of c-fms expression by glucocorticoids (GCs) in vitro and in vivo magnifies the tumor promoting effect seen in these cells from activated c-fms signaling by CSF-1. Targeted molecular therapy against c-fms could therefore abrogate both complementary feedback loops. Using breast cancer cells endogenously co-expressing receptor and ligand, we used complementary approaches to inhibit c-fms expression and function within this autocrine pathway in the context of GC stimulation. Silencing RNA (shRNA), antisense oligonucleotide therapy (AON), and inhibition of c-fms signaling, were all used to quantitate inhibition of GC-stimulated adhesion, motility, and invasion of human breast cancer cells in vitro. shRNA to c-fms downregulated GC-stimulated c-fms mRNA by fourfold over controls, correlating with over twofold reduction in cellular invasiveness. AON therapy was also able to inhibit GC stimulation of c-fms mRNA, and resulted in threefold less invasiveness and 1.5 to 2-fold reductions in adhesion and motility. Finally, the small-molecule c-fms inhibitor Ki20227 was able to decrease in a dose-response manner, breast cancer   cell invasion   by up to fourfold. Inhibition of this receptor/ligand pair may have clinical utility in inhibition of the autocrine as well as the known paracrine interactions in breast cancer, thus further supporting use of targeted therapies in this disease. 
   
  
 SLC22A2 
 
  NCBI Gene  
  GeneRIF  
  Pubmed  
 
 
  Search Term  Rank  Fields  
  [Ii]nvasive  10  PubMed(1)  
 
 
 hide/show details for SLC22A2 
  [Ii]nvasive  PubMed  
 Embryonic stem cells markers SOX2, OCT4 and Nanog expression and their correlations with epithelial-mesenchymal transition in nasopharyngeal carcinoma. Expression of embryonic stem cells (ESCs) markers (SOX2, OCT4, Nanog and Nestin) is crucial for progression of various human malignancies. The purpose of this study was to investigate the expression and prognostic impact of these molecules in nasopharyngeal carcinoma (NPC) patients by immunohistochemistry and immunofluorescence. In the present study, we found that the expression levels of SOX2, OCT4 and Nanog were highly expressed in NPC compared with the non-tumorous tissues. Furthermore, these proteins correlated significantly with several clinicalpathological factors and epithelial-mesenchymal transition (EMT)-associated indicators (E-cadherin/N-cadherin and Snail). In multivariate analyses, high expression of OCT4 (P = 0.013) and Nanog (P = 0.040), but not that of SOX2, was associated with worse survival and had strongly independent prognostic effects. Of note, OCT4 and Nanog were more frequently located   at the   invasive front of tumors, and correlated significantly with various aggressive behaviors including T classification, N classification, M classification and clinical stage. Furthermore, patients with co-expression of OCT4 and Nanog   in the   invasive front had significantly worse survival (P = 0.005). Interestin  gly, at   the invasive front, these molecules correlated significantly with Nestin expression in endothelial cells (P<0.001). These findings provide evidence that ESCs biomarkers OCT4 and Nanog serves as independent prognostic factors for NPC. Additionally, cancer c  ells in   the invasive front of NPC acquiring ESCs-like features should be maintained by vascular niches. 
   
  
 SLC22A23 
 
  NCBI Gene  
  GeneRIF  
  Pubmed  
 
 
  Search Term  Rank  Fields  
  ECM  7  PubMed(1)  
 
 
 hide/show details for SLC22A23 
  ECM  PubMed  
 Investigation of Crohn's disease risk loci in ulcerative colitis further defines their molecular relationship. Identifying shared and disease-specific susceptibility loci for Crohn's disease (CD) and ulcerative colitis (UC) would help define the biologic relationship between the inflammatory bowel diseases. More than 30 CD susceptibility loci have been identified. These represent important candidate susceptibility loci for UC. Loci discovered by the index genome scans in CD have previously been tested for association with UC, but those identified in the recent meta-analysis await such investigation. Furthermore, the recently identified UC locus at   ECM  1 requires formal testing for association with CD.We analyzed 45 single nucleotide polymorphisms, tagging 29 of the loci recently associated with CD in 2527 UC cases and 4070 population controls. We also genotyped the UC-associated   ECM  1 variant rs11205387 in 1560 CD patients and 3028 controls.Nine regions showed association with UC at a threshold corrected for the 29 loci tested (P < .0017). The strongest association (P = 4.13 x 10(-8); odds ratio = 1.27) was identified with a 170-kilobase region on chromosome 1q32 that contains 3 genes. We also found association with JAK2 and replicated a recently reported association with STAT3, further implicating the role of this signaling pathway in inflammatory bowel disease. Additional novel UC susceptibility genes were LYRM4 and CDKAL1. Twenty of the loci were not associated with UC, and several appear to be specific to CD.   ECM  1 variation was not associated with CD.Collectively, these data help define the genetic relationship between CD and UC and characterize common, as well as disease-specific mechanisms of pathogenesis. 
   
  
 TPBG 
 
  NCBI Gene  
  GeneRIF  
  Pubmed  
 
 
  Search Term  Rank  Fields  
  [Mm]igration  6  PubMed(3)  
  [Mm]etastasis  12  PubMed(6); GeneRIFs(1)  
  [Cc]ell [Ii]nvasion  13  PubMed(1)  
 
 
 hide/show details for TPBG 
  [Mm]igration  PubMed  
 Quantitative proteomics identifies a Dab2/integrin module regulating cell   migration  . Clathrin-associated endocytic adapters recruit cargoes to coated pits as a first step in endocytosis. We developed an unbiased quantitative proteomics approach to identify and quantify glycoprotein cargoes for an endocytic adapter, Dab2. Surface levels of integrins beta1, alpha1, alpha2, and alpha3 but not alpha5 or alphav chains were specifically increased on Dab2-deficient HeLa cells. Dab2 colocalizes with integrin beta1 in coated pits that are dispersed over the cell surface, suggesting that it regulates bulk endocytosis of inactive integrins. Depletion of Dab2 inhibits cell   migration   and polarized movement of integrin beta1 and vinculin to the leading edge. By manipulating intracellular and surface integrin beta1 levels, we show that   migration   speed correlates with the intracellular integrin pool but not the surface level. Together, these results suggest that Dab2 internalizes integrins freely diffusing on the cell surface and that Dab2 regulates   migration  , perhaps by maintaining an internal pool of integrins that can be recycled to create new adhesions at the leading edge. 
 5T4 interacts with TIP-2/GIPC, a PDZ protein, with implications for metastasis. Overexpression of the 5T4 transmembrane glycoprotein can have marked effects on both the actin cytoskeleton and cell   migration  . Using a yeast two-hybrid approach, we describe a novel interaction between 5T4 and TIP-2/GIPC, a cytoplasmic interacting protein containing a PDZ domain. The cytoplasmic tail of 5T4 contains a class I PDZ-binding motif (Ser-Asp-Val) and we demonstrate that this region, in particular the terminal valine, is required for 5T4 interaction with TIP-2/GIPC. HeLa cells expressing hemagglutinin-tagged TIP-2/GIPC (HA-TIP-2/GIPC) have an altered distribution of endogenous 5T4, which colocalizes with HA-TIP-2/GIPC, thus supporting an interaction. Furthermore, TIP-2/GIPC can be coimmunoprecipitated with 5T4 from HeLa cell lysates. Identification of the 5T4 and TIP-2/GIPC interaction provides the first link between 5T4 and the actin cytoskeleton. Since other proteins, like 5T4, associate with TIP-2/GIPC and are linked with cancer, we explore the possibility that TIP-2/GIPC may be a common factor involved in the cancer process. 
 Trophoblast glycoprotein promotes pancreatic ductal adenocarcinoma cell metastasis through Wnt/planar cell polarity signaling. Trophoblast glycoprotein (TPBG), a 72 kDa glycoprotein was identified using a monoclonal antibody, which specifically binds human trophoblast. The expression of TPBG in normal tissues is limited; however, it is upregulated in numerous types of cancer. When TPBG is expressed at a high level, this usually indicates a poor clinical outcome. In the present study, it was demonstrated that TPBG was more commonly observed in human pancreatic ductal adenocarcinoma (PDAC) compared with normal pancreatic tissue. Immunohistochemical analysis of PDAC tissue microarrays indicated that the expression of TPBG in PDAC tissues was closely correlated with the tumor-node-metastasis stage of the tumor. Silencing of TPBG in PDAC cell lines resulted in a decreased ability of cancer cell   migration   and invasion. Further investigation demonstrated that the Wnt/planar cell polarity signaling pathway was suppressed, as the expression of Wnt5a and the activation of c-Jun N-terminal kinase was inhibited following TPBG knockdown. In conclusion, the present study provided evidence that TPBG is involved in PDAC metastasis, and that TPBG and its associated signaling pathways may be a suitable target for PDAC therapy. 
  [Mm]etastasis  PubMed  
   Metastasis  -associated 5T4 oncofoetal antigen is concentrated at microvillus projections of the plasma membrane. The 5T4 oncofoetal antigen is a 72 kDa glycoprotein defined by a monoclonal antibody raised against human placental trophoblast and is expressed in many different carcinomas but detected only at low levels in some normal epithelia. Immunohistochemical analysis of the patterns of expression in colorectal carcinomas has indicated a significant association between the presence of the antigen in tumour cells and metastatic spread. A cDNA encoding the 5T4 molecules has been isolated and the extracellular portion contains several leucine-rich repeats which have been implicated in cellular interactions. To study the cell biological role of 5T4 molecules, murine L cells (A9 derivative) were stably transfected with 5T4 cDNA under the control of the CMV immediate-early promoter. The 5T4-expressing cells exhibited a more spindle-shaped morphology compared to the vector alone transfected cells. Confocal immunofluorescence microscopy revealed a 'polkadot' pattern of 5T4 antigen expression, heterogeneous in intensity between cells, but distributed over the entire cell surface. Transmission and scanning electron microscopy showed that the 5T4 antigen is concentrated at microvillus projections of the plasma membrane both in the transfected A9 cells and in various carcinoma cell lines. Such projections express an array of surface molecules which function in cell adhesion and motility. This association of 5T4 antigen with microvillus projections was also observed in various carcinoma cell lines. 5T4 expression in A9 cells was also associated with an altered pattern of cell division, decreased cell-substratum adhesion and increased cellular motility. These results support the hypothesis that 5T4 molecules may have a direct function in trophoblast and tumour cell invasion processes. 
 5T4 interacts with TIP-2/GIPC, a PDZ protein, with implications for   metastasis  . Overexpression of the 5T4 transmembrane glycoprotein can have marked effects on both the actin cytoskeleton and cell migration. Using a yeast two-hybrid approach, we describe a novel interaction between 5T4 and TIP-2/GIPC, a cytoplasmic interacting protein containing a PDZ domain. The cytoplasmic tail of 5T4 contains a class I PDZ-binding motif (Ser-Asp-Val) and we demonstrate that this region, in particular the terminal valine, is required for 5T4 interaction with TIP-2/GIPC. HeLa cells expressing hemagglutinin-tagged TIP-2/GIPC (HA-TIP-2/GIPC) have an altered distribution of endogenous 5T4, which colocalizes with HA-TIP-2/GIPC, thus supporting an interaction. Furthermore, TIP-2/GIPC can be coimmunoprecipitated with 5T4 from HeLa cell lysates. Identification of the 5T4 and TIP-2/GIPC interaction provides the first link between 5T4 and the actin cytoskeleton. Since other proteins, like 5T4, associate with TIP-2/GIPC and are linked with cancer, we explore the possibility that TIP-2/GIPC may be a common factor involved in the cancer process. 
   Metastasis  -associated 5T4 antigen disrupts cell-cell contacts and induces cellular motility in epithelial cells. The 5T4 antigen is defined by a monoclonal antibody (MAb) specific for human trophoblast. It is also expressed by many types of tumour cell and has been associated with   metastasis   and poor clinical outcome in a number of cancers. This pattern of expression is consistent with a mechanistic involvement of 5T4 molecules in the spread of cancer cells. The 5T4 antigen is a transmembrane glycoprotein with a 310 amino acid extracellular domain and a 44 amino acid cytoplasmic domain. Transfection of full-length 5T4 cDNA into epithelial cells alters cell-cell contacts and cellular motility. Thus, in 5T4-transfected CL-S1 murine mammary cells, 5T4 expression is associated with dendritic morphology, accompanied by abrogation of actin/cadherin-containing contacts and increased motility. In transfected MDCK canine kidney epithelial cells, 5T4 over-expression also results in increased motility, but disruption of cell-cell contacts, either by culturing cells in low calcium medium or by addition of HGF/SF, is needed. The effects of 5T4 expression on morphology and motility are separable since cells transfected with a truncated form of 5T4 cDNA in which the cytoplasmic domain is deleted reveal that the latter is necessary to abrogate actin/cadherin-containing contacts but does not influence the effects on motility. Thus, 5T4 molecules can deliver signals through both the extracellular and intracellular domains, and the resultant effects are consistent with a role for 5T4 molecules in invasion processes. 
 Isolation of a cDNA encoding 5T4 oncofetal trophoblast glycoprotein. An antigen associated with   metastasis   contains leucine-rich repeats. The monoclonal antibody 5T4 defines a human oncotrophoblast antigen expressed by a variety of carcinomas but with a restricted pattern of expression in normal adult tissues. The 5T4 antigen has been isolated from term placenta as a 72-kDa glycoprotein consisting of a 42-kDa core protein with extensive N-linked glycosylation. A cDNA has been isolated from a human placental library using pools of oligonucleotides based on amino acid sequence obtained from purified 5T4 molecules. The predicted open reading frame encodes a protein of 420 amino acids with a molecular mass of 46 kDa and 8 potential N-glycosylation sites. There are N- and C-terminal hydrophobic segments corresponding to putative signal and membrane anchorage sequences, respectively. Northern analysis has demonstrated a major 2.5-kilobase mRNA present in cell lines serologically reactive with the monoclonal antibody 5T4. Comparison of the 5T4 protein sequence with current sequence data bases has identified the presence of leucine-rich repeats, which are found in a variety of proteins from yeast, insects, and mammals. The 5T4 antigen expression is strongly associated with   metastasis   in colorectal and gastric cancer, and, hence, the possible functions of the gene product and its relationship to tumor growth and progression are discussed. 
 Organisation of the mouse and human 5T4 oncofoetal leucine-rich glycoprotein genes and expression in foetal and adult murine tissues. The human 5T4 oncotrophoblast leucine-rich glycoprotein may contribute to the process of placentation or   metastasis   by modulating cell adhesion, shape and motility. To understand better the role of 5T4 in development and cancer, the gene structure has been elucidated from both human and mouse genomic clones and mRNA expression has been studied in foetal and adult mouse tissues. The protein coding region is located within the second of two exons, the first exon comprising solely of 5'-untranslated region. Upstream there are no TATA or CAAT boxes, but there are a number of potential Sp1 binding sites. The murine and human proteins show a homologous domain organisation of the leucine rich repeats (LRR) and associated N- and C-terminal flanking regions, although the hydrophilic sequence which intervenes between the two LRR domains contains six additional amino acids in the mouse. The signal peptide, transmembrane region and cytoplasmic tail sequences are identical as are 6 out of the 7 potential N-linked glycosylation sites. Mouse 5T4 transcripts are abundant in placenta and also highly expressed in embryos while in adult tissues transcripts are restricted to brain and ovary. These patterns of expression and the genomic organisation are discussed in relation to possible function and other recently described LRR containing proteins. 
 Trophoblast glycoprotein promotes pancreatic ductal adenocarcinoma cell   metastasis   through Wnt/planar cell polarity signaling. Trophoblast glycoprotein (TPBG), a 72 kDa glycoprotein was identified using a monoclonal antibody, which specifically binds human trophoblast. The expression of TPBG in normal tissues is limited; however, it is upregulated in numerous types of cancer. When TPBG is expressed at a high level, this usually indicates a poor clinical outcome. In the present study, it was demonstrated that TPBG was more commonly observed in human pancreatic ductal adenocarcinoma (PDAC) compared with normal pancreatic tissue. Immunohistochemical analysis of PDAC tissue microarrays indicated that the expression of TPBG in PDAC tissues was closely correlated with the tumor-node-  metastasis   stage of the tumor. Silencing of TPBG in PDAC cell lines resulted in a decreased ability of cancer cell migration and invasion. Further investigation demonstrated that the Wnt/planar cell polarity signaling pathway was suppressed, as the expression of Wnt5a and the activation of c-Jun N-terminal kinase was inhibited following TPBG knockdown. In conclusion, the present study provided evidence that TPBG is involved in PDAC   metastasis  , and that TPBG and its associated signaling pathways may be a suitable target for PDAC therapy. 
  GeneRIFs  
 In conclusion, the present study provided evidence that TPBG is involved in PDAC   metastasis  , and that TPBG and its associated signaling pathways may be a suitable target for pancreatic ductal adenocarcinoma therapy. 
  [Cc]ell [Ii]nvasion  PubMed  
 Metastasis-associated 5T4 oncofoetal antigen is concentrated at microvillus projections of the plasma membrane. The 5T4 oncofoetal antigen is a 72 kDa glycoprotein defined by a monoclonal antibody raised against human placental trophoblast and is expressed in many different carcinomas but detected only at low levels in some normal epithelia. Immunohistochemical analysis of the patterns of expression in colorectal carcinomas has indicated a significant association between the presence of the antigen in tumour cells and metastatic spread. A cDNA encoding the 5T4 molecules has been isolated and the extracellular portion contains several leucine-rich repeats which have been implicated in cellular interactions. To study the cell biological role of 5T4 molecules, murine L cells (A9 derivative) were stably transfected with 5T4 cDNA under the control of the CMV immediate-early promoter. The 5T4-expressing cells exhibited a more spindle-shaped morphology compared to the vector alone transfected cells. Confocal immunofluorescence microscopy revealed a 'polkadot' pattern of 5T4 antigen expression, heterogeneous in intensity between cells, but distributed over the entire cell surface. Transmission and scanning electron microscopy showed that the 5T4 antigen is concentrated at microvillus projections of the plasma membrane both in the transfected A9 cells and in various carcinoma cell lines. Such projections express an array of surface molecules which function in cell adhesion and motility. This association of 5T4 antigen with microvillus projections was also observed in various carcinoma cell lines. 5T4 expression in A9 cells was also associated with an altered pattern of cell division, decreased cell-substratum adhesion and increased cellular motility. These results support the hypothesis that 5T4 molecules may have a direct function in trophoblast and tumour   cell invasion   processes. 
   
  
 ARC 
 
  NCBI Gene  
  GeneRIF  
  Pubmed  
 
 
  Search Term  Rank  Fields  
  [Mm]igration  6  Process(1); PubMed(1)  
  [Ii]nvasive  10  PubMed(1)  
 
 
 hide/show details for ARC 
  [Mm]igration  Process  
 cell   migration   
  PubMed  
 Identification and characterization of a set of conserved and new regulators of cytoskeletal organization, cell morphology and   migration  . Cell   migration   is essential during development and in human disease progression including cancer. Most cell   migration   studies concentrate on known or predicted components of   migration   pathways.Here we use data from a genome-wide RNAi morphology screen in Drosophila melanogaster cells together with bioinformatics to identify 26 new regulators of morphology and cytoskeletal organization in human cells. These include genes previously implicated in a wide range of functions, from mental retardation, Down syndrome and Huntington's disease to RNA and DNA-binding genes. We classify these genes into seven groups according to phenotype and identify those that affect cell   migration  . We further characterize a subset of seven genes, FAM40A, FAM40B, ARC, FMNL3, FNBP3/FBP11, LIMD1 and ZRANB1, each of which has a different effect on cell shape, actin filament distribution and cell   migration  . Interestingly, in several instances closely related isoforms with a single Drosophila homologue have distinct phenotypes. For example, FAM40B depletion induces cell elongation and tail retraction defects, whereas FAM40A depletion reduces cell spreading.Our results identify multiple regulators of cell   migration   and cytoskeletal signalling that are highly conserved between Drosophila and humans, and show that closely related paralogues can have very different functions in these processes. 
  [Ii]nvasive  PubMed  
 Contrast-enhanced magnetic resonance microangiography reveals remodeling of the cerebral microvasculature in transgenic ArcAβ mice. Amyloid-β (Aβ) deposition in the cerebral vasculature is accompanied by remodeling which has a profound influence on vascular integrity and function. In the current study we have quantitatively assessed the age-dependent changes of the cortical vasculature in the arcAβ model of cerebral amyloidosis. To estimate the density of the cortical microvasculature in vivo, we used contrast-enhanced magnetic resonance microangiography (CE-μMRA). Three-dimensional gradient echo datasets with 60 μm isotropic resolution were acquired in 4- and 24-month-old arcAβ mice and compared with wild-type (wt) control mice of the same age before and after administration of superparamagnetic iron oxide nanoparticles. After segmentation of the cortical vasculature from difference images, an automated algorithm was applied for assessing the number and size distribution of intracortical vessels. With CE-μMRA, cerebral arteries and veins with a diameter of less than the nominal pixel resolution (60 μm) can be visualized. A significant age-dependent reduction in the number of functional intracortical microvessels (radii of 20-80 μm) has been observed in 24-month-old arcAβ mice compared with age-matched wt mice, whereas there was no difference between transgenic and wt mice of 4 months of age. Immunohistochemistry demonstrated strong fibrinogen and Aβ deposition in small- and medium-sized vessels, but not in large cerebral arteries, of 24-month-old arcAβ mice. The reduced density of transcortical vessels may thus be attributed to impaired perfusion and vascular occlusion caused by deposition of Aβ and fibrin. The study demonstrated that remodeling of the cerebrovasculature can be   monitor  ed noninvasively with CE-μMRA in mice. 
   
  
 HSD3B1 
 
  NCBI Gene  
  GeneRIF  
  Pubmed  
 
 
  Search Term  Rank  Fields  
  [Ii]nvasive  10  PubMed(4)  
 
 
 hide/show details for HSD3B1 
  [Ii]nvasive  PubMed  
 Genetic susceptibility to distinct bladder cancer subphenotypes. Clinical, pathologic, and molecular evidence indicate that bladder cancer is heterogeneous with pathologic/molecular features that define distinct subphenotypes with different prognoses. It is conceivable that specific patterns of genetic susceptibility are associated with particular subphenotypes.To examine evidence for the contribution of germline genetic variation to bladder cancer heterogeneity.The Spanish Bladder Cancer/EPICURO Study is a case-control study based in 18 hospitals located in five areas in Spain. Cases were patients with a newly diagnosed, histologically confirmed, urothelial cell carcinoma of the bladder from 1998 to 2001. Case diagnoses were reviewed and uniformly classified by pathologists following the World Health Organisation/International Society of Urological Pathology 1999 criteria. Controls were hospital-matched patients (n=1149).A total of 1526 candidate variants in 423 candidate genes were analysed. Three distinct subphenotypes were defined according to stage and grade: low-grade nonmuscle   invasive   (n=586), high-grade nonmuscle   invasive   (n=219), and muscle   invasive   (n=246). The association between each variant and subphenotype was assessed by polytomous risk models adjusting for potential confounders. Heterogeneity in genetic susceptibility among subphenotypes was also tested.Two established bladder cancer susceptibility genotypes, NAT2 slow-acetylation and GSTM1-null, exhibited similar associations among the subphenotypes, as did VEGF-rs25648, which was previously identified in our study. Other variants conferred risks for specific tumour subphenotypes such as PMS2-rs6463524 and CD4-rs3213427 (respective heterogeneity p values of 0.006 and 0.004), which were associated with muscle-  invasive   tumours (per-allele odds ratios [95% confidence interval] of 0.56 [0.41-0.77] and 0.71 [0.57-0.88], respectively) but not with non-muscle-  invasive   tumours. Heterogeneity p values were not robust in multiple testing according to their false-discovery rate.These exploratory analyses suggest that genetic susceptibility loci might be related to the molecular/pathologic diversity of bladder cancer. Validation through large-scale replication studies and the study of additional genes and single nucleotide polymorphisms are required. 
 Genetic susceptibility to distinct bladder cancer subphenotypes. Clinical, pathologic, and molecular evidence indicate that bladder cancer is heterogeneous with pathologic/molecular features that define distinct subphenotypes with different prognoses. It is conceivable that specific patterns of genetic susceptibility are associated with particular subphenotypes.To examine evidence for the contribution of germline genetic variation to bladder cancer heterogeneity.The Spanish Bladder Cancer/EPICURO Study is a case-control study based in 18 hospitals located in five areas in Spain. Cases were patients with a newly diagnosed, histologically confirmed, urothelial cell carcinoma of the bladder from 1998 to 2001. Case diagnoses were reviewed and uniformly classified by pathologists following the World Health Organisation/International Society of Urological Pathology 1999 criteria. Controls were hospital-matched patients (n=1149).A total of 1526 candidate variants in 423 candidate genes were analysed. Three distinct subphenotypes were defined according to stage and grade: low-grade nonmuscle   invasive   (n=586), high-grade nonmuscle   invasive   (n=219), and muscle   invasive   (n=246). The association between each variant and subphenotype was assessed by polytomous risk models adjusting for potential confounders. Heterogeneity in genetic susceptibility among subphenotypes was also tested.Two established bladder cancer susceptibility genotypes, NAT2 slow-acetylation and GSTM1-null, exhibited similar associations among the subphenotypes, as did VEGF-rs25648, which was previously identified in our study. Other variants conferred risks for specific tumour subphenotypes such as PMS2-rs6463524 and CD4-rs3213427 (respective heterogeneity p values of 0.006 and 0.004), which were associated with muscle-  invasive   tumours (per-allele odds ratios [95% confidence interval] of 0.56 [0.41-0.77] and 0.71 [0.57-0.88], respectively) but not with non-muscle-  invasive   tumours. Heterogeneity p values were not robust in multiple testing according to their false-discovery rate.These exploratory analyses suggest that genetic susceptibility loci might be related to the molecular/pathologic diversity of bladder cancer. Validation through large-scale replication studies and the study of additional genes and single nucleotide polymorphisms are required. 
 Localization of type 1 17beta-hydroxysteroid dehydrogenase mRNA and protein in syncytiotrophoblasts and   invasive   cytotrophoblasts in the human term villi. The 17beta-hydroxysteroid dehydrogenases (17beta-HSDs) play a key role in the synthesis of sex steroids. The hallmark of this family of enzymes is the interconversion, through their oxydoreductive reactivity at position C17, of 17-keto- and 17beta-hydroxy-steroids. Because this reaction essentially transforms steroids having low binding activity for the steroid receptor to their more potent 17beta-hydroxysteroids isoforms, it is crucial to the control of the physiological activities of both estrogens and androgens. The human placenta produces large amounts of progesterone and estrogens throughout pregnancy. The placental type 1 17beta-HSD enzyme (E17beta-HSD) catalyzes the reduction of the low activity estrogen, estrone, into the potent estrogen, estradiol. We studied the cell-specific expression of type 1 17beta-HSD in human term placental villous tissue by combining in situ hybridization to localize type 1 17beta-HSD mRNA with immunohistochemistry using an antibody against human placental lactogen, a trophoblast marker. Immunolocalization of E17beta-HSD was also performed. To ascertain whether other steroidogenic enzymes are present in the same cell type, cytochrome P450 cholesterol side-chain cleavage (P450scc), P450 aromatase, and type 1 3beta-hydroxysteroid dehydrogenase (3beta-HSD) were also localized by immunostaining. Our results showed that the syncytium is the major steroidogenic unit of the fetal term villi. In fact, type 1 17beta-HSD mRNA and protein, as well as P450scc, P450 aromatase, and 3beta-HSD immunoreactivities were found in these cells. In addition, our results revealed undoubtedly that extravillous cytotrophoblasts (CTBs), e.g. those from which cell columns of anchoring villous originate, also express the type 1 17beta-HSD gene. However, CTBs lying beneath the syncytial layer, e.g. those from which syncytiotrophoblasts develop, contained barely detectable amounts of type 1 17beta-HSD mRNA as determined by in situ hybridization. These findings, along with those from other laboratories confirm the primordial role of the syncytium in the synthesis of steroids during pregnancy. In addition, our results indicate for the first time that CTBs differentiating along the   invasive   pathway contain type 1 17beta-HSD mRNA. 
 Trifunctional chemical probes for the consolidated detection and identification of enzyme activities from complex proteomes. Chemical probes that covalently modify the active sites of enzymes in complex proteomes are useful tools for identifying enzyme activities associated with discrete (patho) physiological states. Researchers in proteomics typically use two types of activity-based probes to fulfill complementary objectives: fluorescent probes for rapid and sensitive target detection and biotinylated probes for target purification and identification. Accordingly we hypothesized that a strategy in which the target detection and target isolation steps of activity-based proteomic experiments were merged might accelerate the characterization of differentially expressed protein activities. Here we report the synthesis and application of trifunctional chemical proteomic probes in which elements for both target detection (e.g. rhodamine) and isolation (e.g. biotin) are appended to a sulfonate ester reactive group, permitting the consolidated visualization and affinity purification of labeled proteins by a combination of in-gel fluorescence and avidin chromatography procedures. A trifunctional phenyl sulfonate probe was used to identify several technically challenging protein targets, including the integral membrane enzyme 3beta-hydroxysteroid dehydrogenase/Delta5-isomerase and the cofactor-dependent enzymes platelet-type phosphofructokinase and type II tissue transglutaminase. The latter two enzyme activities were significantly up-regulated in the   invasive   estrogen receptor-negative (ER(-)) human breast cancer cell line MDA-MB-231 relative to the non-  invasive   ER(+) breast cancer lines MCF7 and T-47D. Collectively these studies demonstrate that chemical proteomic probes incorporating elements for both target detection and target isolation fortify the important link between the visualization of differentially expressed enzyme activities and their subsequent molecular identification, thereby augmenting the information content achieved in activity-based profiling experiments. 
   
  
 HSD3B2 
 
  NCBI Gene  
  GeneRIF  
  Pubmed  
 
 
  Search Term  Rank  Fields  
  [Mm]igration  6  PubMed(2)  
  [Ii]nvasive  10  PubMed(2)  
  [Mm]etastasis  12  PubMed(1)  
 
 
 hide/show details for HSD3B2 
  [Mm]igration  PubMed  
 Molecular study of the 3 beta-hydroxysteroid dehydrogenase gene type II in patients with hypospadias. To determine whether some patients with idiopathic hypospadias have HSD3B2 mutations, we genotyped this locus in 90 patients with hypospadias (age, 6.0 +/- 0.4 yr) and 101 healthy fertile male controls. We measured basal plasma renin activity and performed an ACTH test for determination of 17-OH-pregnenolone, 17-OH-progesterone, cortisol, dehydroepiandrosterone sulfate, and androstenedione and an human chorionic gonadotropin test for determination of androstenedione, testosterone, and dihydrotestosterone. We did not observe a clear steroidogenic pattern suggestive of 3 beta-HSD deficiency in any patient. DNA was extracted from peripheral lymphocytes; and exons 1, 2, 3, and 4 were amplified by PCR and analyzed by denaturing gradient gel electrophoresis. An abnormal electrophoretic   migration   pattern of exon 4 was observed in five patients. Two patients had missense heterozygous mutations (S213T and S284R). In another three patients, we observed heterozygous nucleotide variants in exon 4 that did not produce a change in amino acids (A238, T259, T320). In vitro enzymatic activity was diminished by 40% and 32% in the S213T and S284R heterozygous mutations, respectively. One control exhibited a heterozygous mutation in exon 3 (V78I), which did not alter in vitro enzyme activity. In addition, we observed possible polymorphisms in intron 1 in four patients and one control. We conclude that subtle molecular abnormalities in the HSD3B2 gene may be observed in some patients with apparent idiopathic hypospadias but that this finding is uncommon. 
 Mutations in the type II 3beta-hydroxysteroid dehydrogenase (HSD3B2) gene can cause premature pubarche in girls. Most previous studies have failed to demonstrate any mutations in the type II 3beta hydroxysteroid dehydrogenase (HSD3B2) gene in patients satisfying the hormonal criteria of nonclassic 3beta-hydroxysteroid dehydrogenase deficiency, suggesting that a mutant 3beta-hydroxysteroid dehydrogenase protein is not the cause of this disorder. We screened the HSD3B2 gene for mutations in girls with premature pubarche and a hormonal diagnosis of 3beta-hydroxysteroid dehydrogenase deficiency.From 30 girls with premature pubarche, we selected 9 whose ACTH-stimulated 17-hydroxypregnenolone levels were elevated (> or =6 SD) and screened the HSD3B2 gene for mutations.All patients were submitted to a standard ACTH stimulation test. Serum steroids were measured and compared to the mean level of pubertal stage matched control subjects. The four exons and exon-intron boundaries of the HSD3B2 gene were amplified by polymerase chain reaction and screened for mutations by denaturing gradient gel electrophoresis. The fragments with abnormal   migration   on denaturing gradient gel electrophoresis were directly sequenced.A homozygous T259M mutation was identified in one girl and a new compound heterozygous G129R/P222H mutation was identified in two sisters. The highest ACTH-stimulated 17-hydroxypregnenolone levels, 147, 339 and 351 nmol/l, were found in those patients with mutations in the HSD3B2 gene. In the patients without mutations, ACTH-stimulated 17-hydroxypregnenolone ranged from 48 to 111 nmol/l. ACTH-stimulated dehydroepiandrosterone levels had an overlap among the girls with and without mutations and the normal controls.Premature pubarche can be caused by mutations in the type II 3beta hydroxysteroid dehydrogenase gene. 
  [Ii]nvasive  PubMed  
 Genetic susceptibility to distinct bladder cancer subphenotypes. Clinical, pathologic, and molecular evidence indicate that bladder cancer is heterogeneous with pathologic/molecular features that define distinct subphenotypes with different prognoses. It is conceivable that specific patterns of genetic susceptibility are associated with particular subphenotypes.To examine evidence for the contribution of germline genetic variation to bladder cancer heterogeneity.The Spanish Bladder Cancer/EPICURO Study is a case-control study based in 18 hospitals located in five areas in Spain. Cases were patients with a newly diagnosed, histologically confirmed, urothelial cell carcinoma of the bladder from 1998 to 2001. Case diagnoses were reviewed and uniformly classified by pathologists following the World Health Organisation/International Society of Urological Pathology 1999 criteria. Controls were hospital-matched patients (n=1149).A total of 1526 candidate variants in 423 candidate genes were analysed. Three distinct subphenotypes were defined according to stage and grade: low-grade nonmuscle   invasive   (n=586), high-grade nonmuscle   invasive   (n=219), and muscle   invasive   (n=246). The association between each variant and subphenotype was assessed by polytomous risk models adjusting for potential confounders. Heterogeneity in genetic susceptibility among subphenotypes was also tested.Two established bladder cancer susceptibility genotypes, NAT2 slow-acetylation and GSTM1-null, exhibited similar associations among the subphenotypes, as did VEGF-rs25648, which was previously identified in our study. Other variants conferred risks for specific tumour subphenotypes such as PMS2-rs6463524 and CD4-rs3213427 (respective heterogeneity p values of 0.006 and 0.004), which were associated with muscle-  invasive   tumours (per-allele odds ratios [95% confidence interval] of 0.56 [0.41-0.77] and 0.71 [0.57-0.88], respectively) but not with non-muscle-  invasive   tumours. Heterogeneity p values were not robust in multiple testing according to their false-discovery rate.These exploratory analyses suggest that genetic susceptibility loci might be related to the molecular/pathologic diversity of bladder cancer. Validation through large-scale replication studies and the study of additional genes and single nucleotide polymorphisms are required. 
 Genetic susceptibility to distinct bladder cancer subphenotypes. Clinical, pathologic, and molecular evidence indicate that bladder cancer is heterogeneous with pathologic/molecular features that define distinct subphenotypes with different prognoses. It is conceivable that specific patterns of genetic susceptibility are associated with particular subphenotypes.To examine evidence for the contribution of germline genetic variation to bladder cancer heterogeneity.The Spanish Bladder Cancer/EPICURO Study is a case-control study based in 18 hospitals located in five areas in Spain. Cases were patients with a newly diagnosed, histologically confirmed, urothelial cell carcinoma of the bladder from 1998 to 2001. Case diagnoses were reviewed and uniformly classified by pathologists following the World Health Organisation/International Society of Urological Pathology 1999 criteria. Controls were hospital-matched patients (n=1149).A total of 1526 candidate variants in 423 candidate genes were analysed. Three distinct subphenotypes were defined according to stage and grade: low-grade nonmuscle   invasive   (n=586), high-grade nonmuscle   invasive   (n=219), and muscle   invasive   (n=246). The association between each variant and subphenotype was assessed by polytomous risk models adjusting for potential confounders. Heterogeneity in genetic susceptibility among subphenotypes was also tested.Two established bladder cancer susceptibility genotypes, NAT2 slow-acetylation and GSTM1-null, exhibited similar associations among the subphenotypes, as did VEGF-rs25648, which was previously identified in our study. Other variants conferred risks for specific tumour subphenotypes such as PMS2-rs6463524 and CD4-rs3213427 (respective heterogeneity p values of 0.006 and 0.004), which were associated with muscle-  invasive   tumours (per-allele odds ratios [95% confidence interval] of 0.56 [0.41-0.77] and 0.71 [0.57-0.88], respectively) but not with non-muscle-  invasive   tumours. Heterogeneity p values were not robust in multiple testing according to their false-discovery rate.These exploratory analyses suggest that genetic susceptibility loci might be related to the molecular/pathologic diversity of bladder cancer. Validation through large-scale replication studies and the study of additional genes and single nucleotide polymorphisms are required. 
  [Mm]etastasis  PubMed  
 Characterization of prostate cancer bone metastases according to expression levels of steroidogenic enzymes and androgen receptor splice variants. Intra-tumoral steroidogenesis and constitutive androgen receptor (AR) activity have been associated with castration-resistant prostate cancer (CRPC). This study aimed to examine if CRPC bone metastases expressed higher levels of steroid-converting enzymes than untreated bone metastases. Steroidogenic enzyme levels were also analyzed in relation to expression of constitutively active AR variants (AR-Vs) and to clinical and pathological variables.Untreated, hormone-naïve (HN, n = 9) and CRPC bone metastases samples (n = 45) were obtained from 54 pat  ients at m  etastasis surgery. Non-malignant and malignant prostate samples were acquired from 13 prostatectomy specimens. Transcript and protein levels were analyzed by real-time RT-PCR, immunohistochemistry and immunoblotting. No differences in steroidogenic enzyme levels were detected between CRPC and HN bone metastases. Significantly higher levels of SRD5A1, AKR1C2, AKR1C3, and HSD17B10 mRNA were however found in bone metastases than in non-malignant and/or malignant prostate tissue, while the CYP11A1, CYP17A1, HSD3B2, SRD5A2, and HSD17B6 mRNA levels in metastases were significantly lower. A sub-group of metastases expressed very high levels of AKR1C3, which was not due to gene amplification as examined by copy number variation assay. No association was found between AKR1C3 expression and nuclear AR staining, tumor cell proliferation or patient outcome after metastases surgery. With only one exception, high AR-V protein levels were found in bone metastases with low AKR1C3 levels, while metastases with high AKR1C3 levels primarily contained low AR-V levels, indicating distinct mechanisms behind castration-resistance in individual bone metastases.Induced capacity of converting adrenal-gland derived steroids into more potent androgens was indicated in a sub-group of PC bone metastases. This was not associated with CRPC but merely with the advanced   stage of m  etastasis. Sub-groups of bone metastases could be identified according to their expression levels of AKR1C3 and AR-Vs, which might be of relevance for patient response to 2(nd) line androgen-deprivation therapy. 
   
  
 SLC6A14 
 
  NCBI Gene  
  GeneRIF  
  Pubmed  
 
 
  Search Term  Rank  Fields  
  [Ee]xtracellular [Mm]atrix  8  PubMed(1)  
  [Mm]etastasis  12  PubMed(1)  
 
 
 hide/show details for SLC6A14 
  [Ee]xtracellular [Mm]atrix  PubMed  
 Proteomic analysis of human parotid gland exosomes by multidimensional protein identification technology (MudPIT). Human ductal saliva contributes over a thousand unique proteins to whole oral fluids. The mechanism by which most of these proteins are secreted by salivary glands remains to be determined. The present study used a mass spectrometry-based, shotgun proteomics approach to explore the possibility that a subset of the proteins found in saliva are derived from exosomes, membrane-bound vesicles of endosomal origin within multivesicular endosomes. Using MudPIT (multidimensional protein identification technology) mass spectrometry, we catalogued 491 proteins in the exosome fraction of human parotid saliva. Many of these proteins were previously observed in ductal saliva from parotid glands (265 proteins). Furthermore, 72 of the proteins in parotid exosomes overlap with those previously identified as urinary exosome proteins, proteins which are also frequently associated with exosomes from other tissues and cell types. Gene Ontology (GO) and KEGG pathway analyses found that cytosolic proteins comprise the largest category of proteins in parotid exosomes (43%), involved in such processes as phosphatidylinositol signaling system, calcium signaling pathway, inositol metabolism, protein export, and signal transduction, among others; whereas the integral plasma membrane proteins and associated/peripheral plasma membrane proteins (26%) were associated with   extracellular matrix  -receptor interaction, epithelial cell signaling, T-cell and B-cell receptor signaling, cytokine receptor interaction, and antigen processing and presentation, among other biological functions. In addition, these putative saliva exosomal proteins were linked to specific diseases (e.g., neurodegenerative disorders, prion disease, cancers, type I and II diabetes). Consequently, parotid glands secrete exosomes that reflect the metabolic and functional status of the gland and may also carry informative protein markers useful in the diagnosis and treatment of systemic diseases. 
  [Mm]etastasis  PubMed  
 Upregulation of the amino acid transporter ATB0,+ (SLC6A14) in colorectal cancer and   metastasis   in humans. ATB(0,+) (SLC6A14) is a Na(+)/Cl(-)-coupled arginine transporter expressed at low levels in normal colon. Arginine is an essential amino acid for tumor cells. Arginine is also the substrate for nitric oxide synthases (NOSs). Since arginine and arginine-derived nitric oxide (NO) play a critical role in cancer, we examined the expression of ATB(0,+) in colorectal cancer. Paired normal and cancer tissues from colectomy specimens of 10 patients with colorectal cancer and from the liver tissue of one patient with hepatic   metastasis   from a colonic primary were used for the analysis of the levels of ATB(0,+) mRNA, inducible NOS (iNOS) mRNA and the corresponding proteins. Tissues samples from the colon, liver, and lymph nodes of an additional patient with metastatic colon cancer were analyzed for ATB(0,+) protein alone. We also examined the levels of nitrotyrosylated proteins. The ATB(0,+) mRNA increased 22.9+/-3.0-fold in colorectal cancer compared to normal tissue and the increase was evident in each of the 10 cases examined. iNOS mRNA increased 5.2+/-1.1-fold in cancer specimens. The changes in mRNA levels were associated with an increase in ATB(0,+), iNOS, and nitrotyrosylated proteins. The increased expression of ATB(0,+) and iNOS was also demonstrated in liver and lymph node specimens with metastases from colonic primaries. This study strongly suggests that the upregulation of ATB(0,+) may have a pathogenic role in colorectal cancer. Since ATB(0,+) is a versatile transporter not only for arginine but also for several drugs including NOS inhibitors, these findings have significant clinical and therapeutic relevance. 
   
  
 RNF2 
 
  NCBI Gene  
  GeneRIF  
  Pubmed  
 
 
  Search Term  Rank  Fields  
  [Mm]igration  6  PubMed(2)  
  [Ii]nvasive  10  PubMed(1)  
  [Mm]etastasis  12  PubMed(5)  
 
 
 hide/show details for RNF2 
  [Mm]igration  PubMed  
 Snail recruits Ring1B to mediate transcriptional repression and cell   migration   in pancreatic cancer cells. Transcriptional repressor Snail is a master regulator of epithelial-mesenchymal transition (EMT), yet the epigenetic mechanism governing Snail to induce EMT is not well understood. Here, we report that in pancreatic ductal adenocarcinoma (PDAC), elevated levels of the ubiquitin E3 ligase Ring1B and Snail, along with elevated monoubiquitination of H2A at K119 (H2AK119Ub1), are highly correlated with poor survival. Mechanistic investigations identified Ring1B as a Snail-interacting protein and showed that the carboxyl zinc fingers of Snail recruit Ring1B and its paralog Ring1A to repress its target promoters. Simultaneous depletion of Ring1A and Ring1B in pancreatic cancer cells decreased Snail binding to the target chromatin, abolished H2AK119Ub1 modification, and thereby compromised Snail-mediated transcriptional repression and cell   migration  . We found that Ring1B and the SNAG-associated chromatin modifier EZH2 formed distinct protein complexes with Snail and that EZH2 was required for Snail-Ring1A/B recruitment to the target promoter. Collectively, our results unravel an epigenetic mechanism underlying transcriptional repression by Snail, suggest Ring1A/B as a candidate therapeutic target, and identify H2AK119Ub1 as a potential biomarker for PDAC diagnosis and prognosis. 
 The Polycomb group protein RING1B is overexpressed in ductal breast carcinoma and is required to sustain FAK steady state levels in breast cancer epithelial cells. In early stages of metastasis malignant cells must acquire phenotypic changes to enhance their migratory behavior and their ability to breach the matrix surrounding tumors and blood vessel walls. Epigenetic regulation of gene expression allows the acquisition of these features that, once tumoral cells have escape from the primary tumor, can be reverted. Here we report that the expression of the Polycomb epigenetic repressor Ring1B is enhanced in tumoral cells that invade the stroma in human ductal breast carcinoma and its expression is coincident with that of Fak in these tumors. Ring1B knockdown in breast cancer cell lines revealed that Ring1B is required to sustain Fak expression in basal conditions as well as in Tgfβ-treated cells. Functionally, endogenous Ring1B is required for cell   migratio  n and invasion in vitro and for in vivo invasion of the mammary fat pad by tumoral cells. Finally we identify p63 as a target of Ring1B to regulate Fak expression: Ring1B depletion results in enhanced p63 expression, which in turns represses Fak expression. Importantly, Fak downregulation upon Ring1B depletion is dependent on p63 expression. Our findings provide new insights in the biology of the breast carcinoma and open new avenues for breast cancer prognosis and therapy. 
  [Ii]nvasive  PubMed  
 Dual Roles of RNF2 in Melanoma Progression. Epigenetic regulators have emerged as critical factors governing the biology of cancer. Here, in the context of melanoma, we show that RNF2 is prognostic, exhibiting progression-correlated expression in human melanocytic neoplasms. Through a series of complementary gain-of-function and loss-of-function studies in mouse and human systems, we establish that RNF2 is oncogenic and prometastatic. Mechanistically, RNF2-mediated   invasive   behavior is dependent on its ability to monoubiquitinate H2AK119 at the promoter of LTBP2, resulting in silencing of this negative regulator of TGFβ signaling. In contrast, RNF2's oncogenic activity does not require its catalytic activity nor does it derive from its canonical gene repression function. Instead, RNF2 drives proliferation through direct transcriptional upregulation of the cell-cycle regulator CCND2. We further show that MEK1-mediated phosphorylation of RNF2 promotes recruitment of activating histone modifiers UTX and p300 to a subset of poised promoters, which activates gene expression. In summary, RNF2 regulates distinct biologic processes in the genesis and progression of melanoma via different molecular mechanisms.The role of epigenetic regulators in cancer progression is being increasingly appreciated. We show novel roles for RNF2 in melanoma tumorigenesis and metastasis, albeit via different mechanisms. Our findings support the notion that epigenetic regulators, such as RNF2, directly and functionally control powerful gene networks that are vital in multiple cancer processes. 
  [Mm]etastasis  PubMed  
 Microarray analysis identifies a death-from-cancer signature predicting therapy failure in patients with multiple types of cancer. Activation in transformed cells of normal stem cells' self-renewal pathways might contribute to the survival life cycle of cancer stem cells and promote tumor progression. The BMI-1 oncogene-driven gene expression pathway is essential for the self-renewal of hematopoietic and neural stem cells. We applied a mouse/human comparative translational genomics approach to identify an 11-gene signature that consistently displays a stem cell-resembling expression profile in distant metastatic lesions as revealed by the analysis of metastases and primary tumors from a transgenic mouse model of prostate cancer and cancer patients. To further validate these results, we examined the prognostic power of the 11-gene signature in several independent therapy-outcome sets of clinical samples obtained from 1,153 cancer patients diagnosed with 11 different types of cancer, including 5 epithelial malignancies (prostate, breast, lung, ovarian, and bladder cancers) and 5 nonepithelial malignancies (lymphoma, mesothelioma, medulloblastoma, glioma, and acute myeloid leukemia). Kaplan-Meier analysis demonstrated that a stem cell-like expression profile of the 11-gene signature in primary tumors is a consistent powerful predictor of a short interval to disease recurrence, distant   metastasis  , and death after therapy in cancer patients diagnosed with 11 distinct types of cancer. These data suggest the presence of a conserved BMI-1-driven pathway, which is similarly engaged in both normal stem cells and a highly malignant subset of human cancers diagnosed in a wide range of organs and uniformly exhibiting a marked propensity toward metastatic dissemination as well as a high probability of unfavorable therapy outcome. 
 Microarray analysis identifies a death-from-cancer signature predicting therapy failure in patients with multiple types of cancer. Activation in transformed cells of normal stem cells' self-renewal pathways might contribute to the survival life cycle of cancer stem cells and promote tumor progression. The BMI-1 oncogene-driven gene expression pathway is essential for the self-renewal of hematopoietic and neural stem cells. We applied a mouse/human comparative translational genomics approach to identify an 11-gene signature that consistently displays a stem cell-resembling expression profile in distant metastatic lesions as revealed by the analysis of metastases and primary tumors from a transgenic mouse model of prostate cancer and cancer patients. To further validate these results, we examined the prognostic power of the 11-gene signature in several independent therapy-outcome sets of clinical samples obtained from 1,153 cancer patients diagnosed with 11 different types of cancer, including 5 epithelial malignancies (prostate, breast, lung, ovarian, and bladder cancers) and 5 nonepithelial malignancies (lymphoma, mesothelioma, medulloblastoma, glioma, and acute myeloid leukemia). Kaplan-Meier analysis demonstrated that a stem cell-like expression profile of the 11-gene signature in primary tumors is a consistent powerful predictor of a short interval to disease recurrence, distant   metastasis  , and death after therapy in cancer patients diagnosed with 11 distinct types of cancer. These data suggest the presence of a conserved BMI-1-driven pathway, which is similarly engaged in both normal stem cells and a highly malignant subset of human cancers diagnosed in a wide range of organs and uniformly exhibiting a marked propensity toward metastatic dissemination as well as a high probability of unfavorable therapy outcome. 
 H2AK119Ub1 and H3K27Me3 in molecular staging for survival prediction of patients with pancreatic ductal adenocarcinoma. Polycomb group (PcG) proteins Ring1B and EZH2, which have been characterized as catalyzing the two epigenetic modifications H2AK119 monoubiquitination (H2AK119Ub1) and H3K27 trimethylation (H3K27Me3), are well-known epigenetic silencers implicated in embryonic development and tumorigenesis. However, the status of polycomb-associated histone modifications and their clinical implications in pancreatic cancer remain unclear. Here, we performed immunohistochemistry on tissue microarrays (TMAs) containing 80 pairs of human pancreatic cancer specimens to assess the expression levels of Ring1B, H2AK119Ub1, EZH2, and H3K27Me3 in tumors. More than 50% of the tumor cells showed a high expression of H2AK119Ub1, Ring1B, and EZH2, whereas more than 50% of the tumor cells showed a low level of H3K27Me3. Different expression patterns of H2AK119Ub1 and H3K27Me3 in tumors were negatively correlated (r = -0.247, P = 0.027). Both H2AK119Ub1 and H3K27Me3 independently predicted the clinical prognosis. In particular, a combinatorial pattern of elevated H2AK119Ub1 and decreased H3K27Me3 in tumors was significantly correlated with a poorer prognosis. Furthermore, compared to the tumor, lymph node,   metastasis   (TNM) staging system, histone modifications can discriminate the survival difference more accurately, especially for patients with stage I or stage II tumors. Simultaneous silencing of Ring1B and EZH2 via shRNA depleted H2AK119Ub1 and H3K27Me3 in the pancreatic cancer cells PanC1 and AsPC1, enhanced HOX gene derepression, and inhibited tumor cell growth in vitro and in tumor xenograft models. These results demonstrated that H2AK119Ub1 and H3K27Me3 cooperate in tumors and are associated with the clinical prognosis in combinatorial patterns. We have proposed that epigenetic modifications may serve as discriminatory biomarkers for molecular staging of pancreatic cancer. 
 Dual Roles of RNF2 in Melanoma Progression. Epigenetic regulators have emerged as critical factors governing the biology of cancer. Here, in the context of melanoma, we show that RNF2 is prognostic, exhibiting progression-correlated expression in human melanocytic neoplasms. Through a series of complementary gain-of-function and loss-of-function studies in mouse and human systems, we establish that RNF2 is oncogenic and prometastatic. Mechanistically, RNF2-mediated invasive behavior is dependent on its ability to monoubiquitinate H2AK119 at the promoter of LTBP2, resulting in silencing of this negative regulator of TGFβ signaling. In contrast, RNF2's oncogenic activity does not require its catalytic activity nor does it derive from its canonical gene repression function. Instead, RNF2 drives proliferation through direct transcriptional upregulation of the cell-cycle regulator CCND2. We further show that MEK1-mediated phosphorylation of RNF2 promotes recruitment of activating histone modifiers UTX and p300 to a subset of poised promoters, which activates gene expression. In summary, RNF2 regulates distinct biologic processes in the genesis and progression of melanoma via different molecular mechanisms.The role of epigenetic regulators in cancer progression is being increasingly appreciated. We show novel roles for RNF2 in melanoma tumorigenesis and   metastasi  s, albeit via different mechanisms. Our findings support the notion that epigenetic regulators, such as RNF2, directly and functionally control powerful gene networks that are vital in multiple cancer processes. 
 The Polycomb group protein RING1B is overexpressed in ductal breast carcinoma and is required to sustain FAK steady state levels in breast cancer epithelial cells. In early stages of   metastasis   malignant cells must acquire phenotypic changes to enhance their migratory behavior and their ability to breach the matrix surrounding tumors and blood vessel walls. Epigenetic regulation of gene expression allows the acquisition of these features that, once tumoral cells have escape from the primary tumor, can be reverted. Here we report that the expression of the Polycomb epigenetic repressor Ring1B is enhanced in tumoral cells that invade the stroma in human ductal breast carcinoma and its expression is coincident with that of Fak in these tumors. Ring1B knockdown in breast cancer cell lines revealed that Ring1B is required to sustain Fak expression in basal conditions as well as in Tgfβ-treated cells. Functionally, endogenous Ring1B is required for cell migration and invasion in vitro and for in vivo invasion of the mammary fat pad by tumoral cells. Finally we identify p63 as a target of Ring1B to regulate Fak expression: Ring1B depletion results in enhanced p63 expression, which in turns represses Fak expression. Importantly, Fak downregulation upon Ring1B depletion is dependent on p63 expression. Our findings provide new insights in the biology of the breast carcinoma and open new avenues for breast cancer prognosis and therapy. 
   
  
 BRCA1 
 
  NCBI Gene  
  GeneRIF  
  Pubmed  
 
 
  Search Term  Rank  Fields  
  [Mm]igration  6  PubMed(17); GeneRIFs(1)  
  ECM  7  PubMed(1)  
  [Ee]xtracellular [Mm]atrix  8  PubMed(4); GeneRIFs(2)  
  [Ii]nvasive  10  PubMed(154); GeneRIFs(14)  
  [Mm]etastasize  11  PubMed(2); GeneRIFs(1)  
  [Mm]etastasis  12  PubMed(60); GeneRIFs(9)  
  [Cc]ell [Ii]nvasion  13  PubMed(1)  
 
 
 hide/show details for BRCA1 
  [Mm]igration  PubMed  
 Interaction with BRCA2 suggests a role for filamin-1 (hsFLNa) in DNA damage response. The BRCA2 tumor suppressor plays significant roles in DNA damage response. The human actin binding protein filamin-1 (hsFLNa, also known as ABP-280) participates in orthogonal actin network, cellular stress responses, signal transduction, and cell   migration  . Through a yeast two-hybrid system, an in vitro binding assay, and in vivo co-immunoprecipitations, we identified an interaction between BRCA2 and hsFLNa. The hsFLNa binding domain of BRCA2 was mapped to an internal conserved region, and the BRCA2-interacting domain of hsFLNa was mapped to its C terminus. Although hsFLNa is known for its cytoplasmic functions in cell   migration   and signal transduction, some hsFLNa resides in the nucleus, raising the possibility that it participates in DNA damage response through a nuclear interaction with BRCA2. Lack of hsFLNa renders a human melanoma cell line (M2) more sensitive to several genotoxic agents including gamma irradiation, bleomycin, and ultraviolet-c light. These results suggest that BRCA2/hsFLNa interaction may serve to connect cytoskeletal signal transduction to DNA damage response pathways. 
 The frequency of founder mutations in the BRCA1, BRCA2, and APC genes in Australian Ashkenazi Jews: implications for the generality of U.S. population data. Several studies have shown that Ashkenazi Jews in the United States and Israel have a high prevalence of the founder mutations BRCA1 185delAG, BRCA1 5382insC, BRCA2 6174delT, and APC I1307K at frequencies of 1.0--1.1%, 0.2--0.3%, 0.6--1.4%, and 6.1--7.0%, respectively. The objective of this study was to compare the prevalence of these alleles in the Australian Jewish population with that of U.S. Jews. Australian Jews have a different history of   migration  , with less opportunity for changes in allele frequency due to conversion or intermarriage with non-Jewish Australians. The results obtained therefore can be used to assess whether U.S. data can be generalized to other Jewish populations. SUBJECTS AND METHODS. Subject samples were ascertained through a screening program for Tay-Sachs disease as part of a community-based screening program in New South Wales and Victoria. DNA extracted from 1200 deidentified blood samples was tested using amplification refractory mutation system polymerase chain reaction.The allele frequencies found were as follows: BRCA1 185delAG 1.25% (95% confidence interval [CI], 0.62--1.88%), BRCA1 5382insC 0.25% (95% CI, 0--0.53%), BRCA2 6174delT 1.08% (95% CI, 0.50--1.67%), and APC I1307K 8.67% (95% CI, 7.07--10.26%). The prevalence of breast carcinoma predisposition alleles therefore is greater than 2.5% in Australian Ashkenazim.There were no significant differences between the allele frequencies in Australian Ashkenazim and those identified in other studies with similar ascertainment strategies, despite the different   migration   patterns of Australian Jews. This suggests the broad applicability of the U.S. and Israeli data, not only to Australian Ashkenazim, but also to Ashkenazi communities throughout the world. 
 The frequency of founder mutations in the BRCA1, BRCA2, and APC genes in Australian Ashkenazi Jews: implications for the generality of U.S. population data. Several studies have shown that Ashkenazi Jews in the United States and Israel have a high prevalence of the founder mutations BRCA1 185delAG, BRCA1 5382insC, BRCA2 6174delT, and APC I1307K at frequencies of 1.0--1.1%, 0.2--0.3%, 0.6--1.4%, and 6.1--7.0%, respectively. The objective of this study was to compare the prevalence of these alleles in the Australian Jewish population with that of U.S. Jews. Australian Jews have a different history of   migration  , with less opportunity for changes in allele frequency due to conversion or intermarriage with non-Jewish Australians. The results obtained therefore can be used to assess whether U.S. data can be generalized to other Jewish populations. SUBJECTS AND METHODS. Subject samples were ascertained through a screening program for Tay-Sachs disease as part of a community-based screening program in New South Wales and Victoria. DNA extracted from 1200 deidentified blood samples was tested using amplification refractory mutation system polymerase chain reaction.The allele frequencies found were as follows: BRCA1 185delAG 1.25% (95% confidence interval [CI], 0.62--1.88%), BRCA1 5382insC 0.25% (95% CI, 0--0.53%), BRCA2 6174delT 1.08% (95% CI, 0.50--1.67%), and APC I1307K 8.67% (95% CI, 7.07--10.26%). The prevalence of breast carcinoma predisposition alleles therefore is greater than 2.5% in Australian Ashkenazim.There were no significant differences between the allele frequencies in Australian Ashkenazim and those identified in other studies with similar ascertainment strategies, despite the different   migration   patterns of Australian Jews. This suggests the broad applicability of the U.S. and Israeli data, not only to Australian Ashkenazim, but also to Ashkenazi communities throughout the world. 
 EZH2 participates in malignant biological behavior of epithelial ovarian cancer through regulating the expression of BRCA1. Aberrant overexpression of the enhancer of zeste homolog 2 (EZH2), a histone methyltransferase inhibiting targets expression via epigenetic mechanisms, is associated with an invasive phenotype and drug resistance in ovarian cancer. Breast cancer 1 (BRCA1) gene is a well-recognized tumor suppressor, whose downregulation plays a key role in the development of ovarian cancer. In the present study, we found depletion of EZH2 increased BRCA1 protein expression and promoted its nuclear translocation, but decreased BRCA1 mRNA expression. Treatment with the Akt-1 activator insulin-like growth factor-1 (IGF-1) prevented EZH2-induced BRCA1 nuclear/cytoplasmic shuttling. Loss of BRCA1 partially rescued the effects of EZH2 downregulation on proliferation, G 2/M transition, and   migration   in ovarian cancer cells. However, in a cisplatin-resistant sub-line of A2780 (A2780/DDP), both EZH2 and BRCA1 were overexpressed compared with parental A2780 cells and depletion of EZH2 reduced BRCA1 expression at both mRNA and protein levels. Downregulation of EZH2 or BRCA1 sensitized A2780/DDP cells to cisplatin, whereas simultaneous inhibition of them only resulted in modest resensitization instead of showing any synergistic effect because EZH2 expression was reactivated when BRCA1 expression was very low. Accordingly, our results suggest the expression of BRCA1 is modulated by EZH2 in epithelial ovarian cancer and BRCA1 is required for the effects of EZH2 downregulation on biological behaviors of tumor cells. 
 BRCA1 is a negative modulator of the PRC2 complex. The Polycomb-repressive complex 2 (PRC2) is important for maintenance of stem cell pluripotency and suppression of cell differentiation by promoting histone H3 lysine 27 trimethylation (H3K27me3) and transcriptional repression of differentiation genes. Here we show that the tumour-suppressor protein BRCA1 interacts with the Polycomb protein EZH2 in mouse embryonic stem (ES) and human breast cancer cells. The BRCA1-binding region in EZH2 overlaps with the noncoding RNA (ncRNA)-binding domain, and BRCA1 expression inhibits the binding of EZH2 to the HOTAIR ncRNA. Decreased expression of BRCA1 causes genome-wide EZH2 re-targeting and elevates H3K27me3 levels at PRC2 target loci in both mouse ES and human breast cancer cells. BRCA1 deficiency blocks ES cell differentiation and enhances breast cancer   migration   and invasion in an EZH2-dependent manner. These results reveal that BRCA1 is a key negative modulator of PRC2 and that loss of BRCA1 inhibits ES cell differentiation and enhances an aggressive breast cancer phenotype by affecting PRC2 function. 
 Suppression of breast cancer invasion and   migration   by indole-3-carbinol: associated with up-regulation of BRCA1 and E-cadherin/catenin complexes. Indole-3-carbinol (I3C) is a compound occurring naturally in cruciferous vegetables and has been indicated as a promising agent in preventing breast cancer development and progression. In the present study we have investigated the effect of I3C on the cell   migration   and invasion behavior in estrogen receptor positive MCF-7 and estrogen receptor negative MDA-MB-468 human breast cancer cell lines. Both MCF-7 and MDA-MB-468 were poorly invasive cell lines and exhibited modest invasion and   migration   capacity in the presence of fibronectin as the chemoattractant. I3C (50 or 100 microM) elicited a significant inhibition of in vitro cell adhesion,   migration  , and invasion as well as in vivo lung metastasis formation in both cell lines. I3C also suppressed the 17beta-estradiol stimulated   migration   and invasion in estrogen-responsive MCF-7 cells. These results indicate that anti-invasion and anti  migration   activities of I3C occur via estrogen-independent and estrogen-dependent pathways. Moreover, I3C significantly caused a dose-dependent increase in E-cadherin, three major catenins (alpha, beta, and gamma-catenin) and BRCA1 expression. Our current finding is the first demonstration that I3C can activate the function of invasion suppressor molecules associated with the suppression of invasion and   migration   in breast cancer cells. Thus, clinical application of I3C may contribute to the potential benefit for suppression of breast cancer invasion and metastasis. 
 The ubiquitin-interacting motif containing protein RAP80 interacts with BRCA1 and functions in DNA damage repair response. In this study, we examine the potential role of receptor-associated protein 80 (RAP80), a nuclear protein containing two ubiquitin-interacting motifs (UIM), in DNA damage response and double-strand break (DSB) repair. We show that following ionizing radiation and treatment with DNA-damaging agents, RAP80 translocates to discrete nuclear foci that colocalize with those of gamma-H2AX. The UIMs and the region of amino acids 204 to 304 are critical for the relocalization of RAP80 to ionizing radiation-induced foci (IRIF). These observations suggest that RAP80 becomes part of a DNA repair complex at the sites of IRIF. We also show that RAP80 forms a complex with the tumor repressor BRCA1 and that this interaction is mediated through the BRCA1 COOH-terminal repeats of BRCA1. The UIMs are not required for the interaction of RAP80 with BRCA1. Knockdown of RAP80 in HEK293 cells significantly reduced DSB-induced homology-directed recombination (HDR). Moreover, inhibition of RAP80 expression by small interfering RNA increased radiosensitivity, whereas increased radioresistance was observed in human breast cancer MCF-7 cells with overexpression of RAP80. Taken together, our data suggest that RAP80 plays an important role in DNA damage response signaling and HDR-mediated DSB repair. We further show that RAP80 can function as a substrate of the ataxia-telangiectasia mutated protein kinase in vitro, which phosphorylates RAP80 at Ser(205) and Ser(402). We show that this phosphorylation is not required for the   migration   of RAP80 to IRIF. 
 BRCA1 regulates follistatin function in ovarian cancer and human ovarian surface epithelial cells. Follistatin (FST), a folliculogenesis regulating protein, is found in relatively high concentrations in female ovarian tissues. FST acts as an antagonist to Activin, which is often elevated in human ovarian carcinoma, and thus may serve as a potential target for therapeutic intervention against ovarian cancer. The breast cancer susceptibility gene 1 (BRCA1) is a known tumor suppressor gene in human breast cancer; however its role in ovarian cancer is not well understood. We performed microarray analysis on human ovarian carcinoma cell line SKOV3 that stably overexpress wild-type BRCA1 and compared with the corresponding empty vector-transfected clones. We found that stable expression of BRCA1 not only stimulates FST secretion but also simultaneously inhibits Activin expression. To determine the physiological importance of this phenomenon, we further investigated the effect of cellular BRCA1 on the FST secretion in immortalized ovarian surface epithelial (IOSE) cells derived from either normal human ovaries or ovaries of an ovarian cancer patient carrying a mutation in BRCA1 gene. Knock-down of BRCA1 in normal IOSE cells demonstrates down-regulation of FST secretion along with the simultaneous up-regulation of Activin expression. Furthermore, knock-down of FST in IOSE cell lines as well as SKOV3 cell line showed significantly reduced cell proliferation and decreased cell   migration   when compared with the respective controls. Thus, these findings suggest a novel function for BRCA1 as a regulator of FST expression and function in human ovarian cells. 
 Association between cytosolic expression of BRCA1 and metastatic risk in breast cancer. Although BRCA1 has been extensively studied for its role as a tumour-suppressor protein, the role of BRCA1 subcellular localisation in oncogenesis and tumour progression has remained unclear. This study explores the impact of BRCA1 mislocalisation on clinical outcomes in breast cancer.Tissue microarrays assembled from a cohort of patients with all stages of breast cancer were analysed for BRCA1 localisation and correlated with patient survival. Tissue microarrays of patients who had breast cancer that had metastasised to the lung were assembled from an independent cohort of patients. These were analysed for BRCA1 subcellular expression. In vitro studies using cultured human breast cancer cells were conducted to examine the effect of cytosolic BRCA1 on cell   migration   and efficiency of invasion.An inverse association was found between cytosolic BRCA1 expression and metastasis-free survival in patients aged >40 years. Further analysis of BRCA1 subcellular expression in a cohort of breast cancer patients with metastatic disease revealed that the cytosolic BRCA1 content of breast tumours that had metastasised to the lung was 36.0% (95% CI=(31.7%, 40.3%), which was markedly higher than what is reported in the literature (8.2-14.8%). Intriguingly, these lung metastases and their corresponding primary breast tumours demonstrated similarly high cytosolic BRCA1 distributions in both paired and unpaired analyses. Finally, in vitro studies using human breast cancer cells demonstrated that genetically induced BRCA1 cytosolic sequestration (achieved using the cytosol-sequestering BRCA1 5382insC mutation) increased cell invasion efficiency.Results from this study suggest a model where BRCA1 cytosolic mislocalisation promotes breast cancer metastasis, making it a potential biomarker of metastatic disease. 
 CAF-like state in primary skin fibroblasts with constitutional BRCA1 epimutation sheds new light on tumor suppressor deficiency-related changes in healthy tissue. Constitutive epimutations of tumor suppressor genes are increasingly considered as cancer predisposing factors equally to sequence mutations. In light of the emerging role of the microenvironment for cancer predisposition, initiation, and progression, we aimed to characterize the consequences of a BRCA1 epimutation in cells of mesenchymal origin. We performed a comprehensive molecular and cellular comparison of primary dermal fibroblasts taken from a monozygous twin pair discordant for recurrent cancers and BRCA1 epimutation, whose exceptional clinical case we previously reported in this journal. Comparative transcriptome analysis identified differential expression of extracellular matrix-related genes and pro-tumorigenic growth factors, such as collagens and CXC chemokines. Moreover, genes known to be key markers of so called cancer-associated fibroblasts (CAFs), such as ACTA2, FAP, PDPN, and TNC, were upregulated in fibroblasts of the affected twin (BRCA1(mosMe)) in comparison to those of the healthy twin (BRCA1(wt)). Further analyses detected CAF-typical cellular features, including an elevated growth rate, enhanced   migration  , altered actin architecture and increased production of ketone bodies in BRCA1(mosMe) fibroblasts compared to BRCA1(wt) fibroblasts. In addition, conditioned medium of BRCA1(mosMe) fibroblasts was more potent than conditioned medium of BRCA1(wt) fibroblasts to promote cell proliferation in an epithelial and a cancer cell line. Our data demonstrate, that a CAF-like state is not an exclusive feature of tumor-associated tissue but also exists in healthy tissue with tumor suppressor deficiency. The naturally occurring phenomenon of twin fibroblasts differing in their BRCA1 methylation status revealed to be a unique powerful tool for exploring tumor suppressor deficiency-related changes in healthy tissue, reinforcing their significance for cancer predisposition. 
 Incidence of BRCA1 and BRCA2 mutations in 54 Chilean families with breast/ovarian cancer, genotype-phenotype correlations. Our aim was to analyze the incidence of mutations in BRCA1 and BRCA2 genes in 54 families with breast/ovarian cancer. Families were selected from three Institutions following the standard criteria for hereditary breast/ovarian cancer. PCR amplification of all exons was performed, followed by SSCP, heteroduplex, PTT and sequencing analysis. We identified eight truncation mutations, three in the BRCA1 gene and five in the BRCA2 gene. Three of these mutations have not been reported previously by other groups: 308insA in one family, 3936 C>T in two families, for BRCA1, and 4970insTG in one family for BRCA2. In addition two families having Ashkenazi Jewish ancestors present the well known mutations 185delAG and 6174delT. Interestingly, 5 out of 11 families have mutations recurrent in Spanish families. Among the 54 families selected, seven have breast and ovary cancer cases, and only two presented a mutation in BRCA1 or BRCA2 genes. Other cancers as prostate and stomach are frequent among relatives carrying the mutation. Five cases of very early onset (<31 years old) breast cancer were detected. The frequencies of BRCA1 (0.074) and BRCA2 (0.13) mutations in our families is low but similar to the incidence found in other populations, like in Spain. Since is widely known that risk factors that modulate the development of breast cancer such as lifestyle risk factors, geographic location, country of origin and socioeconomic status, besides a familial history of breast cancer our findings suggest that the history of colonization and im  migration  s is very relevant when studying hereditary factors associated to breast cancer. 
 Mutation screening of the BRCA1 gene in sporadic breast cancer in southern Chinese populations. The comparison of freshly collected breast cancer tissue DNA with that of the matched blood specimens of 144 patients was studied, and breast tissue from 30 unrelated normal women without cancer was selected as controls. The entire BRCA1 coding sequence was amplified by PCR with primers especially designed for comprehensive mutation screening by single-strand conformation polymorphism (SSCP) analysis. Amplification and electrophoresis were repeated for the confirmation of altered   migration   patterns. Variant bands were subsequently cut from gels, resuspended in distilled water, subjected again to PCR, and then sequenced. The amplified fragments of exon 2 and exon 3, which were not suitable for SSCP (>300bp) were sequenced directly. A total of 20 nucleotide alterations were observed in the breast cancer tissue DNA, and all were single-base substitutions. Sixteen missense mutations (which change the coding from one amino acid to another) have been identified throughout the gene. Ten cases of single nucleotide changes in BRCA1 detected in the study without records in the BIC database consisted of two missense mutations in exon 5 (273C>G, 287A>T), one polymorphism in exon 11 (2630T>G), three missense mutations in exon 11 (2532T>G, 3191C>G, 3876C>A), one missense mutation in exon 12 (4285G>A), two missense mutations in exon 17 (5115T>C, 5116A>G), and one missense mutation in exon 18 (5206T>A). The same analysis was carried out on matched blood specimens for each of the 20 nucleotide alterations revealed to be present in the germline. No nucleotide alterations were detected in the controls. These results suggest that somatic mutations of BRCA1 are infrequent in sporadic breast cancer, and nucleotide alterations were more easily observed in the breast cancer tissue DNA. It is likely that there are other important susceptibility genes that remain to be identified in patients with sporadic breast cancer. 
 [BRCA1 inhibits progesterone-induced proliferation and   migration   of breast cancer cells]. To study the effect of BRCA1 in regulating the proliferation and   migration   of breast cancer cells stimulated by progesterone.Breast cancer MCF-7 and T-47D cell were transfected with a vector containing the coding sequence of BRCA1 (pFlag-CMV2-BRCA1 wt) to induce BRCA1 overexpression or with the empty vector (control). The cells were then stimulated with progesterone, and the cell proliferation and   migration   were observed using MTT assay and wound healing assay, respectively. The proliferation and   migration   of MCF-7 cells were also observed following transfection with a small interfering RNA (siRNA) for BRCA1 knockdown or with a scrambled siRNA prior to progesterone stimulation.Transfection with the empty vector and with pFlag-CMV2-BRCA1 wt prior to progesterone stimulation caused significantly different proliferation rates in MCF-7 cells [(114.4∓6.0)% vs (82.1∓3.2)%, P<0.05] and in T-47D cells [(111.3∓4.3)% vs (84.2∓3.5)%, P<0.05], resulting also in significantly differe  nt cell m  igration rates (55.9% vs 15.8% in MCF-7 cells and 44.83% vs 10.43% in T-47D cells). Compared to the scrambled siRNA, BRCA1 siRNA transfection prior to progesterone stimulation significantly increased the proliferation rates [(114.4∓3.05)% vs (125.3∓4.0)%,   P<0.05] a  nd migration rate (39.2% vs 69.08%) of MCF-7 cells. The progesterone antagonist RU468 could antagonize the effects of BRCA1 knockdown in enhancing progesterone-stimulated MCF-7 cell prolif  eration a  nd migration.A decreased BRCA1 expression can enhance progesterone-stimulated tumor cell prolif  eration a  nd migration in sporadic breast cancer. 
 TNRC9 downregulates BRCA1 expression and promotes breast cancer aggressiveness. Although the linkage between germline mutations of BRCA1 and hereditary breast/ovarian cancers is well established, recent evidence suggests that altered expression of wild-type BRCA1 might contribute to the sporadic forms of breast cancer. The breast cancer gene trinucleotide-repeat-containing 9 (TNRC9; TOX3) has been associated with disease susceptibility but its function is undetermined. Here, we report that TNRC9 is often amplified and overexpressed in breast cancer, particularly in advanced breast cancer. Gene amplification was associated with reduced disease-free and metastasis-free survival rates. Ectopic expression of TNRC9 increased breast cancer cell proliferation,   migration  , and survival after exposure to apoptotic stimuli. These phenotypes were associated with tumor progression in a mouse model of breast cancer. Gene expression profiling, protein analysis, and in silico assays of large datasets of breast and ovarian cancer samples suggested that TNRC9 and BRCA1 expression were inversely correlated. Notably, we found that TNRC9 bound to both the BRCA1 promoter and the cAMP-responsive element-binding protein (CREB) complex, a regulator of BRCA1 transcription. In support of this connection, expression of TNRC9 downregulated expression of BRCA1 by altering the methylation status of its promoter. Our studies unveil a function for TNRC9 in breast cancer that highlights a new paradigm in BRCA1 regulation. 
 BRCA1 germline mutations in Indian familial breast cancer. Germline mutation analysis of BRCA1 gene has demonstrated significant allelic heterogeneity. These differences represent historical influences of   migration  , population structure and geographic or cultural isolation. To date, there have been no reports of Indian families with mutations in BRCA1. We have screened for mutations in selected coding exons of BRCA1 and their flanking intron regions in three breast or breast and ovarian cancer families with family history of three or more cases of breast cancer under age 45 and/or ovarian cancer at any age. We have also analyzed 10 female patients with sporadic breast cancer regardless of age and family history, as well as 50 unrelated normal individuals as controls. Thus a total of 90 samples were analyzed for BRCA1 mutations using polymerase chain reaction-mediated site directed mutagenesis (PSM) and single stranded conformation polymorphism (SSCP) analysis for various selected exons followed by sequencing of variant bands. Eight point mutations were identified. Two deleterious pathogenic, protein truncating non-sense mutations were detected in exon 11 (E1250X) and exon 20 (E1754X) and six novel and unique amino acid substitutions (F1734S, D1739Y, V1741G, Q1747H, P1749A, R1753K). One complex missense mutation of exon 20 [V1741G; P1749A] was seen in two out of three families and another complex combination of missense and non-sense mutations of the same exon [V1741G; E1754X] was observed in only one family. These complex mutations exist only in breast cancer families but not in control populations of women. Three splice site variants (IVS20+3A>C, IVS20+4A>T, IVS20+5A>T) and two intronic variants (IVS20+21_22insG, IVS20+21T>G) were also detected. In the group of 10 sporadic female patients no mutations were found. 
 In vitro platination of human breast cancer suppressor gene1 (BRCA1) by the anticancer drug carboplatin. Carboplatin is an anticancer drug for the treatment of cancers affecting various organs including ovary and testes. It essentially exerts its cytotoxicity against cancerous cells via covalent attachment of platinum atom to DNA, generating various platinum-DNA adducts. Platinum-DNA adducts inhibit biological processes essential for cellular viability. However, carboplatin interacts nonspecifically with DNA, resulting in damaging of normal cell DNA. Potential in vitro interaction of carboplatin with genes encoding tumor suppressor proteins such as human breast cancer suppressor gene 1(BRCA1) was herein investigated. The 696--bp fragment of the 3'-region of BRCA1 gene (nucleotide 4897--5592) was amplified by RT-PCR using mRNA templates isolated from human white blood cells. Retardation of the electrophoretic   migration   on agarose gel of drug-treated DNA, in the dose-response manner, was observed. Analysis by restriction digestion with PvuII and Eco O 109I suggested that the platination favorably occurred at the dGpG sequence of Eco O 109I-cleaved site. The semi-quantitative PCR-based assay was used to determine the lesion frequencies produced by carboplatin in the 696-bp fragment of the 3'-region of BRCA1 gene and in the 3,426-bp fragment of the BRCA1 exon 11 of human breast adenocarcinoma MCF-7 cells. A significant decrease in DNA amplification was observed at 400 microM of carboplatin with approximately 1--2 platinum atoms per BRCA1 fragment. Carboplatin caused slightly less damage at equimolar concentrations in cells than in cell-free BRCA1 fragment. 
 BRCA1 deficiency in ovarian cancer is associated with alteration in expression of several key regulators of cell motility - A proteomics study. Functional loss of expression of breast cancer susceptibility gene 1(BRCA1) has been implicated in genomic instability and cancer progression. There is emerging evidence that BRCA1 gene product (BRCA1) also plays a role in cancer cell   migration  . We performed a quantitative proteomics study of EOC patient tumor tissues and identified changes in expression of several key regulators of actin cytoskeleton/cell adhesion and cell   migration   (CAPN1, 14-3-3, CAPG, PFN1, SPTBN1, CFN1) associated with loss of BRCA1 function. Gene expression analyses demonstrate that several of these proteomic hits are differentially expressed between early and advanced stage EOC thus suggesting clinical relevance of these proteins to disease progression. By immunohistochemistry of ovarian tumors with BRCA1(+/+) and BRCA1(null) status, we further verified our proteomic-based finding of elevated PFN1 expression associated with BRCA1 deficiency. Finally, we established a causal link between PFN1 and BRCA1-induced changes in cell   migration   thus uncovering a novel mechanistic basis for BRCA1-dependent regulation of ovarian cancer cell   migration  . Overall, findings of this study open up multiple avenues by which BRCA1 can potentially regulate   migration   and metastatic phenotype of EOC cells. 
  GeneRIFs  
 Decreased BRCA1 expression can enhance progesterone-stimulated tumor cell proliferation and   migration   in sporadic breast cancer. 
  ECM  PubMed  
 Extracellular matrix enhances heregulin-dependent BRCA1 phosphorylation and suppresses BRCA1 expression through its C terminus. Germ line mutations in the breast cancer susceptibility gene BRCA1 account for the increased risk of early onset of familial breast cancer, whereas overexpression of the ErbB family of receptor tyrosine kinases has been linked to the development of nonfamilial or sporadic breast cancer. To analyze whether there is a link between these two regulatory molecules, we studied the effects of ErbB-2 activation by heregulin (HRG) on BRCA1 function. It was previously demonstrated that HRG induced the phosphorylation of BRCA1, which was mediated by the phosphatidylinositol 3-kinase (PI3K)/Akt pathway. Since altered interaction between cells and the surrounding extracellular matrix (  ECM  ) is a common feature in a variety of tumors and since   ECM   modulates intracellular signaling, we hypothesized that   ECM   may affect the expression and HRG-dependent phosphorylation of BRCA1. Following stimulation by HRG, a strong increase in [(3)H]thymidine incorporation was observed in human T47D breast cancer cells seeded on plastic (PL). When T47D cells were seeded on laminin (LAM) or Matrigel, HRG induced a significantly higher proliferation than it did in cells seeded on PL. T47D cells seeded on poly-L-lysine had an abrogated mitogenic response, indicating the involvement of integrins in this process. HRG treatment induced a transient phosphorylation of BRCA1 that was enhanced in T47D cells grown on LAM. LAM-enhanced BRCA1 phosphorylation was mediated through alpha(6) integrin upon HRG stimulation. Accordingly, T47D cells grown on LAM had the greatest increase in ErbB-2 activation, PI3K activity, and phosphorylation of Akt. A similar pattern of BRCA1 mRNA expression was observed when T47D cells were seeded on PL, LAM, or COL4. There was a significant decrease in the steady state of the BRCA1 mRNA level on both the LAM and COL4 matrices compared to that for cells seeded on PL. In addition, HRG stimulation caused a significant decrease in BRCA1 mRNA expression that was dependent on protein synthesis. Pretreatment with both the calpain inhibitor ALLN (N-acetyl-Leu-Leu-norleucinal) and the proteosome inhibitor lactacystin inhibited the HRG-induced down-regulation of BRCA1 mRNA expression. Likewise, there was a strong decrease in the protein level of BRCA1 in T47D cells 4 h after treatment with HRG compared to its level in control nontreated T47D cells. Pretreatment with the proteosome inhibitors ALLN, lactacystin, and PSI [N-benzyloxycarbonyl-Ile-Glu-(O-t-butyl)-Ala-leucinal] inhibited also the HRG-induced down-regulation of BRCA1 protein in breast cancer cells. Interestingly, BRCA1 mRNA expression in HCC-1937 breast cancer cells, which express C-terminally truncated BRCA1, was not affected by either LAM or CL4. No phosphorylation of BRCA1 from HCC-1937 cells was observed in response to HRG. While Cdk4 phosphorylated wild-type BRCA1 in response to HRG in T47D cells, Cdk4 failed to phosphorylate the truncated form of BRCA1 in HCC-1937 cells. Furthermore, overexpression of wild-type BRCA1 in HCC-1937 cells resulted in the phosphorylation of BRCA1 and decreased BRCA1 expression upon HRG stimulation while overexpression of truncated BRCA1 in T47D cells resulted in a lack of BRCA1 phosphorylation and restoration of BRCA1 expression. These findings suggest that   ECM   enhances HRG-dependent BRCA1 phosphorylation and that   ECM   and HRG down-regulate BRCA1 expression in breast cancer cells. Furthermore,   ECM   suppresses BRCA1 expression through the C terminus of BRCA1. 
  [Ee]xtracellular [Mm]atrix  PubMed  
 CAF-like state in primary skin fibroblasts with constitutional BRCA1 epimutation sheds new light on tumor suppressor deficiency-related changes in healthy tissue. Constitutive epimutations of tumor suppressor genes are increasingly considered as cancer predisposing factors equally to sequence mutations. In light of the emerging role of the microenvironment for cancer predisposition, initiation, and progression, we aimed to characterize the consequences of a BRCA1 epimutation in cells of mesenchymal origin. We performed a comprehensive molecular and cellular comparison of primary dermal fibroblasts taken from a monozygous twin pair discordant for recurrent cancers and BRCA1 epimutation, whose exceptional clinical case we previously reported in this journal. Comparative transcriptome analysis identified differential expression of   extracellular matrix  -related genes and pro-tumorigenic growth factors, such as collagens and CXC chemokines. Moreover, genes known to be key markers of so called cancer-associated fibroblasts (CAFs), such as ACTA2, FAP, PDPN, and TNC, were upregulated in fibroblasts of the affected twin (BRCA1(mosMe)) in comparison to those of the healthy twin (BRCA1(wt)). Further analyses detected CAF-typical cellular features, including an elevated growth rate, enhanced migration, altered actin architecture and increased production of ketone bodies in BRCA1(mosMe) fibroblasts compared to BRCA1(wt) fibroblasts. In addition, conditioned medium of BRCA1(mosMe) fibroblasts was more potent than conditioned medium of BRCA1(wt) fibroblasts to promote cell proliferation in an epithelial and a cancer cell line. Our data demonstrate, that a CAF-like state is not an exclusive feature of tumor-associated tissue but also exists in healthy tissue with tumor suppressor deficiency. The naturally occurring phenomenon of twin fibroblasts differing in their BRCA1 methylation status revealed to be a unique powerful tool for exploring tumor suppressor deficiency-related changes in healthy tissue, reinforcing their significance for cancer predisposition. 
 Depletion of BRCA1 impairs differentiation but enhances proliferation of mammary epithelial cells. Cumulative evidence indicates that breast cancer-associated gene 1 (BRCA1) participates in DNA damage repair and cell-cycle checkpoint control, serving as a tumor susceptibility gene to maintain the global genomic stability. However, whether BRCA1 has a direct role in cell proliferation and differentiation, two key biological functions in tumorigenesis, remains unclear. Here we demonstrate BRCA1 mediates differentiation of mammary epithelial cell (MEC) for acinus formation by using the in vitro 3D culture system. Reduction of BRCA1 in MEC by RNA interference impairs the acinus formation but enhances proliferation. Such aberrations can be rescued by expression of wild-type BRCA1 as well as a mutant at the RAD50-binding domain but not at the C-terminal BRCT domain, suggesting that the C-terminal BRCT domain has a critical role in these processes. Consistently, depletion of BRCA1 up-regulates the gene expression for proliferation but down-regulates that for differentiation. Moreover, application of the medium conditioned by differentiating normal MEC can reverse the phenotypes of differentiation-defective breast cancer cells bearing reduced BRCA1 functions. Our observation implies BRCA1 is involved in secretion of certain paracrine/autocrine factors that induce MEC differentiation in response to   extracellular matrix   signals, providing, in part, an explanation for the etiological basis of either sporadic or familial breast cancer due to the loss or reduction of BRCA1. 
   Extracellular matrix   enhances heregulin-dependent BRCA1 phosphorylation and suppresses BRCA1 expression through its C terminus. Germ line mutations in the breast cancer susceptibility gene BRCA1 account for the increased risk of early onset of familial breast cancer, whereas overexpression of the ErbB family of receptor tyrosine kinases has been linked to the development of nonfamilial or sporadic breast cancer. To analyze whether there is a link between these two regulatory molecules, we studied the effects of ErbB-2 activation by heregulin (HRG) on BRCA1 function. It was previously demonstrated that HRG induced the phosphorylation of BRCA1, which was mediated by the phosphatidylinositol 3-kinase (PI3K)/Akt pathway. Since altered interaction between cells and the surrounding   extracellular matrix   (ECM) is a common feature in a variety of tumors and since ECM modulates intracellular signaling, we hypothesized that ECM may affect the expression and HRG-dependent phosphorylation of BRCA1. Following stimulation by HRG, a strong increase in [(3)H]thymidine incorporation was observed in human T47D breast cancer cells seeded on plastic (PL). When T47D cells were seeded on laminin (LAM) or Matrigel, HRG induced a significantly higher proliferation than it did in cells seeded on PL. T47D cells seeded on poly-L-lysine had an abrogated mitogenic response, indicating the involvement of integrins in this process. HRG treatment induced a transient phosphorylation of BRCA1 that was enhanced in T47D cells grown on LAM. LAM-enhanced BRCA1 phosphorylation was mediated through alpha(6) integrin upon HRG stimulation. Accordingly, T47D cells grown on LAM had the greatest increase in ErbB-2 activation, PI3K activity, and phosphorylation of Akt. A similar pattern of BRCA1 mRNA expression was observed when T47D cells were seeded on PL, LAM, or COL4. There was a significant decrease in the steady state of the BRCA1 mRNA level on both the LAM and COL4 matrices compared to that for cells seeded on PL. In addition, HRG stimulation caused a significant decrease in BRCA1 mRNA expression that was dependent on protein synthesis. Pretreatment with both the calpain inhibitor ALLN (N-acetyl-Leu-Leu-norleucinal) and the proteosome inhibitor lactacystin inhibited the HRG-induced down-regulation of BRCA1 mRNA expression. Likewise, there was a strong decrease in the protein level of BRCA1 in T47D cells 4 h after treatment with HRG compared to its level in control nontreated T47D cells. Pretreatment with the proteosome inhibitors ALLN, lactacystin, and PSI [N-benzyloxycarbonyl-Ile-Glu-(O-t-butyl)-Ala-leucinal] inhibited also the HRG-induced down-regulation of BRCA1 protein in breast cancer cells. Interestingly, BRCA1 mRNA expression in HCC-1937 breast cancer cells, which express C-terminally truncated BRCA1, was not affected by either LAM or CL4. No phosphorylation of BRCA1 from HCC-1937 cells was observed in response to HRG. While Cdk4 phosphorylated wild-type BRCA1 in response to HRG in T47D cells, Cdk4 failed to phosphorylate the truncated form of BRCA1 in HCC-1937 cells. Furthermore, overexpression of wild-type BRCA1 in HCC-1937 cells resulted in the phosphorylation of BRCA1 and decreased BRCA1 expression upon HRG stimulation while overexpression of truncated BRCA1 in T47D cells resulted in a lack of BRCA1 phosphorylation and restoration of BRCA1 expression. These findings suggest that ECM enhances HRG-dependent BRCA1 phosphorylation and that ECM and HRG down-regulate BRCA1 expression in breast cancer cells. Furthermore, ECM suppresses BRCA1 expression through the C terminus of BRCA1. 
 BRCA1 transcriptionally regulates genes involved in breast tumorigenesis. Loss of function of BRCA1 caused by inherited mutation and tissue-specific somatic mutation leads to breast and ovarian cancer. Nearly all BRCA1 germ-line mutations involve truncation or loss of the C-terminal BRCT transcriptional activation domain, suggesting that transcriptional regulation is a critical function of the wild-type gene. The purpose of this project was to determine whether there is a link between the role of BRCA1 in transcriptional regulation and its role in tumor suppression. We developed a cell line (in which BRCA1 can be induced) and used microarray analysis to compare transcription profiles of epithelial cells with low endogenous levels of BRCA1 vs. transcription profiles of cells with 2-4-fold higher induced levels of expression of BRCA1. At these levels of expression, BRCA1 did not induce apoptosis. Undirected cluster analysis of six paired experiments revealed 373 genes, the expression of which was altered significantly and consistently by BRCA1 induction. Expression of 62 genes was altered more than 2-fold. BRCA1-regulated genes associated with breast tumorigenesis included the estrogen-responsive genes MYC and cyclin D1, which are overexpressed in many breast tumors; STAT1 and JAK1, key components of the cytokine signal transduction pathway; the   extracellular matrix   protein laminin 3A; ID4, an inhibitor of DNA-binding transcriptional activators, which in turn negatively regulates BRCA1 expression; and the prohormone stanniocalcin, expression of which is lost in breast tumor cells. Coordinated expression of BRCA1 with ID4 and with stanniocalcin was confirmed in primary breast and ovarian tumors. 
  GeneRIFs  
 BRCA1 is involved in secretion of certain paracrine/autocrine factors that induce mammary epithelial cell differentiation in response to   extracellular matrix   signals 
 BRCA1 is downregulated by heregulin in the   extracellular matrix   in breast tumor cells 
  [Ii]nvasive  PubMed  
 Genetic susceptibility to distinct bladder cancer subphenotypes. Clinical, pathologic, and molecular evidence indicate that bladder cancer is heterogeneous with pathologic/molecular features that define distinct subphenotypes with different prognoses. It is conceivable that specific patterns of genetic susceptibility are associated with particular subphenotypes.To examine evidence for the contribution of germline genetic variation to bladder cancer heterogeneity.The Spanish Bladder Cancer/EPICURO Study is a case-control study based in 18 hospitals located in five areas in Spain. Cases were patients with a newly diagnosed, histologically confirmed, urothelial cell carcinoma of the bladder from 1998 to 2001. Case diagnoses were reviewed and uniformly classified by pathologists following the World Health Organisation/International Society of Urological Pathology 1999 criteria. Controls were hospital-matched patients (n=1149).A total of 1526 candidate variants in 423 candidate genes were analysed. Three distinct subphenotypes were defined according to stage and grade: low-grade nonmuscle   invasive   (n=586), high-grade nonmuscle   invasive   (n=219), and muscle   invasive   (n=246). The association between each variant and subphenotype was assessed by polytomous risk models adjusting for potential confounders. Heterogeneity in genetic susceptibility among subphenotypes was also tested.Two established bladder cancer susceptibility genotypes, NAT2 slow-acetylation and GSTM1-null, exhibited similar associations among the subphenotypes, as did VEGF-rs25648, which was previously identified in our study. Other variants conferred risks for specific tumour subphenotypes such as PMS2-rs6463524 and CD4-rs3213427 (respective heterogeneity p values of 0.006 and 0.004), which were associated with muscle-  invasive   tumours (per-allele odds ratios [95% confidence interval] of 0.56 [0.41-0.77] and 0.71 [0.57-0.88], respectively) but not with non-muscle-  invasive   tumours. Heterogeneity p values were not robust in multiple testing according to their false-discovery rate.These exploratory analyses suggest that genetic susceptibility loci might be related to the molecular/pathologic diversity of bladder cancer. Validation through large-scale replication studies and the study of additional genes and single nucleotide polymorphisms are required. 
 ERBB2, TBX2, RPS6KB1, and MYC alterations in breast tissues of BRCA1 and BRCA2 mutation carriers. Breast cancer risk is greatly increased in women who carry mutations in the BRCA1 or BRCA2 genes. Because breast cancer initiation is different between BRCA1/2 mutation carriers and women who do not carry mutations, it is possible that the mechanism of breast cancer progression is also different. Histopathologic and genetic studies have supported this hypothesis. To test this hypothesis further, we utilized a large cohort of women who underwent therapeutic mastectomy (TM) and contralateral prophylactic mastectomy (PM). From this cohort, we developed case groups of women with a family history of breast cancer with BRCA1/2 deleterious mutations, with unclassified variant alterations, and with no detected mutation and matched these cases with sporadic controls from the same TM and PM cohort. Fluorescence in situ hybridization was performed on paraffin sections by use of dual-color probes for ERBB2/CEP17, MYC/CEP8, TBX2/CEP17, and RPS6KB1/CEP17. All malignant and benign lesions, including putative precursor lesions, were studied. The   invasive   cancers from deleterious mutation carriers had a higher prevalence of duplication of MYC (P = 0.006) and TBX2 (P = 0.0008) compared to controls and a lower prevalence of ERBB2 amplification (P = 0.011). Coduplication of MYC and TBX2 was common in the in situ and   invasive   lesions from the deleterious mutation carriers. The odds ratio of having a BRCA1/2 mutation is 31.4 (95% CI = 1.7-569) when MYC and TBX2 are coduplicated but ERBB2 is normal. Unclassified variant carriers/no mutation detected and sporadic controls had a similar prevalence of alterations, suggesting that hereditary patients with no deleterious mutations follow a progression pathway similar to that of sporadic cases. With the exception of one atypical ductal hyperplasia lesion, no putative precursor lesion showed any detectable alteration of the probes tested. There was no significant intratumoral heterogeneity of genetic alterations. Our data confirm that a specific pattern of genomic instability characterizes BRCA1/2-related cancers and that this pattern has implications for the biology of these cancers. Moreover, our current and previous results emphasize the interaction between phenotype and genotype in BRCA1/2-related breast cancers and that a combination of morphologic features and alterations of ERBB2, MYC, and TBX2 may better define mechanisms of tumor progression, as well as determine which patients are more likely to carry BRCA1/2 mutations. 
 The influence of genetic variation in 30 selected genes on the clinical characteristics of early onset breast cancer. Common variants that alter breast cancer risk are being discovered. Here, we determine how these variants influence breast cancer prognosis, risk and tumour characteristics.We selected 1,001 women with early onset nonfamilial   invasive   breast cancer from the Prospective study of Outcomes in Sporadic versus Hereditary breast cancer (POSH) cohort and genotyped 206 single nucleotide polymorphisms (SNPs) across 30 candidate genes. After quality control, 899 cases and 133 SNPs remained. Survival analyses were used to identify SNPs associated with prognosis and determine their interdependency with recognized prognostic factors. To identify SNPs that alter breast cancer risk, association tests were used to compare cases with controls from the Wellcome Trust Case Control Consortium. To search for SNPs affecting tumour biology, cases were stratified into subgroups according to oestrogen receptor (ER) status and grade and tested for association.We confirmed previous associations between increased breast cancer risk and SNPs in CASP8, TOX3 (previously known as TNRC9) and ESR1. Analysis of prognosis identified eight SNPs in six genes (MAP3K1, DAPK1, LSP1, MMP7, TOX3 and ESR1) and one region without genes on 8q24 that are associated with survival. For MMP7, TOX3 and MAP3K1 the effects on survival are independent of the main recognized clinical prognostic factors. The SNP in 8q24 is more weakly associated with independent effects on survival. Once grade and pathological nodal status (pN stage) were taken into account, SNPs in ESR1 and LSP1 showed no independent survival difference, whereas the effects of the DAPK1 SNP were removed when correcting for ER status. Interestingly, effects on survival for SNPs in ESR1 were most significant when only ER-positive tumours were examined. Stratifying POSH cases by tumour characteristics identified SNPs in FGFR2 and TOX3 associated with ER-positive disease and SNPs in ATM associated with ER-negative disease.We have demonstrated that several SNPs are associated with survival. In some cases this appears to be due to an effect on tumour characteristics known to have a bearing on prognosis; in other cases the effect appears to be independent of these prognostic factors. These findings require validation by further studies in similar patient groups. 
 ERBB2, TBX2, RPS6KB1, and MYC alterations in breast tissues of BRCA1 and BRCA2 mutation carriers. Breast cancer risk is greatly increased in women who carry mutations in the BRCA1 or BRCA2 genes. Because breast cancer initiation is different between BRCA1/2 mutation carriers and women who do not carry mutations, it is possible that the mechanism of breast cancer progression is also different. Histopathologic and genetic studies have supported this hypothesis. To test this hypothesis further, we utilized a large cohort of women who underwent therapeutic mastectomy (TM) and contralateral prophylactic mastectomy (PM). From this cohort, we developed case groups of women with a family history of breast cancer with BRCA1/2 deleterious mutations, with unclassified variant alterations, and with no detected mutation and matched these cases with sporadic controls from the same TM and PM cohort. Fluorescence in situ hybridization was performed on paraffin sections by use of dual-color probes for ERBB2/CEP17, MYC/CEP8, TBX2/CEP17, and RPS6KB1/CEP17. All malignant and benign lesions, including putative precursor lesions, were studied. The   invasive   cancers from deleterious mutation carriers had a higher prevalence of duplication of MYC (P = 0.006) and TBX2 (P = 0.0008) compared to controls and a lower prevalence of ERBB2 amplification (P = 0.011). Coduplication of MYC and TBX2 was common in the in situ and   invasive   lesions from the deleterious mutation carriers. The odds ratio of having a BRCA1/2 mutation is 31.4 (95% CI = 1.7-569) when MYC and TBX2 are coduplicated but ERBB2 is normal. Unclassified variant carriers/no mutation detected and sporadic controls had a similar prevalence of alterations, suggesting that hereditary patients with no deleterious mutations follow a progression pathway similar to that of sporadic cases. With the exception of one atypical ductal hyperplasia lesion, no putative precursor lesion showed any detectable alteration of the probes tested. There was no significant intratumoral heterogeneity of genetic alterations. Our data confirm that a specific pattern of genomic instability characterizes BRCA1/2-related cancers and that this pattern has implications for the biology of these cancers. Moreover, our current and previous results emphasize the interaction between phenotype and genotype in BRCA1/2-related breast cancers and that a combination of morphologic features and alterations of ERBB2, MYC, and TBX2 may better define mechanisms of tumor progression, as well as determine which patients are more likely to carry BRCA1/2 mutations. 
 Genetic susceptibility to distinct bladder cancer subphenotypes. Clinical, pathologic, and molecular evidence indicate that bladder cancer is heterogeneous with pathologic/molecular features that define distinct subphenotypes with different prognoses. It is conceivable that specific patterns of genetic susceptibility are associated with particular subphenotypes.To examine evidence for the contribution of germline genetic variation to bladder cancer heterogeneity.The Spanish Bladder Cancer/EPICURO Study is a case-control study based in 18 hospitals located in five areas in Spain. Cases were patients with a newly diagnosed, histologically confirmed, urothelial cell carcinoma of the bladder from 1998 to 2001. Case diagnoses were reviewed and uniformly classified by pathologists following the World Health Organisation/International Society of Urological Pathology 1999 criteria. Controls were hospital-matched patients (n=1149).A total of 1526 candidate variants in 423 candidate genes were analysed. Three distinct subphenotypes were defined according to stage and grade: low-grade nonmuscle   invasive   (n=586), high-grade nonmuscle   invasive   (n=219), and muscle   invasive   (n=246). The association between each variant and subphenotype was assessed by polytomous risk models adjusting for potential confounders. Heterogeneity in genetic susceptibility among subphenotypes was also tested.Two established bladder cancer susceptibility genotypes, NAT2 slow-acetylation and GSTM1-null, exhibited similar associations among the subphenotypes, as did VEGF-rs25648, which was previously identified in our study. Other variants conferred risks for specific tumour subphenotypes such as PMS2-rs6463524 and CD4-rs3213427 (respective heterogeneity p values of 0.006 and 0.004), which were associated with muscle-  invasive   tumours (per-allele odds ratios [95% confidence interval] of 0.56 [0.41-0.77] and 0.71 [0.57-0.88], respectively) but not with non-muscle-  invasive   tumours. Heterogeneity p values were not robust in multiple testing according to their false-discovery rate.These exploratory analyses suggest that genetic susceptibility loci might be related to the molecular/pathologic diversity of bladder cancer. Validation through large-scale replication studies and the study of additional genes and single nucleotide polymorphisms are required. 
 The influence of genetic variation in 30 selected genes on the clinical characteristics of early onset breast cancer. Common variants that alter breast cancer risk are being discovered. Here, we determine how these variants influence breast cancer prognosis, risk and tumour characteristics.We selected 1,001 women with early onset nonfamilial   invasive   breast cancer from the Prospective study of Outcomes in Sporadic versus Hereditary breast cancer (POSH) cohort and genotyped 206 single nucleotide polymorphisms (SNPs) across 30 candidate genes. After quality control, 899 cases and 133 SNPs remained. Survival analyses were used to identify SNPs associated with prognosis and determine their interdependency with recognized prognostic factors. To identify SNPs that alter breast cancer risk, association tests were used to compare cases with controls from the Wellcome Trust Case Control Consortium. To search for SNPs affecting tumour biology, cases were stratified into subgroups according to oestrogen receptor (ER) status and grade and tested for association.We confirmed previous associations between increased breast cancer risk and SNPs in CASP8, TOX3 (previously known as TNRC9) and ESR1. Analysis of prognosis identified eight SNPs in six genes (MAP3K1, DAPK1, LSP1, MMP7, TOX3 and ESR1) and one region without genes on 8q24 that are associated with survival. For MMP7, TOX3 and MAP3K1 the effects on survival are independent of the main recognized clinical prognostic factors. The SNP in 8q24 is more weakly associated with independent effects on survival. Once grade and pathological nodal status (pN stage) were taken into account, SNPs in ESR1 and LSP1 showed no independent survival difference, whereas the effects of the DAPK1 SNP were removed when correcting for ER status. Interestingly, effects on survival for SNPs in ESR1 were most significant when only ER-positive tumours were examined. Stratifying POSH cases by tumour characteristics identified SNPs in FGFR2 and TOX3 associated with ER-positive disease and SNPs in ATM associated with ER-negative disease.We have demonstrated that several SNPs are associated with survival. In some cases this appears to be due to an effect on tumour characteristics known to have a bearing on prognosis; in other cases the effect appears to be independent of these prognostic factors. These findings require validation by further studies in similar patient groups. 
 Population BRCA1 and BRCA2 mutation frequencies and cancer penetrances: a kin-cohort study in Ontario, Canada. BRCA1 and BRCA2 mutations in general populations and in various types of cancers have not been well characterized. We investigated the presence of these mutations in unselected patients with newly diagnosed incident ovarian cancer in Ontario, Canada, with respect to cancers reported among their relatives.A population series of 1171 unselected patients with incident ovarian cancer diagnosed between January 1, 1995, and December 31, 1999, in Ontario, Canada, was screened for germline mutations throughout the BRCA1 and BRCA2 genes. Screening involved testing for common variants, then protein truncation testing of long exons, and then denaturing gradient gel electrophoresis or denaturing high-performance liquid chromatography for the remainder of BRCA1 and BRCA2, respectively. Cox regression analysis was used to examine cancer outcomes reported by the case probands for their 8680 first-degree relatives. Population allele frequencies and relative risks (RRs) were derived from the regression results by an extension of Saunders-Begg methods. Age-specific Ontario cancer incidence rates were used to estimate cumulative incidence of cancer to age 80 years by mutation status.Among 977 patients with   invasive   ovarian cancer, 75 had BRCA1 mutations and 54 had BRCA2 mutations, for a total mutation frequency of 13.2% (95% confidence interval [CI] = 11.2% to 15.5%). Higher risks for various cancer types in the general Ontario population were associated with BRCA1 mutation carriage than with noncarriage, including ovarian (RR = 21, 95% CI = 12 to 36), female breast (RR = 11, 95% CI = 7.5 to 15), and testis (RR = 17, 95% CI = 1.3 to 230) cancers. Higher risks were also associated with BRCA2 mutation carriage than with noncarriage, particularly for ovarian (RR = 7.0, 95% CI = 3.1 to 16), female and male breast (RR = 4.6, 95% CI = 2.7 to 7.8, and RR = 102, 95% CI = 9.9 to 1050, respectively), and pancreatic (RR = 6.6, 95% CI = 1.9 to 23) cancers. Cancer risks differed according to the mutation's position in the gene. Estimated cumulative incidence to age 80 years among women carrying BRCA1 mutations was 24% for ovarian cancer and 90% for breast cancer and among women carrying BRCA2 mutations was 8.4% for ovarian cancer and 41% for breast cancer. For the general Ontario population, estimated carrier frequencies of BRCA1 and BRCA2 mutations, respectively, were 0.32% (95% CI = 0.23% to 0.45%) and 0.69% (95% CI = 0.43% to 1.10%).BRCA1 and BRCA2 mutations may be more frequent in general populations than previously thought and may be associated with various types of cancers. 
 Association of BRCA1, BRCA2, RAD51, and HER2 gene polymorphisms with the breast cancer risk in the Bangladeshi population. Breast cancer is considered as the most frequent female malignancy. Altered gene expressions due to genetic polymorphisms in the BRCA1, BRCA2, RAD51, and HER2 contribute toward the development of breast cancer, and yet, no such type of study has been conducted in the Bangladeshi population. This study was designed to evaluate the role of BRCA1rs80357713, BRCA1rs80357906, BRCA2rs11571653, RAD51rs1801320, and HER2rs1136201 polymorphisms as risk factors in the development of breast cancer in the Bangladeshi population.A total 310 patients with   invasive   breast cancers were recruited as cases from different public and private hospitals of Bangladesh, and 250 Bangladeshi healthy women matching age with the patients were recruited as controls. Polymerase chain reaction-restriction fragment length polymorphism method was used to analyze the genetic polymorphisms.Patients carrying BRCA1/2 mutations, GC and GC plus CC genotypes of RAD51rs1801320, and AG plus GG genotype of HER2rs1136201 polymorphisms were found to be associated with breast cancer. In subgroup analysis, AG plus GG genotype of HER2rs1136201 was found to be associated with the breast cancer risk in the patients younger than 45 years of age compared with the older patients having more than 45 years of age, and RAD51rs1801320 was related to the tumor size and tumor aggressiveness (higher graded tumor).Our results indicate that BRCA1/BRCA2, RAD51rs1801320 and HER2rs1136201 polymorphisms were associated with breast cancer in the studied population. 
 Estrogen receptor positive breast cancers in BRCA1 mutation carriers: clinical risk factors and pathologic features. Most breast cancers that occur in women with germline BRCA1 mutations are estrogen receptor-negative (ER-) and also typically lack expression of progesterone receptor (PR) and HER2 overexpression. We undertook a study to assess the clinical factors that predict for an estrogen receptor positive (ER+) breast cancer in BRCA1 mutation carriers and to characterize the pathologic features of these tumors.Clinical characteristics of BRCA1 carriers with 58 ER+ and 114 ER- first   invasive   breast cancers were compared. Pathologic features of BRCA1 ER+ cancers were compared to those of BRCA1 ER- cancers and to age-matched ER+ sporadic cancers.BRCA1 carriers aged > or = 50 at diagnosis of first   invasive   breast cancer were more likely to have an ER+ cancer compared to those aged < 50 (57% vs 29%, P = 0.005). ER+ BRCA1 cancers were less likely than ER- BRCA1 cancers to have "BRCA-associated" features such as high mitotic activity, geographic necrosis/fibrotic focus, and pushing margins (RR 0.06, 0.22, 0.24; P < 0.001, 0.02, 0.03 respectively). When compared to sporadic ER+ cancers, ER+ BRCA1 cancers were more often of   invasive   ductal type (RR 2.4, P = 0.03), with a high mitotic rate (RR 5.0, P = 0.006) and absent or mild lymphocytic infiltrate (RR 10.2, P = 0.04).BRCA1 carriers who are older at first breast cancer diagnosis are more likely to have ER+ tumors than younger BRCA1 carriers. These ER+ cancers appear pathologically "intermediate" between ER- BRCA1 cancers and ER+ sporadic breast cancers raising the possibility that either some ER+ BRCA1 cancers are incidental or that there is a unique mechanism by which these cancers develop. 
 Association between BRCA1 and BRCA2 mutations and survival in women with   invasive   epithelial ovarian cancer. Approximately 10% of women with   invasive   epithelial ovarian cancer (EOC) carry deleterious germline mutations in BRCA1 or BRCA2. A recent article suggested that BRCA2-related EOC was associated with an improved prognosis, but the effect of BRCA1 remains unclear.To characterize the survival of BRCA carriers with EOC compared with noncarriers and to determine whether BRCA1 and BRCA2 carriers show similar survival patterns.A pooled analysis of 26 observational studies on the survival of women with ovarian cancer, which included data from 1213 EOC cases with pathogenic germline mutations in BRCA1 (n = 909) or BRCA2 (n = 304) and from 2666 noncarriers recruited and followed up at variable times between 1987 and 2010 (the median year of diagnosis was 1998).Five-year overall mortality.The 5-year overall survival was 36% (95% CI, 34%-38%) for noncarriers, 44% (95% CI, 40%-48%) for BRCA1 carriers, and 52% (95% CI, 46%-58%) for BRCA2 carriers. After adjusting for study and year of diagnosis, BRCA1 and BRCA2 mutation carriers showed a more favorable survival than noncarriers (for BRCA1: hazard ratio [HR], 0.78; 95% CI, 0.68-0.89; P < .001; and for BRCA2: HR, 0.61; 95% CI, 0.50-0.76; P < .001). These survival differences remained after additional adjustment for stage, grade, histology, and age at diagnosis (for BRCA1: HR, 0.73; 95% CI, 0.64-0.84; P < .001; and for BRCA2: HR, 0.49; 95% CI, 0.39-0.61; P < .001). The BRCA1 HR estimate was significantly different from the HR estimated in the adjusted model (P for heterogeneity = .003).Among patients with   invasive   EOC, having a germline mutation in BRCA1 or BRCA2 was associated with improved 5-year overall survival. BRCA2 carriers had the best prognosis. 
 Clinical significance of epigenetic inactivation of hMLH1 and BRCA1 in Tunisian patients with   invasive   breast carcinoma. Aberrant hypermethylation of gene promoter regions is one of the mechanisms for inactivation of tumour suppressor genes in many human cancers including breast carcinoma. In the current study, we aimed to assess by MSP, the methylation pattern of two cancer-related genes involved in DNA repair: hMLH1 (mutL homolog 1, colon cancer, nonpolyposis type 2 (E. coli) and BRCA1 (breast cancer 1, early onset) in 78 primary breast cancers from Tunisian patients. The methylation frequencies were 24.36% for hMLH1 and 46% for BRCA1. BRCA1 methylation correlated with age at diagnosis (P = .015) and 5-years disease free survival (P = .016) while hMLH1 methylation was more frequent in larger tumors (P = .002) and in presence of distant metastasis (P = .004). Furthermore, methylation of hMLH1 significantly correlated with high level of P53 expression (P = .006) and with overall survival (P = .015) suggesting that silencing of hMLH1 through aberrant promoter methylation could be used as a poor prognosis indicator in breast cancer. 
 The contribution of deleterious germline mutations in BRCA1, BRCA2 and the mismatch repair genes to ovarian cancer in the population. The aim of this study was to estimate the contribution of deleterious mutations in BRCA1, BRCA2, MLH1, MSH2, MSH6 and PMS2 to   invasive   epithelial ovarian cancer (EOC) in the population. The coding sequence and splice site boundaries of all six genes were amplified in germline DNA from 2240   invasive   EOC cases and 1535 controls. Barcoded fragment libraries were sequenced using the Illumina GAII or HiSeq and sequence data for each subject de-multiplexed prior to interpretation. GATK and Annovar were used for variant detection and annotation. After quality control 2222 cases (99.2%) and 1528 controls (99.5%) were included in the final analysis. We identified 193 EOC cases (8.7%) carrying a deleterious mutation in at least one gene compared with 10 controls (0.65%). Mutations were most frequent in BRCA1 and BRCA2, with 84 EOC cases (3.8%) carrying a BRCA1 mutation and 94 EOC cases (4.2%) carrying a BRCA2 mutation. The combined BRCA1 and BRCA2 mutation prevalence was 11% in high-grade serous disease. Seventeen EOC cases carried a mutation in a mismatch repair gene, including 10 MSH6 mutation carriers (0.45%) and 4 MSH2 mutation carriers (0.18%). At least 1 in 10 women with high-grade serous EOC has a BRCA1 or BRCA2 mutation. The development of next generation sequencing technologies enables rapid mutation screening for multiple susceptibility genes at once, suggesting that routine clinical testing of all incidence cases should be considered. 
 655Val and 1170Pro ERBB2 SNPs in familial breast cancer risk and BRCA1 alterations. Human ERBB2 presents several SNPs. One of these, Ile655Val, introduces a structural change in the transmembrane region of ERBB2 and has been the focus of debate over its potential role as a susceptibility marker for breast cancer risk. Another SNP, Ala1170Pro, introduces a structural change in the carboxyl-terminal regulatory domain of the protein, but its clinical and biological importance remains undefined. The aim of this study was to investigate the association of rare alleles of both SNPs and the risk of developing breast cancer, BRCA1 alterations and clinical-pathological features of Caucasian breast cancer patients with familial history of breast/ovarian cancer. The originality of the present paper is that it is the only specifically focusing on the relationship between ERBB2 SNPs and familiarity/BRCA1 characteristics. A consecutive series of 628 patients with first diagnosis of breast cancer and 169 healthy people had DNA analyzed for both SNPs. Genotypic or allelic frequencies of ERBB2 SNPs in breast cancer patients were similar than in controls. The variant allele 655Val was significantly associated with younger age (p=0.009) particularly associated with patient family history of breast cancer (p=0.02). The 655Val allele was also more commonly found in   invasive  , while the variant 1170Pro in estrogen receptor positive breast cancers. Furthermore, this last SNP seems to be strictly associated with the presence of BRCA1 polymorphisms. In conclusion, these findings point to the existence of an association of ERBB2 allelic variants at both loci with specific breast tumor phenotypes and to the need of deeply investigate different gene SNPs association for risk defining. 
 Long-term ovarian cancer survival associated with mutation in BRCA1 or BRCA2. Studies have suggested that the 5-year survival of women with ovarian cancer and a BRCA1 or BRCA2 mutation is better than expected. We sought to evaluate the impact of carrying a BRCA1 or BRCA2 mutation on long-term survival of women after a diagnosis of   invasive   ovarian cancer.One thousand six hundred twenty-six unselected women diagnosed with   invasive   ovarian cancer in Ontario, Canada, or in Tampa, Florida, between 1995 and 2004 were followed for a mean of 6.9 years (range = 0.3 to 15.7 years). Mutation screening for BRCA1 and BRCA2 revealed mutations in 218 women (13.4%). Left-truncated survival analysis was conducted to estimate ovarian cancer-specific survival at various time points after diagnosis for women with and without mutations.In the 3-year period after diagnosis, the presence of a BRCA1 or BRCA2 mutation was associated with a better prognosis (adjusted hazard ratio = 0.68, 95% confidence interval [CI] = 0.48 to 0.98; P = .03), but at 10 years after diagnosis, the hazard ratio was 1.00 (95% CI = 0.83 to 1.22; P = .90). Among women with serous ovarian cancers, 27.4% of women who were BRCA1 mutation carriers, 27.7% of women who were BRCA2 carriers, and 27.1% of women who were noncarriers were alive at 12 years past diagnosis.For women with   invasive   ovarian cancer, the short-term survival advantage of carrying a BRCA1 or BRCA2 mutation does not lead to a long-term survival benefit. 
 Prevalence of BRCA1 and BRCA2 germ line mutations among women with carcinoma of the fallopian tube. The purpose of this study is to determine the prevalence of BRCA1 and BRCA2 mutations among a large series of women with carcinoma of the fallopian tube.Two series of women diagnosed with carcinoma of the fallopian tube were studied. Women identified from the Ontario Cancer Registry who were diagnosed with fallopian tube cancer between 1990 and 1998 and between 2002 and 2004. A second, hospital-based series was identified at Cedars Sinai Medical Centre, Los Angeles, California. These women were diagnosed between 1991 and 2007. Each subject was approached to provide her family history and ethnic background and to provide a blood sample for genetic testing for mutations in the BRCA1 and BRCA2 genes.In total, 108 patients with fallopian tube cancer were recruited (70 from Ontario and 38 from Los Angeles). Thirty-three patients (30.6%) were found to have a deleterious mutation; 23 in BRCA1 (21.3%) and 10 in BRCA2 (9.3%). The prevalence of mutations was 55.6% in Jewish women and was 26.4% in non-Jewish women. A family history of ovarian or breast cancer was positive for 24 women (23.3%); of these, 14 had a mutation (58.3%). Fourteen (14.4%) of the patients had a previous history of breast cancer; of these, 10 (71.4%) had a mutation. 40.3% of the women who were diagnosed with fallopian tube cancer before age 60 had a mutation, compared with 17.4% of the women diagnosed at age 60 and above.Approximately 30% of women with fallopian tube cancer have a mutation in BRCA1 or BRCA2. The highest frequencies of BRCA mutations were seen in women with fallopian tube cancer diagnosed under age 60, in Jewish women, in women with a family history of breast or ovarian cancer, and in women with a personal history of breast cancer. All patients diagnosed with   invasive   fallopian tube cancer should be considered candidates for genetic testing. 
 Prevalence and predictors of loss of wild type BRCA1 in estrogen receptor positive and negative BRCA1-associated breast cancers. The majority of breast cancers that occur in BRCA1 mutation carriers (BRCA1 carriers) are estrogen receptor-negative (ER-). Therefore, it has been suggested that ER negativity is intrinsic to BRCA1 cancers and reflects the cell of origin of these tumors. However, approximately 20% of breast cancers that develop in BRCA1 carriers are ER-positive (ER+); these cancers are more likely to develop as BRCA1 carriers age, suggesting that they may be incidental and unrelated to BRCA1 deficiency. The purpose of this study was to compare the prevalence of loss of heterozygosity due to loss of wild type (wt) BRCA1 in ER+ and ER- breast cancers that have occurred in BRCA1 carriers and to determine whether age at diagnosis or any pathologic features or biomarkers predict for loss of wt BRCA1 in these breast cancers.Relative amounts of mutated and wt BRCA1 DNA were measured by quantitative polymerase chain reaction performed on laser capture microdissected cancer cells from 42 ER+ and 35 ER-   invasive   breast cancers that developed in BRCA1 carriers. BRCA1 gene methylation was determined on all cancers in which sufficient DNA was available. Immunostains for cytokeratins (CK) 5/6, 14, 8 and 18, epidermal growth factor receptor and p53 were performed on paraffin sections from tissue microarrays containing these cancers.Loss of wt BRCA1 was equally frequent in ER+ and ER- BRCA1-associated cancers (81.0% vs 88.6%, respectively; P = 0.53). One of nine cancers tested that retained wt BRCA1 demonstrated BRCA1 gene methylation. Age at diagnosis was not significantly different between first   invasive   ER+ BRCA1 breast cancers with and without loss of wt BRCA1 (mean age 45.2 years vs 50.1 years, respectively; P = 0.51). ER+ BRCA1 cancers that retained wt BRCA1 were significantly more likely than those that lost wt BRCA1 to have a low mitotic rate (odds ratio (OR), 5.16; 95% CI, 1.91 to ∞). BRCA1 cancers with loss of wt BRCA1 were more likely to express basal cytokeratins CK 5/6 or 14 (OR 4.7; 95% CI, 1.85 to ∞).We found no difference in the prevalence of loss of wt BRCA1 between ER+ and   ER- inva  sive BRCA1-associated breast cancers. Our findings suggest that many of the newer therapies for BRCA1 breast cancers designed to exploit the BRCA1 deficiency in these cancers may also be effective in ER+ cancers that develop in this population. 
 EZH2 participates in malignant biological behavior of epithelial ovarian cancer through regulating the expression of BRCA1. Aberrant overexpression of the enhancer of zeste homolog 2 (EZH2), a histone methyltransferase inhibiting targets expression via epigenetic mechanisms, is associated with an   invasive   phenotype and drug resistance in ovarian cancer. Breast cancer 1 (BRCA1) gene is a well-recognized tumor suppressor, whose downregulation plays a key role in the development of ovarian cancer. In the present study, we found depletion of EZH2 increased BRCA1 protein expression and promoted its nuclear translocation, but decreased BRCA1 mRNA expression. Treatment with the Akt-1 activator insulin-like growth factor-1 (IGF-1) prevented EZH2-induced BRCA1 nuclear/cytoplasmic shuttling. Loss of BRCA1 partially rescued the effects of EZH2 downregulation on proliferation, G 2/M transition, and migration in ovarian cancer cells. However, in a cisplatin-resistant sub-line of A2780 (A2780/DDP), both EZH2 and BRCA1 were overexpressed compared with parental A2780 cells and depletion of EZH2 reduced BRCA1 expression at both mRNA and protein levels. Downregulation of EZH2 or BRCA1 sensitized A2780/DDP cells to cisplatin, whereas simultaneous inhibition of them only resulted in modest resensitization instead of showing any synergistic effect because EZH2 expression was reactivated when BRCA1 expression was very low. Accordingly, our results suggest the expression of BRCA1 is modulated by EZH2 in epithelial ovarian cancer and BRCA1 is required for the effects of EZH2 downregulation on biological behaviors of tumor cells. 
 Heterozygote BRCA1 status and skewed chromosome X inactivation. A high frequency of skewed X-chromosome inactivation has been reported in peripheral blood lymphocytes from early onset breast cancer or   invasive   ovarian cancer patients. Recent findings have shown that breast and ovarian carcinoma cells from BRCA1 mutation carrier women lack the hallmarks of inactive X chromatin structure. These observations suggested that loss of functional BRCA1 in female cells may perturb the process of X inactivation and have lead us to the hypothesis that analysis of skewing could be used as a predictive test for BRCA1 germline mutation in lymphocytes from breast cancer patients. In the present study, we have compared the X inactivation pattern in lymphoblastoid cell lines from 38 females carrying heterozygous BRCA1 mutation to 41 controls. X inactivation analysis was assessed on the polymorphic CAG repeat within the human androgen receptor gene. Our observations rule out an effect of a monoallelic BRCA1 germline mutation on the choice of inactivated chromosome X and therefore the possibility of using analysis of Xi skewing as a predictive test for BRCA1 germline mutation carrier status. 
 Endometrial cancers in mutation carriers from hereditary breast ovarian cancer syndrome kindreds: report from the Creighton University Hereditary Cancer Registry with review of the implications. The aim of this study was to categorize and report endometrial cancers in mutation carriers from hereditary breast ovarian cancer families.Our Hereditary Cancer Registry was searched for gynecologic and peritoneal cancers linked to mutations in BRCA1 or BRCA2.   Invasive   cancers were registered in 101 mutation carriers with complete pathology reports. Efforts were made to secure diagnostic surgical pathology tissues for review. All records and available diagnostic slides were meticulously studied, and primary cancers were classified.Eight malignancies were classified as primary endometrial cancers. Five of these were low- or intermediate-grade endometrioid carcinomas, and 3 were pure serous carcinomas or contained serous carcinoma elements mixed with high-grade endometrioid carcinoma. Breast cancers were diagnosed in 5 patients before and in 1 patient after endometrial carcinoma. Three endometrioid carcinomas were preceded by estrogen treatment, 2 for many years and the other for only 2 months, and 2 of the patients with serous carcinoma had been treated with tamoxifen.The finding that 8 of gynecologic and peritoneal cancers in 101 mutation carriers were endometrial cancers with a smaller proportion of endometrioid carcinomas than reported in general populations is added to the current controversial literature on endometrial cancer, particularly regarding serous carcinomas, in hereditary breast ovarian cancer syndrome. Well-designed prospective programs for standardized surgical and pathologic handling, processing, and reporting are essential for working out the pathogenesis, true risks, and best management of this disease in carriers of deleterious BRCA1 and BRCA2 germline mutations. 
 BRCA1-IRIS overexpression promotes formation of aggressive breast cancers. Women with HER2(+) or triple negative/basal-like (TN/BL) breast cancers succumb to their cancer rapidly due, in part to acquired Herceptin resistance and lack of TN/BL-targeted therapies. BRCA1-IRIS is a recently discovered, 1399 residue, BRCA1 locus alternative product, which while sharing 1365 residues with the full-length product of this tumor suppressor gene, BRCA1/p220, it has oncoprotein-like properties. Here, we examine whether BRCA1-IRIS is a valuable treatment target for HER2(+) and/or TN/BL tumors.Immunohistochemical staining of large cohort of human breast tumor samples using new monoclonal anti-BRCA1-IRIS antibody, followed by correlation of BRCA1-IRIS expression with that of AKT1, AKT2, p-AKT, survivin and BRCA1/p220, tumor status and age at diagnosis. Generation of subcutaneous tumors in SCID mice using human mammary epithelial (HME) cells overexpressing TERT/LT/BRCA1-IRIS, followed by comparing AKT, survivin, and BRCA1/p220 expression, tumor status and aggressiveness in these tumors to that in tumors developed using TERT/LT/Ras(V12)-overexpressing HME cells. Induction of primary and   invasive   rat mammary tumors using the carcinogen N-methyl-N-nitrosourea (NMU), followed by analysis of rat BRCA1-IRIS and ERα mRNA levels in these tumors. High BRCA1-IRIS expression was detected in the majority of human breast tumors analyzed, which was positively correlated with that of AKT1-, AKT2-, p-AKT-, survivin, but negatively with BRCA1/p220 expression. BRCA1-IRIS-positivity induced high-grade, early onset and metastatic HER2(+) or TN/BL tumors. TERT/LT/BRCA1-IRIS overexpressing HME cells formed   invasiv  e subcutaneous tumors that express high AKT1, AKT2, p-AKT and vimentin, but no CK19, p63 or BRCA1/p220. NMU-induced primary and   invasiv  e rat breast cancers expressed high levels of rat BRCA1-IRIS mRNA but low levels of rat ERα mRNA.BRCA1-IRIS overexpression triggers aggressive breast tumor formation, especially in patients with HER2(+) or TN/BL subtypes. We propose that BRCA1-IRIS inhibition may be pursued as a novel therapeutic option to treat these aggressive breast tumor subtypes. 
 Improved survival in BRCA2 carriers with ovarian cancer. The objective of this study was to investigate survival of ovarian cancer patients with BRCA1 and BRCA2 mutations compared to those without mutations in a population-based sample of incident epithelial ovarian cancer cases.Follow-up for vital status was performed on a population-based sample of 232 women with incident epithelial ovarian cancer recruited between December 13, 2000 and September 30, 2003 in the Tampa Bay area. Survival analysis using Cox regression was performed on (1) all 232 cases and (2) the 209   invasive   epithelial ovarian cancer cases. Results of the two analyses were similar, thus data involving the 209   invasive   epithelial cancer cases are presented, as this was judged to be more clinically relevant.In the multivariate analysis, BRCA status and stage were statistically significant, and were adjusted for in the survival analysis model. The Kaplan-Meier method estimated expected survival at 4 years of 83% of BRCA2 carriers compared to 37% of BRCA1 carriers and 12% of non-carriers. There was a statistically significant difference between BRCA2 carriers and non-carriers (p = 0.013). No statistically significant survival differences were seen for BRCA1 carriers when compared with either BRCA2 carriers or non-carriers.These data suggest that BRCA2 mutation carriers with ovarian cancer may have better survival than BRCA1 carriers and non-carriers. The etiology of this possible survival advantage is currently unknown. Larger studies are needed to confirm these results and to clarify their etiology and clinical significance. 
 The spectrum of BRCA1 and BRCA2 mutations in breast cancer patients in the Bahamas. We sought to identify the full range of founder mutations in BRCA1 and BRCA2 in the Bahamas and to estimate the proportion of all BRCA1 and BRCA2 mutations that are accounted for by founder mutations. We studied 214 Bahamian women with   invasive   breast cancer, unselected for age or family history. A founder mutation had previously been identified in 49 patients. We conducted full sequencing of the BRCA1 and BRCA2 genes and multiplex ligation-dependent probe amplification (MLPA) for 156 patients. A novel founder mutation in BRCA2 (exon 17 818delA) was seen in four different patients and five other unique mutations in BRCA1 and BRCA2, including a large deletion (exons 8-9) in BRCA1. In total, a mutation was seen in 58 of the 214 patients (27%); 92% of carriers carried one of the seven founder mutations. Approximately 27% of unselected cases of breast cancer in the Bahamian population are attributable to a mutation in BRCA1 or BRCA2, a prevalence which far exceeds that of any other country. The majority of women who carry a mutation in the Bahamas, carry one of the seven founder mutations, making it possible to offer genetic testing to all women at risk for breast cancer in the Bahamas. 
 Histone methyltransferase EZH2 induces Akt-dependent genomic instability and BRCA1 inhibition in breast cancer. Increased levels of EZH2, a critical regulator of cellular memory, signal the presence of metastasis and poor outcome in breast cancer patients. High levels of EZH2 are associated with nuclear pleomorphism, lack of estrogen receptor expression, and decreased nuclear levels of BRCA1 tumor suppressor protein in   invasive   breast carcinomas. The mechanism by which EZH2 overexpression promotes the growth of poorly differentiated   invasive   carcinomas remains to be defined. Here, we show that EZH2 controls the intracellular localization of BRCA1 protein. Conditional doxycycline-induced upregulation of EZH2 in benign mammary epithelial cells results in nuclear export of BRCA1 protein, aberrant mitoses with extra centrosomes, and genomic instability. EZH2 inhibition in CAL51 breast cancer cells induces BRCA1 nuclear localization and rescues defects in ploidy and mitosis. Mechanistically, EZH2 overexpression is sufficient for activation of the phosphoinositide 3-kinase/Akt (PI3K/Akt) pathway specifically through activation of Akt isoform 1. EZH2-induced BRCA1 nuclear export, aneuploidy, and mitotic defects were prevented by treatment with the PI3K inhibitors LY294002 or wortmannin. Targeted inhibition of Akt-1, Akt-2, and Akt-3 isoforms revealed that the EZH2-induced phenotype requires specific activation of Akt-1. The relevance of our studies to human breast cancer is highlighted by the finding that high EZH2 protein levels are associated with upregulated expression of phospho-Akt-1 (Ser473) and decreased nuclear expression of phospho-BRCA1 (Ser1423) in 39% of   invasive   breast carcinomas. These results enable us to pinpoint one mechanism by which EZH2 regulates BRCA1 expression and genomic stability mediated by the PI3K/Akt-1 pathway. 
 Breast cancer risk and 6q22.33: combined results from Breast Cancer Association Consortium and Consortium of Investigators on Modifiers of BRCA1/2. Recently, a locus on chromosome 6q22.33 (rs2180341) was reported to be associated with increased breast cancer risk in the Ashkenazi Jewish (AJ) population, and this association was also observed in populations of non-AJ European ancestry. In the present study, we performed a large replication analysis of rs2180341 using data from 31,428   invasive   breast cancer cases and 34,700 controls collected from 25 studies in the Breast Cancer Association Consortium (BCAC). In addition, we evaluated whether rs2180341 modifies breast cancer risk in 3,361 BRCA1 and 2,020 BRCA2 carriers from 11 centers in the Consortium of Investigators of Modifiers of BRCA1/2 (CIMBA). Based on the BCAC data from women of European ancestry, we found evidence for a weak association with breast cancer risk for rs2180341 (per-allele odds ratio (OR) = 1.03, 95% CI 1.00-1.06, p = 0.023). There was evidence for heterogeneity in the ORs among studies (I(2) = 49.3%; p = <0.004). In CIMBA, we observed an inverse association with the minor allele of rs2180341 and breast cancer risk in BRCA1 mutation carriers (per-allele OR = 0.89, 95%CI 0.80-1.00, p = 0.048), indicating a potential protective effect of this allele. These data suggest that that 6q22.33 confers a weak effect on breast cancer risk. 
 Occurrence of both breast and ovarian cancer in a woman is a marker for the BRCA gene mutations: a population-based study from western Sweden. This study aimed to analyze whether the occurrence of both breast and ovarian cancer in a woman serves as a marker for BRCA gene mutations.This population-based study included 256 women in western Sweden who developed both   invasive   breast and ovarian tumors between 1958 and 1999. Archival paraffin tissue blocks of their tumors were retrieved for DNA-extraction to analyze the founder mutation, BRCA1 c.3171_3175dup (c.3171ins5), which is most common in this geographic area and four other common Scandinavian BRCA1 gene mutations and one BRCA2 mutation. Together, account these mutations for approximately 75% of the BRCA1/2 gene mutations in the clinical unit.Ninteen percent (95% confidence interval (CI) 14-24%) of the women carried one of the analyzed BRCA1 gene mutations but none of the women were positive for the analyzed BRCA2 mutation. One-third of the women with both tumors before age 60 were mutation carriers. BRCA1 c.3171_3175dup (c.3171ins5) constituted 84% of all identified mutations. Although the majority of breast cancers were   invasive   ductal and atypical medullary types, a variety of other breast malignancies were seen among mutation carriers. Serous ovarian carcinomas predominated among ovarian tumors. A variety of other ovarian tumors, including three granulosa-theca cell tumors, were also observed among mutation carriers.The occurrence of both breast and ovarian cancer in a woman is associated with a high likelihood of a constitutional BRCA1 mutation. These women and their families might therefore be considered for mutation screening after appropriate genetic counselling. 
 Identification of a preneoplastic gene expression profile in tubal epithelium of BRCA1 mutation carriers. Micro  invasive   carcinomas and high-grade intraepithelial neoplasms are commonly discovered within the fallopian tube of BRCA1 mutation carriers at the time of risk-reducing salpingo-oophorectomy, suggesting that many BRCA1-mutated ovarian carcinomas originate in tubal epithelium. We hypothesized that changes in gene expression profiles within the histologically normal fallopian tube epithelium of BRCA1 mutation carriers would overlap with the expression profiles in BRCA1-mutated ovarian carcinomas and represent a BRCA1 preneoplastic signature. Laser capture microdissection of frozen sections was used to isolate neoplastic cells or histologically normal fallopian tube epithelium, and expression profiles were generated on Affymetrix U133 Plus 2.0 gene expression arrays. Normal-risk controls were 11 women wild type for BRCA1 and BRCA2 (WT-FT). WT-FT were compared with histologically normal fallopian tube epithelium from seven women with deleterious BRCA1 mutations who had foci of at least intraepithelial neoplasm within their fallopian tube (B1-FTocc). WT-FT samples were also compared with 12 BRCA1 ovarian carcinomas (B1-CA). The comparison of WT-FT versus B1-FTocc resulted in 152 differentially expressed probe sets, and the comparison of WT-FT versus B1-CA resulted in 4079 differentially expressed probe sets. The BRCA1 preneoplastic signature was composed of the overlap between these two lists, which included 41 concordant probe sets. Genes in the BRCA1 preneoplastic signature included several known tumor suppressor genes such as CDKN1C and EFEMP1 and several thought to be important in invasion and metastasis such as E2F3. The expression of a subset of genes was validated with quantitative reverse transcription-polymerase chain reaction and immunohistochemistry. 
 Frequency of 5382insC mutation of BRCA1 gene among breast cancer patients: an experience from Eastern India. The incidence of breast cancer in India is on the rise and is rapidly becoming the number one cancer in females pushing the cervical cancer to the second position. The mutations in two breast cancer susceptibility genes, BRCA1 and BRCA2, are frequently associated with familial breast cancer. The main objective of the study was to determine the frequency of the mutation 5382insC in BRCA1 of eastern Indian breast cancer patients and also study the hormonal receptor status and histopathology of the patients. Altogether 92 patients affected with breast cancer were included in this study. ARMS-PCR based amplification was used to detect the presence of mutation. The mutations were considered only after pedigree analysis. Out of 92 patients (age range: 20-77 years) with family history (57 individuals) and without family history (35 individuals) were screened. Fifty controls have been systematically investigated. Seven patients and two family members were found to be carriers of 5382insC mutation in BRCA1 gene. We have found 42.64 % ER(-)/PR(-) cancer and 20.58 % triple negative cancer.   Invasive   ductal carcinoma is the most common histology among the investigated individuals. The presented data confirm a noticeable contribution of BRCA1 5382insC mutation in BC development in Eastern India, which may justify an extended BRCA1 5382insC testing within this patient population. We found HER-2/neu negativity and BRCA1 positivity associated with familial breast cancer. From the hospital's patient history, it was revealed that the age of menarche plays an important role in development of breast cancer. 
 A role of BRCA1 and BRCA2 germline mutations in breast cancer susceptibility within Sardinian population. In recent years, numerous studies have assessed the prevalence of germline mutations in BRCA1 and BRCA2 genes in various cohorts. We here extensively investigated the prevalence and geographical distribution of BRCA1-2 mutations in the entire genetically-homogeneous Sardinian population. The occurrence of phenotypic characteristics which may be predictive for the presence of BRCA1-2 germline mutations was also evaluated.Three hundred and forty-eight breast cancer patients presenting a familial recurrence of   invasive   breast or ovarian carcinoma with at least two affected family members were screened for BRCA1-2 mutations by DHPLC analysis and DNA sequencing. Association of BRCA1 and BRCA2 mutational status with clinical and pathological parameters was evaluated by Pearson's Chi-Squared test.Overall, 8 BRCA1 and 5 BRCA2 deleterious mutations were detected in 35/348 (10%) families; majority (23/35;66%) of mutations was found in BRCA2 gene. The geographical distribution of BRCA1-2 mutations was related to three specific large areas of Sardinia, reflecting its ancient history: a) the Northern area, linguistically different from the rest of the island (where a BRCA2 c.8764_8765delAG mutation with founder effect was predominant); b) the Middle area, land of the ancient Sardinian population (where BRCA2 mutations are still more common than BRCA1 mutations); and c) the South-Western area, with many Phoenician and Carthaginian locations (where BRCA1 mutations are prevalent). We also found that phenotypic features such as high tumor grading and lack of expression of estrogen/progesterone receptors together with age at diagnosis and presence of ovarian cancer in the family may be predictive for the presence of BRCA1-2 germline mutations. 
 DNA methylation profiling in doxorubicin treated primary locally advanced breast tumours identifies novel genes associated with survival and treatment response. Breast cancer is the most frequent cancer in women and consists of a heterogeneous collection of diseases with distinct histopathological, genetic and epigenetic characteristics. In this study, we aimed to identify DNA methylation based biomarkers to distinguish patients with locally advanced breast cancer who may benefit from neoadjuvant doxorubicin treatment.We investigated quantitatively the methylation patterns in the promoter regions of 14 genes (ABCB1, ATM, BRCA1, CDH3, CDKN2A, CXCR4, ESR1, FBXW7, FOXC1, GSTP1, IGF2, HMLH1, PPP2R2B, and PTEN) in 75 well-described pre-treatment samples from locally advanced breast cancer and correlated the results to the available clinical and molecular parameters. Six normal breast tissues were used as controls and 163 unselected breast cancer cases were used to validate associations with histopathological and clinical parameters.Aberrant methylation was detected in 9 out of the 14 genes including the discovery of methylation at the FOXC1 promoter. Absence of methylation at the ABCB1 promoter correlated with progressive disease during doxorubicin treatment. Most importantly, the DNA methylation status at the promoters of GSTP1, FOXC1 and ABCB1 correlated with survival, whereby the combination of methylated genes improved the subdivision with respect to the survival of the patients. In multivariate analysis GSTP1 and FOXC1 methylation status proved to be independent prognostic markers associated with survival.Quantitative DNA methylation profiling is a powerful tool to identify molecular changes associated with specific phenotypes. Methylation at the ABCB1 or GSTP1 promoter improved overall survival probably due to prolonged availability and activity of the drug in the cell while FOXC1 methylation might be a protective factor against tumour   invasive  ness. FOXC1 proved to be general prognostic factor, while ABCB1 and GSTP1 might be predictive factors for the response to and efficacy of doxorubicin treatment. Pharmacoepigenetic effects such as the reported associations in this study provide molecular explanations for differential responses to chemotherapy and it might prove valuable to take the methylation status of selected genes into account for patient management and treatment decisions. 
 Histopathological criteria and selection algorithms for BRCA1 genetic testing. To ensure targeted treatment, it would be useful to know at the time of diagnosis whether a BRCA mutation is causally related to an individual breast cancer. The aim of this study was to investigate in an unselected series of breast cancer patients the value of incorporating morphological and immunohistochemical features for the selection of patients who may benefit from BRCA1 genetic testing. In a retrospective approach, histopathological results of tumors from 897 women were reevaluated regarding age at diagnosis, subtype of cancer, tumor grade, and estrogen (ER), progesterone (PR), and Her2/neu receptor status, as well as p53 and Ki67 status. In all, 142 tumors fulfilled morphological criteria indicative of a BRCA1 mutation. Of the 59 women willing to participate, 26 women concomitantly showed a positive family history. Pathogenic BRCA1 germline mutations were detected in 7 of 18 women (39%) (95% confidence interval = 0.17-0.64). All BRCA1-associated tumors were of high grade,   invasive  -ductal subtype, and PR and Her2/neu negative, and 91% of the tumors were negative for ER; 60% of the tumors showed a high expression of p53 and 60% a high expression of Ki67. There was a significant difference with respect to grading (P = 0.001 for G3), ER negativity (P = 0.0075), Ki67 > or = 65% (P = 0.0039), and triple negativity (i.e., ER(-), PR(-), Her2/neu(-)) (P = 0.0019) between tumors of mutation carriers and noncarriers. 
 The contribution of founder mutations to early-onset breast cancer in French-Canadian women. In an ethnically-homogeneous population, it is valuable to identify founder mutations in cancer-predisposing genes. Founder mutations have been found in four breast-cancer-predisposing genes in French-Canadian breast cancer families. The frequencies of the mutant alleles have been measured neither in a large series of unselected breast cancer patients from Quebec, nor in healthy controls. These estimates are necessary to measure their contribution to the hereditary burden of breast cancer in Quebec and to help develop genetic screening policies which are appropriate for the province. We studied 564 French-Canadian women with early-onset   invasive   breast cancer who were treated at a single Montreal hospital. Patients had been diagnosed at age 50 or less, and were ascertained between 2004 and 2008. We screened all 564 patients for nine founder mutations: four in BRCA1, three in BRCA2 and one each in PALB2 and CHEK2. We also studied 6433 DNA samples from newborn infants from the Quebec City area to estimate the frequency of the nine variant alleles in the French-Canadian population. We identified a mutation in 36 of the 564 breast cancer cases (6.4%) and in 35 of 6443 controls (0.5%). In the breast cancer patients, the majority of mutations were in BRCA2 (54%). However, in the general population (newborn infants), the majority of mutations were in CHEK2 (54%). The odds ratio for breast cancer to age 50, given a BRCA1 mutation, was 10.1 (95% CI: 3.7-28) and given a BRCA2 mutation was 29.5 (95% CI: 12.9-67). The odds ratio for breast cancer to age 50, given a CHEK2 mutation, was 3.6 (95% CI: 1.4-9.1). One-half of the women with a mutation had a first- or second-degree relative diagnosed with breast or ovarian cancer. Thus, it can be concluded that a predisposing mutation in BRCA1, BRCA2, CHEK2 or PALB2 is present in approximately 6% of French-Canadian women with early-onset breast cancer. It is reasonable to offer screening for founder mutations to all French-Canadian women with breast cancer before age 50. The frequency of these mutations in the general population (0.5%) is too low to advocate population-based screening. 
 Breast cancer size estimation with MRI in BRCA mutation carriers and other high risk patients. To assess the value of breast MRI in size assessment of breast cancers in high risk patients, including those with a BRCA 1 or 2 mutation. Guidelines recommend invariably breast MRI screening for these patients and therapy is thus based on these findings. However, the accuracy of breast MRI for staging purposes is only tested in sporadic cancers.We assessed concordance of radiologic staging using MRI with histopathology in 49 tumors in 46 high risk patients (23 BRCA1, 12 BRCA2 and 11 Non-BRCA patients). The size of the total tumor area (TTA) was compared to pathology. In   invasive   carcinomas (n=45) the size of the largest focus (LF) was also addressed.Correlation of MRI measurements with pathology was 0.862 for TTA and 0.793 for LF. TTA was underestimated in 8(16%), overestimated in 5(10%), and correctly measured in 36(73%) cases. LF was underestimated in 4(9%), overestimated in 5(11%), and correctly measured in 36(80%) cases. Impact of BRCA 1 or 2 mutations on the quality of size estimation was not observed.Tumor size estimation using breast MRI in high risk patients is comparable to its performance in sporadic cancers. Therefore, breast MRI can safely be used for treatment planning. 
 Pathologic features and immunophenotype of estrogen receptor-positive breast cancers in BRCA1 mutation carriers. Although most breast cancers in BRCA1 mutation carriers are estrogen receptor negative (ER-) with a basal-like phenotype, up to one third are ER positive (ER+). Little is known about the characteristics of this subgroup. To address this, we compared histologic and immunophenotypic features of 60 BRCA1-related ER+ breast cancers with those of 85 BRCA1-related ER- cancers and 174 matched ER+ sporadic cancers. ER+ BRCA1-related cancers were significantly less likely than ER- BRCA1-related cancers to be of pure   invasive   ductal type (P<0.001) and to be of histologic grade 3 (P<0.001), and less frequently to have a high mitotic rate (P<0.001), pushing (or unknown) margins (P<0.001), a moderate/marked lymphocytic infiltrate (P=0.003), or geographic necrosis/fibrotic focus (P<0.001). In addition, ER+ BRCA1-related cancers less often expressed CK5/6 (P<0.0001), CK14 (P<0.0001), and epidermal growth factor receptor (P<0.0001) and more often expressed progesterone receptor (P<0.0001). In contrast, when compared with ER+ sporadic cancers, ER+ BRCA1-related cancers were significantly more often of   invasive   ductal type (P=0.005) and of histologic grade 3 (P=0.006), more frequently had a high mitotic rate (P=0.0003), and were more often CK14+ (P=0.03). On unsupervised cluster analysis, some ER+ BRCA1 cancers clustered more closely with sporadic ER+ cancers, whereas others clustered more closely with ER- BRCA1-related cancers. Nuclear expression levels of poly(ADP) ribose polymerase 1 in ER+ BRCA1-related cancers were similar to those in ER- BRCA1-related cancers but significantly higher than in ER+ sporadic cancers. We conclude that ER+ BRCA1-related breast cancers show several morphologic and immunophenotypic differences from ER+ sporadic breast cancers as well as some similarities to ER- BRCA1-related cancers. 
 Expression profile of BRCA1 and BRCA2 genes in premenopausal Mexican women with breast cancer: clinical and immunohistochemical correlates. Low BRCA1 gene expression is associated with increased   invasive  ness and influences the response of breast carcinoma (BC) to chemotherapeutics. However, expression of BRCA1 and BRCA2 genes has not been completely characterized in premenopausal BC. We analyzed the clinical and immunohistochemical correlates of BRCA1 and BRCA2 expression in young BC women. We studied 62 women (mean age 38.8 years) who developed BC before the age of 45 years. BRCA1 and BRCA2 mRNA expression was assessed by reverse transcriptase-polymerase chain reaction (RT-PCR) and that of HER-2 and p53 proteins by immunohistochemistry. Body mass index (BMI) > or = 27 (52%) and a declared family history of BC (26%) were the main risk factors. Ductal infiltrative adenocarcinoma was found in 86% of the cases (tumor size >5 cm in 48%). Disease stages I-IV occurred in 2, 40, 55, and 3%, respectively (73% implicating lymph nodes). Women aged < or = 35 years (24%) had more family history of cervical cancer, stage III/IV disease, HER-2 positivity, and lower BRCA1 expression than older women (P < 0.05). BRCA1 and BRCA2 expression correlated in healthy, but not in tumor tissues (TT). Neither BRCA1 nor BRCA2 expression was associated with tumor histology, differentiation, nodal metastasis or p53 and HER-2 expression. After multivariate analysis, only disease stage explained BRCA1 mRNA levels in the lowest quartile. Premenopausal BC has aggressive clinical and molecular characteristics. Low BRCA1 mRNA expression is associated mainly with younger ages and advanced clinical stage of premenopausal BC. BRCA2 expression is not associated with disease severity in young BC women. 
 A combined analysis of outcome following breast cancer: differences in survival based on BRCA1/BRCA2 mutation status and administration of adjuvant treatment. The prognostic significance of germline mutations in BRCA1 and BRCA2 in women with breast cancer remains unclear. A combined analysis was performed to address this uncertainty.Two retrospective cohorts of Ashkenazi Jewish women undergoing breast-conserving treatment for   invasive   cancer between 1980 and 1995 (n = 584) were established. Archived tissue blocks were used as the source of DNA for Ashkenazi Jewish BRCA1/BRCA2 founder mutation analysis. Paraffin-embedded tissue and follow-up information was available for 505 women.Genotyping was successful in 496 women, of whom 56 (11.3%) were found to carry a BRCA1/BRCA2 founder mutation. After a median follow-up period of 116 months, breast cancer specific survival was worse in women with BRCA1 mutations than in those without (62% at 10 years versus 86%; P < 0.0001), but not in women with the BRCA2 mutation (84% versus 86% at 10 years; P = 0.76). Germline BRCA1 mutations were an independent predictor of breast cancer mortality in multivariate analysis (hazard ratio 2.4, 95% confidence interval 1.2-4.8; P = 0.01). BRCA1 status predicted breast cancer mortality only among women who did not receive chemotherapy (hazard ratio 4.8, 95% confidence interval 2.0-11.7; P = 0.001). The risk for metachronous ipsilateral cancer was not greater in women with germline BRCA1/BRCA2 founder mutations than in those without mutations (P = 0.68).BRCA1 mutations, but not BRCA2 mutations, are associated with reduced survival in Ashkenazi women undergoing breast-conserving treatment for   invasive   breast cancer, but the poor prognosis associated with germline BRCA1 mutations is mitigated by adjuvant chemotherapy. The risk for metachronous ipsilateral disease does not appear to be increased for either BRCA1 or BRCA2 mutation carriers, at least up to 10 years of follow up. 
 Prevalence of BRCA1 and BRCA2 mutations in women with breast carcinoma In Situ and referred for genetic testing. Ductal and lobular carcinoma in situ (CIS) accounted for 62,280 (24.5%) of all new breast cancer diagnoses in 2009. BRCA1/2 mutations confer an extremely high risk of breast cancer, and management guidelines for BRCA1/2 mutation carriers advise close follow-up, intensive screening, and consideration of prophylactic surgery to lower this risk. The limited relevant previous data are not definitive in establishing the prevalence of BRCA1/2 mutations in breast CIS patients, creating uncertainty as to whether referral for cancer risk assessment and genetic testing is appropriate for this group. Therefore, we conducted a cross-sectional analysis of the Myriad Genetics BRCA1/2 database to determine the prevalence of these mutations in breast CIS patients. All statistical tests were 2-sided, and confidence intervals (CI) are reported at the 95% level (α = 0.05). The source population was 64,717 consecutive women who were not Ashkenazi Jewish, underwent BRCA1/2 testing, and provided a personal and family history of   invasiv  e breast and ovarian cancer; 7,295 (11.3%) reported a diagnosis of CIS (ductal or lobular) and had an overall 5.9% prevalence of mutated BRCA1/2 (mBRCA). Subgrouped by history (personal or family) of   invasiv  e breast and/or ovarian cancer, these CIS patients had the following prevalences of mBRCA: (1) no personal or family history, 2.3%; (2) personal history, 5.2%; (3) family history, 5%; and (4) personal and family history, 10.3%. mBRCA risk was significantly higher in women with early-onset (<50 years old) CIS than with late-onset (≥ 50 years old) CIS [odds ratio (OR) = 1.5; 95% CI = 1.1-2.1). Disease onset at less than 40 years age was associated with an even higher mBRCA risk (OR = 1.8; 95% CI = 1.3-2.3). By far the largest analysis of BRCA1/2 mutation prevalence in non-Ashkenazi Jewish breast CIS patients, this study shows that early-onset CIS is associated with mBRCA1/2 in patients referred for genetic testing. When a family history of breast and/or ovarian cancer are also present, testing women with early-onset CIS may increase both the likelihood of detecting BRCA1/2 mutations and opportunities for carriers to consider additional cancer prevention strategies. 
 Founder mutations in BRCA1/2 are not frequent in Canadian Ashkenazi Jewish men with prostate cancer. Relatives of BRCA1 and BRCA2 mutation carriers have long been proposed by epidemiological studies to have an increased risk of developing prostate cancer. In the Ashkenazi Jewish (AJ) population, the existence of 3 frequent founder mutations, 185delAG and 5382insC in BRCA1 and 6174delT in BRCA2 greatly facilitates screening for carriers.We tested 146 AJ men with confirmed diagnoses of   invasive   prostate cancer. Thirteen had at least one first degree relative with prostate cancer. The median age at diagnosis of participants was 67.9 years (range 48.6-84.2 years). Subjects were screened for the BRCA1:185delAG, BRCA1:5382insC and BRCA2:6174delT mutations simultaneously using a multiplex sizing assay detecting band shifts in the presence of the variant sequence.Two out of 146 individuals were found to carry the germline BRCA2 mutation 6174delT (1.4%); the previously reported population frequency for this mutation is ~1% in AJ. We found no BRCA1 mutations. One carrier had 2 uncles affected with prostate cancer, while the other had an uncle and daughter with breast cancer. We combined our results with previously published data examining these 3 founder AJ mutations in men with prostate cancer and in population controls. Including our results, studies to date reported 5/463 (1.1%), 2/293 (0.68%) and 7/461 (1.3%) carriers for the BRCA1:185delAG, BRCA1:5382insC and BRCA2:6174delT mutations in prostate cancer cases, respectively. This compares with combined reported frequencies of 85/9371 (0.91%), 24/8867 (0.27%) and 119/9514 (1.3%) for the same mutations in control individuals. There was no statistically significant excess of mutations in cases compared to controls in either gene.Our observations remain preliminary. By combining all studies published to date, we have an 80% power to detect ORs of 2.7, 6.6 and 2.5 (185delAG, 5382insC and 6174 delT, respectively) while the values we observed range between 1.0 and 2.5. However, the contribution of rare mutations with such low odds ratios to the population prostate cancer burden is unlikely to be large enough to be clinically useful. Thus, contrary to suggestions from some previous epidemiological data, our observations do not support an important role for AJ founder BRCA1/2 mutations in prostate cancer risk. 
 Ovarian epithelial dysplasia and prophylactic oophorectomy for genetic risk. To make an accurate histopathological description of ovarian dysplasia in a population at genetic risk of ovarian cancer and devise an ovarian dysplasia score.In this retrospective cohort study, 90 patients who had undergone bilateral oophorectomy or ovarian cystectomy between 1992 and 2005 and whose ovaries were reported as normal were divided into two groups: Group A comprising prophylactic oophorectomies for genetic predisposition (N = 28), and Group B or control group, fertile and non-cancerous (N = 62). Eleven epithelial cytological and architectural features were defined. Ovaries were analysed and reviewed by four pathologists blinded to clinical data. An ovarian dysplasia score was devised to quantify extent of ovarian epithelial abnormalities. The degrees of ovarian epithelial abnormalities (dysplasia scores) were compared between the two groups.Mean dysplasia score was significantly higher in Group A (prophylactic oophorectomies) than in Group B (control group) (9.67 vs. 4.19, P < 0.001). In Group A, we observed a gradation in the severity of the dysplastic lesions between (i) proven BRCA mutations and prophylactic oophorectomies without mutations (11.26 vs. 8.1), and (ii) according to age (10.27 after age 50 years vs. 8.6 before age 50 years, P = 0.2962).These results suggest abnormalities in ovaries from high risk women. The ovarian dysplasia may be a pre-malignant, non-  invasive   histological lesion that could be an important step in early neoplasia. 
 Factors associated with genetic counseling and BRCA testing in a population-based sample of young Black women with breast cancer. Concerns about the potential for genomic advances to increase health disparities have been raised. Thus, it is important to assess referral and uptake of genetic counseling (GC) and testing in minority populations at high risk for hereditary breast and ovarian cancer (HBOC). Black women diagnosed with   invasive   breast cancer ≤age 50 in 2009-2012 were recruited through the Florida State Cancer Registry 6-18 months following diagnosis and completed a baseline questionnaire. Summary statistics, Chi-square tests, and path modeling were conducted to examine which demographic and clinical variables were associated with referral and access to genetic services. Of the 440 participants, all met national criteria for GC, yet only 224 (51 %) were referred for or received GC and/or HBOC testing. Variables most strongly associated with healthcare provider referral for GC included having a college education (OR 2.1), diagnosis at or below age 45 (OR 2.0), and triple negative tumor receptor status (OR 1.7). The strongest association with receipt of GC and/or HBOC testing was healthcare provider referral (OR 7.9), followed by private health insurance at diagnosis (OR 2.8), and household income greater than $35,000 in the year prior to diagnosis (OR 2.0). Study findings suggest efforts are needed to improve genetic services access among a population-based sample of high-risk Black women. These results indicate that socioeconomic factors and physician referral patterns contribute to disparities in access to genetic services within this underserved minority population. 
 Reduced cell death,   invasive   and angiogenic features conferred by BRCA1-deficiency in mammary epithelial cells transformed with H-Ras. To investigate the role of tumor suppressors BRCA1 and p53 proteins in human breast tumorigenesis, we transformed immortalized human mammary epithelial cells, MCF10A, with or without BRCA1/p53 gene-specific knockdowns. Stable knockdown of BRCA1 alone in MCF10A cells led to centrosome amplification, impaired p53 protein stability, increased sensitivity towards DNA-damaging agents, defective chromosomal condensation at mitosis and elevated protein levels of cyclin D1 and c-myc. While over-expression of mutant H-Ras transformed MCF10A cells, depletion of BRCA1 dramatically enhanced the in vivo tumorigenesis that was associated with higher levels of VEGF, enhanced vascularization and less apoptosis in the BRCA1-deficient Ras-transformed tumors. The Ras-transformed BRCA1-deficient tumors exhibited features of the epithelial-to-mesenchymal transition, appeared to secrete matrix metalloproteases as visualized by in vivo bio-imaging of tumors using fluorescent probe MMP680, and were locally metastatic to lymph nodes. Our results suggest that loss of BRCA1 function may contribute to the aggressiveness of Ras-MAPK driven human breast cancer with associated increase in levels of cyclin D1 and c-myc, enhanced MAPK activity, angiogenic potential &   invasive  ness. This mammary xenograft tumor model may be useful as a tool to understand human breast tumor angiogenesis and metastasis, as well as to test candidate therapeutics. 
 Predictive factors for BRCA1/BRCA2 mutations in women with ductal carcinoma in situ. It is unclear whether women with ductal carcinoma in situ (DCIS), like their counterparts with   invasive   breast cancer, warrant genetic risk assessment and testing on the basis of high-risk variables. The authors of this report identified predictive factors for mutations in the breast cancer-susceptibility genes BRCA1 and BRCA2 in women who were diagnosed with DCIS.One hundred eighteen women with DCIS who were referred for genetic counseling and underwent genetic testing for BRCA1/BRCA2 mutations between 2003 and 2010 were included in the study. Logistic regression models were fit to determine the associations between potential predictive factors and BRCA status.Of 118 high-risk women with DCIS, 27% (n = 32) tested positive for BRCA1/BRCA2 mutations. Of those, 10% (n = 12) and 17% (n = 20) had BRCA1 and BRCA2 mutations, respectively. Age, race, and tumor characteristics did not differ between BRCA noncarriers and carriers. In a multivariate logistic model, ≥2 relatives with ovarian cancer (OC) (odds ratio [OR], 8.81; 95% confidence interval [CI], 1.38-56.29; P = .034), and a score ≥10% according to the BRCAPRO mathematical model for calculating the probability that a particular family member carries a germline BRCA mutation (OR, 6.37; 95% CI, 2.23-18.22; P = .0005) remained as independent significant predictors for a BRCA mutation. Fifty-seven percent of mutation carriers but only 25% of noncarriers underwent prophylactic mastectomy(P = .0037). This difference remained significant for patients aged ≤40 years (P = .025).Women who had DCIS and a family history of OC or who had BRCAPRO scores ≥10% had a high rate of BRCA positivity regardless of age at diagnosis. These findings suggest that high-risk patients with DCIS are appropriate candidates for genetic testing for BRCA mutations in the presence of predictive factors even if they do n  ot have   invasive breast cancer. 
 Is mammographic breast density a breast cancer risk factor in women with BRCA mutations? Increased mammographic breast density is well recognized as a breast cancer risk factor in the general population. However, it is unclear whether it is a risk factor in women with BRCA mutations. We present the results of a nested case-control screening study investigating the relationship between breast density and breast cancer incidence in this population.Women ages 25 to 65 years with known BRCA mutations were enrolled onto a single-center, high-risk breast cancer screening program. Using a computer-aided technique (Cumulus), quantitative percentage density (PD) was measured for each participant on her baseline mammogram by a single, blinded observer.Between November 1997 and March 2008, 462 women (mean age, 44 years; 245 BRCA1 and 217 BRCA2) were screened and 50 breast cancers were diagnosed (38   invasive  , 12 ductal carcinoma in situ [DCIS]). Density was not measured in 40 women of whom four developed cancer (three   invasive  , one DCIS). Mean PD (+/- standard deviation [SD]) for 376 women who did not develop breast cancer was 34% (23) compared with 31% (21) for 46 women with cancer (P = .51). Logistic regression model of breast cancer incidence and PD revealed an odds ratio of 0.99 (+/- 0.01 SD) for a one-unit increase in PD (P = .44). Results remained nonsignificant in multivariate analysis, as well as when women with pure DCIS were excluded.Increased mammographic breast density is not associated with higher breast cancer incidence in women with BRCA mutations. On the basis of these findings, density should not be considered a factor for these women in decision making regarding prophylactic surgery or chemoprevention. 
 Spectrum and frequencies of BRCA1/2 mutations in Bulgarian high risk breast cancer patients. About 3885 women are diagnosed with breast cancer and 1285 die from the disease each year in Bulgaria. However no genetic testing to identify the mutations in high-risk families has been provided so far.We evaluated 200 Bulgarian women with primary   invasive   breast cancer and with personal/ family history of breast cancer for the presence of unequivocally damaging germline mutations in BRCA1/2 using Sanger sequencing.Of the 200 patients, 39 (19.5 %) carried a disease predisposing mutation, including 28 (14 %) with a BRCA1 mutation and 11 (5.5 %) with a BRCA2 mutation. At BRCA1, 6 different mutations were identified, including 2 frameshifts, 1 nonsense and 1 missense that had been previously reported (c.5030_5033delCTAA, c.5263_5264insC, c.4603G > T, c.181 T > G), and 2 frameshifts, which were novel to this study (c.464delA, c.5397_5403delCCCTTGG). At BRCA2, 7 different frameshift mutations were identified, including 5 previously reported (5851_5854delAGTT, c.5946delT, c.5718_5719delCT, c.7910_7914delCCTTT,c.9098_9099insA) and 2 novel (c.8532_8533delAA, c.9682delA). A BRCA1 mutation was found in 18.4 % of women diagnosed with breast cancer at/or under the age of 40 compared to 11.2 % of women diagnosed at a later age; a BRCA2 mutation was found in 4 % of women diagnosed at/or under the age of 40 compared to 6.5 % of women diagnosed at a later age. A mutation was present in 26.8 % patients with a positive family history and in 14.4 % of women with a negative family history. The most prevalent mutation observed in 22 patients (11 %) was BRCA1 c.5263_5264insC, a known Slavic mutation with founder effect in Eastern European and AJ communities. Other recurrent mutations were BRCA2 c.9098-9099insA (2 %), BRCA1 c.181T > G (1 %) and BRCA2 c.5851_5854delAGTT (1 %). Notably, BRCA1 c.5263_5264insC represented 56 % of all mutations identified in this series. Of the 22 patients with BRCA1 c.5263_5264insC, 9 were diagnosed with early onset breast cancer, 11 with TNBCs, 4 with bilateral breast cancer, and 6 with both breast and ovarian cancer.This is the first comprehensive study of the BRCA1/2 mutation spectrum in Bulgaria and will assist the establishment of efficient protocols for genetic testing and individualized risk assessment for Bulgarian breast/ovarian cancer patients and healthy individuals at a high-risk. 
 BRCA1 promoter methylation is associated with increased mortality among women with breast cancer. Promoter-CpG island hypermethylation has been proposed as an alternative mechanism to inactivate BRCA1 in the breast where somatic mutations of BRCA1 are rare. To better understand breast cancer etiology and progression, we explored the association between BRCA1 promoter methylation status and prognostic factors as well as survival among women with breast cancer. Promoter methylation of BRCA1 was assessed in 851 archived tumor tissues collected from a population-based study of women diagnosed with   invasive   or in situ breast cancer in 1996-1997, and who were followed for vital status through the end of 2002. About 59% of the tumors were methylated at the promoter of BRCA1. The BRCA1 promoter methylation was more frequent in   invasive   cancers (P = 0.02) and among premenopausal cases (P = 0.05). BRCA1 promoter methylation was associated with increased risk of breast cancer-specific mortality (age-adjusted HR 1.71; 95% CI: 1.05-2.78) and all-cause mortality (age-adjusted HR 1.49; 95% CI: 1.02-2.18). Neither dietary methyl intakes in the year prior to the baseline interview nor the functional polymorphisms in one-carbon metabolism were associated with BRCA1 methylation status. Our study is the first epidemiological investigation on the prognostic value of BRCA1 promoter methylation in a large population-based cohort of breast cancer patients. Our results indicate that BRCA1 promoter methylation is an important factor to consider in predicting breast cancer survival. 
 Ductal carcinoma in situ in BRCA mutation carriers. The current literature suggests that ductal carcinoma in situ (DCIS) of the breast is infrequently diagnosed in patients with BRCA germline mutations. We studied women at high risk of hereditary breast cancer syndromes who underwent testing for BRCA1 and BRCA2 to estimate DCIS prevalence and incidence in known BRCA-positive women compared with high-risk women who were mutation negative.We analyzed breast event outcomes in a retrospective cohort of 129 BRCA-positive and 269 BRCA-negative women undergoing genetic testing for a BRCA mutation between September 1996 and December 2003 at University of California, San Francisco. We estimated the frequency of DCIS and   invasive   cancer and time to breast events from birth using a Cox proportional hazard model for competing risks. Histologic grade of DCIS was also compared between groups.Among BRCA carriers, 48 (37%) had DCIS (with or without   invasive   cancer) compared with 92 noncarriers (34%). Univariate analysis showed that both DCIS and   invasive   cancer had an earlier onset in mutation carriers than in noncarriers, although on a per-woman basis, this difference was not statistically significant. High-grade DCIS was more common in BRCA1 mutation carriers than in patients without a mutation (P = .02).DCIS is equally as prevalent in patients who carry deleterious BRCA mutations as in high familial-risk women who are noncarriers, but occurs at an earlier age. Our results argue for the consideration of DCIS as a criterion for BRCA risk assessments with appropriate weighting in prediction models such as BRCAPRO. 
 The prevalence of BRCA1 mutations among young women with triple-negative breast cancer. Molecular screening for BRCA1 and BRCA2 mutations is now an established component of risk evaluation and management of familial breast cancer. Features of hereditary breast cancer include an early age-of-onset and over-representation of the 'triple-negative' phenotype (negative for estrogen-receptor, progesterone-receptor and HER2). The decision to offer genetic testing to a breast cancer patient is usually based on her family history, but in the absence of a family history of cancer, some women may qualify for testing based on the age-of-onset and/or the pathologic features of the breast cancer.We studied 54 women who were diagnosed with high-grade, triple-negative   invasive   breast cancer at or before age 40. These women were selected for study because they had little or no family history of breast or ovarian cancer and they did not qualify for genetic testing using conventional family history criteria. BRCA1 screening was performed using a combination of fluorescent multiplexed-PCR analysis, BRCA1 exon-13 6 kb duplication screening, the protein truncation test (PTT) and fluorescent multiplexed denaturing gradient gel electrophoresis (DGGE). All coding exons of BRCA1 were screened. The two large exons of BRCA2 were also screened using PTT. All mutations were confirmed with direct sequencing.Five deleterious BRCA1 mutations and one deleterious BRCA2 mutation were identified in the 54 patients with early-onset, triple-negative breast cancer (11%).Women with early-onset triple-negative breast cancer are candidates for genetic testing for BRCA1, even in the absence of a family history of breast or ovarian cancer. 
 Mammography screening and the risk of breast cancer in BRCA1 and BRCA2 mutation carriers: a prospective study. Women with a genetic predisposition to breast cancer may be at increased risk of cancer after exposure to ionizing radiation. It is unclear whether mammography screening increases the risk of breast cancer among BRCA1 and BRCA2 carriers. We identified 2,346 women with a BRCA1 (n = 1844) or BRCA2 (n = 502) mutation and no breast cancer, and we reviewed their history of mammography exposure. These women were followed for an average of 5.3 years and were observed for new breast cancer diagnoses. At study entry, 1808 women (77.1 %) reported ever having had a mammogram; of these, 204 women (11.2 %) reported having had a mammogram before age 30. We estimated the hazard ratios for the developm  ent of i  nvasive breast cancer, conditional on the number of prior mammograms and on the age at first mammogram. Hazard ratios were estimated and stratified by gene (BRCA1 or BRCA2), relative to women with no exposure. We observed no significant association between prior mammography exposure and breast cancer risk for BRCA1 carriers (HR 0.79; 95 % CI 0.53-1.19; P = 0.26) or for BRCA2 carriers (HR 0.90; 95 % CI 0.35-2.34; P = 0.83). An early age at first mammogram (<30 years) did not increase breast cancer risk among BRCA1 carriers (HR 0.75; 95 % CI 0.41-1.37; P = 0.35) or among BRCA2 carriers (HR 0.69; 95 % CI 0.19-2.48; P = 0.57). Exposure to mammography in women with BRCA1 and BRCA2 mutations is not associated with an increased risk of breast cancer. 
 [Analysis of K-ras, BRCA1/2, CHEK2 mutations and microsatellite markers (loss of heterozygosity at 9p, 17p and 18q) in sporadic pancreas adenocarcinomas]. The purpose of this study was to investigate informativety and clinical significance of most frequent somatic alterations in K-ras, TP53, CDKN2A, MADH4 and more uncommon mutations in BRCA1, BRCA2, CHEK2 genes, which arise on pre  invasive   stage in sporadic pancreatic adenocarcinomas (PA), in Russian patients. We examined surgically resected and manually microdissected primary PA tissue samples and samples of normal pancreatic tissue for 37 individuals. K-ras mutations in codon 12 were found in 24 tumors (0.65) and none of normal tissue samples. No mutations were detected in BRCA1(185delAG, 300T > G, 4153delA, 4158A > G,5382insC), BRCA2 (695insT, 6174delT) and CHEK2 (1100delC) genes. Informativety for allelic loss of three tumor suppressor genes studied had not statistically significant differences: 60% - for TP53 (GDB186817) and CDKN2A (D9S974 + D9S162); and 65.7% - for MADH4 (D18S363 + D18S474) (t = 0.48). Maximal frequency of loss of heterozygosity (LOH) was observed for CDKN2A - 0.95. For TP53 and MADH4 it was 0.62 and 0.70 respectively. The tumors included 80% cases showing LOH on different chromosomal loci. The combination of K-ras mutations (c.12) and LOH at 9p, 17p and 18q resulted in a high informativety of selected molecular markers: 85.7%. Instability of microsatellites was found only in 9% of PA. 
 Reproductive factors and ovarian cancer risk in Jewish BRCA1 and BRCA2 mutation carriers (United States). To determine whether oral contraceptive (OC) use, childbearing, breastfeeding and tubal ligation differ between ovarian cancer cases with and without a BRCA1/2 mutation.A case-only study of 242 Jewish women with   invasive   epithelial ovarian cancer. Women were genotyped for three Ashkenazi founder mutations (185delAG and 5382insC in BRCA1 and 6174delT in BRCA2). We obtained data on OC use, childbearing, breastfeeding, gynecologic surgeries and other reproductive factors from each woman. We compared the frequencies of these risk factors in carriers and non-carriers using unconditional logistic-regression, controlling for other covariates.Among the 242 cases, 64 (26.4%) carried one of the BRCA1 founder mutations, and 31 (12.8%) carried the BRCA2 mutation. Although there were no differences in the percent of nulliparous women between carriers and non-carriers, parous BRCA1 carriers reported fewer live births than non-carriers (average of 2.1 versus 2.5 live births, OR = 0.61, 95% CI = 0.39-0.95, adjusted for age at diagnosis, tubal ligation and duration of OC use). Carriers and non-carriers did not differ in their history of breastfeeding, or in their lifetime use of OCs. BRCA1 carriers were more likely than non-carriers to have had a tubal ligation (25.0 versus 10.2%, OR = 3.67, 95% CI = 1.55-8.70, adjusted for age at diagnosis, number of live births and OC duration).In general, OC use, childbearing and breastfeeding do not differ between BRCA1/2 carriers and non-carriers with ovarian cancer. However, the effects of tubal ligation may differ between BRCA1 carriers and non-carriers. 
 Ovarian cancer risk in Ashkenazi Jewish carriers of BRCA1 and BRCA2 mutations. Several studies to date have reported ovarian cancer risk due to inherited BRCA1 and BRCA2 mutations using familial data or population-based series of probands. Familial aggregation associated with both of these methods may result in a substantial ascertainment bias. To address this, we have used a case-control design that does not involve familial aggregation to estimate the lifetime penetrance of ovarian cancer due to BRCA1 and BRCA2 mutations.A total of 382 ovarian cancer cases self-identified as being Jewish with no prior diagnosis of breast cancer were derived from two hospital-based series. In the first series, all 197   invasive   epithelial ovarian cancer cases self-identified as Jewish and without a prior history of breast cancer, diagnosed and treated at Memorial Sloan-Kettering Cancer Center between 1986 and 2000, were identified. In the second series, 185 Jewish   invasive   epithelial ovarian cancer patients without prior breast cancer were identified in a study conducted at 11 centers in North America and Israel from 1995 to 1996. Controls were 3434 Jewish women without any prior history of breast or ovarian cancer from a large study of genotyped volunteers of Jewish origin in the Washington, D. C. area recruited by investigators at the National Cancer Institute. The cases and controls were genotyped for three Ashkenazi Jewish founder mutations, namely 185delAG and 5382insC in BRCA1 and 6174delT in BRCA2. The lifetime penetrances were estimated using the odds ratios, mutation prevalence in the controls, and ovarian cancer incidence rates in the general American population obtained from the Surveillance, Epidemiology and End Results database adjusted for the incidence of ovarian cancer following breast cancer.Mutations were identified in 147 cases and 62 controls. The estimated penetrances at age 70 years were 37% (95% confidence interval, 25-71%) for a BRCA1 mutation and 21% (95% CI, 13-41%) for a BRCA2 mutation.The lifetime penetrances of BRCA1 mutations are lower than estimates obtained using familial data with multiple affected members but larger than estimates from some population-based proband series. The lifetime penetrance estimate of a BRCA2 mutation is in the range reported by some of the studies based on familial data. These results could have implications for clinical counseling, surgical interventions, and screening recommendations in women carrying these founder mutations. 
 Downregulation of EZH2 decreases growth of estrogen receptor-negative   invasive   breast carcinoma and requires BRCA1. Increased levels of enhancer of zeste homolog 2 (EZH2), a critical regulator of cellular memory, are associated with negative estrogen receptor (ER) expression and disease progression in breast cancer. High levels of EZH2 signal the presence of metastasis and poor outcome in breast cancer patients. To test the hypothesis that deregulation of EZH2 contributes to ER-negative breast cancer progression, EZH2 expression was inhibited in ER-negative breast cancer cells MDA-MB-231 and CAL51 using a lentivirus system. EZH2 knockdown decreased proliferation and delayed the G(2)/M cell-cycle transition, although not affecting apoptosis. In vivo, EZH2 downregulation significantly decreased breast xenograft growth and improved survival. EZH2 knockdown upregulated BRCA1 protein. Of note, BRCA1 knockdown was sufficient to rescue the effects of EZH2 downregulation on proliferation, G(2)/M arrest, and on the levels of hyperphosphorylated mitotic Cdc25C and Cyclin B1 proteins, crucial for entry into mitosis.   Invasive   ER-negative breast carcinomas show significant overexpression of EZH2 and downregulation of BRCA1 proteins. Taken together, we show that EZH2 is important in ER-negative breast cancer growth in vivo and in vitro, and that BRCA1 is required for the proliferative effects of EZH2. Blockade of EZH2 may provide a prime target to prevent and/or halt ER-negative breast cancer progression. 
 Contribution of BRCA1 and BRCA2 Germline Mutations to Early Algerian Breast Cancer. Breast cancer is the most common female malignancy and the leading cancer mortality cause among Algerian women. Germline mutations in the BRCA1 and BRCA2 genes in patients with early-onset breast cancer have not been clearly identified within the Algerian population. It is necessary to study the BRCA1/2 genes involvement in the Algerian breast cancer occurrence. We performed this study to define germline mutations in BRCA1/2 and their implication in breast cancer among young women from eastern Algeria diagnosed or treated with primary   invasive   breast cancer at the age of 40 or less who were referred to Anti-Cancer Center of Setif, Algeria. Case series were unselected for family history. Eight distinct pathogenic mutations were identified in eight unrelated families. Three deleterious mutations and one large genomic rearrangement involving deletion of exon 2 were found in BRCA1 gene. In addition, four mutations within the BRCA2 gene and one large genomic rearrangement were identified. Novel mutation was found among Algerian population. Moreover, five variants of uncertain clinical significance and favor polymorphisms were identified. Our data suggest that BRCA1/2 mutations are responsible for a significant proportion of breast cancer in Algerian young women. 
 Impact of Age at Primary Breast Cancer on Contralateral Breast Cancer Risk in BRCA1/2 Mutation Carriers. To determine prospectively overall and age-specific estimates of contralateral breast cancer (CBC) risk for young patients with breast cancer with or without BRCA1/2 mutations.A cohort of 6,294 patients with   invasive   breast cancer diagnosed under 50 years of age and treated between 1970 and 2003 in 10 Dutch centers was tested for the most prevalent BRCA1/2 mutations. We report absolute risks and hazard ratios within the cohort from competing risk analyses.After a median follow-up of 12.5 years, 578 CBCs were observed in our study population. CBC risk for BRCA1 and BRCA2 mutation carriers was two to three times higher than for noncarriers (hazard ratios, 3.31 [95% CI, 2.41 to 4.55; P < .001] and 2.17 [95% CI,1.22 to 3.85; P = .01], respectively). Ten-year cumulative CBC risks were 21.1% (95% CI, 15.4 to 27.4) for BRCA1, 10.8% (95% CI, 4.7 to 19.6) for BRCA2 mutation carriers and 5.1% (95% CI, 4.5 to 5.7) for noncarriers. Age at diagnosis of the first breast cancer was a significant predictor of CBC risk in BRCA1/2 mutation carriers only; those diagnosed before age 41 years had a 10-year cumulative CBC risk of 23.9% (BRCA1: 25.5%; BRCA2: 17.2%) compared with 12.6% (BRCA1: 15.6%; BRCA2: 7.2%) for those 41 to 49 years of age (P = .02); our review of published studies showed ranges of 24% to 31% before age 40 years (BRCA1: 24% to 32%; BRCA2:17% to 29%) and 8% to 21% after 40 years (BRCA1: 11% to 52%; BRCA2: 7% to 18%), respectively.Age at first breast cancer is a strong risk factor for cumulative CBC risk in BRCA1/2 mutation carriers. Considering the available evidence, age-specific risk estimates should be included in counseling. 
 Suppression of breast cancer invasion and migration by indole-3-carbinol: associated with up-regulation of BRCA1 and E-cadherin/catenin complexes. Indole-3-carbinol (I3C) is a compound occurring naturally in cruciferous vegetables and has been indicated as a promising agent in preventing breast cancer development and progression. In the present study we have investigated the effect of I3C on the cell migration and invasion behavior in estrogen receptor positive MCF-7 and estrogen receptor negative MDA-MB-468 human breast cancer cell lines. Both MCF-7 and MDA-MB-468 were poorly   invasive   cell lines and exhibited modest invasion and migration capacity in the presence of fibronectin as the chemoattractant. I3C (50 or 100 microM) elicited a significant inhibition of in vitro cell adhesion, migration, and invasion as well as in vivo lung metastasis formation in both cell lines. I3C also suppressed the 17beta-estradiol stimulated migration and invasion in estrogen-responsive MCF-7 cells. These results indicate that anti-invasion and antimigration activities of I3C occur via estrogen-independent and estrogen-dependent pathways. Moreover, I3C significantly caused a dose-dependent increase in E-cadherin, three major catenins (alpha, beta, and gamma-catenin) and BRCA1 expression. Our current finding is the first demonstration that I3C can activate the function of invasion suppressor molecules associated with the suppression of invasion and migration in breast cancer cells. Thus, clinical application of I3C may contribute to the potential benefit for suppression of breast cancer invasion and metastasis. 
 Tubal ligation and risk of ovarian cancer in carriers of BRCA1 or BRCA2 mutations: a case-control study. In several case-control and prospective studies, tubal ligation has been associated with a decreased risk of   invasive   epithelial ovarian cancer. We aimed to assess the potential of tubal ligation in reducing the risk of ovarian cancer in women who carry predisposing mutations in the BRCA1 or BRCA2 genes.We did a matched case-control study among women from Canada, the USA, and the UK who had undergone genetic testing and who carried a pathogenic mutation in BRCA1 or BRCA2. Cases were 232 women with a history of   invasive   ovarian cancer, and controls were 232 women without ovarian cancer, and who had both ovaries intact. Cases and controls were matched for year of birth, country of residence, and mutation (BRCA1 or BRCA2). The odds ratio for developing ovarian cancer was estimated for tubal ligation, adjusting for oral contraceptive use, parity, history of breast cancer, and ethnic group.In an unadjusted analysis among BRCA1 carriers, significantly fewer cases than controls had ever had tubal ligation (30 of 173 [18%] vs 60 of 173 [35%], odds ratio 0.37 [95% CI 0.21-0.63]; p=0.0003). After adjustment for oral contraceptive use, parity, history of breast cancer and ethnic group, the odds ratio was 0.39 (p=0.002). Combination of tubal ligation and past use of an oral contraceptive was associated with an odds ratio of 0.28 (0.15-0.52). No protective effect of tubal ligation was seen among carriers of the BRCA2 mutation.Tubal ligation is a feasible option to reduce the risk of ovarian cancer in women with BRCA1 mutations who have completed childbearing. 
 Basal-like breast cancer displays distinct patterns of promoter methylation. Recent microarray profiling studies on breast cancer have identified distinct subtypes that are associated with different clinical outcomes. Promoter hypermethylation of several known or putative tumor suppressor genes occurs frequently during the pathogenesis of breast cancer. We proposed that immunohistopathologic subtypes of breast cancer are likely to contain distinct promoter methylation patterns. A panel of 10 gene promoters was assessed by quantitative multiplex methylation-specific PCR in 114   invasive   ductal carcinomas from Korea representing the three major subtypes [57 luminal, 24 human epidermal growth factor 2 (HER2), and 33 basal-like] based on immunohistochemical findings of estrogen receptor, progesterone receptor, HER2, cytokeratin 5/6 and epidermal growth factor receptor. The median methylation levels of HIN1, RASSF1A and TWIST, and the average methylation ratio were significantly lower in basal-like subtype compared to luminal or HER2 subtypes. In contrast, BRCA1 methylation level was significantly higher in basal-like subtype than in luminal subtype. The methylation status of a panel of four genes (APC1, CDH, BRCA1 and RAR-β) in luminal and HER2 subtypes were dissimilar, where HER2 tumors showed a significantly higher level of methylation compared to luminal tumors. These results suggest that gene methylation in breast cancer can potentially serve as epigenetic biomarkers and may contribute further to current breast cancer classification. 
 The molecular significance of methylated BRCA1 promoter in white blood cells of cancer-free females. BRCA1 promoter methylation has been detected in DNA from peripheral blood cells of both breast cancer patients and cancer-free females. However, the pathological significance of this epigenetic change in white blood cells (WBC) remains an open question. In this study, we hypothesized that if constitutional BRCA1 methylation reflects an elevated risk for developing breast cancer (BC), WBC that harbor methylated BRCA1 in both cancer-free females and BC patients should exhibit similar molecular changes.BRCA1 promoter methylation was examined by methylation-specific PCR in WBC from 155 breast cancer patients and 143 cancer-free females. The Human Breast Cancer EpiTect Methyl II Signature PCR Array and The Human Breast Cancer RT2 Profiler™ PCR Array were used to study the methylation status and the expression profile of several breast cancer-related genes, respectively. In addition, we used label-free MS-based technique to study protein expression in plasma.We have shown that 14.2% of BC patients and 9.1% of cancer-free females (carriers) harbored methylated BRCA1 promoter in their WBC. Interestingly, 66.7% of patients harbored methylated BRCA1 promoter in both WBC and tumors. Importantly, we have shown the presence of epigenetic changes in 9 other BC-related genes in WBC of both patients and carriers. Additionally, BRCA1 and 15 other important cancer -related genes were found to be differentially expressed in WBC from patients and carriers as compared to controls. Furthermore, we have shown that the carriers exhibited a unique plasma protein pattern different from those of BC patients and controls, with 10 proteins similarly differentially expressed in patients and carriers as compared to controls.The present results suggest the presence of a strong link between aberrant methylation of the BRCA1 promoter in WBC and breast cancer -related molecular changes, which indicate the potential predisposition of the carriers for developing breast cancer. This informs the potential use of the aberrant methylation of BRCA1 promoter in WBC as a powerful no  n-invasi  ve molecular marker for detecting predisposed individuals at a very early age. 
 The effect of symptom duration in epithelial ovarian cancer on prognostic factors. To assess the association between duration of symptoms and main prognostic factors of   invasive   epithelial ovarian cancer (EOC).The data of all histologically confirmed EOC patients diagnosed in Israel during the period 1994-1999 (n = 1,005) were retrieved from discharge summaries and admission records. Of the 371 (36.9%) patients with known presenting symptoms, the durations of 187 (50.4%) were recorded.The most common presenting symptoms were abdominal pain (65.2%). The percentage of patients with three or more symptoms increased significantly with stage (P = 0.001). No statistically significant association between duration of symptoms and prognostic factors was found.Our findings did not show an association between duration of symptoms and prognostic factors in EOC patients and may indicate that prognosis is not a function of delay in diagnosis. 
 BRCA1 and BRCA2 mutations account for a large proportion of ovarian carcinoma cases. It is believed that BRCA1 and BRCA2 germline mutations account for the majority of hereditary ovarian carcinomas; however, to the authors' knowledge, there are scant data on the prevalence and spectrum of mutations, genotype/phenotype correlations, tumor histology, and family history characteristics. To address this gap, the authors conducted a population-based study of 232 incident epithelial ovarian carcinomas in the Tampa Bay area.Genetic testing for the BRCA1 and BRCA2 genes was performed through full sequencing and BRCA1 rearrangement testing.Of 209 women with   invasive   ovarian carcinoma, 32 women (15.3%) had mutations in BRCA1 or BRCA2, including 20 BRCA1 mutations and 12 BRCA2 mutations. Of the BRCA2 mutations, 58% were outside the "ovarian cancer cluster region" (OCCR). Variants of uncertain significance were detected in 8.2% of women with   invasive   ovarian carcinoma. No mutations were identified in women with borderline or   invasive   mucinous tumors. Among the BRCA mutation-positive women, 63% had serous tumors. A family history of breast and/or ovarian carcinoma was reported in 65%, 75%, and 43.5% of relatives of BRCA1 carriers, BRCA2 carriers, and non-BRCA1/BRCA2 carriers, respectively.The data from this study suggested that 1) previous studies may have underestimated the frequency of BRCA1 and BRCA2 mutations in ovarian carcinomas, especially outside the OCCR; 2) it may be reasonable to offer genetic counseling to any woman with an   invasive  , nonmucinous epithelial ovarian tumor; and 3) among patients with   invasive   ovarian carcinoma, family history is not sufficiently accurate to predict mutation status. 
 Fertility treatments and   invasive   epithelial ovarian cancer risk in Jewish Israeli BRCA1 or BRCA2 mutation carriers. To determine whether BRCA mutation carriers who undergo fertility treatments are at increased risk of developing   invasive   epithelial ovarian cancer (IEOC).Historical cohort study.Tertiary university-affiliated medical center and the National Cancer Registry.A total of 1,073 Jewish Israeli BRCA mutation carriers diagnosed in a single institution between 1995 and 2013, including 164 carriers (15.2%) who had fertility treatments that included clomiphene citrate (n = 82), gonadotropin (n = 69), in vitro fertilization (IVF) (n = 66), or a combination (n = 50), and 909 carriers not treated for infertility.None.Odds ratios (OR) and 95% confidence intervals (CI) for IEOC association with fertility treatments and other hormone and reproductive variables.In 175 (16.3%) mutation carriers, IEOC was diagnosed; 139 women carried BRCA1, 33 carried BRCA2, and 3 had unknown mutations. Fertility treatments were not associated with IEOC risk (age-adjusted OR 0.63; 95% CI, 0.38-1.05) regardless of treatment type (with clomiphene citrate, OR 0.87; 95% CI, 0.46-1.63; with gonadotropin, OR 0.59; 95% CI, 0.26-1.31; with IVF, OR 1.08, 95% CI, 0.57-2.06). Multivariate analysis indicated an increased risk of IEOC with hormone-replacement therapy (OR 2.22; 95% CI, 1.33-3.69) and a reduced risk with oral contraceptives (OR 0.19; 95% CI, 0.13-0.28) in both BRCA1 and BRCA2 mutation carriers. Parity was a risk factor for IEOC by univariate but not multivariate analysis.According to our results, treatments for infertile BRCA mutation carriers should not be contraindicated or viewed as risk modifiers for IEOC. Parity as a risk factor in BRCA mutation carriers warrants further investigation. 
 Lipid and Metabolite Deregulation in the Breast Tissue of Women Carrying BRCA1 and BRCA2 Genetic Mutations. To use localized correlated spectroscopy (COSY) to assess for an altered biochemical state or states in breast tissue of women with BRCA gene mutations that potentially constitute pre  invasive   conditions.Institutional review board approval was obtained. Participants provided written informed consent. In vivo localized COSY images were recorded at 3 T in the breast tissue of women carrying BRCA1 (n = 9) or BRCA2 (n = 14) gene mutations and were compared with images in healthy control subjects with no family history of breast cancer (n = 10). All participants underwent contrast material-enhanced MR imaging and ultrasonography (US). Statistical significance was calculated with the Mann-Whitney two-sided nonparametric test.No abnormality was recorded with MR imaging or US. Metabolite levels in the BRCA1 cohort were reduced by 79% (P = .014) when compared with triglycerides level, and there was a 19% increase in lipid unsaturation and triglyceride levels (P = .027 and P = .086, respectively) when compared with cellular cholesterol level. Cholesterol level was reduced by 47% (P = .027) when compared with diallylic lipid level. Metabolite levels in the BRCA2 cohort showed increased unsaturation of 21% (P = .030) relative to triglycerides level. Comparison of the BRCA1 and BRCA2 cohorts showed a 47% (P = .002) increase in cholesterol level in the BRCA2 cohort when compared with diallylic lipid level and a 52% (P = .003) increase when compared with triglycerides level. Levels of diallylic lipid, unsaturated lipid, triglycerides, and terminal methyl on the acyl chain are reduced by 46% (P = .002), 57% (P = .003), 66% (P = .003), and 29% (P = .010), respectively, when compared with cholesterol level.Localized COSY recorded significant changes in women with BRCA1 and BRCA2 gene mutations when compared with control subjects. If these changes are ultimately proven to be a premalignant stage, this method may prove useful in screening. 
 Loss of heterozygosity at BRCA1 locus is significantly associated with aggressiveness and poor prognosis in breast cancer. BRCA1 and BRCA2 are two major tumor suppressor genes for hereditary breast and ovarian cancer. In sporadic breast cancer, although somatic mutations of these genes are rare, loss of heterozygosity (LOH) at BRCA1 and BRCA2 loci is common.LOH at BRCA1 and BRCA2 loci were investigated in 202 Japanese   invasive   breast cancer patients. The relationships between LOH at these gene loci and clinicopathologic characteristics were analyzed.Among 166 informative cases for both BRCA1 and BRCA2 loci, 69 (41.6%) and 52 (31.3%) tumors revealed LOH at BRCA1 and BRCA2 loci, respectively. LOH at BRCA1 LOH or BRCA2 locus was associated with higher nuclear grade (P < 0.0001, P = 0.0187). LOH at BRCA1 locus was associated with estrogen receptor and progesterone receptor negativity (P = 0.001 and P = 0.015) and significantly shorter disease-free survival (P < 0.0001), distant metastasis-free survival (P < 0.0001), and overall survival (P < 0.0001). In contrast, LOH at BRCA2 locus had no associations with estrogen receptor or progesterone receptor status and prognosis. LOH at BRCA1 locus was independently associated with poor prognosis in terms of disease-free survival (hazard ratio 3.08, 95% confidence interval [CI] 1.58-6.18, P = 0.0009), distant metastasis-free survival (hazard ratio 5.18, 95% CI 2.35-12.19, P < 0.0001), and overall survival (hazard ratio 4.97, 95% CI 1.84-15.1, P = 0.0013).LOH at BRCA1 locus could be an independent prognostic biomarker useful in identifying a subgroup of patients with poor prognosis. 
 Frequency of BRCA mutations in primary peritoneal carcinoma in Israeli Jewish women. The aim of the present study was to compare demographic and clinical characteristics of primary peritoneal carcinoma (PPC) to ovarian carcinoma (OvC) with regard to BRCA mutation frequencies.Incident cases of histologically confirmed cancer of the ovary or peritoneum diagnosed in Israeli Jewish women between March 1, 1994, and June 30, 1999, were identified within the framework of an ongoing nationwide epidemiological study of these neoplasms in Israel. The present study comprises 609 (81.5% of 747) Jewish women with epithelial stage III-IV OvC and 68 (77.3% of 88) Jewish women with PPC who were genetically tested for the BRCA mutations. Data from each patient were collected by the aid of a prestructured questionnaire and medical records. Blood samples or tumor tissue was tested for the 185delAG and 5382insC mutations in BRCA1 and the 6174delT mutations in BRCA2.A carrier rate of 28% of any BRCA 1/2 mutation was observed among the PPC group and of 30% among the   invasive   stage III-IV OvC. No differences were found between PPC and OvC neither in the overall distribution of BRCA1/2 mutation carrier rates nor according to type of mutation, age, ethnic origin, and histologic subtype. Among women with a positive family history, a higher rate of mutation carriers was observed in the PPC group compared to the OvC group (72.7 vs 43.8%, respectively, P = 0.07).The similar frequency distribution of BRCA1/2 mutations in PPC and OvC observed in the present study indicates that these mutations may predispose to PPC as well and that this neoplasm is part of the hereditary breast-ovarian cancer syndrome. 
 miRNA expression patterns in normal breast tissue and   invasive   breast cancers of BRCA1 and BRCA2 germ-line mutation carriers. miRNA deregulation has been found to promote carcinogenesis. Little is known about miRNA deregulation in hereditary breast tumors as no miRNA expression profiling studies have been performed in normal breast tissue of BRCA1 and BRCA2 mutation carriers. miRNA profiles of 17 BRCA1- and 9 BRCA2-associated breast carcinomas were analyzed using microarrays. Normal breast tissues from BRCA1 and BRCA2 mutation carriers (both n = 5) and non-mutation carriers (n = 10) were also included. Candidate miRNAs were validated by qRT-PCR. Breast carcinomas showed extensive miRNA alteration compared to normal breast tissues in BRCA1 and BRCA2 mutation carriers. Moreover, normal breast tissue from BRCA1 mutation carriers already showed miRNA alterations compared to non-mutation carriers. Chromosomal distribution analysis showed several hotspots containing down- or up-regulated miRNAs. Pathway analysis yielded many similarities between the BRCA1 and BRCA2 axes with miRNAs involved in cell cycle regulation, proliferation and apoptosis. Lesser known pathways were also affected, including cellular movement and protein trafficking. This study provides a comprehensive insight into the potential role of miRNA deregulation in BRCA1/2-associated breast carcinogenesis. The observed extensive miRNA deregulation is likely the result of genome-wide effects of chromosomal instability caused by impaired BRCA1 or BRCA2 function. This study's results also suggest the existence of common pathways driving breast carcinogenesis in both BRCA1 and BRCA2 germ-line mutation carriers. 
 Adjuvant radiotherapy for primary breast cancer in BRCA1 and BRCA2 mutation carriers and risk of contralateral breast cancer with special attention to patients irradiated at younger age. The purpose of this study was to estimate the influence of adjuvant radiotherapy for primary breast cancer (BC) on the risk of contralateral BC (CBC) in BRCA1 or BRCA2 (BRCA1/2) mutation carriers, with special attention to patients irradiated at age younger than 40 years. Additionally, tendencies in locoregional treatments and rates of contralateral risk-reducing mastectomy over time were explored. In this retrospective cohort study, 691 BRCA1/2-associated BC patients treated between 1980 and 2013 were followed from diagnosis until CBC or censoring event including ipsilateral BC recurrence, distant metastasis, contralateral risk-reducing mastectomy, other   invasive   cancer diagnosis, death, or loss to follow up. Hazard ratios (HR) for CBC associated with radiotherapy were estimated using Cox regression. Median follow-up time was 8.6 years [range 0.3–34.3 years]. No association between radiotherapy for primary BC and risk of CBC was found, neither in the total population (HR 0.82, 95 % CI 0.45–1.49) nor in the subgroup of patients younger than 40 years at primary diagnosis (HR 1.36, 95 % CI 0.60–3.09). During follow-up, the number of patients at risk decreased substantially since a large proportion of patients were censored after contralateral risk-reducing mastectomy or BC recurrence. Over the years, increasing preference for mastectomy without radiotherapy compared to breast-conserving surgery with radiotherapy was found ranging from less than 30 % in 1995 to almost 50 % after 2010. The rate of contralateral risk-reducing mastectomy increased over the years from less than 40 % in 1995 to more than 60 % after 2010. In this cohort of BRCA1/2-associated BC patients, no association between radiotherapy for primary BC and risk of CBC was observed in the total group, nor in the patients irradiated before the age of 40 years. The number of patients at risk after 10 and 15 years of follow-up, however, was too small to definitively exclude harmful effects of adjuvant radiotherapy. 
 Oral contraceptive use and risk of early-onset breast cancer in carriers and noncarriers of BRCA1 and BRCA2 mutations. Recent oral contraceptive use has been associated with a small increase in breast cancer risk and a substantial decrease in ovarian cancer risk. The effects on risks for women with germ line mutations in BRCA1 or BRCA2 are unclear.Subjects were population-based samples of Caucasian women that comprised 1,156 incident cases of   invasive   breast cancer diagnosed before age 40 (including 47 BRCA1 and 36 BRCA2 mutation carriers) and 815 controls from the San Francisco Bay area, California, Ontario, Canada, and Melbourne and Sydney, Australia. Relative risks by carrier status were estimated using unconditional logistic regression, comparing oral contraceptive use in case groups defined by mutation status with that in controls.After adjustment for potential confounders, oral contraceptive use for at least 12 months was associated with decreased breast cancer risk for BRCA1 mutation carriers [odds ratio (OR), 0.22; 95% confidence interval (CI), 0.10-0.49; P < 0.001], but not for BRCA2 mutation carriers (OR, 1.02; 95% CI, 0.34-3.09) or noncarriers (OR, 0.93; 95% CI, 0.69-1.24). First use during or before 1975 was associated with increased risk for noncarriers (OR, 1.52 per year of use before 1976; 95% CI, 1.22-1.91; P < 0.001).There was no evidence that use of current low-dose oral contraceptive formulations increases risk of early-onset breast cancer for mutation carriers, and there may be a reduced risk for BRCA1 mutation carriers. Because current formulations of oral contraceptives may reduce, or at least not exacerbate, ovarian cancer risk for mutation carriers, they should not be contraindicated for a woman with a germ line mutation in BRCA1 or BRCA2. 
 Total skin-sparing mastectomy in BRCA mutation carriers. Total skin-sparing mastectomy (TSSM) with preservation of the nipple-areolar complex skin has become increasingly accepted as an oncologically safe procedure. Oncologic outcomes after TSSM in BRCA mutation carriers have not been well-studied.We identified 53 BRCA-positive patients who underwent bilateral TSSM for prophylactic (26 patients) or therapeutic indications (27 patients) from 2001 to 2011. Cases were age-matched (for prophylactic cases) or age- and stage-matched (for therapeutic cases) with non-BRCA-positive patients. Outcomes included tumor involvement of resected nipple tissue, the development of new breast cancers in patients who underwent risk-reducing TSSM, and local-regional recurrence in patients who underwent therapeutic TSSM.Outcomes from 212 TSSM procedures in 53 cases and 53 controls were analyzed. In patients undergoing TSSM for prophylactic indications, in situ cancer was found in one (1.9 %) nipple specimen in BRCA-positive patients versus two specimens (3.8 %) in the non-BRCA-positive cohort (p = 1). At a mean follow-up of 51 months, no new cancers developed in either cohort. In patients undergoing TSSM for therapeutic indications, in situ or   invasive   cancer was found in zero of the nipple specimens in BRCA-positive patients versus two specimens (3.7 %) in the non-BRCA-positive cohort (p = 0.49). At a mean follow-up of 37 months, there were no local-regional recurrences in the BRCA-positive cohort and 1 (3.7 %) in the non-BRCA-positive cohort.TSSM is an oncologically safe procedure in BRCA-positive patients. In patients undergoing TSSM as a risk-reducing strategy, 4-year follow-up demonstrates no increased risk of developing new breast cancers; longer-term follow-up is ongoing. 
 Prophylactic and therapeutic mastectomy in BRCA mutation carriers: can the nipple be preserved? Use of nipple-sparing mastectomy (NSM) is increasing. We sought to look at the role of NSM in BRCA mutation carriers.Tissue from women with a BRCA1 or BRCA2 mutation who underwent mastectomy between March 1987 and June 2009 at a single institution was reviewed. The entire nipple-areolar complex (NAC) was excised and histologically evaluated. The presence of terminal duct lobular units (TDLUs) and premalignant or malignant lesions in the NAC was noted.Sixty-two NACs from 33 women (25 BRCA1, 8 BRCA2) were studied. TDLUs were present in 15 (24%) NAC specimens. No evidence of atypical hyperplasia, carcinoma in situ, or   invasive   carcinoma was found in any of the 33 prophylactic mastectomy specimens. Among the 29 breasts with cancer and available tissue, 2 (7%) had malignant findings and 1 (3%) had atypia in the NAC. One woman who underwent bilateral mastectomy for bilateral   invasive   carcinoma had one nipple with tumor within lymphatics, and her contralateral nipple had atypical lobular hyperplasia. A second woman had ductal carcinoma in situ involving a single major lactiferous duct.The probability of nipple involvement by premalignant or malignant lesions in the NAC of BRCA mutation carriers is low at time of prophylactic mastectomy, but higher (10%) in women undergoing therapeutic mastectomy. NSM may be appropriate and oncologically safe for selected women with BRCA mutations. However, 24% of NACs contained TDLUs, with only 8% found in the nipple papilla; the significance of this for long-term risk is unknown. 
 [BRCA1 selectively regulates the expression of progesterone receptor A in sporadic   invasive   ductal breast carcinoma]. To investigate the expression level of BRCA1, progesterone receptor A (PRA) and B (PRB) in tissue of sporadic   invasive   ductal breast carcinoma and further statistically analyze the BRCA1 effects on the expression rates of PRA and PRB.Sixty-eight cases of adenosis of breast and sporadic   invasive   ductal breast carcinoma were selected. The corresponding paraffin-embodied tissues were collected from the archive of Department of Pathology, Nanfang Hospital. The expressions level of BRCA1, PRA and PRB were detected by immunohistochemistry. Wilcoxon two-sample test was used to analyze the differential expression of BRCA1 between adenosis and sporadic   invasive   ductal breast carcinoma. Chi-square test was used to analyze the effects of BRCA1 on the PRA or PRB expression rate. P value < 0.05 was considered statistically significant.(1) In   invasive   sporadic ductal breast carcinoma, the positive rate of BRCA1 expression of 60.29% (41/68) was lower than the positive rate of BRCA1 expression at 85.30% (58/68) in adenosis of breast. And the difference of BRCA1 expression between two groups was statistically significant (P < 0.01); (2) In   invasive   sporadic ductal breast carcinoma, the positive rate of PRA expression for negative BRCA1 expression samples was 81.48% (22/27) and it was higher than the positive rate of PRA expression at 53.66% (22/47) for positive BRCA1 expression samples. And the difference of PRA expression rates between two groups was statistically significant (P < 0.05). It indicated that the expression of BRCA1 affected the expression rate of PRA; In   invasive   sporadic ductal breast carcinoma, the PRB expression rates between positive and negative BRCA1 expression samples were not statistically significant (P > 0.05). It indicated that BRCA1 had no effect upon the expression rate of PRB.In sporadic breast carcinoma, a negative expression of BRCA1 is selectively associated with a higher expression rate of PRA rather than PRB. Thus BRCA1 selectively regulates the expression of PRA in sporadic breast carcinoma. 
 BRCA1-associated breast cancers present differently from BRCA2-associated and familial cases: long-term follow-up of the Dutch MRISC Screening Study. The Dutch MRI Screening Study on early detection of hereditary breast cancer started in 1999. We evaluated the long-term results including separate analyses of BRCA1 and BRCA2 mutation carriers and first results on survival.Women with higher than 15% cumulative lifetime risk (CLTR) of breast cancer were screened with biannual clinical breast examination and annual mammography and magnetic resonance imaging (MRI). Participants were divided into subgroups: carriers of a gene mutation (50% to 85% CLTR) and two familial groups with high (30% to 50% CLTR) or moderate risk (15% to 30% CLTR).Our update contains 2,157 eligible women including 599 mutation carriers (median follow-up of 4.9 years from entry) with 97 primary breast cancers detected (median follow-up of 5.0 years from diagnosis). MRI sensitivity was superior to that of mammography for   invasive   cancer (77.4% v 35.5%; P<.00005), but not for ductal carcinoma in situ. Results in the BRCA1 group were worse compared to the BRCA2, the high-, and the moderate-risk groups, respectively, for mammography sensitivity (25.0% v 61.5%, 45.5%, 46.7%), tumor size at diagnosis≤1 cm (21.4% v 61.5%, 40.9%, 63.6%), proportion of DCIS (6.5% v 18.8%, 14.8%, 31.3%) and interval cancers (32.3% v 6.3%, 3.7%, 6.3%), and age at diagnosis younger than 30 years (9.7% v 0%). Cumulative distant metastasis-free and overall survival at 6 years in all 42 BRCA1/2 mutation carriers wit  h invasi  ve breast cancer were 83.9% (95% CI, 64.1% to 93.3%) and 92.7% (95% CI, 79.0% to 97.6%), respectively, and 100% in the familial groups (n=43).Screening results were somewhat worse in BRCA1 mutation carriers, but 6-year survival was high in all risk groups. 
 Effect of BRCA1/2 mutations on long-term survival of patients with   invasive   ovarian cancer: the national Israeli study of ovarian cancer. To evaluate the long-term survival of ovarian cancer (OvC) patients in total and by BRCA1/2 mutation status.In a nationwide case-control study on OvC conducted in Israel between 1994 and 1999, 779 Jewish women with epithelial   invasive   OvC were tested for the three Ashkenazi Jewish founder mutations in BRCA1 (185delAG; 5382insC) and BRCA2 (6174delT) genes and followed for survival up to 2003. Of the 605 women of Ashkenazi origin, 213 (35.2%) carried a mutation in the BRCA1/2 genes. Clinical characteristics were abstracted from the patients' medical records. The Kaplan-Meier method, log-rank tests, and stepwise Cox regression model were used for survival analyses.The 5-year survival rate for the entire group was 39%. Median survival for carriers was significantly longer than for noncarriers (53.7 v 37.9 months, respectively; P = .002). This differential survival was pronounced among women diagnosed at stages III to IV (5-year survival rates of 38.1% and 24.5% for carriers and noncarriers, respectively; P < .001) and for women with poor grade (45.4% v 31.5%, for carriers and noncarriers, respectively; P < .001). These results remained significant after controlling for age at diagnosis, grade, and morphology. This benefit in prognosis was seen for both BRCA1 and BRCA2 carriers compared with noncarriers. During the study period (median follow-up, 6.2 years), being a BRCA1/2 mutation carrier decreased the mortality rate by 28%.This study confirms that, among Ashkenazi OvC patients, BRCA1/2 mutations are associated with improved long-term survival. This may be due to distinct clinical behavior and/or to a better response to chemotherapy. 
 BRCA1 interacts with Smad3 and regulates Smad3-mediated TGF-beta signaling during oxidative stress responses. BRCA1 is a key regulatory protein participating in cell cycle checkpoint and DNA damage repair networks. BRCA1 plays important roles in protecting numerous cellular processes in response to cell damaging signals. Transforming growth factor-beta (TGF-beta) is a potent regulator of growth, apoptosis and   invasive  ness of tumor cells. TFG-beta activates Smad signaling via its two cell surface receptors, the TbetaRII and ALK5/TbetaRI, leading to Smad-mediated transcriptional regulation.Here, we report an important role of BRCA1 in modulating TGF-beta signaling during oxidative stress responses. Wild-type (WT) BRCA1, but not mutated BRCA1 failed to activate TGF-beta mediated transactivation of the TGF-beta responsive reporter, p3TP-Lux. Further, WT-BRCA1, but not mutated BRCA1 increased the expression of Smad3 protein in a dose-dependent manner, while silencing of WT-BRCA1 by siRNA decreased Smad3 and Smad4 interaction induced by TGF-beta in MCF-7 breast cancer cells. BRCA1 interacted with Smad3 upon TGF-beta1 stimulation in MCF-7 cells and this interaction was mediated via the domain of 298-436aa of BRCA1 and Smad3 domain of 207-426aa. In addition, H(2)O(2) increased the colocalization and the interaction of Smad3 with WT-BRCA1. Interestingly, TGF-beta1 induced Smad3 and Smad4 interaction was increased in the presence of H(2)O(2) in cells expressing WT-BRCA1, while the TGF-beta1 induced interaction between Smad3 and Smad4 was decreased upon H(2)O(2) treatment in a dose-dependent manner in HCC1937 breast cancer cells, deficient for endogenous BRCA1. This interaction between Smad3 and Smad4 was increased in reconstituted HCC1937 cells expressing WT-BRCA1 (HCC1937/BRCA1). Further, loss of BRCA1 resulted in H(2)O(2) induced nuclear export of phosphor-Smad3 protein to the cytoplasm, resulting decreased of Smad3 and Smad4 interaction induced by TGF-beta and in significant decrease in Smad3 and Smad4 transcriptional activities.These results strongly suggest that loss or reduction of BRCA1 alters TGF-beta growth inhibiting activity via Smad3 during oxidative stress responses. 
 Prevalence of BRCA1 mutation carriers among U.S. non-Hispanic Whites. Data from several countries indicate that 1% to 2% of Ashkenazi Jews carry a pathogenic ancestral mutation of the tumor suppressor gene BRCA1. However, the prevalence of BRCA1 mutations among non-Ashkenazi Whites is uncertain. We estimated mutation carrier prevalence in U.S. non-Hispanic Whites, specific for Ashkenazi status, using data from two population-based series of San Francisco Bay Area patients with   invasive   cancers of the breast or ovary, and data on breast and ovarian cancer risks in Ashkenazi and non-Ashkenazi carriers. Assuming that 90% of the BRCA1 mutations were detected, we estimate a carrier prevalence of 0.24% (95% confidence interval, 0.15-0.39%) in non-Ashkenazi Whites, and 1.2% (95% confidence interval, 0.5-2.6%) in Ashkenazim. When combined with U.S. White census counts, these prevalence estimates suggest that approximately 550,513 U.S. Whites (506,206 non-Ashkenazim and 44,307 Ashkenazim) carry germ line BRCA1 mutations. These estimates may be useful in guiding resource allocation for genetic testing and genetic counseling and in planning preventive interventions. 
 Chemotherapy-induced amenorrhea in patients with breast cancer with a BRCA1 or BRCA2 mutation. To determine the likelihood of long-term amenorrhea after treatment with chemotherapy in women with breast cancer who carry a BRCA1 or BRCA2 mutation.We conducted a multicenter survey of 1,954 young women with a BRCA1 or BRCA2 mutation who were treated for breast cancer. We included premenopausal women who were diagnosed with   invasive   breast cancer between 26 and 47 years of age. We determined the age of onset of amenorrhea after breast cancer for women who were and were not treated with chemotherapy, alone or with tamoxifen. We considered chemotherapy-induced amenorrhea to have occurred when the patient experienced ≥ 2 years of amenorrhea, commencing within 2 years of initiating chemotherapy, with no resumption of menses.Of the 1,426 women who received chemotherapy, 35% experienced long-term amenorrhea. Of the 528 women who did not receive chemotherapy, 5.3% developed long-term amenorrhea. The probabilities of chemotherapy-induced amenorrhea were 7.2% for women diagnosed before age 30 years, 33% for women age 31 to 44 years, and 79% for women diagnosed after age 45 years (P trend < .001). The probability of induced amenorrhea was higher for women who received tamoxifen than for those who did not (52% v 29%; P < .001).Age at treatment and use of tamoxifen are important predictors of chemotherapy-induced amenorrhea in women who carry a BRCA1 or BRCA2 mutation. The risk of induced long-term amenorrhea does not seem to be greater among mutation carriers than among women who do not carry a mutation. 
 Prevalence of BRCA mutations and founder effect in high-risk Hispanic families. Approximately 12% of the U.S. population is Hispanic, with the majority residing in urban centers such as Los Angeles. The prevalence of BRCA mutations among high-risk Hispanic families is unknown.One hundred and ten unrelated probands of Hispanic origin, with a personal or family history of breast and/or ovarian cancer, presented for genetic cancer risk assessment, were enrolled in an Institutional Review Board-approved registry and underwent BRCA testing. Haplotype analyses were done if BRCA mutations were observed in two or more unrelated probands.Mean age at diagnosis was 37 years (range = 23-59) for the 89 (81%) probands with   invasive   breast cancer. Overall, 34 (30.9%) had deleterious mutations (25 in BRCA1, 9 in BRCA2), 25 (22.7%) had one or more unclassified variants, and 51 (46.4%) had negative results. The mean pretest mutation probability using the Couch model, Myriad model, and BRCAPro was 19.6% (range = 4-77%). The combined average mutation probability was 32.8% for carriers, 15.5% for noncarriers, and 12.9% for variant carriers (P < 0.0001). The most common deleterious mutation was 185delAG (4 of 34, 11.8%). The Hispanic 185delAG carrier families share the same haplotype from D17s1320 through BRCA1, as do two reference Ashkenazi Jewish families. Haplotype analyses of additional recurrent BRCA1 mutations [IVS5+1G>A (n=2),S955X (n = 3), R1443X (n = 3), and 2552delC (n = 2)] also suggest founder effects, with four of six mutations seen almost exclusively in families with Latin American/Caribbean or Spanish ancestry.This is the largest study to date of high-risk Hispanic families in the United States. Six recurrent mutations accounted for 47% (16 of 34) of the deleterious mutations in this cohort. The BRCA1185delAG mutation was prevalent (3.6%) in this clinic-based cohort of predominantly Mexican descent, and shared the Ashkenazi Jewish founder haplotype. 
 Surveillance of BRCA1 and BRCA2 mutation carriers with magnetic resonance imaging, ultrasound, mammography, and clinical breast examination. Current recommendations for women who have a BRCA1 or BRCA2 mutation are to undergo breast surveillance from age 25 years onward with mammography annually and clinical breast examination (CBE) every 6 months; however, many tumors are detected at a relatively advanced stage. Magnetic resonance imaging (MRI) and ultrasound may improve the ability to detect breast cancer at an early stage.To compare the sensitivity and specificity of 4 methods of breast cancer surveillance (mammography, ultrasound, MRI, and CBE) in women with hereditary susceptibility to breast cancer due to a BRCA1 or BRCA2 mutation.A surveillance study of 236 Canadian women aged 25 to 65 years with BRCA1 or BRCA2 mutations who underwent 1 to 3 annual screening examinations, consisting of MRI, mammography, and ultrasound at a single tertiary care teaching hospital between November 3, 1997, and March 31, 2003. On the day of imaging and at 6-month intervals, CBE was performed.Sensitivity and specificity of each of the 4 surveillance modalities, and sensitivity of all 4 screening modalities vs mammography and CBE.Each imaging modality was read independently by a radiologist and scored on a 5-point Breast Imaging Reporting and Data System scale. All lesions with a score of 4 or 5 (suspicious or highly suspicious for malignancy) were biopsied. There were 22 cancers detected (16   invasive   and 6 ductal carcinoma in situ). Of these, 17 (77%) were detected by MRI vs 8 (36%) by mammography, 7 (33%) by ultrasound, and 2 (9.1%) by CBE. The sensitivity and specificity (based on biopsy rates) were 77% and 95.4% for MRI, 36% and 99.8% for mammography, 33% and 96% for ultrasound, and 9.1% and 99.3% for CBE, respectively. There was 1 interval cancer. All 4 screening modalities combined had a sensitivity of 95% vs 45% for mammography and CBE combined.In BRCA1 and BRCA2 mutation carriers, MRI is more sensitive for detecting breast cancers than mammography, ultrasound, or CBE alone. Whether surveillance regimens that include MRI will reduce mortality from breast cancer in high-risk women requires further investigation. 
 Prevalence of BRCA1 and BRCA2 mutations in women diagnosed with ductal carcinoma in situ. The distribution of BRCA1 and BRCA2 mutations in women diagnosed with non  invasive   breast carcinoma is unknown.To estimate the BRCA1 and BRCA2 mutation prevalence in women with ductal carcinoma in situ (DCIS), unselected for age, family history, or ethnicity.The data were 369 DCIS cases diagnosed among female residents aged 20 to 79 years from the state of Connecticut between September 15, 1994, and March 14, 1998. These women were participants in a large population-based case-control study of breast carcinoma in situ. Telephone interviews were used to collect risk factor information and blood or buccal specimens were collected for BRCA1 and BRCA2 mutation testing.Prevalence of disease-associated mutations of BRCA1 and BRCA2 in women diagnosed with DCIS.Three (0.8%) and 9 (2.4%) of 369 DCIS cases had disease-associated mutations in BRCA1 or BRCA2, respectively. One woman had a mutation in both genes (BRCA1 W321X and BRCA2 3398del5). Carriers were significantly more likely than noncarriers to report a first-degree (mother, sister, or daughter) family history of breast cancer (odds ratio [OR], 3.7; 95% confidence interval [CI], 1.1-12.4), as well as a personal history of ovarian cancer. In addition, carriers were more likely than noncarriers to be diagnosed at an early age (<50 years) (OR, 3.4; 95% CI, 1.0-11.7), as well as to report at least 1 first-degree relative diagnosed with breast cancer before 50 years (OR, 10.6; 95% CI, 3.0-37.0).Ductal carcinoma in situ is a part of the breast/ovarian cancer syndromes defined by BRCA1 and BRCA2, with mutation rates similar to those found for   invasive   breast cancer. These findings suggest that patients with breast cancer with an appropriate personal or family history of breast and/or ovarian cancer should be screened and followed according to high-risk protocols, regardless of whether they are diagnosed with in situ or   invasive   breast cancer. 
 Preventing future cancers by testing women with ovarian cancer for BRCA mutations. Women with ovarian cancer have a 10% probability of carrying a BRCA mutation. If a mutation is identified, unaffected family members can undergo genetic testing and cancer risk-reducing strategies. We estimated the net health benefits and cost-effectiveness of different criteria for BRCA mutation testing in women with ovarian cancer, and the downstream benefits for their first-degree relatives (FDRs).We developed a Markov Monte Carlo simulation model to compare four criteria for BRCA testing in women with ovarian cancer: no testing (reference); only if personal history of breast cancer, family history of breast/ovarian cancer, or Ashkenazi Jewish ancestry; only if   invasive   serous cancer; any   invasive   nonmucinous epithelial cancer. Net health benefit was life expectancy for FDRs and primary outcome was the incremental cost-effectiveness ratio (ICER). The model estimated the number of future breast and ovarian cancer cases in FDRs.BRCA testing based on personal/family history and ancestry could prevent future cases in FDRs with an ICER of $32,018 per year of life (LY) gained compared with the reference strategy. BRCA testing based on serous or any nonmucinous epithelial ovarian cancer could prevent more cancer cases, but at ICERs of $128,465 and $148,363 per LY gained, respectively.BRCA testing of women with ovarian cancer based on personal/family history of cancer or Ashkenazi Jewish ancestry is a cost-effective strategy to prevent future breast and ovarian cancers among FDRs. More inclusive testing strategies prevent additional cancer cases but at significant cost. 
 Early onset breast cancer in a registry-based sample of African-american women: BRCA mutation prevalence, and other personal and system-level clinical characteristics. Young Black women are disproportionately afflicted with breast cancer, a proportion of which may be due to BRCA1 and BRCA2 (BRCA) gene mutations. In a sample of Black women with early onset breast cancer, we evaluated BRCA mutations and explored personal and system-level clinical characteristics. Black women diagnosed with   invasive   breast cancer (age ≤50) were recruited through the state cancer registry. Participants completed a questionnaire, genetic counseling and BRCA testing. Of the 48 women who consented to study participation, 46 provided a usable biologic specimen for BRCA testing. The overall prevalence of BRCA mutations and variants of uncertain significance (VUS) in participants was 6.5% and 34.8%, respectively. Of these, only 14 were referred for genetic counseling prior to study enrollment. Overall, those participants who chose to undergo bilateral mastectomy had a higher number of relatives with breast and ovarian cancer (p = 0.024) and a higher household income (p = 0.009). BRCA mutation prevalence and the high prevalence of VUS in participants are consistent with prior studies. Furthermore, clinical factors such as family history and financial means may influence type of surgery recommended and chosen, at both the provider and patient level, respectively. Finally, the limited number of patients referred for genetic counseling prior to surgical treatment for breast cancer may represent a missed clinical opportunity to inform surgical decisions. 
 BRCA1/p220 loss triggers BRCA1-IRIS overexpression via mRNA stabilization in breast cancer cells. BRCA1/p220-assocaited and triple negative/basal-like (TN/BL) tumors are aggressive and incurable breast cancer diseases that share among other features the no/low BRCA1/p220 expression. Here we show that BRCA1/p220 silencing in normal human mammary epithelial (HME) cells reduces expression of two RNA-destabilizing proteins, namely AUF1 and pCBP2, both proteins bind and destabilize BRCA1-IRIS mRNA. BRCA1-IRIS overexpression in HME cells triggers expression of several TN/BL markers, e.g., cytokeratins 5 and 17, p-cadherin, EGFR and cyclin E as well as expression and activation of the pro-survival proteins; AKT and survivin. BRCA1-IRIS silencing in the TN/BL cell line, SUM149 or restoration of BRCA1/p220 expression in the mutant cell line, HCC1937 reduced expression of TN/BL markers, AKT and survivin and induced cell death. Collectively, we propose that BRCA1/p220 loss of expression or function triggers BRCA1-IRIS overexpression through a post-transcriptional mechanism, which in turn promotes formation of aggressive and   invasive   breast tumors by inducing expression of TN/BL and survival proteins. 
 Breast cancer risks in individuals testing negative for a known family mutation in BRCA1 or BRCA2. Genetic testing for BRCA1 and BRCA2 mutations in family members of individuals with known deleterious mutations can distinguish between patients at high risk of disease and those who are not. Some studies have suggested that individuals testing negative for known familial mutations (true negatives), may still have a higher risk of breast cancer (BC) than the general population. We have examined a prospectively followed cohort of true negative women in the US. Subjects were close relatives of known BRCA1 and BRCA2 mutation carriers who had undergone genetic testing, were negative for the known familial mutation, and were unaffected at the time of genetic testing. Standardized incidence ratios (SIR) and 95% confidence intervals (CI) were calculated using SEER incidence rates. Among 375 true negatives, two   invasive   and two in situ BC and no ovarian cancers were diagnosed with mean follow up of 4.9 years (total of 1,962 person years).Four   invasive   BC were expected, whereas two were observed, for an age-adjusted SIR of 0.52 (95% CI 0.13-2.09). We observed more cases of in situ BC (n = 2) than were expected (n = 0.9; SIR = 2.30; 95% CI 0.57-9.19).There were no cases of ovarian cancer observed; 0.4 case was expected. In this prospective study of women who were unaffected at the time of genetic testing and who were negative for the known familial mutation in BRCA1/2, no excess risk of   invasive   BC was observed. Our data suggest that such women in the US should adhere to population based guidelines for breast cancer screening. 
 The penetrance of ductal carcinoma in situ among BRCA1 and BRCA2 mutation carriers. Ductal carcinoma in situ (DCIS) is a precancerous lesion of the female breast and is strongly suspected to be a precursor of   invasive   breast cancer (IBC). Our goal is the estimation of the age-specific and lifetime penetrances of DCIS among carriers of either a BRCA1 or BRCA2 deleterious mutation. We jointly re-analyze the SEER9 database and a previous study by Claus et al. (JAMA 293:964-969, 2005). Estimation is performed via Bayes theorem after the evaluation of the ratio of age-specific DCIS incidences, and extrapolation to the general population of the study-specific penetrance obtained from Claus et al. From the SEER9 database, we estimate the lifetime risk of DCIS to be 0.98 %, in contrast to value of 12.5 % usually reported for IBC. By extending the result in Claus et al. to the general population, we obtain a lifetime risk for carriers of a deleterious mutation of either BRCA1 or BRCA2 of 6.21 % (95 % CI 6.09-6.33 %). The increase in lifetime risk of DCIS for a BRCA mutation carrier compared to a non-carrier is therefore about six-fold. Our quantification is directly relevant to the identification and genetic counseling of BRCA mutation carriers, and emphasizes the potential importance of including information on diagnoses of DCIS in counseling of individuals who are at familial risk for breast cancer. All these factors can contribute to a more specific and targeted prevention, potentially reducing the impact of IBC among BRCA mutation carriers. 
 Reproductive risk factors for ovarian cancer in carriers of BRCA1 or BRCA2 mutations: a case-control study. Several of the known risk factors for ovarian cancer are thought to act through their effects on ovulation and the menstrual cycle, such as parity, breastfeeding, and use of oral contraceptives. We aimed to assess the effect of these three risk factors, and of tubal ligation, on the risk of ovarian cancer in women who carry a mutation in the BRCA1 or BRCA2 genes.We did a matched case-control study in women who were found to carry a pathogenetic mutation in BRCA1 or BRCA2. Participants were derived from a population-based study of ovarian cancer in Ontario, Canada, and from an international registry of mutation carriers based in Toronto, ON, Canada. All participants completed a written questionnaire that detailed their reproductive history. Women with   invasive   ovarian cancer and controls were matched on year of birth, country of residence, mutation (BRCA1 or BRCA2), and history of breast cancer. The odds ratios and 95% CI for ovarian cancer were estimated with respect to use of oral contraceptives, parity, breastfeeding, and tubal ligation.Questionnaires were completed by 799 women with a history of   invasive   ovarian cancer (670 with BRCA1 mutations, 128 with BRCA2 mutations, and one with a mutation in both genes), and controls were 2424 women without ovarian cancer (2043 with BRCA1 mutations, 380 with BRCA2 mutations, and one with a mutation in both genes). Use of oral contraceptives reduced the risk of ovarian cancer in carriers of BRCA1 mutations (odds ratio 0.56 [95% CI 0.45-0.71]; p<0.0001) and carriers of BRCA2 mutations (0.39 [0.23-0.66]; p=0.0004). Parity was associated with a reduced risk for carriers of BRCA1 mutations (0.67 [0.46-0.96]; p=0.03), but with an increased risk for those with BRCA2 mutations (2.74 [1.18-6.41]; p=0.02). Breastfeeding was associated with a reduced risk for carriers of BRCA1 mutations (0.74 [0.56-0.97]; p=0.03). An effect of similar magnitude was seen for carriers of BRCA2 mutations (0.72 [0.41-1.29]; p=0.27), but this was not statistically significant. The association with tubal ligation was not significant for carriers of BRCA1 mutations (0.80 [0.59-1.08]; p=0.15), or for carriers of BRCA2 mutations (0.63 [0.34-1.15]; p=0.13).Oral contraceptives could be used as a means to prevent ovarian cancer in carriers of BRCA1 and BRCA2 mutations. The possible adverse effect of parity on ovarian-cancer risk in women with a BRCA2 mutation needs further study. 
 Estrogen receptor status in BRCA1- and BRCA2-related breast cancer: the influence of age, grade, and histological type. BRCA1-related breast cancers are more frequently estrogen receptor (ER) negative than are either BRCA2-related or nonhereditary breast cancers. The relationship between ER status and other clinical features of hereditary breast cancers has not been well studied.ER status, grade, and histological tumor type were evaluated in 1131 women with   invasive   breast cancer, ascertained at 10 centers in North America. There were 208 BRCA1 mutation carriers, 88 BRCA2 carriers, and 804 women without a known mutation. We stratified the patients by mutation status, grade, age, and histological type and calculated the percentage of ER-positive tumors within each stratum.BRCA1 mutation carriers were more likely to have ER-negative breast cancers than were women in other groups, after adjustment for age, grade, and histological subtype (P < 0.001). Only 3.9% of BRCA1-related breast cancers were ER-positive cancers occurring in women in their postmenopausal years. The direction and magnitude of the change in ER status with increasing age at diagnosis in BRCA1 carriers was significantly different from in BRCA2 carriers (P(intercept) = 0.0002, P(slope) = 0.04). Notably, changes in ER status with age at diagnosis for BRCA1 carriers and noncarriers were almost identical (P(slope) = 0.98).The strong relationship between the presence of a BRCA1 mutation and the ER-negative status of the breast cancers is neither a consequence of the young age at onset nor the high grade but is an intrinsic property of BRCA1-related cancers. The ER-negative status of these cancers may reflect the cell of origin of BRCA1-related cancers. 
 Prognosis of early-onset breast cancer based on BRCA1/2 mutation status in a French population-based cohort and review. The debate concerning poorer survival for patients with breast cancer (BC) carrying a BRCA1 germline mutation is unresolved, and requires additional data from population-based studies.We followed 232 women with   invasive   BC under age 46, ascertained prospectively through a French population-based BC registry and tested for BRCA1/2 mutations (median follow-up: 82 months). We compared tumour characteristics and survival rates between 21 BRCA1/2 deleterious mutation carriers and 211 non-carriers.As compared to sporadic tumours, BRCA1/2 tumours showed higher grade (P = 0.02), fewer ductal carcinoma in situ (P = 0.02), more frequent medullary histology (P = 0.02), more frequent negative oestrogen and progesterone receptors (P = 0.001 each). At 5 years, BC-specific survival, metastasis-free survival, ipsilateral recurrence-free survival and contralateral BC-free survival rates for BRCA1/2 mutation carriers were 95.0%, 94.7%, 100% and 90.0% respectively, compared with 89.6%, 78.2%, 88.8% and 94.4% respectively, for non-carriers (not significant). Rates for women carrying only a BRCA1 mutation were 93.3%, 93.3%, 100%, 86.7%, respectively. 76% of BRCA1/2 carriers received chemotherapy.Despite unfavourable tumour features, we found no evidence for poorer short-term survival in BRCA1 mutation carriers compared to non-carriers in this prospective population-based cohort. The high rate of BRCA1 carriers who received chemotherapy for their BC should question the positive impact of this treatment, as suggested by preclinical studies showing increased chemosensitivity of BRCA1-associated tumours. 
 A high prevalence of BRCA1 mutations among breast cancer patients from the Bahamas. The Bahamas is a group of islands in the Caribbean with a high incidence of early onset breast cancer. In isolated populations, the identification of founder mutations in cancer predisposing genes may facilitate genetic testing and counseling. To date, six distinct BRCA1 mutations have been found in patients from cancer families from the Bahamas. The frequencies of these mutant alleles have not been measured in a large series of unselected breast cancer patients from Bahamas. We studied 214 Bahamian women with   invasive   breast cancer, unselected for age or family history of cancer. All patients were screened for six mutations in the BRCA1 gene that have previously been reported in cancer patients from the Bahamas. A mutation was identified in 49 of the 214 breast cancer patients (23%). The mutation frequency was particularly high in women diagnosed before age 50 (33%) in women with a first-degree relative with breast or ovarian cancer (41%) and in women with bilateral breast cancer (58%). Approximately 23% of unselected cases of breast cancer in the Bahamian population are attributable to a founder mutation in the BRCA1 gene-this is the highest reported mutation prevalence for any country studied to date. Genetic testing for these mutations is advisable for all women diagnosed with breast cancer in the Bahamas. 
 One in 10 ovarian cancer patients carry germ line BRCA1 or BRCA2 mutations: results of a prospective study in Southern Sweden. At least 10% of all ovarian cancers are estimated to have a hereditary background. Hereditary breast-ovarian cancer (HBOC) due to mutations in the BRCA genes is a major cause of hereditary ovarian cancer, although its frequency and relationship to age and family history in unselected series of ovarian cancers is not completely known. We report here the results of a full mutational screening analysis for germ line BRCA1 and BRCA2 mutations in 161 patients with   invasive   epithelial ovarian carcinomas. Age at diagnosis ranged from 22 to 82 years (mean 59 years). Deleterious (frame-shift, nonsense and missense) mutations were detected in 13/161 (8%) of the patients and affected BRCA1 in 12 cases and BRCA2 in one case. Four additional missense variants (one in BRCA1 and three in BRCA2) with a possible association with an increased risk ovarian cancer were revealed, resulting in a total frequency of BRCA gene alterations of 17/161 (11%). The 13 patients with deleterious mutations had a mean age of 57 years (range 41-76 years) and only three of these patients were below 50 years of age. A family history of at least one breast cancer and/or ovarian cancer was reported in all but 1 of the patients with BRCA mutations compared with only 24% of patients without mutations. Our findings in this prospective study confirm approximately 1 in 10 patients with ovarian cancer carry a germ line BRCA gene mutation associated with HBOC, and also indicate that a large number of these patients are over 50 years of age at diagnosis. 
 Morphological predictors of BRCA1 germline mutations in young women with breast cancer. Knowing a young woman with newly diagnosed breast cancer has a germline BRCA1 mutation informs her clinical management and that of her relatives. We sought an optimal strategy for identifying carriers using family history, breast cancer morphology and hormone receptor status data.We studied a population-based sample of 452 Australian women with   invasive   breast cancer diagnosed before age 40 years for whom we conducted extensive germline mutation testing (29 carried a BRCA1 mutation) and a systematic pathology review, and collected three-generational family history and tumour ER and PR status. Predictors of mutation status were identified using multiple logistic regression. Areas under receiver operator characteristic (ROC) curves were estimated using five-fold stratified cross-validation.The probability of being a BRCA1 mutation carrier increased with number of selected histology features even after adjusting for family history and ER and PR status (P<0.0001). From the most parsimonious multivariate model, the odds ratio for being a carrier were: 9.7 (95% confidence interval: 2.6-47.0) for trabecular growth pattern (P=0.001); 7.8 (2.7-25.7) for mitotic index over 50 mitoses per 10 high-powered field (P=0.0003); and 2.7 (1.3-5.9) for each first-degree relative with breast cancer diagnosed before age 60 years (P=0.01).The area under the ROC curve was 0.87 (0.83-0.90).Pathology review, with attention to a few specific morphological features of   invasive   breast cancers, can identify almost all BRCA1 germline mutation carriers among women with early-onset breast cancer without taking into account family history. 
 Detection of BRCA1 and BRCA2 gene mutation in Egyptian females with breast cancer and their relatives by PCR-SSCP method. Germline mutations in the BRCA1 or BRCA2 genes predispose their carriers to breast or/and ovarian cancers during their lifetime. This study was performed to identify germline mutations in BRCA1 and BRCA2 genes for the early detection of pre-symptomatic mutation carriers in Egyptian healthy females who were first-degree relatives of affected women from families with and without family history of breast cancer. Sixty-two patients (index cases) with   invasive   breast cancer belonging to sixty families and their asymptomatic female first-degree relatives (300 cases) were studied for germline mutations of BRCA1 and BRCA2 genes. Five mutations were detected in 52 families (86.7%) with inherited mutations in either BRCA1 or BRCA2. Sixty percent of these families had BRCA1 mutation and 26.7% had BRCA2 mutations. They were identified by using the combination of SSCP and heteroduplex analysis. All but one of the mutations were detected within the BRCA1 gene in addition to one mutation in the BRCA2 gene. 
 Multistep level sections to detect occult fallopian tube carcinoma in risk-reducing salpingo-oophorectomies from women with BRCA mutations: implications for defining an optimal specimen dissection protocol. Risk-reducing salpingo-oophorectomy (RRSO) significantly lowers the incidence of ovarian, tubal, peritoneal, and breast cancer in women who carry BRCA1 or BRCA2 germline mutations. A minority of RRSO specimens from these women will contain occult early-stage carcinoma. Most occult cancer is localized in the fallopian tube fimbriae and is as small as 1 mm in size. Pathologic detection is dependent on thoroughness of tissue examination. Recommended protocols to maximize tumor detection emphasize the role of thinly slicing the tubes and ovaries and embedding the entire specimen for microscopic examination. Additional multistep level sections of tubal fimbriae tissue blocks could theoretically increase detection of occult tubal carcinoma but the value of level sections has not been formally evaluated. This study tests the diagnostic utility of multistep level sections in RRSO specimens from 102 women with BRCA germline mutations. The original diagnoses were based on a single section from each block of thinly sliced (2 to 3 mm intervals) tissues of the entire RRSO specimen. Three multistep level sections were retrospectively obtained from each block containing tubal fimbriae. Clinically occult carcinoma ranging in size from 1 to 13 mm was initially detected in 11 of 102 women (5 in tubal fimbriae only, 1 in tubal isthmus only, 2 in fimbriae and ovary, and 3 in ovary only). Diagnoses in the original fimbrial slides and their level sections were concordant in all cases. All tubal cancers were detected in both the original sections and in the multistep level sections. None of the tubal carcinomas that were non  invasive   on the original slides showed   invasive   growth on additional level sections. No tubal carcinoma was identified in the level sections of any case originally classified as benign. Clinical follow-up among women with benign RRSO findings revealed that 2 women subsequently developed peritoneal carcinomatosis at 22 and 62 months postoperatively. Retrospective exhaustive multistep level sectioning of all remaining tubal and ovarian blocks from both these women confirmed the original benign diagnosis in 1 woman but in the other woman, the deepest levels of 1 ovarian block revealed a single 1-mm nodule of cancer at the base of an ovarian surface epithelial invagination. This specimen was one of the first RRSO cases in our experience and on review of the original report, this ovary was not dissected into multiple slices along its short axis but was only bivalved along its long axis. We propose that there does not seem to be any diagnostic value in automatically performing multistep deeper level sections of RRSO specimens if the tissue is sectioned appropriately and if the specimen is sliced at intervals that are no more than 3 mm thick. Guidelines for evaluation of RRSO specimens should emphasize the use of an optimal dissection protocol and the importance of thin tissue slice intervals. 
 Clinicopathological study of male breast carcinoma: 24 years of experience. Because breast cancer in men is rare, few patients are available for prospective studies. To learn more about its epidemiology, risk factors, clinical features, genetics and pathology in our country, we conducted a retrospective study of all cases seen in recent decades at our institution.We identified each case of male breast cancer in the database at the Sher-i-Kashmir Institute of Medical Sciences, Srinagar, Kashmir, India, between the years 1983 to 2007.We identified only 32 cases of male breast cancer over the 24-year period. Male breast cancer accounted for 32 (2.8%) of 1141 resected breast specimens, which included all breast lesions and 32 (4.1%) of 780 breast cancer cases. Of the 32 cases, 20 (62.5%) had various associated risk factors.   Invasive   ductal carcinoma was seen in 30 cases (93.7%). Of 20 cases that underwent molecular studies, 16 (80%) patients had estrogen receptor positivity whereas 14 (70%) had progesterone receptor positivity. Six cases (30%) overexpressed HER2 and p53. The BRCA2 mutation was observed in 4 cases (40%) while no patient presented with the BRCA1 mutation.An incidence of 4.1% for male breast cancer indicates that this disease is not as uncommon as presumed in this part of the world. Breast cancer in men seems more frequently to be hormone receptor positive and the BRCA2 mutation confers a significant risk to men. 
 Clinical outcomes of breast cancer in carriers of BRCA1 and BRCA2 mutations. Some features of breast cancer in women with a BRCA1 mutation suggest that hereditary breast cancer has a poor outcome. We conducted a national population-based study of Israeli women to determine the influence, if any, of a BRCA1 or a BRCA2 mutation on the prognosis in breast cancer.We obtained data on all incident cases of   invasive   breast cancer that were diagnosed from January 1, 1987, to December 31, 1988, and recorded in the Israel National Cancer Registry. We requested a paraffin-embedded tumor block or an unstained slide and the corresponding pathological and clinical records for all such cases. DNA extracted from the tumor specimens was analyzed for the three founder mutations in BRCA1 and BRCA2. For each subject, available pathological and oncologic records were reviewed.We were able to retrieve a pathological sample from 1794 of 2514 subjects (71%). Among those women, we obtained medical records for 1545 (86%). A BRCA1 or BRCA2 mutation was identified in 10% of the women who were of Ashkenazi Jewish ancestry. The adjusted hazard ratios for death from breast cancer were not significantly different among mutation carriers and noncarriers (hazard ratio among BRCA1 carriers, 0.76; 95% confidence interval [CI], 0.45 to 1.30; P=0.31; hazard ratio among BRCA2 carriers, 1.31; 95% CI, 0.80 to 2.15; P=0.28). Among women who were treated with chemotherapy, the hazard ratio for death among BRCA1 carriers was 0.48 (95% CI, 0.19 to 1.21; P=0.12).Breast cancer-specific rates of death among Israeli women are similar for carriers of a BRCA founder mutation and noncarriers. 
 Tubal epithelial lesions in salpingo-oophorectomy specimens of BRCA-mutation carriers and controls. A precursor lesion for ovarian carcinoma, tubal intraepithelial carcinoma (TIC), has been identified in BRCA-mutation carriers undergoing prophylactic bilateral salpingo-oophorectomy (pBSO). Other lesions were also identified in fallopian tubes, but different terminology, interpretation, and lack of knowledge of normal epithelium, have hampered to unravel their possible role in carcinogenesis. The aim of this study is to classify tubal epithelial lesions in BRCA-mutation carriers and controls to enable comparison of prevalence, area of localization, and possible malignant potential.Two hundred twenty-six BRCA1/2-mutation carriers were included; ovaries and fallopian tubes, embedded completely, were reviewed. Controls included 105 women who underwent BSO for non-malignant reasons. Tubal epithelial lesions included the following categories: hyperplasia, minor epithelial atypia, TIC, and   invasive   carcinoma.Tubal neoplasia was identified in 7.1% (  invasive   carcinoma, 0.9%; TIC, 6.2%) of BRCA-mutation carriers compared to none in controls (p=0.004, Fisher's exact test). Hyperplasia and minor epithelial atypia were identified in 41.6% BRCA-mutation carriers and compared to 58.1% in controls (p=0.005, Pearson's chi square).   Invasive   carcinoma and TIC showed preference for the fimbrial ends (p=0.027, Pearson's chi square), while hyperplasia and minor epithelial atypia displayed more variation in localization.  Invasive   tubal carcinoma and TIC were limited to BRCA-mutation carriers, whereas hyperplasia and minor epithelial atypia were commonly found in both BRCA-mutation carriers and controls. It is suggested that hyperplasia and minor atypia represent variations of normal tubal epithelium instead of premalignant lesions. Furthermore, total salpingectomy is strongly recommended as most but not all TIC occurred in the fimbriae. 
 Population-based study of the risk of second primary contralateral breast cancer associated with carrying a mutation in BRCA1 or BRCA2. Women with breast cancer diagnosed early in life comprise a substantial portion of those tested for BRCA1/BRCA2 mutations; however, little information is available on the subsequent risks of contralateral breast cancer in mutation carriers. This study assessed the risk of subsequent contralateral breast cancer associated with carrying a BRCA1 or BRCA2 mutation.In this nested case-control study, patients with contralateral breast cancer diagnosed 1 year or more after a first primary breast cancer (n = 705) and controls with unilateral breast cancer (n = 1,398) were ascertained from an underlying population-based cohort of 52,536 women diagnosed with a first   invasive   breast cancer before age 55 years. Interviews and medical record reviews were used to collect risk factor and treatment histories. All women were tested for BRCA1/BRCA2 mutations. Relative (rate ratios) and absolute (5- and 10-year cumulative) risks of developing contralateral breast cancer following a first   invasive   breast cancer were computed.Compared with noncarriers, BRCA1 and BRCA2 mutation carriers had 4.5-fold (95% CI, 2.8- to 7.1-fold) and 3.4-fold (95% CI, 2.0- to 5.8-fold) increased risks of contralateral breast cancer, respectively. The relative risk of contralateral breast cancer for BRCA1 mutation carriers increased as age of first diagnosis decreased. Age-specific cumulative risks are provided for clinical guidance.The risks of subsequent contralateral breast cancer are substantial for women who carry a BRCA1/BRCA2 mutation. These findings have important clinical relevance regarding the assessment of BRCA1/BRCA2 status in patients with breast cancer and the counseling and clinical management of patients found to carry a mutation. 
 Transcriptional down-regulation of Brca1 and E-cadherin by CtBP1 in breast cancer. Carboxyl-terminal binding protein 1 (CtBP1) is a transcriptional co-repressor with oncogenic potential. Immunohistochemistry staining using human breast cancer tissue arrays revealed that 92% of   invasive   ductal breast cancer cases have CtBP1-positive staining compared to 4% CtBP1-positive in normal breast tissue. To explore the functional impact of CtBP1 in breast cancer, we examined CtBP1's transcriptional regulation of known tumor suppressors, breast cancer susceptibility gene 1 (Brca1), and E-cadherin. We found CtBP1 was recruited to the promoter regions of Brca1 and E-cadherin genes in breast cancer cells. Concomitantly, Brca1 loss was detected in 57% and E-cadherin loss was detected in 76% of human   invasive   ductal breast cancers, and correlated with CtBP1 nuclear staining in these lesions. Importantly, siRNA knock down of CtBP1 restored Brca1 and E-cadherin expression in breast cancer cell lines, implying CtBP1 down-regulates Brca1 and E-cadherin genes in human breast cancer. This study provides evidence that although genetic loss of Brca1 and E-cadherin are infrequent in breast cancer, they are down-regulated at the transcriptional level by CtBP1 expression. Thus, CtBP1 activation could be a potential biomarker for breast cancer development. 
 Prediction of BRCA1/2 mutation status in patients with ovarian cancer from a hospital-based cohort. To describe patient, tumor, and family histories of cancer in a hospital-based cohort of patients with ovarian cancer and to identify the predictive value of these characteristics for (non)carrying a BRCA1 or BRCA2 mutation.Women diagnosed with   invasive   ovarian cancer between 1999 and 2003 in the west region of The Netherlands and unselected for age at diagnosis or cancer family history were included. Information was gathered on patient and tumor characteristics; p53; HER-2/neu, and KI-67 protein-expression; BRCA1/2 mutations; and family histories of cancer. Prediction tests were constructed using multivariate analyses.Our study included 85 women (mean age at diagnosis, 57.6 years; standard deviation, 11.0 years). Six of these women had been previously or concurrently diagnosed with another tumor. Of the ovarian cancers, 41 (48.2%) were in an early stage (FIGO I or II). Five pathogenic mutations (6.1%) and six unclassified variants (7.3%) were identified in BRCA1/2; when the total sensitivity of the mutation scanning was taken into account, it was estimated to reflect seven pathogenic mutations (8.5%) and eight unclassified variants (9.8%). Sixty-nine women (81.2%) had at least one relative with cancer. A personal history of breast cancer and a family history of breast, ovarian, or uterine/endometrioid cancer were found to predict the presence of pathogenic mutations.As the combination of a personal history of breast cancer and a family history of breast, ovarian, or uterine/endometrioid cancer had good predictive value for the presence of a pathogenic BRCA1/2 mutation, the presented prediction test is a useful instrument to identify those women eligible for DNA testing. 
 Hereditary breast cancer growth rates and its impact on screening policy. Imaging is often performed yearly for the surveillance of BRCA1/2 mutation carriers and women at high familial breast cancer risk. Growth of cancers in carriers may be faster as these tumours are predominantly high grade. Quantitative data on tumour growth rates in these 2 groups are lacking. Here, we have examined 80 high-risk women under surveillance for tumour size at diagnosis and preceding examinations at mammography and/or MRI. Tumour volume doubling time (DT) was assessed in 30 cancers in BRCA1/2 mutation carriers and 25 non-carriers. Impact of age and menopausal status were also evaluated. Mean DT of all   invasive   cancers was shorter in carriers (45 days CI: 26-73) than non-carriers (84 days CI: 58-131) (P = 0.048). Mean age at diagnosis was lower in carriers (40 years) than non-carriers (45 years) (P = 0.007). At multivariable analysis only age (P = 0.03), not risk-group (P = 0.26) nor menopause (P = 0.58) correlated significantly with DT. The mean growth rate slowed down to half in each successive 10 years-older group. In conclusion, age at detection indicated the growth rates of hereditary and familial breast cancers. It is recommended that the screening frequency should be adjusted according to a woman's age and a high-sensitive biannual test may be appropriate before the age of 40 years. 
 A review of histopathological subtypes of ovarian cancer in BRCA-related French Canadian cancer families. Pathogenic mutations in BRCA1 and BRCA2 have been reported in about 55-59% of breast and ovarian cancer (HBOC) families of French Canadian descent, where about 70% of families with more than two cases of ovarian cancer were mutation-positive. Given that specific subtypes of ovarian cancer are associated with mutation-positive families, we reviewed the features of 54 HBOC families of French Canadian descent that had histopathologically confirmed cases of   invasive   ovarian cancer where the BRCA1 and BRCA2 mutation status is known, and 27 families harbored germline mutations. The number of cases and ages of diagnosis of either breast cancers or ovarian cancers did not differ significantly in comparisons of mutation-positive and mutation-negative groups. However, the distribution of histopathological subtypes for the 79 cases of   invasive   epithelial cancer from the 54 HBOC families differed when grouped according to familial mutation status. The mutation-negative group had significantly more cases of the mucinous subtype of ovarian cancer when compared with the BRCA1 (P = 0.005) and BRCA2 (P = 0.017) mutation-positive groups. The presence of a mucinous subtype ovarian cancer in multiple young age of onset breast and/or ovarian mutation-negative HBOC cancer families warrants further investigation, as these families appear to exhibit features most consistent with BRCA1 and BRCA2 carrier status. 
 Histopathological findings and follow-up after prophylactic mastectomy and immediate breast reconstruction in 100 women from families with hereditary breast cancer. To survey the histopathological abnormalities in breasts of women who have undergone risk reducing mastectomy and to evaluate the effect of this measure on future breast cancer development.Between August 1995 and October 2006 100 consecutive women with a hereditary increased risk of breast cancer underwent prophylactic mastectomy (PM) at Malmö University Hospital. Fifty of the 100 women had no previous breast cancer. Fifty were BRCA1 or BRCA2 mutation carriers. All breast specimens have been examined histopathologically according to a prospective protocol. Follow-up data was collected from medical records and data in the Regional Cancer Registry.In the PM specimens abnormal lesions were found in 18 women (three with   invasiv  e cancers, eight in situ cancers and seven atypical hyperplasia). In previously healthy women lesions were more frequent after the age of 40 than among younger women (p=0.03). BRCA mutation carriers were more likely to present with ADH (atypical ductal hyperplasia)/ALH (atypical lobular hyperplasia) compared to the non-carriers/untested cases (p=0.01). After a median follow-up of 52 months (range 1-136 months) none of the women have developed breast cancer in the area of the prophylactically removed breast.Prevalent atypical or malignant lesions are relatively a common finding in PM specimens in asymptomatic women with hereditary increased risk of breast cancer. Such findings were significantly more common above age 40 in women without previous breast cancer. The risk of newly formed breast cancer after PM is small. The clinical importance of detecting a premalignant or pr  einvasiv  e lesion in the breast at PM is still unclear. 
 Effectiveness of breast cancer surveillance in BRCA1/2 gene mutation carriers and women with high familial risk. Women with a high breast cancer risk due to a familial predisposition may choose between preventive surgery and regular surveillance. The effectiveness of surveillance in high-risk women and especially BRCA1/2 mutation carriers is unknown. We present first results from a single large family cancer clinic.Women with breast cancer risk over 15% were examined by physical examination every 6 months and mammography every year. Detection rates and screening parameters were calculated for the total group and separately for different age and genetic risk groups.At least one examination was performed in 1,198 women: 449 moderate and 621 high-risk women and 128 BRCA1/2 mutation carriers. Within a median follow-up of 3 years, 35 breast cancers were detected (four ductal carcinoma-in-situ; 31   invasive   tumors); the average detection rate was 9.7 per 1,000. Detection rates (95% confidence interval) for moderate and high-risk women and BRCA1/2 carriers were 3.3 (1.1 to 8.6), 8.4 (5.4 to 13.2), and 33 (17 to 63) per 1,000 person-years, respectively. The ratio of observed cases versus breast cancers expected in an average-risk population of comparable age was 2.7, 7.0 and 23.7 respectively. Overall, node negativity was 65%; 34% of primary tumors were less than 10 mm; sensitivity was 74%. Results with respect to tumor stage and sensitivity were less favorable in BRCA1/2 carriers and in women under the age of 40.It is possible to identify young women at high risk for breast cancer. The number of cancers detected was significantly greater than expected in an age-matched average-risk population and related to the risk category. Overall, screening parameters were comparable to population screening data, with less favorable results in the youngest age group (< 40) and BRCA1/2 carriers. 
 Surveillance for familial breast cancer: Differences in outcome according to BRCA mutation status. Women with a family history of breast cancer are commonly offered regular clinical or mammographic surveillance from age 30. Data on the efficacy of such programmes are limited. Clinical, pathological and outcome data were recorded on all breast and ovarian cancers diagnosed within familial breast cancer surveillance programmes at collaborating centers in Norway and the UK up to the end of 2005. These have been analyzed according to the mutation status of the affected women (BRCA1+ve, BRCA2+ve or mutation-negative). Breast cancer was diagnosed in 442 patients subsequently followed for a total of 2095 years. Eighty-nine (20%) had BRCA1 mutations, 35 (8%) BRCA2 mutations and in 318 (72%) no mutation could be detected ("mut neg"). Five-year survival in BRCA1 was 73% compared to 96% in BRCA2 and 92% in mut neg (p = 0.000). Among BRCA1 mutation-carriers, 5-year survival was 67% for cases diagnosed as carcinoma in situ, 84% for node-negative   invasive   cancers and 58% for those with nodal involvement (p > 0.05). For BRCA2 mutation-carriers the corresponding figures were 100, 100 and 90% (p > 0.05), while for mut neg women they were 100, 97 and 71% (p = 0.03). Regular surveillance in women at increased familial risk of breast cancer is associated with a good outcome if they carry BRCA2 mutations or no detectable mutation. Carriers of BRCA1 mutations fare significantly worse, even when their tumors are diagnosed at an apparently early stage. The differences in outcome associated with different genetic causes of disease were associated with demonstrated differences in tumor biology. The findings demonstrate the outcome for genetically different breast cancers detected within a programme for early diagnosis and treatment, which is relevant to genetic counseling when women at risk have to chose between the options for preventing death from inherited breast cancer. 
 Effect of BRCA1 immunohistochemical localizations on prognosis of patients with sporadic breast carcinomas. Our purpose was to investigate the expression pattern of BRCA1 protein in sporadic breast carcinomas, as well as the clinicopathological and prognostic value of its subcellular localizations. Immunohistochemistry was performed on paraffin embedded tissue specimens from 111 sporadic,   invasive   breast carcinomas to detect the expression of the proteins BRCA1, ER, PR, erbB2, p53 and Ki67. BRCA1 protein was detected in the nuclei and the cytoplasm of the tumor cells. Nuclear BRCA1 immunoreactivity showed no relation with the classic clinicopathological markers and the expression of cerbB2, p53 and Ki67. Reduced expression of nuclear BRCA1 protein was found to exert an independent favorable impact on both the overall and relapse-free (RF) survival of the patients (p=0.019 and p=0.043, respectively). Cytoplasmic BRCA1 was associated with none of the classic histomorphological indices, except from the lymph node metastasis, with which its relation was found to be inverse (p=0.05), prolonging the RF survival of the patients (p=0.05). Our findings suggest that BRCA1 protein depicts different prognostic significance, according to its subcellular distribution. Nuclear detection of the protein was associated with a worse prognosis, while the cytoplasmic one was related to fewer recurrences as a result of fewer lymph node metastases. 
 Tissue-based predictors of germ-line BRCA1 mutations: implications for triaging of genetic testing. BRCA testing of patients with breast cancer considered at high risk for having a germ-line BRCA mutation usually consists of comprehensive mutational analysis of both BRCA1 and BRCA2. A more cost-effective strategy of triaging patients for analysis of a single gene could be adopted if tissue-based predictors indicated a high risk specifically for either BRCA1 or BRCA2. To identify potentially useful tissue-based predictors of BRCA mutation status in breast cancer, we evaluated multiple histopathologic features of   invasive   breast carcinoma on archival tissue sections from 196 high-risk patients who had undergone BRCA testing, and we analyzed which individual or combination of features was most associated with BRCA mutations. Of the 196 patients with   invasive   breast carcinoma, there were 44 (22%) with a deleterious BRCA1 mutation and 27 (14%) with a deleterious BRCA2 mutation. For patients with available untreated surgical resection specimens for evaluation (n=172), estrogen receptor-positive phenotype was inversely associated with the presence of a BRCA1 mutation (odds ratio, 0.243; 95% confidence interval, 0.070-0.840; P=.025), and high mitotic activity (≥25 mitotic figures per 10 high-power fields) was directly associated with the presence of a BRCA1 mutation (odds ratio, 4.222; 95% confidence interval, 1.353-13.18; P=.013). The combination of estrogen receptor-negative phenotype and high mitotic rate had high specificity (99%; 95% confidence interval, 95%-100%) but low sensitivity (43%; 95% confidence interval, 26%-61%) for identifying a deleterious BRCA1 mutation. In patients with breast cancer at high risk for carrying a BRCA mutation, those with estrogen receptor-negative phenotype and high mitotic rate could be triaged specifically for BRCA1 testing instead of initially performing mutational analysis for both BRCA1 and BRCA2. 
 Polymorphisms in BRCA1 and BRCA2 and risk of epithelial ovarian cancer. Because inherited BRCA1 or BRCA2 mutations strikingly increase ovarian cancer risk, polymorphisms in these genes could represent low penetrance susceptibility alleles. Previous studies of the BRCA2 N372H polymorphism suggested that HH homozygotes have a modestly increased risk of both breast and ovarian cancer. We have examined whether BRCA2 N372H or common amino acid-changing polymorphisms in BRCA1 predispose to ovarian cancer.A population-based, case control study of ovarian cancer was performed in North Carolina. Cases included 312 women with ovarian cancer (76%   invasive   and 24% borderline) and 401 age- and race-matched controls. Blood DNA from subjects was genotyped for BRCA2 N372H and BRCA1 Q356R and P871L.There was no association between BRCA2 N372H and risk of borderline or   invasive   epithelial ovarian cancer. The overall odds ratio (OR) for HH homozygotes was 0.8 [95% confidence interval (CI) = 0.4-1.5] and was similar in all subsets, including   invasive   serous cases. In addition, neither the BRCA1 Q356R (OR = 0.9, 95% CI 0.5-1.4) nor P871L (OR = 0.9, 95% CI 0.6-1.9) polymorphisms were associated with ovarian cancer risk. There was a significant racial difference in allele frequencies of the P871L polymorphism (P = 0.64 in Caucasians, L = 0.76 in African-Americans, P < 0.0001).In this population-based, case control study, common amino acid changing BRCA1 and 2 polymorphisms were not found to affect the risk of developing ovarian cancer. 
 BRCA1 expression in   invasive   breast carcinomas and clinicopathological correlations. BRCA1-positive tumors characterize a heterogeneous group of breast cancers with a specific range of histopathological and immuno-histochemical features. We evaluated the relationship between morphological characteristics and immunohistochemical profile of 14 BRCA1-positive breast tumors from an initial study group of 40 breast tumors. Morphological parameters of all cases were studied on Hematoxylin-Eosin-stained sections and their immunophenotypic profile was assessed using antibodies against estrogen receptors (ER), progesteron receptors (PR), c-erbB2 oncoprotein (Her2/neu), BRCA1-protein, and the proliferative rate of the tumoral cells was assessed by Ki-67-proliferative index. All patients were females with an average age of 43.71-year-old. Fourteen out of the 40 tumors were BRCA1-positive. All breast carcinomas in this study were   invasive  . The most common histological type in our study was   invasive   ductal carcinoma. The majority of the tumors were high-grade G3. The examination of the lymph node found metastasis in eight cases. We found 11 cases of triple negative (ER, PR and Her2/neu), while the rest showed positivity for all these three markers. We observed a high-celullar proliferation index in all cases. The immunohistochemical study of BRCA1-protein is important to the study of the development and progression of the disease. 
 Placental cadherin and the basal epithelial phenotype of BRCA1-related breast cancer. BRCA1-related breast cancer frequently has a basal epithelial phenotype, and P-cadherin is a basal marker. We undertook a detailed evaluation of the relationship among P-cadherin, prognostic markers in breast cancer, and outcome.This study was restricted to 292 cases of first primary   invasive   breast cancer diagnosed in Ashkenazi Jewish women between 1980 and 1995. All available blocks were stained for P-cadherin, and 261 were included in the final statistical analyses, including 27 germ line BRCA1 mutation carriers and 8 BRCA2 mutation carriers. Descriptive analyses were done followed by survival analyses and a Poisson regression analysis.P-cadherin was present in 80 of the 261 breast cancers (31%) and was more frequently present in tumors that have a basal epithelial phenotype [i.e., high-grade, estrogen receptor- and KIP1 (p27(Kip1))-negative tumors, with expression of cytokeratin 5/6, cyclin E, TP53, and presence of BRCA1 mutations and vascular nests (all P < 0.001)]. In a univariate survival model, expression of P-cadherin was associated with a relative risk (RR) of death from breast cancer at a 10-year follow-up of 2.9 (95% confidence interval, 1.8-4.7; P < 0.0001) and was a predictor of poor univariate survival in both lymph node-negative and -positive breast cancers. In a multivariate analysis, the effect of P-cadherin levels was not independent of other basal-related markers. Multivariable interaction modeling showed that P-cadherin positivity was highly predictive of a poor prognosis in small, node-negative breast cancers (RR, 7.1; P = 0.006).P-cadherin is a marker for basal-like breast cancers and is strongly associated with the presence of a BRCA1 mutation. It is an adverse prognostic factor, particularly in small, node-negative breast cancers. 
 Loss of expression of FANCD2 protein in sporadic and hereditary breast cancer. Fanconi anemia (FA) is a recessive disorder associated with progressive pancytopenia, multiple developmental defects, and marked predisposition to malignancies. FA is genetically heterogeneous, comprising at least 12 complementation groups (A-M). Activation of one of the FA proteins (FANCD2) by mono-ubiquitination is an essential step in DNA damage response. As FANCD2 interacts with BRCA1, is expressed in proliferating normal breast cells, and FANCD2 knockout mice develop breast tumors, we investigated the expression of FANCD2 in sporadic and hereditary   invasive   breast cancer patients to evaluate its possible role in breast carcinogenesis. Two tissue microarrays of 129 and 220 sporadic breast cancers and a tissue microarray containing 25 BRCA1 germline mutation-related   invasive   breast cancers were stained for FANCD2. Expression results were compared with several clinicopathological variables and tested for prognostic value. Eighteen of 96 (19%) sporadic breast cancers and two of 21 (10%) BRCA1-related breast cancers were completely FANCD2-negative, which, however, still showed proliferation. In the remaining cases, the percentage of FANCD2-expressing cells correlated strongly with mitotic index and percentage of cells positive for the proliferation markers Ki-67 and Cyclin A. In immunofluorescence double staining, coexpression of FANCD2 and Ki-67 was apparent. In survival analysis, high FANCD2 expression appeared to be prognostically unfavorable for overall survival (p = 0.03), independent from other major prognosticators (p = 0.026). In conclusion, FANCD2 expression is absent in 10-20% of sporadic and BRCA1-related breast cancers, indicating that somatic inactivating (epi)genetic events in FANCD2 may be important in both sporadic and hereditary breast carcinogenesis. FANCD2 is of independent prognostic value in sporadic breast cancer. 
 Frequent loss of BRCA1 nuclear expression in young women with breast cancer: an immunohistochemical study from an area of low incidence but early onset. Young women with breast cancer have a more unfavorable outcome and advanced disease than older women. This study was initiated to determine the difference in tumor biology between younger and older groups. One hundred fifty-five patients with   invasive   ductal carcinoma, not otherwise specified, comprised the study group, including 50 women aged less than 35 years, 50 aged 36 to 50 years, and 55 aged more than 50 years. Histopathologic parameters, including tumor size, combined histologic grade, and axillary lymph node status, were studied. Biomarkers, including estrogen receptor status, tumor proliferation rate as determined by Ki-67, and gene expressions of c-erbB-2, p53, bcl-2, and BRCA1, were determined by immunohistochemistry. Comparisons of the distribution of these parameters in three age groups were performed. Breast cancer occurring in women aged less than 35 years had a significantly higher incidence of large tumor, high proliferation rate, and loss of nuclear BRCA1 expression (44.0% versus 22.0% or 23.6%) than in the two older age groups. Breast cancer in women aged less than 35 years also had higher histologic grade and higher frequency of bcl-2-negative tumor than that found in the 36- to 50-year age group. No difference was found in lymph node status and c-erbB-2 and p53 gene expressions between the age groups. Loss of BRCA1 nuclear expression significantly correlated with higher histologic grade and high Ki-67 index (P < 0.05) in group A. These findings suggested that women aged less than 35 years have frequent loss of nuclear BRCA1 expression, which may be responsible for the specific tumor biology different from older women. However, c-erbB-2 and p53 gene expressions seem to have no important role in the adverse tumor behavior of breast cancer in young women. 
 Inherited predisposition to breast cancer among African American women. African Americans have a disproportionate burden of aggressive young-onset breast cancer. Genomic testing for inherited predisposition to breast cancer is increasingly common in clinical practice, but comprehensive mutation profiles remain unknown for most minority populations. We evaluated 289 patients who self-identified as African American with primary   invasive   breast cancer and with personal or family cancer history or tumor characteristics associated with high genetic risk for all classes of germline mutations in known breast cancer susceptibility genes using a validated targeted capture and multiplex sequencing approach. Sixty-eight damaging germline mutations were identified in 65 (22 %, 95 % CI 18-28 %) of the 289 subjects. Proportions of patients with unequivocally damaging mutations in a breast cancer gene were 26 % (47/180; 95 % confident interval [CI] 20-33 %) of those with breast cancer diagnosis before age 45; 25 % (26/103; 95 % CI 17-35 %) of those with triple-negative breast cancer (TNBC); 29 % (45/156; 95 % CI 22-37 %) of those with a first or second degree relative with breast cancer before age 60 or with ovarian cancer; and 57 % (4/7; 95 % CI 18-90 %) of those with both breast and ovarian cancer. Of patients with mutations, 80 % (52/65) carried mutations in BRCA1 and BRCA2 genes and 20 % (13/65) carried mutations in PALB2, CHEK2, BARD1, ATM, PTEN, or TP53. The mutational allelic spectrum was highly heterogeneous, with 57 different mutations in 65 patients. Of patients meeting selection criteria other than family history (i.e., with young age at diagnosis or TNBC), 48 % (64/133) had very limited information about the history of cancer in previous generations of their families. Mutations in BRCA1 and BRCA2 or another breast cancer gene occur in one in four African American breast cancer patients with early onset disease, family history of breast or ovarian cancer, or TNBC. Each of these criteria defines patients who would benefit from genomic testing and novel therapies targeting DNA repair pathways. 
 Prophylactic mastectomy in BRCA1 and BRCA2 mutation carriers: very low risk for subsequent breast cancer. To examine the outcome of prophylactic mastectomy in a hospital-based series of BRCA1/2 gene mutation carriers with and without a history of breast cancer.A center-based consecutive series of 254 BRCA1/2 gene mutation carriers that had prophylactic mastectomy after a normal surveillance round including breast-magnetic resonance imaging were identified. One hundred forty-seven asymptomatic carriers underwent bilateral mastectomy and 107 symptomatic women had contralateral mastectomy after a mean cancer free interval of 3.6 years. All removed breasts were histopathologically examined.In one asymptomatic BRCA2 carrier (0.7%) an occult small   invasive   breast cancer was diagnosed, while in 6 asymptomatic carriers (4.0% BRCA1 and 4.3% BRCA2) and in 5 symptomatic carriers (2.5% BRCA1 and 10.7% BRCA2) DCIS was detected at prophylactic mastectomy. No breast cancer occurred in the asymptomatic group after a postprophylactic follow-up period of 778 women-years. In the symptomatic carriers 1   invasive   breast cancer was detected after 580 follow-up years. From age-, cohort-, and gene-specific reference data we calculated that 15   invasive   first cancers in the asymptomatic carriers were prevented during follow-up.One   invasive   breast cancer in 147 bilateral prophylactic mastectomies (0.7%) was detected, this makes a sentinel node procedure redundant and preoperative imaging vital. The prophylactic procedure is highly effective in preventing   invasive   breast cancer in BRCA1/2 mutation carriers. Since the remaining risk is less than 0.2%/woman-year, continued surveillance of the asymptomatic carriers is not warranted. 
 Relation of contraceptive and reproductive history to ovarian cancer risk in carriers and noncarriers of BRCA1 gene mutations. In the general population, ovarian cancer risk is inversely associated with oral contraceptive use, tubal ligation, and childbearing. Among carriers of BRCA1 gene mutations, the data are conflicting. The authors identified women diagnosed with incident   invasive   epithelial ovarian cancer in the San Francisco Bay Area of California from March 1997 through July 2001. They compared the contraceptive and reproductive histories of 36 carrier cases and 381 noncarrier cases with those of 568 controls identified by random digit dialing who were frequency matched to cases on age and race/ethnicity. In both carriers and noncarriers, reduced risk was associated with ever use of oral contraceptives (odds ratio = 0.54 (95% confidence interval (CI): 0.26, 1.13) for carriers and 0.55 (95% CI: 0.41, 0.73) for noncarriers), duration of oral contraceptive use (risk reduction per year = 13% (p = 0.01) for carriers and 6% (p < 0.001) for noncarriers), history of tubal ligation (odds ratio = 0.68 (95% CI: 0.25, 1.90) for carriers and 0.65 (95% CI: 0.45, 0.95) for noncarriers), and increasing parity (risk reduction per childbirth = 16% (p = 0.26) for carriers and 24% (p < 0.001) for noncarriers). These data suggest that BRCA1 mutation carriers and noncarriers have similar risk reductions associated with oral contraceptive use, tubal ligation, and parity. 
 CDC25A, VAV1, TP73, BRCA1 and ZAP70 gene overexpression correlates with radiation response in colorectal cancer. Radiotherapy is increasingly used in adjuvant approaches for colorectal cancer (CRC) to reduce local recurrence and improve survival. However, the principal limitation is the large variability in response among different individuals due to tumor heterogeneity. In the present study, we compared gene expression profiles between radiosensitive and radioresistant colorectal cancer cell lines to identify radiation-related molecules that can be used to evaluate the effects of radiation. The CRC cell line SW620 was irradiated with a high-energy photo beam. Following radiation treatment, RNA was extracted from non-irradiated and irradiated cells, respectively, and gene expression analysis was performed by oligonucleotide microarray and the DAVID bioinformatics method. To further confirm the results, an additional 4 CRC cell lines, COLO205, T84, HCT116, SW480 and SW403 were purchased from ATCC. The radiosensitivities of each were determined by the survival fraction at 2 Gray (SF2) of the surviving cells using the ATPLite assay, and the gene expression profiles after irradiation among the radiosensitive and radioresistant cell lines were analyzed by membrane arrays. The relationships between gene expression and patient clinicopathological features were also analyzed using membrane arrays and RT-PCR. The results from oligonucleotide microarray analysis show that 1601 genes were up-regulated (gene expression ratio of post- to pre-radiation treatment>2). By bioinformatic database analysis, 30 up-regulated genes were identified as involved in DNA damage response pathways, immune response pathways and the complement and coagulation cascades pathway. Fifteen genes showed differential gene expression profiles between radiosensitive (HCT116 and SW620) and radioresistant CRC cell lines (SW403 and SW480). In 110 CRC tissues, we detected five genes CDC25A, VAV1, TP73, BRCA1 and ZAP70 from 15 overexpressed genes that significantly related to prognostic factors (tumor size, advanced stage,   invasive   depth, lymph node metastasis and differentiation). These findings suggest that CDC25A, VAV1, TP73, BRCA1 and ZAP70 may be novel markers for predicting the effectiveness of radiotherapy in CRC patients. 
 TGFbeta1/Smad3 counteracts BRCA1-dependent repair of DNA damage. Inactivation of the BRCA1 gene has been found to confer susceptibility to early-onset familial breast and ovarian cancers. BRCA1 regulates DNA repair, chromatin remodeling and affects gene transcription. Transforming growth factor-beta (TGFbeta) is a potent regulator of growth, apoptosis and   invasive  ness of tumor cells, including breast cancer cells. Here we show that Smad3 which is a component of the TGFbeta signaling pathway, forms a complex with BRCA1 in vitro and in vivo. The interaction is mediated by the MH1 domain of Smad3 and the C-terminal part of BRCA1. We observed a co-localization of Smad3 and BRCA1 in nuclear complexes. We also found that TGFbeta1/Smad3 counteracted BRCA1-dependent repair of DNA double-strand breaks in human breast epithelial cells, as evaluated by BRCA1 nuclear foci formation, single-cell gel electrophoresis and cell survival assays. Thus, TGFbeta1/Smad3 suppresses BRCA1-dependent DNA repair in response to a DNA damaging agent. 
 Lympho-vascular invasion in BRCA related breast cancer compared to sporadic controls. Germline mutations in the BRCA1 gene predispose to the development of breast cancer, exhibiting a specific histological phenotype. Identification of possible hallmarks of these tumors is important for selecting patients for genetic screening and provides inside in carcinogenetic pathways.Since BRCA1-associated breast cancers have pushing borders that prevent them from easily reaching vessels and are often of the medullary (like) type that is known to have a low rate of lympho-vascular invasion (LVI), we hypothesized that absence of LVI could characterize BRCA1 related breast cancer.A population of 68 BRCA1 related   invasive   breast cancers was evaluated for LVI by an experienced breast pathologist blinded to mutation status, and compared to a control group matched for age, grade and tumor type.LVI was present in 25.0% of BRCA1 related cases, compared to 20.6% of controls (P = 0.54, OR = 1.29, CI 0.58-2.78).LVI is frequent in BRCA1 germline mutation related breast cancers, but seems to occur as often in sporadic controls matched for age, grade and tumor type. Apparently, these hereditary cancers find their way to the blood and lymph vessels despite their well demarcation and often medullary differentiation. 
 Attitudes toward preventive oophorectomy among BRCA1 mutation carriers in Poland. Currently genetic testing for BRCA1 and BRCA2 susceptibility genes is performed throughout Europe and North America. In Poland three founder mutations in BRCA1 account for 14% of all   invasive   ovarian cancers and oophorectomy is frequently recommended to mutation carriers as a preventive measure. The purpose of the present study was to evaluate patient acceptance of the recommendation for prophylactic oophorectomy in a hereditary cancer clinic. Seventy-two women over the age of 40 and who carried a BRCA1 mutation were advised to undergo prophylactic oophorectomy. After a mean follow-up period of 19 months, 43 of the women (60%) had undergone the procedure. Of the 29 women who had not had an oophorectomy, five indicated that they did not intend to do so, 19 indicated that they intended to have the operation in the near future and five were undecided. In conclusion, preventive oophorectomy is acceptable to most Polish women at high risk of hereditary ovarian cancer and should be among the range of services offered in cancer genetics clinics. 
 Differences in natural history between breast cancers in BRCA1 and BRCA2 mutation carriers and effects of MRI screening-MRISC, MARIBS, and Canadian studies combined. It is recommended that BRCA1/2 mutation carriers undergo breast cancer screening using MRI because of their very high cancer risk and the high sensitivity of MRI in detecting   invasive   cancers. Clinical observations suggest important differences in the natural history between breast cancers due to mutations in BRCA1 and BRCA2, potentially requiring different screening guidelines.Three studies of mutation carriers using annual MRI and mammography were analyzed. Separate natural history models for BRCA1 and BRCA2 were calibrated to the results of these studies and used to predict the impact of various screening protocols on detection characteristics and mortality.BRCA1/2 mutation carriers (N = 1,275) participated in the studies and 124 cancers (99   invasive  ) were diagnosed. Cancers detected in BRCA2 mutation carriers were smaller [80% ductal carcinoma in situ (DCIS) or ≤10 mm vs. 49% for BRCA1, P < 0.001]. Below the age of 40, one   (invasi  ve) cancer of the 25 screen-detected cancers in BRCA1 mutation carriers was detected by mammography alone, compared with seven (thre  e invasi  ve) of 11 screen-detected cancers in BRCA2 (P < 0.0001). In the model, the preclinical period during which cancer is screen-detectable was 1 to 4 years for BRCA1 and 2 to 7 years for BRCA2. The model predicted breast cancer mortality reductions of 42% to 47% for mammography, 48% to 61% for MRI, and 50% to 62% for combined screening.Our studies suggest substantial mortality benefits in using MRI to screen BRCA1/2 mutation carriers aged 25 to 60 years but show important clinical differences in natural history.BRCA1 and BRCA2 mutation carriers may benefit from different screening protocols, for example, below the age of 40. 
 miR-638 mediated regulation of BRCA1 affects DNA repair and sensitivity to UV and cisplatin in triple-negative breast cancer. Triple-negative breast cancer (TNBC) represents 15 to 20% of all types of breast cancer; however, it accounts for a large number of metastatic cases and deaths, and there is still no effective treatment. The deregulation of microRNAs (miRNAs) in breast cancer has been widely reported. We previously identified that miR-638 was one of the most deregulated miRNAs in breast cancer progression. Bioinformatics analysis revealed that miR-638 directly targets BRCA1. The aim of this study was to investigate the role of miR-638 in breast cancer prognosis and treatment.Formalin-fixed, paraffin-embedded (FFPE) breast cancer samples were microdissected into normal epithelial and   invasive   ductal carcinoma (IDC) cells, and total RNA was isolated. Several breast cancer cell lines were used for the functional analysis. miR-638 target genes were identified by TARGETSCAN-VERT 6.2 and miRanda. The expression of miR-638 and its target genes was analyzed by real-time qRT-PCR and Western blotting. Dual-luciferase reporter assay was employed to confirm the specificity of miR-638 target genes. The biological function of miR-638 was analyzed by MTT chemosensitivity, matrigel invasion and host cell reactivation assays.The expression of miR-638 was decreased in IDC tissue samples compared to their adjacent normal controls. The decreased miR-638 expression was more prevalent in non-TNBC compared with TNBC cases. miR-638 expression was significantly downregulated in breast cancer cell lines compared to the immortalized MCF-10A epithelial cells. BRCA1 was predicted as one of the direct targets of miR-638, which was subsequently confirmed by dual-luciferase reporter assay. Forced expression of miR-638 resulted in a significantly reduced proliferation rate as well as decreased   invasive   ability in TNBC cells. Furthermore, miR-638 overexpression increased sensitivity to DNA-damaging agents, ultraviolet (UV) and cisplatin, but not to 5-fluorouracil (5-FU) and epirubicin exposure in TNBC cells. Host cell reactivation assays showed that miR-638 reduced DNA repair capability in post UV/cisplatin-exposed TNBC cells. The reduced proliferation,   invasive   ability, and DNA repair capabilities are associated with downregulated BRCA1 expression.Our findings suggest that miR-638 plays an important role in TNBC progression via BRCA1 deregulation. Therefore, miR-638 might serve as a potential prognostic biomarker and therapeutic target for breast cancer. 
 Prospective study of breast MRI in BRCA1 and BRCA2 mutation carriers: effect of mutation status on cancer incidence. Annual MRI screening is recommended as an adjunct to mammography for BRCA1 and BRCA2 mutation carriers. Prophylactic oophorectomy has been shown to decrease breast cancer risk in BRCA1/2 mutation carriers. Here, we aimed to examine the combined effects of MRI and oophorectomy. For this purpose, 93 BRCA1/2 mutation carriers were screened with yearly mammograms and yearly MRI scans. Study endpoints were defined as date of breast cancer diagnosis, date of prophylactic mastectomy, or date of most recent contact. Of 93 women, with a median age of 47, 80 (86%) had prophylactic oophorectomy. Fifty-one women (55%) had BRCA1 mutations. A total of 283 MRI scans were performed. Eleven breast cancers (9   invasive  , 2 ductal carcinoma in situ) were detected in 93 women (12%) with a median follow-up of 3.2 years (incidence 40 per 1,000 person-years). Six cancers were first detected on MRI, three were first detected by mammogram, and two were "interval cancers." All breast cancers occurred in BRCA1 mutation carriers (incidence 67 per 1,000 person-years). Apart from BRCA1 vs. BRCA2 mutation status, there were no other significant predictors of breast cancer incidence. Most   invasive   breast cancers were estrogen receptor negative (7 of 9) and lymph node negative (7 of 9). There have been no systemic recurrences with a median follow-up of 19 months after cancer diagnosis. Finally, it was concluded that all breast cancers occurred in BRCA1 mutation carriers, in most cases despite oophorectomy. These data suggest that surveillance and prevention strategies may have different outcomes in BRCA1 and BRCA2 mutation carriers. 
 Risk factors associated with the occurrence of breast cancer after bilateral salpingo-oophorectomy in high-risk women. Bilateral salpingo-oophorectomy (BSO) is a common procedure for preventing breast and ovarian cancer in high-risk women. The goal of this study was to determine the incidence of subsequent breast cancer (BC) in a high-risk population and to identify clinical and epidemiological predictors of BC following BSO.One hundred and thirty-three consecutive high-risk women, tested for BRCA1 and BRCA2 mutations due to family history, underwent preventive or therapeutic BSO at one of the study hospitals. One hundred and three patients had breast tissue at risk and were considered evaluable for the event-free survival analysis. Twenty-five women harbored a deleterious mutation in BRCA1 and 25 in BRCA2 genes.Fifteen cases of   invasive   BC were diagnosed with a median interval of 49 months after BSO. Multivariate analysis showed that a prior BC after 50 years of age (p=0.004), age over 50 years at the time of BSO (p=0.005), and prior replacement or contraceptive hormonal treatment (p=0.007) were significantly associated with a shorter event-free survival.In conclusion, age at prior diagnosis of cancer, age at BSO and prior hormonal treatment may be predictors of breast cancer after BSO. 
 Correlation between BRCA1 expression and apoptosis-related biological parameters in sporadic breast carcinomas. BRCA1 is a tumor suppressor gene that is responsible for hereditary breast and ovarian cancer syndrome. Increased evidence suggests that BRCA1 protein is involved in mammary carcinogenesis in sporadic and hereditary forms. Recent experimental results suggest that BRCA1 plays a role in the regulation of apoptosis. In order to test whether the analysis of human tumors would provide data supporting this hypothesis in sporadic breast carcinomas, we have investigated the relationship between BRCA1 and apoptosis-related genes. Immunohistochemical analysis was performed to determine BRCA1 and the apoptosis-related proteins bcl-2, Bax and p53 in paraffin-embedded tissues of 156 sporadic   invasive   ductal carcinomas. BRCA1 expression was positively-correlated with Bcl-2 expression (p = 0.0008), but no relationship between BRCA1 expression and Bax or p53 expression could be established. In addition, loss of BRCA1 expression was also related to poor tumor differentiation and lymph node metastasis. Our study shows that bcl-2 might be one of the target genes involved in the oncogenesis related to BRCA1. Loss of BRCA1 may contribute to tumor development in breast carcinomas, which may be independent of the p53 tumor suppressor. 
 Pathology of breast and ovarian cancers among BRCA1 and BRCA2 mutation carriers: results from the Consortium of Investigators of Modifiers of BRCA1/2 (CIMBA). Previously, small studies have found that BRCA1 and BRCA2 breast tumors differ in their pathology. Analysis of larger datasets of mutation carriers should allow further tumor characterization.We used data from 4,325 BRCA1 and 2,568 BRCA2 mutation carriers to analyze the pathology of   invasive   breast, ovarian, and contralateral breast cancers.There was strong evidence that the proportion of estrogen receptor (ER)-negative breast tumors decreased with age at diagnosis among BRCA1 (P-trend = 1.2 × 10(-5)), but increased with age at diagnosis among BRCA2, carriers (P-trend = 6.8 × 10(-6)). The proportion of triple-negative tumors decreased with age at diagnosis in BRCA1 carriers but increased with age at diagnosis of BRCA2 carriers. In both BRCA1 and BRCA2 carriers, ER-negative tumors were of higher histologic grade than ER-positive tumors (grade 3 vs. grade 1; P = 1.2 × 10(-13) for BRCA1 and P = 0.001 for BRCA2). ER and progesterone receptor (PR) expression were independently associated with mutation carrier status [ER-positive odds ratio (OR) for BRCA2 = 9.4, 95% CI: 7.0-12.6 and PR-positive OR = 1.7, 95% CI: 1.3-2.3, under joint analysis]. Lobular tumors were more likely to be BRCA2-related (OR for BRCA2 = 3.3, 95% CI: 2.4-4.4; P = 4.4 × 10(-14)), and medullary tumors BRCA1-related (OR for BRCA2 = 0.25, 95% CI: 0.18-0.35; P = 2.3 × 10(-15)). ER-status of the first breast cancer was predictive of ER-status of asynchronous contralateral breast cancer (P = 0.0004 for BRCA1; P = 0.002 for BRCA2). There were no significant differences in ovarian cancer morphology between BRCA1 and BRCA2 carriers (serous: 67%; mucinous: 1%; endometrioid: 12%; clear-cell: 2%). CONCLUSIONS/IMPACT: Pathologic characteristics of BRCA1 and BRCA2 tumors may be useful for improving risk-prediction algorithms and informing clinical strategies for screening and prophylaxis. 
 High expression of stem cell marker ALDH1 is associated with reduced BRCA1 in   invasive   breast carcinomas. Cancer stem cells (CSC) have been described in a variety of malignancies, including breast carcinomas. Among several markers, aldehyde dehydrogenase 1 (ALDH1) has been identified as reliable for breast cancer stem cells. Knockdown of BRCA1 in primary breast epithelial cells leads to an increase in cells expressing ALDH1.We examined 127 breast carcinomas for expression of ALDH1, using immunohistochemistry and correlated with clinicopathological parameters as well as the BRAC1 status.Comparing the results for both ALDH1 and BRCA1 expression showed a significant inverse association between the two, indicating that reduced BRCA1 was more often seen in breast cancer cells expressing ALDH1 (p-value = 0.044). A total of 24/110 (22%) of tumours displayed the ALDH1 + / BRCA1 -/low phenotype, which showed a trend for a relation with a high grade (p-value= 0.056). Cytoplasmic expression of ALDH1 was not correlated with tumour characteristics.Taken together, our findings suggest that increased ALDH1 is inversely correlated with decreased BRCA1 in a series of unselected breast carcinomas. Therefore, ALDH1 positive (cancer stem) cells with reduced BRCA1 phenotype may indicate a subset of patients for whom specific targeting of the CSC marker ALDH1 and more aggressive adjuvant treatment is appropriate. 
 Pathology of ovarian cancers in BRCA1 and BRCA2 carriers. Germline mutations in the BRCA1 and BRCA2 genes confer increased susceptibility to ovarian cancer. There is evidence that tumors in carriers may exhibit a distinct distribution of pathological features, but previous studies on the pathology of such tumors have been small. Our aim was to evaluate the morphologies and immunophenotypes in a large cohort of patients with familial ovarian cancer.We performed a systematic review of ovarian tumors from 178 BRCA1 mutation carriers, 29 BRCA2 mutation carriers, and 235 controls with a similar age distribution. Tumors were evaluated by four pathologists blinded to mutation status. Both morphological features and immunochemical staining for p53 and HER2 were evaluated.Tumors in BRCA1 mutation carriers were more likely than tumors in age-matched controls to be   invasive   serous adenocarcinomas (odds ratio, 1.84; 95% confidence interval, 1.21-2.79) and unlikely to be borderline or mucinous tumors. Tumors in BRCA1 carriers were of higher grade (P < 0.0001), had a higher percentage solid component (P = 0.001), and were more likely to stain strongly for p53 (P = 0.018). The distribution of pathological features in BRCA2 carriers was similar to that in BRCA1 carriers.Use of pathological features can substantially improve the targeting of predictive genetic testing. Results also suggest that BRCA1 and BRCA2 tumors are relatively aggressive and may be expected to have poor prognosis, although this may be treatment dependent. 
 Germ-line mutations in BRCA1 or BRCA2 in the normal breast are associated with altered expression of estrogen-responsive proteins and the predominance of progesterone receptor A. The breast cancer susceptibility genes BRCA1 and BRCA2 are responsible for a large proportion of familial breast and ovarian cancer, yet little is known of how disruptions in the functions of the proteins these genes encode increased cancer risk preferentially in hormone-dependent tissue. There is no information on whether a germ-line mutation in BRCA1 or BRCA2 causes disruptions in hormone-signaling pathways in the normal breast. In this study markers of hormone responsiveness were measured in prophylactically removed normal breast tissue (n = 31) in women bearing a germ-line pathogenic mutation in one of the BRCA genes. The estrogen receptor (ER) and proteins associated with ER action in hormone-sensitive tissues, namely, PS2 and the progesterone receptor (PR), were detected immunohistochemically. ER expression was not different in BRCA mutation carriers than in noncarriers, but there was a reduction in PS2 expression. PR expression was also reduced, and there was a striking lack of expression of the PRB isoform, which resulted in cases with PRA-only expression in BRCA1 and BRCA2 mutation carriers. The alterations in PS2 and PR expression were similar in the BRCA1 and BRCA2 carriers, demonstrating that although these proteins are structurally and functionally distinct, there is overlap in their interaction with hormone-signaling pathways. This study provides evidence for altered cell function arising from loss of function of one BRCA allele in the normal breast, leading to PS2 loss, preferential PRB loss, and expression of PRA alone. In breast cancer development, PRA overexpression becomes evident in premalignant lesions and is associated with features of poor prognosis in   invasive   disease and altered cell function in vitro. The results of this study suggest that heterozygosity for a germ-line mutation in BRCA1 or BRCA2 results in development of PRA predominance. This is likely to lead to changes in progesterone signaling in hormone-dependent tissues, which may be a factor in the increased risk of cancer in these tissues in women with germ-line BRCA1 or BRCA2 mutations. 
 Germline BRCA1 mutations and a basal epithelial phenotype in breast cancer. A basal epithelial phenotype is found in not more than 15% of all   invasive   breast cancers. Microarray studies have shown that this phenotype is associated with breast cancers that express neither estrogen receptor (ER) nor erbB-2 (HER2/neu) (i.e., ER/erbB-2-negative tumors). The ER/erbB-2- negative phenotype is also found in breast cancers occurring in BRCA1 mutation carriers (i.e., BRCA1-related breast cancers). We tested the hypothesis that BRCA1-related breast cancers are more likely than non-BRCA1/ 2-related breast cancer to express a basal epithelial phenotype. Among 292 breast cancer specimens previously analyzed for ER, erbB-2, p53, and germline mutations in BRCA1 and BRCA2, we identified 76 that did not overexpress ER or erbB-2. Of the 72 specimens with sufficient material for testing, 40 expressed stratified epithelial cytokeratin 5 and/or 6 (5/6). In univariate analysis, the expression of cytokeratin 5/6 was statistically significantly associated with BRCA1-related breast cancers (odds ratio = 9.0, 95% confidence interval = 1.9 to 43; P =.002, two-sided Fisher's exact test). Thus, germline BRCA1 mutations appear to be associated with a distinctive breast cancer phenotype. 
 DCIS in BRCA1 and BRCA2 mutation carriers: prevalence, phenotype, and expression of oncodrivers C-MET and HER3. Studies report conflicting evidence regarding the existence of a DCIS-associated premalignant pathway in BRCA mutation carriers. We aimed to examine the prevalence, phenotype, and expression of oncodrivers in pure DCIS (pDCIS) and   invasive   breast cancer with concurrent DCIS (IBC + DCIS) in mutation carriers.A cohort of BRCA1 and BRCA2 mutation carriers >18 years old who underwent surgery for breast cancer at an academic hospital (1992-2011) and had pathology available for review were included for study.   Invasive   breast cancer (IBC) and DCIS were stained for ER, PR, HER1, HER2, and HER3, and C-MET. DCIS prevalence was evaluated. Correlation of IBC and DCIS phenotypes was evaluated in patients with IBC + DCIS. DCIS and IBC expression of tumor markers were examined by BRCA mutation.We identified 114 breast tumors. Of all BRCA1-associated tumors, 21.1 % were pDCIS and 63.4 % were IBC + DCIS. Of all BRCA2-associated tumors, 23.3 % were pDCIS and 60.5 % were IBC + DCIS. In BRCA1 and BRCA2 mutation carriers with IBC + DCIS, there was a significant correlation in ER, PR, and HER3 expression between the DCIS and IBC components. Most BRCA1-associated DCIS did not express ER, PR or HER2, while most BRCA2-associated DCIS did express ER and PR. BRCA1- as well as BRCA2-associated DCIS had expression of HER3 and C-MET.The majority of BRCA-associated tumors had DCIS present. Concordance of DCIS and IBC phenotypes was high, arguing for the existence of a DCIS-associated premalignant pathway. Oncodrivers HER3 and C-MET were expressed in the DCIS of mutation carriers, suggesting an opportunity for prevention strategies. 
 Screening for familial ovarian cancer: poor survival of BRCA1/2 related cancers. To assess the effectiveness of annual ovarian cancer screening (transvaginal ultrasound and serum CA125 estimation) in reducing mortality from ovarian cancer in women at increased genetic risk.A cohort of 3532 women at increased risk of ovarian cancer was screened at five European centres between January 1991 and March 2007. Survival from diagnosis of ovarian cancer was calculated using Kaplan-Meier analysis and compared for proven BRCA1/2 carriers with non-carriers and whether the cancer was detected at prevalence or post-prevalent scan. Screening was performed by annual transvaginal ultrasound and serum CA125 measurement.64 epithelial ovarian malignancies (59   invasive   and 5 borderline), developed in the cohort. 26 tumours were detected at prevalent round; there were 27 incident detected cancers and 11 interval. 65% of cancers were stage 3 or 4, however, stage and survival were little different for prevalent versus post-prevalent cancers. Five year and 10 year survival in 49 BRCA1/2 mutation carriers was 58.6% (95% CI 50.9% to 66.3%) and 36% (95% CI 27% to 45%), which was significantly worse than for 15 non-BRCA carriers (91.8%, 95% CI 84% to 99.6%, both 5 and 10 year survival p = 0.015). However, when borderline tumours were excluded, the difference in survival between carriers and non-carriers was no longer significant.Annual surveillance, by transvaginal ultrasound scanning and serum CA125 measurement, in women at increased familial risk of ovarian cancer is ineffective in detecting tumours at a sufficiently early stage to influence substantially survival in BRCA1/2 carriers. 
 HIF-1α overexpression in ductal carcinoma in situ of the breast in BRCA1 and BRCA2 mutation carriers. Recent studies have revealed that BRCA1 and BRCA2 germline mutation-related breast cancers show frequent overexpression of hypoxia inducible factor-1α (HIF-1α), the key regulator of the hypoxia response. However, the question remained whether hypoxia is a late stage bystander or a true carcinogenetic event in patients with hereditary predisposition. We therefore studied HIF-1α overexpression in ductal carcinoma in situ (DCIS), an established precursor   of inva  sive breast cancer.We used immunohistochemistry to examine the expression of the hypoxia markers HIF-1α, CAIX and Glut-1 in DCIS and avail  able inv  asive carcinoma lesions of 32 BRCA1, 16 BRCA2 and 77 non-BRCA mutation-related cases. HIF-1α expression was detected in 63% of BRCA1 and 62% of BRCA2 as compared to 34% of non-BRCA mutation-related DCIS cases (p = 0.005). CAIX overexpression was present in 56% of BRCA1 and 44% of BRCA2 as compared to 6% of non-BRCA mutation-related DCIS cases (p = 0.000). Glut-1 overexpression was observed in 59% of BRCA1, 75% of BRCA2 and 67% of non-BRCA mutation-related DCIS cases (p = 0.527). Overall, HIF-1α, CAIX and Glut-1 expression in BRCA mutation-related DCIS matched the expression i  n the ac  companying invasive cancers in 60% or more of cases. In non-BRCA mutation-related cases the expression of the hypoxia markers in DCIS matched the   express  ion in the invasive part in 46% or more of the cases.Although BRCA1 and BRCA2 germlin  e mutati  on-related invasive breast cancers are different in many ways, the hypoxia-related proteins HIF-1α, CAIX and Glut-1 are express  ed in bo  th DCIS and invasive lesions of BRCA1 and BRCA2 mutation carriers. This suggests that hypoxia may already play a role in the DCIS stage of BRCA1 and BRCA2 germline mutation related breast carcinogenesis, and may also drive cancer progression. Hypoxia-related proteins are therefore putative targets for therapy and molecular imaging for early detection and monitoring therapy response in BRCA mutation patients. 
 BRCA1 mutation and young age predict fast breast cancer growth in the Dutch, United Kingdom, and Canadian magnetic resonance imaging screening trials. Magnetic resonance imaging (MRI) screening enables early detection of breast cancers in women with an inherited predisposition. Interval cancers occurred in women with a BRCA1 mutation, possibly due to fast tumor growth. We investigated the effect of a BRCA1 or BRCA2 mutation and age on the growth rate of breast cancers, as this may influence the optimal screening frequency.We reviewed the   invasive   cancers from the United Kingdom, Dutch, and Canadian MRI screening trials for women at hereditary risk, measuring tumor size at diagnosis and on preceding MRI and/or mammography. We could assess tumor volume doubling time (DT) in 100 cancers.Tumor DT was estimated for 43 women with a BRCA1 mutation, 16 women with a BRCA2 mutation, and 41 women at high risk without an identified mutation. Growth rate slowed continuously with increasing age (P = 0.004). Growth was twice as fast in BRCA1 (P = 0.003) or BRCA2 (P = 0.03) patients as in high-risk patients of the same age. The mean DT for women with BRCA1/2 mutations diagnosed at ages < or =40, 41 to 50, and >50 years was 28, 68, and 81 days, respectively, and 83, 121, and 173 days, respectively, in the high-risk group. Pathologic tumor size decreased with increasing age (P = 0.001). Median size was 15 mm for patients ages < or =40 years compared with 9 mm in older patients (P = 0.003); tumors were largest in young women with BRCA1 mutations.Tumors grow quickly in women with BRCA1 mutations and in young women. Age and risk group should be taken into account in screening protocols. 
 Clinical significance of promoter hypermethylation of DNA repair genes in tumor and serum DNA in   invasive   ductal breast carcinoma patients. The clinical relevance of frequent methylation of CpG islands of key cancer genes in breast cancer is being increasingly recognized. Our study aimed to evaluate the promoter methylation status of DNA repair genes-BRCA1MGMT and GSTP1 in tumor and circulating DNA of   invasive   ductal breast carcinoma patients.Methylation-specific PCR was carried out to investigate the promoter methylation status of genes in tumor and circulating DNA of 100 breast cancer patients in a prospective study. The effect of promoter methylation on protein expression was evaluated by immunohistochemistry.The frequency of tumor hypermethylation was 27% in BRCA1, 32% in MGMT and 25% in GSTP1 and correlated with methylation of these genes in paired serum DNA. Immunohistochemical analysis showed no detectable expression of BRCA1 and MGMT in 51/89 (57%) and 35/89 (39%) tumors, respectively. MGMT promoter methylation mediated gene silencing was associated with loss of its protein expression (p=0.002, O.R.=4.5, 95% C.I.=1.7-12.0). BRCA1 promoter methylation was not associated with loss of its protein expression, indicating that methylation is not the sole mechanism accounting for the loss/reduced BRCA1 protein expression. Importantly, GSTP1 and BRCA1 hypermethylation were found to be independent of other prognostic factors in predicting disease recurrence (p=0.02, HR=7.6, 95% C.I.=1.4-44.1; p=0.04, HR=6.2, 95% C.I.=1.1-35.7).Our study underscores the potential utility of DNA methylation of these genes in serum as a promising biomarker and can serve as a surrogate for tumor DNA methylation for diagnosis and prognosis of breast cancer. 
 Expression of the stem cell marker ALDH1 in BRCA1 related breast cancer. The BRCA1 protein makes mammary stem cells differentiate into mature luminal and myoepithelial cells. If a BRCA1 mutation results in a differentiation block, an enlarged stem cell component might be present in the benign tissue of BRCA1 mutation carriers, and these mammary stem cells could be the origin of BRCA1 related breast cancer. Since ALDH1 is a marker of both mammary stem cells and breast cancer stem cells, we compared ALDH1 expression in malignant tissue of BRCA1 mutation carriers to non-carriers.Forty-one BRCA1 related breast cancers and 41 age-matched sporadic breast cancers were immunohistochemically stained for ALDH1. Expression in epithelium and stroma was scored and compared.Epithelial (P = 0.001) and peritumoral (P = 0.001) ALDH1 expression was significantly higher in   invasive   BRCA1 related carcinomas compared to sporadic carcinomas. Intratumoral stromal ALDH1 expression was similarly high in both groups. ALDH1 tumor cell expression was an independent predictor of BRCA1 mutation status.BRCA1 related breast cancers showed significantly more frequent epithelial ALDH1 expression, indicating that these hereditary tumors have an enlarged cancer stem cell component. Besides, (peritumoral) stromal ALDH1 expression was also more frequent in BRCA1 mutation carriers. ALDH1 may therefore be a diagnostic marker and a therapeutic target of BRCA1 related breast cancer. 
 Expression of BRCA1 and BRCA2 proteins and their correlation with clinical staging in breast cancer. The purpose of this study was to evaluate the level of expression of the BRCA1 and BRCA2 proteins in sporadic breast cancer cases to determine the functional role of these genes in breast carcinogenesis.Paraffin embedded histologically proven   invasive   breast tissue sections that were obtained from 40 patients and the adjacent normal breast tissue sections used as controls to determine breast carcinoma specific changes in the expression of BRCA1 and BRCA2 by immunohistochemistry (IHC).Majority of the cases express either low or no detectable level of BRCA1 expression in tumor tissues in comparison with control; the decline in BRCA1 expression was found to be more prominent in advanced grade 3 disease. On the other hand, the expression of BRCA2 protein was moderate or low in breast cancer cases and its overall distribution did not show significant difference when compared with controls. Interestingly, those breast cancer cases, which were found to express low or no BRCA1 expression, demonstrated a higher protein level of BRCA2. The inverse correlation of BRCA1 and BRCA2 expression was more prominent in post-menopausal patients.Our results demonstrate in a subset of cases that decline in BRCA1 expression that may be associated with potentially compensatory increase in BRCA2 protein, which may depend on tumor grade as well as menopausal status. 
 Clinical characteristics of individuals with germline mutations in BRCA1 and BRCA2: analysis of 10,000 individuals. To assess the characteristics that correlate best with the presence of mutations in BRCA1 and BRCA2 in individuals tested in a clinical setting.The results of 10,000 consecutive gene sequence analyses performed to identify mutations anywhere in the BRCA1 and BRCA2 genes (7,461 analyses) or for three specific Ashkenazi Jewish founder mutations (2,539 analyses) were correlated with personal and family history of cancer, ancestry,   invasive   versus non  invasive   breast neoplasia, and sex.Mutations were identified in 1,720 (17.2%) of the 10,000 individuals tested, including 968 (20%) of 4,843 women with breast cancer and 281 (34%) of 824 with ovarian cancer, and the prevalence of mutations was correlated with specific features of the personal and family histories of the individuals tested. Mutations were as prevalent in high-risk women of African (25 [19%] of 133) and other non-Ashkenazi ancestries as those of European ancestry (712 [16%] of 4379) and were significantly less prevalent in women diagnosed before 50 years of age with ductal carcinoma in situ than with   invasive   breast cancer (13% v 24%, P =.0007). Of the 74 mutations identified in individuals of Ashkenazi ancestry through full sequence analysis of both BRCA1 and BRCA2, 16 (21.6%) were nonfounder mutations, including seven in BRCA1 and nine in BRCA2. Twenty-one (28%) of 76 men with breast cancer carried mutations, of which more than one third occurred in BRCA1.Specific features of personal and family history can be used to assess the likelihood of identifying a mutation in BRCA1 or BRCA2 in individuals tested in a clinical setting. 
 Tamoxifen and risk of contralateral breast cancer for BRCA1 and BRCA2 mutation carriers. To determine whether adjuvant tamoxifen treatment for breast cancer (BC) is associated with reduced contralateral breast cancer (CBC) risk for BRCA1 and/or BRCA2 mutation carriers.Analysis of pooled observational cohort data, self-reported at enrollment and at follow-up from the International BRCA1, and BRCA2 Carrier Cohort Study, Kathleen Cuningham Foundation Consortium for Research into Familial Breast Cancer, and Breast Cancer Family Registry. Eligible women were BRCA1 and BRCA2 mutation carriers diagnosed with unilateral BC since 1970 and no other   invasive   cancer or tamoxifen use before first BC. Hazard ratios (HRs) for CBC associated with tamoxifen use were estimated using Cox regression, adjusting for year and age of diagnosis, country, and bilateral oophorectomy and censoring at contralateral mastectomy, death, or loss to follow-up.Of 1,583 BRCA1 and 881 BRCA2 mutation carriers, 383 (24%) and 454 (52%), respectively, took tamoxifen after first BC diagnosis. There were 520 CBCs over 20,104 person-years of observation. The adjusted HR estimates were 0.38 (95% CI, 0.27 to 0.55) and 0.33 (95% CI, 0.22 to 0.50) for BRCA1 and BRCA2 mutation carriers, respectively. After left truncating at recruitment to the cohort, adjusted HR estimates were 0.58 (95% CI, 0.29 to 1.13) and 0.48 (95% CI, 0.22 to 1.05) based on 657 BRCA1 and 426 BRCA2 mutation carriers with 100 CBCs over 4,392 person-years of prospective follow-up. HRs did not differ by estrogen receptor status of the first BC (missing for 56% of cases).This study provides evidence that tamoxifen use is associated with a reduction in CBC risk for BRCA1 and BRCA2 mutation carriers. Further follow-up of these cohorts will provide increased statistical power for future prospective analyses. 
 Basal cytokeratin and epidermal growth factor receptor expression are not predictive of BRCA1 mutation status in women with triple-negative breast cancers. Over 80% of breast cancers in women with germline BRCA1 mutations are estrogen receptor, progesterone receptor, and human epidermal growth factor receptor 2-negative ("triple negative") and most of these have a basal-like phenotype by expression profiling and immunophenotypic analysis. However, whether or not expression of biomarkers characteristic of basal-like breast cancers helps to define a subset of women with triple-negative breast cancers who are likely to harbor BRCA1 mutations is an unresolved issue.We randomly identified 165 women from the Dana-Farber/Harvard Cancer Center SPORE annotated specimen bank with primary   invasive  , triple-negative breast cancers. Tissue microarrays were constructed by obtaining triplicate 0.6 mm cores from available paraffin blocks from 130 cases: only unstained slides were available for immunostaining from 35 cases. Slides cut from the tissue microarrays and the unstained slides were immunostained for estrogen receptor, progesterone receptor, and human epidermal growth factor receptor 2 (to confirm triple-negative status) and for several markers that have been reported to be useful in defining the basal-like phenotype, including basal cytokeratins CK5/6, CK14, and CK17 and epidermal growth factor receptor (EGFR). Full sequencing analysis for BRCA1 germline mutations was performed on blood specimens from all cases. The final study population consisted of 144 cases in which (1) triple-negative status was confirmed; (2) there was sufficient material for analysis of basal cytokeratins and EGFR; and (3) germline BRCA1 mutation status was known.Among these triple-negative breast cancer cases, 97 (67%) expressed one or more basal cytokeratins and 102 (71%) showed EGFR expression. Basal cytokeratin expression was detected in 65% of the tumor from the 20 BRCA1 mutation carriers and in 68% of the cancers from women without mutations (P=NS). EGFR expression was identified in a similar proportion of tumors from women with and without BRCA1 mutations (75% vs. 72%, P=NS).Basal cytokeratin and EGFR expression are both highly prevalent among triple-negative breast cancers. The frequency of expression of basal cytokeratins and EGFR was similar in women with and without BRCA1 mutations. Therefore, although the expression of basal cytokeratins and/or EGFR can be used to identify triple-negative breast cancers that have a basal-like phenotype, expression of these markers alone is not sufficient to distinguish which women with triple-negative breast cancers are likely to harbor BRCA1 germline mutations. 
 Methylation biomarkers for pleomorphic lobular breast cancer - a short report. Pleomorphic   invasive   lobular cancer (pleomorphic ILC) is a rare variant of ILC that is characterized by a classic ILC-like growth pattern combined with an infiltrative ductal cancer (IDC)-like high nuclear atypicality. There is an ongoing discussion whether pleomorphic ILC is a dedifferentiated form of ILC or in origin an IDC with a secondary loss of cohesion. Since gene promoter hypermethylation is an early event in breast carcinogenesis and thus may provide information on tumor progression, we set out to compare the methylation patterns of pleomorphic ILC, classic ILC and IDC. In addition, we aimed at analyzing the methylation status of pleomorphic ILC.We performed promoter methylation profiling of 24 established and putative tumor suppressor genes by methylation-specific multiplex ligation-dependent probe amplification (MS-MLPA) analysis in 20 classical ILC, 16 pleomorphic ILC and 20 IDC cases.We found that pleomorphic ILC showed relatively low TP73 and MLH1 methylation levels and relatively high RASSF1A methylation levels compared to classic ILC. Compared to IDC, pleomorphic ILC showed relatively low MLH1 and BRCA1 methylation levels. Hierarchical cluster analysis revealed a similar methylation pattern for pleomorphic ILC and IDC, while the methylation pattern of classic ILC was different.This is the first report to identify TP73, RASSF1A, MLH1 and BRCA1 as possible biomarkers to distinguish pleomorphic ILC from classic ILC and IDC. 
 The ectopic expression of BRCA1 is associated with genesis, progression, and prognosis of breast cancer in young patients. The study is to explore the histopathological features and the molecular marker expression of young women with breast cancers.The pathological data of 367 cases of female breast cancer patients were retrospectively analyzed, focusing on the analysis of young breast cancer incidence trends and the clinical and pathological features.Compared with elderly breast cancer patients, young women with breast cancers had larger tumor sizes, higher histological grades, and lymph node metastasis rates. The majority of patients were in the PTNM III stage, with the clinical and pathological features of strong   invasive  ness. The positive expression rate of the BRCA1 protein in the young group was higher than that in the old group. BRCA1 expression was positively correlated with the PTNM stage and axillary lymph node metastasis (P < 0.05).The ectopic expression of BRCA1 is associated with the genesis, progression, and prognosis of young breast cancer patients.The virtual slide(s) for this article can be found here: http://www.diagnosticpathology.diagnomx.eu/vs/1628000054838044. 
 Effect of BRCA mutations on the length of survival in epithelial ovarian tumors. To study the role of BRCA mutations in ovarian cancer survival.Blood samples and specimens of ovarian tumors (whenever blood samples were not available) at the time of the primary surgery were obtained in the course of a nationwide case-control study of women with ovarian cancer in Israel. The three common BRCA mutations in Israel (185delAG, 5382insC, and 6174delT) were analyzed with a multiplex polymerase chain reaction to amplify the exons containing the three mutations using fluor-labeled primers in a single reaction. Because each mutation is a small insertion or deletion, they can be detected as length polymorphisms. Patients were followed for up to 5 years (range, 20 to 64 months). Statistical analysis was performed using the Kaplan-Meier method and the log-rank test. Stepwise Cox regression analysis was used for determination of independent prognostic factors.This report is based on 896 blood or tumor specimens analyzed for the presence of the BRCA mutations. Of these, 234 women (26.1%) were found to be positive. A significant difference in survival pattern was found between BRCA1/BRCA2 carriers and noncarriers among the women with   invasive   ovarian cancer (median survival, 53.4 months v. 37.8 months; 3-year survival, 65.8% v. 51.9%, respectively). These differences were independent of age at diagnosis or stage of the disease.Our data indicate that the survival of patients with ovarian cancer is affected by BRCA germline mutation, at least in the early years after diagnosis. 
 Impact of teenage oral contraceptive use in a population-based series of early-onset breast cancer cases who have undergone BRCA mutation testing. Oral contraceptive (OC) use in young women has been associated with an increased risk of breast cancer. This matched case-control study aims to elucidate the combined effects of OC use and genetic factors in a population-based series of BRCA1/2 mutation-tested early-onset breast cancers. A first   invasive   breast cancer was diagnosed in 259 women aged 40 years between 1990 and 1995 in the South Swedish Health Care Region. A total of 245 women were included in this study. Information on family history of cancer, reproductive factors, smoking and OC use was obtained from questionnaires or patient charts. Three age-matched controls per case were chosen from a prospective South Swedish cohort. Ever OC use and current OC use were not associated with breast cancer. Cases were more likely to have used OCs before age 20 years (adjusted odds ratio (OR) 2.10 (95% CI 1.32-3.33)) and before their first child (adjusted OR 1.63 (95% CI 1.02-2.62)). When stratified by age, the effect of early OC use was limited to women diagnosed prior to age 36 years (OR 1.53 (1.17-1.99) per year of OC use prior to age 20 years). The risks were similar for low-dose and high-dose OCs. The probability of being a BRCA1/2 mutation carrier was three times higher among cases who started OC use prior to age 20 years compared with cases who started at age 20 years or older or who had never used OCs. However, the duration of OC use was similar among cases with and without BRCA1/2 mutations. No association was seen with a first-degree family history of breast cancer. Each year of OC use prior to age 20 years conferred a significantly increased risk for early-onset breast cancer, while there was no risk associated with use after age 20 years. 
 Candidate serous cancer precursors in fallopian tube epithelium of BRCA1/2 mutation carriers. Occult   invasive   and intraepithelial carcinomas have been identified in the tubal fimbria of BRCA mutation carriers undergoing prophylactic surgery, and recently described lesions overexpressing p53 in the distal tubes of mutation carriers, and non-carriers, have been proposed as histological precursors of high-grade serous carcinoma. The aim of this study was to confirm these findings in a larger, independent case set, to further characterize the cancer precursor lesions, and to determine their frequency in BRCA mutation-positive (n=176) and control groups (n=64). For the purposes of this study, we excluded cases without documentation of a germline mutation of BRCA1/2, and without histological examination of the entire tube, and cases with a diagnosis of   invasive   carcinoma. Controls included salpingectomies from women undergoing surgery for reasons other than ovarian malignancy. Diagnostic categories were assigned based on combined histological review and immunostaining results. Histological abnormalities were identified in 23% of the BRCA group and in 25% of the control group, and included localized p53 overexpression in 20% of the BRCA group and 25% of the control group. Tubal intramucosal carcinoma was identified in 8% of the BRCA cases and in 3% of the control group. Four cases of intraepithelial carcinoma (21%) did not overexpress p53. There was no significant difference in the median age, frequency of histological abnormalities, p53 signatures, or tubal intraepithelial carcinoma between the BRCA mutation-positive and control groups. This large, blinded review of tubes from BRCA mutation carriers confirms previous reports of putative cancer precursors in distal tubal mucosa, and that p53 signatures occur with similar frequency in women at low and high genetic risk of tubal/ovarian carcinoma. Tubal intraepithelial carcinoma, which, like   invasive   serous cancer, usually but not always overexpresses p53 protein, is more frequent in BRCA mutation carriers. 
 Risk-reducing salpingo-oophorectomy (RRSO) in BRCA mutation carriers: experience with a consecutive series of 111 patients using a standardized surgical-pathological protocol. Women carriers of BRCA mutations often have occult malignancy found at the time of risk-reducing bilateral salpingo-oophorectomy (RRSO). We report outcomes in 111 consecutive BRCA-positive women who had RRSO using a rigorous surgical-pathological protocol from 1996 to 2008.We identified risk factors associated with finding an occult malignancy at RRSO with outcomes followed for a median of 61 months.A total of 111 BRCA carriers elected RRSO, 10 patients [9.1%] had 14 sites of occult neoplasia. Two patients had   invasive   serous fallopian tube carcinoma (TSC) only, 1 patient had   invasive   serous ovarian carcinoma (OSC) only, 5 patients had tubal intraepithelial carcinoma (TIC) only, and 2 patients had multifocal lesions of the ovary (OSC) and TIC. Occult ovarian carcinomas were only detected in BRCA1 patients, and all BRCA2 carcinomas involved only the fallopian tube. The odds of finding occult carcinoma is 4 times greater (odds ratio, 4.3; 95% confidence interval, 1.06-20.7) in women older than 50 than in younger ones (P=0.023). A history of   invasive   breast cancer was associated with a reduced risk of occult carcinoma (odds ratio, 0.2; 95% confidence interval, 0.05-0.85). In median follow-up of 5 years, recurrence rate after detection of an occult carcinoma was 10% and the risk for primary peritoneal carcinoma was less than 1%.A rigorous surgical protocol with meticulous pathologic review at RRSO yielded an overall detection rate of 9.1% for occult gynecological carcinoma in BRCA mutation carriers followed by a multidisciplinary team at a single institution. Primary peritoneal carcinoma after RRSO is rare. 
 Measuring women's preferences for breast cancer treatments and BRCA1/BRCA2 testing. In establishing decision models in the treatment and prevention of breast cancer, it is important to evaluate patients' preferences for such interventions. The objectives of the present study were: (i) to characterize women's preferences for breast cancer treatments and BRCA1/BRCA2 testing, using the rating scale and standard gamble techniques; and (ii) to identify factors associated with these quality of life indices. Data were collected from women with breast cancer (n = 60), high-risk relatives of women with breast cancer (n = 58), and women in the general population (n = 51). Regardless of group membership, participants favoured treatment and prevention options that involved minimal physical   invasive  ness. Women with breast cancer rated lumpectomy and radiation treatment more highly than high-risk relatives and women in the general population. Preferences did not differ according to participants' intentions to undergo BRCA testing. Age was the only demographic variable associated with health state preferences. These findings hold implications for the application of patient preferences to clinical decision making. 
 A pilot genome-wide association study of early-onset breast cancer. High-density oligonucleotide microarrays containing a large number of single nucleotide polymorphisms (SNPs) have enabled genome-wide association (GWA) analysis to become a reality. We used the early access Affymetrix Mendel Nsp 250K chips in a GWA case-control pilot study to identify genomic regions associated with breast cancer. We included 30 randomly sampled incident   invasive   breast cancer cases aged <45 years without deleterious mutations in the BRCA1 or BRCA2 genes, and 30 population controls individually matched on age, ethnicity and geographical area. The overall genotype call rate was 97.13+/-1.33% for controls and 97.48+/-1.42% for cases. Comparison was made between cases and controls for 203,477 genotyped SNPs using (a) unconditional logistic regression (ULR), (b) conditional logistic regression (CLR) models with adjustment for the matched pairs, (c) allelic tests for single marker tests and (d) haplotype trend regression (HTR). Genomic control and EIGENSTRAT methods were used for correction of population stratification in appropriate models. We demonstrate the similarity and dissimilarity of results from different statistical analyses. We found several possible significant regions harboring biologically meaningful known candidate genes, such as genes encoding fibroblast growth factor, transforming growth factor, epidermal growth factor, and estrogen synthesis enzymes to be associated with early-onset breast cancer. In single marker analysis, none of the SNPs were statistically significant after correction for multiple testing. However, haplotype association tests, using 90730 tag-SNPs, suggested two regions in GLG1 and UGT1 genes retaining significance even after Bonferroni correction. Nevertheless, without systematic replication, findings from this pilot study, especially the associations of breast cancer in relation to specific SNPs, should be interpreted with great caution. 
 A locus on 19p13 modifies risk of breast cancer in BRCA1 mutation carriers and is associated with hormone receptor-negative breast cancer in the general population. Germline BRCA1 mutations predispose to breast cancer. To identify genetic modifiers of this risk, we performed a genome-wide association study in 1,193 individuals with BRCA1 mutations who were diagnosed with   invasive   breast cancer under age 40 and 1,190 BRCA1 carriers without breast cancer diagnosis over age 35. We took forward 96 SNPs for replication in another 5,986 BRCA1 carriers (2,974 individuals with breast cancer and 3,012 unaffected individuals). Five SNPs on 19p13 were associated with breast cancer risk (P(trend) = 2.3 × 10⁻⁹ to P(trend) = 3.9 × 10⁻⁷), two of which showed independent associations (rs8170, hazard ratio (HR) = 1.26, 95% CI 1.17-1.35; rs2363956 HR = 0.84, 95% CI 0.80-0.89). Genotyping these SNPs in 6,800 population-based breast cancer cases and 6,613 controls identified a similar association with estrogen receptor-negative breast cancer (rs2363956 per-allele odds ratio (OR) = 0.83, 95% CI 0.75-0.92, P(trend) = 0.0003) and an association with estrogen receptor-positive disease in the opposite direction (OR = 1.07, 95% CI 1.01-1.14, P(trend) = 0.016). The five SNPs were also associated with triple-negative breast cancer in a separate study of 2,301 triple-negative cases and 3,949 controls (P(trend) = 1 × 10⁻⁷) to P(trend) = 8 × 10⁻⁵; rs2363956 per-allele OR = 0.80, 95% CI 0.74-0.87, P(trend) = 1.1 × 10⁻⁷ 
 Risk factors for non-  invasive   lesions of the fallopian tube in BRCA mutation carriers. To identify risk factors for the presence of a non-  invasive   lesion of the fallopian tube in women with a BRCA1 or BRCA2 mutation.173 BRCA mutation carriers underwent a prophylactic salpingo-oophorectomy at the University Health Network, Toronto between 2000 and 2008 and were evaluated for the presence of a non-  invasive   lesion of the fallopian tube. Patients were classified as having p53 overexpression ("p53-signature"), a tubal intra-epithelial carcinoma (TIC) or normal tubal epithelium. We obtained a risk factor questionnaire from all patients. We calculated odds ratios for several risk factors, comparing patients with a tubal abnormality to those with normal histology.Of the 173 patients, 43 (25%) were found to have a tubal lesion, including 23% of the BRCA1 mutation carriers and 27% of the BRCA2 mutation carriers. The prevalence of a non-  invasive   tubal lesion increased with age; an abnormality was present in 5% of women who had surgery before the age of 40 (1 of 12) and in 56% of women who underwent surgery at age 60 or above (6 of 13; p=0.004). A non-  invasive   lesion of either type was found in 31.2% of women with a BMI > 25 kg/m2) compared to 18.0% of patients with a BMI < 25 kg/m2 at the time of surgery (p=0.05). The average duration of oral contraceptive use among women with normal tubes was 6.0 years, compared to an average duration of 4.0 years for women with a p53 signature (p=0.09) and was 2.7 years for women with a TIC (p=0.0003).The prevalence of tubal p53 signature and TIC increases with age at salpingectomy and with BMI. Oral contraceptive use is associated with a decrease in the prevalence of TICs. 
 High frequency of HIF-1alpha overexpression in BRCA1 related breast cancer. Hypoxia is a hallmark of cancer. Hypoxia inducible factor-1alpha (HIF-1alpha) is the key regulator of the hypoxia response. HIF-1alpha is overexpressed during sporadic breast carcinogenesis and correlated with poor prognosis. Little is known on the role of HIF-1alpha in hereditary breast carcinogenesis. A recent study suggests a role for BRCA1 in HIF-1alpha regulation. We therefore examined the expression of HIF-1alpha in BRCA1 related breast cancers. By immunohistochemistry we studied expression of HIF-1alpha and some of its downstream targets in 30 hereditary   invasive   breast cancers in comparison with 200 sporadic controls. HIF-1alpha overexpression was significantly more frequent in BRCA1 related breast cancers (27/30, 90%) than in sporadic controls (88/200, 44%) (P < 0.0001). 19/30 (63%) of BRCA1 tumors showed perinecrotic (hypoxia induced) and 8/30 (27%) a diffuse HIF-1alpha overexpression pattern, the latter more likely related to genetic alterations in oncogenes and tumor suppressor genes. In contrast, sporadic breast cancer HIF-1 expressing tumors showed an inverse ratio of perinecrotic/diffuse expression (31 vs. 69%, P = 0.0002). Glut-1 and CAIX, downstream HIF1 targets, were expressed in 27/30 (90%) and 15/21 (71%) of hereditary cases versus 54/183 (29%) and 24/183 (13%) in sporadic cases. p300 levels, necessary for HIF-1 downstream activation, were significantly higher in hereditary cases (20/21, 95%) compared to sporadic cases (91/183, 50%, P = 0.0001). In conclusion, in BRCA1 germline mutation related breast cancer, functional HIF-1alpha overexpression is seen at a much higher frequency than in sporadic breast cancer, mostly hypoxia induced. This points to an important role of hypoxia and its key regulator HIF-1alpha in hereditary breast carcinogenesis. 
 Expression of BRCA1 protein in breast cancer and its prognostic significance. BRCA1 is a tumor suppressor gene which, when mutated, is associated with the development of hereditary breast cancers. In sporadic tumors, although inherent gene mutations are rare, loss of BRCA1, resulting from reduced expression or incorrect subcellular localization, is postulated to be important. The purpose of the current study was to examine the expression and localization of BRCA1 protein and to assess its prognostic value, in a well-characterized series of unselected breast carcinomas. We have examined BRCA1 in a series of   invasive   breast carcinoma (1940 cases) using tissue microarray and immunohistochemistry, to evaluate its expression pattern and to correlate this with clinicopathologic variables and patient outcome. In breast cancer, complete loss of nuclear expression was observed in 223 cases (15%) and cytoplasmic expression was found in 541 breast cancers (36.6%). Absent or reduced nuclear BRCA1 expression was observed more frequently in ductal carcinoma of no special type and medullary-like carcinoma and less frequently in lobular and tubular mixed carcinomas. It was also associated with high-grade, advanced lymph node stage, larger size, vascular invasion, negative estrogen receptor, progesterone receptor and androgen receptor expression, and positive p53 and P-cadherin expression, and with the basal-like class of breast cancer. Altered BRCA1 was associated with shorter disease-free interval. Cytoplasmic expression was also associated with development of recurrence and positive EGFR and HER2 expression. It showed an inverse association with survival particularly in low-grade, small-size, and estrogen receptor-positive subgroups. In the grade 1 subgroup, multivariate analysis with adjustment for other prognostic factors showed that cytoplasmic expression of BRCA1 was an independent predictor of disease-free interval. BRCA1 alteration may play a significant role in the development and progression of breast cancer. Immunohistochemical assessment of BRCA1 expression could provide additional clinically relevant information in routine classification of breast cancer. 
 Frequencies of BRCA1 and BRCA2 mutations among 1,342 unselected patients with   invasive   ovarian cancer. The heritable fraction of ovarian cancer exceeds that of any other common adult cancer. Most inherited cases of ovarian cancer are due to a germline mutation in BRCA1 or BRCA2. It is important to have an accurate estimate of the proportion of ovarian cancer patients who carry a mutation and the specific factors which predict the presence of a mutation.We tested a population-based series of 1342 unselected patients diagnosed with   invasive   ovarian cancer between 1995-1999 and 2002-2004 in Ontario, Canada, for germline mutations in BRCA1 and BRCA2. The two genes were tested in their entirety, using a range of techniques, including multiplex ligation-dependent probe amplification (MLPA).Among the 1342 women, 176 women carried a mutation (107 in BRCA1, 67 in BRCA2, and two in both genes) for a combined mutation frequency of 13.3%. Seven deletions were identified using MLPA (3.9% of all detected mutations). The prevalence of mutations was particularly high among women diagnosed in their forties (24.0%), in women with serous ovarian cancer (18.0%) and women of Italian (43.5%), Jewish (30.0%) or Indo-Pakistani origin (29.4%). A mutation was seen in 33.9% of women with a first-degree relative with breast or ovarian cancer and in 7.9% of women with no first-degree relative with breast or ovarian cancer. No mutation was seen in women with mucinous carcinoma.BRCA1 and BRCA2 mutations are common in women with   invasive   ovarian cancer. All women diagnosed with   invasive   non-mucinous ovarian cancer should be considered to be candidates for genetic testing. 
 Frequently increased epidermal growth factor receptor (EGFR) copy numbers and decreased BRCA1 mRNA expression in Japanese triple-negative breast cancers. Triple-negative breast cancer (estrogen receptor-, progesterone receptor-, and HER2-negative) (TNBC) is a high risk breast cancer that lacks specific therapy targeting these proteins.We studied 969 consecutive Japanese patients diagnosed with   invasive   breast cancer from January 1981 to December 2003, and selected TNBCs based on the immunohistochemical data. Analyses of epidermal growth factor receptor (EGFR) gene mutations and amplification, and BRCA1 mRNA expression were performed on these samples using TaqMan PCR assays. The prognostic significance of TNBCs was also explored. Median follow-up was 8.3 years.A total of 110 (11.3%) patients had TNBCs in our series. Genotyping of the EGFR gene was performed to detect 14 known EGFR mutations, but none was identified. However, EGFR gene copy number was increased in 21% of TNBCs, while only 2% of ER- and PgR-positive, HER2-negative tumors showed slightly increased EGFR gene copy numbers. Thirty-one percent of TNBCs stained positive for EGFR protein by immunohistochemistry. BRCA1 mRNA expression was also decreased in TNBCs compared with controls. Triple negativity was significantly associated with grade 3 tumors, TP53 protein accumulation, and high Ki67 expression. TNBC patients had shorter disease-free survival than non-TNBC in node-negative breast cancers.TNBCs have an aggressive clinical course, and EGFR and BRCA1 might be candidate therapeutic targets in this disease. 
 Population attributes affecting the prevalence of BRCA mutation carriers in epithelial ovarian cancer cases in israel. The objective was to evaluate the prevalence of BRCA1/2 mutations in selected categories of ovarian cancer patients in Israel.Blood samples and specimens of ovarian tumors were obtained in the course of a national case control study of women with ovarian cancer in Israel. Eight hundred ninety-six patients with epithelial ovarian cancer, 40 cases with nonepithelial ovarian cancer, and 68 with primary peritoneal cancer were tested for the BRCA mutations. Analysis of the three common BRCA mutations in Israel (185delAG, 5382insC in BRCA1, and 6174delT in BRCA2) was done using a multiplex polymerase chain reaction assay. A multivariate logistic regression model was used to assess the association of mutation carrier status and other factors (age, origin, family history, and clinical variables).Of the 779   invasive   epithelial ovarian cancer cases, 29.4% were mutation carriers. The prevalence of the mutations was higher among women below age 60 and in more advanced cases. The prevalence was low in mucinous tumors. There was almost a twofold excess of mutations among women with positive family history (45.7%), but still 26.5% of the family history negative cases were carriers. As expected, we found a higher rate of mutation carriers among the Ashkenazi group (34.2%) and 55% among Ashkenazi women with positive family history. No subjects born in North Africa were mutation positive.BRCA mutations are strongly associated with ovarian cancer and they are present in variable rates in distinct age, ethnic, and histopathologic categories. 
 BRCA mutations in women with ductal carcinoma in situ. The strength of the association between ductal carcinoma in situ (DCIS) and BRCA mutations has not been defined.Mutation frequency was compared in three groups: (1) a prevalent series of women with DCIS, (2) an incident series of women with DCIS, and (3) a clinic-based series of women with DCIS referred for hereditary cancer risk assessment. In groups 1 and 2, limited to Ashkenazi Jewish (AJ) cases, mutation frequency was compared with that in age-matched AJ controls with   invasive   breast cancer (IBC).In group 1, 3 of 62 (4.8%) women with DCIS and 15 of 130 (11.5%) controls with IBC had BRCA mutations. In group 2, 0 of 58 (0%) women with DCIS and 6 of 116 (5.2%) controls with IBC had BRCA mutations [combined odds ratios (OR) in groups 1 and 2: 3.64, 95% confidence interval (95% CI), 1.06-12.46; P=0.04]. In group 3, deleterious mutations were identified in 10 of 79 (12.7%) probands with DCIS, similar to the frequency in IBC probands. In group 3, mutations were associated with family history of ovarian cancer (OR, 13.35; 95% CI, 2.48-71.94; P=0.003) or early onset breast cancer (OR, 16.23; 95% CI, 1.68-157.01; P=0.02) but not with AJ ethnicity or age at diagnosis.BRCA mutations were less frequent in women with DCIS not selected for family history or age at diagnosis than in women with IBC. Nonetheless, mutations were found in a significant proportion of women with DCIS who presented for hereditary risk assessment. 
 Impact of germline BRCA1 mutations and overexpression of p53 on prognosis and response to treatment following breast carcinoma: 10-year follow up data. Overexpression of p53 has been associated with poor survival following breast carcinoma. BRCA1 interacts biochemically with p53 and may also contribute to poor outcome when constitutionally mutated. The joint effect of both abnormalities has not been studied. The primary objective of this study was to assess the impact of germline BRCA1 mutations and p53 overexpression on survival after 10 years of follow-up.A historical cohort of Ashkenazi Jewish women 65 years or younger with   invasive   breast carcinoma was tested for BRCA1 founder mutations. p53 overexpression was assessed by immunohistochemistry. Clinicopathologic information was obtained by chart review.In total, 278 women were analyzed. On univariate analysis, p53 overexpression (n = 63) was prognostic for worse overall survival (relative risk [RR] 2.6, P = 0.001) whereas BRCA1 germline mutations (n = 30) were of borderline significance (RR 1.9, P = 0.052). In the lymph node-negative subpopulation, BRCA1 mutation status conferred a higher mortality on univariate (RR 5.6, P < 0.001) and multivariate (RR 3.5, P = 0.03) analyses. There was a trend in favor of a worse prognosis for women who carried a germline BRCA1 mutation and whose tumor overexpressed p53. When compared with noncarriers, BRCA1 mutation carriers had a worse overall survival if they did not receive adjuvant chemotherapy (RR 3.3, P= 0.01) or adjuvant hormonal therapy (RR 2.3, P = 0.02).Germline BRCA1 mutations and p53 overexpression carry a negative prognosis that is not additive to known prognostic factors. Given the experimental sensitivity of BRCA1-mutated cells to chemotherapy, the worse survival among BRCA1 mutation-carrying lymph node-negative breast carcinoma patients may be partly explained by the significantly lower proportion of lymph node-negative patients who received adjuvant chemotherapy (P < 0.001). 
 Microcephalin is a new novel prognostic indicator in breast cancer associated with BRCA1 inactivation. The authors have investigated the expression of the microcephalin (MCPH1) protein to evaluate its prognostic importance in breast cancer. Microcephalin is a damage response protein involved in the regulation of BRCA1 and BRCA2. BRCA1 mutations are often associated with basal-like breast cancer, which are also often negative for oestrogen receptor (ER), progesterone receptor (PR) and HER2. MCPH1 immunohistochemistry was performed on 319 breast cancers prepared as tissue microarray and correlated with pathology, survival, ER, PR, HER2, EGFR, CK5/6, CK14 and BRCA1 expression. After performing continuous data analysis, mean microcephalin expression decreased with increasing grade (P < 0.006). Mean microcephalin expression was lower in ER/PR negative (P < 0.001) and triple negative cancers (P < 0.004). Conversely, an association with HER2-positive cancers was also identified (P < 0.034). Reduced microcephalin also correlated with reduced nuclear BRCA1 staining (P < 0.001). No association was identified with basal markers. After dichotomising the data into low and high microcephalin expression, reduced expression was identified in 29% (93/319) of breast cancers. An association with low expression was identified in   invasive   ductal carcinomas with breast cancer-specific survival (BCSS) (P = 0.052). Multivariate analysis of ductal carcinomas showed that microcephalin, together with lymph node involvement and tumour size were independent predictors of BCSS (P = 0.037). Microcephalin expression is reduced in 29% of breast cancers, particularly in higher grade tumours and BRCA1-negative cases. Microcephalin is an independent predictor of BCSS in   invasive   ductal breast cancer patients and may prove to be a useful biomarker for the identification of aggressive breast cancers. 
 Promoter hypermethylation of TMS1, BRCA1, ERalpha and PRB in serum and tumor DNA of   invasive   ductal breast carcinoma patients. Breast cancer is fast emerging as the leading cancer amongst females, especially in younger age group in India; a large proportion of these tumors are often aggressive and ER and/or PR negative. Promoter methylation of genes involved in DNA repair and hormonal regulation may, in part, account for this behavior. To test this hypothesis methylation status of tumor suppressor genes TMS1, BRCA1, ERalpha and PRB was determined in   invasive   ductal carcinoma of breast and paired serum DNA. Of the 50 breast cancer patients investigated, 36/50 (72%) tumors and 32/50 (64%) paired sera showed methylation of at least one of these genes, while 17/50 (34%) tumors and 12/50 (24%) sera showed methylation of three genes. Methylation frequencies ranged from 24% for TMS1 to 63% for ERalpha. Significant association was observed between ERalpha and PRB methylation (p< or =0.001) and there was concordance between DNA methylation in tumor and serum for each gene. Immunohistochemical analysis showed no detectable expression of ERalpha, PRB and BRCA1 in 21/36 (58%), 20/36 (56%) and 23/36 (64%) tumors respectively; significant correlation was observed between promoter methylation and loss of protein expression confirming our hypothesis that promoter methylation is an important mechanism for transcriptional silencing of these genes in breast cancer in this population. This study also underscores the potential utility of DNA methylation based screening of serum (a surrogate for tumor DNA methylation) as a tool for detection of breast cancer. 
  GeneRIFs  
 BRCA1 mutation is associated with   invasive   breast cancer. 
 Data indicate tumor suppressors TP73, RASSF1A, MLH1 and BRCA1 as possible biomarkers to distinguish Pleomorphic   invasive   lobular cancer (pleomorphic ILC} from classic ILC and infiltrative ductal cancer (IDC). 
 Fertility treatments are not associated with an increased risk of   invasive   epithelial ovarian cancer in Jewish Israeli BRCA1/2 mutation carriers. 
 for women with   invasive   ovarian cancer, the short-term survival advantage of carrying a BRCA1 or BRCA2 mutation does not lead to a long-term survival benefit 
   Invasive   tubal carcinoma and TIC were limited to BRCA-mutation carriers, whereas hyperplasia and minor epithelial atypia were commonly found in both BRCA-mutation carriers and controls. 
 Sixty-two patients (index cases) with   invasive   breast cancer belonging to sixty families and their asymptomatic female first-degree relatives (300 cases) were studied for germline mutations of BRCA1 and BRCA2 genes. 
 BRCA1/p220 loss of expression or function triggers BRCA1-IRIS overexpression through a post-transcriptional mechanism, which in turn promotes formation of aggressive and   invasive   breast tumors. 
 Among patients with   invasive   epithelial ovarian cancer, having a germline mutation in BRCA1 or BRCA2 was associated with improved 5-year overall survival. BRCA2 carriers had the best prognosis. 
 study found no difference in the prevalence of loss of wt BRCA1 between ER+ and ER-   invasive   BRCA1-associated breast cancers 
 BRCA1 and BRCA2 mutations are common in women with   invasive   ovarian cancer. All women diagnosed with   invasive   non-mucinous ovarian cancer should be considered to be candidates for genetic testing. 
 loss of BRCA1 function may contribute to the aggressiveness of Ras-MAPK driven breast cancer with associated increase in levels of cyclin D1 and c-myc, enhanced MAPK activity, angiogenic potential &   invasive  ness 
 The prophylactic procedure is highly effective in preventing   invasive   breast cancer in BRCA1 mutation carriers 
 In this prospective study of women who were unaffected at the time of genetic testing and who were negative for the known familial mutation in BRCA1/2, no excess risk of   invasive   breast cancer was observed. 
 Ninteen percent of the women who developed both   invasive   breast and ovarian tumors carried one of the analyzed BRCA1 gene mutations but none of the women were positive for the analyzed BRCA2 mutation. 
  [Mm]etastasize  PubMed  
 Downregulation of BRCA1 in A375 melanoma cell line increases radio-sensitivity and modifies metastatic and angiogenic gene expression. The participation of BRCA1 (breast cancer 1) in DNA repair is well established, especially in mammary and ovarian cells. Our purpose was to develop a new in vivo radio-sensitizing therapy for melanoma. We therefore investigated the effect of downregulation of BRCA1 on irradiated melanoma cells using an anti-BRCA1 ribozyme. Our results show that BRCA1 downregulation increased radio-sensitivity of the A375 cell line, suggesting that BRCA1 could act as a caretaker in melanoma; however, as BRCA1 functions are not limited to maintaining genomic integrity but also regulate transcription and the cell cycle, we confirmed that the proliferative rate of BRCA1 downregulated clones did not change. We also demonstrate that: (1) among the major pro-angiogenic genes, FGF-2 was not increased before or after irradiation and vascular endothelial growth factor strongly inhibited after irradiation; (2) expression of two important metalloproteinases, matrix metalloproteinase 2 and 9, involved in melanoma metastasis were decreased before and after irradiation; (3) expression of their major inhibitor, tissue inhibitor of metalloproteinase, was mainly upregulated; and (4) that invasion of BRCA1 downregulated cells was modified. Together these data suggest that BRCA1 downregulation in melanoma cells did not make them more aggressive and could lead to new therapeutic strategies for this tumor, which is so difficult to control once   metastasize  d. 
 Increased incidence of visceral metastases in scottish patients with BRCA1/2-defective ovarian cancer: an extension of the ovarian BRCAness phenotype. To compare the frequency of visceral relapse of BRCA1/2-deficient ovarian cancer to that of nonhereditary controls.All patients diagnosed in Scotland with epithelial ovarian cancer (EOC) or primary peritoneal cancer (PPC) and a germline BRCA1/2 mutation were identified. Those with previous malignancy were excluded. Each remaining patient who experienced relapse was matched with two nonhereditary controls.Seventy-nine patients with EOC/PPC and germline BRCA1/2 mutations were identified. Fifteen had inadequate clinical data, two had carcinosarcoma, 27 had previous breast cancer, and 16 were in remission. Of the remaining 19 patients who were BRCA1/2 deficient, 14 patients (74%) developed visceral metastases compared with six (16%) of 38 patients in the control group. The percentages of liver, lung, and splenic metastases were 53%, 32%, and 32%, respectively, in the patients compared with 5%, 3%, and 5%, respectively, in the controls. When events occurring outside the matched follow-up period were omitted, the percentages of visceral, liver, lung, and splenic metastases were 58%, 42%, 16%, and 32% in the patients compared with 5%, 0%, 0%, and 3% in controls (P < .001, P < .001, P = .066, and P = .011, respectively). In an independent validation set, the corresponding percentages of visceral, liver, lung, and splenic metastases were 63%, 46%, 13%, and 17% in the patients compared with 11%, 4%, 2%, and 2% in controls (P < .001, P < .001, P = .153, and P = .052, respectively).Although sporadic EOC commonly remains confined to the peritoneum, BRCA1/2-deficient ovarian cancer frequently   metastasize  s to viscera. These data extend the ovarian BRCAness phenotype, imply BRCA1/2-deficient ovarian cancer is biologically distinct, and suggest that patients with visceral metastases should be considered for BRCA1/2 sequencing. 
  GeneRIFs  
 BRCA1 downregulation in melanoma cells did not make them more aggressive and could lead to new therapeutic strategies for this tumor, which is so difficult to control once   metastasize  d. 
  [Mm]etastasis  PubMed  
 MYC overexpression and poor prognosis in sporadic breast cancer with BRCA1 deficiency. Breast cancer is a complex disease; the molecular mechanisms involved in sporadic breast carcinogenesis remain to be elucidated. The present study aimed to explore the deficiency of breast cancer susceptibility gene 1 (BRCA1), including protein loss expression, promoter hypermethylation and gene copy deletion, its correlationship with other tumor markers expression (TP53, MYC, etc.), and clinical significance in sporadic breast cancer. BRCA1 protein expression was negative in 226 of 374 (60.4%) cases of this study. Cases negative for BRCA1 protein were more often with pathological tumor-node-  metastasis   stage III, positive for lymph node   metastasis   and MYC overexpression than BRCA1-positive tumors. BRCA1 hypermethylation was detected in 16.4% (31 of 189) breast cancers, which correlated with BRCA1 negative, ER negative, MYC overexpression, and triple-negative phenotype. In addition, the percentage of cells with BRCA1 gene copy deletion was significantly increased in BRCA1-methylated tumors. Kaplan-Meier survival analysis showed that patients with BRCA1-negative expression showed a worse overall survival (OS) than those with BRCA1-positive expression, and patients with BRCA1-methylated tumors had a significantly worse disease-free survival than did patients with unmethylated tumors. Furthermore, BRCA1 hypermethylation showed an inverse association with OS in LN-positive or p53-negative subgroup patients. Importantly, uni- and multivariate Cox regression analyses revealed that BRCA1 was an independent prognostic indicator of OS in sporadic breast cancer. Thus, we found MYC overexpression and poor prognosis in sporadic breast cancer with BRCA1 deficiency. The targeting of BRCA1 deficiency in combination with MYC-pathways inhibitors may provide a promising strategy for sporadic breast cancer care, the triple-negative subtype in particular. 
 Microenvironmental regulation of BRCA1 gene expression by c-Jun and Fra2 in premalignant human ovarian surface epithelial cells. Reduced BRCA1 gene expression is common in the sporadic form of ovarian carcinoma. The spread of this highly lethal cancer often begins when tumor cell clusters are shed into the fluid of the abdominopelvic cavity such that they can float freely before seeding distant sites on the peritoneal walls and organs. Thus, the microenvironment that tumor cells find themselves in changes dramatically during these early shedding and floating stages of transperitoneal   metastasis  . To mimic this microenvironmental change in vitro, we released premalignant human ovarian surface epithelial cells from the substratum and forced them to cluster in suspension. Under these conditions, steady state levels of BRCA1 mRNA and protein fell significantly and the transcriptional activation state of the BRCA1 promoter was suppressed. Analysis of the promoter indicated that the previously identified "CRE" element located within the "positive regulatory region" (PRR) contributed to this suppression. More specifically, we show that the suppression was mediated, at least in part, by a suspension culture-driven decrease in the levels of two members of the AP1 transcription factor complex, c-Jun and Fra2, that bind to the CRE element. Therefore, a microenvironmental change that is manifested during the initial stages of ovarian carcinoma dissemination may, potentially, help suppress BRCA1 expression in sporadic tumors and thus promote their progression. 
 MYC overexpression and poor prognosis in sporadic breast cancer with BRCA1 deficiency. Breast cancer is a complex disease; the molecular mechanisms involved in sporadic breast carcinogenesis remain to be elucidated. The present study aimed to explore the deficiency of breast cancer susceptibility gene 1 (BRCA1), including protein loss expression, promoter hypermethylation and gene copy deletion, its correlationship with other tumor markers expression (TP53, MYC, etc.), and clinical significance in sporadic breast cancer. BRCA1 protein expression was negative in 226 of 374 (60.4%) cases of this study. Cases negative for BRCA1 protein were more often with pathological tumor-node-  metastasis   stage III, positive for lymph node   metastasis   and MYC overexpression than BRCA1-positive tumors. BRCA1 hypermethylation was detected in 16.4% (31 of 189) breast cancers, which correlated with BRCA1 negative, ER negative, MYC overexpression, and triple-negative phenotype. In addition, the percentage of cells with BRCA1 gene copy deletion was significantly increased in BRCA1-methylated tumors. Kaplan-Meier survival analysis showed that patients with BRCA1-negative expression showed a worse overall survival (OS) than those with BRCA1-positive expression, and patients with BRCA1-methylated tumors had a significantly worse disease-free survival than did patients with unmethylated tumors. Furthermore, BRCA1 hypermethylation showed an inverse association with OS in LN-positive or p53-negative subgroup patients. Importantly, uni- and multivariate Cox regression analyses revealed that BRCA1 was an independent prognostic indicator of OS in sporadic breast cancer. Thus, we found MYC overexpression and poor prognosis in sporadic breast cancer with BRCA1 deficiency. The targeting of BRCA1 deficiency in combination with MYC-pathways inhibitors may provide a promising strategy for sporadic breast cancer care, the triple-negative subtype in particular. 
 Microenvironmental regulation of BRCA1 gene expression by c-Jun and Fra2 in premalignant human ovarian surface epithelial cells. Reduced BRCA1 gene expression is common in the sporadic form of ovarian carcinoma. The spread of this highly lethal cancer often begins when tumor cell clusters are shed into the fluid of the abdominopelvic cavity such that they can float freely before seeding distant sites on the peritoneal walls and organs. Thus, the microenvironment that tumor cells find themselves in changes dramatically during these early shedding and floating stages of transperitoneal   metastasis  . To mimic this microenvironmental change in vitro, we released premalignant human ovarian surface epithelial cells from the substratum and forced them to cluster in suspension. Under these conditions, steady state levels of BRCA1 mRNA and protein fell significantly and the transcriptional activation state of the BRCA1 promoter was suppressed. Analysis of the promoter indicated that the previously identified "CRE" element located within the "positive regulatory region" (PRR) contributed to this suppression. More specifically, we show that the suppression was mediated, at least in part, by a suspension culture-driven decrease in the levels of two members of the AP1 transcription factor complex, c-Jun and Fra2, that bind to the CRE element. Therefore, a microenvironmental change that is manifested during the initial stages of ovarian carcinoma dissemination may, potentially, help suppress BRCA1 expression in sporadic tumors and thus promote their progression. 
 Association of BRCA1 promoter methylation with sporadic breast cancers: Evidence from 40 studies. Breast cancer susceptibility gene 1 (BRCA1) located at chromosome 17q12-21 is a classic tumor suppressor gene, and has been considered as a significant role in hereditary breast cancers. Moreover, numerous studies demonstrated the methylation status of CpG islands in the promoter regions of BRCA1 gene was aberrant in patients with sporadic breast tumors compared with healthy females or patients with benign diseases. However, these conclusions were not always consistent. Hence, a meta-analysis was performed to get a more precise estimate for these associations. Crude odds ratio with 95% confidence interval were used to assess the association of BRCA1 promoter methylation and the risk or clinicopathologic characteristics of breast cancers under fixed or random effect model. A total of 40 studies were eligible for this present study. We observed the frequency of BRCA1 promoter methylation was statistically significant higher in breast cancers than non-cancer controls. Furthermore, BRCA1 methylation was statistically associated with lymph node   metastasis  , histological grade 3, ER(-), PR(-), triple-negative phenotype, and decreased or lack levels of BRCA1 protein expression. In conclusion, this study indicated that BRCA1 promoter methylation appeared to be a useful predictive or prognostic biomarker for breast cancers in clinical assessment. 
 HOXA9 regulates BRCA1 expression to modulate human breast tumor phenotype. Breast cancer 1, early onset (BRCA1) expression is often reduced in sporadic breast tumors, even in the absence of BRCA1 genetic modifications, but the molecular basis for this is unknown. In this study, we identified homeobox A9 (HOXA9) as a gene frequently downregulated in human breast cancers and tumor cell lines and noted that reduced HOXA9 transcript levels associated with tumor aggression,   metastasis  , and patient mortality. Experiments revealed that loss of HOXA9 promoted mammary epithelial cell growth and survival and perturbed tissue morphogenesis. Restoring HOXA9 expression repressed growth and survival and inhibited the malignant phenotype of breast cancer cells in culture and in a xenograft mouse model. Molecular studies showed that HOXA9 restricted breast tumor behavior by directly modulating the expression of BRCA1. Indeed, ectopic expression of wild-type BRCA1 phenocopied the tumor suppressor function of HOXA9, and reducing BRCA1 levels or function inhibited the antitumor activity of HOXA9. Consistently, HOXA9 expression correlated with BRCA1 in clinical specimens and with tumor aggression in patients lacking estrogen receptor/progesterone receptor expression in their breast tissue. These findings indicate that HOXA9 restricts breast tumor aggression by modulating expression of the tumor suppressor gene BRCA1, which we believe provides an explanation for the loss of BRCA1 expression in sporadic breast tumors in the absence of BRCA1 genetic modifications. 
 The Leu33Pro polymorphism in the ITGB3 gene does not modify BRCA1/2-associated breast or ovarian cancer risks: results from a multicenter study among 15,542 BRCA1 and BRCA2 mutation carriers. Integrins containing the beta(3) subunit are key players in tumor growth and   metastasis  . A functional Leu33Pro polymorphism (rs5918) in the beta(3) subunit of the integrin gene (ITGB3) has previously been suggested to act as a modifier of ovarian cancer risk in Polish BRCA1 mutation carriers. To investigate the association further, we genotyped 9,998 BRCA1 and 5,544 BRCA2 mutation carriers from 34 studies from the Consortium of Investigators of Modifiers of BRCA1/2 for the ITGB3 Leu33Pro polymorphism. Data were analysed within a Cox-proportional hazards framework using a retrospective likelihood approach. There was marginal evidence that the ITGB3 polymorphism was associated with an increased risk of ovarian cancer for BRCA1 mutation carriers (per-allele Hazard Ratio (HR) 1.11, 95% CI 1.00-1.23, p-trend 0.05). However, when the original Polish study was excluded from the analysis, the polymorphism was no longer significantly associated with ovarian cancer risk (HR 1.07, 95% CI 0.96-1.19, p-trend 0.25). There was no evidence of an association with ovarian cancer risk for BRCA2 mutation carriers (HR 1.09, 95% CI 0.89-1.32). The polymorphism was not associated with breast cancer risk for either BRCA1 or BRCA2 mutation carriers. The ITGB3 Leu33Pro polymorphism does not modify breast or ovarian cancer risk in BRCA1 or BRCA2 mutation carriers. 
 Clinical significance of epigenetic inactivation of hMLH1 and BRCA1 in Tunisian patients with invasive breast carcinoma. Aberrant hypermethylation of gene promoter regions is one of the mechanisms for inactivation of tumour suppressor genes in many human cancers including breast carcinoma. In the current study, we aimed to assess by MSP, the methylation pattern of two cancer-related genes involved in DNA repair: hMLH1 (mutL homolog 1, colon cancer, nonpolyposis type 2 (E. coli) and BRCA1 (breast cancer 1, early onset) in 78 primary breast cancers from Tunisian patients. The methylation frequencies were 24.36% for hMLH1 and 46% for BRCA1. BRCA1 methylation correlated with age at diagnosis (P = .015) and 5-years disease free survival (P = .016) while hMLH1 methylation was more frequent in larger tumors (P = .002) and in presence of distant   metastasis   (P = .004). Furthermore, methylation of hMLH1 significantly correlated with high level of P53 expression (P = .006) and with overall survival (P = .015) suggesting that silencing of hMLH1 through aberrant promoter methylation could be used as a poor prognosis indicator in breast cancer. 
 Activated networking of platelet activating factor receptor and FAK/STAT1 induces malignant potential in BRCA1-mutant at-risk ovarian epithelium. It is essential to understand the molecular basis of ovarian cancer etiology and tumor development to provide more effective preventive and therapeutic approaches to reduce mortality. Particularly, the molecular targets and pathways involved in early malignant transformation are still not clear. Pro-inflammatory lipids and pathways have been reported to play significant roles in ovarian cancer progression and   metastasis  . The major objective of this study was to explore and determine whether platelet activating factor (PAF) and receptor associated networking pathways might significantly induce malignant potential in BRCA1-mutant at-risk epithelial cells.BRCA1-mutant ovarian epithelial cell lines including (HOSE-636, HOSE-642), BRCA1-mutant ovarian cancer cell (UWB1.289), wild type normal ovarian epithelial cell (HOSE-E6E7) and cancerous cell line (OVCA429), and the non-malignant BRCA1-mutant distal fallopian tube (fimbria) tissue specimens were used in this study. Mutation analysis, kinase microarray, western blot, immune staining, co-immune precipitation, cell cycle, apoptosis, proliferation and bioinformatic pathway analysis were applied.We found that PAF, as a potent pro-inflammatory mediator, induced significant anti-apoptotic effect in BRCA1-mutant ovarian surface epithelial cells, but not in wild type HOSE cells. With kinase microarray technology and the specific immune approaches, we found that phosphor-STAT1 was activated by 100 nM PAF treatment only in BRCA1-mutant associated at-risk ovarian epithelial cells and ovarian cancer cells, but not in BRCA1-wild type normal (HOSE-E6E7) or malignant (OVCA429) ovarian epithelial cells. Co-immune precipitation revealed that elevated PAFR expression is associated with protein-protein interactions of PAFR-FAK and FAK-STAT1 in BRCA1-mutant ovarian epithelial cells, but not in the wild-type control cells.Previous studies showed that potent inflammatory lipid mediators such as PAF and its receptor (PAFR) significantly contribute to cancer progression and   metastasis  . Our findings suggest that these potent inflammatory lipids and receptor pathways are significantly involved in the early malignant transformation through PAFR-FAK-STAT1 networking and to block apoptosis pathway in BRCA1 dysfunctional at-risk ovarian epithelium. 
 Histone methyltransferase EZH2 induces Akt-dependent genomic instability and BRCA1 inhibition in breast cancer. Increased levels of EZH2, a critical regulator of cellular memory, signal the presence of   metastasis   and poor outcome in breast cancer patients. High levels of EZH2 are associated with nuclear pleomorphism, lack of estrogen receptor expression, and decreased nuclear levels of BRCA1 tumor suppressor protein in invasive breast carcinomas. The mechanism by which EZH2 overexpression promotes the growth of poorly differentiated invasive carcinomas remains to be defined. Here, we show that EZH2 controls the intracellular localization of BRCA1 protein. Conditional doxycycline-induced upregulation of EZH2 in benign mammary epithelial cells results in nuclear export of BRCA1 protein, aberrant mitoses with extra centrosomes, and genomic instability. EZH2 inhibition in CAL51 breast cancer cells induces BRCA1 nuclear localization and rescues defects in ploidy and mitosis. Mechanistically, EZH2 overexpression is sufficient for activation of the phosphoinositide 3-kinase/Akt (PI3K/Akt) pathway specifically through activation of Akt isoform 1. EZH2-induced BRCA1 nuclear export, aneuploidy, and mitotic defects were prevented by treatment with the PI3K inhibitors LY294002 or wortmannin. Targeted inhibition of Akt-1, Akt-2, and Akt-3 isoforms revealed that the EZH2-induced phenotype requires specific activation of Akt-1. The relevance of our studies to human breast cancer is highlighted by the finding that high EZH2 protein levels are associated with upregulated expression of phospho-Akt-1 (Ser473) and decreased nuclear expression of phospho-BRCA1 (Ser1423) in 39% of invasive breast carcinomas. These results enable us to pinpoint one mechanism by which EZH2 regulates BRCA1 expression and genomic stability mediated by the PI3K/Akt-1 pathway. 
 The Fanconi anemia family of genes and its correlation with breast cancer susceptibility and breast cancer features. Fanconi anemia (FA) family of proteins participates in the DNA repair pathway by homologous recombination, and it is currently formed by 13 genes. Some of these proteins also confer susceptibility to hereditary breast and ovarian cancer (HBOC), since FANCD1 is the BRCA2 breast cancer susceptibility gene, and FANCN/PALB2 and FANCJ/BRIP1 explain 2% of non-BRCA1/2 HBOC families. Thus, there is an important connection between FA and BRCA pathways. In a previous case-control association study analysing FANCA, FANCD2 and FANCL, we reported an association between FANCD2 and sporadic breast cancer (BC) risk (OR = 1.35). In order to know whether variants in other FA genes could also be involved in this association, we have extended our study with the rest of FA genes and some others implicated in the BRCA pathway. We have also analyzed the correlation with survival, nodal   metastasis   and hormonal receptors (ER- and PR-). A total of 61 SNPs in ten FA genes (FANC-B, -C, -D1, -E, -F, -G, -I, -J, -M, -N) and five FA related genes (ATM, ATR, BRCA1, H2AX and USP1) were studied in a total of 547 consecutive and nonrelated sporadic BC cases and 552 unaffected controls from the Spanish population. Association analyses reported marginal statistically significant results with the minor allele of intronic SNPs in three genes: BRCA1, BRCA2/FANCD1, and ATM. Survival association with SNPs on FANCC and BRCA2/FANCD1 genes were also reported. Sub-group analyses revealed associations between SNPs on FANCI and ATM and nodal   metastasis   status and between FANCJ/BRIP1 and FANCN/PALB2 and PR- status. 
 Outcome of metastatic breast cancer in selected women with or without deleterious BRCA mutations. The aim of this study was to compare the time-to progression and overall survival (OS) in patients with metastatic breast cancer (MBC) with and without deleterious BRCA1/2 mutations. 195 women with MBC who were referred for BRCA genetic testing between 1997 and 2011 were included in the study. Logistic regression models and Cox proportional hazards models were fit to determine the associations between clinical variables and outcomes. Of 195 women with MBC, 21 % (n = 41) were positive for BRCA1/2 mutations. The number of metastatic sites at the time of metastatic disease was not different between BRCA1 versus BRCA2 carriers versus non-carriers (P = 0.77). The site of first   metastasis   was visceral-only in 70 % of BRCA1 carriers compared to 9 % in BRCA2 carriers and 37 % in non-carriers (P = 0.001). Median follow-up was 2.8 years. BRCA non-carriers and BRCA2 carriers had a longer time-to progression and OS compared to BRCA1 carriers (median time-to progression = 1.3 vs. 0.9 vs. 0.7 years; P = 0.31, and median OS = 4.88 vs. 4.94 vs. 1.34 years; P = 0.0065). In a multivariate model, no association was identified between BRCA positivity and time-to-event outcomes (P > 0.28). In addition, patients with triple-negative MBC carried a poorer prognosis irrespective of their BRCA status (P = 0.058 and P = 0.15 for the interaction term of BRCA status and triple-negative for time-to progression and OS, respectively). Our data indicate that BRCA1 carriers diagnosed with MBC have worse outcomes compared to BRCA2 carriers and non-carriers. However, the differences in outcome did not reach statistical significance likely due to small sample sizes. 
 miR-185 suppresses tumor proliferation by directly targeting E2F6 and DNMT1 and indirectly upregulating BRCA1 in triple-negative breast cancer. Breast cancer is a major public health problem all over the world, and the current treatment strategies are not potent enough for some patients, especially those with triple-negative breast cancer (TNBC). Recent studies have demonstrated that microRNAs (miRNA) play vital roles in the development of TNBC. In this study, we found that miR-185 was strongly downregulated in TNBC tissues and cell lines and that its expression levels were associated with lymph node   metastasis  , clinical stage, overall survival, and relapse-free survival in TNBC. We also found that ectopic expression of miR-185 inhibited TNBC cell proliferation in vitro and in vivo. We further identified that miR-185 directly targeted DNMT1 and E2F6, which resulted in a marked increase in the expression of BRCA1 at the mRNA and protein levels in TNBC. Our data suggest that miR-185 functions as a tumor suppressor in TNBC development. It is a promising prognostic biomarker and potential therapeutic target for TNBC. 
 Identification of a preneoplastic gene expression profile in tubal epithelium of BRCA1 mutation carriers. Microinvasive carcinomas and high-grade intraepithelial neoplasms are commonly discovered within the fallopian tube of BRCA1 mutation carriers at the time of risk-reducing salpingo-oophorectomy, suggesting that many BRCA1-mutated ovarian carcinomas originate in tubal epithelium. We hypothesized that changes in gene expression profiles within the histologically normal fallopian tube epithelium of BRCA1 mutation carriers would overlap with the expression profiles in BRCA1-mutated ovarian carcinomas and represent a BRCA1 preneoplastic signature. Laser capture microdissection of frozen sections was used to isolate neoplastic cells or histologically normal fallopian tube epithelium, and expression profiles were generated on Affymetrix U133 Plus 2.0 gene expression arrays. Normal-risk controls were 11 women wild type for BRCA1 and BRCA2 (WT-FT). WT-FT were compared with histologically normal fallopian tube epithelium from seven women with deleterious BRCA1 mutations who had foci of at least intraepithelial neoplasm within their fallopian tube (B1-FTocc). WT-FT samples were also compared with 12 BRCA1 ovarian carcinomas (B1-CA). The comparison of WT-FT versus B1-FTocc resulted in 152 differentially expressed probe sets, and the comparison of WT-FT versus B1-CA resulted in 4079 differentially expressed probe sets. The BRCA1 preneoplastic signature was composed of the overlap between these two lists, which included 41 concordant probe sets. Genes in the BRCA1 preneoplastic signature included several known tumor suppressor genes such as CDKN1C and EFEMP1 and several thought to be important in invasion and   metastasis   such as E2F3. The expression of a subset of genes was validated with quantitative reverse transcription-polymerase chain reaction and immunohistochemistry. 
 DNA methyltransferase 1/3a overexpression in sporadic breast cancer is associated with reduced expression of estrogen receptor-alpha/breast cancer susceptibility gene 1 and poor prognosis. DNA methyltransferases (DNMTs), including DNMT1, 3a, and 3b, play an important role in the progression of many malignant tumors. However, it remains unclear whether expression of DNMTs is associated with the development of breast cancer. This study aimed to explore the clinical significance of DNMT proteins in sporadic breast cancer. We investigated the expression of DNMT1, 3a, and 3b in 256 breast cancer and 36 breast fibroadenoma, using immunohistochemistry. The expression of DNMT1 and 3a was significantly higher in breast cancer than in fibroadenoma. In breast cancer, the expression of DNMT1 was significantly correlated with lymph node   metastasis   (P = 0.020), and the expression of DNMT3a and 3b was significantly correlated with advanced clinical stages (P = 0.046 and 0.012, respectively). Overexpression of DNMT1/3a was correlated with promoter hypermethylation and reduced expression of ERα and BRCA1. The expression levels of DNMT1 or DNMT3a were associated with a significantly shorter DFS or OS in a subgroup of breast cancer patients (patients with the age ≤50 years old, ERα-negative status, or HER2-postive status). The expression of DNMT1 or a combined expression of DNMT1 and 3a was associated with poor prognosis in patients who received chemotherapy and endocrine therapy, but not in patients who received chemotherapy alone. These findings suggest that DNMT1 and 3a may be involved in the progression and prognosis of sporadic breast cancer. 
 Expression profile of BRCA1 and BRCA2 genes in premenopausal Mexican women with breast cancer: clinical and immunohistochemical correlates. Low BRCA1 gene expression is associated with increased invasiveness and influences the response of breast carcinoma (BC) to chemotherapeutics. However, expression of BRCA1 and BRCA2 genes has not been completely characterized in premenopausal BC. We analyzed the clinical and immunohistochemical correlates of BRCA1 and BRCA2 expression in young BC women. We studied 62 women (mean age 38.8 years) who developed BC before the age of 45 years. BRCA1 and BRCA2 mRNA expression was assessed by reverse transcriptase-polymerase chain reaction (RT-PCR) and that of HER-2 and p53 proteins by immunohistochemistry. Body mass index (BMI) > or = 27 (52%) and a declared family history of BC (26%) were the main risk factors. Ductal infiltrative adenocarcinoma was found in 86% of the cases (tumor size >5 cm in 48%). Disease stages I-IV occurred in 2, 40, 55, and 3%, respectively (73% implicating lymph nodes). Women aged < or = 35 years (24%) had more family history of cervical cancer, stage III/IV disease, HER-2 positivity, and lower BRCA1 expression than older women (P < 0.05). BRCA1 and BRCA2 expression correlated in healthy, but not in tumor tissues (TT). Neither BRCA1 nor BRCA2 expression was associated with tumor histology, differentiation, nodal   metastasis   or p53 and HER-2 expression. After multivariate analysis, only disease stage explained BRCA1 mRNA levels in the lowest quartile. Premenopausal BC has aggressive clinical and molecular characteristics. Low BRCA1 mRNA expression is associated mainly with younger ages and advanced clinical stage of premenopausal BC. BRCA2 expression is not associated with disease severity in young BC women. 
 Downregulation of BRCA1 in A375 melanoma cell line increases radio-sensitivity and modifies metastatic and angiogenic gene expression. The participation of BRCA1 (breast cancer 1) in DNA repair is well established, especially in mammary and ovarian cells. Our purpose was to develop a new in vivo radio-sensitizing therapy for melanoma. We therefore investigated the effect of downregulation of BRCA1 on irradiated melanoma cells using an anti-BRCA1 ribozyme. Our results show that BRCA1 downregulation increased radio-sensitivity of the A375 cell line, suggesting that BRCA1 could act as a caretaker in melanoma; however, as BRCA1 functions are not limited to maintaining genomic integrity but also regulate transcription and the cell cycle, we confirmed that the proliferative rate of BRCA1 downregulated clones did not change. We also demonstrate that: (1) among the major pro-angiogenic genes, FGF-2 was not increased before or after irradiation and vascular endothelial growth factor strongly inhibited after irradiation; (2) expression of two important metalloproteinases, matrix metalloproteinase 2 and 9, involved in melanoma   metastasis   were decreased before and after irradiation; (3) expression of their major inhibitor, tissue inhibitor of metalloproteinase, was mainly upregulated; and (4) that invasion of BRCA1 downregulated cells was modified. Together these data suggest that BRCA1 downregulation in melanoma cells did not make them more aggressive and could lead to new therapeutic strategies for this tumor, which is so difficult to control once metastasized. 
 Distant disease-free interval, site of first relapse and post-relapse survival in BRCA1- and BRCA2-associated compared to sporadic breast cancer patients. Data on distant disease-free interval (DDFI) and the localization of the first distant   metastasis   (DM) in BRCA1- and BRCA2-associated breast cancer (BC) patients are as yet scarcely available.We identified 57 BRCA1-associated and 31 BRCA2-associated BC patients, diagnosed between 1980 and 2001, and developing DM disease before 2004, July 1. DDFI, the site(s) of first DM and post-relapse survival of these patients were compared with those of 192 sporadic BC patients.As compared to sporadic patients, BRCA1 patients developed less often bone DM (30% vs. 51%; P = 0.005), but tended to develop more often lung DM (26% vs. 16%; P = 0.07), and DM at multiple sites (44% vs. 32%; P = 0.11). In BRCA2-associated compared to sporadic patients, first DM more commonly occurred in lymph nodes (23% vs. 7%; P = 0.007) and at multiple sites (48% vs. 32%; P = 0.08). Adjuvant systemic therapy appeared to be most effective in BRCA2 mutation carriers. Post-relapse survival was worse for BRCA1- and better for BRCA2-associated patients as compared to sporadic patients, but differences disappeared after adjustment for ER-status, site of first DM and DDFI.The site of first DM is different between BRCA1- and BRCA2-associated and sporadic BC patients. Differences in post-relapse survival could be explained by differences in site of first DM, in ER-status and in DDFI. Treatment efficacy may differ dependent on genetic status. 
 Reduced cell death, invasive and angiogenic features conferred by BRCA1-deficiency in mammary epithelial cells transformed with H-Ras. To investigate the role of tumor suppressors BRCA1 and p53 proteins in human breast tumorigenesis, we transformed immortalized human mammary epithelial cells, MCF10A, with or without BRCA1/p53 gene-specific knockdowns. Stable knockdown of BRCA1 alone in MCF10A cells led to centrosome amplification, impaired p53 protein stability, increased sensitivity towards DNA-damaging agents, defective chromosomal condensation at mitosis and elevated protein levels of cyclin D1 and c-myc. While over-expression of mutant H-Ras transformed MCF10A cells, depletion of BRCA1 dramatically enhanced the in vivo tumorigenesis that was associated with higher levels of VEGF, enhanced vascularization and less apoptosis in the BRCA1-deficient Ras-transformed tumors. The Ras-transformed BRCA1-deficient tumors exhibited features of the epithelial-to-mesenchymal transition, appeared to secrete matrix metalloproteases as visualized by in vivo bio-imaging of tumors using fluorescent probe MMP680, and were locally metastatic to lymph nodes. Our results suggest that loss of BRCA1 function may contribute to the aggressiveness of Ras-MAPK driven human breast cancer with associated increase in levels of cyclin D1 and c-myc, enhanced MAPK activity, angiogenic potential & invasiveness. This mammary xenograft tumor model may be useful as a tool to understand human breast tumor angiogenesis and   metastasis  , as well as to test candidate therapeutics. 
 Prognostic value of ERCC1, RRM1, BRCA1 and SETDB1 in early stage of non-small cell lung cancer. Nowadays, 40 % of early-stage NSCLC patients relapse in the 2 years following resection, suggesting a mis-staging in this group of patients who are not receiving adjuvant chemotherapy. Although different biomarkers, such as ERCC1, RRM1 and BRCA1 have been found to present prognostic value in advanced NSCLC patients, in early-stage NSCLC patients its relevance remains unclear. Moreover, SETDB1 has been recently proposed as a bona fide oncogene in lung tumourigenesis and related wit  h metastas  is. The aim of the present study was to analyze the prognostic value of ERCC1, RRM1, BRCA1 and SETDB1 expression levels in NSCLC patients at stage I.ERCC1, RRM1, BRCA1 and SETDB1 expression at mRNA level was analyzed by real-time quantitative RT-PCR in fresh-frozen tumor and normal adjacent lung tissue samples from 64 stage I NSCLC patients. Later, significant association between gene expression levels, clinicopathological characteristics and patient's disease-free survival was assessed.We did not find any statistically significant correlation between gene expression and gender, age, histological type or smoking status. Univariate followed by multivariate Cox analysis showed that higher levels of BRCA1 and SETDB1 expression were significantly associated with shorter disease-free survival in stage I NSCLC patients.Our study finds that ERCC1 and RRM1 are not independent prognostic factors of recurrence in stage I NSCLC patients. By contrast, BRCA1 and SETDB1 stand out as the most significant prognostic markers in this group of patients, appearing as promising tools to predict tumor recurrence in early-stage NSCLC patients. 
 Methylation of BRCA1 promoter region is associated with unfavorable prognosis in women with early-stage breast cancer. BRCA1-associated breast cancers are associated with particular features such as early onset, poor histological differentiation, and hormone receptor negativity. Previous studies conducted in Taiwanese population showed that the mutation of BRCA1 gene does not play a significant role in the occurrence of breast cancer. The present study explored methylation of BRCA1 promoter and its relationship to clinical features and outcome in Taiwanese breast cancer patients. Tumor specimens from a cohort of 139 early-stage breast cancer patients were obtained during surgery before adjuvant treatment for DNA extraction. Methylation of BRCA1 promoter region was determined by methylation-specific PCR and the results were related to clinical features and outcome of patients using statistical analysis. Methylation of the BRCA1 promoter was detected in 78 (56%) of the 139 tumors. Chi-square analysis indicated that BRCA1 promoter methylation correlated significantly with triple-negative (ER-/PR-/HER2-) status of breast cancer patients (p = 0.041). The Kaplan-Meier method showed that BRCA1 promoter methylation was significantly associated with poor overall survival (p = 0.026) and disease-free survival (p = 0.001). Multivariate analysis which incorporated variables of patients' age, tumor size, grade, and   lymph nod  e metastasis revealed that BRCA1 promoter methylation was associated with overall survival (p = 0.027; hazard ratio, 16.38) and disease-free survival (p = 0.003; hazard ratio, 12.19) [corrected].Our findings underscore the clinical relevance of the methylation of BRCA1 promoter in Taiwanese patients with early-stage breast cancer. 
 Clinical significance of promoter hypermethylation of RASSF1A, RARbeta2, BRCA1 and HOXA5 in breast cancers of Indian patients. Promoter hypermethylation of genes is implicated in the pathogenesis of many cancers, including breast cancer. Herein, we analyzed the promoter methylation status of a panel of critical growth regulatory genes, RASSF1A, RARbeta2, BRCA1 and HOXA5, in 54 breast cancers and 5 distant normal breast tissues of Indian patients. The methylation data were correlated with clinicopathological characteristics and hormone receptor status to determine the impact of methylation in breast carcinogenesis. Promoter hypermethylation of RASSF1A was observed in 39/54 (72%), HOXA5 in 36/54 (67%), BRCA1 in 15/54 (28%) and RARbeta2 in 8/54 (15%) breast cancers. Our most significant findings were the association of RASSF1A methylation with nodal   metastasis   (p=0.05); and RARbeta2 methylation with age (all tumors in patients in the older age group were methylated, p=0.04). Further, the interactions between DNA methylation and hormone receptor biology in breast cancer cells are beginning to be clearly understood. In this context the association of HOXA5 methylation with loss of ERalpha (p=0.009) is noteworthy. 
 Expression of TRF1, TRF2, TIN2, TERT, KU70, and BRCA1 proteins is associated with telomere shortening and may contribute to multistage carcinogenesis of gastric cancer. Telomere dysfunction is believed to be a significant factor in carcinogenesis. To elucidate the carcinogenesis mechanism in gastric cancer, the expression of telomeric proteins and changes in telomere length were investigated during multistage carcinogenesis of gastric cancer.Tissue samples were obtained during surgical operations from the normal gastric mucosa of 10 patients, the precancerous lesions of 15 patients, the gastric cancer tissues (GC) of 20 patients, and of tumors due to gastric cancer with lymph node   metastasis   (GCLM) from 5 patients. The expression of TRF1, TRF2, and TIN2 proteins was measured by Western blotting, while the expression of TERT, KU70, and BRCA1 proteins was detected using the immunohistochemical method. The mean telomere length was determined by Southern blotting.Compared with normal gastric mucosa tissues, the expression of TRF1, TRF2, and TIN2 proteins was significantly higher in precancerous lesions, GC, and GCLM (P < 0.01). The expression of TRF1, TRF2, and TIN2 proteins was significantly higher in GC and GCLM than in precancerous lesions (P < 0.01). The expression of TERT and Ku70 proteins in precancerous lesions and GC tissues was significantly higher than that in normal gastric mucosa tissues (P < 0.01). The expression of TERT and Ku70 proteins in GC tissues was significantly higher than in precancerous lesions (P < 0.01). In normal gastric mucosa, the BRCA1 protein was primarily located in the cell nucleus. In precancerous lesions and GC, the expression of the BRCA1 protein was apparent in the cell cytoplasm. The mean telomere length in precancerous lesions, GC, and GCLM was significantly shorter than that in normal gastric mucosa tissues (P < 0.05). The mean telomere length in GC and GCLM was significantly shorter than that in precancerous lesions (P < 0.05). The mean telomere length in all tissue samples was inversely correlated with the level of TRF1, TRF2, TIN2, TERT, and Ku70 proteins.Our results suggest that the over-expression of telomeric proteins, TRF1, TRF2, TIN2, TERT, and Ku70, and the transposition of the BRCA1 protein may work together to reduce the telomere length in precancerous lesions and gastric cancer, and could contribute to the multistage carcinogenesis of gastric cancer. These findings offer new insight into the mechanism of carcinogenesis in gastric cancer. 
 BRCA mutations cause reduction in miR-200c expression in triple negative breast cancer. Triple negative breast cancer (TNBC) is the most aggressive and poorly understood subclass of breast cancer (BC). Over the recent years, miRNA expression studies have been providing certain detailed overview that aberrant expression of miRNAs is associated with TNBC. Although TNBC tumors are strongly connected with loss of function of BRCA genes, there is no knowledge about the effect of BRCA mutation status on miRNA expressions in TNBC cases. The aims of this study were to evaluate the expression profile of miRNAs that plays role in TNBC progression and the role of BRCA mutations in their regulation. The expression level of BC associated 13 miRNAs was analyzed in 7 BRCA mutations positive, 6 BRCA mutations negative TNBC cases and 20 non-tumoral tissues using RT-PCR. According to RT2 Profiler PCR Array Data Analysis, let-7a expression was 4.67 fold reduced in TNBCs as compared to normal tissues (P=0.031). In addition, miR-200c expression was 5.75 fold reduced in BRCA mutation positive TNBC tumors (P=0.005). Analysis revealed a negative correlation between miR-200c and VEGFA expressions (r=-468). Thus, miR-200c may be involved in invasion and   metastasis   in TNBC cases with BRCA mutation. In this study we provide the knowledge on the first report of association between microRNA-200c and BRCA mutations in TNBC. Further studies and evaluations are required, but this miRNA may provide novel therapeutic molecular targets for TNBC treatment and new directions for the development of anticancer drugs. 
 Downregulation of EZH2 decreases growth of estrogen receptor-negative invasive breast carcinoma and requires BRCA1. Increased levels of enhancer of zeste homolog 2 (EZH2), a critical regulator of cellular memory, are associated with negative estrogen receptor (ER) expression and disease progression in breast cancer. High levels of EZH2 signal the presence of   metastasis   and poor outcome in breast cancer patients. To test the hypothesis that deregulation of EZH2 contributes to ER-negative breast cancer progression, EZH2 expression was inhibited in ER-negative breast cancer cells MDA-MB-231 and CAL51 using a lentivirus system. EZH2 knockdown decreased proliferation and delayed the G(2)/M cell-cycle transition, although not affecting apoptosis. In vivo, EZH2 downregulation significantly decreased breast xenograft growth and improved survival. EZH2 knockdown upregulated BRCA1 protein. Of note, BRCA1 knockdown was sufficient to rescue the effects of EZH2 downregulation on proliferation, G(2)/M arrest, and on the levels of hyperphosphorylated mitotic Cdc25C and Cyclin B1 proteins, crucial for entry into mitosis. Invasive ER-negative breast carcinomas show significant overexpression of EZH2 and downregulation of BRCA1 proteins. Taken together, we show that EZH2 is important in ER-negative breast cancer growth in vivo and in vitro, and that BRCA1 is required for the proliferative effects of EZH2. Blockade of EZH2 may provide a prime target to prevent and/or halt ER-negative breast cancer progression. 
 A BRCA1 deficient-like signature is enriched in breast cancer brain metastases and predicts DNA damage-induced poly (ADP-ribose) polymerase inhibitor sensitivity. There is an unmet clinical need for biomarkers to identify breast cancer patients at an increased risk of developing brain metastases. The objective is to identify gene signatures and biological pathways associated with human epidermal growth factor receptor 2-positive (HER2+) brain   metastasis  .We combined laser capture microdissection and gene expression microarrays to analyze malignant epithelium from HER2+ breast cancer brain metastases with that from HER2+ nonmetastatic primary tumors. Differential gene expression was performed including gene set enrichment analysis (GSEA) using publicly available breast cancer gene expression data sets.In a cohort of HER2+ breast cancer brain metastases, we identified a gene expression signature that anti-correlates with overexpression of BRCA1. Sequence analysis of the HER2+ brain metastases revealed no pathogenic mutations of BRCA1, and therefore the aforementioned signature was designated BRCA1 Deficient-Like (BD-L). Evaluation of an independent cohort of breast cancer metastases demonstrated that BD-L values are significantly higher in brain metastases as compared to other metastatic sites. Although the BD-L signature is present in all subtypes of breast cancer, it is significantly higher in BRCA1 mutant primary tumors as compared with sporadic breast tumors. Additionally, BD-L signature values are significantly higher in HER2-/ER- primary tumors as compared with HER2+/ER + and HER2-/ER + tumors. The BD-L signature correlates with breast cancer cell line pharmacologic response to a combination of poly (ADP-ribose) polymerase (PARP) inhibitor and temozolomide, and the signature outperformed four published gene signatures of BRCA1/2 deficiency.A BD-L signature is enriched in HER2+ breast cancer brain metastases without pathogenic BRCA1 mutations. Unexpectedly, elevated BD-L values are found in a subset of primary tumors across all breast cancer subtypes. Evaluation of pharmacological sensitivity in breast cancer cell lines representing all breast cancer subtypes suggests the BD-L signature may serve as a biomarker to identify sporadic breast cancer patients who might benefit from a therapeutic combination of PARP inhibitor and temozolomide and may be indicative of a dysfunctional BRCA1-associated pathway. 
 Suppression of breast cancer invasion and migration by indole-3-carbinol: associated with up-regulation of BRCA1 and E-cadherin/catenin complexes. Indole-3-carbinol (I3C) is a compound occurring naturally in cruciferous vegetables and has been indicated as a promising agent in preventing breast cancer development and progression. In the present study we have investigated the effect of I3C on the cell migration and invasion behavior in estrogen receptor positive MCF-7 and estrogen receptor negative MDA-MB-468 human breast cancer cell lines. Both MCF-7 and MDA-MB-468 were poorly invasive cell lines and exhibited modest invasion and migration capacity in the presence of fibronectin as the chemoattractant. I3C (50 or 100 microM) elicited a significant inhibition of in vitro cell adhesion, migration, and invasion as well as in vivo lung   metastasis   formation in both cell lines. I3C also suppressed the 17beta-estradiol stimulated migration and invasion in estrogen-responsive MCF-7 cells. These results indicate that anti-invasion and antimigration activities of I3C occur via estrogen-independent and estrogen-dependent pathways. Moreover, I3C significantly caused a dose-dependent increase in E-cadherin, three major catenins (alpha, beta, and gamma-catenin) and BRCA1 expression. Our current finding is the first demonstration that I3C can activate the function of invasion suppressor molecules associated with the suppression of invasion and migration in breast cancer cells. Thus, clinical application of I3C may contribute to the potential benefit for suppression of breast cancer invasion and   metastasis  . 
 Wild-type genotypes of BRCA1 gene SNPs combined with micro-RNA over-expression in mammary tissue leading to familial breast cancer with an increased risk of distant metastases' occurrence. Germ line deleterious mutations of BRCA1 gene are not the unique factor that could inactivate BRCA1 protein which leads to familial breast cancer onset with distant metastases' occurrence. The present research explores the role that could be assigned to BRCA1 SNPs to inactivate BRCA1 protein and therefore to the occurrence of familial breast cancer with an increased risk of distant metastases' occurrence. The presence or the absence of BRCA1 protein was first analyzed by applying the immunohistochemistry technique to the tumors with sporadic and familial breast cancer. Then, a case-control study was conducted including 40 patients with familial breast cancer, 46 ones with sporadic breast cancer and 34 healthy controls based on the genotyping of nine BRCA1 SNPs (c.442.58delT, c.2082C>T, c.2311T>C, c.2612C>T, c.3113A>G, c.3119G>A, c.3548A>G, c.4308T>C and 4837A>G) via direct sequencing. Finally, the functional role that could be assigned to these SNPs was focused upon. miRbase site was used as a bioinformatics tool to predict potential micro-RNAs (miRs) targeting SNPs that are associated with familial breast cancer according to the results of this research. These predicted miRs were confirmed by Q-PCR analysis and correlated with BRCA1 protein expression among patients along with potential distant metastases. Clinical outcome showed that distant   metastasis   concerned 45 % of familial breast cancer patients and 19.5 % with sporadic breast cancer. Analysis of BRCA1 protein expression revealed a negative staining among 46.6 % of familial breast cancer patients and only 16.6 % within sporadic breast cancer ones. The association of four variants was identified within BRCA1 gene (c.442.58 delT, c.2311T>C, c.2612C>T and c.4308T>C) to familial breast cancer across their wild genotypes. miR-1179 was selected as potential miR that targets the region of BRCA1 mRNA containing the c.2311T>C variant within the TT genotype. The expression of miR-1179 was significantly associated with familial breast cancer patients without BRCA1 deleterious mutations compared to those with sporadic breast cancer according to TT genotype along with BRCA1 negative staining and according to the occurrence of distant metastases. Combination between TT genotype of c.2311T>C and miR-1179 over-expression could generate a lack of BRCA1 protein leading to a high risk of familial breast cancer with distant metastases. 
 BRCA1 and ERCC1 mRNA levels are associated with lymph node   metastasis   in Chinese patients with colorectal cancer. Although both excision repair cross-complementing group 1 (ERCC1) and breast cancer susceptibility gene 1 (BRCA1) can be effective biomarkers for chemosensitivity in primary malignant tumors, their applicability to metastases is poorly understood. Here, ERCC1 and BRCA1, which are linked to lymph node   metastasis   (LNM) in colorectal cancer (CRC), were evaluated in primary CRC samples from Chinese patients with LNM (LNM CRC) or without LNM (non-LNM CRC). mRNA levels of ERCC1 and BRCA1 in CRC samples, and their relationships to primary CRC and LNM, were also examined.Differences in BRCA1 and ERCC1 gene expression between primary CRC with or without LNM were assessed in CRC samples from 120 Chinese patients, using real-time polymerase chain reaction. Relationships between ERCC1 and BRCA1 expression and clinicopathological parameters and prognoses were also examined.ERCC1 and BRCA1 were significantly down-regulated in LNM CRC compared with non-LNM CRC. Down-expression of ERCC1 and BRCA1 was significantly associated with LNM (P < 0.001), advanced TNM stage (P < 0.001), and decreased 5-year overall survival rate (P < 0.001). Univariate and multivariate analyses showed ERCC1 and BRCA1 expression as independent predictors of recurrence and survival in CRC patients (P < 0.05).ERCC1 and BRCA1 mRNA expression levels correlate inversely to CRC   metastasis  . ERCC1 and BRCA1 might serve as biomarkers for LNM and as prognostic indicators for CRC; their down-expressions are predictors of poor outcome in CRC patients. 
 Immunohistochemical loss of BRCA1 protein in uterine serous carcinoma. Uterine serous carcinoma is a uncommon aggressive variant of endometrial cancer whose biologic origin is unclear. Mutations in p53 and BRCA1 genes play a key role in ovarian serous carcinogenesis. We investigated whether the loss of BRCA1 expression plays a similar role in uterine serous carcinoma. Loss of BRCA1 expression and Wilms tumor 1 (WT-1) overexpression were detected by immunohistochemical analysis. Depth of myometrial invasion, the presence of precursor lesions or polyps, and clinical parameters (age, history of breast cancer, and germline BRCA1 mutation status) were recorded. A total of 27 cases were available for evaluation. Three tumors (11.1%, 95% confidence interval, 2%-29%) showed the loss of BRCA1 expression. Two of these had known germline mutation in BRCA1, and the third had not been analyzed. Two of these cases expressed WT-1 or showed some morphologic features suggestive of drop   metastasis   from the adnexa, but no case showed detectable serous tubal intraepithelial carcinoma or features of an ovarian primary tumor. Overall, 5 women in the group had a personal history of breast cancer, and the finding was significantly associated with BRCA1 staining (P=0.049). A subset of uterine serous carcinomas shows the loss of BRCA1 protein and is associated with germline mutation. 
 Loss of heterozygosity at BRCA1 locus is significantly associated with aggressiveness and poor prognosis in breast cancer. BRCA1 and BRCA2 are two major tumor suppressor genes for hereditary breast and ovarian cancer. In sporadic breast cancer, although somatic mutations of these genes are rare, loss of heterozygosity (LOH) at BRCA1 and BRCA2 loci is common.LOH at BRCA1 and BRCA2 loci were investigated in 202 Japanese invasive breast cancer patients. The relationships between LOH at these gene loci and clinicopathologic characteristics were analyzed.Among 166 informative cases for both BRCA1 and BRCA2 loci, 69 (41.6%) and 52 (31.3%) tumors revealed LOH at BRCA1 and BRCA2 loci, respectively. LOH at BRCA1 LOH or BRCA2 locus was associated with higher nuclear grade (P < 0.0001, P = 0.0187). LOH at BRCA1 locus was associated with estrogen receptor and progesterone receptor negativity (P = 0.001 and P = 0.015) and significantly shorter disease-free survival (P < 0.0001), distant   metastasis  -free survival (P < 0.0001), and overall survival (P < 0.0001). In contrast, LOH at BRCA2 locus had no associations with estrogen receptor or progesterone receptor status and prognosis. LOH at BRCA1 locus was independently associated with poor prognosis in terms of disease-free survival (hazard ratio 3.08, 95% confidence interval [CI] 1.58-6.18, P = 0.0009), distant   metastasis  -free survival (hazard ratio 5.18, 95% CI 2.35-12.19, P < 0.0001), and overall survival (hazard ratio 4.97, 95% CI 1.84-15.1, P = 0.0013).LOH at BRCA1 locus could be an independent prognostic biomarker useful in identifying a subgroup of patients with poor prognosis. 
 Invasion patterns of metastatic high-grade serous carcinoma of ovary or fallopian tube associated with BRCA deficiency. High-grade serous carcinomas of the uterine adnexa with BRCA1 deficiency (high-grade serous carcinomas-BRCA) have recently been described to demonstrate characteristic histopathological features. We hypothesize that metastatic high-grade serous carcinomas-BRCA cases exhibit characteristic morphological features as well. We studied 102 high-grade serous carcinomas with known BRCA1 and BRCA2 genotype from the archives of the Department of Pathology at Memorial Sloan-Kettering Cancer Center. The primary site morphological characteristics of these cases were reported previously; we now focus solely on tumor morphology in sites other than the uterine adnexa (ie, metastatic sites). The study group consisted of the following case types: 13 BRCA1 germline mutations; 5 BRCA1 somatic mutations; 10 BRCA1 promoter methylation; 4 BRCA2 germline mutations; 1 BRCA2 somatic mutation; 11 lacking BRCA1 or BRCA2 abnormality; 58 cases lacking BRCA1 or BRCA2 germline mutation. Two observers independently scored invasion patterns and microscopic tumor architecture while blinded to genotype. Concordance between observers and correlations between metastatic patterns and the following indices were studied: genotype, primary site tumor characteristics, and BRCA1 immunohistochemistry. Concordance between observers was excellent (κ values >0.9). All cases with BRCA1 or 2 abnormalities showed either pushing pattern metastases (76%) or infiltrative metastases composed only of micropapillae (24%). In contrast, all cases lacking BRCA1 or 2 abnormalities showed infiltrative metastases that contained combinations of papillary, glandular, and, rarely, cribriform and micropapillary architecture (P<0.0001 for comparison with pushing   metastasi  s and P<0.001 for comparison with purely micropapillary architecture). Morphological assessment of metastatic carcinomas, a highly reproducible exercise, accurately correlated with BRCA1 status in every case, unlike morphological assessment of primary site adnexal high-grade serous carcinomas or BRCA1 immunohistochemistry. Metastatic high-grade serous carcinomas-BRCAs exhibit characteristic morphological features that appear more sensitive and specific for BRCA mutations than two other morphologically based prediction systems and should be easier to apply in practice. These findings should be validated prospectively in an independent cohort. 
 Adjuvant radiotherapy for primary breast cancer in BRCA1 and BRCA2 mutation carriers and risk of contralateral breast cancer with special attention to patients irradiated at younger age. The purpose of this study was to estimate the influence of adjuvant radiotherapy for primary breast cancer (BC) on the risk of contralateral BC (CBC) in BRCA1 or BRCA2 (BRCA1/2) mutation carriers, with special attention to patients irradiated at age younger than 40 years. Additionally, tendencies in locoregional treatments and rates of contralateral risk-reducing mastectomy over time were explored. In this retrospective cohort study, 691 BRCA1/2-associated BC patients treated between 1980 and 2013 were followed from diagnosis until CBC or censoring event including ipsilateral BC recurrence, distant   metastasis  , contralateral risk-reducing mastectomy, other invasive cancer diagnosis, death, or loss to follow up. Hazard ratios (HR) for CBC associated with radiotherapy were estimated using Cox regression. Median follow-up time was 8.6 years [range 0.3–34.3 years]. No association between radiotherapy for primary BC and risk of CBC was found, neither in the total population (HR 0.82, 95 % CI 0.45–1.49) nor in the subgroup of patients younger than 40 years at primary diagnosis (HR 1.36, 95 % CI 0.60–3.09). During follow-up, the number of patients at risk decreased substantially since a large proportion of patients were censored after contralateral risk-reducing mastectomy or BC recurrence. Over the years, increasing preference for mastectomy without radiotherapy compared to breast-conserving surgery with radiotherapy was found ranging from less than 30 % in 1995 to almost 50 % after 2010. The rate of contralateral risk-reducing mastectomy increased over the years from less than 40 % in 1995 to more than 60 % after 2010. In this cohort of BRCA1/2-associated BC patients, no association between radiotherapy for primary BC and risk of CBC was observed in the total group, nor in the patients irradiated before the age of 40 years. The number of patients at risk after 10 and 15 years of follow-up, however, was too small to definitively exclude harmful effects of adjuvant radiotherapy. 
 BRCA1-associated breast cancers present differently from BRCA2-associated and familial cases: long-term follow-up of the Dutch MRISC Screening Study. The Dutch MRI Screening Study on early detection of hereditary breast cancer started in 1999. We evaluated the long-term results including separate analyses of BRCA1 and BRCA2 mutation carriers and first results on survival.Women with higher than 15% cumulative lifetime risk (CLTR) of breast cancer were screened with biannual clinical breast examination and annual mammography and magnetic resonance imaging (MRI). Participants were divided into subgroups: carriers of a gene mutation (50% to 85% CLTR) and two familial groups with high (30% to 50% CLTR) or moderate risk (15% to 30% CLTR).Our update contains 2,157 eligible women including 599 mutation carriers (median follow-up of 4.9 years from entry) with 97 primary breast cancers detected (median follow-up of 5.0 years from diagnosis). MRI sensitivity was superior to that of mammography for invasive cancer (77.4% v 35.5%; P<.00005), but not for ductal carcinoma in situ. Results in the BRCA1 group were worse compared to the BRCA2, the high-, and the moderate-risk groups, respectively, for mammography sensitivity (25.0% v 61.5%, 45.5%, 46.7%), tumor size at diagnosis≤1 cm (21.4% v 61.5%, 40.9%, 63.6%), proportion of DCIS (6.5% v 18.8%, 14.8%, 31.3%) and interval cancers (32.3% v 6.3%, 3.7%, 6.3%), and age at diagnosis younger than 30 years (9.7% v 0%). Cumulative distan  t metastas  is-free and overall survival at 6 years in all 42 BRCA1/2 mutation carriers with invasive breast cancer were 83.9% (95% CI, 64.1% to 93.3%) and 92.7% (95% CI, 79.0% to 97.6%), respectively, and 100% in the familial groups (n=43).Screening results were somewhat worse in BRCA1 mutation carriers, but 6-year survival was high in all risk groups. 
 Gene expression profiling reveals activation of the FA/BRCA pathway in advanced squamous cervical cancer with intrinsic resistance and therapy failure. Advanced squamous cervical cancer, one of the most commonly diagnosed cancers in women, still remains a major problem in oncology due to treatment failure and distant   metastasis  . Antitumor therapy failure is due to both intrinsic and acquired resistance; intrinsic resistance is often decisive for treatment response. In this study, we investigated the specific pathways and molecules responsible for baseline therapy failure in locally advanced squamous cervical cancer.Twenty-one patients with locally advanced squamous cell carcinoma were enrolled in this study. Primary biopsies harvested prior to therapy were analyzed for whole human gene expression (Agilent) based on the patient's 6 months clinical response. Ingenuity Pathway Analysis was used to investigate the altered molecular function and canonical pathways between the responding and non-responding patients. The microarray results were validated by qRT-PCR and immunohistochemistry. An additional set of 24 formalin-fixed paraffin-embedded cervical cancer samples was used for independent validation of the proteins of interest.A 2859-gene signature was identified to distinguish between responder and non-responder patients. 'DNA Replication, Recombination and Repair' represented one of the most important mechanisms activated in non-responsive cervical tumors, and the 'Role of BRCA1 in DNA Damage Response' was predicted to be the most significantly altered canonical pathway involved in intrinsic resistance (p = 1.86E-04, ratio = 0.262). Immunohistological staining confirmed increased expression of BRCA1, BRIP1, FANCD2 and RAD51 in non-responsive compared with responsive advanced squamous cervical cancer, both in the initial set of 21 cervical cancer samples and the second set of 24 samples.Our findings suggest that FA/BRCA pathway plays an important role in treatment failure in advanced cervical cancer. The assessment of FANCD2, RAD51, BRCA1 and BRIP1 nuclear proteins could provide important information about the patients at risk for treatment failure. 
 Effect of BRCA Mutations on Metastatic Relapse and Cause-specific Survival After Radical Treatment for Localised Prostate Cancer. Germline BRCA mutations are associated with worse prostate cancer (PCa) outcomes; however, the most appropriate management for mutation carriers has not yet been investigated.To evaluate the response of BRCA carriers to conventional treatments for localised PCa by analysing   metastasis  -free survival (MFS) and cause-specific survival (CSS) following radical prostatectomy (RP) or external-beam radiation therapy (RT).Tumour features and outcomes of 1302 patients with local/locally advanced PCa (including 67 BRCA mutation carriers) were analysed. RP was undergone by 535 patients (35 BRCA); 767 received RT (32 BRCA). Median follow-up was 64 mo.Median survival and 3-, 5-, and 10-yr survival rates were estimated using the Kaplan-Meier method. Generated survival curves were compared using the log-rank test. Cox regression analyses were used to assess the prognostic value of BRCA mutations.A total of 67 BRCA carriers and 1235 noncarriers were included. At 3, 5, and 10 yr after treatment, 97%, 94%, and 84% of noncarriers and 90%, 72%, and 50% of carriers were free from   metastasis   (p<0.001). The 3-, 5- and 10-yr CSS rates were significantly better in the noncarrier cohort (99%, 97%, and 85%, respectively) than in carriers (96%, 76%, and 61%, respectively; p<0.001). Multivariate analysis confirmed BRCA mutations as an independent prognostic factor for MFS (hazard ratio [HR]: 2.36; 95% confidence interval [CI], 1.38-4.03; p=0.002) and CSS (HR: 2.17; 95% CI, 1.16-4.07; p=0.016).BRCA carriers had worse outcomes than noncarriers when conventionally treated for local/locally advanced PCa.Prostate cancer patients with germline BRCA mutations had worse outcomes than noncarriers when conventionally treated with surgery or radiation therapy. 
 ABCG2, a novel antigen to sort luminal progenitors of BRCA1- breast cancer cells. Tumor-initiating cells (TICs), aka "cancer stem cells", are believed to fuel tumors and to sustain therapy resistance and systemic   metastasis  . Breast cancer is the first human carcinoma in which a subpopulation of cells displaying a specific CD44+/CD24-/low/ESA+ antigenic phenotype was found to have TIC properties. However, CD44+/CD24-/low/ESA+ is not a universal marker phenotype of TICs in all breast cancer subtypes. The aim of this study was to identify novel antigens with which to isolate the TIC population of the basal-A/basal-like breast cancer cell lines.We used polychromatic flow-cytometry to characterize the cell surface of several breast cancer cell lines that may represent different tumor molecular subtypes. We next used fluorescence-activated cell sorting to isolate the cell subpopulations of interest from the cell lines. Finally, we explored the stem-like and tumorigenic properties of the sorted cell subpopulations using complementary in vitro and in vivo approaches: mammosphere formation assays, soft-agar colony assays, and tumorigenic assays in NOD/SCID mice.The CD44+/CD24+ subpopulation of the BRCA1-mutated basal-A/basal-like cell line HCC1937 is enriched in several stemness markers, including the ABCG2 transporter (i.e., the CD338 antigen). Consistently, CD338-expressing cells were also enriched in CD24 expression, suggesting that coexpression of these two antigenic markers may segregate TICs in this cell line. In support of ABCG2 expression in TICs, culturing of HCC1937 cells in ultra-low adherent conditions to enrich them in precursor/stem-cells resulted in an increase in CD338-expressing cells. Furthermore, CD338-expressing cells, unlike their CD338-negative counterparts, displayed stemness and transformation potential, as assessed in mammosphere and colony formation assays. Lastly, CD338-expressing cells cultured in ultra-low adherent conditions maintained the expression of CD326/EpCAM and CD49f/α6-integrin, which is a combination of antigens previously assigned to luminal progenitors.Collectively, our data suggest that CD338 expression is specific to the tumor-initiating luminal progenitor subpopulation of BRCA1-mutated cells and is a novel antigen with which to sort this subpopulation. 
 Prognosis of early-onset breast cancer based on BRCA1/2 mutation status in a French population-based cohort and review. The debate concerning poorer survival for patients with breast cancer (BC) carrying a BRCA1 germline mutation is unresolved, and requires additional data from population-based studies.We followed 232 women with invasive BC under age 46, ascertained prospectively through a French population-based BC registry and tested for BRCA1/2 mutations (median follow-up: 82 months). We compared tumour characteristics and survival rates between 21 BRCA1/2 deleterious mutation carriers and 211 non-carriers.As compared to sporadic tumours, BRCA1/2 tumours showed higher grade (P = 0.02), fewer ductal carcinoma in situ (P = 0.02), more frequent medullary histology (P = 0.02), more frequent negative oestrogen and progesterone receptors (P = 0.001 each). At 5 years, BC-specific survival,   metastasis  -free survival, ipsilateral recurrence-free survival and contralateral BC-free survival rates for BRCA1/2 mutation carriers were 95.0%, 94.7%, 100% and 90.0% respectively, compared with 89.6%, 78.2%, 88.8% and 94.4% respectively, for non-carriers (not significant). Rates for women carrying only a BRCA1 mutation were 93.3%, 93.3%, 100%, 86.7%, respectively. 76% of BRCA1/2 carriers received chemotherapy.Despite unfavourable tumour features, we found no evidence for poorer short-term survival in BRCA1 mutation carriers compared to non-carriers in this prospective population-based cohort. The high rate of BRCA1 carriers who received chemotherapy for their BC should question the positive impact of this treatment, as suggested by preclinical studies showing increased chemosensitivity of BRCA1-associated tumours. 
 Association between cytosolic expression of BRCA1 and metastatic risk in breast cancer. Although BRCA1 has been extensively studied for its role as a tumour-suppressor protein, the role of BRCA1 subcellular localisation in oncogenesis and tumour progression has remained unclear. This study explores the impact of BRCA1 mislocalisation on clinical outcomes in breast cancer.Tissue microarrays assembled from a cohort of patients with all stages of breast cancer were analysed for BRCA1 localisation and correlated with patient survival. Tissue microarrays of patients who had breast cancer that had   metastasis  ed to the lung were assembled from an independent cohort of patients. These were analysed for BRCA1 subcellular expression. In vitro studies using cultured human breast cancer cells were conducted to examine the effect of cytosolic BRCA1 on cell migration and efficiency of invasion.An inverse association was found between cytosolic BRCA1 expression and   metastasis  -free survival in patients aged >40 years. Further analysis of BRCA1 subcellular expression in a cohort of breast cancer patients with metastatic disease revealed that the cytosolic BRCA1 content of breast tumours that had   metastasis  ed to the lung was 36.0% (95% CI=(31.7%, 40.3%), which was markedly higher than what is reported in the literature (8.2-14.8%). Intriguingly, these lung metastases and their corresponding primary breast tumours demonstrated similarly high cytosolic BRCA1 distributions in both paired and unpaired analyses. Finally, in vitro studies using human breast cancer cells demonstrated that genetically induced BRCA1 cytosolic sequestration (achieved using the cytosol-sequestering BRCA1 5382insC mutation) increased cell invasion efficiency.Results from this study suggest a model where BRCA1 cytosolic mislocalisation promotes breast cancer   metastasis  , making it a potential biomarker of metastatic disease. 
 BRCA1-IRIS inactivation sensitizes ovarian tumors to cisplatin. Ovarian cancer is the first in mortalities among gynecologic cancers in the United States, often due to late diagnosis and/or acquired platinum-resistant recurrences. This study investigates whether BRCA1-IRIS is a novel treatment target for ovarian cancers and in platinum-resistant recurrences. Here we show that more than half of the ovarian cancer samples analyzed showed BRCA1-IRIS and survivin overexpression and lacked nuclear FOXO3a expression. Normal ovarian epithelial cells overexpressing BRCA1-IRIS formed   metastasis   in mice when injected in the peritoneal cavity, whereas aggressive ovarian cancer cell lines failed to form tumors or metastases in mice when BRCA1-IRIS was silenced in them. We show that BRCA1-IRIS activates two autocrine signaling loops, brain-derived neurotrophic factor/tyrosine kinase B receptor (BDNF/TrkB) and neuregulin 1 (NRG1)/ErbB2. These loops are involved in anoikis resistance and   metastasis   promotion. These loops operate in several ovarian cancer cell lines, and BRCA1-IRIS silencing or inactivation using a novel inhibitory peptide renders both non-functional and promoted cell death. In a mouse xenograft model, BRCA1-IRIS inactivation using this novel inhibitory peptide resulted in significant reduction in ovarian tumor growth. More importantly, this treatment sensitized ovarian tumors to low cisplatin concentrations. Taken together, these data strongly suggest that BRCA1-IRIS and/or BDNF/TrkB and NRG1/ErbB2 could serve as rational therapeutic targets for advanced ovarian cancers. 
 BRCA1 tumours correlate with a HIF-1alpha phenotype and have a poor prognosis through modulation of hydroxylase enzyme profile expression. There are limited data regarding the hypoxia pathway in familial breast cancers. We therefore performed a study of hypoxic factors in BRCA1, BRCA2 and BRCAX breast cancers.Immunoperoxidase staining for HIF-1alpha, PHD1, PHD2, PHD3, VEGF and FIH was carried out in 125 (38 BRCA1, 33 BRCA2 and 54 BRCAX) breast carcinomas. These were correlated with clinicopathological parameters and the intrinsic breast cancer phenotypes.BRCA1 tumours correlated with positivity for HIF-1alpha (P=0.008) and negativity for PHD3 (P=0.037). HIF-1alpha positivity (P=0.001), PHD3 negativity (P=0.037) and nuclear FIH negativity (P=0.011) was associated with basal phenotype. HIF-1alpha expression correlated with high tumour grade (P=0.009), negative oestrogen receptor (ER) status (P=0.001) and the absence of lymph node   metastasis   (P=0.028). Nuclear FIH expression and PHD3 correlated with positive ER expression (P=0.024 and P=0.035, respectively). BRCA1 cancers with positive HIF-1alpha or cytoplasmic FIH had a significantly shorter relapse-free survival (P=0.007 and P=0.049, respectively).The aggressive nature of BRCA1 and basal-type tumours may be partly explained by an enhanced hypoxic drive and hypoxia driven ER degradation because of suppressed PHD and aberrantly located FIH expression. This may have important implications, as these tumours may respond to compounds directed against HIF-1alpha or its downstream targets. 
 MTA1-mediated transcriptional repression of BRCA1 tumor suppressor gene.   Metastasis  -associated tumor antigen 1 (MTA1), a component of the nucleosome remodeling and deacetylating (NuRD) complex is routinely upregulated in several cancers. In the present study, we investigated the potential role of MTA1 in BRCA1 transcriptional repression and subsequent chromosomal instability. MTA1-NuRD complex was found to negatively regulate BRCA1 transcription by physically associating with an atypical estrogen-responsive element (ERE) on the BRCA1 promoter. Moreover, MTA1 and HDAC complex recruited to the ERE of BRCA1 promoter in an ER alpha-dependent manner. Accordingly, BRCA1 protein levels were enhanced by silencing of either MTA1 expression or by treatment with the specific histone deacetylase inhibitor trichostatin A. MTA1's strong repressive effects on BRCA1 expression was supported by our observation that cells stably overexpressing MTA1 showed centrosome amplification which has been long implicated as a phenotype for BRCA1 repression. Accordingly, overexpression of BRCA1 in cells stably over expressing MTA1 resulted in restoration of normal centrosome numbers. Together, these findings strongly implicate MTA1 in the transcriptional repression of BRCA1 leading to abnormal centrosome number and chromosomal instability. 
 Effect of BRCA1 immunohistochemical localizations on prognosis of patients with sporadic breast carcinomas. Our purpose was to investigate the expression pattern of BRCA1 protein in sporadic breast carcinomas, as well as the clinicopathological and prognostic value of its subcellular localizations. Immunohistochemistry was performed on paraffin embedded tissue specimens from 111 sporadic, invasive breast carcinomas to detect the expression of the proteins BRCA1, ER, PR, erbB2, p53 and Ki67. BRCA1 protein was detected in the nuclei and the cytoplasm of the tumor cells. Nuclear BRCA1 immunoreactivity showed no relation with the classic clinicopathological markers and the expression of cerbB2, p53 and Ki67. Reduced expression of nuclear BRCA1 protein was found to exert an independent favorable impact on both the overall and relapse-free (RF) survival of the patients (p=0.019 and p=0.043, respectively). Cytoplasmic BRCA1 was associated with none of the classic histomorphological indices, except from the lymph node   metastasis  , with which its relation was found to be inverse (p=0.05), prolonging the RF survival of the patients (p=0.05). Our findings suggest that BRCA1 protein depicts different prognostic significance, according to its subcellular distribution. Nuclear detection of the protein was associated with a worse prognosis, while the cytoplasmic one was related to fewer recurrences as a result of fewer lymph node metastases. 
 BRCA1 and pancreatic cancer: pedigree findings and their causal relationships. Anecdotal reports and series studies indicate that 5-10% of pancreatic cancer (PC) cases are familial. In addition, PC is associated with a variety of hereditary cancer syndromes. PC appears to be an integral cancer in the hereditary breast-ovarian cancer (HBOC) syndrome, with most notice given to the role of BRCA2. Our purpose is to call attention to BRCA1, which also predisposes to PC. Using data from our familial breast cancer registry, we identified 19 BRCA1/2 families that contain PC affecteds in the pedigrees, 15 with BRCA1 mutations and 4 with BRCA2 mutations. The association between BRCA2 and pancreatic cancer is well established; however, a definite link with pancreatic cancer in families carrying a BRCA1 mutation has been far less studied. Thus, the focus of this report is on 9 of the 15 BRCA1 families, in which PC affecteds were either confirmed carriers of the BRCA1 mutation or were inferred as probable obligate BRCA1 mutation carriers. The numbers are small, but nevertheless illustrate the finding of others of an apparent association between PC and BRCA1-mutation-bearing families. Given the dismal prognosis of PC, with the only current hope for survival being through surgical extirpation of the pancreas prior to   metastasis  , it is prudent that we realize the potential predisposition toward PC via BRCA1, in the hope of early diagnosis and prevention. 
 BRCA1 expression in invasive breast carcinomas and clinicopathological correlations. BRCA1-positive tumors characterize a heterogeneous group of breast cancers with a specific range of histopathological and immuno-histochemical features. We evaluated the relationship between morphological characteristics and immunohistochemical profile of 14 BRCA1-positive breast tumors from an initial study group of 40 breast tumors. Morphological parameters of all cases were studied on Hematoxylin-Eosin-stained sections and their immunophenotypic profile was assessed using antibodies against estrogen receptors (ER), progesteron receptors (PR), c-erbB2 oncoprotein (Her2/neu), BRCA1-protein, and the proliferative rate of the tumoral cells was assessed by Ki-67-proliferative index. All patients were females with an average age of 43.71-year-old. Fourteen out of the 40 tumors were BRCA1-positive. All breast carcinomas in this study were invasive. The most common histological type in our study was invasive ductal carcinoma. The majority of the tumors were high-grade G3. The examination of the lymph node found   metastasis   in eight cases. We found 11 cases of triple negative (ER, PR and Her2/neu), while the rest showed positivity for all these three markers. We observed a high-celullar proliferation index in all cases. The immunohistochemical study of BRCA1-protein is important to the study of the development and progression of the disease. 
 TNRC9 downregulates BRCA1 expression and promotes breast cancer aggressiveness. Although the linkage between germline mutations of BRCA1 and hereditary breast/ovarian cancers is well established, recent evidence suggests that altered expression of wild-type BRCA1 might contribute to the sporadic forms of breast cancer. The breast cancer gene trinucleotide-repeat-containing 9 (TNRC9; TOX3) has been associated with disease susceptibility but its function is undetermined. Here, we report that TNRC9 is often amplified and overexpressed in breast cancer, particularly in advanced breast cancer. Gene amplification was associated with reduced disease-free and   metastasis  -free survival rates. Ectopic expression of TNRC9 increased breast cancer cell proliferation, migration, and survival after exposure to apoptotic stimuli. These phenotypes were associated with tumor progression in a mouse model of breast cancer. Gene expression profiling, protein analysis, and in silico assays of large datasets of breast and ovarian cancer samples suggested that TNRC9 and BRCA1 expression were inversely correlated. Notably, we found that TNRC9 bound to both the BRCA1 promoter and the cAMP-responsive element-binding protein (CREB) complex, a regulator of BRCA1 transcription. In support of this connection, expression of TNRC9 downregulated expression of BRCA1 by altering the methylation status of its promoter. Our studies unveil a function for TNRC9 in breast cancer that highlights a new paradigm in BRCA1 regulation. 
 [Expression and clinical significance of BRCA1 in human esophageal squamous cell carcinoma]. To investigate the expression of BRCA1 in esophageal squamous cell carcinoma (ESCC) tissues and evaluate its correlation with clinicopathological features as well as the prognosis of ESCC patients.The expression of BRCA1 was detected by immunohistochemistry (IHC) in 201 specimens of T3 stage ESCC tissues and corresponding adjacent normal tissues using tissue microarray. The correlation between BRCA1 expression and clinicopathological features of ESCC was determined by chi-square analysis. The cumulative survival rate was analyzed by Kaplan-Meier method.The positive rate of BRCA1 expression in ESCC tissues was significantly higher than that in adjacent normal tissues [88.6% (178/201) vs. 36.8% (74/201), P < 0.001]. There was a significant correlation between the expression of BRCA1 and lymph node   metastasis  . In the tumors with positive lymph nodes, strong positive expression of BRCA1 was found in 45.0% (49/109), while only 19.6% (18/92) in tumors without lymph node   metastasis  , showing a significant difference (P < 0.001). A close relationship was also found between the expression of BRCA1 and gross typing of tumors (P < 0.05). The expression of BRCA1 was not significantly correlated with gender, age, tumor location, differentiation, and tumor thrombus (P > 0.05). The results of Kaplan-Meier analysis indicated that ESCC patients with a higher positive rate of BRCA1 expression have a poorer prognosis (P < 0.05).The expression of BRCA1 is related to the occurrence and development of esophageal carcinoma. BRCA1 protein may serve as a new potential biomarker in estimating the biological behavior of ESCC. 
 CDC25A, VAV1, TP73, BRCA1 and ZAP70 gene overexpression correlates with radiation response in colorectal cancer. Radiotherapy is increasingly used in adjuvant approaches for colorectal cancer (CRC) to reduce local recurrence and improve survival. However, the principal limitation is the large variability in response among different individuals due to tumor heterogeneity. In the present study, we compared gene expression profiles between radiosensitive and radioresistant colorectal cancer cell lines to identify radiation-related molecules that can be used to evaluate the effects of radiation. The CRC cell line SW620 was irradiated with a high-energy photo beam. Following radiation treatment, RNA was extracted from non-irradiated and irradiated cells, respectively, and gene expression analysis was performed by oligonucleotide microarray and the DAVID bioinformatics method. To further confirm the results, an additional 4 CRC cell lines, COLO205, T84, HCT116, SW480 and SW403 were purchased from ATCC. The radiosensitivities of each were determined by the survival fraction at 2 Gray (SF2) of the surviving cells using the ATPLite assay, and the gene expression profiles after irradiation among the radiosensitive and radioresistant cell lines were analyzed by membrane arrays. The relationships between gene expression and patient clinicopathological features were also analyzed using membrane arrays and RT-PCR. The results from oligonucleotide microarray analysis show that 1601 genes were up-regulated (gene expression ratio of post- to pre-radiation treatment>2). By bioinformatic database analysis, 30 up-regulated genes were identified as involved in DNA damage response pathways, immune response pathways and the complement and coagulation cascades pathway. Fifteen genes showed differential gene expression profiles between radiosensitive (HCT116 and SW620) and radioresistant CRC cell lines (SW403 and SW480). In 110 CRC tissues, we detected five genes CDC25A, VAV1, TP73, BRCA1 and ZAP70 from 15 overexpressed genes that significantly related to prognostic factors (tumor size, advanced stage, invasive depth, lymph node   metastasis   and differentiation). These findings suggest that CDC25A, VAV1, TP73, BRCA1 and ZAP70 may be novel markers for predicting the effectiveness of radiotherapy in CRC patients. 
 BRCA1 mislocalization associated with breast carcinogenesis and poor prognosis in Taiwanese women. We aimed to elucidate whether and how BRCA1 mislocalization [including membranous nuclear (MN) forms and negative patterns] is associated with the occurrence and the prognosis of breast cancer in young Taiwanese women. A case-control study was carried out to enroll 84 patients with breast cancer and 81 patients with benign breast disease. The subcellular localization of BRCA1 was examined using immunofluorescent assays on fresh tissue touch-imprinting slides to classify staining results into diffuse nuclear (DN), MN, and negative staining. Genetic variations of BRCA1 nuclear localization/transportation-related sequences were analyzed by cDNA sequencing of both nuclear localization signals (NLS) and nuclear export signals (NES). The BRCA1 antibody was verified by two other published ones. Comparisons of immunofluorescent assay with immunohistochemical and H&E staining methods were also performed. BRCA1 mislocalization conferred a 3.13-fold [95% confidence interval (CI): 1.31-7.50] risk of developing breast cancer for the MN form and a 5.79-fold (95% CI:1.58-21.23) risk for BRCA1-negative cases compared with DN staining. However, no genetic variant was found in the NES or the NLS region of the BRCA1 gene. In terms of prognosis, BRCA1 mislocalization showed a 3.5-fold (95% CI: 1.20-10.09) increased risk of breast cancer death compared with DN staining after adjusting for tumor node   metastasis   stage. BRCA1 MN forms and BRCA1-negative patterns led to a higher risk of carcinogenesis and a poor prognosis of breast cancer. Such BRCA1 mislocalization may not be directly caused by the genetic effects of the NLS and NES domains, but stem from nongenetic modifications (such as epigenetic silencing). 
 Genetic instability of BRCA1 gene at locus D17S855 is related to clinicopathological behaviors of gastric cancer from Chinese population. To investigate genetic instability of gene BRCA1 at locus D17S855, and their relationship with clinicopathological characteristics of gastric cancer in Chinese population.Microsatellite instability (MSI) and loss of heterozygosity (LOH) of gene BRCA1 at locus D17S855 were compared between 37 samples of gastric cancer and corresponding non-cancerous gastric tissue.MSI at locus D17S855 was positive in 7 of 37 samples of gastric cancer (18.95%). MSI had a close relationship with TNM staging but no relation with lymph node   metastasis  , histological type or tumor differentiation. MSI positive frequency in TNM I + II (31.58%, 6/19) was much higher than that in TNM III + IV (5.56%, 1/18), (P < 0.05). LOH positive rate was 18.92% (7/37). LOH had no relationship to histological type, tumor differentiation or lymph node   metastasis  , but LOH positive rate in TNM III + IV was 33.33% (6/18), much higher than that in TNM I + II ( 5.26%, 1/19), (P < 0.05). BRCA1 protein was expressed in 14 of 37 samples of gastric cancer. The positive rates of BRCA1 protein in TNM I + II and TNM III + IV were 57.89% and 16.67%, respectively, (P < 0.05). The positive rate of BRCA1 protein was 77.78% in high differentiation samples, 30.77% in middle differentiation and 12.50% in lower differentiation samples, (P < 0.05).MSI of BRCA1 gene could be used as a molecular marker in early phases of sporadic gastric cancer in Chinese population. LOH occurs at later period of gastric cancer, therefore, it could be used as prognostic factor. 
 Correlation between BRCA1 expression and apoptosis-related biological parameters in sporadic breast carcinomas. BRCA1 is a tumor suppressor gene that is responsible for hereditary breast and ovarian cancer syndrome. Increased evidence suggests that BRCA1 protein is involved in mammary carcinogenesis in sporadic and hereditary forms. Recent experimental results suggest that BRCA1 plays a role in the regulation of apoptosis. In order to test whether the analysis of human tumors would provide data supporting this hypothesis in sporadic breast carcinomas, we have investigated the relationship between BRCA1 and apoptosis-related genes. Immunohistochemical analysis was performed to determine BRCA1 and the apoptosis-related proteins bcl-2, Bax and p53 in paraffin-embedded tissues of 156 sporadic invasive ductal carcinomas. BRCA1 expression was positively-correlated with Bcl-2 expression (p = 0.0008), but no relationship between BRCA1 expression and Bax or p53 expression could be established. In addition, loss of BRCA1 expression was also related to poor tumor differentiation and lymph node   metastasis  . Our study shows that bcl-2 might be one of the target genes involved in the oncogenesis related to BRCA1. Loss of BRCA1 may contribute to tumor development in breast carcinomas, which may be independent of the p53 tumor suppressor. 
 Osteopontin can act as an effector for a germline mutation of BRCA1 in malignant transformation of breast cancer-related cells. Breast cancer-associated 1 (BRCA1) plays an important role in breast cancer initiation and progression through its functions in the cell cycle and DNA repair processes; however, its role in metastatic development in human breast cancer is still poorly understood. We have previously shown that osteopontin (OPN) expression was suppressed by wild-type BRCA1 (Wt.BRCA1) and that a natural mutant allele of BRCA1 (Mut.BRCA1) diminished the effect of Wt.BRCA1 on OPN in vitro. In this study, we show that while Wt.BRCA1 suppresses OPN-induced   metastasis   in a rat syngeneic system, Mut.BRCA1 enhances the development of   metastasis   through OPN, suggesting that OPN and BRCA1 work closely to regulate metastatic development in the rat. To test whether these findings are relevant to human breast cancer, we have investigated the relationship between BRCA1, OPN, and metastatic properties in human breast cancer-related cells. Using western blot analysis, we show that Wt.BRCA1 suppresses, while Mut.BRCA1 enhances, OPN protein expression; and in parallel that Wt.BRCA1 suppresses, while Mut.BRCA1 enhances, OPN-mediated in vitro properties associated with the metastatic state in both MCF-7 and MDA MB435s cells. Overall, these results suggest that Mut.BRCA1 can elicit some of the changes involved in metastatic progression in human breast cancer via the overexpression of OPN. 
 [Genetic instability on chromosome 17q21 in gastric cancer of Chinese patients]. To investigate microsatellite instability (MSI) and loss of heterozygosity (LOH) of locus D17S396, D17S579 and D17S855, and their effect on the expression of nm23H1 and BRCA1 of gastric cancer, which would provide experimental basis for clinical treatment and prognosis analysis of gastric cancer. DNA was extracted from paraffin-embedded materials. Polymerase chain reaction-single strand conformation polymorphism (PCR-SSCP) was used to analyze MSI and LOH. Expression of nm23H, and BRCA1 was detected by Envision immuno-histochemistry and Leica-Qwin computer imaging techniques. In the forty cases of gastric cancer, the frequency of MSI, LOH and nm23H1 protein were 20.00%, 17.50% and 55.00% respectively at locus D17S396, while at locus D17S579, the frequency of MSI, LOH and BRCA1 protein were 22.50%, 15.00% and 37.50% respectively; at locus D17S855, the frequency of MSI, LOH and BRCA1 of thirty-seven cases were 18.92%, 18.92%, 37.84% respectively. In tumor node   metastasis   (TNM) staging, at locus D17S396, D17S579 and D17S855, MSI in stages I + II appeared more frequently than that in stages III + IV, while LOH appeared the contrary tendency. In the group of   metastasis   of gastric cancer, MSI had a less frequency (5.00%) than that with no   metastasis   (35.00%, P < 0.05) at locus D17S396, but LOH appeared more frequently (30.00%) than that with no   metastasis   (5.00%, P < 0.05). At locus D17S579, MSI had an increasing tendency with the degree of tumor differentiation (50.00% in high differentiation cases, 20.00% in middle differentiation cases, and 0% in low differentiation cases, P < 0.05). The frequency of nm23H1 and BRCA1 protein in stages TNM I + II was higher than that in stages TNM III + IV; and that in higher differentiation cases was higher than in poor differentiation cases. The frequency of nm23H1 protein in the group of   metastasis   (30.00%) was less than that with no   metastasis   significantly (80.00%, P<0.01). The frequency of nm23H1 protein in the group positive to MSI (87.50%) was higher than that in the group negative to MSI (46.88%, P < 0.05). However, nm23H1 protein in group positive to LOH (14.29%) was lower than that in the group negative to LOH (63.64%, P < 0.05). The frequency of BRCA1 protein in the group positive to MSI (66.67%) was more than that in the group negative to MSI (29.03%, P < 0.05). The results of experiments indicate that MSI and LOH may separately control the development of sporadic colon cancer with different pathways. MSI may be an early period molecule marker for sporadic colon cancer, enhanced expression of nm23H1 protein can effectively inhibit colon cancer   metastasis   and improve prognosis of sporadic colon cancer patients. By comparison, LOH mostly arises in the late period of sporadic colon cancer and endows a high aggressive and poor prognostic phenotype. nm23H1 protein could effectively restrain gastric cancer   metastasis   and development; and BRCA1 protein could restain tumor from becoming lower differentiation. 
 Comparison of nodal   metastasis   between BRCA mutation carriers and non-BRCA mutation carriers with breast cancer. This study evaluates whether nodal status differs between breast cancer patients with BRCA mutations and those confirmed not to harbor mutations.A prospective database identified patients with breast cancer who underwent genetic testing and axillary staging. Comparative variables included age, as well as tumor characteristics such as size, grade, lymphovascular invasion (LVI), estrogen receptor, progesterone receptor, human epidermal growth factor receptor 2 (HER2-neu), and nodal status.Overall, 235 patients with breast cancer underwent genetic testing for BRCA mutations from June 2000 to May 2012. Of these patients, 74 (31.4 %) were found to express BRCA 1 and/or 2 mutations, and 161 (68.5 %) patients were verified to have no detectable BRCA mutation. Among the entire 235 patients tested, 92 (39.1 %) were found to have nodal disease. In univariable analysis, only LVI and tumor size correlated with presence of nodal   metastasis  . Of the 74 BRCA mutation carriers, 34 (45.9 %) had nodal   metastasis   compared with 58 of the 161 (36 %; p = 0.15) patients without a BRCA mutation. BRCA mutation carriers with nodal disease were more likely to have poorly differentiated tumors than those without mutations who had nodal disease (24/33 [72.7 %] vs. 27/57 [47.4 %]; p = 0.027).BRCA mutations are not themselves predictive of nodal   metastasis  . Patients with BRCA mutations did not have a statistically significant higher prevalence of nodal   metastasis   than those without mutations. 
 Germline BRCA mutations are associated with higher risk of nodal involvement, distant   metastasis  , and poor survival outcomes in prostate cancer. To analyze the baseline clinicopathologic characteristics of prostate tumors with germline BRCA1 and BRCA2 (BRCA1/2) mutations and the prognostic value of those mutations on prostate cancer (PCa) outcomes.This study analyzed the tumor features and outcomes of 2,019 patients with PCa (18 BRCA1 carriers, 61 BRCA2 carriers, and 1,940 noncarriers). The Kaplan-Meier method and Cox regression analysis were used to evaluate the associations between BRCA1/2 status and other PCa prognostic factors with overall survival (OS), cause-specific OS (CSS), CSS in localized PCa (CSS_M0),   metastasis  -free survival (MFS), and CSS from   metastasis   (CSS_M1).PCa with germline BRCA1/2 mutations were more frequently associated with Gleason ≥ 8 (P = .00003), T3/T4 stage (P = .003), nodal involvement (P = .00005), and metastases at diagnosis (P = .005) than PCa in noncarriers. CSS was significantly longer in noncarriers than in carriers (15.7 v 8.6 years, multivariable analyses [MVA] P = .015; hazard ratio [HR] = 1.8). For localized PCa, 5-year CSS and MFS were significantly higher in noncarriers (96% v 82%; MVA P = .01; HR = 2.6%; and 93% v 77%; MVA P = .009; HR = 2.7, respectively). Subgroup analyses confirmed the poor outcomes in BRCA2 patients, whereas the role of BRCA1 was not well defined due to the limited size and follow-up in this subgroup.Our results confirm that BRCA1/2 mutations confer a more aggressive PCa phenotype with a higher probability of nodal involvement and distan  t metastas  is. BRCA mutations are associated with poor survival outcomes and this should be considered for tailoring clinical management of these patients. 
 The ectopic expression of BRCA1 is associated with genesis, progression, and prognosis of breast cancer in young patients. The study is to explore the histopathological features and the molecular marker expression of young women with breast cancers.The pathological data of 367 cases of female breast cancer patients were retrospectively analyzed, focusing on the analysis of young breast cancer incidence trends and the clinical and pathological features.Compared with elderly breast cancer patients, young women with breast cancers had larger tumor sizes, higher histological grades, and lymph node   metastasis   rates. The majority of patients were in the PTNM III stage, with the clinical and pathological features of strong invasiveness. The positive expression rate of the BRCA1 protein in the young group was higher than that in the old group. BRCA1 expression was positively correlated with the PTNM stage and axillary lymph node   metastasis   (P < 0.05).The ectopic expression of BRCA1 is associated with the genesis, progression, and prognosis of young breast cancer patients.The virtual slide(s) for this article can be found here: http://www.diagnosticpathology.diagnomx.eu/vs/1628000054838044. 
 Coexpression of the mutated BRCA1 mRNA and p53 mRNA and its association in Chinese prostate cancer. The mutations of BRCA1 and p53 genes have been simultaneously characterized in many tumors. However, their coexpression and associations have not been investigated quantitatively in prostate cancer. In the present study, the expressions of the mutated BRCA1 mRNA and p53 mRNA were examined in 48 Chinese prostate cancer cases and 10 corresponding adjacent benign tissues with in situ hybridization. The 5-year survival rates of the corresponding patients after operation were investigated. The results showed that the positive expressions of the mutated BCRA1 mRNA and p53 mRNA are involved in prostate cancer (P < 0.05). Moreover, there is a closed negative association between the expressions of the mutated BRCA1 gene and p53 gene in the mRNA level with the progression, angiogenesis,   metastasis  , and survival rate of prostate cancer. Their coexpression and negative association suggest that the two altered tumor suppressor genes might interact functionally in prostate cancer to provide a potential signal determining a prognosis of the tumor   metastasis   and survival rate. Further work will be done to elucidate the interaction mechanisms in prostate cancer. 
 Clinicopathological features and prognostic factors of young breast cancers in Eastern Guangdong of China. Breast cancer in young women is typically with higher proportion of adverse pathological features. Breast cancer with BRCA1 mutation is often early-onset, and is usually associated with triple negative phenotpe. In this study, we aim to analyze the clinicopathological characteristics and prognosis in young breast cancer patients (≤35 years old) comparing to non-young patients (>35 years old). A total of 1913 cases of primary breast carcinoma with stage I-III were enrolled, with 283 cases diagnosed as young patients. No significant difference was observed in tumor size, TNM staging, lymph nod  e metastas  is, ER, HER-2 or histological grade between young and non-young patients. Multivariate analysis demonstrated that age was an independent prognostic factor for overall survival (OS). In 70 samples of young patients available, BRCA1 was immunohistochemically positive 85.7% in cytoplasm and 41.4% in nuclear. BRCA1 nuclear expression is not significantly associated with clinicopathological characteristics in young breast cancer patients. 
 Clinical implications for BRCA gene mutation in breast cancer. To investigate the mutations of BRCA1 and BRCA2 and determine whether clinic-pathological factors related to BRCA gene mutation. Mastectomy specimens from 360 breast cancers were enrolled and examined in the study. The relationship between BRCA gene mutation and clinic-pathological factors was evaluated. Overall, 280 patients were BRCA negative and 80 got BRCA gene mutation. Triple-negative breast cancers--i.e., breast cancers that do not express estrogen receptors (ER), progesterone receptors (PR) or human epidermal growth factor receptor 2 (HER2/neu)--was observed in 53.85% of the BRCA1 mutation patients, in 28.57% of the BRCA2 mutation cases, while 14.29% of BRCA negative patients. BRCA1 mutation patients got a heavy lymph node   metastasis   and higher nuclear grade tumors than the others (P = 0.004, 0.007). Furthermore, BRCA mutation was also found to be significantly related to ER, PR and HER2/neu status (P < 0.05). BRCA1 expression was not associated with breast cancer-specific survival in the triple-negative breast cancers (P = 0.742). After Cox regression, BRCA1 mutation was not shown to be an independent prognostic factor for breast cancer. These findings substantiated the possibility of tumors associated with BRCA1 mutations divided into two distinct groups, triple-negative and non-triple-negative groups requires further investigation. 
 BRCA1 suppresses epithelial-to-mesenchymal transition and stem cell dedifferentiation during mammary and tumor development. BRCA1 mutation carriers are predisposed to developing basal-like breast cancers with high   metastasis   and poor prognosis. Yet, how BRCA1 suppresses formation of basal-like breast cancers is still obscure. Deletion of p18(Ink4c) (p18), an inhibitor of CDK4 and CDK6, functionally inactivates the RB pathway, stimulates mammary luminal stem cell (LSC) proliferation, and leads to spontaneous luminal tumor development. Alternately, germline mutation of Brca1 shifts the fate of luminal cells to cause luminal-to-basal mammary tumor transformation. Here, we report that disrupting Brca1 by either germline or epithelium-specific mutation in p18-deficient mice activates epithelial-to-mesenchymal transition (EMT) and induces dedifferentiation of LSCs, which associate closely with expansion of basal and cancer stem cells and formation of basal-like tumors. Mechanistically, BRCA1 bound to the TWIST promoter, suppressing its activity and inhibiting EMT in mammary tumor cells. In human luminal cancer cells, BRCA1 silencing was sufficient to activate TWIST and EMT and increase tumor formation. In parallel, TWIST expression and EMT features correlated inversely with BRCA1 expression in human breast cancers. Together, our findings showed that BRCA1 suppressed TWIST and EMT, inhibited LSC dedifferentiation, and repressed expansion of basal stem cells and basal-like tumors. Thus, our work offers the first genetic evidence that Brca1 directly suppresses EMT and LSC dedifferentiation during breast tumorigenesis. 
  GeneRIFs  
 A model where BRCA1 cytosolic mislocalisation promotes breast cancer   metastasis  , making it a potential biomarker of metastatic disease. 
 BRCA1 mutation is not associated with nodal   metastasis   in breast cancer. 
 Data indicate that patients negative for BRCA1 protein were more often with pathological tumor-node-  metastasis   stage III, positive for lymph node   metastasis   and MYC overexpression than BRCA1-positive tumors. 
 ERCC1 and BRCA1 mRNA expression levels correlate inversely to CRC   metastasis  . ERCC1 and BRCA1 might serve as biomarkers for lymphatic   metastasis   and as prognostic indicators for CRC; their down-expressions are predictors of poor outcome in CRC patients 
 BRCA1/2 germ-line mutations confer a more aggressive prostate cancer phenotype with a higher probability of nodal involvement, distant   metastasis   and poor survival. 
 nm23H1 protein could effectively restrain gastric cancer   metastasis   and development; and BRCA1 protein could restain tumor from becoming lower differentiation. 
 Studied BRCA1 and BRCA2 exp'n in young breast cancer patients;BRCA1 & BRCA2 expression correlated in healthy, but not in tumor tissues. Neither BRCA1 nor BRCA2 exp'n was assoc'd with tumor histology, diff'n, nodal   metastasis   or p53 and HER-2 exp'n. 
 BRCA1 methylation correlated with age at diagnosis (P = .015) and 5-years disease free survival (P = .016) while hMLH1 methylation was more frequent in larger tumors (P = .002) and in presence of distant   metastasis   (P = .004). 
 The site of first distant   metastasis   is different between BRCA1- and BRCA2-associated and sporadic breast cancer patients. 
  [Cc]ell [Ii]nvasion  PubMed  
 Association between cytosolic expression of BRCA1 and metastatic risk in breast cancer. Although BRCA1 has been extensively studied for its role as a tumour-suppressor protein, the role of BRCA1 subcellular localisation in oncogenesis and tumour progression has remained unclear. This study explores the impact of BRCA1 mislocalisation on clinical outcomes in breast cancer.Tissue microarrays assembled from a cohort of patients with all stages of breast cancer were analysed for BRCA1 localisation and correlated with patient survival. Tissue microarrays of patients who had breast cancer that had metastasised to the lung were assembled from an independent cohort of patients. These were analysed for BRCA1 subcellular expression. In vitro studies using cultured human breast cancer cells were conducted to examine the effect of cytosolic BRCA1 on cell migration and efficiency of invasion.An inverse association was found between cytosolic BRCA1 expression and metastasis-free survival in patients aged >40 years. Further analysis of BRCA1 subcellular expression in a cohort of breast cancer patients with metastatic disease revealed that the cytosolic BRCA1 content of breast tumours that had metastasised to the lung was 36.0% (95% CI=(31.7%, 40.3%), which was markedly higher than what is reported in the literature (8.2-14.8%). Intriguingly, these lung metastases and their corresponding primary breast tumours demonstrated similarly high cytosolic BRCA1 distributions in both paired and unpaired analyses. Finally, in vitro studies using human breast cancer cells demonstrated that genetically induced BRCA1 cytosolic sequestration (achieved using the cytosol-sequestering BRCA1 5382insC mutation) increased   cell invasion   efficiency.Results from this study suggest a model where BRCA1 cytosolic mislocalisation promotes breast cancer metastasis, making it a potential biomarker of metastatic disease. 
   
  
 SMYD1 
 
  NCBI Gene  
  GeneRIF  
  Pubmed  
 
 
  Search Term  Rank  Fields  
  [Mm]igration  6  PubMed(1); GeneRIFs(1)  
 
 
 hide/show details for SMYD1 
  [Mm]igration  PubMed  
 SMYD1, an SRF-Interacting Partner, Is Involved in Angiogenesis. Previous studies have demonstrated that Smyd1 plays a critical role in cardiomyocyte differentiation, cardiac morphogenesis and myofibril organization. In this study, we uncovered a novel function of Smyd1 in the regulation of endothelial cells (ECs). Our data showed that Smyd1 is expressed in vascular endothelial cells, and knockdown of SMYD1 in endothelial cells impairs EC   migration   and tube formation. Furthermore, Co-IP and GST pull-down assays demonstrated that SMYD1 is associated with the Serum Response Factor (SRF). EMSA assays further showed that SMYD1 forms a complex with SRF and enhances SRF DNA binding activity. Our studies indicate that SMYD1 serves as an SRF-interacting protein, enhances SRF DNA binding activity, and is required for EC   migration   and tube formation to regulate angiogenesis. 
  GeneRIFs  
 SMYD1 serves as an Serum Response Factor-interacting protein, enhances Serum Response Factor DNA binding activity, and is required for endothelial cell   migration   and tube formation to regulate angiogenesis. 
   
  
 SMYD2 
 
  NCBI Gene  
  GeneRIF  
  Pubmed  
 
 
  Search Term  Rank  Fields  
  [Mm]igration  6  PubMed(1)  
  [Mm]etastasis  12  PubMed(2)  
 
 
 hide/show details for SMYD2 
  [Mm]igration  PubMed  
 Overexpression of SMYD2 contributes to malignant outcome in gastric cancer. SET and MYND domain-containing protein 2 (SMYD2) is a lysine methyltransferase for histone H3, p53 and Rb and inhibits their transactivation activities. In this study, we tested whether SMYD2 (1q42) acts as a cancer-promoting factor by being overexpressed in gastric cancer.We analysed 7 gastric cancer cell lines and 147 primary tumor samples of gastric cancer, which were curatively resected in our hospital.SET and MYND domain-containing protein 2 was detected in these cell lines (five out of seven cell lines; 71.4%) and primary tumor samples (fifty-six out of one hundred and forty-seven cases; 38.1%). Knockdown of SMYD2 using specific small interfering RNA inhibited proliferation,   migration   and invasion of SMYD2-overexpressing cells in a TP53 mutation-independent manner. Overexpression of SMYD2 protein correlated with larger tumor size, more aggressive lymphatic invasion, deeper tumor invasion and higher rates of lymph node metastasis and recurrence. Patients with SMYD2-overexpressing tumours had a worse overall rate of survival than those with non-expressing tumours (P=0.0073, log-rank test) in an intensity and proportion score-dependent manner. Moreover, multivariate analysis demonstrated that SMYD2 was independently associated with worse outcome (P=0.0021, hazard ratio 4.25 (1.69-10.7)).These findings suggest that SMYD2 has a crucial role in tumor cell proliferation by its overexpression and highlight its usefulness as a prognostic factor and potential therapeutic target in gastric cancer. 
  [Mm]etastasis  PubMed  
 JTE-522, a selective COX-2 inhibitor, inhibits growth of pulmonary metastases of colorectal cancer in rats. Epidemiological studies have shown that individuals who regularly consume NSAIDs have lower rates of mortality associated with colorectal cancer. Because COX-2 inhibitors prevent tumor growth through some mechanisms, we assessed the effect of JTE-522, a selective COX-2 inhibitor, on pulmonary metastases of colon cancer in a rat model.A suspension of 5 x 10(6) RCN-9 (rat colon cancer cells) was injected into the tail vein of 24 anesthetized male F344/DuCrj rats. Oral JTE-522 (0, 3, 10, or 30 mg/kg/day) was administered from the day before RCN-9 injection until the end of the study. Twenty-four days later, the lungs were removed from sacrificed rats and weighed. Pulmonary metastatic tumors were microscopically evaluated in the largest cross sections. We also performed immunohistochemical staining for both COX-2 and VEGF.JTE-522 dose-dependently decreased lung weight (p = 0.001) and the size of pulmonary metastatic tumors (p = 0.0002). However, the differences in the number of metastatic tumors among 4 groups were insignificant. Significant adverse effects of JTE-522 were undetectable. Immunohistochemical staining showed high levels of both COX-2 and VEGF in pulmonary metastatic tumors.JTE-522 dose-dependently decreased the size, but not the number of pulmonary metastases. COX-2 inhibitors might block metastatic tumor growth, but not actual   metastasis  . Selective COX-2 inhibitors might be useful as therapeutic agents that inhibit the growth of metastatic tumors, as well as the tumorigenesis of colorectal cancer. 
 Overexpression of SMYD2 contributes to malignant outcome in gastric cancer. SET and MYND domain-containing protein 2 (SMYD2) is a lysine methyltransferase for histone H3, p53 and Rb and inhibits their transactivation activities. In this study, we tested whether SMYD2 (1q42) acts as a cancer-promoting factor by being overexpressed in gastric cancer.We analysed 7 gastric cancer cell lines and 147 primary tumor samples of gastric cancer, which were curatively resected in our hospital.SET and MYND domain-containing protein 2 was detected in these cell lines (five out of seven cell lines; 71.4%) and primary tumor samples (fifty-six out of one hundred and forty-seven cases; 38.1%). Knockdown of SMYD2 using specific small interfering RNA inhibited proliferation, migration and invasion of SMYD2-overexpressing cells in a TP53 mutation-independent manner. Overexpression of SMYD2 protein correlated with larger tumor size, more aggressive lymphatic invasion, deeper tumor invasion and higher rates of lymph node   metastasis   and recurrence. Patients with SMYD2-overexpressing tumours had a worse overall rate of survival than those with non-expressing tumours (P=0.0073, log-rank test) in an intensity and proportion score-dependent manner. Moreover, multivariate analysis demonstrated that SMYD2 was independently associated with worse outcome (P=0.0021, hazard ratio 4.25 (1.69-10.7)).These findings suggest that SMYD2 has a crucial role in tumor cell proliferation by its overexpression and highlight its usefulness as a prognostic factor and potential therapeutic target in gastric cancer. 
   
  
 SMYD3 
 
  NCBI Gene  
  GeneRIF  
  Pubmed  
 
 
  Search Term  Rank  Fields  
  [Mm]igration  6  PubMed(9); GeneRIFs(5)  
  [Mm]etastasis  12  PubMed(8); GeneRIFs(2)  
 
 
 hide/show details for SMYD3 
  [Mm]igration  PubMed  
 Histone methyltransferase SMYD3 promotes MRTF-A-mediated transactivation of MYL9 and   migration   of MCF-7 breast cancer cells. Myocardin-related transcription factor-A (MRTF-A) is a Rho signal-responsive transcriptional coactivator of serum response factor (SRF). Recent studies indicated that MRTF-A might be an important regulator of mammary gland and be involved in cancer metastasis. However, the roles of histone modification in the MRTF-A-dependent signal pathway and tumor   migration   are still not very clear. Here, we report that histone methylation is required for the MRTF-A-mediated upregulation of myosin regulatory light chain 9 (MYL9), an important cytoskeletal component which is implicated in cell   migration  . Furthermore, we demonstrate that SET and MYND domain containing protein 3 (SMYD3), a hitone methyltransferase (HMT) associated with carcinogenesis, might be the one which is responsible for the histone methylation occurred in the MRTF-A-mediated- transactivation of MYL9 and   migration   of breast cancer cells. Overexpression of SMYD3 promotes MRTF-A-mediated upregulation of MYL9 and   migration   of MCF-7 breast cancer cells, while contrary results were observed when the endogenous MRTF-A and SMYD3 were suppressed with specific siRNAs. In addition, the mutation analysis suggested that this cooperative transactivation is mainly mediated via the proximal binding element of MRTF-A in the promoter of MYL9, and the HMT activity of SMYD3 is required as well. Our findings reveal a new mechanism by which MRTF-A and SMYD3 functions in transcriptional regulation and cell   migration  , and provide a better understanding for metastasis of breast cancer. 
 Histone methyltransferase SMYD3 promotes MRTF-A-mediated transactivation of MYL9 and   migration   of MCF-7 breast cancer cells. Myocardin-related transcription factor-A (MRTF-A) is a Rho signal-responsive transcriptional coactivator of serum response factor (SRF). Recent studies indicated that MRTF-A might be an important regulator of mammary gland and be involved in cancer metastasis. However, the roles of histone modification in the MRTF-A-dependent signal pathway and tumor   migration   are still not very clear. Here, we report that histone methylation is required for the MRTF-A-mediated upregulation of myosin regulatory light chain 9 (MYL9), an important cytoskeletal component which is implicated in cell   migration  . Furthermore, we demonstrate that SET and MYND domain containing protein 3 (SMYD3), a hitone methyltransferase (HMT) associated with carcinogenesis, might be the one which is responsible for the histone methylation occurred in the MRTF-A-mediated- transactivation of MYL9 and   migration   of breast cancer cells. Overexpression of SMYD3 promotes MRTF-A-mediated upregulation of MYL9 and   migration   of MCF-7 breast cancer cells, while contrary results were observed when the endogenous MRTF-A and SMYD3 were suppressed with specific siRNAs. In addition, the mutation analysis suggested that this cooperative transactivation is mainly mediated via the proximal binding element of MRTF-A in the promoter of MYL9, and the HMT activity of SMYD3 is required as well. Our findings reveal a new mechanism by which MRTF-A and SMYD3 functions in transcriptional regulation and cell   migration  , and provide a better understanding for metastasis of breast cancer. 
 Epigenetic regulation of miR-124 by hepatitis C virus core protein promotes   migration   and invasion of intrahepatic cholangiocarcinoma cells by targeting SMYD3. Hepatitis C Virus core protein (HCVc) plays important roles in the development of intrahepatic cholangiocarcinoma (ICC). MicroRNAs (miRNAs) contribute to tumor progression by interacting with downstream target genes. However, the regulation and role of miRNAs in HCV-related intrahepatic cholangiocarcinoma (HCV-ICC) is poorly understood. In this study, we found that miR-124 was down-regulated in HCV-ICC and the induction of DNMT1 by HCVc mediated the suppression of miR-124. Over-expression of miR-124 suppressed cell   migration   and invasion in vitro, and reduced the protein levels of SMYD3 and downstream target genes (c-Myc and MMP9). Knockdown of SMYD3 inhibited cell   migration   and invasion resembling that of miR-124 over-expression. In conclusion, our studies indicate that low miR-124 levels mediated by HCVc via DNMT1 promote ICC cell   migration   and invasion by targeting SMYD3. 
 Knockdown of SMYD3 by RNA interference down-regulates c-Met expression and inhibits cells   migration   and invasion induced by HGF. We previously reported that over-expression of SMYD3, a histone H3-K4 specific di- and tri-methyltransferase, plays a key role in cell viability, adhesion,   migration   and invasion. In this study, we investigated the mechanisms underlying these phenomena and found that knocking down SMYD3 expression in tumor cells significantly reduced the biological function of HGF and inhibited carcinoma cells   migration   and invasion. Due to the fact that the proto-oncogene c-Met encodes the high-affinity receptor for HGF, and the HGF-c-Met signaling plays a critical role in the tumor genesis, we further identified the partial correlation between SMYD3 and c-Met. The results showed that high expression of c-Met accompanied with over-expression of SMYD3. Silencing SMYD3 expression in tumor cells by specific shRNAs down-regulated c-Met gene transcription, while over-expressing SMYD3 induced c-Met transcription. Moreover, we demonstrated here that two SMYD3 binding sites within the c-Met core promoter region were significant in the transactivation of c-Met. The present findings provide significant insights into the epigenetic regulatory mechanisms of oncogene c-Met expression, and develop the strategies that may inhibit the progression of cancer   migration   and invasion. 
 SMYD3 promotes cancer invasion by epigenetic upregulation of the metalloproteinase MMP-9. Upregulation of the matrix metalloproteinase (MMP)-9 plays a central role in tumor progression and metastasis by stimulating cell   migration  , tumor invasion, and angiogenesis. To gain insights into MMP-9 expression, we investigated its epigenetic control in a reversible model of cancer that is initiated by infection with intracellular Theileria parasites. Gene induction by parasite infection was associated with trimethylation of histone H3K4 (H3K4me3) at the MMP-9 promoter. Notably, we found that the H3K4 methyltransferase SMYD3 was the only histone methyltransferase upregulated upon infection. SMYD3 is overexpressed in many types of cancer cells, but its contributions to malignant pathophysiology are unclear. We found that overexpression of SMYD3 was sufficient to induce MMP-9 expression in transformed leukocytes and fibrosarcoma cells and that proinflammatory phorbol esters further enhanced this effect. Furthermore, SMYD3 was sufficient to increase cell   migration   associated with MMP-9 expression. In contrast, RNA interference-mediated knockdown of SMYD3 decreased H3K4me3 modification of the MMP-9 promoter, reduced MMP-9 expression, and reduced tumor cell proliferation. Furthermore, SMYD3 knockdown also reduced cellular invasion in a zebrafish xenograft model of cancer. Together, our results define SMYD3 as an important new regulator of MMP-9 transcription, and they provide a molecular link between SMYD3 overexpression and metastatic cancer progression. 
 Knockdown of SMYD3 by RNA interference inhibits cervical carcinoma cell growth and invasion in vitro. Elevated expression of SMYD3 is a frequent genetic abnormality in several malignancies. Few studies knocking down SMYD3 expression in cervical carcinoma cells have been performed to date. In this paper, we established an inducible short hairpin RNA expression system to examine its role in maintaining the malignant phenotype of HeLa cells. After being induced by doxycycline, SMYD3 mRNA and protein expression were both reduced, and significant reductions in cell proliferation, colony formation and   migration  /invasion activity were observed in the SMYD3-silenced HeLa cells. The percentage of cells in sub-G1 was elevated and DNA ladder formation could be detected, indicating potent induction of apoptosis by SMYD3 knockdown. These findings imply that SMYD3 plays crucial roles in HeLa cell proliferation and   migration  /invasion, and that it may be a useful therapeutic target in human cervical carcinomas. 
 Silencing SMYD3 in hepatoma demethylates RIZI promoter induces apoptosis and inhibits cell proliferation and   migration  . To investigate the role of SMYD3 in hepatocellular carcinoma (HCC) development and progression and to verify whether its regulation activity was through RIZ1 inactivation.Expression of SMYD3 in HCC cell lines and tissues were measured; silencing of SMYD3 by RNA interference (RNAi) was effectuated, hepatoma cell proliferation,   migration   and apoptosis were tested, with RIZ1 CpG promoter methylation, and corresponding mRNA expression were investigated.SMYD3 over-expression in HCC was associated with RIZ1 hypermethylation and mRNA down-expression. Suppression of SMYD3 expression de-methylated RIZ1 CpG promoter (P < 0.01) and increased RIZ1 mRNA expression (P < 0.01). Consequently, SMYD3 down-expression with RIZ1 de-methylation strongly inhibited hepatoma cell growth (MTT inhibitory rates: Pgenesil-1-s1 60.95% +/- 7.97%, Pgenesil-1-s2 72.14% +/- 9.68% vs Pgenesil-1-hk 6.89% +/- 4.12%, P < 0.01) and   migration   (Pgenesil-1-s1 4.24% +/- 1.58%, Pgenesil-1-s1 4.87% +/- 0.73% vs Pgenesil-1 19.03% +/- 4.63%, Pgenesil-1-hk 19.95% +/- 5.21%, P < 0.01) and induced apoptosis (FCM subG1 phase Pgenesil-1-s1 19.07% +/- 1.78%, Pgenesil-1-s2 17.68% +/- 2.36% vs Pgenesil-1 0.47% +/- 0.12%, Pgenesil-1-hk 1.46% +/- 0.28%, P < 0.01. TUNEL-positive cells: Pgenesil-1-s1 40.24% +/- 5.18%, Pgenesil-1-s2 38.48% +/- 4.65% vs Pgenesil-1 2.18% +/- 1.34%, Pgenesil-1-hk 2.84% +/- 1.22%, P < 0.01) in HepG2 cells.These results demonstrate that SMYD3 plays a critical role in the carcinogenesis and progression of HCC. The proliferation,   migration   induction and apoptosis inhibition activities of SMYD3 may be mediated through RIZ1 CpG promoter hypermethylation. 
 SMYD3 as an oncogenic driver in prostate cancer by stimulation of androgen receptor transcription. Androgen receptor (AR) is critical for prostate tumorigenesis and is frequently overexpressed during prostate cancer (PC) progression. However, few studies have addressed the epigenetic regulation of AR expression.We analyzed SMYD3 expression in human PC with Western blot and immunohistochemistry. SMYD3 expression was knocked down using short hairpin RNA (shRNA) or small interfering RNA (siRNA). Cell proliferation, colony formation, and apoptosis analyses and xenograft transplantation were performed to evaluate the impact of SMYD3 depletion on PC cells. AR expression and promoter activity were determined using real-time quantitative polymerase chain reaction, western blot, and luciferase reporter assay. AR promoter association with Sp1, SMYD3, and histone modifications was assessed by chromatin immunoprecipitation. Differences in AR mRNA abundance and promoter activity were analyzed using Wilcoxon signed-rank tests, SMYD3 expression was analyzed using with Mann-Whitney U tests for unpaired samples, and tumor weight was analyzed with Student t test. All statistical tests were two-sided.The upregulation of SMYD3 protein expression was observed in seven of eight prostate tumor specimens, compared with matched normal tissues. Immunohistochemical analysis showed a strong SMYD3 staining in the nuclei of PC tissues in eight of 25 (32%) cases and in the cytoplasm in 23 out of 25 (92%) cases, whereas benign prostate tissue exhibited weak immunostaining. Depletion of SMYD3 by siRNA or shRNA inhibited PC cell proliferation (72 hours relative to 24 hours: control shRNA vs SMYD3 shRNA 1: mean fold change = 2.76 vs 1.68; difference = 1.08; 95% confidence interval = 0.78 to 1.38, P < .001), colony formation, cell   migration  , invasion, and xenograft tumor formation. Two functional SMYD3-binding motifs were identified in the AR promoter region.SMYD3 promotes prostate tumorigenesis and mediates epigenetic upregulation of AR expression. 
 SMYD3 contributes to a more aggressive phenotype of prostate cancer and targets Cyclin D2 through H4K20me3. Prostate cancer (PCa) is one of the most incident cancers worldwide but clinical and pathological parameters have limited ability to discriminate between clinically significant and indolent PCa. Altered expression of histone methyltransferases and histone methylation patterns are involved in prostate carcinogenesis. SMYD3 transcript levels have prognostic value and discriminate among PCa with different clinical aggressiveness, so we decided to investigate its putative oncogenic role on PCa.We silenced SMYD3 and assess its impact through in vitro (cell viability, cell cycle, apoptosis,   migration  , invasion assays) and in vivo (tumor formation, angiogenesis). We evaluated SET domain's impact in PCa cells' phenotype. Histone marks deposition on SMYD3 putative target genes was assessed by ChIP analysis.Knockdown of SMYD3 attenuated malignant phenotype of LNCaP and PC3 cell lines. Deletions affecting the SET domain showed phenotypic impact similar to SMYD3 silencing, suggesting that tumorigenic effect is mediated through its histone methyltransferase activity. Moreover, CCND2 was identified as a putative target gene for SMYD3 transcriptional regulation, through trimethylation of H4K20.Our results support a proto-oncogenic role for SMYD3 in prostate carcinogenesis, mainly due to its methyltransferase enzymatic activity. Thus, SMYD3 overexpression is a potential biomarker for clinically aggressive disease and an attractive therapeutic target in PCa. 
  GeneRIFs  
 Overexpression of SMYD3 promotes MRTF-A-mediated upregulation of MYL9 and   migration   of MCF-7 breast cancer cells 
 low miR-124 levels mediated by HCV core protein via DNMT1 promote intrahepatic cholangiocarcinoma cell   migration   and invasion by targeting SMYD3 
 SMYD3 has a role in regulation of c-Met expression and in cell   migration   and invasion induced by HGF 
 SMYD3 plays crucial roles in HeLa cell proliferation and   migration  /invasion, and that it may be a useful therapeutic target in human cervical carcinomas. 
 The proliferation,   migration   induction and apoptosis inhibition activities of SMYD3 in hepatocellular carcinoma may be mediated through RIZ1 CpG promoter hypermethylation. 
  [Mm]etastasis  PubMed  
 Histone methyltransferase SMYD3 promotes MRTF-A-mediated transactivation of MYL9 and migration of MCF-7 breast cancer cells. Myocardin-related transcription factor-A (MRTF-A) is a Rho signal-responsive transcriptional coactivator of serum response factor (SRF). Recent studies indicated that MRTF-A might be an important regulator of mammary gland and be involved in cancer   metastasis  . However, the roles of histone modification in the MRTF-A-dependent signal pathway and tumor migration are still not very clear. Here, we report that histone methylation is required for the MRTF-A-mediated upregulation of myosin regulatory light chain 9 (MYL9), an important cytoskeletal component which is implicated in cell migration. Furthermore, we demonstrate that SET and MYND domain containing protein 3 (SMYD3), a hitone methyltransferase (HMT) associated with carcinogenesis, might be the one which is responsible for the histone methylation occurred in the MRTF-A-mediated- transactivation of MYL9 and migration of breast cancer cells. Overexpression of SMYD3 promotes MRTF-A-mediated upregulation of MYL9 and migration of MCF-7 breast cancer cells, while contrary results were observed when the endogenous MRTF-A and SMYD3 were suppressed with specific siRNAs. In addition, the mutation analysis suggested that this cooperative transactivation is mainly mediated via the proximal binding element of MRTF-A in the promoter of MYL9, and the HMT activity of SMYD3 is required as well. Our findings reveal a new mechanism by which MRTF-A and SMYD3 functions in transcriptional regulation and cell migration, and provide a better understanding for   metastasis   of breast cancer. 
 Histone methyltransferase SMYD3 promotes MRTF-A-mediated transactivation of MYL9 and migration of MCF-7 breast cancer cells. Myocardin-related transcription factor-A (MRTF-A) is a Rho signal-responsive transcriptional coactivator of serum response factor (SRF). Recent studies indicated that MRTF-A might be an important regulator of mammary gland and be involved in cancer   metastasis  . However, the roles of histone modification in the MRTF-A-dependent signal pathway and tumor migration are still not very clear. Here, we report that histone methylation is required for the MRTF-A-mediated upregulation of myosin regulatory light chain 9 (MYL9), an important cytoskeletal component which is implicated in cell migration. Furthermore, we demonstrate that SET and MYND domain containing protein 3 (SMYD3), a hitone methyltransferase (HMT) associated with carcinogenesis, might be the one which is responsible for the histone methylation occurred in the MRTF-A-mediated- transactivation of MYL9 and migration of breast cancer cells. Overexpression of SMYD3 promotes MRTF-A-mediated upregulation of MYL9 and migration of MCF-7 breast cancer cells, while contrary results were observed when the endogenous MRTF-A and SMYD3 were suppressed with specific siRNAs. In addition, the mutation analysis suggested that this cooperative transactivation is mainly mediated via the proximal binding element of MRTF-A in the promoter of MYL9, and the HMT activity of SMYD3 is required as well. Our findings reveal a new mechanism by which MRTF-A and SMYD3 functions in transcriptional regulation and cell migration, and provide a better understanding for   metastasis   of breast cancer. 
 SMYD3 tandem repeats polymorphism is not associated with the occurrence and   metastasis   of hepatocellular carcinoma in a Chinese population. A variable number of tandem repeats (VNTR) polymorphism in regulatory region of SMYD3 coding for histone methyltransferase has been shown to be associated with colorectal cancer, hepatocellular carcinoma (HCC), and breast cancer in Japanese population. Aim of the study is to investigate the potential association between the functional SMYD3 tandem repeats polymorphism and HCC in Chinese population.The case-control study included 200 HCC patients and 261 healthy controls. The VNTR polymorphism in the promoter of SMDY3 was genotyped by PCR and direct-sequencing analysis. Odds ratio and 95% confidence interval were used to estimate the association between the polymorphisms and risk of HCC.The allele frequencies for SMYD3 2 and 3 repeats were 15.71% and 84.29% among controls; and 12.75%, and 87.25% among cases (P = 0.22). The odds ratio for 3/3 versus 2/2 and 2/3 genotypes was 1.30 (P = 0.18). The frequencies of 3 alleles were not increased with HCC stage increased (trend test, P = 0.45).SMYD3 polymorphism is not associated with the occurrence and   metastasis   of HCC in Chinese population. 
 Overexpression of SMYD3 was associated with increased STAT3 activation in gastric cancer. This study aimed to investigate mRNA and protein expressions of SET and MYND domain-containing protein 3 (SMYD3), STAT3, and phosphorylated STAT3 (pSTAT3) in gastric cancer (GC). This study was also conducted to explore the correlations between these proteins and biological behaviors of GC. SMYD3, STAT3, and pSTAT3 expressions were detected in GC tissues and adjacent non-tumor tissues by semiquantitative/quantitative reverse transcription polymerase chain reaction and Western blot analysis. SMYD3, STAT3, and pSTAT3 expressions in tissue sections were evaluated by immunohistochemistry. Staining results were compared with clinicopathological characteristics and the outcome of patients. The mRNA expression levels of SMYD3 or STAT3 and the protein expression levels of SMYD3, STAT3, or pSTAT3 in GC tissues were significantly higher than those in adjacent non-tumor tissues. Lymph node   metastasis   was identified as an independently relative factor for SMYD3 expression; the degree of differentiation and serosal invasion were identified as the independently relative factors for pSTAT3 expression in GC tissues. SMYD3 expression and STAT3 or pSTAT3 expressions in GC tissues were significantly and positively correlated. Multivariate analysis results demonstrated that primary tumor location, lymph node   metastasis  , SMYD3 expression, and pSTAT3 expression were independent prognostic indicators of GC. pSTAT3 expression was an optimal prognostic predictor of patients, as identified by Cox regression with Akaike's information criterion value calculation. High SMYD3 and pSTAT3 expressions may indicate poor prognosis of patients with GC. 
 SMYD3 promotes cancer invasion by epigenetic upregulation of the metalloproteinase MMP-9. Upregulation of the matrix metalloproteinase (MMP)-9 plays a central role in tumor progression and   metastasis   by stimulating cell migration, tumor invasion, and angiogenesis. To gain insights into MMP-9 expression, we investigated its epigenetic control in a reversible model of cancer that is initiated by infection with intracellular Theileria parasites. Gene induction by parasite infection was associated with trimethylation of histone H3K4 (H3K4me3) at the MMP-9 promoter. Notably, we found that the H3K4 methyltransferase SMYD3 was the only histone methyltransferase upregulated upon infection. SMYD3 is overexpressed in many types of cancer cells, but its contributions to malignant pathophysiology are unclear. We found that overexpression of SMYD3 was sufficient to induce MMP-9 expression in transformed leukocytes and fibrosarcoma cells and that proinflammatory phorbol esters further enhanced this effect. Furthermore, SMYD3 was sufficient to increase cell migration associated with MMP-9 expression. In contrast, RNA interference-mediated knockdown of SMYD3 decreased H3K4me3 modification of the MMP-9 promoter, reduced MMP-9 expression, and reduced tumor cell proliferation. Furthermore, SMYD3 knockdown also reduced cellular invasion in a zebrafish xenograft model of cancer. Together, our results define SMYD3 as an important new regulator of MMP-9 transcription, and they provide a molecular link between SMYD3 overexpression and metastatic cancer progression. 
 Overexpression of SMYD3 and matrix metalloproteinase-9 are associated with poor prognosis of patients with gastric cancer. SET and MYND domain-containing protein 3 (SMYD3) plays a key role in the progression of human cancer. Matrix metalloproteinase (MMP)-9 is being related to tumor progression. It has been reported that SMYD3 and MMP-9 are overexpressed in human cancers. However, the exact roles of SMYD3 and MMP-9 in the   metastasis   and prognosis of gastric cancer (GC) remain unclear. The expressions of SMYD3 and MMP-9 were detected by semiquantitative reverse transcription polymerase chain reaction and Western blotting in gastric cancer and adjacent nontumor tissues. In addition, SMYD3 and MMP-9 expressions were analyzed by immunohistochemistry in formalin-fixed samples from 186 gastric cancer patients. The messenger RNA (mRNA) and protein expression levels of SMYD3 and MMP-9 in gastric cancer tissues were both significantly higher than those in adjacent nontumor tissues. In addition, the expression of SMYD3 was correlated with size of primary tumor and lymph node   metastasis  , while size of primary tumor and serosal invasion were identified as the independently relative factors of MMP-9 expression in GC tissues. SMYD3 expression and MMP-9 expression in GC tissues were significantly and positively correlated. Multivariate analysis results demonstrated that degree of differentiation, lymph node   metastasis  , TNM stage, SMYD3 expression, and MMP-9 expression were the independent prognostic indicators of gastric cancer. SMYD3 and MMP-9 may play important roles in tumor invasion,   metastasis  , and prognosis and could work as promising targets for prognostic prediction in gastric cancer. 
 SMYD3 overexpression was a risk factor in the biological behavior and prognosis of gastric carcinoma. SET and MYND domain-containing protein 3 (SMYD3), a histone methyltransferase, plays a key function in the progression of human cancer. However, the role of SMYD3 in gastric carcinoma carcinogenesis has yet to be elucidated. This study aimed to determine the relationships of SMYD3 expression with clinicopathological characteristics and prognosis in gastric carcinoma. The expression of SMYD3 was detected by real-time quantitative reverse transcription PCR and Western blot in gastric carcinoma (GC) cell lines, normal gastric mucosa cell line, GC tissues, and adjacent non-tumor tissues. SMYD3 expression in tissue sections of 180 gastric carcinoma samples were evaluated using immunohistochemistry. The staining results were compared with clinicopathological characteristics and to the outcome of patients. The expression levels of SMYD3 messenger RNA (mRNA) and protein in GC tissues were both higher than those in adjacent non-tumor tissues (p < 0.05). SMYD3 mRNA and protein expression levels were higher in GC cell lines MKN28, SGC7901, and MGC803 than normal gastric mucosa cell line GES-1. SMYD3 expression in gastric carcinoma was significantly correlated with primary tumor size (p < 0.001), lym  ph node me  tastasis (p < 0.001), and TNM stage (p = 0.011). Degree of differentiation [hazard ratio (HR) = 5.113; p = 0.006], serosal invasion (HR = 2.  074; p =   0.024), lymph node metastasis (HR = 1.354; p < 0.001), and SMYD3 expression (HR = 0.564; p = 0.004) were identified as the independent factors of the overall survival (OS) in all enrolled GC   patients.   For patients with positive lymph node metastasis, degree of diff  erentiatio  n (HR = 5.974; p = 0.015), lymph node metastasis (HR = 1.257; p < 0.001), and SMYD3 expression (HR = 0.529; p = 0.004) were the independent prognostic factors of the OS. SMYD3 performed an important function in the aggressiveness of gastric carcinoma and may act as a promising target for prognostic prediction. 
 Elevated Levels of SET and MYND Domain-Containing Protein 3 Are Correlated with Overexpression of Transforming Growth Factor-β1 in Gastric Cancer. The aim of this study was to investigate the messenger RNA and protein expressions of SET and MYND domain-containing protein 3 (SMYD3) and transforming growth factor-β1 (TGF-β1) in gastric cancer (GC) and to explore the correlations between these proteins and the biologic behavior of GC.Expressions of SMYD3 and TGF-β1 were detected by real-time quantitative reverse transcription polymerase chain reaction and Western blot in GC tissues and adjacent nontumor tissues. In addition, SMYD3 and TGF-β1 expressions were analyzed by immunohistochemistry in formalin-fixed samples from 166 GC patients.The messenger RNA and protein expression levels of SMYD3 and TGF-β1 in GC tissues were significantly higher than those in adjacent nontumor tissues. A significantly positive correlation was found between SMYD3 expression and TGF-β1 expression in GC tissues. In addition, the size of the primary tumor and lymp  h node met  astasis were identified as the independently relative factors of SMYD3 expression in GC tissues, and lymp  h node met  astasis was identified as the independently relative factor of TGF-β1 expression. Multivariate analysis demonstrated that the degree of differentiation, serosal invasion, lym  ph node me  tastasis, SMYD3 expression, and TGF-β1 expression were the independent prognostic indicators for GC. Transforming growth factor-β1 expression was one of the optimal prognostic predictors of patients identified using the Cox regression with Akaike Information Criterion value calculation.SET and MYND domain-containing protein 3 expression and TGF-β1 expression in GC tissues were significantly and positively correlated. High expression levels of SMYD3 and TGF-β1 can indicate poor prognoses for GC patients. 
  GeneRIFs  
 SMYD3 and MMP-9 may play important roles in tumor invasion,   metastasis  , and prognosis and could work as promising targets for prognostic prediction in gastric cancer. 
 study shows that SMYD3 polymorphism is not associated with the occurrence and   metastasis   of hepatocellular carcinoma in Chinese population 
   
  
 VGLL4 
 
  NCBI Gene  
  GeneRIF  
  Pubmed  
 
 
  Search Term  Rank  Fields  
  [Mm]igration  6  PubMed(2)  
 
 
 hide/show details for VGLL4 
  [Mm]igration  PubMed  
 VGLL4 inhibits EMT in part through suppressing Wnt/β-catenin signaling pathway in gastric cancer. VGLL4 is a member of the Vestigial-like proteins that functions as a tumor suppressor, which directly competes with YAP for binding TEADs in several cancer types. Recently, an increasing number of studies have reported that VGLL4 acts as a crucial role in regulating cell mobility,   migratio  n, and invasion. However, little is known about the signaling mechanisms in regulating epithelial-mesenchymal transition (EMT) of gastric cancer. In our study, we confirmed that the expression level of VGLL4 was down-regulated in gastric cancer tissues, and reduced VGLL4 expression levels inhibited apoptosis and promoted proliferation,   migratio  n, and invasion. Additionally, we found a phenomenon that VGLL4 was associated with the change in nuclear location of β-catenin, which suggested that β-catenin was a significant downstream factor of VGLL4. These results suggest that VGLL4 suppressed EMT in part via negative regulation of Wnt/β-catenin signaling pathway. Taken together, our study demonstrated that VGLL4 is important in the process of suppressing tumor progression of gastric cancer and provided a potential therapeutic strategy for gastric cancer. 
 Downregulation of VGLL4 in the progression of esophageal squamous cell carcinoma. VGLL4, a member of the Vestigial-like (VGLL) proteins, has been reported to be dysregulated in several cancer types. However, its function in esophageal squamous cell carcinoma (ESCC) remains poorly understood. Here, it was found that the expression level of VGLL4 was decreased in ESCC tissues. Moreover, forced expression of VGLL4 in ESCC cells inhibited cell growth and   migration  , while knockdown of VGLL4 expression promoted the tumorigenecity of ESCC cells. Mechanistically, VGLL4 regulated the growth and motility of ESCC cells through downregulating the expression of connective tissue growth factor (CTGF), a known oncogene in the progression of ESCC. Taken together, our study suggested that downregulation of VGLL4 was very important in the progression of ESCC, and restoring the function of VGLL4 might be a promising therapeutic strategy for ESCC. 
   
  
 CTSG 
 
  NCBI Gene  
  GeneRIF  
  Pubmed  
 
 
  Search Term  Rank  Fields  
  [Mm]igration  6  PubMed(2)  
  ECM  7  PubMed(1)  
  [Ee]xtracellular [Mm]atrix  8  Process(1); PubMed(4); Component(1)  
 
 
 hide/show details for CTSG 
  [Mm]igration  PubMed  
 Cathepsin G: the significance in rheumatoid arthritis as a monocyte chemoattractant. Human cathepsin G (EC 3.4.21.20) has been reported to have the in vitro chemotactic activity for human monocytes. In this study, we examined the role of cathepsin G in monocyte involvement in joint inflammation of rheumatoid arthritis (RA) as a monocyte chemoattractant. Eighteen patients with RA and four patients with osteoarthritis (OA) were used in this study. Thiobenzylester substrate, Succ-Phe-Leu-Phe-S-Bzl, was used to measure the activity of cathepsin G in synovial fluids. Monocyte   migration   induced by cathepsin G and synovial fluids was assessed by a 48-well microchemotaxis chamber technique. Immunohistochemical staining was performed to determine the cellular origin of cathepsin G in RA synovial tissue. A very low activity of cathepsin G was detected in synovial fluids from patients with OA. On the other hand, significantly increased activity of cathepsin G was detected in patients with RA when compared with the value of OA patients. A considerable monocyte chemotactic activity was detected in the synovial fluid of RA patients, and the activity was partially decreased by the treatment with inhibitors for cathepsin G, alpha1-antichymotrypsin and phenylmethylsulfonyl fluoride. The activity of cathepsin G was significantly correlated with the neutrophil counts in synovial fluids and the concentration of interleukin-6. Immunohistochemical studies showed that cathepsin G was strongly expressed by synovial lining cells, and weakly expressed by macrophages and neutrophils in synovial tissues. This study indicates that the monocyte chemotactic activity of cathepsin G may have a role in the pathogenesis of RA synovial inflammation. 
 Regulation of P-selectin binding to the neutrophil P-selectin counter-receptor P-selectin glycoprotein ligand-1 by neutrophil elastase and cathepsin G. In the inflammatory response, leukocyte rolling before adhesion and trans  migration   through the blood vessel wall is mediated by specific cell surface adhesion receptors. Neutrophil rolling involves the interaction of P-selectin expressed on activated endothelium and its counter-receptor on neutrophils, P-selectin glycoprotein ligand-1 (PSGL-1). Here, it is reported that P-selectin binding to neutrophils is lost under conditions that cause the release of proteinases from neutrophil primary granules. Treatment of neutrophils with the purified neutrophil granule proteinases, cathepsin G and elastase, rapidly abolished their capacity to bind P-selectin. This inactivation corresponded to loss of the N-terminal domain of PSGL-1, as assessed by Western blot analysis. A loss of intact PSGL-1 protein from the surfaces of neutrophils after the induction of degranulation was also detected by Western blot analysis. Cathepsin G initially cleaved near the PSGL-1 N-terminus, whereas neutrophil elastase predominantly cleaved at a more C-terminal site within the protein mucin core. Consistent with this, cathepsin G cleaved a synthetic peptide based on the PSGL-1 N-terminus between Tyr-7/Leu-8. Under conditions producing neutrophil degranulation in incubations containing mixtures of platelets and neutrophils, the loss of PSGL-1, but not P-selectin, from platelet-neutrophil lysates was detected. Cathepsin G- or neutrophil elastase-mediated PSGL-1 proteolysis may constitute a potential autocrine mechanism for down-regulation of neutrophil adhesion to P-selectin. 
  ECM  PubMed  
 Proteomics characterization of extracellular space components in the human aorta. The vascular extracellular matrix (  ECM  ) is essential for the structural integrity of the vessel wall and also serves as a substrate for the binding and retention of secreted products of vascular cells as well as molecules coming from the circulation. Although proteomics has been previously applied to vascular tissues, few studies have specifically targeted the vascular   ECM   and its associated proteins. Thus, its detailed composition remains to be characterized. In this study, we describe a methodology for the extraction of extracellular proteins from human aortas and their identification by proteomics. The approach is based on (a) effective decellularization to enrich for scarce extracellular proteins, (b) successful solubilization and deglycosylation of   ECM   proteins, and (c) relative estimation of protein abundance using spectral counting. Our three-step extraction approach resulted in the identification of 103 extracellular proteins of which one-third have never been reported in the proteomics literature of vascular tissues. In particular, three glycoproteins (podocan, sclerostin, and agrin) were identified for the first time in human aortas at the protein level. We also identified extracellular adipocyte enhancer-binding protein 1, the cartilage glycoprotein asporin, and a previously hypothetical protein, retinal pigment epithelium (RPE) spondin. Moreover, our methodology allowed us to screen for proteolysis in the aortic samples based on the identification of proteolytic enzymes and their corresponding degradation products. For instance, we were able to detect matrix metalloproteinase-9 by mass spectrometry and relate its presence to degradation of fibronectin in a clinical specimen. We expect this proteomics methodology to further our understanding of the composition of the vascular extracellular environment, shed light on   ECM   remodeling and degradation, and provide insights into important pathological processes, such as plaque rupture, aneurysm formation, and restenosis. 
  [Ee]xtracellular [Mm]atrix  Process  
   extracellular matrix   disassembly 
  PubMed  
 Proteomics characterization of extracellular space components in the human aorta. The vascular   extracellular matrix   (ECM) is essential for the structural integrity of the vessel wall and also serves as a substrate for the binding and retention of secreted products of vascular cells as well as molecules coming from the circulation. Although proteomics has been previously applied to vascular tissues, few studies have specifically targeted the vascular ECM and its associated proteins. Thus, its detailed composition remains to be characterized. In this study, we describe a methodology for the extraction of extracellular proteins from human aortas and their identification by proteomics. The approach is based on (a) effective decellularization to enrich for scarce extracellular proteins, (b) successful solubilization and deglycosylation of ECM proteins, and (c) relative estimation of protein abundance using spectral counting. Our three-step extraction approach resulted in the identification of 103 extracellular proteins of which one-third have never been reported in the proteomics literature of vascular tissues. In particular, three glycoproteins (podocan, sclerostin, and agrin) were identified for the first time in human aortas at the protein level. We also identified extracellular adipocyte enhancer-binding protein 1, the cartilage glycoprotein asporin, and a previously hypothetical protein, retinal pigment epithelium (RPE) spondin. Moreover, our methodology allowed us to screen for proteolysis in the aortic samples based on the identification of proteolytic enzymes and their corresponding degradation products. For instance, we were able to detect matrix metalloproteinase-9 by mass spectrometry and relate its presence to degradation of fibronectin in a clinical specimen. We expect this proteomics methodology to further our understanding of the composition of the vascular extracellular environment, shed light on ECM remodeling and degradation, and provide insights into important pathological processes, such as plaque rupture, aneurysm formation, and restenosis. 
 Inflammation-related neutrophil proteases, cathepsin G and elastase, function as insulin-like growth factor binding protein proteases. Over the past few years, several proteolytic enzymes have been identified as insulin-like growth factor binding protein (IGFBP) proteases. It has been suggested that proteolytic cleavage of IGFBPs is associated with regulation of the proliferative effects of IGFs on their target cells. In this study, we have demonstrated that two neutrophil proteases, cathepsin G and elastase, effectively cleave IGFBPs in vitro and in vivo at concentrations lower than previous described IGFBP proteases. Purified leukocyte cathepsin G and elastase cleaved all six well-characterized IGFBPs into distinct fragments in a concentration-dependent manner. Under similar experimental conditions, cathepsin G preferentially cleaved IGFBP-5, followed by BP-2, BP-3, BP-4, BP-1, and BP-6. In comparison, elastase equally preferred IGFBP-3 and IGFBP-4, followed by BP-1, BP-5, BP-6, and BP-2. Proteolysis of rh(125)I-IGFBP-3 by cathepsin G was blocked by alpha(1)-antichymotrypsin, while elastase proteolytic activity was blocked by alpha(1)-proteinase inhibitor as expected. Elastase, but not cathepsin G, cleaved free IGF-I into a smaller molecular weight fragment in vitro, possibly designating unique functions for each protease within the IGF axis. Sequence analysis of IGFBP-3 fragments produced by cathepsin G and elastase demonstrated that each protease cleaved IGFBP-3 at unique sites within its midregion. More importantly, extracts from purified neutrophils have demonstrated significant proteolytic cleavage of IGFBP-3 that resembles elastase proteolysis of IGFBP-3. Recent studies using a monocyte-like cell model have also shown significant cleavage of IGFBP-3. These in vitro and in vivo data suggest that the neutrophil proteases, cathepsin G and elastase, in addition to their previously described functions as   extracellular matrix  -degrading enzymes, may potentially act as IGFBP proteases involved in regulation of IGFs and IGFBPs during inflammation and wound healing. 
 Activity and localization of cathepsin B, D and G in aortic aneurysm. The activities of cathepsin B, D and G, and their distribution in the walls of normal aorta and those of aneurysms of abdominal aorta were studied. The immunohistochemical reaction for these cathepsins was positive in the aneurysm wall both in the cells and in the   extracellular matrix  . It was more apparent than in normal aortas. The activities of cathepsin B, D and G in aneurysm were 1.5 times higher than in normal aortas. It may be suspected that besides the action of collagenases and elastases the cathepsins participate in an enhanced degradation of collagen and elastin fibers of aneurysm wall. 
 Cell surface-bound elastase and cathepsin G on human neutrophils: a novel, non-oxidative mechanism by which neutrophils focus and preserve catalytic activity of serine proteinases. Serine proteinases of human polymorphonuclear neutrophils play an important role in neutrophil-mediated proteolytic events; however, the non-oxidative mechanisms by which the cells can degrade   extracellular matrix   in the presence of proteinase inhibitors have not been elucidated. Herein, we provide the first report that human neutrophils express persistently active cell surface-bound human leukocyte elastase and cathepsin G on their cell surface. Unstimulated neutrophils have minimal cell surface expression of these enzymes; however, phorbol ester induces a 30-fold increase. While exposure of neutrophils to chemoattractants (fMLP and C5a) stimulates modest (two- to threefold) increases in cell surface expression of serine proteinases, priming with concentrations of lipopolysaccharide as low as 100 fg/ml leads to striking (up to 10-fold) increase in chemoattractant-induced cell surface expression, even in the presence of serum proteins. LPS-primed and fMLP-stimulated neutrophils have approximately 100 ng of cell surface human leukocyte elastase activity per 10(6) cells. Cell surface-bound human leukocyte elastase is catalytically active, yet is remarkably resistant to inhibition by naturally occurring proteinase inhibitors. These data indicate that binding of serine proteinases to the cell surface focuses and preserves their catalytic activity, even in the presence of proteinase inhibitors. Upregulated expression of persistently active cell surface-bound serine proteinases on activated neutrophils provides a novel mechanism to facilitate their egress from the vasculature, penetration of tissue barriers, and recruitment into sites of inflammation. Dysregulation of the cell surface expression of these enzymes has the potential to cause tissue destruction during inflammation. 
  Component  
   extracellular matrix   
   
  
 GZMH 
 
  NCBI Gene  
  GeneRIF  
  Pubmed  
 
 
  Search Term  Rank  Fields  
  [Ee]xtracellular [Mm]atrix  8  PubMed(1)  
 
 
 hide/show details for GZMH 
  [Ee]xtracellular [Mm]atrix  PubMed  
 The human cytotoxic T cell granule serine protease granzyme H has chymotrypsin-like (chymase) activity and is taken up into cytoplasmic vesicles reminiscent of granzyme B-containing endosomes. Serine proteases (granzymes) contained within the cytoplasmic granules of cytotoxic T cells and natural killer cells play a variety of roles including the induction of target cell apoptosis, breakdown of   extracellular matrix   proteins and induction of cytokine secretion by bystander leukocytes. Different granzymes display proteolytic specificities that mimic the activities of trypsin or chymotrypsin, or may cleave substrates at acidic ("Asp-ase") or at long unbranched amino acids such as Met ("Met-ase"). Here, we report that recombinant granzyme H has chymotrypsin-like (chymase) activity, the first report of a human granzyme with this proteolytic specificity. Recombinant 32-kDa granzyme H expressed in the baculovirus vector pBacPAK8 was secreted from Sf21 cells and recovered by Ni-affinity chromatography, using a poly-His tag encoded at the predicted carboxyl terminus of full-length granzyme H cDNA. The granzyme H efficiently cleaved Suc-Phe-Leu-Phe-SBzl (v = 185 nM/s at [S] = 0.217 mM) and also hydrolyzed Boc-Ala-Ala-X-SBzl (X = Phe, Tyr, Met, Nle, or Nva) with slower rates but had little tryptase or Asp-ase activity. Enzymatic activity was inhibited completely by 0.1 mM 3,4-dichloroisocoumarin and 84% by 1.0 mM phenylmethylsulfonyl fluoride. Fluoresceinated granzyme H was internalized in a temperature-dependent manner by Jurkat cells into endosome-like vesicles, suggesting that it can bind to cell surface receptors similar to those that bind granzyme B. This suggests a hitherto unsuspected intracellular function for granzyme H. 
   
  
 CMA1 
 
  NCBI Gene  
  GeneRIF  
  Pubmed  
 
 
  Search Term  Rank  Fields  
  [Mm]igration  6  PubMed(4); GeneRIFs(3)  
  ECM  7  PubMed(1)  
  [Ee]xtracellular [Mm]atrix  8  Process(1); PubMed(7); Component(1); GeneRIFs(1); Summary(1)  
 
 
 hide/show details for CMA1 
  [Mm]igration  PubMed  
 Eosinophil   migration   induced by mast cell chymase is mediated by extracellular signal-regulated kinase pathway. Mast cell chymase is known to induce eosinophil   migration   in vivo and in vitro. In the present study, we investigated possible involvement of mitogen-activated protein (MAP) kinases; extracellular signal-regulated kinase (ERK), c-Jun amino-terminal kinase (JNK), and p38, in the chymase-induced eosinophil   migration  . Human chymase induced a rapid phosphorylation of ERK1/2 and p38 in human eosinophilic leukemia EoL-1 cells, while no phosphorylation was detected in JNK. The chymase-induced phosphorylation of ERK and p38 was inhibited by pertussis toxin. Similar results were obtained in the experiments using mouse chymase and eosinophils. U0126 (the inhibitor for MAP/ERK kinase) suppressed chymase-induced   migration   of EoL-1 cells and mouse eosinophils. However, SB203580 (p38 inhibitor) and SP600125 (JNK inhibitor) showed little effect on the   migration  . It is suggested therefore that chymase activates ERK and p38 probably through G-protein-coupled receptor, and that ERK but not p38 cascade may have a crucial role in chymase-induced   migration   of eosinophils. 
 Eosinophil   migration   induced by mast cell chymase is mediated by extracellular signal-regulated kinase pathway. Mast cell chymase is known to induce eosinophil   migration   in vivo and in vitro. In the present study, we investigated possible involvement of mitogen-activated protein (MAP) kinases; extracellular signal-regulated kinase (ERK), c-Jun amino-terminal kinase (JNK), and p38, in the chymase-induced eosinophil   migration  . Human chymase induced a rapid phosphorylation of ERK1/2 and p38 in human eosinophilic leukemia EoL-1 cells, while no phosphorylation was detected in JNK. The chymase-induced phosphorylation of ERK and p38 was inhibited by pertussis toxin. Similar results were obtained in the experiments using mouse chymase and eosinophils. U0126 (the inhibitor for MAP/ERK kinase) suppressed chymase-induced   migration   of EoL-1 cells and mouse eosinophils. However, SB203580 (p38 inhibitor) and SP600125 (JNK inhibitor) showed little effect on the   migration  . It is suggested therefore that chymase activates ERK and p38 probably through G-protein-coupled receptor, and that ERK but not p38 cascade may have a crucial role in chymase-induced   migration   of eosinophils. 
 Mechanical induction of an epithelial cell chymase associated with wound edge   migration  . Chymase is a chymotrypsin-like serine protease predominantly produced by mast cells. In this study, human cutaneous and gingival keratinocytes, ovary surface epithelia, and a porcine epithelial cell line were assayed by homology-based cloning, and the amplified DNA fragment was identified as a chymase. In vitro, chymase could not be induced by serum or cytokine treatment alone. Chymase was activated 3-fold within 60 min in basal media by scratch wounding cultured monolayers and further potentiated over 10-fold at 18 h by additional serum and cytokine treatment. Chymase activity was cell-associated and found to peak within 24 h of wounding and then steadily decreased as cultures healed, reaching baseline levels before confluence was reestablished. Affinity column purified enzyme effectively degraded fibronectin and was found by Western blot analysis using a human chymase antibody to be of about 30 kDa. Immunostaining revealed chymase activation at the wound edge colocalizing with reactive oxygen species generation. Specifically, chymase activation was attenuated by inhibition of nitric oxide, superoxide, and peroxynitrite. Exogenous peroxynitrite but not hydrogen peroxide also resulted in chymase activation in unwounded monolayers. Disruption of cytoskeletal stress fibers by cytochalasin D attenuated both wound-activated chymase and reactive oxygen species generation. Chymase inhibitor chymostatin reduced the loss of cell-cell contacts and the onset of porcine and human skin epithelial cell   migration   at the wound edge. This shows that an epithelial chymase is rapidly activated by a ligand-independent mechanism following mechanical stress via cytoskeletal and reactive oxygen species signaling and is associated with the onset of epithelial cell   migration  . 
 The effects of chymase on matrix metalloproteinase-2 activation in neointimal hyperplasia after balloon injury in dogs. Chymase is known to generate angiotensin II in the vascular wall. In this study we investigated a novel role for chymase other than angiotensin II production in vascular proliferation after balloon injury. Chymase promoted the   migration   of vascular smooth muscle cells in the matrix-coated invasion chambers and activated promatrix metalloproteinase-2 obtained from the culture medium of vascular smooth muscle cells. Two weeks after balloon injury, significant neointimal formation was found in dog carotid arteries. After injury, active matrix metalloproteinase-2 was increased in parallel with the augmentation of chymase activity that was seen in the proliferating region of the vascular wall. The oral administration of NK3201 (1 mg/kg per day), a chymase inhibitor, prevented neointimal formation and significantly suppressed both active matrix metalloproteinase-2 and chymase activities 2 weeks after injury. These results suggest that chymase inhibitors can prevent the development of intimal hyperplasia via the inhibition of matrix metalloproteinase-2 activation in balloon-injured arteries. 
  GeneRIFs  
 epithelial chymase is rapidly activated by a ligand-independent mechanism following mechanical stress via cytoskeletal and reactive oxygen species signaling and is associated with the onset of epithelial cell   migration   
 Chymase promoted the   migration   of vascular smooth muscle cells in the matrix-coated invasion chambers and activated promatrix metalloproteinase-2 obtained from the culture medium of vascular smooth muscle cells. 
 mast cell chymase activates ERK and p38 probably through G-protein-coupled receptor, and the ERK but not p38 cascade may have a crucial role in chymase-induced   migration   of eosinophils 
  ECM  PubMed  
 Proteomics characterization of extracellular space components in the human aorta. The vascular extracellular matrix (  ECM  ) is essential for the structural integrity of the vessel wall and also serves as a substrate for the binding and retention of secreted products of vascular cells as well as molecules coming from the circulation. Although proteomics has been previously applied to vascular tissues, few studies have specifically targeted the vascular   ECM   and its associated proteins. Thus, its detailed composition remains to be characterized. In this study, we describe a methodology for the extraction of extracellular proteins from human aortas and their identification by proteomics. The approach is based on (a) effective decellularization to enrich for scarce extracellular proteins, (b) successful solubilization and deglycosylation of   ECM   proteins, and (c) relative estimation of protein abundance using spectral counting. Our three-step extraction approach resulted in the identification of 103 extracellular proteins of which one-third have never been reported in the proteomics literature of vascular tissues. In particular, three glycoproteins (podocan, sclerostin, and agrin) were identified for the first time in human aortas at the protein level. We also identified extracellular adipocyte enhancer-binding protein 1, the cartilage glycoprotein asporin, and a previously hypothetical protein, retinal pigment epithelium (RPE) spondin. Moreover, our methodology allowed us to screen for proteolysis in the aortic samples based on the identification of proteolytic enzymes and their corresponding degradation products. For instance, we were able to detect matrix metalloproteinase-9 by mass spectrometry and relate its presence to degradation of fibronectin in a clinical specimen. We expect this proteomics methodology to further our understanding of the composition of the vascular extracellular environment, shed light on   ECM   remodeling and degradation, and provide insights into important pathological processes, such as plaque rupture, aneurysm formation, and restenosis. 
  [Ee]xtracellular [Mm]atrix  Process  
   extracellular matrix   disassembly 
  PubMed  
 Proteomics characterization of extracellular space components in the human aorta. The vascular   extracellular matrix   (ECM) is essential for the structural integrity of the vessel wall and also serves as a substrate for the binding and retention of secreted products of vascular cells as well as molecules coming from the circulation. Although proteomics has been previously applied to vascular tissues, few studies have specifically targeted the vascular ECM and its associated proteins. Thus, its detailed composition remains to be characterized. In this study, we describe a methodology for the extraction of extracellular proteins from human aortas and their identification by proteomics. The approach is based on (a) effective decellularization to enrich for scarce extracellular proteins, (b) successful solubilization and deglycosylation of ECM proteins, and (c) relative estimation of protein abundance using spectral counting. Our three-step extraction approach resulted in the identification of 103 extracellular proteins of which one-third have never been reported in the proteomics literature of vascular tissues. In particular, three glycoproteins (podocan, sclerostin, and agrin) were identified for the first time in human aortas at the protein level. We also identified extracellular adipocyte enhancer-binding protein 1, the cartilage glycoprotein asporin, and a previously hypothetical protein, retinal pigment epithelium (RPE) spondin. Moreover, our methodology allowed us to screen for proteolysis in the aortic samples based on the identification of proteolytic enzymes and their corresponding degradation products. For instance, we were able to detect matrix metalloproteinase-9 by mass spectrometry and relate its presence to degradation of fibronectin in a clinical specimen. We expect this proteomics methodology to further our understanding of the composition of the vascular extracellular environment, shed light on ECM remodeling and degradation, and provide insights into important pathological processes, such as plaque rupture, aneurysm formation, and restenosis. 
 Heparanase-mediated cleavage of macromolecular heparin accelerates release of granular components of mast cells from extracellular matrices. Heparanase cleaves macromolecular heparin in the secretory granules of connective tissue-type mast cells. We investigated roles of the cleavage under a microenvironment mimicking where the mast cells physiologically reside. A connective tissue-type mast cell line MST and mouse peritoneal cell-derived mast cells stored macromolecular heparin in the secretory granules. The cells expressing heparanase stored fragmented heparin (~10 kDa) due to heparanase-dependent cleavage of the heparin. We produced an artificial collagen-based   extracellular matrix   and placed the live cells or glycosaminoglycans purified from the cells in the matrix to measure the release of sulfated macromolecules into the medium. The sulfate-radiolabelled molecules from the degranulating heparanase-expressing cells and the purified glycosaminoglycans showed significantly greater release into the medium than those derived from mock cells, which was not the case in suspension culture. The mast cell granular enzyme chymase, but not β-hexosaminidase, showed significantly greater release from the degranulating heparanase-expressing cells than from mock cells. Purified chymase mixed with fragmented heparin derived from heparanase-expressing cells showed greater release from collagen gels than the enzyme alone or mixed with macromolecular heparin derived from mock cells. We propose that the cleavage of macromolecular heparin by heparanase accelerates the release of heparin and chymase from extracellular matrices. 
 Mast cell chymase induces conjunctival epithelial cell apoptosis by a mechanism involving degradation of fibronectin. To determine the mechanism of conjunctival epithelial injury in vernal keratoconjunctivitis, we investigated the effects of human chymase on conjunctival epithelial cells in vitro.Human conjunctival epithelial cells were incubated with human chymase for 24 or 48 hr at levels of activity that were likely to exist in the tear fluid of patients with vernal keratoconjunctivitis. Morphologic changes of the cells were observed by phase contrast microscopy. To determine the number of detached cells, we used an automated cell counter, while apoptotic cells were quantitated by flow cytometry. The level of soluble fibronectin in conditioned medium was measured by ELISA.Most of the cells in the incubation with chymase were detached by 24 hr. However, chymase-mediated apoptosis was a slower process and was only detected after incubation of cells with chymase for 36 to 48 hr. Both cell detachment and apoptosis were blocked when cells were incubated in fibronectin-coated plates. The increase of soluble fibronectin was dependent on the amount of chymase added and the exposure time. A caspase inhibitor (antiapoptotic agent) rescued cells from apoptosis but did not prevent cell detachment. These results indicate that chymase-induced apoptosis of conjunctival epithelial cells represents anoikis, which is a slowly occurring apoptotic process induced by lack of adhesion to an   extracellular matrix  .Human mast cell chymase caused conjunctival epithelial cell detachment by degrading fibronectin, and this led to secondary apoptosis. 
 Effect of chymase inhibitor on vascular proliferation. In vascular tissues, angiotensin II is potentially cleaved from angiotensin I by chymase and angiotensin-converting enzyme (ACE). In the normal state, vascular ACE regulates local angiotensin II formation and plays a crucial role in the regulation of blood pressure, whereas chymase is stored in mast cells and has no enzymatic activity. Chymase is activated immediately upon its release into the   extracellular matrix   in vascular tissues after mast cells have been activated by stimuli such as vessel injury by grafting or a balloon catheter. In dog grafted veins, chymase activity is increased, and the vascular proliferation is suppressed by either a chymase inhibitor or an angiotensin II receptor blocker. After balloon injury in dog vessels, chymase activity is significantly increased in the injured artery, and a chymase inhibitor is effective in preventing the vascular proliferation, but an ACE inhibitor is ineffective. Chymase plays an important role in the development of vascular proliferation via the induction of local angiotensin II formation in injured vessels. 
 Polymorphisms of matrix metalloproteinase-7 and chymase are associated with susceptibility to and progression of gastric cancer in Japan. Matrix metalloproteinases (MMPs) are a family of enzymes that degrade most macromolecules making up the   extracellular matrix  . MMPs are involved in not only the gastric mucosal inflammatory response but also the pathogenesis of Helicobacter pylori-associated diseases. In the renin-angiotensin system, chymase (CMA) is related to gastric carcinogenesis and angiogenesis in H. pylori-infected patients. We aimed to clarify the association of MMP-7-181 and CMA/B polymorphisms with susceptibility to gastric cancer and cancer progression in H. pylori-infected patients.We assessed the MMP-7-181 and CMA/B polymorphisms in H. pylori-positive patients with gastric cancer (n = 160), gastric ulcer (n = 157), duodenal ulcer (n = 121), and H. pylori-positive gastritis alone as controls (n = 156).For gastric cancer risk, the age-and sex-adjusted odds ratio (OR) of the MMP-7-181 G allele carrier relative to the A/A genotype was significantly increased [OR, 2.32; 95% confidence interval (CI), 1.24-4.35], especially in patients with noncardia cancer (OR, 2.31; 95% CI, 1.22-4.36) and those with clinical stage III or IV cancer (OR, 3.66; 95% CI, 1.54-8.73). Carriage of the CMA/B A allele was significantly associated with gastric cancer development (OR, 1.73; 95% CI, 1.10-2.71). Simultaneous carriage of both the MMP-7-181 G allele and the CMA/B A allele dramatically increased the gastric cancer risk (OR, 8.18; 95% CI, 2.79-23.93).In Japan, carriage of the MMP-7-181 G allele and of the CMA/B A allele were each associated with an increased risk for H. pylori-related noncardia gastric cancer development. MMP-7-181 and CMA/B genotyping tests might be useful tools for screening for individuals with higher gastric cancer risk. 
 Mast cell chymase modifies cell-matrix interactions and inhibits mitogen-induced proliferation of human airway smooth muscle cells. The hallmarks of chronic, severe asthma include prominent airway inflammation and airway smooth muscle (ASM) hypertrophy and hyperplasia. One of the factors that contribute to the injury and repair process within the airway is activation of proteases and turnover of   extracellular matrix   components. Mast cells, which are present in increased numbers in the asthmatic airway, are a rich source of the neutral protease chymase, which can degrade several basement membrane components. Recent data suggest that proteases also play a critical role in regulating the expression of CD44, the primary receptor for the matrix glycosaminoglycan hyaluronan. In this study we investigated the effects of chymase treatment on human ASM cell function. We found that chymase degraded the smooth muscle cell pericellular matrix. This was accompanied by an increased release of fibronectin and soluble CD44, but not soluble ICAM-1 or soluble hyaluronan, into the conditioned medium. In addition, chymase inhibited T cell adhesion to ASM and dramatically reduced epidermal growth factor-induced smooth muscle cell proliferation. These data suggest that the local release of mast cell chymase may have profound effects on ASM cell function and airway remodeling. 
 Cellular localization and regional distribution of an angiotensin II-forming chymase in the heart. The human heart is a target organ for the octapeptide hormone, angiotensin II (Ang II). Recent studies suggest that the human heart contains a dual pathway of Ang II formation in which the major Ang II-forming enzymes are angiotensin I-converting enzyme (ACE) and chymase. Human heart chymase has recently been purified and its cDNA and gene cloned. This cardiac serine proteinase is the most efficient and specific Ang II-forming enzyme described. To obtain insights into the cardiac sites of chymase-dependent Ang II formation, we examined the cellular localization and regional distribution of chymase in the human heart. Electron microscope immunocytochemistry using an anti-human chymase antibody showed the presence of chymase-like immunoreactivity in the cardiac interstitium and in cytosolic granules of mast cells, endothelial cells, and some mesenchymal interstitial cells. In the cardiac interstitium, chymase-like immunoreactivity is associated with the   extracellular matrix  . In situ hybridization studies further indicated that chymase mRNA is expressed in endothelial cells and in interstitial cells, including mast cells. Tissue chymase levels were determined by activity assays and by Western blot analyses. Chymase levels were approximately twofold higher in ventricles than in atria. There were no significant differences in chymase levels in ventricular tissues obtained from non-failing donor hearts, failing ischemic hearts, or hearts from patients with ischemic cardiomyopathy. These findings suggest that a major site of chymase-dependent Ang II formation in the heart is the interstitium and that cardiac mast cells, mesenchymal interstitial cells, and endothelial cells are the cellular sites of synthesis and storage of chymase. In the human heart, because ACE levels are highest in the atria and chymase levels are highest in ventricles, it is likely that the relative contribution of ACE and chymase to cardiac Ang II formation varies with the cardiac chamber. Such differences may lead to differential suppression of cardiac Ang II levels during chronic ACE inhibitor therapy in patients with congestive heart failure. 
  Component  
   extracellular matrix   
  GeneRIFs  
 Chymase-induced apoptosis of conjunctival epithelial cells represents anoikis, which is a slowly occurring apoptotic process induced by lack of adhesion to an   extracellular matrix  . 
  Summary  
 This gene encodes a chymotryptic serine proteinase that belongs to the peptidase family S1. It is expressed in mast cells and is thought to function in the degradation of the   extracellular matrix  , the regulation of submucosal gland secretion, and the generation of vasoactive peptides. In the heart and blood vessels, this protein, rather than angiotensin converting enzyme, is largely responsible for converting angiotensin I to the vasoactive peptide angiotensin II. Alternative splicing results in multiple variants. [provided by RefSeq, Apr 2015] 
   
  
 GZMA 
 
  NCBI Gene  
  GeneRIF  
  Pubmed  
 
 
  Search Term  Rank  Fields  
  [Mm]igration  6  PubMed(1)  
 
 
 hide/show details for GZMA 
  [Mm]igration  PubMed  
 Granzyme A Is Required for Regulatory T-Cell Mediated Prevention of Gastrointestinal Graft-versus-Host Disease. In our previous work we could identify defects in human regulatory T cells (Tregs) likely favoring the development of graft-versus-host disease (GvHD) following allogeneic stem cell transplantation (SCT). Treg transcriptome analyses comparing GvHD and immune tolerant patients uncovered regulated gene transcripts highly relevant for Treg cell function. Moreover, granzyme A (GZMA) also showed a significant lower expression at the protein level in Tregs of GvHD patients. GZMA induces cytolysis in a perforin-dependent, FAS-FASL independent manner and represents a cell-contact dependent mechanism for Tregs to control immune responses. We therefore analyzed the functional role of GZMA in a murine standard model for GvHD. For this purpose, adoptively transferred CD4+CD25+ Tregs from gzmA-/- mice were analyzed in comparison to their wild type counterparts for their capability to prevent murine GvHD. GzmA-/- Tregs home efficiently to secondary lymphoid organs and do not show phenotypic alterations with respect to activation and   migration   properties to inflammatory sites. Whereas gzmA-/- Tregs are highly suppressive in vitro, Tregs require GZMA to rescue hosts from murine GvHD, especially regarding gastrointestinal target organ damage. We herewith identify GZMA as critical effector molecule of human Treg function for gastrointestinal immune response in an experimental GvHD model. 
   
  
 ST14 
 
  NCBI Gene  
  GeneRIF  
  Pubmed  
 
 
  Search Term  Rank  Fields  
  [Mm]igration  6  PubMed(14); GeneRIFs(1)  
  ECM  7  PubMed(2); GeneRIFs(1)  
  [Ee]xtracellular [Mm]atrix  8  PubMed(10); GeneRIFs(1)  
  [Ii]nvasive  10  PubMed(15); GeneRIFs(3)  
  [Mm]etastasis  12  PubMed(18); GeneRIFs(4); Summary(1)  
  [Cc]ell [Ii]nvasion  13  PubMed(7); GeneRIFs(3)  
 
 
 hide/show details for ST14 
  [Mm]igration  PubMed  
 Epithin/PRSS14 proteolytically regulates angiopoietin receptor Tie2 during transendothelial   migration  . Epithin/PRSS14, a type II transmembrane serine protease, is involved in normal epithelial development and tumor progression. Here we report, as an interacting substrate of epithin, a receptor tyrosine kinase Tie2 that is well known for important roles in the vessel stability. Epithin interacts with and degrades the Tie2 extracellular portion that contains the ligand-binding domain. Epithin is located in the neighbor of Tie2-expressing vessels in normal tissue. Furthermore, epithin can cleave and degrade Tie2 not only in the same cell but also from neighboring cells nearby, resulting in the degradation of the Tie2 ectodomain. The remaining Tie2 fragment was highly phosphorylated and was able to recruit a downstream effector, phosphatidylinositol 3-kinase. Knocking down epithin expression using short hairpin RNA in thymoma cell severely impaired the   migration   through endothelial cells that show the actin rearrangement during the process. The diminution of epithin protein expression in 4T1 breast cancer cells caused the significant decrease in the number of transendothelial migrating cells in vitro as well as in those of metastasizing tumor nodules in vivo, Therefore, we propose that epithin, which regulates endothelial Tie2 functions, plays a critical role in the fine tuning of transendothelial   migration   for normal and cancer cells. 
 Epithin/PRSS14 proteolytically regulates angiopoietin receptor Tie2 during transendothelial   migration  . Epithin/PRSS14, a type II transmembrane serine protease, is involved in normal epithelial development and tumor progression. Here we report, as an interacting substrate of epithin, a receptor tyrosine kinase Tie2 that is well known for important roles in the vessel stability. Epithin interacts with and degrades the Tie2 extracellular portion that contains the ligand-binding domain. Epithin is located in the neighbor of Tie2-expressing vessels in normal tissue. Furthermore, epithin can cleave and degrade Tie2 not only in the same cell but also from neighboring cells nearby, resulting in the degradation of the Tie2 ectodomain. The remaining Tie2 fragment was highly phosphorylated and was able to recruit a downstream effector, phosphatidylinositol 3-kinase. Knocking down epithin expression using short hairpin RNA in thymoma cell severely impaired the   migration   through endothelial cells that show the actin rearrangement during the process. The diminution of epithin protein expression in 4T1 breast cancer cells caused the significant decrease in the number of transendothelial migrating cells in vitro as well as in those of metastasizing tumor nodules in vivo, Therefore, we propose that epithin, which regulates endothelial Tie2 functions, plays a critical role in the fine tuning of transendothelial   migration   for normal and cancer cells. 
 Androgen receptor non-nuclear regulation of prostate cancer cell invasion mediated by Src and matriptase. Castration-resistant prostate cancers still depend on nuclear androgen receptor (AR) function despite their lack of dependence on exogenous androgen. Second generation anti-androgen therapies are more efficient at blocking nuclear AR; however resistant tumors still develop. Recent studies indicate Src is highly active in these resistant tumors. By manipulating AR activity in several different prostate cancer cell lines through RNAi, drug treatment, and the use of a nuclear-deficient AR mutant, we demonstrate that androgen acting on cytoplasmic AR rapidly stimulates Src tyrosine kinase via a non-genomic mechanism. Cytoplasmic AR, acting through Src enhances laminin integrin-dependent invasion. Active Matriptase, which cleaves laminin, is elevated within minutes after androgen stimulation, and is subsequently shed into the medium. Matriptase activation and shedding induced by cytoplasmic AR is dependent on Src. Concomitantly, CDCP1/gp140, a Matriptase and Src substrate that controls integrin-based   migration  , is activated. However, only inhibition of Matriptase, but not CDCP1, suppresses the AR/Src-dependent increase in invasion. Matriptase, present in conditioned medium from AR-stimulated cells, is sufficient to enhance invasion in the absence of androgen. Thus, invasion is stimulated by a rapid but sustained increase in Src activity, mediated non-genomically by cytoplasmic AR, leading to rapid activation and shedding of the laminin protease Matriptase. 
 Genetic reduction of matriptase-1 expression is associated with a reduction in the aggressive phenotype of prostate cancer cells in vitro and in vivo. Matriptase-1 has been implicated as playing an important role in various types of cancer progression through many different cancer related pathways. In the current study we assessed the efficacy of targeting matriptase-1 using ribozyme technology in vitro and in vivo.Matriptase-1 expression was reduced in the PC-3 and DU-145 cell line using hammerhead ribozyme transgenes. In vitro assays were set up to assess changes in growth, invasion, adhesion and   migration   in these cells. In vivo tumour development model was also used to examine the efficacy of targeting matriptase-1 in a living environment.The in vitro results suggest an overall reduction in the aggressive nature of the two cell lines (PC-3 and DU-145) when matriptase-1 levels are reduced, with properties such as growth, invasiveness and   migration   all being reduced (in most cases a greater than 50% reduction in   migration   and invasion compared to the control was observed), though strangely an increase in adhesion is seen in the PC-3 knockout. The in vitro data is strongly backed up by the results of the in vivo work which demonstrates matriptase-1 deficient cells have a substantially reduced ability to grow and develop in vivo compared to control cells when explanted into nude mice, with significant differences in growth and development (P < or = 0.05) being seen after 7 days, and highly significant differences (p < or = 0.001) after 15 days.Together this data strongly implicates matriptase-1 as playing a vital role in the aggressive nature and progression of prostate cancer. 
 The HIV-1 gp41 ectodomain is cleaved by matriptase to produce a chemotactic peptide that acts through FPR2. Several aspects of HIV-1 virulence and pathogenesis are mediated by the envelope protein gp41. Additionally, peptides derived from the gp41 ectodomain have been shown to induce chemotaxis in monocytes and neutrophils. Whereas this chemotactic activity has been reported, it is not known how these peptides could be produced under biological conditions. The heptad repeat 1 (HR1) region of gp41 is exposed to the extracellular environment and could therefore be susceptible to proteolytic processing into smaller peptides. Matriptase is a serine protease expressed at the surface of most epithelia, including the prostate and mucosal surfaces. Here, we present evidence that matriptase efficiently cleaves the HR1 portion of gp41 into a 22-residue chemotactic peptide MAT-1, the sequence of which is highly conserved across HIV-1 clades. We found that MAT-1 induced   migration   of primary neutrophils and monocytes, the latter of which act as a cellular reservoir of HIV during early stage infection. We then used formyl peptide receptor 1 (FPR1) and FPR2 inhibitors, along with HEK 293 cells, to demonstrate that MAT-1 can induce chemotaxis specifically using FPR2, a receptor found on the surface of monocytes, macrophages and neutrophils. These findings are the first to identify a proteolytic cleavage product of gp41 with chemotactic activity and highlight a potential role for matriptase in HIV-1 transmission and infection at epithelial surfaces and within tissue reservoirs of HIV-1. 
 ST14 (suppression of tumorigenicity 14) gene is a target for miR-27b, and the inhibitory effect of ST14 on cell growth is independent of miR-27b regulation. MicroRNAs are noncoding, endogenous small RNAs that regulate target genes by cleavage of the targeted mRNA or translational repression. We investigated the microRNAome using 2-color microarrays in a highly invasive human breast cancer cell line, MDA-MB-231 (subline 4175) and a noninvasive breast epithelial cell line, MCF10A. We found 13 microRNAs that were up-regulated, and nine were down-regulated significantly in 4175 cells (p < 0.05, -fold change >2) compared with MCF10A cells. Interestingly, miR-27b and its putative target gene, ST14 (suppressor of tumorigenicity 14), had inverse expression pattern in breast cancer cells. The 3'-untranslated region of ST14 contains a regulatory element for miR-27b, and our luciferase experiments indicate that antisense miR-27b enhances ST14 expression in cancer cells. Furthermore, antagomir of miR-27b suppressed cell invasion in 4175 cells, whereas pre-miR-27b stimulated invasion in moderately invasive ZR75 breast cancer cells. In addition, ST14 reduces cell proliferation as well as cell   migration   and invasion. Analysis of human breast tumors revealed that miR-27b expression increases during cancer progression, paralleling a decrease in ST14 expression. Furthermore, our data indicate that ST14 inhibits cells from entering into S phase by up-regulating p27, which results in down-regulation of cyclin E-CDK2 complexes, suggesting ST14 reduces cell growth through its effects on cell cycle-related proteins. Introduction of miR-27b into ST14-expressing cells did not suppress the effect on cell growth. These findings suggest that ST14 plays an important role in several biological processes, and some effects are not completely dependent on miR-27b regulation. 
 Matriptase activation and shedding through PDGF-D-mediated extracellular acidosis. Activation of β-platelet-derived growth factor receptor (β-PDGFR) is associated with prostate cancer (PCa) progression and recurrence after prostatectomy. Analysis of the β-PDGFR ligands in PCa revealed association between PDGF-D expression and Gleason score as well as tumor stage. During the course of studying the functional consequences of PDGF ligand-specific β-PDGFR signaling in PCa, we discovered a novel function of PDGF-D for activation/shedding of the serine protease matriptase leading to cell invasi  on, migra  tion, and tumorigenesis. The present study showed that PDGF-D, not PDGF-B, induces extracellular acidification, which correlates with increased matriptase activation. A cDNA microarray analysis revealed that PDGF-D/β-PDGFR signaling upregulates expression of the acidosis regulator carbonic anhydrase IX (CAIX), a classic target of the transcriptional factor hypoxia-inducible factor-1α (HIF-1α). Cellular fractionation displayed a strong HIF-1α nuclear localization in PDGF-D-expressing cells. Treatment of vector control or PDGF-B-expressing cells with the HIF-1α activator CoCl2 led to increased CAIX expression accompanied by extracellular acidosis and matriptase activation. Furthermore, the analysis of the CAFTD cell lines, variants of the BPH-1 transformation model, showed that increased PDGF-D expression is associated with enhanced HIF-1α activity, CAIX induction, cellular acidosis, and matriptase shedding. Importantly, shRNA-mediated knockdown of CAIX expression effectively reversed extracellular acidosis and matriptase activation in PDGF-D-transfected BPH-1 cells and in CAFTD variants that express endogenous PDGF-D at a high level. Taken together, these novel findings reveal a new paradigm in matriptase activation involving PDGF-D-specific signal transduction leading to extracellular acidosis. 
 Decreasing the ratio of matriptase/HAI‑1 by downregulation of matriptase as a potential adjuvant therapy in ovarian cancer. Tumor invasion and metastasis are complex biological processes. Matriptase and its endogenous inhibitor, hepatocyte growth factor activator inhibitor‑1 (HAI‑1) are involved in invasion and metastasis. To evaluate the ratio of matriptase/HAI‑1 and their potential therapeutic value in ovarian cancer, HO‑8910 human ovarian cancer cells and the homologous high‑metastatic HO‑8910PM cells were used as in vitro cellular models ovarian cancer. The invasive and metastatic abilities, and the expression of matriptase and HAI‑1 in these cells were detected using scratch assays, Transwell chamber assays, reverse transcription‑quantitative polymerase chain reaction, western blotting and fluorescent immunocytochemistry. Following infection with lentivirus‑mediated matriptase‑targeting small interfering RNA (siRNA), cell cycle progression and apoptosis w  ere also   analyzed. The migration distance and number of invading HO‑8910PM cells were significantly increased compared with HO‑8910 cells. HO‑8910PM cells exhibited a significantly higher ratio of matriptase/HAI‑1 mRNA levels compared with HO‑8910 cells (0.51 vs. 0.24, ~2.2 fold increase). Compared with HO‑8910 cells, the matriptase mRNA level was increased by ~3.6 fold in HO‑8910PM cells, whereas the HAI‑1 mRNA level was increased by ~1.7 fold. Similar increases in protein expression levels were also observed in H  O‑8910P  M cells compared with HO‑8910 cells. Migration and invasiveness were positively correlated with matriptase expression level (r=0.994, P<0.01) and the ratio of matriptase/HAI‑1 (r=0.929, P<0.01). Downregulation of matriptase using siRNA resulted in inhibition of the invasive and metastatic abilities of HO‑8910PM cells, cell cycle arrest in the G0/G1 phase and increased apoptosis. The present study demonstrated that ovarian cancer cell metastasis and invasion were more dependent on upregulation of matriptase levels than downregulation of HAI‑1. Matriptase may be a potential adjuvant therapeutic target for inhibiting ovarian cancer invasion and metastasis. 
 Effect of SNC19/ST14 gene overexpression on invasion of colorectal cancer cells. To study the effect of SNC19/ST14 gene overexpression on invasion in vitro of colorectal cancer cells.The adhesion of SNC19/ST14 gene-transfected cells to ECM was measured by MTT assay. The cell movement was evaluated by wound healing assay. Cell invasion and   migration   were determined by invasion assay in vitro.SNC19/ST14 gene overexpression could enhance invasion of colorectal cancer cells in vitro significantly and influence early cell adherence to ECM, but could not change cell movement significantly.SNC19/ST14 gene overexpression increases the local invasion of colorectal cancer cells in vitro. 
 Activation of hepatocyte growth factor and urokinase/plasminogen activator by matriptase, an epithelial membrane serine protease. Matriptase is an epithelial-derived, integral membrane serine protease. The enzyme was initially isolated from human breast cancer cells and has been implicated in breast cancer invasion and metastasis. In the current study, using active matriptase isolated from human milk, we demonstrate that matriptase is able to cleave various synthetic substrates with arginine or lysine as their P1 sites and prefers small side chain amino acids, such as Ala and Gly, at P2 sites. For the most reactive substrates, N-tert-butoxycarbonyl (N-t-Boc)-gamma-benzyl-Glu-Ala-Arg-7-amino-4-methylcoumarin (AMC) and N-t-Boc-Gln-Ala-Arg-AMC, the K(m) values were determined to be 3. 81 and 4.89 microm, respectively. We further demonstrated that matriptase can convert hepatocyte growth factor/scattering factor to its active form, which can induce scatter of Madin-Darby canine kidney epithelial cells and can activate c-Met tyrosine phosphorylation in A549 human lung carcinoma cells. In addition, we noted that matriptase can activate urokinase plasminogen activator but has no affect on plasminogen. These results suggest that matriptase could act as an epithelial, upstream membrane activator to recruit and activate stromal-derived downstream effectors important for extracellular matrix degradation and epithelial   migration  , two major events of tissue remodeling, cancer invasion, and metastasis. 
 Soluble epithin/PRSS14 secreted from cancer cells contains active angiogenic potential. Epithin (PRSS14/matriptase/ST14), a type II membrane protein, is involved in progression of epithelial cancers and metastasis as well as in the normal epidermal barrier function. When activated, it translocates into the cell-cell contacts and sheds into media. In order to understand the specific mechanism during tumor progression, we tested the angiogenic potential of secreted form of epithin. Epithin produced from the cancer cells shed more in hypoxia and induced motility of endothelial cells. Epithin enhanced the   migration   and invasion of mouse and bovine endothelial cells without cell proliferation. Furthermore, soluble epithin induced endothelial differentiation in the assay of the human endothelial microvessel-like tube formation and in that of the chicken chorioallantoic membrane. The knock-down of epithin in the 427 thymoma cell line abolished the protease activity of secreted epithin fraction, reduced the invasion of endothelial cells through matrigel, and tube formation activity. Only specific antibodies abolished the   migration   of endothelial cell and the vessel morphogenesis, suggesting that epithin specifically functions in these systems. Therefore, we propose that the secreted epithin in the hypoxic cancer microenvironment plays a role as a proangiogenic factor, and can be modulated with specific antibodies. 
 Matriptase is involved in ErbB-2-induced prostate cancer cell invasion. Deregulation of both ErbB-2 signaling and matriptase activity has been associated with human prostate cancer (PCa) progression. In this communication, we investigated the roles of both ErbB-2 signaling in matriptase zymogen activation and matriptase in ErbB-2-induced PCa malignancy. In a human PCa cell progression model, we observed that advanced PCa C-81 LNCaP cells exhibited an aggressive phenotype with increased cell   migration   and invasion capacity; these cells concurrently showed both enhanced ErbB-2 phosphorylation and increased matriptase zymogen activation compared with parental C-33 LNCaP cells. Moreover, ErbB2 activation, both ligand-dependent (eg, epidermal growth factor treatment) and ligand-independent (eg, overexpression), was able to induce matriptase zymogen activation in this cell line. Inhibition of ErbB-2 activity by either the specific inhibitor, AG825, in epidermal growth factor-treated C-33 LNCaP cells or ErbB-2 knockdown in C-81 LNCaP cells, reduced matriptase activation. These observations were confirmed by similar studies using both DU145 and PC3 cells. Together, these data suggest that ErbB-2 signaling plays an important role in matriptase zymogen activation. ErbB-2-enhanced matriptase activation was suppressed by a phosphatidylinositol 3-kinase inhibitor (ie, LY294002) but not by a MEK inhibitor (ie, PD98059). Suppression of matriptase expression by small hairpin RNA knockdown in ErbB-2-overexpressing LNCaP cells dramatically suppressed cancer cell invasion. In summary, our data indicate that ErbB-2 signaling via the phosphatidylinositol 3-kinase pathway results in up-regulated matriptase zymogen activity, which contributes to PCa cell invasion. 
 Expression of matriptase correlates with tumour progression and clinical prognosis in oral squamous cell carcinoma. To investigate the relationship of matriptase expression in oral squamous cell carcinoma (OSCC) to clinicopathological characteristics, patient survival and cell-invasive properties.Matriptase expression in OSCC was evaluated by immunohistochemical staining, and its relationship to clinicopathological features and outcomes was assessed statistically. The shRNA-mediated stable knockdown of matriptase in OSCC cells was used to analyse cell proliferation,   migration   and invasion in vitro. Matriptase immunostaining score was correlated with histopathological grade, clinical stage, positive lymph node and distant metastasis, and higher matriptase immunostaining score was associated significantly with poor prognosis. Elevated matriptase expression in oral cancer cell lines was a significant promoter of oral cancer cell   migration   and invasion.Matriptase expression correlates with tumour progression and invasive capability in OSCC and may be an adverse prognostic marker for this cancer. 
 Matriptase and its putative role in cancer. Tumor progression and metastasis are the pathologic effects of uncontrolled or deregulated invasive growth, a process in which proteases play a fundamental role. They mediate the degradation of extracellular matrix components and intercellular cohesive structures to allow   migration   of the cells into the extracellular environment and activate growth and angiogenic factors. In addition to metalloproteases and the plasminogen activation system, another protease, matriptase, contributes substantially to these processes. Matriptase is a type II transmembrane trypsin-like serine protease that is expressed by cells of epithelial origin and is overexpressed in a variety of human cancers. It has been suggested that this protease not only facilitates cellular invasiveness but may also activate oncogenic pathways. This review summarizes current knowledge about matriptase, its putative role in tumor initiation and progression, and its potential as a novel target in anti-cancer therapy. 
  GeneRIFs  
 Epithin, which regulates endothelial Tie2 functions, plays a critical role in the fine tuning of transendothelial   migration   for normal and cancer cells. 
  ECM  PubMed  
 Effect of SNC19/ST14 gene overexpression on invasion of colorectal cancer cells. To study the effect of SNC19/ST14 gene overexpression on invasion in vitro of colorectal cancer cells.The adhesion of SNC19/ST14 gene-transfected cells to   ECM   was measured by MTT assay. The cell movement was evaluated by wound healing assay. Cell invasion and migration were determined by invasion assay in vitro.SNC19/ST14 gene overexpression could enhance invasion of colorectal cancer cells in vitro significantly and influence early cell adherence to   ECM  , but could not change cell movement significantly.SNC19/ST14 gene overexpression increases the local invasion of colorectal cancer cells in vitro. 
 [SNC19/ST14 gene transfection and expression influence the biological behavior of colorectal cancer cells]. To study the effect of SNC19/ST14 gene transfection and expression into colorectal cancer cells on biological behavior.The recombination vector pSecTag2a-SNC19/ST14 was constructed, and transfected into the RKO colorectal cancer cell line by liposome. The transfected cell was screened by real time PCR, Western Blot and Immunohistochemical technique. The population doubling time (T(D)) and cell cycle of the transfected cell were analyzed by MTT assay and flow cytometer. Rhodamine-labeled Phallodin was used to label the cell cytoskeletal protein-F-actin, and the F-actin distribution was observed by confocal scanning microscope. The adhesion ability of the transfected cell to extracellular matrix (  ECM  ) was measured by MTT assay.The full length Open Reading Frame (ORF) of SNC19/ST14 gene was inserted into the vector pSecTag2a, and transfected into RKO cells, and expressed successfully. The changes of F-actin organization took place in transfected cell. The Adherence ability of the transfected cell to   ECM   was decreased, but the proliferation ability was not significantly changed except highly expressing of the SNC19/ST14, and not the cell cycle and the apoptosis.SNC19/ST14 gene was successfully expressed into the RKO cell line, and could influence on the cell cytoskeletal protein (F-actin) organization and on cell adherence ability to   ECM  , but could not make the cell cycle, apoptosis and proliferation ability change significantly. 
  GeneRIFs  
 SNC19/ST14 gene overexpression could enhance invasion of colorectal cancer cells in vitro significantly and influence early cell adherence to   ECM  , but could not change cell movement significantly. 
  [Ee]xtracellular [Mm]atrix  PubMed  
 Matriptase and HAI-1 are expressed by normal and malignant epithelial cells in vitro and in vivo. Matriptase and its cognate, Kunitz-type serine protease inhibitor, HAI-1, comprise a newly characterized   extracellular matrix  -degrading protease system that may function as an epithelial membrane activator for other proteases and latent growth factors. Both enzyme and inhibitor have been detected in breast cancer cells, immortalized mammary epithelial cells, and human milk, but not in cultured fibroblasts nor in fibrosarcoma cells. To test the hypothesis that this system is expressed by normal breast epithelium, invasive breast cancers, and other cancers of an epithelial origin (carcinomas) but not in cancers of a mesenchymal origin, we have expanded our expression analysis of matriptase and HAI-1 in vitro and in vivo. Matriptase and HAI-1 were detected at the protein and mRNA levels both in hormone-dependent and hormone-independent cultured breast cancer cells, and this expression correlated with the expression of the epithelial markers E-cadherin or ZO-1. However, none of the breast cancer cell lines tested that express the mesenchymal marker vimentin express matriptase or HAI-1, consistent with an epithelial-selective expression of this system. Expression of matriptase, as determined by Western blot analysis, was observed in primary human breast, gynecological, and colon carcinomas, but not in stromal-derived ovarian tumors and human sarcomas of various origins and histological grades. The epithelial-selective expression of matriptase and HAI-1 was further confirmed in human breast cancers by immunohistochemistry and in situ hybridization, where the expression of the protease and the inhibitor were found in the carcinoma cells and in surrounding normal breast epithelia. The expression of the matriptase/HAI-1 system by malignant epithelial cells in vivo suggests a possible role for this protease in multiple aspects of the pathophysiology of epithelial malignancy, including invasion and metastasis. 
 Strong expression association between matriptase and its substrate prostasin in breast cancer. Breast cancer tumorigenesis is accompanied by increased levels of extracellular proteases that are capable of remodeling the   extracellular matrix   as well as cleaving and activating growth factors and signaling receptors that are critically involved in neoplastic progression. Multiple studies implicate the membrane anchored serine protease matriptase (also known as MT-SP1 and epithin) in breast cancer. The pro-form of the GPI-anchored serine protease prostasin has recently been identified as a physiological substrate of matriptase and the two proteases are co-expressed in multiple healthy tissues. In this study, the inter-relationship between the two membrane-anchored serine proteases in breast cancer was investigated using breast cancer cell lines and breast cancer patient samples to delineate the association between matriptase and prostasin. We used Western blotting to determine the expression of matriptase and prostasin proteins in a panel of breast cancer cell lines and immunohistochemistry to assess the expression in serial sections from breast cancer tissue arrays. We demonstrate that the expression of matriptase and prostasin is closely correlated in breast cancer cell lines as well as in breast cancer tissue samples. Furthermore, matriptase and prostasin display a near identical spatial expression pattern in the epithelial compartment of breast cancer tissue. These data suggest that the matriptase-prostasin cascade might play a critical role in breast cancer. 
 Inhibition of tumor invasion by genomic down-regulation of matriptase through suppression of activation of receptor-bound pro-urokinase. Urokinase-type plasminogen activator (uPA) degrades the   extracellular matrix   and plays critical roles in tumor invasion and metastasis. Matriptase, a membrane-bound serine protease, was shown to activate uPA in a uPA receptor-free, solution-based study. We now investigate whether matriptase affects activation of receptor-bound uPA and contributes to the invasiveness of HRA human ovarian cancer cells in vitro and tumor behavior in nude mice. Here we show the following. 1) uPA expression was effectively stimulated by TGF-beta1 in HRA cells. 2) Antisense (AS)-matriptase transfection achieved a marked inhibition of receptor-bound pro-uPA activation without altering expression of uPA and uPA receptor mRNA and proteins, irrespective of whether cells were stimulated with TGF-beta1. 3) Tumor cell receptor-bound pro-uPA could be efficiently cleaved by matriptase to generate enzymatically active two-chain uPA. Thus, matriptase can substitute for plasmin in the proteolytic activation of pro-uPA to enzymatically active uPA. 4) The AS-matriptase-treated cells had a decreased ability to invade an   extracellular matrix   layer, as compared with control cells. 5) When the AS-matriptase-treated cells were injected intraperitoneally into nude mice, the mice developed smaller tumors. Our data identify a novel role for matriptase for activation of receptor-bound uPA. 
 The novel serine protease tumor-associated differentially expressed gene-15 (matriptase/MT-SP1) is highly overexpressed in cervical carcinoma. Tumor-associated differentially expressed gene-15 (TADG-15/matriptase/MT-SP1) is a novel transmembrane serine protease involved in numerous biologic processes, including activation of growth and angiogenic factors and degradation of   extracellular matrix   components. To assess the value of TADG-15 as a possible marker for tumor detection and/or as a target for therapeutic intervention, the authors investigated the frequency of expression of TADG-15 in human cervical tumors.TADG-15 expression was evaluated in 19 cervical carcinoma cell lines (i.e., 11 primary tumor cell lines and 8 established cell lines) and in 8 normal cervical keratinocyte control cultures using reverse transcriptase-polymerase chain reaction (RT-PCR). In addition, to validate gene expression data at the protein level, TADG-15 expression was evaluated by immunohistochemistry on paraffin embedded tissue from which all 11 primary tumor cell lines were established.TADG-15 was expressed at high levels in 8 of 11 (73%) primary cervical carcinoma cell lines and in 6 of 8 (75%) established cervical carcinoma cell lines by RT-PCR. Expression of TADG-15 was found in 6 of 6 (100%) primary squamous cell cervical carcinomas, whereas 2 of 5 (40%) primary adenocarcinomas expressed TADG-15. In contrast, none of the normal cervical keratinocyte control cultures (n = 4) or flash-frozen normal cervical biopsy specimens (n = 4) expressed TADG-15. Immunohistochemistry staining of paraffin embedded cervical carcinoma specimens confirmed TADG-15 expression in tumor cells and its absence on normal cervical epithelial cells.Cervical carcinoma cells expressed high levels of TADG-15, suggesting that this protease may play an important role in invasion and metastasis. Because TADG-15 appears only in abundance in squamous tumor tissue and contains a proteolytic cleavage site, suggesting that the TADG-15 protease domain is released, it may prove to be a useful diagnostic tool for the early detection of recurrent/persistent cervical carcinoma after standard treatment or as a novel molecular target for therapy in patients with cervical carcinoma. 
 Activation of hepatocyte growth factor and urokinase/plasminogen activator by matriptase, an epithelial membrane serine protease. Matriptase is an epithelial-derived, integral membrane serine protease. The enzyme was initially isolated from human breast cancer cells and has been implicated in breast cancer invasion and metastasis. In the current study, using active matriptase isolated from human milk, we demonstrate that matriptase is able to cleave various synthetic substrates with arginine or lysine as their P1 sites and prefers small side chain amino acids, such as Ala and Gly, at P2 sites. For the most reactive substrates, N-tert-butoxycarbonyl (N-t-Boc)-gamma-benzyl-Glu-Ala-Arg-7-amino-4-methylcoumarin (AMC) and N-t-Boc-Gln-Ala-Arg-AMC, the K(m) values were determined to be 3. 81 and 4.89 microm, respectively. We further demonstrated that matriptase can convert hepatocyte growth factor/scattering factor to its active form, which can induce scatter of Madin-Darby canine kidney epithelial cells and can activate c-Met tyrosine phosphorylation in A549 human lung carcinoma cells. In addition, we noted that matriptase can activate urokinase plasminogen activator but has no affect on plasminogen. These results suggest that matriptase could act as an epithelial, upstream membrane activator to recruit and activate stromal-derived downstream effectors important for   extracellular matrix   degradation and epithelial migration, two major events of tissue remodeling, cancer invasion, and metastasis. 
 Molecular cloning of cDNA for matriptase, a matrix-degrading serine protease with trypsin-like activity. A major protease from human breast cancer cells was previously detected by gelatin zymography and proposed to play a role in breast cancer invasion and metastasis. To structurally characterize the enzyme, we isolated a cDNA encoding the protease. Analysis of the cDNA reveals three sequence motifs: a carboxyl-terminal region with similarity to the trypsin-like serine proteases, four tandem cysteine-rich repeats homologous to the low density lipoprotein receptor, and two copies of tandem repeats originally found in the complement subcomponents C1r and C1s. By comparison with other serine proteases, the active-site triad was identified as His-484, Asp-539, and Ser-633. The protease contains a characteristic Arg-Val-Val-Gly-Gly motif that may serve as a proteolytic activation site. The bottom of the substrate specificity pocket was identified to be Asp-627 by comparison with other trypsin-like serine proteases. In addition, this protease exhibits trypsin-like activity as defined by cleavage of synthetic substrates with Arg or Lys as the P1 site. Thus, the protease is a mosaic protein with broad spectrum cleavage activity and two potential regulatory modules. Given its ability to degrade   extracellular matrix   and its trypsin-like activity, the name matriptase is proposed for the protease. 
 Purification and characterization of a complex containing matriptase and a Kunitz-type serine protease inhibitor from human milk. Matriptase, a trypsin-like serine protease with two potential regulatory modules (low density lipoprotein receptor and complement C1r/s domains), was initially purified from T-47D breast cancer cells. Given its plasma membrane localization,   extracellular matrix  -degrading activity, and expression by breast cancer cells, this protease may be involved in multiple aspects of breast tumor progression, including cancer invasion. In breast cancer cells, matriptase was detected mainly as an uncomplexed form; however, low levels of matriptase were detected in complexes. In striking contrast, only the complexed matriptase was detected in human milk. The complexed matriptase has now been purified. Amino acid sequences obtained from the matriptase-associated proteins reveal that they are fragments of a Kunitz-type serine protease inhibitor that was previously reported to be an inhibitor of the hepatocyte growth factor activator. In addition, matriptase and its complexes were detected in milk-derived, SV40 T-antigen-immortalized mammary luminal epithelial cell lines, but not in human foreskin fibroblasts or in HT-1080 fibrosarcoma cells. These results suggest that the milk-derived matriptase complexes are likely to be produced by the epithelial components of the lactating mammary gland in vivo and that the activity and function of matriptase may be differentially regulated by its cognate inhibitor, comparing breast cancer with the lactating mammary gland. 
 Expression of matriptase and clinical outcome of human endometrial cancer. Matriptase, a type II transmembrane serine protease is involved in angiogenesis, degradation of   extracellular matrix   and in progression of some epithelial cancers. The purpose of the present study was to examine matriptase expression and evaluate its clinicopathological significance in endometrial cancer.Matriptase expression was examined in normal endometrium (n=20), endometrial hyperplasia (n=11) and endometrial cancer (n=65) samples. The distribution of cases that scored positive for each of the biological parameters examined was correlated with matriptase expression status obtained by immunohistochemistry.Matriptase was found to be significantly overexpressed in endometrial cancer as compared with normal endometrium and endometrial hyperplasia. Interestingly, matriptase expression is associated with stage (p=0.010), grade (p=0.021), depth of myometrial invasion (p=0.004), cervical involvement (p=0.021), lymph node metastasis (p=0.009), LVS involvement (p=0.041) and peritoneal cytology (p=0.045). The high matriptase expression was a significant predictor for poor prognosis when compared with low matriptase expression (Disease-free survival rate; p=0.032, Overall survival rate; p=0.011).High matriptase expression in endometrial cancer may be associated with poor prognosis. 
 [SNC19/ST14 gene transfection and expression influence the biological behavior of colorectal cancer cells]. To study the effect of SNC19/ST14 gene transfection and expression into colorectal cancer cells on biological behavior.The recombination vector pSecTag2a-SNC19/ST14 was constructed, and transfected into the RKO colorectal cancer cell line by liposome. The transfected cell was screened by real time PCR, Western Blot and Immunohistochemical technique. The population doubling time (T(D)) and cell cycle of the transfected cell were analyzed by MTT assay and flow cytometer. Rhodamine-labeled Phallodin was used to label the cell cytoskeletal protein-F-actin, and the F-actin distribution was observed by confocal scanning microscope. The adhesion ability of the transfected cell to   extracellular matrix   (ECM) was measured by MTT assay.The full length Open Reading Frame (ORF) of SNC19/ST14 gene was inserted into the vector pSecTag2a, and transfected into RKO cells, and expressed successfully. The changes of F-actin organization took place in transfected cell. The Adherence ability of the transfected cell to ECM was decreased, but the proliferation ability was not significantly changed except highly expressing of the SNC19/ST14, and not the cell cycle and the apoptosis.SNC19/ST14 gene was successfully expressed into the RKO cell line, and could influence on the cell cytoskeletal protein (F-actin) organization and on cell adherence ability to ECM, but could not make the cell cycle, apoptosis and proliferation ability change significantly. 
 Matriptase and its putative role in cancer. Tumor progression and metastasis are the pathologic effects of uncontrolled or deregulated invasive growth, a process in which proteases play a fundamental role. They mediate the degradation of   extracellular matrix   components and intercellular cohesive structures to allow migration of the cells into the extracellular environment and activate growth and angiogenic factors. In addition to metalloproteases and the plasminogen activation system, another protease, matriptase, contributes substantially to these processes. Matriptase is a type II transmembrane trypsin-like serine protease that is expressed by cells of epithelial origin and is overexpressed in a variety of human cancers. It has been suggested that this protease not only facilitates cellular invasiveness but may also activate oncogenic pathways. This review summarizes current knowledge about matriptase, its putative role in tumor initiation and progression, and its potential as a novel target in anti-cancer therapy. 
  GeneRIFs  
 SNC19/ST14 could influence on the cell cytoskeletal protein (F-actin) organization and on cell adherence ability to   extracellular matrix  . 
  [Ii]nvasive  PubMed  
 Cleavage of hepatocyte growth factor activator inhibitor-1 by membrane-type MMP-1 activates matriptase. Co-expression of membrane-type 1 (MT1)-MMP with hepatocyte growth factor activator inhibitor-1 (HAI-1) in HEK293T cells resulted in cleavage of HAI-1 to produce three fragments. Recombinant MT1-MMP was shown to cleave HAI-1 protein in vitro. Hepatocyte growth factor activator inhibitor-1 was initially identified as the cognate inhibitor of matriptase, a transmembrane serine protease that processes urokinase-type plasminogen activator (uPA). Co-expression of HAI-1 with matriptase suppressed matriptase protease activity, and co-expression of MT1-MMP with them resulted in recovery of matriptase activity by stimulating shedding of HAI-1 fragments. Matriptase protein was detected in squamous carcinoma-derived HSC-4 cells, however, matriptase protease activity was undetectable. Transfection of siRNA for HAI-1 enhanced serine protease activity, which was suppressed by cotransfection of matriptase siRNA. Collagen-gel culture or treatment with concanavalin A (ConA) of HSC-4 cells enhanced MT1-MMP activity, which induced shedding of HAI-1 fragments and conversely stimulated uPA activation by these cells. Serine protease activity, including uPA activation of cells treated with ConA, was abrogated by downregulation of either matriptase or MT1-MMP through the transfection of each siRNA. These results suggest that MT1-MMP induced by collagen-gel culture or ConA treatment causes cleavage and shedding of HAI-1 protein, which allows activation of matriptase in HSC-4 cells. HSC-4 cells showed a characteristic   invasive   growth by forming vacuole-like structures in collagen gel, which was suppressed by transfection of siRNA for either MT1-MMP or matriptase, suggesting that activation of matriptase through the cleavage of HAI-1 is one of the MT1-MMP multifunctions essential for   invasive   growth of HSC-4 cells. 
 Cleavage of hepatocyte growth factor activator inhibitor-1 by membrane-type MMP-1 activates matriptase. Co-expression of membrane-type 1 (MT1)-MMP with hepatocyte growth factor activator inhibitor-1 (HAI-1) in HEK293T cells resulted in cleavage of HAI-1 to produce three fragments. Recombinant MT1-MMP was shown to cleave HAI-1 protein in vitro. Hepatocyte growth factor activator inhibitor-1 was initially identified as the cognate inhibitor of matriptase, a transmembrane serine protease that processes urokinase-type plasminogen activator (uPA). Co-expression of HAI-1 with matriptase suppressed matriptase protease activity, and co-expression of MT1-MMP with them resulted in recovery of matriptase activity by stimulating shedding of HAI-1 fragments. Matriptase protein was detected in squamous carcinoma-derived HSC-4 cells, however, matriptase protease activity was undetectable. Transfection of siRNA for HAI-1 enhanced serine protease activity, which was suppressed by cotransfection of matriptase siRNA. Collagen-gel culture or treatment with concanavalin A (ConA) of HSC-4 cells enhanced MT1-MMP activity, which induced shedding of HAI-1 fragments and conversely stimulated uPA activation by these cells. Serine protease activity, including uPA activation of cells treated with ConA, was abrogated by downregulation of either matriptase or MT1-MMP through the transfection of each siRNA. These results suggest that MT1-MMP induced by collagen-gel culture or ConA treatment causes cleavage and shedding of HAI-1 protein, which allows activation of matriptase in HSC-4 cells. HSC-4 cells showed a characteristic   invasive   growth by forming vacuole-like structures in collagen gel, which was suppressed by transfection of siRNA for either MT1-MMP or matriptase, suggesting that activation of matriptase through the cleavage of HAI-1 is one of the MT1-MMP multifunctions essential for   invasive   growth of HSC-4 cells. 
 Imaging a functional tumorigenic biomarker in the transformed epithelium. Proteases responsible for the increased peritumoral proteolysis associated with cancer represent functional biomarkers for monitoring tumorigenesis. One attractive extracellular biomarker is the transmembrane serine protease matriptase. Found on the surface of epithelial cells, the activity of matriptase is regulated by its cognate inhibitor hepatocyte growth factor activator inhibitor-1 (HAI-1). Quantitative mass spectrometry allowed us to show that, in selected cancers, HAI-1 expression decreases, leading to active matriptase. A preclinical probe specific for the measurement of emergent active matriptase was developed. Using an active-site-specific, recombinant human antibody for matriptase, we found that the selective targeting of active matriptase can be used to visualize the tumorigenic epithelium. Live-cell fluorescence imaging validated the selectivity of the antibody in vitro by showing that the probe localized only to cancer cell lines with active matriptase on the surface. Immunofluorescence with the antibody documented significant levels of active matriptase in 68% of primary and metastatic colon cancer sections from tissue microarrays. Labeling of the active form of matriptase in vivo was measured in human colon cancer xenografts and in a patient-derived xenograft model using near-infrared and single-photon emission computed tomography imaging. Tumor uptake of the radiolabeled antibody, (111)In-A11, by active matriptase was high in xenografts (28% injected dose per gram) and was blocked in vivo by the addition of a matriptase-specific variant of ecotin. These findings suggest, through a HAI-1-dependent mechanism, that emergent active matriptase is a functional biomarker of the transformed epithelium and that its proteolytic activity can be exploited to non  invasive  ly evaluate tumorigenesis in vivo. 
 Matriptase and HAI-1 are expressed by normal and malignant epithelial cells in vitro and in vivo. Matriptase and its cognate, Kunitz-type serine protease inhibitor, HAI-1, comprise a newly characterized extracellular matrix-degrading protease system that may function as an epithelial membrane activator for other proteases and latent growth factors. Both enzyme and inhibitor have been detected in breast cancer cells, immortalized mammary epithelial cells, and human milk, but not in cultured fibroblasts nor in fibrosarcoma cells. To test the hypothesis that this system is expressed by normal breast epithelium,   invasive   breast cancers, and other cancers of an epithelial origin (carcinomas) but not in cancers of a mesenchymal origin, we have expanded our expression analysis of matriptase and HAI-1 in vitro and in vivo. Matriptase and HAI-1 were detected at the protein and mRNA levels both in hormone-dependent and hormone-independent cultured breast cancer cells, and this expression correlated with the expression of the epithelial markers E-cadherin or ZO-1. However, none of the breast cancer cell lines tested that express the mesenchymal marker vimentin express matriptase or HAI-1, consistent with an epithelial-selective expression of this system. Expression of matriptase, as determined by Western blot analysis, was observed in primary human breast, gynecological, and colon carcinomas, but not in stromal-derived ovarian tumors and human sarcomas of various origins and histological grades. The epithelial-selective expression of matriptase and HAI-1 was further confirmed in human breast cancers by immunohistochemistry and in situ hybridization, where the expression of the protease and the inhibitor were found in the carcinoma cells and in surrounding normal breast epithelia. The expression of the matriptase/HAI-1 system by malignant epithelial cells in vivo suggests a possible role for this protease in multiple aspects of the pathophysiology of epithelial malignancy, including invasion and metastasis. 
 Use of IHC and newly designed matriptase inhibitors to elucidate the role of matriptase in pancreatic ductal adenocarcinoma. Matriptase, also known as MT-SP1, is a type II transmembrane serine protease strongly implicated in both the development and progression of a variety of epithelial cancers. Evidence comes from studies of its expression in human cancers and from mouse models of spontaneous cancer. Matriptase is considered to be a major activator of two key stimulators of   invasive   growth, namely hepatocyte growth factor/scatter factor and urokinase-type plasminogen activator. The aim of this study was to examine the role of matriptase in pancreatic ductal adenocarcinoma by expression analysis and functional assays in vitro. Immunohistochemical analysis of matriptase performed on microtissue arrays and large samples of 55 pancreatic ductal adenocarcinomas and on 31 samples of normal pancreatic ducts revealed that although matriptase expression differed greatly in both malignant and normal ductal pancreatic tissue, matriptase scores were significantly (p=0.02) elevated in pancreatic ductal adenocarcinoma compared to normal pancreatic ducts. To evaluate the role of matriptase during development of pancreatic cancer, we studied the effects of newly designed matriptase inhibitors on the processing of the zymogen of urokinase-type plasminogen activator in the human adenocarcinoma cell lines AsPC-1 and BxPC-3. In both cell lines, at 1 microM, all matriptase inhibitors completely prevented zymogen activation. At lower inhibitor concentrations, the degree of inhibition of zymogen processing correlated with the affinities of the inhibitors towards matriptase indicating that this is a specific result of matriptase inhibition. Furthermore, matriptase inhibitors reduced the phosphorylation of the HGF receptor/cMet and the overall cellular   invasive  ness of the human pancreatic adenocarcinoma cell line AsPC-1. Our findings demonstrate for the first time that matriptase may be involved in the progression of pancreatic ductal adenocarcinoma and that matriptase inhibition may contribute to preventing the progression of this devastating disease. 
 Genetic reduction of matriptase-1 expression is associated with a reduction in the aggressive phenotype of prostate cancer cells in vitro and in vivo. Matriptase-1 has been implicated as playing an important role in various types of cancer progression through many different cancer related pathways. In the current study we assessed the efficacy of targeting matriptase-1 using ribozyme technology in vitro and in vivo.Matriptase-1 expression was reduced in the PC-3 and DU-145 cell line using hammerhead ribozyme transgenes. In vitro assays were set up to assess changes in growth, invasion, adhesion and migration in these cells. In vivo tumour development model was also used to examine the efficacy of targeting matriptase-1 in a living environment.The in vitro results suggest an overall reduction in the aggressive nature of the two cell lines (PC-3 and DU-145) when matriptase-1 levels are reduced, with properties such as growth,   invasive  ness and migration all being reduced (in most cases a greater than 50% reduction in migration and invasion compared to the control was observed), though strangely an increase in adhesion is seen in the PC-3 knockout. The in vitro data is strongly backed up by the results of the in vivo work which demonstrates matriptase-1 deficient cells have a substantially reduced ability to grow and develop in vivo compared to control cells when explanted into nude mice, with significant differences in growth and development (P < or = 0.05) being seen after 7 days, and highly significant differences (p < or = 0.001) after 15 days.Together this data strongly implicates matriptase-1 as playing a vital role in the aggressive nature and progression of prostate cancer. 
 Expression of serine protease SNC19/matriptase and its inhibitor hepatocyte growth factor activator inhibitor type 1 in normal and malignant tissues of gastrointestinal tract. To provide the expression profile of serine protease SNC19/matriptase and its inhibitor hepatocyte growth factor activator inhibitor type 1 (HAI-1) in normal and malignant tissues of gastrointestinal tract at mRNA level for further study on their correlations with tumor progression and metastasis.Total RNAs were prepared from 37 samples of colorectal cancer tissues, 40 samples of gastric cancer tissues, and their adjacent normal tissues. The expression of SNC19/matriptase and HAI-1 in these samples was detected by real-time fluorescent quantitative PCR using glyceraldehyde-3-phosphate dehydrogenase as internal standard, and the clinical significance for the correlation with clinicopathological parameters was evaluated.In gastric cancer tissues the expression of HAI-1 and SNC19/matriptase was significantly lower than that in the corresponding adjacent normal tissues (Z = -3.280, P = 0.006; Z = -4.651, P = 0.000). HAI-1:SNC19/matriptase ratio showed no difference between normal and malignant tissues (P>0.05). Analysis of clinicopathological parameters showed decreased expression of HAI-1 and HAI-1:SNC19/matriptase ratio associated with stage III/IV gastric tumors as compared to stage I/II ones (Z = -2.140, P = 0.031; Z = -2.155, P = 0.031), and with lymph node-positive gastric cancer tissues as compared to lymph node-negative ones (Z = -2.081, P = 0.036; Z = -2.686, P = 0.006). The expression of SNC19/matriptase had no relationship with stages and lymph node metastasis (P>0.05). The expression of HAI-1 and HAI-1:SNC19/matriptase ratio increased in well-differentiated gastric cancer tissues, but there was no statistical significance (P>0.05). The difference of SNC19/matriptase expression was not significant in gastric cancer tissues of different histological differentiation status (P>0.05). In colorectal cancer tissues, the expression of HAI-1 and SNC19/matriptase was also markedly lower than that in their adjacent normal tissues (Z = -3.100, P = 0.002; Z = -2.731, P = 0.006), whereas HAI-1:SNC19/matriptase ratio showed no difference. Decreased expression of HAI-1 was associated with increased   invasive   depth and lymph node metastasis, but there was no statistical significance (P>0.05). The difference of SNC19/matriptase expression and HAI-1:SNC19/matriptase ratio was not significant in different stages and different lymph node metastasis status (P>0.05). The expression of SNC19/matriptase, HAI-1 or HAI-1:SNC19/martiptase ratio showed no difference in colorectal cancer tissues of different histological differentiation status (P>0.05).The expressions of SNC19/matriptase and its inhibitor HAI-1 are decreased in gastrointestinal cancer tissues compared to their normal counterparts, and the decreased expression of HAI-1 may correlate with invasion and lymph node metastasis. The possible mechanisms involved need to be further investigated. 
 Inhibition of tumor invasion by genomic down-regulation of matriptase through suppression of activation of receptor-bound pro-urokinase. Urokinase-type plasminogen activator (uPA) degrades the extracellular matrix and plays critical roles in tumor invasion and metastasis. Matriptase, a membrane-bound serine protease, was shown to activate uPA in a uPA receptor-free, solution-based study. We now investigate whether matriptase affects activation of receptor-bound uPA and contributes to the   invasive  ness of HRA human ovarian cancer cells in vitro and tumor behavior in nude mice. Here we show the following. 1) uPA expression was effectively stimulated by TGF-beta1 in HRA cells. 2) Antisense (AS)-matriptase transfection achieved a marked inhibition of receptor-bound pro-uPA activation without altering expression of uPA and uPA receptor mRNA and proteins, irrespective of whether cells were stimulated with TGF-beta1. 3) Tumor cell receptor-bound pro-uPA could be efficiently cleaved by matriptase to generate enzymatically active two-chain uPA. Thus, matriptase can substitute for plasmin in the proteolytic activation of pro-uPA to enzymatically active uPA. 4) The AS-matriptase-treated cells had a decreased ability to invade an extracellular matrix layer, as compared with control cells. 5) When the AS-matriptase-treated cells were injected intraperitoneally into nude mice, the mice developed smaller tumors. Our data identify a novel role for matriptase for activation of receptor-bound uPA. 
 ST14 (suppression of tumorigenicity 14) gene is a target for miR-27b, and the inhibitory effect of ST14 on cell growth is independent of miR-27b regulation. MicroRNAs are noncoding, endogenous small RNAs that regulate target genes by cleavage of the targeted mRNA or translational repression. We investigated the microRNAome using 2-color microarrays in a highly   invasive   human breast cancer cell line, MDA-MB-231 (subline 4175) and a non  invasive   breast epithelial cell line, MCF10A. We found 13 microRNAs that were up-regulated, and nine were down-regulated significantly in 4175 cells (p < 0.05, -fold change >2) compared with MCF10A cells. Interestingly, miR-27b and its putative target gene, ST14 (suppressor of tumorigenicity 14), had inverse expression pattern in breast cancer cells. The 3'-untranslated region of ST14 contains a regulatory element for miR-27b, and our luciferase experiments indicate that antisense miR-27b enhances ST14 expression in cancer cells. Furthermore, antagomir of miR-27b suppressed cell invasion in 4175 cells, whereas pre-miR-27b stimulated invasion in moderately   invasive   ZR75 breast cancer cells. In addition, ST14 reduces cell proliferation as well as cell migration and invasion. Analysis of human breast tumors revealed that miR-27b expression increases during cancer progression, paralleling a decrease in ST14 expression. Furthermore, our data indicate that ST14 inhibits cells from entering into S phase by up-regulating p27, which results in down-regulation of cyclin E-CDK2 complexes, suggesting ST14 reduces cell growth through its effects on cell cycle-related proteins. Introduction of miR-27b into ST14-expressing cells did not suppress the effect on cell growth. These findings suggest that ST14 plays an important role in several biological processes, and some effects are not completely dependent on miR-27b regulation. 
 ST14 gene variant and decreased matriptase protein expression predict poor breast cancer survival. Matriptase plays a role in carcinogenesis, but the role of its genetic variation or that of the hepatocyte growth factor activator inhibitor-1 (HAI-1) has not been evaluated. This study aimed to examine the genetic variation of matriptase (ST14 gene) and HAI-1 (SPINT1 gene) in breast cancer risk and prognosis, to assess matriptase and HAI-1 gene and protein expression in breast tumors, and to identify their clinicopathologic correlations and prognostic significance.Five single nucleotide polymorphisms in ST14 and three in SPINT1 were genotyped in 470   invasive   breast cancer cases and 446 healthy controls. Gene expression analysis was done for 40 breast cancer samples. Protein expression was assessed by immunohistochemical analyses in 377   invasive   breast tumors. The statistical significance of the associations among genotypes, clinicopathologic variables, and prognosis was assessed.The ST14 single nucleotide polymorphism rs704624 independently predicted breast cancer survival, a poor outcome associated with the minor allele (P = 0.001; risk ratio, 2.221; 95% confidence interval, 1.382-3.568). Moreover, ST14 gene expression levels were lower among the minor allele carriers (P = 0.009), and negative/low matriptase protein expression was independently predictive of poorer survival (P = 0.046; risk ratio, 1.554; 95% confidence interval, 1.008-2.396).The ST14 variant rs704624 and protein expression of matriptase have prognostic significance in breast cancer. This study adds to the evidence for the role of matriptase in breast cancer and has found new evidence for the genotypes having an impact in breast cancer.This is the first study showing that genetic variation in matriptase has clinical importance. The results encourage further study on the genetic variation affecting protein levels and function in type II transmembrane serine proteases. 
 Decreasing the ratio of matriptase/HAI‑1 by downregulation of matriptase as a potential adjuvant therapy in ovarian cancer. Tumor invasion and metastasis are complex biological processes. Matriptase and its endogenous inhibitor, hepatocyte growth factor activator inhibitor‑1 (HAI‑1) are involved in invasion and metastasis. To evaluate the ratio of matriptase/HAI‑1 and their potential therapeutic value in ovarian cancer, HO‑8910 human ovarian cancer cells and the homologous high‑metastatic HO‑8910PM cells were used as in vitro cellular models ovari  an cance  r. The invasive and metastatic abilities, and the expression of matriptase and HAI‑1 in these cells were detected using scratch assays, Transwell chamber assays, reverse transcription‑quantitative polymerase chain reaction, western blotting and fluorescent immunocytochemistry. Following infection with lentivirus‑mediated matriptase‑targeting small interfering RNA (siRNA), cell cycle progression and apoptosis were also analyzed. The migration distance and number of invading HO‑8910PM cells were significantly increased compared with HO‑8910 cells. HO‑8910PM cells exhibited a significantly higher ratio of matriptase/HAI‑1 mRNA levels compared with HO‑8910 cells (0.51 vs. 0.24, ~2.2 fold increase). Compared with HO‑8910 cells, the matriptase mRNA level was increased by ~3.6 fold in HO‑8910PM cells, whereas the HAI‑1 mRNA level was increased by ~1.7 fold. Similar increases in protein expression levels were also observed in HO‑8910PM cel  ls compa  red with HO‑8910 cells. Migration and invasiveness were positively correlated with matriptase expression level (r=0.994, P<0.01) and the ratio of matriptase/HAI‑1 (r=0.929, P<0.01). Downregulation of mat  riptase   using siRNA resulted in inhibition of the invasive and metastatic abilities of HO‑8910PM cells, cell cycle arrest in the G0/G1 phase and increased apoptosis. The present study demonstrated that ovarian cancer cell metastasis and invasion were more dependent on upregulation of matriptase levels than downregulation of HAI‑1. Matriptase may be a potential adjuvant therapeutic target for inhibiting ovarian cancer invasion and metastasis. 
 Curcumin-targeting pericellular serine protease matriptase role in suppression of prostate cancer cell invasion, tumor growth, and metastasis. Curcumin has been shown to possess potent chemopreventive and antitumor effects on prostate cancer. However, the molecular mechanism involved in curcumin's ability to suppress prostate cancer cell invasion, tumor growth, and metastasis is not yet well understood. In this study, we have shown that curcumin can suppress epidermal growth factor (EGF)- stimulated and heregulin-stimulated PC-3 cell invasion, as well as androgen-induced LNCaP cell invasion. Curcumin treatment significantly resulted in reduced matrix metalloproteinase 9 activity and downregulation of cellular matriptase, a membrane-anchored serine protease with oncogenic roles in tumor formation and invasion. Our data further show that curcumin is able to inhibit the induction effects of androgens and EGF on matriptase activation, as well as to reduce the activated levels of matriptase after its overexpression, thus suggesting that curcumin may interrupt diverse signal pathways to block the protease. Furthermore, the reduction of activated matriptase in cells by curcumin was also partly due to curcumin's effect on promoting the shedding of matriptase into an extracellular environment, but not via altering matriptase gene expression. In addition, curcumin significantly suppressed the   invasive   ability of prostate cancer cells induced by matriptase overexpression. In xenograft model, curcumin not only inhibits prostate cancer tumor growth and metastasis but also downregulates matriptase activity in vivo. Overall, the data indicate that curcumin exhibits a suppressive effect on prostate cancer cell invasion, tumor growth, and metastasis, at least in part via downregulating matriptase function. 
 Tumor detection by imaging proteolytic activity. The cell surface protease membrane-type serine protease-1 (MT-SP1), also known as matriptase, is often upregulated in epithelial cancers. We hypothesized that dysregulation of MT-SP1 with regard to its cognate inhibitor hepatocyte growth factor activator inhibitor-1 (HAI-1), a situation that increases proteolytic activity, might be exploited for imaging purposes to differentiate malignant from normal tissue. In this study, we show that MT-SP1 is active on cancer cells and that its activity may be targeted in vivo for tumor detection. A proteolytic activity assay with several MT-SP1-positive human cancer cell lines showed that MT-SP1 antibodies that inhibit recombinant enzyme activity in vitro also bind and inhibit the full-length enzyme expressed on cells. In contrast, in the same assay, MT-SP1-negative cancer cell lines were inactive. Fluorescence microscopy confirmed the cell surface localization of labeled antibodies bound to MT-SP1-positive cells. To evaluate in vivo targeting capability, 0.7 to 2 nmoles of fluorescently labeled antibodies were administered to mice bearing tumors that were positive or negative for MT-SP1. Antibodies localized to MT-SP1-positive tumors (n = 3), permitting visualization of MT-SP1 activity, whereas MT-SP1-negative tumors (n = 2) were not visualized. Our findings define MT-SP1 activity as a useful biomarker to visualize epithelial cancers using a non  invasive   antibody-based method. 
 Expression of matriptase correlates with tumour progression and clinical prognosis in oral squamous cell carcinoma. To investigate the relationship of matriptase expression in oral squamous cell carcinoma (OSCC) to clinicopathological characteristics, patient survival and cell-  invasive   properties.Matriptase expression in OSCC was evaluated by immunohistochemical staining, and its relationship to clinicopathological features and outcomes was assessed statistically. The shRNA-mediated stable knockdown of matriptase in OSCC cells was used to analyse cell proliferation, migration and invasion in vitro. Matriptase immunostaining score was correlated with histopathological grade, clinical stage, positive lymph node and distant metastasis, and higher matriptase immunostaining score was associated significantly with poor prognosis. Elevated matriptase expression in oral cancer cell lines was a significant promoter of oral cancer cell migration and invasion.Matriptase expression correlates with tumour progression and   invasive   capability in OSCC and may be an adverse prognostic marker for this cancer. 
 Matriptase and its putative role in cancer. Tumor progression and metastasis are the pathologic effects of uncontrolled or deregulated   invasive   growth, a process in which proteases play a fundamental role. They mediate the degradation of extracellular matrix components and intercellular cohesive structures to allow migration of the cells into the extracellular environment and activate growth and angiogenic factors. In addition to metalloproteases and the plasminogen activation system, another protease, matriptase, contributes substantially to these processes. Matriptase is a type II transmembrane trypsin-like serine protease that is expressed by cells of epithelial origin and is overexpressed in a variety of human cancers. It has been suggested that this protease not only facilitates cellular   invasive  ness but may also activate oncogenic pathways. This review summarizes current knowledge about matriptase, its putative role in tumor initiation and progression, and its potential as a novel target in anti-cancer therapy. 
  GeneRIFs  
 Matriptase expression correlates with tumour progression and   invasive   capability in oral squamous cell carcinoma 
 Emergent active matriptase is a functional biomarker of the transformed epithelium and that its proteolytic activity can be exploited to non  invasive  ly evaluate tumorigenesis in vivo. 
 MT-SP1 activity as a useful biomarker to visualize epithelial cancers using a non  invasive   antibody-based method. 
  [Mm]etastasis  PubMed  
 Evaluating the function of matriptase and N-acetylglucosaminyltransferase V in prostate cancer   metastasis  . Previous studies have identified a subclone cell line (PC-J) which was isolated from a metastatic human prostate cell line, PC-3. In vitro matrigel invasion assays and xenograft animal studies suggested that matriptase was a putative metastatic gene in human prostate carcinoma cells. Although low metastatic prostate tumor cells, LNCaP, also expressed high levels of matriptase mRNA, gelatin zymography indicated that LNCaP cells had extremely low matriptase activity. Further studies using RT-PCR and lectin blotting assays revealed that the expression of N-acetylglucosaminyltransferase V (MGAT5), a glycoprotein that stabilizes matriptase, was low in LNCaP cells compared to PC-3 and PC-J cells. The transient overexpression of MGAT5 significantly enhanced the activity of matriptase and the invasion ability in the LNCaP cells. Knock-down of MGAT5 in PC-3 cells attenuated the metastatic ability of the cells, as determined by the in vitro invasion assay and the xenograft animal studies. Matriptase and MGAT5 may play important role in the   metastasis   of prostate cancer. 
 Matriptase and HAI-1 are expressed by normal and malignant epithelial cells in vitro and in vivo. Matriptase and its cognate, Kunitz-type serine protease inhibitor, HAI-1, comprise a newly characterized extracellular matrix-degrading protease system that may function as an epithelial membrane activator for other proteases and latent growth factors. Both enzyme and inhibitor have been detected in breast cancer cells, immortalized mammary epithelial cells, and human milk, but not in cultured fibroblasts nor in fibrosarcoma cells. To test the hypothesis that this system is expressed by normal breast epithelium, invasive breast cancers, and other cancers of an epithelial origin (carcinomas) but not in cancers of a mesenchymal origin, we have expanded our expression analysis of matriptase and HAI-1 in vitro and in vivo. Matriptase and HAI-1 were detected at the protein and mRNA levels both in hormone-dependent and hormone-independent cultured breast cancer cells, and this expression correlated with the expression of the epithelial markers E-cadherin or ZO-1. However, none of the breast cancer cell lines tested that express the mesenchymal marker vimentin express matriptase or HAI-1, consistent with an epithelial-selective expression of this system. Expression of matriptase, as determined by Western blot analysis, was observed in primary human breast, gynecological, and colon carcinomas, but not in stromal-derived ovarian tumors and human sarcomas of various origins and histological grades. The epithelial-selective expression of matriptase and HAI-1 was further confirmed in human breast cancers by immunohistochemistry and in situ hybridization, where the expression of the protease and the inhibitor were found in the carcinoma cells and in surrounding normal breast epithelia. The expression of the matriptase/HAI-1 system by malignant epithelial cells in vivo suggests a possible role for this protease in multiple aspects of the pathophysiology of epithelial malignancy, including invasion and   metastasis  . 
 Expression of serine protease SNC19/matriptase and its inhibitor hepatocyte growth factor activator inhibitor type 1 in normal and malignant tissues of gastrointestinal tract. To provide the expression profile of serine protease SNC19/matriptase and its inhibitor hepatocyte growth factor activator inhibitor type 1 (HAI-1) in normal and malignant tissues of gastrointestinal tract at mRNA level for further study on their correlations with tumor progression and   metastasis  .Total RNAs were prepared from 37 samples of colorectal cancer tissues, 40 samples of gastric cancer tissues, and their adjacent normal tissues. The expression of SNC19/matriptase and HAI-1 in these samples was detected by real-time fluorescent quantitative PCR using glyceraldehyde-3-phosphate dehydrogenase as internal standard, and the clinical significance for the correlation with clinicopathological parameters was evaluated.In gastric cancer tissues the expression of HAI-1 and SNC19/matriptase was significantly lower than that in the corresponding adjacent normal tissues (Z = -3.280, P = 0.006; Z = -4.651, P = 0.000). HAI-1:SNC19/matriptase ratio showed no difference between normal and malignant tissues (P>0.05). Analysis of clinicopathological parameters showed decreased expression of HAI-1 and HAI-1:SNC19/matriptase ratio associated with stage III/IV gastric tumors as compared to stage I/II ones (Z = -2.140, P = 0.031; Z = -2.155, P = 0.031), and with lymph node-positive gastric cancer tissues as compared to lymph node-negative ones (Z = -2.081, P = 0.036; Z = -2.686, P = 0.006). The expression of SNC19/matriptase had no relationship with stages and lymph node   metastasis   (P>0.05). The expression of HAI-1 and HAI-1:SNC19/matriptase ratio increased in well-differentiated gastric cancer tissues, but there was no statistical significance (P>0.05). The difference of SNC19/matriptase expression was not significant in gastric cancer tissues of different histological differentiation status (P>0.05). In colorectal cancer tissues, the expression of HAI-1 and SNC19/matriptase was also markedly lower than that in their adjacent normal tissues (Z = -3.100, P = 0.002; Z = -2.731, P = 0.006), whereas HAI-1:SNC19/matriptase ratio showed no difference. Decreased expression of HAI-1 was associated with increased invasive depth and lymph node   metastasis  , but there was no statistical significance (P>0.05). The difference of SNC19/matriptase expression and HAI-1:SNC19/matriptase ratio was not significant in different stages and different lymph node   metastasis   status (P>0.05). The expression of SNC19/matriptase, HAI-1 or HAI-1:SNC19/martiptase ratio showed no difference in colorectal cancer tissues of different histological differentiation status (P>0.05).The expressions of SNC19/matriptase and its inhibitor HAI-1 are decreased in gastrointestinal cancer tissues compared to their normal counterparts, and the decreased expression of HAI-1 may correlate with invasion and lymph node   metastasis  . The possible mechanisms involved need to be further investigated. 
 Inhibition of tumor invasion by genomic down-regulation of matriptase through suppression of activation of receptor-bound pro-urokinase. Urokinase-type plasminogen activator (uPA) degrades the extracellular matrix and plays critical roles in tumor invasion and   metastasis  . Matriptase, a membrane-bound serine protease, was shown to activate uPA in a uPA receptor-free, solution-based study. We now investigate whether matriptase affects activation of receptor-bound uPA and contributes to the invasiveness of HRA human ovarian cancer cells in vitro and tumor behavior in nude mice. Here we show the following. 1) uPA expression was effectively stimulated by TGF-beta1 in HRA cells. 2) Antisense (AS)-matriptase transfection achieved a marked inhibition of receptor-bound pro-uPA activation without altering expression of uPA and uPA receptor mRNA and proteins, irrespective of whether cells were stimulated with TGF-beta1. 3) Tumor cell receptor-bound pro-uPA could be efficiently cleaved by matriptase to generate enzymatically active two-chain uPA. Thus, matriptase can substitute for plasmin in the proteolytic activation of pro-uPA to enzymatically active uPA. 4) The AS-matriptase-treated cells had a decreased ability to invade an extracellular matrix layer, as compared with control cells. 5) When the AS-matriptase-treated cells were injected intraperitoneally into nude mice, the mice developed smaller tumors. Our data identify a novel role for matriptase for activation of receptor-bound uPA. 
 Prometastatic effect of N-acetylglucosaminyltransferase V is due to modification and stabilization of active matriptase by adding beta 1-6 GlcNAc branching. Oligosaccharide moieties of glycoproteins are structurally altered during development, carcinogenesis, and malignant transformations. It is well known that beta1-6 GlcNAc branching, a product of UDP-GlcNAc alpha-mannoside beta1-6-N-acetylglucosaminyltransferase (GnT-V), is associated with malignant transformation as the results of such alterations. However, the mechanism by which beta1-6 GlcNAc branching is linked to   metastasis   remains unclear, because the identification of specific glycoprotein(s) that are glycosylated by GnT-V and its biological function have not been examined. We herein report that matriptase, which activates both urokinase-type plasminogen activator and hepatocyte growth factor, is a target protein for GnT-V. The overexpression of GnT-V in gastric cancer cells leads to severe peritoneal dissemination in athymic mice, which can be attributed to the increased expression of matriptase. This increase was due to the acquired resistance of matriptase to degradation, since it is glycosylated by GnT-V and a corresponding increase in the active form. These results indicate that this process is a key element in malignant transformation, as the direct result of oligosaccharide modification. 
 Down-regulation of matriptase by overexpression of bikunin attenuates cell invasion in prostate carcinoma cells. The precise mechanisms of   metastasis   in prostatic cancer are still unknown. A subculture cell line (PC-J) was isolated from the   metastasis   human prostate cell line PC-3. In vitro cell proliferation, wound healing and invasion assays revealed that tumorigenesis and   metastasis   differed between PC-3 and PC-J cells. Eight weeks after nude mice were prostate-injected with PC-J and PC-3 cells, the PC-3 group had low tumor volume and exhibited   metastasis   whereas the PC-J group had high tumor volume and no   metastasis  . Subsequent RT-PCR and immunoblot assays indicated that matriptase was the putative metastatic gene. Overexpression of bikunin significantly reduced the gene expression of matriptase, which attenuated in vitro cell invasion in the PC-3 cells. In vitro and xenograft animal models indicated different metastatic characteristics between PC-3 and PC-J cells, suggesting that matriptase plays an important role in the   metastasis   of prostate cancer. 
 The novel serine protease tumor-associated differentially expressed gene-15 (matriptase/MT-SP1) is highly overexpressed in cervical carcinoma. Tumor-associated differentially expressed gene-15 (TADG-15/matriptase/MT-SP1) is a novel transmembrane serine protease involved in numerous biologic processes, including activation of growth and angiogenic factors and degradation of extracellular matrix components. To assess the value of TADG-15 as a possible marker for tumor detection and/or as a target for therapeutic intervention, the authors investigated the frequency of expression of TADG-15 in human cervical tumors.TADG-15 expression was evaluated in 19 cervical carcinoma cell lines (i.e., 11 primary tumor cell lines and 8 established cell lines) and in 8 normal cervical keratinocyte control cultures using reverse transcriptase-polymerase chain reaction (RT-PCR). In addition, to validate gene expression data at the protein level, TADG-15 expression was evaluated by immunohistochemistry on paraffin embedded tissue from which all 11 primary tumor cell lines were established.TADG-15 was expressed at high levels in 8 of 11 (73%) primary cervical carcinoma cell lines and in 6 of 8 (75%) established cervical carcinoma cell lines by RT-PCR. Expression of TADG-15 was found in 6 of 6 (100%) primary squamous cell cervical carcinomas, whereas 2 of 5 (40%) primary adenocarcinomas expressed TADG-15. In contrast, none of the normal cervical keratinocyte control cultures (n = 4) or flash-frozen normal cervical biopsy specimens (n = 4) expressed TADG-15. Immunohistochemistry staining of paraffin embedded cervical carcinoma specimens confirmed TADG-15 expression in tumor cells and its absence on normal cervical epithelial cells.Cervical carcinoma cells expressed high levels of TADG-15, suggesting that this protease may play an important role in invasion and   metastasis  . Because TADG-15 appears only in abundance in squamous tumor tissue and contains a proteolytic cleavage site, suggesting that the TADG-15 protease domain is released, it may prove to be a useful diagnostic tool for the early detection of recurrent/persistent cervical carcinoma after standard treatment or as a novel molecular target for therapy in patients with cervical carcinoma. 
 Decreasing the ratio of matriptase/HAI‑1 by downregulation of matriptase as a potential adjuvant therapy in ovarian cancer. Tumor invasion an  d metastas  is are complex biological processes. Matriptase and its endogenous inhibitor, hepatocyte growth factor activator inhibitor‑1 (HAI‑1) are involved in invasio  n and meta  stasis. To evaluate the ratio of matriptase/HAI‑1 and their potential therapeutic value in ovarian cancer, HO‑8910 human ovarian cancer cells and the homologous high‑metastatic HO‑8910PM cells were used as in vitro cellular models ovarian cancer. The invasive and metastatic abilities, and the expression of matriptase and HAI‑1 in these cells were detected using scratch assays, Transwell chamber assays, reverse transcription‑quantitative polymerase chain reaction, western blotting and fluorescent immunocytochemistry. Following infection with lentivirus‑mediated matriptase‑targeting small interfering RNA (siRNA), cell cycle progression and apoptosis were also analyzed. The migration distance and number of invading HO‑8910PM cells were significantly increased compared with HO‑8910 cells. HO‑8910PM cells exhibited a significantly higher ratio of matriptase/HAI‑1 mRNA levels compared with HO‑8910 cells (0.51 vs. 0.24, ~2.2 fold increase). Compared with HO‑8910 cells, the matriptase mRNA level was increased by ~3.6 fold in HO‑8910PM cells, whereas the HAI‑1 mRNA level was increased by ~1.7 fold. Similar increases in protein expression levels were also observed in HO‑8910PM cells compared with HO‑8910 cells. Migration and invasiveness were positively correlated with matriptase expression level (r=0.994, P<0.01) and the ratio of matriptase/HAI‑1 (r=0.929, P<0.01). Downregulation of matriptase using siRNA resulted in inhibition of the invasive and metastatic abilities of HO‑8910PM cells, cell cycle arrest in the G0/G1 phase and increased apoptosis. Th  e present   study demonstrated that ovarian cancer cell metastasis and invasion were more dependent on upregulation of matriptase levels than downregulation of HAI‑1. Matriptase may be a potential adjuvant therap  eutic targ  et for inhibiting ovarian cancer invasion and metastasis. 
 Serum hepatocyte growth factor activator inhibitor type I (HAI-I) and type 2 (HAI-2) in prostate cancer. Hepatocyte growth factor activator inhibitor type 1 (HAI-1) and type 2 (HAI-2) are Kunitz-type serine protease inhibitors for hepatocyte growth factor activator (HGFA). We attempted to clarify whether serum levels of HAI-1 and HAI-2 could be a useful marker in patients with prostate cancer.Serum levels of HAI-1 and HAI-2 were measured by enzyme-linked immunosorbent assay in 27 patients with benign prostatic hyperplasia (BPH) and 118 patients with prostate cancer.The mean serum levels of HAI-1 in patients with prostate cancer were significantly higher than those in patients with BPH. Furthermore, the serum HAI-1 levels in patients with distant   metastasis   and hormone resistant prostate cancer were significantly elevated compared with those in patients with organ-confined diseases. There were no significant differences in serum HAI-2 levels among prostate cancer subgroups according to clinical stage. Significantly elevated levels of HAI-1 were detected in 38 patients with prostate cancer before any treatment.HAI-1 may be a potential tumor marker for prostate cancer. Further studies in large groups of patients are needed to define the clinical value of HAI-1. 
 Activation of hepatocyte growth factor and urokinase/plasminogen activator by matriptase, an epithelial membrane serine protease. Matriptase is an epithelial-derived, integral membrane serine protease. The enzyme was initially isolated from human breast cancer cells and has been implicated in breast cancer invasion and   metastasis  . In the current study, using active matriptase isolated from human milk, we demonstrate that matriptase is able to cleave various synthetic substrates with arginine or lysine as their P1 sites and prefers small side chain amino acids, such as Ala and Gly, at P2 sites. For the most reactive substrates, N-tert-butoxycarbonyl (N-t-Boc)-gamma-benzyl-Glu-Ala-Arg-7-amino-4-methylcoumarin (AMC) and N-t-Boc-Gln-Ala-Arg-AMC, the K(m) values were determined to be 3. 81 and 4.89 microm, respectively. We further demonstrated that matriptase can convert hepatocyte growth factor/scattering factor to its active form, which can induce scatter of Madin-Darby canine kidney epithelial cells and can activate c-Met tyrosine phosphorylation in A549 human lung carcinoma cells. In addition, we noted that matriptase can activate urokinase plasminogen activator but has no affect on plasminogen. These results suggest that matriptase could act as an epithelial, upstream membrane activator to recruit and activate stromal-derived downstream effectors important for extracellular matrix degradation and epithelial migration, two major events of tissue remodeling, cancer invasion, and   metastasis  . 
 Soluble epithin/PRSS14 secreted from cancer cells contains active angiogenic potential. Epithin (PRSS14/matriptase/ST14), a type II membrane protein, is involved in progression of epithelial cancers and   metastasis   as well as in the normal epidermal barrier function. When activated, it translocates into the cell-cell contacts and sheds into media. In order to understand the specific mechanism during tumor progression, we tested the angiogenic potential of secreted form of epithin. Epithin produced from the cancer cells shed more in hypoxia and induced motility of endothelial cells. Epithin enhanced the migration and invasion of mouse and bovine endothelial cells without cell proliferation. Furthermore, soluble epithin induced endothelial differentiation in the assay of the human endothelial microvessel-like tube formation and in that of the chicken chorioallantoic membrane. The knock-down of epithin in the 427 thymoma cell line abolished the protease activity of secreted epithin fraction, reduced the invasion of endothelial cells through matrigel, and tube formation activity. Only specific antibodies abolished the migration of endothelial cell and the vessel morphogenesis, suggesting that epithin specifically functions in these systems. Therefore, we propose that the secreted epithin in the hypoxic cancer microenvironment plays a role as a proangiogenic factor, and can be modulated with specific antibodies. 
 Curcumin-targeting pericellular serine protease matriptase role in suppression of prostate cancer cell invasion, tumor growth, and   metastasis  . Curcumin has been shown to possess potent chemopreventive and antitumor effects on prostate cancer. However, the molecular mechanism involved in curcumin's ability to suppress prostate cancer cell invasion, tumor growth, and   metastasis   is not yet well understood. In this study, we have shown that curcumin can suppress epidermal growth factor (EGF)- stimulated and heregulin-stimulated PC-3 cell invasion, as well as androgen-induced LNCaP cell invasion. Curcumin treatment significantly resulted in reduced matrix metalloproteinase 9 activity and downregulation of cellular matriptase, a membrane-anchored serine protease with oncogenic roles in tumor formation and invasion. Our data further show that curcumin is able to inhibit the induction effects of androgens and EGF on matriptase activation, as well as to reduce the activated levels of matriptase after its overexpression, thus suggesting that curcumin may interrupt diverse signal pathways to block the protease. Furthermore, the reduction of activated matriptase in cells by curcumin was also partly due to curcumin's effect on promoting the shedding of matriptase into an extracellular environment, but not via altering matriptase gene expression. In addition, curcumin significantly suppressed the invasive ability of prostate cancer cells induced by matriptase overexpression. In xenograft model, curcumin not only inhibits prostate cancer tumor growth and   metastasis   but also downregulates matriptase activity in vivo. Overall, the data indicate that curcumin exhibits a suppressive effect on prostate cancer cell invasion, tumor growth, and   metastasis  , at least in part via downregulating matriptase function. 
 Expression of matriptase correlates with tumour progression and clinical prognosis in oral squamous cell carcinoma. To investigate the relationship of matriptase expression in oral squamous cell carcinoma (OSCC) to clinicopathological characteristics, patient survival and cell-invasive properties.Matriptase expression in OSCC was evaluated by immunohistochemical staining, and its relationship to clinicopathological features and outcomes was assessed statistically. The shRNA-mediated stable knockdown of matriptase in OSCC cells was used to analyse cell proliferation, migration and invasion in vitro. Matriptase immunostaining score was correlated with histopathological grade, clinical stage, positive lymph node and distant   metastasis  , and higher matriptase immunostaining score was associated significantly with poor prognosis. Elevated matriptase expression in oral cancer cell lines was a significant promoter of oral cancer cell migration and invasion.Matriptase expression correlates with tumour progression and invasive capability in OSCC and may be an adverse prognostic marker for this cancer. 
 Molecular cloning of cDNA for matriptase, a matrix-degrading serine protease with trypsin-like activity. A major protease from human breast cancer cells was previously detected by gelatin zymography and proposed to play a role in breast cancer invasion and   metastasis  . To structurally characterize the enzyme, we isolated a cDNA encoding the protease. Analysis of the cDNA reveals three sequence motifs: a carboxyl-terminal region with similarity to the trypsin-like serine proteases, four tandem cysteine-rich repeats homologous to the low density lipoprotein receptor, and two copies of tandem repeats originally found in the complement subcomponents C1r and C1s. By comparison with other serine proteases, the active-site triad was identified as His-484, Asp-539, and Ser-633. The protease contains a characteristic Arg-Val-Val-Gly-Gly motif that may serve as a proteolytic activation site. The bottom of the substrate specificity pocket was identified to be Asp-627 by comparison with other trypsin-like serine proteases. In addition, this protease exhibits trypsin-like activity as defined by cleavage of synthetic substrates with Arg or Lys as the P1 site. Thus, the protease is a mosaic protein with broad spectrum cleavage activity and two potential regulatory modules. Given its ability to degrade extracellular matrix and its trypsin-like activity, the name matriptase is proposed for the protease. 
 Expression of matriptase and clinical outcome of human endometrial cancer. Matriptase, a type II transmembrane serine protease is involved in angiogenesis, degradation of extracellular matrix and in progression of some epithelial cancers. The purpose of the present study was to examine matriptase expression and evaluate its clinicopathological significance in endometrial cancer.Matriptase expression was examined in normal endometrium (n=20), endometrial hyperplasia (n=11) and endometrial cancer (n=65) samples. The distribution of cases that scored positive for each of the biological parameters examined was correlated with matriptase expression status obtained by immunohistochemistry.Matriptase was found to be significantly overexpressed in endometrial cancer as compared with normal endometrium and endometrial hyperplasia. Interestingly, matriptase expression is associated with stage (p=0.010), grade (p=0.021), depth of myometrial invasion (p=0.004), cervical involvement (p=0.021), lymph node   metastasis   (p=0.009), LVS involvement (p=0.041) and peritoneal cytology (p=0.045). The high matriptase expression was a significant predictor for poor prognosis when compared with low matriptase expression (Disease-free survival rate; p=0.032, Overall survival rate; p=0.011).High matriptase expression in endometrial cancer may be associated with poor prognosis. 
 Coordinate expression and functional profiling identify an extracellular proteolytic signaling pathway. A multidisciplinary method combining transcriptional data, specificity profiling, and biological characterization of an enzyme may be used to predict novel substrates. By integrating protease substrate profiling with microarray gene coexpression data from nearly 2,000 human normal and cancerous tissue samples, three fundamental components of a protease-activated signaling pathway were identified. We find that MT-SP1 mediates extracellular signaling by regulating the local activation of the prometastatic growth factor MSP-1. We demonstrate MT-SP1 expression in peritoneal macrophages, and biochemical methods confirm the ability of MT-SP1 to cleave and activate pro-MSP-1 in vitro and in a cellular context. MT-SP1 induced the ability of MSP-1 to inhibit nitric oxide production in bone marrow macrophages. Addition of HAI-1 or an MT-SP1-specific antibody inhibitor blocked the proteolytic activation of MSP-1 at the cell surface of peritoneal macrophages. Taken together, our work indicates that MT-SP1 is sufficient for MSP-1 activation and that MT-SP1, MSP-1, and the previously shown MSP-1 tyrosine kinase receptor RON are required for peritoneal macrophage activation. This work shows that this triad of growth factor, growth factor activator protease, and growth factor receptor is a protease-activated signaling pathway. Individually, MT-SP1 and RON overexpression have been implicated in cancer progression and   metastasis  . Transcriptional coexpression of these genes suggests that this signaling pathway may be involved in several human cancers. 
 The macrophage-stimulating protein pathway promotes   metastasis   in a mouse model for breast cancer and predicts poor prognosis in humans. A better understanding of tumor   metastasis   requires development of animal models that authentically reproduce the metastatic process. By modifying an existing mouse model of breast cancer, we discovered that macrophage-stimulating protein promoted breast tumor growth and   metastasis   to several organs. A special feature of our findings was the occurrence of osteolytic bone metastases, which are prominent in human breast cancer. To explore the clinical relevance of our model, we examined expression levels of three genes involved in activation of the MSP signaling pathway (MSP, MT-SP1, and MST1R) in human breast tumors. We found that overexpression of MSP, MT-SP1, and MST1R was a strong independent indicator of both   metastasis   and death in human breast cancer patients and significantly increased the accuracy of an existing gene expression signature for poor prognosis. These data suggest that signaling initiated by MSP is an important contributor to   metastasis   of breast cancer and introduce an independent biomarker for assessing the prognosis of humans with breast cancer. 
 Matriptase and its putative role in cancer. Tumor progression and   metastasis   are the pathologic effects of uncontrolled or deregulated invasive growth, a process in which proteases play a fundamental role. They mediate the degradation of extracellular matrix components and intercellular cohesive structures to allow migration of the cells into the extracellular environment and activate growth and angiogenic factors. In addition to metalloproteases and the plasminogen activation system, another protease, matriptase, contributes substantially to these processes. Matriptase is a type II transmembrane trypsin-like serine protease that is expressed by cells of epithelial origin and is overexpressed in a variety of human cancers. It has been suggested that this protease not only facilitates cellular invasiveness but may also activate oncogenic pathways. This review summarizes current knowledge about matriptase, its putative role in tumor initiation and progression, and its potential as a novel target in anti-cancer therapy. 
  GeneRIFs  
 The present study demonstrated that ovarian cancer cell   metastasis   and invasion were more dependent on upregulation of matriptase levels than downregulation of HAI1. Matriptase may be a potential adjuvant therapeutic target for inhibiting ovarian cancer invasion and   metastasis  . 
 Overexpression of bikunin reduced the gene expression of matriptase, which attenuated in vitro cell invasion. Different metastatic characteristics between PC-3 and PC-J cells suggest that matriptase plays a role in the   metastasis   of prostate cancer. 
 Cervical carcinoma cells expressed high levels of TADG-15, suggesting that this protease may play an important role in invasion and   metastasis  . 
 overexpression of MSP, MT-SP1, and MST1R was a strong independent indicator of both   metastasis   and death in human breast cancer 
  Summary  
 The protein encoded by this gene is an epithelial-derived, integral membrane serine protease. This protease forms a complex with the Kunitz-type serine protease inhibitor, HAI-1, and is found to be activated by sphingosine 1-phosphate. This protease has been shown to cleave and activate hepatocyte growth factor/scattering factor, and urokinase plasminogen activator, which suggest the function of this protease as an epithelial membrane activator for other proteases and latent growth factors. The expression of this protease has been associated with breast, colon, prostate, and ovarian tumors, which implicates its role in cancer invasion, and   metastasis  . [provided by RefSeq, Jul 2008] 
  [Cc]ell [Ii]nvasion  PubMed  
 Androgen receptor non-nuclear regulation of prostate cancer   cell invasion   mediated by Src and matriptase. Castration-resistant prostate cancers still depend on nuclear androgen receptor (AR) function despite their lack of dependence on exogenous androgen. Second generation anti-androgen therapies are more efficient at blocking nuclear AR; however resistant tumors still develop. Recent studies indicate Src is highly active in these resistant tumors. By manipulating AR activity in several different prostate cancer cell lines through RNAi, drug treatment, and the use of a nuclear-deficient AR mutant, we demonstrate that androgen acting on cytoplasmic AR rapidly stimulates Src tyrosine kinase via a non-genomic mechanism. Cytoplasmic AR, acting through Src enhances laminin integrin-dependent invasion. Active Matriptase, which cleaves laminin, is elevated within minutes after androgen stimulation, and is subsequently shed into the medium. Matriptase activation and shedding induced by cytoplasmic AR is dependent on Src. Concomitantly, CDCP1/gp140, a Matriptase and Src substrate that controls integrin-based migration, is activated. However, only inhibition of Matriptase, but not CDCP1, suppresses the AR/Src-dependent increase in invasion. Matriptase, present in conditioned medium from AR-stimulated cells, is sufficient to enhance invasion in the absence of androgen. Thus, invasion is stimulated by a rapid but sustained increase in Src activity, mediated non-genomically by cytoplasmic AR, leading to rapid activation and shedding of the laminin protease Matriptase. 
 ST14 (suppression of tumorigenicity 14) gene is a target for miR-27b, and the inhibitory effect of ST14 on cell growth is independent of miR-27b regulation. MicroRNAs are noncoding, endogenous small RNAs that regulate target genes by cleavage of the targeted mRNA or translational repression. We investigated the microRNAome using 2-color microarrays in a highly invasive human breast cancer cell line, MDA-MB-231 (subline 4175) and a noninvasive breast epithelial cell line, MCF10A. We found 13 microRNAs that were up-regulated, and nine were down-regulated significantly in 4175 cells (p < 0.05, -fold change >2) compared with MCF10A cells. Interestingly, miR-27b and its putative target gene, ST14 (suppressor of tumorigenicity 14), had inverse expression pattern in breast cancer cells. The 3'-untranslated region of ST14 contains a regulatory element for miR-27b, and our luciferase experiments indicate that antisense miR-27b enhances ST14 expression in cancer cells. Furthermore, antagomir of miR-27b suppressed   cell invasion   in 4175 cells, whereas pre-miR-27b stimulated invasion in moderately invasive ZR75 breast cancer cells. In addition, ST14 reduces cell proliferation as well as cell migration and invasion. Analysis of human breast tumors revealed that miR-27b expression increases during cancer progression, paralleling a decrease in ST14 expression. Furthermore, our data indicate that ST14 inhibits cells from entering into S phase by up-regulating p27, which results in down-regulation of cyclin E-CDK2 complexes, suggesting ST14 reduces cell growth through its effects on cell cycle-related proteins. Introduction of miR-27b into ST14-expressing cells did not suppress the effect on cell growth. These findings suggest that ST14 plays an important role in several biological processes, and some effects are not completely dependent on miR-27b regulation. 
 Down-regulation of matriptase by overexpression of bikunin attenuates   cell invasion   in prostate carcinoma cells. The precise mechanisms of metastasis in prostatic cancer are still unknown. A subculture cell line (PC-J) was isolated from the metastasis human prostate cell line PC-3. In vitro cell proliferation, wound healing and invasion assays revealed that tumorigenesis and metastasis differed between PC-3 and PC-J cells. Eight weeks after nude mice were prostate-injected with PC-J and PC-3 cells, the PC-3 group had low tumor volume and exhibited metastasis whereas the PC-J group had high tumor volume and no metastasis. Subsequent RT-PCR and immunoblot assays indicated that matriptase was the putative metastatic gene. Overexpression of bikunin significantly reduced the gene expression of matriptase, which attenuated in vitro   cell invasion   in the PC-3 cells. In vitro and xenograft animal models indicated different metastatic characteristics between PC-3 and PC-J cells, suggesting that matriptase plays an important role in the metastasis of prostate cancer. 
 Matriptase activation and shedding through PDGF-D-mediated extracellular acidosis. Activation of β-platelet-derived growth factor receptor (β-PDGFR) is associated with prostate cancer (PCa) progression and recurrence after prostatectomy. Analysis of the β-PDGFR ligands in PCa revealed association between PDGF-D expression and Gleason score as well as tumor stage. During the course of studying the functional consequences of PDGF ligand-specific β-PDGFR signaling in PCa, we discovered a novel function of PDGF-D for activation/shedding of the serine protease matriptase leading   to cell inva  sion, migration, and tumorigenesis. The present study showed that PDGF-D, not PDGF-B, induces extracellular acidification, which correlates with increased matriptase activation. A cDNA microarray analysis revealed that PDGF-D/β-PDGFR signaling upregulates expression of the acidosis regulator carbonic anhydrase IX (CAIX), a classic target of the transcriptional factor hypoxia-inducible factor-1α (HIF-1α). Cellular fractionation displayed a strong HIF-1α nuclear localization in PDGF-D-expressing cells. Treatment of vector control or PDGF-B-expressing cells with the HIF-1α activator CoCl2 led to increased CAIX expression accompanied by extracellular acidosis and matriptase activation. Furthermore, the analysis of the CAFTD cell lines, variants of the BPH-1 transformation model, showed that increased PDGF-D expression is associated with enhanced HIF-1α activity, CAIX induction, cellular acidosis, and matriptase shedding. Importantly, shRNA-mediated knockdown of CAIX expression effectively reversed extracellular acidosis and matriptase activation in PDGF-D-transfected BPH-1 cells and in CAFTD variants that express endogenous PDGF-D at a high level. Taken together, these novel findings reveal a new paradigm in matriptase activation involving PDGF-D-specific signal transduction leading to extracellular acidosis. 
 Effect of SNC19/ST14 gene overexpression on invasion of colorectal cancer cells. To study the effect of SNC19/ST14 gene overexpression on invasion in vitro of colorectal cancer cells.The adhesion of SNC19/ST14 gene-transfected cells to ECM was measured by MTT assay. The cell movement was evaluated by wound healing assay.   Cell invasion   and migration were determined by invasion assay in vitro.SNC19/ST14 gene overexpression could enhance invasion of colorectal cancer cells in vitro significantly and influence early cell adherence to ECM, but could not change cell movement significantly.SNC19/ST14 gene overexpression increases the local invasion of colorectal cancer cells in vitro. 
 Curcumin-targeting pericellular serine protease matriptase role in suppression of prostate cancer   cell invasion  , tumor growth, and metastasis. Curcumin has been shown to possess potent chemopreventive and antitumor effects on prostate cancer. However, the molecular mechanism involved in curcumin's ability to suppress prostate cancer   cell invasion  , tumor growth, and metastasis is not yet well understood. In this study, we have shown that curcumin can suppress epidermal growth factor (EGF)- stimulated and heregulin-stimulated PC-3   cell invasion  , as well as androgen-induced LNCaP   cell invasion  . Curcumin treatment significantly resulted in reduced matrix metalloproteinase 9 activity and downregulation of cellular matriptase, a membrane-anchored serine protease with oncogenic roles in tumor formation and invasion. Our data further show that curcumin is able to inhibit the induction effects of androgens and EGF on matriptase activation, as well as to reduce the activated levels of matriptase after its overexpression, thus suggesting that curcumin may interrupt diverse signal pathways to block the protease. Furthermore, the reduction of activated matriptase in cells by curcumin was also partly due to curcumin's effect on promoting the shedding of matriptase into an extracellular environment, but not via altering matriptase gene expression. In addition, curcumin significantly suppressed the invasive ability of prostate cancer cells induced by matriptase overexpression. In xenograft model, curcumin not only inhibits prostate cancer tumor growth and metastasis but also downregulates matriptase activity in vivo. Overall, the data indicate that curcumin exhibits a suppressive effect on prostate cancer   cell invasion  , tumor growth, and metastasis, at least in part via downregulating matriptase function. 
 Matriptase is involved in ErbB-2-induced prostate cancer   cell invasion  . Deregulation of both ErbB-2 signaling and matriptase activity has been associated with human prostate cancer (PCa) progression. In this communication, we investigated the roles of both ErbB-2 signaling in matriptase zymogen activation and matriptase in ErbB-2-induced PCa malignancy. In a human PCa cell progression model, we observed that advanced PCa C-81 LNCaP cells exhibited an aggressive phenotype with increased cell migration and invasion capacity; these cells concurrently showed both enhanced ErbB-2 phosphorylation and increased matriptase zymogen activation compared with parental C-33 LNCaP cells. Moreover, ErbB2 activation, both ligand-dependent (eg, epidermal growth factor treatment) and ligand-independent (eg, overexpression), was able to induce matriptase zymogen activation in this cell line. Inhibition of ErbB-2 activity by either the specific inhibitor, AG825, in epidermal growth factor-treated C-33 LNCaP cells or ErbB-2 knockdown in C-81 LNCaP cells, reduced matriptase activation. These observations were confirmed by similar studies using both DU145 and PC3 cells. Together, these data suggest that ErbB-2 signaling plays an important role in matriptase zymogen activation. ErbB-2-enhanced matriptase activation was suppressed by a phosphatidylinositol 3-kinase inhibitor (ie, LY294002) but not by a MEK inhibitor (ie, PD98059). Suppression of matriptase expression by small hairpin RNA knockdown in ErbB-2-overexpressing LNCaP cells dramatically suppressed cancer   cell invasion  . In summary, our data indicate that ErbB-2 signaling via the phosphatidylinositol 3-kinase pathway results in up-regulated matriptase zymogen activity, which contributes to PCa   cell invasion  . 
  GeneRIFs  
 prostate cancer   cell invasion   is stimulated by a rapid but sustained increase in Src activity, mediated non-genomically by cytoplasmic AR, leading to rapid activation and shedding of the laminin protease Matriptase 
 ErbB-2 signaling via the phosphatidylinositol 3-kinase pathway results in up-regulated matriptase zymogen activity, which contributes to prostate cancer   cell invasion  . 
 Overexpression of bikunin reduced the gene expression of matriptase, which attenuated in vitro   cell invasion  . Different metastatic characteristics between PC-3 and PC-J cells suggest that matriptase plays a role in the metastasis of prostate cancer. 
   
  
 SYK 
 
  NCBI Gene  
  GeneRIF  
  Pubmed  
 
 
  Search Term  Rank  Fields  
  [Ff]ilopodia  1  PubMed(1)  
  [Ii]nvadopodia  3  PubMed(1)  
  [Pp]odosome(s)?  5  PubMed(1)  
  [Mm]igration  6  PubMed(19); GeneRIFs(4)  
  [Ee]xtracellular [Mm]atrix  8  PubMed(6)  
  [Ii]nvasive  10  PubMed(18); GeneRIFs(1)  
  [Mm]etastasis  12  PubMed(18); GeneRIFs(8)  
  [Cc]ell [Ii]nvasion  13  PubMed(6); GeneRIFs(1)  
 
 
 hide/show details for SYK 
  [Ff]ilopodia  PubMed  
 Coordinate interactions of Csk, Src, and Syk kinases with [alpha]IIb[beta]3 initiate integrin signaling to the cytoskeleton. Integrins regulate cell adhesion and motility through tyrosine kinases, but initiation of this process is poorly understood. We find here that Src associates constitutively with integrin alphaIIbbeta3 in platelets. Platelet adhesion to fibrinogen caused a rapid increase in alphaIIbbeta3-associated Src activity, and active Src localized to   filopodia   and cell edges. Csk, which negatively regulates Src by phosphorylating Tyr-529, was also constitutively associated with alphaIIbbeta3. However, fibrinogen binding caused Csk to dissociate from alphaIIbbeta3, concomitant with dephosphorylation of Src Tyr-529 and phosphorylation of Src activation loop Tyr-418. In contrast to the behavior of Src and Csk, Syk was associated with alphaIIbbeta3 only after fibrinogen binding. Platelets multiply deficient in Src, Hck, Fgr, and Lyn, or normal platelets treated with Src kinase inhibitors failed to spread on fibrinogen. Inhibition of Src kinases blocked Syk activation and inhibited phosphorylation of Syk substrates (Vav1, Vav3, SLP-76) implicated in cytoskeletal regulation. Syk-deficient platelets exhibited Src activation upon adhesion to fibrinogen, but no spreading or phosphorylation of Vav1, Vav3, and SLP-76. These studies establish that platelet spreading on fibrinogen requires sequential activation of Src and Syk in proximity to alphaIIbbeta3, thus providing a paradigm for initiation of integrin signaling to the actin cytoskeleton. 
  [Ii]nvadopodia  PubMed  
 Tumor suppressor function of Syk in human MCF10A in vitro and normal mouse mammary epithelium in vivo. The normal function of Syk in epithelium of the developing or adult breast is not known, however, Syk suppresses tumor growth, invasion, and metastasis in breast cancer cells. Here, we demonstrate that in the mouse mammary gland, loss of one Syk allele profoundly increases proliferation and ductal branching and invasion of epithelial cells through the mammary fat pad during puberty. Mammary carcinomas develop by one year. Syk also suppresses proliferation and invasion in vitro. siRNA or shRNA knockdown of Syk in MCF10A breast epithelial cells dramatically increased proliferation, anchorage independent growth, cellular motility, and invasion, with formation of functional, extracellular matrix-degrading   invadopodia  . Morphological and gene microarray analysis following Syk knockdown revealed a loss of luminal and differentiated epithelial features with epithelial to mesenchymal transition and a gain in   invadopodia  l cell surface markers CD44, CD49F, and MMP14. These results support the role of Syk in limiting proliferation and invasion of epithelial cells during normal morphogenesis, and emphasize the critical role of Syk as a tumor suppressor for breast cancer. The question of breast cancer risk following systemic anti-Syk therapy is raised since only partial loss of Syk was sufficient to induce mammary carcinomas. 
  [Pp]odosome(s)?  PubMed  
 CrkL is an adapter for Wiskott-Aldrich syndrome protein and Syk. Wiskott-Aldrich syndrome (WAS) and X-linked thrombocytopenia are caused by mutations of the WAS protein (WASP) gene. WASP may be involved in the regulation of   podosome  , an actin-rich dynamic cell adhesion structure formed by various types of cells. The molecular links between WASP and   podosomes   or other cell adhesion structures are unknown. Platelets express an SH2-SH3 adapter molecule, CrkL, that can directly associate with paxillin, which is localized in   podosomes  . The hypothesis that CrkL binds to WASP was, therefore, tested. Results from coprecipitation experiments using anti-CrkL and GST-fusion proteins suggest that CrkL binds to WASP through its SH3 domain and that the binding was not affected by WASP tyrosine phosphorylation. The binding of GST-fusion SH3 domain of PSTPIP1 in vitro was also not affected by WASP tyrosine phosphorylation, suggesting that the binding of the SH3 domains to WASP is not inhibited by tyrosine phosphorylation of WASP. Anti-CrkL also coprecipitates a 72-kd protein, which was identified as syk tyrosine kinase, critical for collagen induced-platelet activation. CrkL immunoprecipitates contain kinase-active syk, as evidenced by an in vitro kinase assay. Coprecipitation experiments using GST-fusion CrkL proteins suggest that both SH2 and SH3 domains of CrkL are involved in the binding of CrkL to syk. WASP, CrkL, syk, and paxillin-like Hic-5 incorporated to platelet cytoskeleton after platelet aggregation. Thus, CrkL is a novel molecular adapter for WASP and syk and may potentially transfer these molecules to the cytoskeleton through association with cytoskeletal proteins such as Hic-5. 
  [Mm]igration  PubMed  
 Role of the docking protein Gab2 in beta(1)-integrin signaling pathway-mediated hematopoietic cell adhesion and   migration  . Gab2, a newly identified pleckstrin homology domain-containing docking protein, is a major binding protein of SHP-2 tyrosine phosphatase in interleukin (IL)-3-stimulated hematopoietic cells. Its signaling mechanism remains largely unknown. We report here an important regulatory role for Gab2 in beta(1) integrin signaling pathway that mediates hematopoietic cell adhesion and   migration  . Cross-linking of the beta(1) integrin on Ba/F3 cells induced rapid tyrosine phosphorylation of Gab2 and its association with Syk kinase, SHP-2 phosphatase, and the p85 subunit of phosphatidylinositol (PI)-3 kinase. In addition, Gab2 was also constitutively associated with SHP-1 phosphatase via its C-terminal Src homology 2 domain. Overexpression of the pleckstrin homology domain or a mutant Gab2 molecule lacking SHP-2 binding sites resulted in significant reductions in Ba/F3 cell adhesion and   migration  . Biochemical analyses revealed that enforced expression of Gab2 mutant molecules dramatically reduced beta(1)-integrin ligation-triggered PI3 kinase activation, whereas Erk kinase activation remained unaltered. Furthermore, transduction of primary hematopoietic progenitor cells from viable motheaten mice with these mutant Gab2 molecules also significantly ameliorated their enhanced   migration   capacity associated with the SHP1 gene mutation. Taken together, these results suggest an important signaling role for Gab2 in regulating hematopoietic cell adhesion and   migration  . 
 Angiotensin II-induced process of angiogenesis is mediated by spleen tyrosine kinase via VEGF receptor-1 phosphorylation. Spleen tyrosine kinase (Syk), expressed in endothelial cells, has been implicated in   migration   and proliferation and in vasculogenesis. This study was conducted to determine the contribution of Syk and the underlying mechanism to the angiogenic effect of ANG II and VEGF. Angiogenesis was determined by tube formation from the endothelial cell line EA.hy926 (EA) and human umbilical vein endothelial cells (HUVECs) and microvessel sprouting in rat aortic rings. ANG II (10 nM), EGF (30 ng/ml), and VEGF (50 ng/ml) stimulated EA cells and HUVECs to form tubular networks and increased aortic sprouting; these effects were blocked by VEGF receptor-1 and Flt-1 antibody (Flt-1/Fc) but not by the VEGF receptor-2 (Flk-1) antagonist SU-1498. ANG II increased the phosphorylation of Flt-1 but not Flk-1, whereas VEGF increased the phosphorylation of both receptors in EA cells and HUVECs. VEGF expression elicited by ANG II was not altered by Flt-1/Fc or SU-1498. EGF stimulated tube formation from EA cells and HUVECs and Flt-1 phosphorylation and aortic sprouting, which were blocked by the EGF receptor antagonist AG-1478 and Flt-1/Fc but not by SU-1498. ANG II-, EGF-, and VEGF-induced tube formation and aortic sprouting were attenuated by the Syk inhibitor piceatannol and by Syk short hairpin interfering (sh)RNA and small interfering RNA, respectively. ANG II, EGF, and VEGF increased Syk phosphorylation, which was inhibited by piceatannol and Syk shRNA in EA cells and HUVECs. Neither piceatannol nor Syk shRNA altered ANG II-, EGF-, or VEGF-induced phosphorylation of Flt-1. These data suggest that ANG II stimulates angiogenesis via transactivation of the EGF receptor, which promotes the phosphorylation of Flt-1 and activation of Syk independent of VEGF expression. 
 Angiotensin II-induced process of angiogenesis is mediated by spleen tyrosine kinase via VEGF receptor-1 phosphorylation. Spleen tyrosine kinase (Syk), expressed in endothelial cells, has been implicated in   migration   and proliferation and in vasculogenesis. This study was conducted to determine the contribution of Syk and the underlying mechanism to the angiogenic effect of ANG II and VEGF. Angiogenesis was determined by tube formation from the endothelial cell line EA.hy926 (EA) and human umbilical vein endothelial cells (HUVECs) and microvessel sprouting in rat aortic rings. ANG II (10 nM), EGF (30 ng/ml), and VEGF (50 ng/ml) stimulated EA cells and HUVECs to form tubular networks and increased aortic sprouting; these effects were blocked by VEGF receptor-1 and Flt-1 antibody (Flt-1/Fc) but not by the VEGF receptor-2 (Flk-1) antagonist SU-1498. ANG II increased the phosphorylation of Flt-1 but not Flk-1, whereas VEGF increased the phosphorylation of both receptors in EA cells and HUVECs. VEGF expression elicited by ANG II was not altered by Flt-1/Fc or SU-1498. EGF stimulated tube formation from EA cells and HUVECs and Flt-1 phosphorylation and aortic sprouting, which were blocked by the EGF receptor antagonist AG-1478 and Flt-1/Fc but not by SU-1498. ANG II-, EGF-, and VEGF-induced tube formation and aortic sprouting were attenuated by the Syk inhibitor piceatannol and by Syk short hairpin interfering (sh)RNA and small interfering RNA, respectively. ANG II, EGF, and VEGF increased Syk phosphorylation, which was inhibited by piceatannol and Syk shRNA in EA cells and HUVECs. Neither piceatannol nor Syk shRNA altered ANG II-, EGF-, or VEGF-induced phosphorylation of Flt-1. These data suggest that ANG II stimulates angiogenesis via transactivation of the EGF receptor, which promotes the phosphorylation of Flt-1 and activation of Syk independent of VEGF expression. 
 Beta-chemokine receptor CCR5 signals through SHP1, SHP2, and Syk. The beta-chemokine receptor CCR5 has been shown to modulate cell   migration  , proliferation, and immune functions and to serve as a co-receptor for the human immunodeficiency virus. We and others have shown that CCR5 activates related adhesion focal tyrosine kinase (RAFTK)/Pyk2/CAK-beta. In this study, we further characterize the signaling molecules activated by CCR5 upon binding to its cognate ligand, macrophage inflammatory protein-1beta (MIP1beta). We observed enhanced tyrosine phosphorylation of the phosphatases SHP1 and SHP2 upon MIP1beta stimulation of CCR5 L1.2 transfectants and T-cells derived from peripheral blood mononuclear cells. Furthermore, we observed that SHP1 associated with RAFTK. However, using a dominant-negative phosphatase-binding mutant of RAFTK (RAFTK(m906)), we found that RAFTK does not mediate SHP1 or SHP2 phosphorylation. SHP1 and SHP2 also associated with the adaptor protein Grb2 and the Src-related kinase Syk. Pretreatment of CCR5 L1.2 transfectants or T-cells with the phosphatase inhibitor orthovanadate markedly abolished MIP1beta-induced chemotaxis. Syk was also activated upon MIP1beta stimulation of CCR5 L1.2 transfectants or T-cells and associated with RAFTK. Overexpression of a dominant-negative Src-binding mutant of RAFTK (RAFTK(m402)) significantly attenuated Syk activation, whereas overexpression of wild-type RAFTK enhanced Syk activity, indicating that RAFTK acts upstream of CCR5-mediated Syk activation. Taken together, these results suggest that MIP1beta stimulation mediated by CCR5 induces the formation of a signaling complex consisting of RAFTK, Syk, SHP1, and Grb2. 
 The non-receptor tyrosine kinase Syk regulates lamellipodium formation and site-directed   migration   of human leukocytes. The tyrosine kinase Syk is associated with CD18, the beta-subunit of the leukocyte adhesion molecules of the beta(2) integrin family (CD11/CD18), and becomes activated upon beta(2) integrin-mediated adhesion. In this study, we elucidated the role of Syk in polarization and site-directed   migration   of neutrophil-like differentiated HL-60 cells and monocytic THP-1 cells. By means of confocal microscopy, we detected a homogenous distribution of Syk in unstimulated cells in suspension. The stimulation of HL-60 cells by formyl-methionyl-leucyl-phenylalanine (fMLP, 100 nM) or the activation of THP-1 cells by monocyte chemoattractant protein-1 (10 ng/ml) induced beta(2) integrin-mediated cell adhesion and polarization on immobilized fibrinogen which was associated with an enrichment of Syk at the lamellipodium forming site. This effect was abolished by function blocking anti-CD18 antibody or by treatment of the cells with the Syk inhibitor piceatannol (30 microM) suggesting that the redistribution of Syk required both, beta(2) integrin-mediated adhesion and Syk activation. Moreover, the inhibition of Syk by piceatannol or the downregulation of Syk by antisense technique resulted in an excessive formation of lamellipodia indicating that Syk may act as a negative regulator that limits lamellipodium formation. The analysis of chemotaxis revealed that the inhibition of Syk impaired the ability of the cells to follow a chemotactic gradient whereas random   migration   was intact. Taken together, our data suggest a novel role for Syk in the maintenance of a bipolar phenotype by regulating lamellipodium formation, which is a critical prerequisite for site-directed   migration   of leukocytes. 
 A role for Syk-kinase in the control of the binding cycle of the beta2 integrins (CD11/CD18) in human polymorphonuclear neutrophils. A fine control of beta(2) integrin (CD11/CD18)-mediated firm adhesion of human neutrophils to the endothelial cell monolayer is required to allow ordered e  migration  . To elucidate the molecular mechanisms that control this process, intracellular protein tyrosine signaling subsequent to beta(2) integrin-mediated ligand binding was studied by immunoprecipitation and Western blotting techniques. The 72-kDa Syk-kinase, which was tyrosine-phosphorylated upon adhesion, was found to coprecipitate with CD18, the beta-subunit of the beta(2) integrins. Moreover, inhibition of Syk-kinase by piceatannol enhanced adhesion and spreading but diminished N-formyl-Met-Leu-Phe-induced chemotactic   migration  . The enhancement of adhesiveness was associated with integrin clustering, which results in increased integrin avidity. In contrast, piceatannol had no effect on the surface expression or on the affinity of beta(2) integrins. Altogether, this suggests that Syk-kinase controls alternation of beta(2) integrin-mediated ligand binding with integrin detachment. 
 Epstein-Barr virus latent membrane protein-2A induces ITAM/Syk- and Akt-dependent epithelial   migration   through αv-integrin membrane translocation. Epstein-Barr virus (EBV) is a highly prevalent herpesvirus associated with epithelial cancers, including nasopharyngeal carcinoma (NPC). The EBV protein latent membrane protein 2 (LMP2) is expressed in NPC tumor tissue and has been shown to induce transformation, inhibit differentiation, and promote   migratio  n of epithelial cells. In this study, the effect of LMP2A on   migratio  n of human epithelial cells was further analyzed. LMP2A expression induced   migratio  n in human foreskin keratinocytes (HFK) and HaCaT keratinocytes measured by wound healing scratch assay and chemoattractant-induced Transwell   migratio  n assay. The induction of   migratio  n by LMP2A required the ITAM signaling domain of LMP2A and activation of the Syk tyrosine kinase. LMP2A-induced Transwell   migratio  n required the Akt signaling pathway, and activation of Akt by LMP2A required the ITAM signaling domain of LMP2A. LMP2A also induced phosphorylation of the Akt target GSK3β, a Wnt signaling mediator that has been shown to regulate the activity of focal adhesion kinase (FAK), a tyrosine kinase activated by clustering and ligand interaction of integrins. Inhibition of either FAK or its signaling mediator Src kinase inhibited LMP2A-induce  d migrati  on. Interestingly, αV-integrin was greatly increased in membrane-enriched fractions by LMP2A, and a neutralizing antibody to αV-integrin bloc  ked migra  tion, suggesting that the effects of LMP2A on membrane-localized αV-integrin prom  oted migr  ation. The results of this study indicate that LMP2A expression in human epithelial cells induces αV-integrin-depe  ndent mig  ration through a mechanism requiring ITAM-mediated Syk and Akt activation and inducing membrane translocation or stabilization of αV-integrin and FAK activation. The specific effects of LMP2A on an integrin with a diverse repertoire of ligand specificities could p  romote mi  gration of different cell types and be initiated by multiple chemoattractants. 
 Involvement of the Syk-mTOR pathway in follicular lymphoma cell invasion and angiogenesis. Follicular lymphoma (FL) is the second-most common non-Hodgkin's lymphoma. The disease affects the lymph nodes, and 50% of patients present with bone marrow infiltration; however, the mechanisms involved in dissemination of the disease are not yet known. We previously reported that FL cells display an overexpression of Syk, a tyrosine kinase involved in many cellular processes including cell   migration  . Therefore, we sought to explore its role in the invasive process. Here, we show that FL patients display higher matrix metalloproteinase (MMP)-9 and vascular endothelial growth factor (VEGF) levels than healthy donors. Moreover, using Syk small interfering RNA and the Syk inhibitor R406, we demonstrate that, in FL cells, Syk is involved in the regulation of MMP-9 and VEGF expression, and that invasion and angiogenesis is mediated through a phosphatidylinositol-3 kinase (PI3K)-mammalian target of rapamycin module. Finally, using a FL xenograft mouse model we observe that fostamatinib (R788), inhibits MMP-9 expression and angiogenesis in vivo. Altogether, this study provides strong evidence that Syk represents an encouraging therapeutic target in FL and suggests the potential use of fostamatinib as an anti-invasive and anti-angiogenic drug. 
 Full-length spleen tyrosine kinase inhibits the invasion and metastasis of human laryngeal squamous cell carcinoma. This study aimed to investigate correlation between full-length spleen tyrosine kinase [SYK (L)] expression and clinical characteristics of laryngeal squamous cell carcinoma (LSCC), and explore effects of SYK (L) on invasion and metastasis of LSCC.The human laryngeal cancer Hep-2 cells with low SYK (L) expression were transfected with pIRES2-EGFP-SYK (L) vector and empty vector pIRES2-EGFP to generate Hep-2-SYK (L) cells and Hep-2-neo cells. The cell invasion and   migration   abilities were determined.The SYK (L) positive expression rate in LSCC tissues was significantly lower than in vocal cord dysplasia tissues and normal laryngeal tissues (P < 0.05). There was a significant correlation between SYK (L) expression and LSCC T stage, histopathological grade and lymph node metastasis (P < 0.05). mRNA expression of SYK (L) in Hep-2-SYK (L) cells was significantly higher than in Hep-2-neo cells and Hep-2 cells (P < 0.01). The protein expression of SYK (L) in Hep-2-SYK (L) cells was markedly higher than in Hep-2-neo cells and Hep-2 cells (P < 0.01). The number of invasive cells was significantly lower in Hep-2-SYK (L) group than in Hep-2-neo group and Hep-2 group (P < 0.01). The average number of migrating cells in Hep-2-SYK (L) group also markedly reduced as compared to Hep-2-neo group and Hep-2 group (P < 0.01).The SYK (L) expression was down-regulated in LSCC, which was closely correlated with cancer growth and lymph node metastasis. SYK (L) up-regulation was able to inhibit the invasion and metastasis of LSCC, therefore suppressing tumor development. Thus, SYK (L) may be a potential target for the LSCC treatment. 
 A role for prohibitin in mast cell activation: location matters. Prohibitin 1 (PHB1) and PHB2 are evolutionarily conserved, ubiquitously expressed, pleotropic proteins that control various fundamental cellular processes, including proliferation,   migration  , metabolism, and death. Studies have unveiled a crucial role for plasma membrane-associated PHBs in regulating tumor metastasis, viral entry, and immune cell activation. A study now identifies a role for PHB1 in the activation of mast cells and allergic reactions mediated by immunoglobulin E (IgE). PHB1 was primarily localized in mast cell granules; however, in response to stimulation with antigen, PHB1 translocated to plasma membrane lipid rafts to form a ternary complex with the high-affinity IgE receptor FcεRIγ and the nonreceptor tyrosine kinase Syk. Syk became activated, which led to the activation of downstream signaling that stimulated mast cell degranulation and the secretion of cytokines. PHB1 was phosphorylated by the Src family tyrosine kinase Lyn, and palmitoylation of PHB1 was required for its association with the plasma membrane. These observations unveil a previously uncharacterized facet of prohibitin biology and shed further light on the proximal events that drive the activation of FcεRI by IgE in mast cells during allergic reactions. 
 The Epstein-Barr virus causes epithelial-mesenchymal transition in human corneal epithelial cells via Syk/src and Akt/Erk signaling pathways. Although Epstein-Barr virus (EBV)-associated keratitis is rare, it can cause acute corneal necrosis and neovascularization. We aimed to examine the signaling mechanism by which EBV causes epithelial-mesenchymal transition (EMT) in human corneal epithelial cells (HCECs) in vitro.The cellular response to EBV was assessed by real-time PCR, Western blot,   migration   assay, invasion assay, inhibitor assay, and ELISA assay.A model of EBV-induced EMT was established in HCECs. The EBV induced morphologic changes in the cells; the loss of epithelial markers E-cadherin, ZO-1, and β-catenin; and an increase in the mesenchymal markers N-cadherin, Vimentin, Snail, and TCF8/Zeb1. The EBV infection also led to the nuclear translocation of Snail and TCF8/Zeb1; enhanced the secretion of IL-6, IL-8, VEGF, TGF-β1, TNF-α, and MCP-1; and upregulated the expression of MMP2 and MMP9. The EBV-infected HCECs exhibited increas  ed migrat  ion and invasiveness compared to uninfected HCECs. We measured the involvement of Syk, Src, PI3K/Akt, and Erk signaling, but not Smad, in EMT by EBV-induced TGF-β1. We demonstrated that treatment with TGF-β1, TGF-β receptors, Syk, or Src inhibitor blocked TGF-β1, Syk, or Src signaling activation, and EMT development by EBV. Moreover, these inhibitors prevented PI3K/Akt and Erk activation.An EBV infection in HCECs can lead to a mesenchymal fibroblast-like morphology, and cause EMT through the activation of PI3K/Akt and Erk by TGF-β1-mediated Syk and Src signaling. This phenomenon may have implications for EBV-associated keratitis and molecular approaches to treatment. 
 Protein-tyrosine kinase, Syk, is required for CXCL12-induced polarization of B cells. Cell polarization and   migration   in response to CXCL12 is essential for hematopoiesis. To investigate the role of Syk in CXCL12/CXCR4-induced signaling, wild-type Syk or its dominant-negative form (DN-Syk) was introduced in mouse pro-B cells, BAF3. With CXCL12 stimulation, BAF3 cells became polarized with the formation of a leading edge and contractile uropod at the rear end with increased motility. Overexpression of wild-type Syk caused enhanced polarization, whereas DN-Syk inhibited cell polarity due to the loss of contractile structure at the rear end, and the altered phenotype was enhanced after CXCL12 stimulation. Motility of mutant BAF3 containing DN-Syk increased independent of CXCL12 stimulation. As beta1 integrin-mediated cell adhesion was inhibited, decreased adhesion might promote motility. CXCL12 stimulation led to prompt activation of RhoA, but expression of DN-Syk suppressed RhoA activation. These results demonstrate that Syk participates in CXCL12-induced cell polarization, which occurs in concert with cell adhesion mediated by beta1 integrin. 
 Inhibition of beta 2 integrin receptor and Syk kinase signaling in monocytes by the Src family kinase Fgr. While beta 2 integrin ligand-receptor recognition interactions are well characterized, less is known about how these events trigger signal transduction cascades to regulate the transition from tethering to firm adhesion, spreading, and transendothelial   migration  . We have identified critical positive and negative regulatory components of this cascade in monocytes. Whereas the Syk tyrosine kinase is essential for beta 2 integrin signaling and cell spreading, the Src family kinase Fgr is a negative regulator of this pathway. Fgr selectively inhibits beta 2 but not beta 1 integrin signaling and Syk kinase function via a direct association between the Fgr SH2 domain and Syk tyrosine Y342. The inhibitory effects of Fgr are independent of its kinase activity, are dose dependent, and can be overcome by chemokines and inflammatory mediators. 
 Syk, a protein-tyrosine kinase, suppresses the cell motility and nuclear factor kappa B-mediated secretion of urokinase type plasminogen activator by inhibiting the phosphatidylinositol 3'-kinase activity in breast cancer cells. Tumor growth and metastasis are multifaceted processes that mainly involve cell adhesion, proteolytic degradation of the extracellular matrix, and cell   migration  . Syk is a member of a tyrosine kinase family that is expressed mostly in hematopoietic cells. Syk is expressed in cell lines of epithelial origin, but its function in these cells remains unknown. Here we report that Syk is expressed in MCF-7 cells but not in MDA-MB-231 cells. The overexpression of wild type Syk kinase but not kinase-negative Syk suppressed cell motility and inhibited the activation of phosphatidylinositol (PI) 3'-kinase in MDA-MB-231 cells. In contrast, when Syk-specific antisense S-oligonucleotide but not the sense S-oligonucleotide was transfected to MCF-7 cells the level of PI 3'-kinase activity as well as cell motility were increased. The MDA-MB-231 cells transfected with wild type Syk cDNA followed by treatment with piceatannol, a Syk inhibitor, enhanced cell motility and PI 3'-kinase activity. Pervanadate, a phosphotyrosine phosphatase inhibitor, induced PI 3'-kinase activity and stimulated the interaction between the inhibitor of nuclear factor kappa B alpha (I kappa B alpha) and the p85 alpha domain of PI 3'-kinase through tyrosine phosphorylation of the I kappa B alpha, which ultimately resulted in nuclear factor kappa B (NF kappa B) activation. Pervanadate had no effect on the activation of Syk in these cells. However, Syk suppressed the NF kappa B transcriptional activation and interaction between I kappa B alpha and PI 3'-kinase by inhibiting the tyrosine phosphorylation of I kappa B alpha. Syk, PI 3'-kinase inhibitors, and NF kappa B inhibitory peptide inhibited urokinase type plasminogen activator (uPA) secretion and cell motility in these cells. To our knowledge, this is the first report that Syk suppresses the cell motility and inhibits the PI 3'-kinase activity and uPA secretion by blocking NF kappa B activity through tyrosine phosphorylation of I kappa B alpha. These data further demonstrate a functional molecular link between Syk-regulated PI 3'-kinase activity and NF kappa B-mediated uPA secretion, and all of these ultimately control the motility of breast cancer cells. 
 Spleen tyrosine kinase mediates BEAS-2B cell   migration   and proliferation and human rhinovirus-induced expression of vascular endothelial growth factor and interleukin-8. Spleen tyrosine kinase (Syk) is an immunoregulatory tyrosine kinase that was identified originally in leukocytes. It is a key regulator of innate immunity as well as hematopoietic cell differentiation and proliferation. A role for Syk in regulating normal cellular functions in nonhematopoietic cells is increasingly recognized. We have shown previously robust Syk expression in airway epithelium, where it regulates the early inflammatory response to human rhinovirus (HRV) infections, and HRV cell entry by clathrin-mediated endocytosis. To test the hypothesis that Syk plays a role in modulating airway epithelial cell proliferation,   migration  , and production of vascular endothelial growth factor and interleukin-8, we studied the BEAS-2B human bronchial epithelial cell line and primary human airway epithelia from normal and asthmatic donors using Syk-specific pharmacologic inhibitors and small interfering RNA. Using an in vitro "wounding" model, we demonstrated significant impairment of "wound" closure after treatment with the Syk inhibitors N4-(2,2-dimethyl-3-oxo-4H-pyrid[1,4]oxazin-6-yl)-5-fluoro-N2-(3,4,5-trimethoxyphenyl)-2,4-pyrimidinediamine (R406) and 2-[7-(3,4-dimethoxyphenyl)-imidazo[1,2-c]pyrimidin-5-ylamino]-nicotinamide dihydrochloride (BAY61-3606), overexpression of the kinase-inactive Syk(K396R) mutant, and Syk knockdown by small interfering RNA. HRV infection also impaired wound healing, an effect that was partly Syk-dependent because wound healing was impaired further when HRV infection occurred in the presence of Syk inhibition. Further investigation of potential regulatory mechanisms revealed that inhibition of Syk suppressed HRV-induced vascular endothelial growth factor expression while promoting the activation of caspase-3, a mediator of epithelial cell apoptosis. Together, these results indicate that Syk plays a role in promoting epithelial cell proliferation and   migration  , while mitigating the effects of apoptosis. 
 SYK interaction with ITGβ4 suppressed by Epstein-Barr virus LMP2A modulates   migratio  n and invasion of nasopharyngeal carcinoma cells. Epstein-Barr virus (EBV)-encoded Latent Membrane Protein 2A (LMP2A) is an EBV latency-associated protein regularly expressed in nasopharyngeal carcinoma (NPC). In B cells, LMP2A activity resembles that of a constitutively activated antigen receptor, which recruits the Syk tyrosine kinase to activate a set of downstream signaling pathways. LMP2A also downregulates cellular Syk levels. In the present study, we demonstrate that Syk interacts with the integrin β4 subunit (ITGβ4) of integrin α6β4 in epithelial cells and that concurrent LMP2A expression interferes with this interaction by competitive binding to Syk. We find that both Syk and LMP2A have an effect on ITGβ4 cell surface expression. However, in LMP2A expressing cells, ITGβ4 remains concentrated at the cellular protrusions, an expression pattern characteristic of motile cells, including NPC-derived epithelial cells. This effect of LMP2A on ITGβ4 localization is associated with a greater propens  ity for m  igration and invasion in-vitro, and may contribute to the invasive property of LMP2A-expressing NPC. 
 Neutrophil activation via beta2 integrins (CD11/CD18): molecular mechanisms and clinical implications. Polymorphonuclear neutrophils (PMN) are key components of the innate immunity and their efficient recruitment to the sites of lesion is a prerequisite for acute inflammation. Signaling via adhesion molecules of the beta2 integrin family (CD11/CD18) plays an essential role for PMN recruitment and activation during inflammation. In this review, we will focus on the non-receptor tyrosine kinase Syk, an important downstream signaling component of beta2 integrins that is required for the control of different PMN functions including adhesion,   migration   and phagocytosis. The exploration of beta2 integrin-mediated Syk activation provided not only novel insights into the control of PMN functions but also led to the identification of Syk as a new molecular target for therapeutic intervention during inflammatory diseases. 
 Thrombin-induced tyrosine phosphorylation of HS1 in human platelets is sequentially catalyzed by Syk and Lyn tyrosine kinases and associated with the cellular   migration   of the protein. Thrombin stimulation of platelets triggers Tyr phosphorylation of several signaling proteins, most of which remain unidentified. In this study, we demonstrate for the first time that hematopoietic lineage cell-specific protein 1 (HS1) undergoes a transient Tyr phosphorylation in human platelets stimulated with thrombin. The protein is synergistically phosphorylated by Syk and Lyn tyrosine kinases according to a sequential phosphorylation mechanism. By means of specific inhibitors (PP2, SU6656, and piceatannol) and phosphopeptide-specific antibodies, as well as by coimmunoprecipitation and binding competition experiments, we show that Syk acts as the primary kinase that phosphorylates HS1 at Tyr397 and that Syk phosphorylation is required for HS1 interaction with the Lyn SH2 domain. Upon docking to Syk-phosphorylated HS1, Lyn catalyzes the secondary phosphorylation of the protein at Tyr222. Once the secondary Tyr phosphorylation of HS1 is accomplished the protein dissociates from Lyn and undergoes a dephosphorylation process. HS1 Tyr phosphorylation does not occur when thrombin-induced actin assembly is inhibited by cytochalasin D even under conditions in which Syk and Lyn are still active. Immunofluorescence microscopic analysis shows that the agonist promotes HS1   migration   to the plasma membrane and that the inhibition of Lyn-mediated secondary phosphorylation of HS1 abrogates the subcellular translocation of the protein. All together these results indicate that HS1 Tyr phosphorylation catalyzed by Syk and Lyn plays a crucial role in the translocation of the protein to the membrane and is involved in the cytoskeleton rearrangement triggered by thrombin in human platelets. 
 Priming of the vascular endothelial growth factor signaling pathway by thrombospondin-1, CD36, and spleen tyrosine kinase. CD36 plays a critical role in the inhibition of angiogenesis through binding to the type 1 repeats of thrombospondin-1 (TSP-1) and activating Fyn tyrosine kinase and MAPK pathways. Here, we reveal a novel association of CD36 with VEGFR-2 and spleen tyrosine kinase (Syk). We also address the correlation between the expression of CD36 and Syk by demonstrating that overexpression of CD36 in HUVECs up-regulates endogenous Syk expression. We also define a new role for TSP-1 and CD36 in the activation of the VEGFR-2 signaling pathway that requires Syk. Our findings also identify a role for Syk as a stimulator of VEGF-A-induced angiogenesis by increasing phosphorylation of Y1175 in VEGFR-2, which is a major tyrosine for promoting VEGF-A-induced endothelial cell   migration  . Together, these studies introduce a new signaling pathway for TSP-1, CD36, and Syk, and address the role of these proteins in regulating the angiogenic switch. 
  GeneRIFs  
 Epstein-Barr virus latent membrane protein-2A induces ITAM/Syk- and Akt-dependent epithelial   migration   through alphav-integrin membrane translocation 
 Syk plays a role in promoting epithelial cell proliferation and   migration  , while mitigating the effects of apoptosis. 
 review focuses on the non-receptor tyrosine kinase Syk as an important downstream signaling component of beta2 integrins (CD11/CD18) that is required for the control of different PMN functions including adhesion,   migration   and phagocytosis. 
 Novel role for Syk in the maintenance of a bipolar phenotype by regulating lamellipodium formation, which is a critical prerequisite for site-directed   migration   of leukocytes. 
  [Ee]xtracellular [Mm]atrix  PubMed  
 Src-family and Syk kinases in activating and inhibitory pathways in innate immune cells: signaling cross talk. The response of innate immune cells to growth factors, immune complexes,   extracellular matrix   proteins, cytokines, pathogens, cellular damage, and many other stimuli is regulated by a complex net of intracellular signal transduction pathways. The majority of these pathways are either initiated or modulated by Src-family or Syk tyrosine kinases present in innate cells. The Src-family kinases modulate the broadest range of signaling responses, including regulating immunoreceptors, C-type lectins, integrins, G-protein-coupled receptors, and many others. Src-family kinases also modulate the activity of other kinases, including the Tec-family members as well as FAK and Pyk2. Syk kinase is required for initiation of signaling involving receptors that utilize immunoreceptor tyrosine activation (ITAM) domains. This article reviews the major activating and inhibitory signaling pathways regulated by these cytoplasmic tyrosine kinases, illuminating the many examples of signaling cross talk between pathways. 
 Tumor suppressor function of Syk in human MCF10A in vitro and normal mouse mammary epithelium in vivo. The normal function of Syk in epithelium of the developing or adult breast is not known, however, Syk suppresses tumor growth, invasion, and metastasis in breast cancer cells. Here, we demonstrate that in the mouse mammary gland, loss of one Syk allele profoundly increases proliferation and ductal branching and invasion of epithelial cells through the mammary fat pad during puberty. Mammary carcinomas develop by one year. Syk also suppresses proliferation and invasion in vitro. siRNA or shRNA knockdown of Syk in MCF10A breast epithelial cells dramatically increased proliferation, anchorage independent growth, cellular motility, and invasion, with formation of functional,   extracellular matrix  -degrading invadopodia. Morphological and gene microarray analysis following Syk knockdown revealed a loss of luminal and differentiated epithelial features with epithelial to mesenchymal transition and a gain in invadopodial cell surface markers CD44, CD49F, and MMP14. These results support the role of Syk in limiting proliferation and invasion of epithelial cells during normal morphogenesis, and emphasize the critical role of Syk as a tumor suppressor for breast cancer. The question of breast cancer risk following systemic anti-Syk therapy is raised since only partial loss of Syk was sufficient to induce mammary carcinomas. 
 Spleen tyrosine kinase is important in the production of proinflammatory cytokines and cell proliferation in human mesangial cells following stimulation with IgA1 isolated from IgA nephropathy patients. IgA immune complexes are capable of inducing human mesangial cell (HMC) activation, resulting in release of proinflammatory and profibrogenic mediators. The subsequent inflammation, cellular proliferation, and synthesis of   extracellular matrix   lead to the progression of IgA nephropathy (IgAN). Spleen tyrosine kinase (SYK) is an intracellular protein tyrosine kinase involved in cell signaling downstream of immunoreceptors. In this study, we determined whether SYK is involved in the downstream signaling of IgA1 stimulation in HMC, leading to production of proinflammatory cytokines/chemokines and cell proliferation. Incubation of HMC with IgA1 purified from IgAN patients significantly increased the synthesis of MCP-1 in a dose-dependent manner. There was also significantly increased production of IL-6, IL-8, IFN-γ-inducible protein-10, RANTES, and platelet-derived growth factor-BB. Stimulation of HMC with heat-aggregated IgA1 purified from IgAN patients induced significantly increased HMC proliferation. Both pharmacological inhibition of SYK and knockdown of SYK by small interfering RNA significantly reduced the synthesis of these mediators and inhibited HMC proliferation. Moreover, positive immunostaining for total and phospho-SYK in glomeruli of kidney biopsies from IgAN patients strongly suggests the involvement of SYK in the pathogenesis of IgAN. To our knowledge, we demonstrate, for the first time, the involvement of SYK in the downstream signaling of IgA1 stimulation in HMC and in the pathogenesis of IgAN. Hence, SYK represents a potential therapeutic target for IgAN. 
 Syk, a protein-tyrosine kinase, suppresses the cell motility and nuclear factor kappa B-mediated secretion of urokinase type plasminogen activator by inhibiting the phosphatidylinositol 3'-kinase activity in breast cancer cells. Tumor growth and metastasis are multifaceted processes that mainly involve cell adhesion, proteolytic degradation of the   extracellular matrix  , and cell migration. Syk is a member of a tyrosine kinase family that is expressed mostly in hematopoietic cells. Syk is expressed in cell lines of epithelial origin, but its function in these cells remains unknown. Here we report that Syk is expressed in MCF-7 cells but not in MDA-MB-231 cells. The overexpression of wild type Syk kinase but not kinase-negative Syk suppressed cell motility and inhibited the activation of phosphatidylinositol (PI) 3'-kinase in MDA-MB-231 cells. In contrast, when Syk-specific antisense S-oligonucleotide but not the sense S-oligonucleotide was transfected to MCF-7 cells the level of PI 3'-kinase activity as well as cell motility were increased. The MDA-MB-231 cells transfected with wild type Syk cDNA followed by treatment with piceatannol, a Syk inhibitor, enhanced cell motility and PI 3'-kinase activity. Pervanadate, a phosphotyrosine phosphatase inhibitor, induced PI 3'-kinase activity and stimulated the interaction between the inhibitor of nuclear factor kappa B alpha (I kappa B alpha) and the p85 alpha domain of PI 3'-kinase through tyrosine phosphorylation of the I kappa B alpha, which ultimately resulted in nuclear factor kappa B (NF kappa B) activation. Pervanadate had no effect on the activation of Syk in these cells. However, Syk suppressed the NF kappa B transcriptional activation and interaction between I kappa B alpha and PI 3'-kinase by inhibiting the tyrosine phosphorylation of I kappa B alpha. Syk, PI 3'-kinase inhibitors, and NF kappa B inhibitory peptide inhibited urokinase type plasminogen activator (uPA) secretion and cell motility in these cells. To our knowledge, this is the first report that Syk suppresses the cell motility and inhibits the PI 3'-kinase activity and uPA secretion by blocking NF kappa B activity through tyrosine phosphorylation of I kappa B alpha. These data further demonstrate a functional molecular link between Syk-regulated PI 3'-kinase activity and NF kappa B-mediated uPA secretion, and all of these ultimately control the motility of breast cancer cells. 
 Syk activation is required for spreading and H2O2 release in adherent human neutrophils. Chemoattractant-stimulated polymorphonuclear leukocytes (PMNs) that are adherent to   extracellular matrix   proteins exhibit a massive, sustained respiratory burst that requires cell spreading. However, the signaling pathways culminating in PMN spreading are not well characterized. Studies showing that protein tyrosine phosphorylation increases with PMN spreading suggest that phosphorylation is critical for this process. In the present study, we observed increased tyrosine phosphorylation of both focal adhesion kinase and Syk in FMLP-activated PMNs that had been plated onto fibrinogen; an increase in Syk activity, but not focal adhesion kinase activity, was apparent. The time course of Syk phosphorylation correlated with the initiation of cell spreading and H2O2 release. Pretreatment of PMNs with piceatannol, a Syk-selective inhibitor, blocked Syk activity, cell spreading, and H2O2 release, indicating that Syk activity was required for the activation of adherent PMNs. Paxillin is a cytoskeletally associated protein that is also tyrosine phosphorylated during PMN spreading and H2O2 release. Paxillin phosphorylation is kinetically slower than Syk phosphorylation and is inhibited with piceatannol, suggesting that paxillin is a substrate for Syk. An analysis of Syk immunoprecipitates indicated that Syk and paxillin associate during PMN spreading. This interaction is not mediated by the src kinases Lyn and Fgr, since neither kinase coprecipitated with Syk. Syk from FMLP-activated, adherent PMNs phosphorylated paxillin-glutathione S-transferase, suggesting that paxillin is a substrate for Syk in vivo. These results indicate that PMN spreading and H2O2 release require a Syk-dependent signaling pathway leading to paxillin phosphorylation. 
 The protein-tyrosine kinase Syk interacts with the C-terminal region of tensin2. Syk is a 72-kDa protein-tyrosine kinase that regulates signaling through multiple cell surface receptors including those for antigens, immunoglobulins and proteins of the   extracellular matrix  . As part of its function, Syk binds a variety of downstream effectors through interactions that are often mediated by motifs that recognize phosphotyrosines. In a search for novel Syk-interacting proteins by yeast two-hybrid analysis, we identified tensin2 as a Syk-binding protein. Syk interacts with a fragment of tensin2 located near the C-terminus that contains SH2 and PTB domains. In epithelial cells, tensin2 localizes both to focal adhesions and to large cytoplasmic puncta. It is within these punctuate structures that Syk and tensin2 are co-localized. The clustering of Syk within these structures leads to its phosphorylation on tyrosine. 
  [Ii]nvasive  PubMed  
 Promoter hypermethylation of the 14-3-3 sigma, SYK and CAGE-1 genes is related to the various phenotypes of urinary bladder carcinomas and associated with progression of transitional cell carcinomas. To explore the significance of epigenetic mechanisms in urinary bladder carcinogenesis mediated by methylation of cytosine in CpG dinucleotides at 5' promoter regions, we analysed the methylation status of a broad panel of different genes in transitional cell carcinomas (TCC) and nonurothelial cancers, among which the 14-3-3 sigma, SYK and CAGE-1 genes were recognised as promising target genes. Using methylation-specific PCR, the rate of DNA hypermethylation proved to be related to the various histopathological cancer subtypes. The higher frequency of promoter methylation of the 14-3-3 sigma (57.1%) and SYK (64.3%) genes in high-grade, high-stage TCC in association with a reduced or even lacking immunohistochemical protein expression than in low-grade, low-stage TCC (28.6% and 42.9%, respectively), indicates that aberrant methylation of these genes plays an essential role in the progression of TCC. The importance of DNA hypermethylation in the conversion of TCC from a low to a high malignant potential was strongly supported by the finding that, unlike superficial low-grade TCC, advanced muscle   invasive   TCC showed a concurrent promoter methylation of the 14-3-3 sigma, SYK and CAGE-1 genes. Squamous cell carcinomas revealed a peak incidence of hypermethylation of the 14-3-3 sigma gene (80%), and conversely, the lowest methylation frequency of the SYK gene (13.3%). Undifferentiated small cell carcinomas disclosed a promoter methylation of the 14-3-3 sigma, SYK and CAGE-1 genes in only a quarter each for the cases. Although a correlation between the methylation status and gene activity in squamous cell and undifferentiated small cell carcinomas was not observed, the underexpression of the SYK protein products in both cancer types and additionally of the 14-3-3 sigma protein in small cell carcinomas appeared to be related to the aggressive clinical behaviour of both these nonurothelial bladder carcinomas. The relevance of the high frequency of DNA hypermethylation of the CAGE-1 antigen in TCC and squamous cell carcinomas merits further study, particularly in relation to anticancer immunotherapy. The methylation status of the PTEN, COX-2, RUNX-3 and HIC-1 genes was found to be unaltered. In conclusion, the different patterns of aberrant methylation of the 14-3-3 sigma, SYK and CAGE-1 genes in the various histopathological cancer types of the urinary bladder point to a role in tumor cell differentiation, resulting in the phenotypical conversion of TCC into nonurothelial carcinomas and in the progression of TCC to a more malignant potential. 
 Inhibitory effects of syk transfection on lung cancer cell invasion. Spleen tyrosine kinase (Syk) is closely related to tumor invasion and metastasis, and has been shown to have potential inhibitory effects in tumors. In this study, we constructed a eukaryotic expression vector for Syk and analyzed its effects on   invasive   ability of the A549 non-small cell lung cancer cell line in vitro.A fragment of Syk was obtained by RT-PCR from human lung cancer cells and cloned into the expression vector pLNCXSyk. After restriction endonuclease digestion, PCR and DNA sequencing confirmation, the recombinant Syk expression plasmid was transfected into A549 human lung cancer cells using lipofectamine protocols. After selection, the cells stably expressed Syk. Detection of Syk expression of the cells by RT-PCR, and   invasive   ability were examined.The eukaryotic expression plamid pLNCXSyk was constructed and expressed stably in the A549 human lung cancer cells. The RT-PCR results showed that Syk mRNA expression was upregulated significantly (P<0.05). Lower invasion through a basal membrane were apparent after transfection (P<0.05).A eukaryotic expression plasmid to cause Syk expression in lung cancer cells can obviously inhibit their   invasive   ability in vitro. 
 Involvement of the Syk-mTOR pathway in follicular lymphoma cell invasion and angiogenesis. Follicular lymphoma (FL) is the second-most common non-Hodgkin's lymphoma. The disease affects the lymph nodes, and 50% of patients present with bone marrow infiltration; however, the mechanisms involved in dissemination of the disease are not yet known. We previously reported that FL cells display an overexpression of Syk, a tyrosine kinase involved in many cellular processes including cell migration. Therefore, we sought to explore its role in the   invasive   process. Here, we show that FL patients display higher matrix metalloproteinase (MMP)-9 and vascular endothelial growth factor (VEGF) levels than healthy donors. Moreover, using Syk small interfering RNA and the Syk inhibitor R406, we demonstrate that, in FL cells, Syk is involved in the regulation of MMP-9 and VEGF expression, and that invasion and angiogenesis is mediated through a phosphatidylinositol-3 kinase (PI3K)-mammalian target of rapamycin module. Finally, using a FL xenograft mouse model we observe that fostamatinib (R788), inhibits MMP-9 expression and angiogenesis in vivo. Altogether, this study provides strong evidence that Syk represents an encouraging therapeutic target in FL and suggests the potential use of fostamatinib as an anti-  invasive   and anti-angiogenic drug. 
 Full-length spleen tyrosine kinase inhibits the invasion and metastasis of human laryngeal squamous cell carcinoma. This study aimed to investigate correlation between full-length spleen tyrosine kinase [SYK (L)] expression and clinical characteristics of laryngeal squamous cell carcinoma (LSCC), and explore effects of SYK (L) on invasion and metastasis of LSCC.The human laryngeal cancer Hep-2 cells with low SYK (L) expression were transfected with pIRES2-EGFP-SYK (L) vector and empty vector pIRES2-EGFP to generate Hep-2-SYK (L) cells and Hep-2-neo cells. The cell invasion and migration abilities were determined.The SYK (L) positive expression rate in LSCC tissues was significantly lower than in vocal cord dysplasia tissues and normal laryngeal tissues (P < 0.05). There was a significant correlation between SYK (L) expression and LSCC T stage, histopathological grade and lymph node metastasis (P < 0.05). mRNA expression of SYK (L) in Hep-2-SYK (L) cells was significantly higher than in Hep-2-neo cells and Hep-2 cells (P < 0.01). The protein expression of SYK (L) in Hep-2-SYK (L) cells was markedly higher than in Hep-2-neo cells and Hep-2 cells (P < 0.01). The number of   invasive   cells was significantly lower in Hep-2-SYK (L) group than in Hep-2-neo group and Hep-2 group (P < 0.01). The average number of migrating cells in Hep-2-SYK (L) group also markedly reduced as compared to Hep-2-neo group and Hep-2 group (P < 0.01).The SYK (L) expression was down-regulated in LSCC, which was closely correlated with cancer growth and lymph node metastasis. SYK (L) up-regulation was able to inhibit the invasion and metastasis of LSCC, therefore suppressing tumor development. Thus, SYK (L) may be a potential target for the LSCC treatment. 
 Role of the protein tyrosine kinase Syk in regulating cell-cell adhesion and motility in breast cancer cells. The expression of the Syk protein tyrosine kinase in breast cancer cells is inversely correlated with   invasive   growth and metastasis. The expression of Syk inhibits cell motility while supporting the formation of cell clusters by enhancing cell-cell contacts and promoting the redistribution of the adhesion proteins cortactin and vinculin to these contacts. Syk associates physically with cortactin and catalyzes its phosphorylation on tyrosine. The clustering of integrins leads to the phosphorylation of Syk and of numerous cellular proteins in a manner dependent on the activity of the kinase and on the presence of tyrosine 342 located in the linker B region. The ability of Syk to participate in integrin-mediated protein tyrosine phosphorylation correlates well with its ability to inhibit cell motility. 
 The prognostic value of Stathmin-1, S100A2, and SYK proteins in ER-positive primary breast cancer patients treated with adjuvant tamoxifen monotherapy: an immunohistochemical study. We recently found that DNA methylation of S100A2, spleen tyrosine kinase (SYK), and Stathmin-1 (STMN1) correlates with response to tamoxifen therapy in metastatic breast cancer. In this retrospective study, we investigated immunohistochemically whether these three markers are predictors of relapse in early breast cancer (EBC) patients treated with adjuvant tamoxifen alone.Immunohistochemical staining was performed for S100A2, SYK and STMN1 on a tissue microarray containing ER-positive   invasive   breast carcinomas from a study cohort of 215 operable breast cancer patients, who underwent radical local therapy and who were treated with adjuvant tamoxifen monotherapy. Cox regression was used to correlate staining intensity of the three markers with main endpoints in our study; disease-free survival (DFS), and disease-specific survival (DSS).In univariate analysis, only STMN1 staining intensity strongly correlated with DFS (P = 0.014) and DSS (P = 0.002). In the groups of low and high STMN1 intensity, DFS was 84% and 63%, and DSS was 89% and 70%. STMN1 retained its prognostic value for DFS (P = 0.002) and DSS (<0.001) in the multivariate model together with lymph node status. We found also a trend to better DFS in patients with low STMN1 intensity in both lymph node-positive (P = 0.001) and -negative patients (P = 0.065). As the tumour cells did not express S100A2 (except in one case) the potential prognostic value of this marker was not evaluated.Staining intensity of STMN1, but not SYK, predicted outcome in our collective of ER- positive tamoxifen treated EBC patients. 
 Aspergillus fumigatus stimulates the NLRP3 inflammasome through a pathway requiring ROS production and the Syk tyrosine kinase.   Invasive   aspergillosis (IA) is a life-threatening disease that occurs in immunodepressed patients when infected with Aspergillus fumigatus. This fungus is the second most-common causative agent of fungal disease after Candida albicans. Nevertheless, much remains to be learned about the mechanisms by which A. fulmigatus activates the innate immune system. We investigated the inflammatory response to conidia and hyphae of A. fumigatus and specifically, their capacity to trigger activation of an inflammasome. Our results show that in contrast to conidia, hyphal fragments induce NLRP3 inflammasome assembly, caspase-1 activation and IL-1beta release from a human monocyte cell line. The ability of Aspergillus hyphae to activate the NLRP3 inflammasome in the monocytes requires K(+) efflux and ROS production. In addition, our data show that NLRP3 inflammasome activation as well as pro-IL-1beta expression relies on the Syk tyrosine kinase, which is downstream from the pathogen recognition receptor Dectin-1, reinforcing the importance of Dectin-1 in the innate immune response against fungal infection. Furthermore, we show that treatment of monocytes with corticosteroids inhibits transcription of the gene encoding IL-1beta. Thus, our data demonstrate that the innate immune response against A. fumigatus infection involves a two step activation process, with a first signal promoting expression and synthesis of pro-IL-1beta; and a second signal, involving Syk-induced activation of the NLRP3 inflammasome and caspase-1, allowing processing and secretion of the mature cytokine. 
 The Epstein-Barr virus causes epithelial-mesenchymal transition in human corneal epithelial cells via Syk/src and Akt/Erk signaling pathways. Although Epstein-Barr virus (EBV)-associated keratitis is rare, it can cause acute corneal necrosis and neovascularization. We aimed to examine the signaling mechanism by which EBV causes epithelial-mesenchymal transition (EMT) in human corneal epithelial cells (HCECs) in vitro.The cellular response to EBV was assessed by real-time PCR, Western blot, migration assay, invasion assay, inhibitor assay, and ELISA assay.A model of EBV-induced EMT was established in HCECs. The EBV induced morphologic changes in the cells; the loss of epithelial markers E-cadherin, ZO-1, and β-catenin; and an increase in the mesenchymal markers N-cadherin, Vimentin, Snail, and TCF8/Zeb1. The EBV infection also led to the nuclear translocation of Snail and TCF8/Zeb1; enhanced the secretion of IL-6, IL-8, VEGF, TGF-β1, TNF-α, and MCP-1; and upregulated the expression of MMP2 and MMP9. The EBV-infected HCECs exhibited increased migration a  nd invas  iveness compared to uninfected HCECs. We measured the involvement of Syk, Src, PI3K/Akt, and Erk signaling, but not Smad, in EMT by EBV-induced TGF-β1. We demonstrated that treatment with TGF-β1, TGF-β receptors, Syk, or Src inhibitor blocked TGF-β1, Syk, or Src signaling activation, and EMT development by EBV. Moreover, these inhibitors prevented PI3K/Akt and Erk activation.An EBV infection in HCECs can lead to a mesenchymal fibroblast-like morphology, and cause EMT through the activation of PI3K/Akt and Erk by TGF-β1-mediated Syk and Src signaling. This phenomenon may have implications for EBV-associated keratitis and molecular approaches to treatment. 
 Alternative splicing disrupts a nuclear localization signal in spleen tyrosine kinase that is required for invasion suppression in breast cancer. Spleen tyrosine kinase (Syk) is a candidate tumor (metastasis) suppressor that is highly expressed in mammary epithelial cells. Loss of Syk expression through promoter hypermethylation is associated with increased   invasive  ness in a subset of breast cancer. Here, we show that in addition to full-length Syk [Syk(L)], an alternatively spliced variant, Syk(S), is frequently expressed in breast cancer cells. Syk(S) is identical to Syk(L), except that it lacks 23 amino acid residues (deletion) within the interdomain B (IDB) of Syk. We also show that the aberrant expression of Syk(S) occurs frequently in primary breast tumors but never in matched normal mammary tissues, suggesting a contribution of Syk(S) to mammary tumor progression. Expression of Syk(L) suppressed breast cancer cell   invasive  ness. In contrast, Syk(S) expression did not affect the cell invasion potential. This differential phenotypic response is accompanied by their different subcellular localization. Immunocytochemical studies and nuclear and cytoplasmic fractionation experiments indicated that Syk(L) could enter the nucleus, whereas Syk(S) was located exclusively in the cytoplasm. Five basic residues in deletion were found to be critical in determining Syk(L) nuclear transport and invasion suppression activity; mutations completely excluded Syk(L) from the nucleus and blocked Syk(L)-inducible invasion suppression. Moreover, IDB acted as an autonomous nuclear localization signal to facilitate nuclear transport of a heterologous protein. Thus, the IDB of Syk(L) contains a nuclear localization signal that is responsible for Syk(L) nuclear translocation. The correlation of the nuclear localization and invasion suppression function of Syk(L) indicated that nuclear Syk possesses biological activities associated with tumor suppression in mammary epithelial cells. 
 Spleen tyrosine kinase as a novel candidate tumor suppressor gene for human oral squamous cell carcinoma. We analyzed the mutational and methylation status of the spleen tyrosine kinase (Syk) gene and both mRNA and protein levels in primary oral squamous cell carcinoma (OSCC) and OSCC-derived cell lines and examined the function of the Syk gene in OSCC-derived cell lines in vitro. Using quantitative real-time reverse transcription polymerase chain reaction, Western blotting and immunofluorescence on 7 OSCC-derived cell lines and normal oral keratinocytes (NOKs), Syk mRNA and protein expression were commonly downregulated in all cell lines compared to the NOKs. Although no sequence variation in the coding region of the Syk gene was identified in these cell lines, we found frequent hypermethylation in the CpG island region. Syk expression was restored by experimental demethylation. In addition, using a wound healing assay and in vitro invasion assay, we performed functional analysis using Syk transfected into the OSCC-derived cell lines, and they showed significant inhibition of motility and   invasive  ness. In clinical samples, high frequencies of Syk downregulation were detected by immunohistochemistry (33 of 53 [62%]). Furthermore, the Syk expression status was correlated significantly (p = 0.047) with tumor metastasis to cervical lymph nodes. These results suggest that the Syk gene is frequently inactivated during oral carcinogenesis and that an epigenetic mechanism may regulate loss of expression possibly leading to metastasis. 
 Angiopoietin-like protein 2 renders colorectal cancer cells resistant to chemotherapy by activating spleen tyrosine kinase-phosphoinositide 3-kinase-dependent anti-apoptotic signaling. Angiopoietin-like protein 2 (ANGPTL2) plays an important role in inflammatory carcinogenesis and tumor metastasis by activating tumor angiogenesis and tumor cell chemotaxis and   invasive  ness. However, it is unclear whether ANGPTL2 expression has an effect on tumor cell survival. Here, we explored that possibility by determining whether ANGPTL2 expression altered survival of human colorectal cancer cell lines treated with antineoplastic drugs. To do so, we generated SW480 cells expressing ANGPTL2 (SW480/ANGPTL2) and control (SW480/Ctrl) cells. Apoptosis induced by antineoplastic drug treatment was significantly decreased in SW480/ANGPTL2 compared to control cells. Expression of anti-apoptotic BCL-2 family genes was upregulated in SW480/ANGPTL2 compared to SW480/Ctrl cells. To assess signaling downstream of ANGPTL2 underlying this effect, we carried out RNA sequencing analysis of SW480/ANGPTL2 and SW480/Ctrl cells. That analysis, combined with in vitro experiments, indicated that Syk-PI3K signaling induced expression of BCL-2 family genes in SW480/ANGPTL2 cells. Furthermore, ANGPTL2 increased its own expression in a feedback loop by activating the spleen tyrosine kinase-nuclear factor of activated T cells (Syk-NFAT) pathway. Finally, we observed a correlation between higher ANGPTL2 expression in primary unresectable tumors from colorectal cancer patients who underwent chemotherapy with a lower objective response rate. These findings suggest that attenuating ANGPTL2 signaling in tumor cells may block tumor cell resistance to antineoplastic therapies. 
 The non-receptor-associated tyrosine kinase Syk is a regulator of metastatic behavior in human melanoma cells. Melanoma is one of the most aggressive neoplastic transformations and characterized by a high metastatic potential. The current study was performed to assess the impact of "spleen tyrosine kinase" (Syk), a non-receptor-associated tyrosine kinase, on growth and metastatic behavior of melanoma cells in vitro and in a severe combined immunodeficient (SCID)-mouse/human-melanoma xenotransplantation model in vivo. Syk was expressed in melanocytes but was found to be downregulated in melanoma cells. Vector-driven expression of Syk in two different melanoma cell lines did not influence growth speed, but significantly reduced the   invasive   growth potential of both cell lines in a Matrigel assay in vitro. In a SCID-mouse/human melanoma xenotransplantation model, Syk expressing Mel-Juso cells exhibited delayed and reduced tumor growth. After intravenous as well as subcutaneous injection of tumor cells, Syk-transfected cells formed significantly fewer metastatic tumor lesions than control cells. The presented data define Syk as a novel regulator of metastatic behavior of melanoma cells. 
 The protein-tyrosine kinase Syk interacts with TRAF-interacting protein TRIP in breast epithelial cells. The nonreceptor, protein-tyrosine kinase Syk is a suppressor of breast cancer progression whose expression is inversely correlated with the   invasive   behavior of cancer cells. In contrast, Syk has a positive function in murine mammary tumor virus-mediated tumorigenesis. A yeast two-hybrid screen using a library from human mammary gland identified tumor necrosis factor (TNF) receptor-associated factor-interacting protein (TRIP) as an Syk-binding partner. This interaction is mediated by the C-terminal region of TRIP and is enhanced by the treatment of cells with TNF and the tyrosine phosphorylation of Syk. Syk and TRIP have opposing functions in TNF-signaling pathways. Syk enhances the activation of nuclear factor-kappaB by TNF and this is antagonized by TRIP. The overexpression of TRIP sensitizes cells to TNF-induced apoptosis, an effect that can be reversed by the coexpression of Syk. 
 The Syk tyrosine kinase suppresses malignant growth of human breast cancer cells. Syk is a protein tyrosine kinase that is widely expressed in haematopoietic cells. It is involved in coupling activated immunoreceptors to downstream signalling events that mediate diverse cellular responses including proliferation, differentiation and phagocytosis. Syk expression has been reported in cell lines of epithelial origin, but its function in these cells remains unknown. Here we show that Syk is commonly expressed in normal human breast tissue, benign breast lesions and low-tumorigenic breast cancer cell lines. Syk messenger RNA and protein, however, are low or undetectable in   invasive   breast carcinoma tissue and cell lines. Transfection of wild-type Syk into a Syk-negative breast cancer cell line markedly inhibited its tumour growth and metastasis formation in athymic mice. Conversely, overexpression of a kinase-deficient Syk in a Syk-positive breast cancer cell line significantly increased its tumour incidence and growth. Suppression of tumour growth by the reintroduction of Syk appeared to be the result of aberrant mitosis and cytokinesis. We propose that Syk is a potent modulator of epithelial cell growth and a potential tumour suppressor in human breast carcinomas. 
 Syk tyrosine kinase acts as a pancreatic adenocarcinoma tumor suppressor by regulating cellular growth and invasion. We have identified the nonreceptor tyrosine kinase syk as a marker of differentiation/tumor suppressor in pancreatic ductal adenocarcinoma (PDAC). Syk expression is lost in poorly differentiated PDAC cells in vitro and in situ, and stable reexpression of syk in endogenously syk-negative Panc1 (Panc1/syk) cells retarded their growth in vitro and in vivo and reduced anchorage-independent growth in vitro. Panc1/syk cells exhibited a more differentiated morphology and down-regulated cyclin D1, akt, and CD171, which are overexpressed by Panc1 cells. Loss of PDAC syk expression in culture is due to promoter methylation, and reversal of promoter methylation caused reexpression of syk and concomitant down-regulation of CD171. Moreover, suppression of syk expression in BxPC3 cells caused de novo CD171 expression, consistent with the reciprocal expression of syk and CD171 we observe in situ. Importantly, Panc1/syk cells demonstrated dramatically reduced invasion in vitro. Affymetrix analysis identified statistically significant regulation of >2000 gene products by syk in Panc1 cells. Of these, matrix metalloproteinase-2 (MMP2) and tissue inhibitor of metalloproteinase-2 were down-regulated, suggesting that the MMP2 axis might mediate Panc1/mock invasion. Accordingly, MMP2 inhibition suppressed the in vitro invasion of Panc1/mock cells without effect on Panc1/syk cells. This study demonstrates a prominent role for syk in regulating the differentiation state and   invasive   phenotype of PDAC cells. 
 SYK interaction with ITGβ4 suppressed by Epstein-Barr virus LMP2A modulates migration and invasion of nasopharyngeal carcinoma cells. Epstein-Barr virus (EBV)-encoded Latent Membrane Protein 2A (LMP2A) is an EBV latency-associated protein regularly expressed in nasopharyngeal carcinoma (NPC). In B cells, LMP2A activity resembles that of a constitutively activated antigen receptor, which recruits the Syk tyrosine kinase to activate a set of downstream signaling pathways. LMP2A also downregulates cellular Syk levels. In the present study, we demonstrate that Syk interacts with the integrin β4 subunit (ITGβ4) of integrin α6β4 in epithelial cells and that concurrent LMP2A expression interferes with this interaction by competitive binding to Syk. We find that both Syk and LMP2A have an effect on ITGβ4 cell surface expression. However, in LMP2A expressing cells, ITGβ4 remains concentrated at the cellular protrusions, an expression pattern characteristic of motile cells, including NPC-derived epithelial cells. This effect of LMP2A on ITGβ4 localization is associated with a greater propensity for migration and invasion in-vitro, and may contribute   to the   invasive property of LMP2A-expressing NPC. 
 The specific innate immune receptor CEACAM3 triggers neutrophil bactericidal activities via a Syk kinase-dependent pathway. The human-restricted pathogens Neisseria gonorrhoeae, Neisseria meningitidis, Haemophilus influenzae and Moraxella catarrhalis colonize host tissues via carcinoembryonic antigen-related cellular adhesion molecules (CEACAMs). One such receptor, CEACAM3, acts in a host-protective manner by orchestrating the capture and engulfment of   invasive   bacteria by human neutrophils. Herein, we show that bacterial binding to CEACAM3 causes recruitment of the cytoplasmic tyrosine kinase Syk, resulting in the phosphorylation of both CEACAM3 and Syk. This interaction is specific for the immunoreceptor tyrosine-based activation motif (ITAM) in the CEACAM3 cytoplasmic domain. While dispensable for the phagocytic uptake of single bacteria by CEACAM3, Syk is necessary for internalization when cargo size increases or when the density of CEACAM-binding ligand on the cargo surface is below a critical threshold. Moreover, Syk engagement is required for an effective bacterial killing response, including the neutrophil oxidative burst and degranulation functions in response to N. gonorrhoeae. These data reveal CEACAM3 as a specific innate immune receptor that mediates the opsonin-independent clearance of CEACAM-binding bacteria via Syk, a molecular trigger for functional immunoreceptor responses of both the adaptive (TCR, BCR, FcR) and innate (Dectin-1, CEACAM3) immune systems. 
 SYK allelic loss and the role of Syk-regulated genes in breast cancer survival. Heterozygotic loss of SYK, a non-receptor tyrosine kinase, gives rise to mouse mammary tumor formation where Syk protein levels are reduced by about half; loss of SYK mRNA is correlated with   invasive   cell behavior in in vitro models; and SYK loss has been correlated with distant metastases in patients. Here, allelic loss of the SYK gene was explored in breast ductal carcinoma in situ (DCIS) using fluorescence in situ hybridization and pyrosequencing, respectively, and in infiltrating ductal carcinoma (IDC) using genomic data from The Cancer Genome Atlas (TCGA). Allelic loss was present in a subset of DCIS cases where adjacent IDC was present. SYK copy number loss was found in about 26% of 1002 total breast cancer cases and 30% of IDC cases. Quantitative immunofluorescence revealed Syk protein to be six-fold higher in infiltrating immune cells compared with epithelial cells. This difference distorted tumor cell mRNA and protein levels in extracts. 20% of 1002 IDC cases contained elevated immune cell infiltration as estimated by elevated immune-specific mRNAs. In cases without immune cell infiltration, loss of SYK copy number was associated with a significant reduction of SYK mRNA. Here we define a 55 Gene Set consisting of Syk interacting, motility- and invasion-related genes. We found that overall survival was significantly reduced in IDC and Luminal A+B cases where copy number and mutations of these 55 genes were affected (Kaplan-Meier, Logrank test p-value 0.007141 and Logrank test p-value 0.001198, respectively). We conclude that reduction in Syk expression and contributions of genomic instability to copy number and mutations in the 55 Syk interacting genes significantly contribute to poorer overall patient survival. A closer examination of the role of Syk interacting motility and invasion genes and their prognostic and/or causative association with metastatic disease and patient outcome is warranted. 
  GeneRIFs  
 This study demonstrates a prominent role for syk in regulating the differentiation state and   invasive   phenotype of pancreatic ductal adenocarcinoma cells. 
  [Mm]etastasis  PubMed  
 Inhibitory effects of syk transfection on lung cancer cell invasion. Spleen tyrosine kinase (Syk) is closely related to tumor invasion and   metastasis  , and has been shown to have potential inhibitory effects in tumors. In this study, we constructed a eukaryotic expression vector for Syk and analyzed its effects on invasive ability of the A549 non-small cell lung cancer cell line in vitro.A fragment of Syk was obtained by RT-PCR from human lung cancer cells and cloned into the expression vector pLNCXSyk. After restriction endonuclease digestion, PCR and DNA sequencing confirmation, the recombinant Syk expression plasmid was transfected into A549 human lung cancer cells using lipofectamine protocols. After selection, the cells stably expressed Syk. Detection of Syk expression of the cells by RT-PCR, and invasive ability were examined.The eukaryotic expression plamid pLNCXSyk was constructed and expressed stably in the A549 human lung cancer cells. The RT-PCR results showed that Syk mRNA expression was upregulated significantly (P<0.05). Lower invasion through a basal membrane were apparent after transfection (P<0.05).A eukaryotic expression plasmid to cause Syk expression in lung cancer cells can obviously inhibit their invasive ability in vitro. 
 [Regulatory role of Syk gene on vascular endothelial growth factor C expression in breast cancer]. To investigate the effect of Syk on the VEGF-C expression in breast cancer.Immunohistochemical EnVision method was used to detect the protein expression of Syk, NFκB and VEGF-C in breast carcinoma; and the relationship between protein expression of Syk, NFκB, VEGF-C and lymph nod  e metastas  is was analysed. MDA-MB-231 cells were transfected with pcDNA3.1(-)-Syk, and the effect of Syk gene on the VEGF-C and NFκB expression was determined.In the lymph node metastatic group, a lower expression rate of Syk and higher expression rate of VEGF-C and NFκB were detected as compared to the non-metastatic group. The expression of Syk was negatively associated with NFκB (r = -0.448, P = 0.002) and VEGF-C (r = -0.620, P = 0.000) expression, and VEGF-C was associated with the nuclear expression of NFκB (r = 0.310, P = 0.036). Compared with the non-transfected cells, the pcDNA3.1(-)-Syk transfected MDA-MB-231 cells showed significantly lower transcriptional level of VEGF-C mRNA, expression level of VEGF-C protein and NFκB activity (P < 0.05).Syk may play an important role in the lymp  h node met  astasis of breast cancer. It may down-regulate the expression of VEGF-C by inhibiting the activity of NFκB, which thus suppresses lym  ph node me  tastasis of breast cancer. 
 [Expression of tyrosine kinase Syk in breast cancer and their clinical significance]. To evaluate the effects of the Syk mRNA expression in human breast cancer on tumor growth and   metastasis  , and to study the correlation of expression of the Syk gene with ER, PR, p53 and HER2/neu.Specimens from 40 breast cancer patients (tumor tissues, adjacent normal tissues), 15 fibroadenoma were detected for their expression of the Syk gene and level of Syk mRNA by semi-RT-PCR technique. Meanwhile, ER, PR, p53, HER2/neu were detected in 40 tumor tissues from breast cancer with immunohistochemical staining.All normal breast tissues were detected the expression of the Syk gene. Unlike normal breast tissue, 31 out of 40 breast cancer tissue did not show any detectable Syk mRNA expression, there were significant difference in two groups (chi(2) = 47.4, P < 0.05). The level of Syk mRNA in the primary breast cancer tissues were significantly lower than that in the adjacent non-cancerous breast tissues (t = 3.41, P < 0.05). Furthermore, only one breast cancer tissue in 18 patients with lymph node   metastasis   had the Syk mRNA expression, the rate and level of Syk mRNA expression in the patients with lymph node   metastasis   were lower than those without lymph node   metastasis   (chi(2) = 3.77, P < 0.05, t = 2.74, P < 0.05). Syk expression was correlated to p53 expression.The expression of the Syk gene may play an important role in suppressing growth and   metastasis   of breast cancer. 
 Hypermethylation of Syk gene in promoter region associated with oncogenesis and   metastasis   of gastric carcinoma. To investigate the relationship between methylation of Syk (spleen tyrosine kinase) gene in promoter region and oncogenesis,   metastasis   of gastric carcinoma. The relation between silencing of the Syk gene and methylation of Syk promoter region was also studied.By using methylation-specific PCR (MSP) technique, the methylation of Syk promoter region in specimens from 61 gastric cancer patients (tumor tissues and adjacent normal tissues) was detected. Meanwhile, RT-PCR was used to analyse syk expression exclusively.The expression of the Syk gene was detected in all normal gastric tissues. Syk expression in gastric carcinoma was lower in 14 out of 61 gastric cancer samples than in adjacent normal tissues (chi(2)=72.3, P<0.05). No methylation of Syk promoter was found in adjacent normal tissues. hypermethylation of Syk gene in promoter was detected 21 cases in 61 gastric carcinoma patients. The rate of methylation of Syk promoter in gastric carcinoma was higher than that in adjacent normal tissues (chi(2)=25.1, P<0.05). In 31 patients with lymph node   metastasis  , 17 were found with Syk promoter methylation. A significant difference was noted between two groups (chi(2)=11.4,P<0.05).Hypermethylation leads to silencing of the Syk gene in human gastric carcinoma. Methylation of Syk promoter is correlated to oncogenesis and   metastasis   of gastric carcinoma. Syk is considered to be a potential tumor suppressor and anti-  metastasis   gene in human gastric cancer. 
 Full-length spleen tyrosine kinase inhibits the invasion and   metastasis   of human laryngeal squamous cell carcinoma. This study aimed to investigate correlation between full-length spleen tyrosine kinase [SYK (L)] expression and clinical characteristics of laryngeal squamous cell carcinoma (LSCC), and explore effects of SYK (L) on invasion and   metastasis   of LSCC.The human laryngeal cancer Hep-2 cells with low SYK (L) expression were transfected with pIRES2-EGFP-SYK (L) vector and empty vector pIRES2-EGFP to generate Hep-2-SYK (L) cells and Hep-2-neo cells. The cell invasion and migration abilities were determined.The SYK (L) positive expression rate in LSCC tissues was significantly lower than in vocal cord dysplasia tissues and normal laryngeal tissues (P < 0.05). There was a significant correlation between SYK (L) expression and LSCC T stage, histopathological grade and lymph node   metastasis   (P < 0.05). mRNA expression of SYK (L) in Hep-2-SYK (L) cells was significantly higher than in Hep-2-neo cells and Hep-2 cells (P < 0.01). The protein expression of SYK (L) in Hep-2-SYK (L) cells was markedly higher than in Hep-2-neo cells and Hep-2 cells (P < 0.01). The number of invasive cells was significantly lower in Hep-2-SYK (L) group than in Hep-2-neo group and Hep-2 group (P < 0.01). The average number of migrating cells in Hep-2-SYK (L) group also markedly reduced as compared to Hep-2-neo group and Hep-2 group (P < 0.01).The SYK (L) expression was down-regulated in LSCC, which was closely correlated with cancer growth and lymph node   metastasis  . SYK (L) up-regulation was able to inhibit the invasion and   metastasis   of LSCC, therefore suppressing tumor development. Thus, SYK (L) may be a potential target for the LSCC treatment. 
 Role of the protein tyrosine kinase Syk in regulating cell-cell adhesion and motility in breast cancer cells. The expression of the Syk protein tyrosine kinase in breast cancer cells is inversely correlated with invasive growth and   metastasis  . The expression of Syk inhibits cell motility while supporting the formation of cell clusters by enhancing cell-cell contacts and promoting the redistribution of the adhesion proteins cortactin and vinculin to these contacts. Syk associates physically with cortactin and catalyzes its phosphorylation on tyrosine. The clustering of integrins leads to the phosphorylation of Syk and of numerous cellular proteins in a manner dependent on the activity of the kinase and on the presence of tyrosine 342 located in the linker B region. The ability of Syk to participate in integrin-mediated protein tyrosine phosphorylation correlates well with its ability to inhibit cell motility. 
 [Hypermethylation of syk gene in promoter region is associated with oncogenesis,   metastasis   of breast carcinoma]. To investigate the relationship between methylation of syk (spleen tyrosine kinase) gene in promoter region and oncogenesis,   metastasis   in breast carcinoma.Using RT-PCR technique, specimens from 40 breast cancer patients (tumor tissues, adjacent normal tissues), 15 fibroadenoma were detected for their expression of the syk gene. Meanwhile, Methylation-specific PCR was used to detect methylation of syk promoter region.The expression of syk gene were detected in normal breast tissues and fibroadenoma samples, 9 out of 40 breast carcinoma samples were found the expression of syk mRNA, syk expression in breast carcinoma was lower than that in adjacent normal tissues and fibroadenoma samples. No methylation of syk promoter was found in adjacent normal tissues and fibroadenoma samples, 17 cases in 40 breast carcinoma patients (42.5%) were detected hypermethylation of syk gene in promoter region, the rate of methylation of syk promoter in breast carcinoma was higher than that of in adjacent normal tissues (P < 0.05). In 18 cases with lymph node   metastasis  , 14 were found with syk promoter methylation. A significant difference was noted between two groups (P < 0.05).Hypermethylation leads to lose of the syk gene expression in human breast carcinoma. Methylation of syk promoter may be correlated to oncogenesis,   metastasis   of breast carcinoma. 
 A role for prohibitin in mast cell activation: location matters. Prohibitin 1 (PHB1) and PHB2 are evolutionarily conserved, ubiquitously expressed, pleotropic proteins that control various fundamental cellular processes, including proliferation, migration, metabolism, and death. Studies have unveiled a crucial role for plasma membrane-associated PHBs in regulating tumor   metastasis  , viral entry, and immune cell activation. A study now identifies a role for PHB1 in the activation of mast cells and allergic reactions mediated by immunoglobulin E (IgE). PHB1 was primarily localized in mast cell granules; however, in response to stimulation with antigen, PHB1 translocated to plasma membrane lipid rafts to form a ternary complex with the high-affinity IgE receptor FcεRIγ and the nonreceptor tyrosine kinase Syk. Syk became activated, which led to the activation of downstream signaling that stimulated mast cell degranulation and the secretion of cytokines. PHB1 was phosphorylated by the Src family tyrosine kinase Lyn, and palmitoylation of PHB1 was required for its association with the plasma membrane. These observations unveil a previously uncharacterized facet of prohibitin biology and shed further light on the proximal events that drive the activation of FcεRI by IgE in mast cells during allergic reactions. 
 Alternative splicing disrupts a nuclear localization signal in spleen tyrosine kinase that is required for invasion suppression in breast cancer. Spleen tyrosine kinase (Syk) is a candidate tumor (  metastasis  ) suppressor that is highly expressed in mammary epithelial cells. Loss of Syk expression through promoter hypermethylation is associated with increased invasiveness in a subset of breast cancer. Here, we show that in addition to full-length Syk [Syk(L)], an alternatively spliced variant, Syk(S), is frequently expressed in breast cancer cells. Syk(S) is identical to Syk(L), except that it lacks 23 amino acid residues (deletion) within the interdomain B (IDB) of Syk. We also show that the aberrant expression of Syk(S) occurs frequently in primary breast tumors but never in matched normal mammary tissues, suggesting a contribution of Syk(S) to mammary tumor progression. Expression of Syk(L) suppressed breast cancer cell invasiveness. In contrast, Syk(S) expression did not affect the cell invasion potential. This differential phenotypic response is accompanied by their different subcellular localization. Immunocytochemical studies and nuclear and cytoplasmic fractionation experiments indicated that Syk(L) could enter the nucleus, whereas Syk(S) was located exclusively in the cytoplasm. Five basic residues in deletion were found to be critical in determining Syk(L) nuclear transport and invasion suppression activity; mutations completely excluded Syk(L) from the nucleus and blocked Syk(L)-inducible invasion suppression. Moreover, IDB acted as an autonomous nuclear localization signal to facilitate nuclear transport of a heterologous protein. Thus, the IDB of Syk(L) contains a nuclear localization signal that is responsible for Syk(L) nuclear translocation. The correlation of the nuclear localization and invasion suppression function of Syk(L) indicated that nuclear Syk possesses biological activities associated with tumor suppression in mammary epithelial cells. 
 Spleen tyrosine kinase as a novel candidate tumor suppressor gene for human oral squamous cell carcinoma. We analyzed the mutational and methylation status of the spleen tyrosine kinase (Syk) gene and both mRNA and protein levels in primary oral squamous cell carcinoma (OSCC) and OSCC-derived cell lines and examined the function of the Syk gene in OSCC-derived cell lines in vitro. Using quantitative real-time reverse transcription polymerase chain reaction, Western blotting and immunofluorescence on 7 OSCC-derived cell lines and normal oral keratinocytes (NOKs), Syk mRNA and protein expression were commonly downregulated in all cell lines compared to the NOKs. Although no sequence variation in the coding region of the Syk gene was identified in these cell lines, we found frequent hypermethylation in the CpG island region. Syk expression was restored by experimental demethylation. In addition, using a wound healing assay and in vitro invasion assay, we performed functional analysis using Syk transfected into the OSCC-derived cell lines, and they showed significant inhibition of motility and invasiveness. In clinical samples, high frequencies of Syk downregulation were detected by immunohistochemistry (33 of 53 [62%]). Furthermore, the Syk expression status was correlated significantly (p = 0.047) with tumor   metastasis   to cervical lymph nodes. These results suggest that the Syk gene is frequently inactivated during oral carcinogenesis and that an epigenetic mechanism may regulate loss of expression possibly leading to   metastasis  . 
 Angiopoietin-like protein 2 renders colorectal cancer cells resistant to chemotherapy by activating spleen tyrosine kinase-phosphoinositide 3-kinase-dependent anti-apoptotic signaling. Angiopoietin-like protein 2 (ANGPTL2) plays an important role in inflammatory carcinogenesis and tumor   metastasis   by activating tumor angiogenesis and tumor cell chemotaxis and invasiveness. However, it is unclear whether ANGPTL2 expression has an effect on tumor cell survival. Here, we explored that possibility by determining whether ANGPTL2 expression altered survival of human colorectal cancer cell lines treated with antineoplastic drugs. To do so, we generated SW480 cells expressing ANGPTL2 (SW480/ANGPTL2) and control (SW480/Ctrl) cells. Apoptosis induced by antineoplastic drug treatment was significantly decreased in SW480/ANGPTL2 compared to control cells. Expression of anti-apoptotic BCL-2 family genes was upregulated in SW480/ANGPTL2 compared to SW480/Ctrl cells. To assess signaling downstream of ANGPTL2 underlying this effect, we carried out RNA sequencing analysis of SW480/ANGPTL2 and SW480/Ctrl cells. That analysis, combined with in vitro experiments, indicated that Syk-PI3K signaling induced expression of BCL-2 family genes in SW480/ANGPTL2 cells. Furthermore, ANGPTL2 increased its own expression in a feedback loop by activating the spleen tyrosine kinase-nuclear factor of activated T cells (Syk-NFAT) pathway. Finally, we observed a correlation between higher ANGPTL2 expression in primary unresectable tumors from colorectal cancer patients who underwent chemotherapy with a lower objective response rate. These findings suggest that attenuating ANGPTL2 signaling in tumor cells may block tumor cell resistance to antineoplastic therapies. 
 Tumor suppressor function of Syk in human MCF10A in vitro and normal mouse mammary epithelium in vivo. The normal function of Syk in epithelium of the developing or adult breast is not known, however, Syk suppresses tumor growth, invasion, and   metastasis   in breast cancer cells. Here, we demonstrate that in the mouse mammary gland, loss of one Syk allele profoundly increases proliferation and ductal branching and invasion of epithelial cells through the mammary fat pad during puberty. Mammary carcinomas develop by one year. Syk also suppresses proliferation and invasion in vitro. siRNA or shRNA knockdown of Syk in MCF10A breast epithelial cells dramatically increased proliferation, anchorage independent growth, cellular motility, and invasion, with formation of functional, extracellular matrix-degrading invadopodia. Morphological and gene microarray analysis following Syk knockdown revealed a loss of luminal and differentiated epithelial features with epithelial to mesenchymal transition and a gain in invadopodial cell surface markers CD44, CD49F, and MMP14. These results support the role of Syk in limiting proliferation and invasion of epithelial cells during normal morphogenesis, and emphasize the critical role of Syk as a tumor suppressor for breast cancer. The question of breast cancer risk following systemic anti-Syk therapy is raised since only partial loss of Syk was sufficient to induce mammary carcinomas. 
 The Syk tyrosine kinase suppresses malignant growth of human breast cancer cells. Syk is a protein tyrosine kinase that is widely expressed in haematopoietic cells. It is involved in coupling activated immunoreceptors to downstream signalling events that mediate diverse cellular responses including proliferation, differentiation and phagocytosis. Syk expression has been reported in cell lines of epithelial origin, but its function in these cells remains unknown. Here we show that Syk is commonly expressed in normal human breast tissue, benign breast lesions and low-tumorigenic breast cancer cell lines. Syk messenger RNA and protein, however, are low or undetectable in invasive breast carcinoma tissue and cell lines. Transfection of wild-type Syk into a Syk-negative breast cancer cell line markedly inhibited its tumour growth and   metastasis   formation in athymic mice. Conversely, overexpression of a kinase-deficient Syk in a Syk-positive breast cancer cell line significantly increased its tumour incidence and growth. Suppression of tumour growth by the reintroduction of Syk appeared to be the result of aberrant mitosis and cytokinesis. We propose that Syk is a potent modulator of epithelial cell growth and a potential tumour suppressor in human breast carcinomas. 
 Syk, a protein-tyrosine kinase, suppresses the cell motility and nuclear factor kappa B-mediated secretion of urokinase type plasminogen activator by inhibiting the phosphatidylinositol 3'-kinase activity in breast cancer cells. Tumor growth and   metastasis   are multifaceted processes that mainly involve cell adhesion, proteolytic degradation of the extracellular matrix, and cell migration. Syk is a member of a tyrosine kinase family that is expressed mostly in hematopoietic cells. Syk is expressed in cell lines of epithelial origin, but its function in these cells remains unknown. Here we report that Syk is expressed in MCF-7 cells but not in MDA-MB-231 cells. The overexpression of wild type Syk kinase but not kinase-negative Syk suppressed cell motility and inhibited the activation of phosphatidylinositol (PI) 3'-kinase in MDA-MB-231 cells. In contrast, when Syk-specific antisense S-oligonucleotide but not the sense S-oligonucleotide was transfected to MCF-7 cells the level of PI 3'-kinase activity as well as cell motility were increased. The MDA-MB-231 cells transfected with wild type Syk cDNA followed by treatment with piceatannol, a Syk inhibitor, enhanced cell motility and PI 3'-kinase activity. Pervanadate, a phosphotyrosine phosphatase inhibitor, induced PI 3'-kinase activity and stimulated the interaction between the inhibitor of nuclear factor kappa B alpha (I kappa B alpha) and the p85 alpha domain of PI 3'-kinase through tyrosine phosphorylation of the I kappa B alpha, which ultimately resulted in nuclear factor kappa B (NF kappa B) activation. Pervanadate had no effect on the activation of Syk in these cells. However, Syk suppressed the NF kappa B transcriptional activation and interaction between I kappa B alpha and PI 3'-kinase by inhibiting the tyrosine phosphorylation of I kappa B alpha. Syk, PI 3'-kinase inhibitors, and NF kappa B inhibitory peptide inhibited urokinase type plasminogen activator (uPA) secretion and cell motility in these cells. To our knowledge, this is the first report that Syk suppresses the cell motility and inhibits the PI 3'-kinase activity and uPA secretion by blocking NF kappa B activity through tyrosine phosphorylation of I kappa B alpha. These data further demonstrate a functional molecular link between Syk-regulated PI 3'-kinase activity and NF kappa B-mediated uPA secretion, and all of these ultimately control the motility of breast cancer cells. 
 Expression of variant isoforms of the tyrosine kinase SYK determines the prognosis of hepatocellular carcinoma. The spleen tyrosine kinase (SYK) has been reported as a novel biomarker for human hepatocellular carcinoma, but the functional contributions of its two isoforms SYK(L) and SYK(S) are undefined. In this study, we investigated their biologic functions and possible prognostic values in hepatocellular carcinoma. SYK(L) was downregulated in 38% of human specimens of hepatocellular carcinoma examined, whereas SYK(S) was detectable in 40% of these specimens but not in normal liver tissue samples without cirrhosis. SYK(S) expression correlated with pathologic parameters characteristic of tumor   metastasis  , including multiple tumors (P = 0.003) and vascular invasion (P = 0.001). Further, SYK(S) was specifically associated with epithelial-mesenchymal transition (EMT) in hepatocellular carcinoma specimens. Functional studies showed that SYK(S) promoted tumor growth, suppressed apoptosis, and induced EMT through the extracellular signal-regulated kinase pathway, countering the opposite effects of SYK(L). Patients with SYK(L(+)/S(-)) tumors exhibited longer overall survival and time to recurrence than those with SYK(L(-)/S(-)) or SYK(L(+)/S(+)) tumors (P < 0.001). Taken together, our findings showed that SYK(S) enhances invasion, whereas SYK(L) inhibits   metastasis   in hepatocellular carcinoma. We suggest that SYK(L) downregulation or SYK(S) elevation are strong predictors of poor survival in patients with hepatocellular carcinoma, indicative of a need for aggressive therapeutic intervention. 
 Involvement of syk and VEGF-C in invasion of lung adenocarcinoma A549 cells. Lung cancer has become one of the most dangerous malignant tumors in the world nowadays, whose pathogenesis is complex involving multi-genes and multi-elements. This study aims to investigate the values of spleen tyrosine kinase (Syk) and vascular endothelial growth factor-C (VEGF-C) in lymphangiogenesis and   metastasis   of lung adenocarcinoma A549 cells.The pcDNA3.1-VEGF-C and pLNCX-syk were constructed and transfected into A549 cells. After cells with stable expression were sorted, the level of VEGF-C was tested by RT-PCR and immunohistochemistry and the mRNA of syk was tested by RT-PCR. The cell invasion assay was investigated by transwell chamber in vitro. Restriction enzyme digestion and gel electrophoresis demonstrated successful construction of the pcDNA3.1-VEGF-C.RT-PCR and immunohistochemistry revealed higher expression of VEGF-C in VEGFC-construct-transfected A549 cells than that in controls (P < 0.05). Successful construction of the pLNCX-syk was demonstrated by restriction enzyme electrophoresis and sequencing. RT-PCR revealed Syk expression higher in syk-construct-transfected cells than in controls (P < 0.05).The results indicate a potential link between the upregulation of Syk and VEGF-C expression and lung adenocarcinoma. 
 Serum spleen tyrosine kinase and vascular endothelial growth factor-C levels predict lymph node   metastasis   of oesophageal squamous cell carcinoma. Lymph node involvement is a key feature and an independent prognostic factor of oesophageal squamous cell carcinoma. However, an accurate and robust assay to predict the lymphatic spread of oesophageal squamous cell carcinoma is unavailable. The purpose of this study was to determine whether serum vascular endothelial growth factor-C (VEGF-C) and spleen tyrosine kinase levels are potential markers of lymph node   metastasis   in patients with oesophageal squamous cell carcinoma.In the study, 108 patients with oesophageal squamous cell carcinoma and 24 healthy subjects participated. Serum spleen tyrosine kinase and VEGF-C concentrations were examined by enzyme-linked immunosorbent assays.Patients with oesophageal squamous cell carcinoma had significantly elevated VEGF-C and decreased spleen tyrosine kinase in serum when compared with normal controls (P < 0.05). Pearson's correlation analysis revealed that serum spleen tyrosine kinase was negatively correlated with the serum VEGF-C level in oesophageal squamous cell carcinoma patients (r = -0.453, P = 0.000). In addition, surgical resections of the oesophageal tumour profoundly brought both serum spleen tyrosine kinase and VEGF-C closer to the normal range at 2 weeks after operation (P < 0.05). Statistical analysis showed that enhanced serum VEGF-C and reduced spleen tyrosine kinase significantly correlated with the stage of regional lymph node   metastasis  , tumour size and the status of primary tumour extension, but not with other clinicopathological features of the patients (P < 0.05).Our data suggest that both serum spleen tyrosine kinase and VEGF-C levels are valuable in the preoperative evaluation of lymph node   metastasis   in patients with oesophageal squamous cell carcinoma. 
 Reduced expression of the Syk gene is correlated with poor prognosis in human breast cancer. Syk is a non-receptor type of protein-tyrosine kinase that is widely expressed in hematopoietic cells. Syk expression has been reported in breast tissue. However, the function of Syk in breast tissue remains unclear. In 25 (28%) of the 90 paired samples of human breast cancer and the matched, adjacent non-cancerous tissues examined here, reduced mRNA expression of Syk in the breast cancer tissues was observed by real-time quantitative reverse transcription-polymerase chain reaction. Eight (32%) of the 25 cases showing reduced Syk mRNA expression demonstrated distant   metastasis   of breast cancer, while only 4 (6%) of the 65 cases who did not show reduced Syk expression had distant   metastasis  . Kaplan-Meier curves demonstrated that patients with reduced Syk expression were at significantly increased risk for distant   metastasis   (P=0.0003). Our data suggest that reduced Syk expression in breast cancers is associated with distant   metastasis   and poor prognosis; therefore, Syk is considered to be a potential tumor suppressor and anti-  metastasis   gene in human breast cancer. 
  GeneRIFs  
 SYK expression was down-regulated in laryngeal squamous cell carcinoma, which was closely correlated with cancer growth and lymph node   metastasis  . SYK up-regulation was able to inhibit the invasion and   metastasis   of LSCC. 
 The purpose of this study was to determine whether serum vascular endothelial growth factor-C (VEGF-C) and spleen tyrosine kinase levels are potential markers of lymph node   metastasis   in patients with oesophageal squamous cell carcinoma. 
 Syk might down-regulate the expression of VEGF-C by inhibiting the activity of NFkappaB, which thus suppresses lymph node   metastasis   of breast cancer. 
 Syk gene is frequently inactivated during oral carcinogenesis and that an epigenetic mechanism may regulate loss of expression possibly leading to   metastasis  . 
 Our data suggest that reduced Syk expression in breast cancers is associated with distant   metastasis   and poor prognosis. 
 Methylation of Syk promoter is correlated to oncogenesis and   metastasis   of gastric carcinoma. 
 Hypermethylation leads to lose of the syk gene expression in human breast carcinoma and may be correlated to oncogenesis,   metastasis   of breast carcinoma. 
 The expression of the Syk gene may play an important role in suppressing growth and   metastasis   of breast cancer. 
  [Cc]ell [Ii]nvasion  PubMed  
 Inhibitory effects of syk transfection on lung cancer   cell invasion  . Spleen tyrosine kinase (Syk) is closely related to tumor invasion and metastasis, and has been shown to have potential inhibitory effects in tumors. In this study, we constructed a eukaryotic expression vector for Syk and analyzed its effects on invasive ability of the A549 non-small cell lung cancer cell line in vitro.A fragment of Syk was obtained by RT-PCR from human lung cancer cells and cloned into the expression vector pLNCXSyk. After restriction endonuclease digestion, PCR and DNA sequencing confirmation, the recombinant Syk expression plasmid was transfected into A549 human lung cancer cells using lipofectamine protocols. After selection, the cells stably expressed Syk. Detection of Syk expression of the cells by RT-PCR, and invasive ability were examined.The eukaryotic expression plamid pLNCXSyk was constructed and expressed stably in the A549 human lung cancer cells. The RT-PCR results showed that Syk mRNA expression was upregulated significantly (P<0.05). Lower invasion through a basal membrane were apparent after transfection (P<0.05).A eukaryotic expression plasmid to cause Syk expression in lung cancer cells can obviously inhibit their invasive ability in vitro. 
 Involvement of the Syk-mTOR pathway in follicular lymphoma   cell invasion   and angiogenesis. Follicular lymphoma (FL) is the second-most common non-Hodgkin's lymphoma. The disease affects the lymph nodes, and 50% of patients present with bone marrow infiltration; however, the mechanisms involved in dissemination of the disease are not yet known. We previously reported that FL cells display an overexpression of Syk, a tyrosine kinase involved in many cellular processes including cell migration. Therefore, we sought to explore its role in the invasive process. Here, we show that FL patients display higher matrix metalloproteinase (MMP)-9 and vascular endothelial growth factor (VEGF) levels than healthy donors. Moreover, using Syk small interfering RNA and the Syk inhibitor R406, we demonstrate that, in FL cells, Syk is involved in the regulation of MMP-9 and VEGF expression, and that invasion and angiogenesis is mediated through a phosphatidylinositol-3 kinase (PI3K)-mammalian target of rapamycin module. Finally, using a FL xenograft mouse model we observe that fostamatinib (R788), inhibits MMP-9 expression and angiogenesis in vivo. Altogether, this study provides strong evidence that Syk represents an encouraging therapeutic target in FL and suggests the potential use of fostamatinib as an anti-invasive and anti-angiogenic drug. 
 Full-length spleen tyrosine kinase inhibits the invasion and metastasis of human laryngeal squamous cell carcinoma. This study aimed to investigate correlation between full-length spleen tyrosine kinase [SYK (L)] expression and clinical characteristics of laryngeal squamous cell carcinoma (LSCC), and explore effects of SYK (L) on invasion and metastasis of LSCC.The human laryngeal cancer Hep-2 cells with low SYK (L) expression were transfected with pIRES2-EGFP-SYK (L) vector and empty vector pIRES2-EGFP to generate Hep-2-SYK (L) cells and Hep-2-neo cells. The   cell invasion   and migration abilities were determined.The SYK (L) positive expression rate in LSCC tissues was significantly lower than in vocal cord dysplasia tissues and normal laryngeal tissues (P < 0.05). There was a significant correlation between SYK (L) expression and LSCC T stage, histopathological grade and lymph node metastasis (P < 0.05). mRNA expression of SYK (L) in Hep-2-SYK (L) cells was significantly higher than in Hep-2-neo cells and Hep-2 cells (P < 0.01). The protein expression of SYK (L) in Hep-2-SYK (L) cells was markedly higher than in Hep-2-neo cells and Hep-2 cells (P < 0.01). The number of invasive cells was significantly lower in Hep-2-SYK (L) group than in Hep-2-neo group and Hep-2 group (P < 0.01). The average number of migrating cells in Hep-2-SYK (L) group also markedly reduced as compared to Hep-2-neo group and Hep-2 group (P < 0.01).The SYK (L) expression was down-regulated in LSCC, which was closely correlated with cancer growth and lymph node metastasis. SYK (L) up-regulation was able to inhibit the invasion and metastasis of LSCC, therefore suppressing tumor development. Thus, SYK (L) may be a potential target for the LSCC treatment. 
 Alternative splicing disrupts a nuclear localization signal in spleen tyrosine kinase that is required for invasion suppression in breast cancer. Spleen tyrosine kinase (Syk) is a candidate tumor (metastasis) suppressor that is highly expressed in mammary epithelial cells. Loss of Syk expression through promoter hypermethylation is associated with increased invasiveness in a subset of breast cancer. Here, we show that in addition to full-length Syk [Syk(L)], an alternatively spliced variant, Syk(S), is frequently expressed in breast cancer cells. Syk(S) is identical to Syk(L), except that it lacks 23 amino acid residues (deletion) within the interdomain B (IDB) of Syk. We also show that the aberrant expression of Syk(S) occurs frequently in primary breast tumors but never in matched normal mammary tissues, suggesting a contribution of Syk(S) to mammary tumor progression. Expression of Syk(L) suppressed breast cancer cell invasiveness. In contrast, Syk(S) expression did not affect the   cell invasion   potential. This differential phenotypic response is accompanied by their different subcellular localization. Immunocytochemical studies and nuclear and cytoplasmic fractionation experiments indicated that Syk(L) could enter the nucleus, whereas Syk(S) was located exclusively in the cytoplasm. Five basic residues in deletion were found to be critical in determining Syk(L) nuclear transport and invasion suppression activity; mutations completely excluded Syk(L) from the nucleus and blocked Syk(L)-inducible invasion suppression. Moreover, IDB acted as an autonomous nuclear localization signal to facilitate nuclear transport of a heterologous protein. Thus, the IDB of Syk(L) contains a nuclear localization signal that is responsible for Syk(L) nuclear translocation. The correlation of the nuclear localization and invasion suppression function of Syk(L) indicated that nuclear Syk possesses biological activities associated with tumor suppression in mammary epithelial cells. 
 Involvement of syk and VEGF-C in invasion of lung adenocarcinoma A549 cells. Lung cancer has become one of the most dangerous malignant tumors in the world nowadays, whose pathogenesis is complex involving multi-genes and multi-elements. This study aims to investigate the values of spleen tyrosine kinase (Syk) and vascular endothelial growth factor-C (VEGF-C) in lymphangiogenesis and metastasis of lung adenocarcinoma A549 cells.The pcDNA3.1-VEGF-C and pLNCX-syk were constructed and transfected into A549 cells. After cells with stable expression were sorted, the level of VEGF-C was tested by RT-PCR and immunohistochemistry and the mRNA of syk was tested by RT-PCR. The   cell invasion   assay was investigated by transwell chamber in vitro. Restriction enzyme digestion and gel electrophoresis demonstrated successful construction of the pcDNA3.1-VEGF-C.RT-PCR and immunohistochemistry revealed higher expression of VEGF-C in VEGFC-construct-transfected A549 cells than that in controls (P < 0.05). Successful construction of the pLNCX-syk was demonstrated by restriction enzyme electrophoresis and sequencing. RT-PCR revealed Syk expression higher in syk-construct-transfected cells than in controls (P < 0.05).The results indicate a potential link between the upregulation of Syk and VEGF-C expression and lung adenocarcinoma. 
 Association of SYK genetic variations with breast cancer pathogenesis. Spleen tyrosine kinase (SYK) is a non-receptor type cytoplasmic protein and a known tumor suppressor gene in breast cancer. Polymorphisms in SYK have been reported to be associated with   cell invasion  /cell morality and an increased risk of cancer development. In this case control study, all exons of the SYK gene and its exon/ intron boundaries were amplified in 200 breast cancer cases and 100 matched controls and then analyzed by single stranded conformational polymorphism. Amplified products showing altered mobility patterns were sequenced and analyzed. Twelve variations were identified in exonic and intronic regions of DNA encoding SH2 domain and kinase domain of the SYK gene. All of these mutations are novel. Among them, 5 missense mutations were observed in exon 15 while one missense mutation was found in exon 8. In addition to these mutations, six mutations were also identified in intronic regions. We found a significant association between SYK mutations and breast cancer and observed that Glu241Arg, a missense mutation is associated with an increase risk of ~7 fold (OR=6.7, 95% CI=1.54-28.8), Thr581Pro (missense mutation) is associated with increased risk of ~16 fold (OR=15.5, 95%CI=2.07-115.45) and 63367 T>G (missense mutation) is associated with increased risk of ~13 fold (OR=12.8, 95%CI=1.71-96.71) for breast cancer. Significant associations were observed for each of these variations with both late menopause (p<0.01) and early menarche (p<0.005) cases when compared to controls. Our findings suggest that the polymorphic gene SYK may contribute to the development of breast cancer in at least the Pakistani population. This study provides an insight view of SYK which may provide a significant finding for the pharmaceutical and biotechnology industry. 
  GeneRIFs  
 Syk-mTOR pathway is involed in follicular lymphoma   cell invasion   and angiogenesis. 
   
  
 ZAP70 
 
  NCBI Gene  
  GeneRIF  
  Pubmed  
 
 
  Search Term  Rank  Fields  
  [Mm]igration  6  Process(1); PubMed(14); GeneRIFs(7)  
  [Ii]nvasive  10  PubMed(3)  
  [Mm]etastasis  12  PubMed(2)  
 
 
 hide/show details for ZAP70 
  [Mm]igration  Process  
 T cell   migration   
  PubMed  
 CBAP functions as a novel component in chemokine-induced ZAP70-mediated T-cell adhesion and   migration  . Activated chemokine receptor initiates inside-out signaling to transiently trigger activation of integrins, a process involving multiple components that have not been fully characterized. Here we report that GM-CSF/IL-3/IL-5 receptor common beta-chain-associated protein (CBAP) is required to optimize this inside-out signaling and activation of integrins. First, knockdown of CBAP expression in human Jurkat T cells caused attenuated CXC chemokine ligand-12 (CXCL12)-induced cell   migration   and integrin α4β1- and αLβ2-mediated cell adhesion in vitro, which could be rescued sufficiently upon expression of murine CBAP proteins. Freshly isolated CBAP-deficient primary T cells also exhibited diminution of chemotaxis toward CC chemokine ligand-21 (CCL21) and CXCL12, and these chemokines-induced T-cell adhesions in vitro. Adoptive transfer of isolated naive T cells demonstrated that CBAP deficiency significantly reduced lymph node homing ability in vivo. Final  ly, migra  tion of T cell-receptor-activated T cells induced by inflammatory chemokines was also attenuated in CBAP-deficient cells. Further analyses revealed that CBAP constitutively associated with both integrin β1 and ZAP70 and that CBAP is required for chemokine-induced initial binding of the talin-Vav1 complex to integrin β1 and to facilitate subsequent ZAP70-mediated dissociation of the talin-Vav1 complex and Vav1 phosphorylation. Within such an integrin signaling complex, CBAP likely functions as an adaptor and ultimately leads to activation of both integrin α4β1 and Rac1. Taken together, our data suggest that CBAP indeed can function as a novel signaling component within the ZAP70/Vav1/talin complex and plays an important role in regulating chemokine-promoted T-cell trafficking. 
 CBAP functions as a novel component in chemokine-induced ZAP70-mediated T-cell adhesion and   migration  . Activated chemokine receptor initiates inside-out signaling to transiently trigger activation of integrins, a process involving multiple components that have not been fully characterized. Here we report that GM-CSF/IL-3/IL-5 receptor common beta-chain-associated protein (CBAP) is required to optimize this inside-out signaling and activation of integrins. First, knockdown of CBAP expression in human Jurkat T cells caused attenuated CXC chemokine ligand-12 (CXCL12)-induced cell   migration   and integrin α4β1- and αLβ2-mediated cell adhesion in vitro, which could be rescued sufficiently upon expression of murine CBAP proteins. Freshly isolated CBAP-deficient primary T cells also exhibited diminution of chemotaxis toward CC chemokine ligand-21 (CCL21) and CXCL12, and these chemokines-induced T-cell adhesions in vitro. Adoptive transfer of isolated naive T cells demonstrated that CBAP deficiency significantly reduced lymph node homing ability in vivo. Final  ly, migra  tion of T cell-receptor-activated T cells induced by inflammatory chemokines was also attenuated in CBAP-deficient cells. Further analyses revealed that CBAP constitutively associated with both integrin β1 and ZAP70 and that CBAP is required for chemokine-induced initial binding of the talin-Vav1 complex to integrin β1 and to facilitate subsequent ZAP70-mediated dissociation of the talin-Vav1 complex and Vav1 phosphorylation. Within such an integrin signaling complex, CBAP likely functions as an adaptor and ultimately leads to activation of both integrin α4β1 and Rac1. Taken together, our data suggest that CBAP indeed can function as a novel signaling component within the ZAP70/Vav1/talin complex and plays an important role in regulating chemokine-promoted T-cell trafficking. 
 ZAP-70 promotes the infiltration of malignant B-lymphocytes into the bone marrow by enhancing signaling and   migration   after CXCR4 stimulation. ZAP-70 in chronic lymphocytic leukemia (CLL) is associated with enhanced response to microenvironmental stimuli. We analyzed the functional consequences of ZAP-70 ectopic expression in malignant B-cells in a xenograft mouse model of disseminated B-cell leukemia. Mice injected with B-cells expressing ZAP-70 showed a prominently higher infiltration of the bone marrow. In vitro analysis of the response of malignant B-cells to CXCL12, the main attracting chemokine regulating trafficking of lymphocytes to the bone marrow, or to bone marrow stromal cells, revealed that ZAP-70 induces an increased response in terms of signaling and   migration  . These effects are probably mediated by direct participation of ZAP-70 in CXCL12-CXCR4 signaling since CXCR4 stimulation led to activation of ZAP-70 and downstream signaling pathways, such as MAPK and Akt, whereas ZAP-70 did not alter the expression of the CXCR4 receptor. In addition, subclones of primary CLL cells with high expression of ZAP-70 also showed increased migrative capacity toward CXCL12. Neutralization of CXCR4 with a monoclonal antibody resulted in impaired in vitro responses to CXCL12 and bone marrow stromal cells. We conclude that ZAP-70 enhances the   migration   of malignant B-cells into the supportive microenvironment found in the bone marrow mainly by enhancing signaling and   migration   after CXCR4 stimulation. 
 G alpha i2 and ZAP-70 mediate RasGRP1 membrane localization and activation of SDF-1-induced T cell functions. RasGRP1, a Ras guanine-nucleotide exchange factor, critically mediates T cell development and function and controls immunodeficiency and autoimmunity. In this study, we describe a unique mechanism of mobilization and activation of RasGRP1 in response to SDF-1, a chemokine that signals via the G protein-coupled receptor CXCR4. Depletion of RasGRP1 impaired SDF-1-stimulated human T cell   migration  , expression of the activation marker CD69, and activation of the ERK MAPK pathway, indicating that RasGRP1 mediates SDF-1 functions. SDF-1 treatment caused RasGRP1 to localize to the plasma membrane to activate K-Ras and to the Golgi to activate N-Ras. These events were required for cellular   migration   and for ERK activation that mediates downstream transcriptional events in response to SDF-1. SDF-1-dependent localization of RasGRP1 did not require its diacylglycerol-binding domain, even though diacyglycerol was previously shown to mediate localization of RasGRP1 in response to Ag stimulation. This domain was, however, required for activity of RasGRP1 after its localization. Intriguingly, SDF-1 treatment of T cells induced the formation of a novel molecular signaling complex containing RasGRP1, Gαi2, and ZAP-70. Moreover, SDF-1-mediated signaling by both Gi proteins and ZAP-70 was required for RasGRP1 mobilization. In addition, RasGRP1 mobilization and activation in response to SDF-1 was dependent on TCR expression, suggesting that CXCR4 heterodimerizes with the TCR to couple to ZAP-70 and mobilize RasGRP1. These results increase understanding of the molecular mechanisms that mediate SDF-1 effects on T cells and reveal a novel mechanism of RasGRP1 regulation. Other G protein-coupled receptors may similarly contribute to regulation of RasGRP1. 
 MiR-631/ZAP70: A novel axis in the   migration   and invasion of prostate cancer cells. MicroRNAs (miRNAs) are short, endogenous non-coding RNA molecules involved in cancer initiation and progression. Using transwell   migration   and invasion assays, we found that miR-631 inhibited the   migration   and invasion of prostate cancer (PCa) cells. Bioinformatic algorithms indicated the 3'-untranslated region (3'-UTR) of zeta-associated protein 70 (ZAP70) has a putative binding site for miR-631. We found that miR-631 can bind to the 3'-UTR of ZAP70 and decrease its expression. Further studies confirmed that ZAP70 facilitates PCa cell   migration   and invasion. Interestingly, using gain- and loss-of function experiments, we found that ZAP70 is a major target of miR-631 and largely mediates its activity. In addition, we further discovered that miR-631 was downregulated and ZAP70 was overexpressed in PCa cell lines and PCa tissues. A concordant inverse correlation between miR-631 and ZAP70 was also found in PCa tissues. In all, our study demonstrates that miR-631 decreases PCa cell   migration   and invasion by dampening ZAP70 expression. 
 Zap70 controls the interaction of talin with integrin to regulate the chemotactic directionality of T-cell   migration  . Aberrant lymphocyte infiltration is crucial for many disorders such as tumor immune escape and autoimmunity. In this study, we have investigated T-cell   migration   in a three-dimensional collagen matrix containing tumor spheroids and by using micro-Slide chemotaxis and found that Zap70 regulates directionality during cell chemotaxis. Jurkat cells actively migrated toward SDF-1, nutrition, and spheroids of MCF-7 breast carcinoma cells embedded in collagen matrix. Inhibition of Zap70 activity impaired trans  migration   and mu-Slide chemotaxis but not the random movement of T cells in the collagen/fibronectin matrix. P116 cells, a Zap70 deficient variant of Jurkat, showed active random movement but failed to migrate against chemoattractants. P116 cells exhibited a reduced polarization of cell morphology, showing less lamellipodia formation accompanied with a fast pseudopod turnover rate. Instead of direct interacting with F-actin, Zap70 formed a complex with talin which is an integrin scaffold for F-actin. SDF-1 enhanced Zap70 phosphorylation and also stimulated binding of talin and beta1 integrin activation. P116 cells showed reduced complex of talin and beta1 integrin in parallel with impaired integrin activation. Collectively, Zap70 modulates integrin activation by interacting with talin, which contributes to directionality of T-cell   migration  , severing as a potential target for anti-inflammation therapy. 
 CXCL12-induced chemotaxis is impaired in T cells from patients with ZAP-70-negative chronic lymphocytic leukemia. T cells from patients with chronic lymphocytic leukemia may play an important role in contributing to the onset, sustenance, and exacerbation of the disease by providing survival and proliferative signals to the leukemic clone within lymph nodes and bone marrow.By performing chemotaxis assays towards CXCL12, CCL21 and CCL19, we sought to evaluate the migratory potential of T cells from chronic lymphocytic leukemia patients. We next analyzed the chemokine-induced   migration   of T cells, dividing the chronic lymphocytic leukemia samples according to their expression of the poor prognostic factors CD38 and ZAP-70 in leukemic cells determined by flow cytometry.We found that T cells from patients with chronic lymphocytic leukemia are less responsive to CXCL12, CCL21 and CCL19 than T cells from healthy adults despite similar CXCR4 and CCR7 expression. Following separation of the patients into two groups according to ZAP-70 expression, we found that T cells from ZAP-70-negative samples showed significantly less   migration   towards CXCL12 compared to T cells from ZAP-70-positive samples and that this was not due to defective CXCR4 down-regulation, F-actin polymerization or to a lesser expression of ZAP-70, CD3, CD45, CD38 or CXCR7 on these cells. Interestingly, we found that leukemic cells from ZAP-70-negative samples seem to be responsible for the defective CXCR4 migratory response observed in their T cells.Impaired   migration   towards CXCL12 may reduce the access of T cells from ZAP-70-negative patients to lymphoid organs, creating a less favorable microenvironment for leukemic cell survival and proliferation. 
 MIF interacts with CXCR7 to promote receptor internalization, ERK1/2 and ZAP-70 signaling, and lymphocyte chemotaxis. Macrophage   migration  -inhibitory factor (MIF) is a pleiotropic cytokine with chemokine-like functions and is a mediator in numerous inflammatory conditions. Depending on the context, MIF signals through 1 or more of its receptors cluster of differentiation (CD)74, CXC-motif chemokine receptor (CXCR)2, and CXCR4. In addition, heteromeric receptor complexes have been identified. We characterized the atypical chemokine receptor CXCR7 as a novel receptor for MIF. MIF promoted human CXCR7 internalization up to 40%, peaking at 50-400 nM and 30 min, but CXCR7 internalization by MIF was not dependent on CXCR4. Yet, by coimmunoprecipitation, fluorescence microscopy, and a proximity ligation assay, CXCR7 was found to engage in MIF receptor complexes with CXCR4 and CD74, both after ectopic overexpression and in endogenous conditions in a human B-cell line. Receptor competition binding and coimmunoprecipitation studies combined with sulfo-SBED-biotin-transfer provided evidence for a direct interaction between MIF and CXCR7. Finally, we demonstrated MIF/CXCR7-mediated functional responses. Blockade of CXCR7 suppressed MIF-mediated ERK- and zeta-chain-associated protein kinase (ZAP)-70 activation (from 2.1- to 1.2-fold and from 2.5- to 1.6-fold, respectively) and fully abrogated primary murine B-cell chemotaxis triggered by MIF, but not by CXCL12. B cells from Cxcr7(-/-) mice exhibited an ablated trans  migration   response to MIF, indicating that CXCR7 is essential for MIF-promoted B-cell   migration  . Our findings provide biochemical and functional evidence that MIF is an alternative ligand of CXCR7 and suggest a functional role of the MIF-CXCR7 axis in B-lymphocyte   migration  . 
 Differential expression and function of CD27 in chronic lymphocytic leukemia cells expressing ZAP-70. Chronic lymphocytic leukemia is a malignancy driven by abberant B cell signaling and survival. Leukemic B cells accumulate in the peripheral blood and the lymphoid organs where contact with stromal cells and T cells provide critical survival signals. Clinical severity of CLL is associated with several prognostic markers including expression of the kinase ZAP-70. ZAP-70 expression enhances signaling via the B cell antigen receptor and is associated with increased cell adhesion and   migration   capacity. Here we report that ZAP-70-positive CLL patients display significantly higher expression of the TNF superfamily receptor and memory marker CD27 than do ZAP-70 negative patients. CD27 expression by CLL was acutely elevated upon BCR cross-linking, or upon ectopic expression of ZAP-70. CD27 expression correlated with functional capacity to adhere to stromal cells and antibody blockade of CD27 impaired CLL binding to stroma. These results provide the first evidence for differential expression of CD27 among CLL prognostic groups, suggest a role for ZAP-70 dependent signaling in CD27 induction and implicate CD27 in cell-cell interactions with the lymphoid tissue microenvironment. 
 Gene expression profiling reveals differences in microenvironment interaction between patients with chronic lymphocytic leukemia expressing high versus low ZAP70 mRNA. Zeta-associated protein 70 (ZAP70) is a widely recognized prognostic factor in chronic lymphocytic leukemia, but mechanisms by which its higher expression leads to a poor outcome must still be fully explained.In an attempt to unveil unfavorable cellular properties linked to high ZAP70 expression, we used gene expression profiling to identify genes associated with disparities in B cells from chronic lymphocytic leukemia patients expressing high versus low ZAP70 mRNA, measured by quantitative real-time PCR. Two groups of 7 patients were compared, selected on the basis of either high or low ZAP70 mRNA expression.Twenty-seven genes were differentially expressed with an FDR<10%, and several genes were significant predictors of treatment-free survival (TFS) and/or overall survival; PDE8A and FCRL family genes (down-regulated in ZAP70(+) patients) could predict TFS and overall survival; ITGA4 mRNA (up-regulated in ZAP70(+) patients) could significantly predict overall survival. Importantly, gene set enrichment analysis revealed overrepresentation of adhesion/  migration   genes. We therefore investigated in vitro adhesion/  migration   capacity of chronic lymphocytic leukemia cells into a stromal microenvironment or in response to conditioned medium. We showed that ZAP70(+) cells had better adhesion/  migration   capacities and only ZAP70(+) patient cells responded to microenvironment contact by CXCR4 downregulation.We concluded that several prognostic factors are the reflection of microenvironment interactions and that the increased adhesion/migratory capacity of ZAP70(+) cells in their microenvironment can explain their better survival and thus the aggressiveness of the disease. 
 The integrin LFA-1 signals through ZAP-70 to regulate expression of high-affinity LFA-1 on T lymphocytes. The integrin lymphocyte function-associated antigen 1 (LFA-1) controls many functions of T lymphocytes and is particularly essential during lymphocyte   migration   from blood into tissues. LFA-1 is considered to initiate "outside-in" signaling when bound to ligand intercellular adhesion molecule 1 (ICAM-1), but little is known about the proteins involved or where in the cell such LFA-1-mediated signaling might be operating. Here we show that LFA-1 is constitutively associated with the protein tyrosine kinases Lck and zeta chain-associated protein of 70 kDa (ZAP-70). When LFA-1 binds ICAM-1, both kinases become phosphorylated and the consequence of kinase activation is the conversion of intermediate- to high-affinity LFA-1 and an increase in close contact with ICAM-1. In the polarized T lymphocyte, phospho-ZAP-70 is concentrated within a region of high-affinity LFA-1 that includes talin and encompasses the lamella/lamellipodial interface as well as further back in the cell. Deficiency of ZAP-70 through inhibition or knockdown in T lymphocytes decreases the speed of   migration   on ICAM-1, as well as reducing firm adhesion under shear-flow conditions. Through its control of high-affinity LFA-1, the LFA-1/Lck/ZAP-70 complex is in position to initiate the rapid adhesion strengthening and   migration   necessary for T-lymphocyte responses when stimulated vasculature is encountered at sites of infection or injury. 
 Signaling through ZAP-70 is required for CXCL12-mediated T-cell transendothelial   migration  . Transendothelial   migration   of activated lymphocytes from the blood into the tissues is an essential step for immune functions. The housekeeping chemokine CXCL12 (or stroma cell-derived factor-1alpha), a highly efficient chemoattractant for T lymphocytes, drives lymphocytes to sites where they are highly likely to encounter antigens. This suggests that cross-talk between the T-cell receptor (TCR) and CXCR4 (the CXCL12 receptor) might occur within these sites. Here we show that the zeta-associated protein 70 (ZAP-70), a key element in TCR signaling, is required for CXCR4 signal transduction. The pharmacologic inhibition of ZAP-70, or the absence of ZAP-70 in Jurkat T cells and in primary CD4(+) T cells obtained from a patient with ZAP deficiency, resulted in an impairment of transendothelial   migration   that was rescued by the transfection of ZAP-70. Moreover, the overexpression of mutated forms of ZAP-70, whose kinase domain was inactivated, also abrogated the migratory response of Jurkat T cells to CXCL12. In contrast, no involvement of ZAP-70 in T-cell arrest on inflammatory endothelium under flow conditions or in CXCL12-induced actin polymerization was observed. Furthermore, CXCL12 induced time-dependent phosphorylation of ZAP-70, Vav1, and extracellular signal-regulated kinases (ERKs); the latter were reduced in the absence of functional ZAP-70. However, though a dominant-negative Vav1 mutant (Vav1 L213A) blocked CXCL12-induced T-cell   migration  , pharmacologic inhibition of the ERK pathway did not affect   migration  , suggesting that ERK activation is dispensable for T-cell chemotaxis. We conclude that cross-talk between the ZAP-70 signaling pathway and the chemokine receptor CXCR4 is required for T-cell   migration  . 
 ROZA-XL, an improved FRET based biosensor with an increased dynamic range for visualizing zeta associated protein 70 kD (ZAP-70) tyrosine kinase activity in live T cells. Genetically encoded FRET based biosensors allow one to visualize the spatial and temporal evolution of specific enzyme activities in live cells. We have previously reported the creation of a FRET based biosensor specific for Zeta-Associated Protein -70 kD (ZAP-70) (Randriamampita et al., 2008), a Syk family protein tyrosine kinase. ZAP-70 is essential for early T cell receptor (TCR) signaling events, T lymphocyte development and has also been implicated in integrin mediated T lymphocyte   migration  . In order to facilitate the study of ZAP-70 kinase activity during dynamic phenomena such as immunological synapse formation or cell   migration  , we have designed and prepared a second generation of ZAP-70 specific biosensors. Here we describe a novel biosensor named ROZA-XL, that displays a 3-4 times greater dynamic range than its predecessor and possesses a robust baseline FRET value when expressed in the Jurkat human T cell line. We demonstrate that the robust behavior of this biosensor allows for rapid analysis of TCR mediated of ZAP-70 kinase activity at a single cell level, as shown in a simple end point assay in which ROZA-XL expressing cells are allowed to interact with stimulatory anti-CD3epsilon coated coverslips. 
 Analysis of Phosphorylation-dependent Protein Interactions of Adhesion and Degranulation Promoting Adaptor Protein (ADAP) Reveals Novel Interaction Partners Required for Chemokine-directed T cell   Migration  . Stimulation of T cells leads to distinct changes of their adhesive and migratory properties. Signal propagation from activated receptors to integrins depends on scaffolding proteins such as the adhesion and degranulation promoting adaptor protein (ADAP)(1). Here we have comprehensively investigated the phosphotyrosine interactome of ADAP in T cells and define known and novel interaction partners of functional relevance. While most phosphosites reside in unstructured regions of the protein, thereby defining classical SH2 domain interaction sites for master regulators of T cell signaling such as SLP76, Fyn-kinase, and NCK, other binding events depend on structural context. Interaction proteomics using different ADAP constructs comprising most of the known phosphotyrosine motifs as well as the structured domains confirm that a distinct set of proteins is attracted by pY571 of ADAP, including the ζ-chain-associated protein kinase of 70 kDa (ZAP70). The interaction of ADAP and ZAP70 is inducible upon stimulation either of the T cell receptor (TCR) or by chemokine. NMR spectroscopy reveals that the N-terminal SH2 domains within a ZAP70-tandem-SH2 construct is the major site of interaction with phosphorylated ADAP-hSH3(N) and microscale thermophoresis (MST) indicates an intermediate binding affinity (Kd = 2.3 μm). Interestingly, although T cell receptor dependent events such as T cell/antigen presenting cell (APC) conjugate formation and adhesion are not affected by mutation of Y571  , migrati  on of T cells along a chemokine gradient is compromised. Thus, although most phospho-sites in ADAP are linked to T cell receptor related functions we have identified a unique phosphotyrosine that is solely required for chemokine induced T cell behavior. 
  GeneRIFs  
 A distinct set of proteins interaction partners required for chemokine-directed T cell   migration   is attracted by phosphotyrosine 571 of ADAP, including ZAP70. 
 In all, our study demonstrates that miR-631 decreases PCa cell   migration   and invasion by dampening ZAP70 expression. 
 ZAP-70 enhances the   migration   of malignant B-cells into the supportive microenvironment found in the bone marrow mainly by enhancing signaling and   migration   after CXCR4 stimulation. 
 T cells from ZAP-70-negative samples show significantly less   migration   towards (C-X-C motif) chemokine ligand (CXCL)12 compared to T cells from ZAP-70-positive samples. 
 Through its control of high-affinity LFA-1, the LFA-1/Lck/ZAP-70 complex is in position to initiate the rapid adhesion and   migration   necessary for T-lymphocyte responses when stimulated vasculature is encountered at sites of infection or injury. 
 Zap70 modulates integrin activation by interacting with talin, which contributes to directionality of T-cell   migration  , severing as a potential target for anti-inflammation therapy. 
 Signaling through ZAP-70 is required for CXCL12-mediated T-cell transendothelial   migration  . 
  [Ii]nvasive  PubMed  
 CDC25A, VAV1, TP73, BRCA1 and ZAP70 gene overexpression correlates with radiation response in colorectal cancer. Radiotherapy is increasingly used in adjuvant approaches for colorectal cancer (CRC) to reduce local recurrence and improve survival. However, the principal limitation is the large variability in response among different individuals due to tumor heterogeneity. In the present study, we compared gene expression profiles between radiosensitive and radioresistant colorectal cancer cell lines to identify radiation-related molecules that can be used to evaluate the effects of radiation. The CRC cell line SW620 was irradiated with a high-energy photo beam. Following radiation treatment, RNA was extracted from non-irradiated and irradiated cells, respectively, and gene expression analysis was performed by oligonucleotide microarray and the DAVID bioinformatics method. To further confirm the results, an additional 4 CRC cell lines, COLO205, T84, HCT116, SW480 and SW403 were purchased from ATCC. The radiosensitivities of each were determined by the survival fraction at 2 Gray (SF2) of the surviving cells using the ATPLite assay, and the gene expression profiles after irradiation among the radiosensitive and radioresistant cell lines were analyzed by membrane arrays. The relationships between gene expression and patient clinicopathological features were also analyzed using membrane arrays and RT-PCR. The results from oligonucleotide microarray analysis show that 1601 genes were up-regulated (gene expression ratio of post- to pre-radiation treatment>2). By bioinformatic database analysis, 30 up-regulated genes were identified as involved in DNA damage response pathways, immune response pathways and the complement and coagulation cascades pathway. Fifteen genes showed differential gene expression profiles between radiosensitive (HCT116 and SW620) and radioresistant CRC cell lines (SW403 and SW480). In 110 CRC tissues, we detected five genes CDC25A, VAV1, TP73, BRCA1 and ZAP70 from 15 overexpressed genes that significantly related to prognostic factors (tumor size, advanced stage,   invasive   depth, lymph node metastasis and differentiation). These findings suggest that CDC25A, VAV1, TP73, BRCA1 and ZAP70 may be novel markers for predicting the effectiveness of radiotherapy in CRC patients. 
 CDC25A, VAV1, TP73, BRCA1 and ZAP70 gene overexpression correlates with radiation response in colorectal cancer. Radiotherapy is increasingly used in adjuvant approaches for colorectal cancer (CRC) to reduce local recurrence and improve survival. However, the principal limitation is the large variability in response among different individuals due to tumor heterogeneity. In the present study, we compared gene expression profiles between radiosensitive and radioresistant colorectal cancer cell lines to identify radiation-related molecules that can be used to evaluate the effects of radiation. The CRC cell line SW620 was irradiated with a high-energy photo beam. Following radiation treatment, RNA was extracted from non-irradiated and irradiated cells, respectively, and gene expression analysis was performed by oligonucleotide microarray and the DAVID bioinformatics method. To further confirm the results, an additional 4 CRC cell lines, COLO205, T84, HCT116, SW480 and SW403 were purchased from ATCC. The radiosensitivities of each were determined by the survival fraction at 2 Gray (SF2) of the surviving cells using the ATPLite assay, and the gene expression profiles after irradiation among the radiosensitive and radioresistant cell lines were analyzed by membrane arrays. The relationships between gene expression and patient clinicopathological features were also analyzed using membrane arrays and RT-PCR. The results from oligonucleotide microarray analysis show that 1601 genes were up-regulated (gene expression ratio of post- to pre-radiation treatment>2). By bioinformatic database analysis, 30 up-regulated genes were identified as involved in DNA damage response pathways, immune response pathways and the complement and coagulation cascades pathway. Fifteen genes showed differential gene expression profiles between radiosensitive (HCT116 and SW620) and radioresistant CRC cell lines (SW403 and SW480). In 110 CRC tissues, we detected five genes CDC25A, VAV1, TP73, BRCA1 and ZAP70 from 15 overexpressed genes that significantly related to prognostic factors (tumor size, advanced stage,   invasive   depth, lymph node metastasis and differentiation). These findings suggest that CDC25A, VAV1, TP73, BRCA1 and ZAP70 may be novel markers for predicting the effectiveness of radiotherapy in CRC patients. 
 Syk tyrosine kinase expression during multistep mammary carcinogenesis. To analyze the expression of Syk tyrosine kinase, recently implicated as a tumor suppressor in mammary carcinogenesis, during the multi step development of human breast carcinoma.Syk expression was examined in   invasive   carcinomas of the breast and in corresponding premalignant epithelial lesions in 50 women, using immunohistochemical method and semi-quantitative scoring system (H-score). The results were correlated with the expression of Syk in the lymph node metastases.Syk was strongly and uniformly expressed in normal mammary epithelium (H score=2.36-/+0.75), and no significant reduction in Syk expression was observed in hyperplastic lesions (H=2.31-/+0.87), carcinoma in situ (H=1.90-/+0.93), or   invasive   carcinomas (H=1.83-/+0.88). Loss of Syk expression (defined as H< or =1.0) was seen in approximately 1/5 of   invasive   breast carcinomas, but the majority of metastatic carcinomas (15/21) still strongly expressed Syk, irrespective of Syk expression in primary tumor.The loss of Syk characterizes a subset of breast carcinomas but does not apparently contribute to the development of the metastatic potential of these carcinomas, limiting the potential diagnostic utility of immunohistochemical test in the prediction of malignant behavior. 
  [Mm]etastasis  PubMed  
 CDC25A, VAV1, TP73, BRCA1 and ZAP70 gene overexpression correlates with radiation response in colorectal cancer. Radiotherapy is increasingly used in adjuvant approaches for colorectal cancer (CRC) to reduce local recurrence and improve survival. However, the principal limitation is the large variability in response among different individuals due to tumor heterogeneity. In the present study, we compared gene expression profiles between radiosensitive and radioresistant colorectal cancer cell lines to identify radiation-related molecules that can be used to evaluate the effects of radiation. The CRC cell line SW620 was irradiated with a high-energy photo beam. Following radiation treatment, RNA was extracted from non-irradiated and irradiated cells, respectively, and gene expression analysis was performed by oligonucleotide microarray and the DAVID bioinformatics method. To further confirm the results, an additional 4 CRC cell lines, COLO205, T84, HCT116, SW480 and SW403 were purchased from ATCC. The radiosensitivities of each were determined by the survival fraction at 2 Gray (SF2) of the surviving cells using the ATPLite assay, and the gene expression profiles after irradiation among the radiosensitive and radioresistant cell lines were analyzed by membrane arrays. The relationships between gene expression and patient clinicopathological features were also analyzed using membrane arrays and RT-PCR. The results from oligonucleotide microarray analysis show that 1601 genes were up-regulated (gene expression ratio of post- to pre-radiation treatment>2). By bioinformatic database analysis, 30 up-regulated genes were identified as involved in DNA damage response pathways, immune response pathways and the complement and coagulation cascades pathway. Fifteen genes showed differential gene expression profiles between radiosensitive (HCT116 and SW620) and radioresistant CRC cell lines (SW403 and SW480). In 110 CRC tissues, we detected five genes CDC25A, VAV1, TP73, BRCA1 and ZAP70 from 15 overexpressed genes that significantly related to prognostic factors (tumor size, advanced stage, invasive depth, lymph node   metastasis   and differentiation). These findings suggest that CDC25A, VAV1, TP73, BRCA1 and ZAP70 may be novel markers for predicting the effectiveness of radiotherapy in CRC patients. 
 CDC25A, VAV1, TP73, BRCA1 and ZAP70 gene overexpression correlates with radiation response in colorectal cancer. Radiotherapy is increasingly used in adjuvant approaches for colorectal cancer (CRC) to reduce local recurrence and improve survival. However, the principal limitation is the large variability in response among different individuals due to tumor heterogeneity. In the present study, we compared gene expression profiles between radiosensitive and radioresistant colorectal cancer cell lines to identify radiation-related molecules that can be used to evaluate the effects of radiation. The CRC cell line SW620 was irradiated with a high-energy photo beam. Following radiation treatment, RNA was extracted from non-irradiated and irradiated cells, respectively, and gene expression analysis was performed by oligonucleotide microarray and the DAVID bioinformatics method. To further confirm the results, an additional 4 CRC cell lines, COLO205, T84, HCT116, SW480 and SW403 were purchased from ATCC. The radiosensitivities of each were determined by the survival fraction at 2 Gray (SF2) of the surviving cells using the ATPLite assay, and the gene expression profiles after irradiation among the radiosensitive and radioresistant cell lines were analyzed by membrane arrays. The relationships between gene expression and patient clinicopathological features were also analyzed using membrane arrays and RT-PCR. The results from oligonucleotide microarray analysis show that 1601 genes were up-regulated (gene expression ratio of post- to pre-radiation treatment>2). By bioinformatic database analysis, 30 up-regulated genes were identified as involved in DNA damage response pathways, immune response pathways and the complement and coagulation cascades pathway. Fifteen genes showed differential gene expression profiles between radiosensitive (HCT116 and SW620) and radioresistant CRC cell lines (SW403 and SW480). In 110 CRC tissues, we detected five genes CDC25A, VAV1, TP73, BRCA1 and ZAP70 from 15 overexpressed genes that significantly related to prognostic factors (tumor size, advanced stage, invasive depth, lymph node   metastasis   and differentiation). These findings suggest that CDC25A, VAV1, TP73, BRCA1 and ZAP70 may be novel markers for predicting the effectiveness of radiotherapy in CRC patients. 
   
  
 TNK1 
 
  NCBI Gene  
  GeneRIF  
  Pubmed  
 
 
  Search Term  Rank  Fields  
  [Mm]igration  6  Process(1); PubMed(1)  
 
 
 hide/show details for TNK1 
  [Mm]igration  Process  
 cell   migration   
  PubMed  
 Targeted proteomic analysis of 14-3-3 sigma, a p53 effector commonly silenced in cancer. To comprehensively identify proteins interacting with 14-3-3 sigma in vivo, tandem affinity purification and the multidimensional protein identification technology were combined to characterize 117 proteins associated with 14-3-3 sigma in human cells. The majority of identified proteins contained one or several phosphorylatable 14-3-3-binding sites indicating a potential direct interaction with 14-3-3 sigma. 25 proteins were not previously assigned to any function and were named SIP2-26 (for 14-3-3 sigma-interacting protein). Among the 92 interactors with known function were a number of proteins previously implicated in oncogenic signaling (APC, A-RAF, B-RAF, and c-RAF) and cell cycle regulation (AJUBA, c-TAK, PTOV-1, and WEE1). The largest functional classes comprised proteins involved in the regulation of cytoskeletal dynamics, polarity, adhesion, mitogenic signaling, and motility. Accordingly ectopic 14-3-3 sigma expression prevented cellular   migration   in a wounding assay and enhanced mitogen-activated protein kinase signaling. The functional diversity of the identified proteins indicates that induction of 14-3-3 sigma could allow p53 to affect numerous processes in addition to the previously characterized inhibitory effect on G2/M progression. The data suggest that the cancer-specific loss of 14-3-3 sigma expression by epigenetic silencing or p53 mutations contributes to cancer formation by multiple routes. 
   
  
 TNK2 
 
  NCBI Gene  
  GeneRIF  
  Pubmed  
 
 
  Search Term  Rank  Fields  
  [Ff]ilopodia  1  PubMed(2)  
  [Mm]igration  6  Process(1); PubMed(12); GeneRIFs(3)  
  [Ee]xtracellular [Mm]atrix  8  PubMed(1); GeneRIFs(1)  
  [Ii]nvasive  10  PubMed(2); GeneRIFs(1)  
  [Mm]etastasize  11  PubMed(1)  
  [Mm]etastasis  12  PubMed(8); GeneRIFs(2)  
  [Cc]ell [Ii]nvasion  13  PubMed(1)  
 
 
 hide/show details for TNK2 
  [Ff]ilopodia  PubMed  
 Cdc42Hs facilitates cytoskeletal reorganization and neurite outgrowth by localizing the 58-kD insulin receptor substrate to filamentous actin. Cdc42Hs is involved in cytoskeletal reorganization and is required for neurite outgrowth in N1E-115 cells. To investigate the molecular mechanism by which Cdc42Hs regulates these processes, a search for novel Cdc42Hs protein partners was undertaken by yeast two-hybrid assay. Here, we identify the 58-kD substrate of the insulin receptor tyrosine kinase (IRS-58) as a Cdc42Hs target. IRS-58 is a brain-enriched protein comprising at least four protein-protein interaction sites: a Cdc42Hs binding site, an Src homology (SH)3-binding site, an SH3 domain, and a tryptophan, tyrptophan (WW)-binding domain. Expression of IRS-58 in Swiss 3T3 cells leads to reorganization of the filamentous (F)-actin cytoskeleton, involving loss of stress fibers and formation of   filopodia   and clusters. In N1E-115 cells IRS-58 induces neurite outgrowth with high complexity. Expression of a deletion mutant of IRS-58, which lacks the SH3- and WW-binding domains, induced neurite extension without complexity in N1E-115 cells. In Swiss 3T3 cells and N1E-115 cells, IRS-58 colocalizes with F-actin in clusters and   filopodia  . An IRS-58(1267N) mutant unable to bind Cdc42Hs failed to localize with F-actin to induce neurite outgrowth or significant cytoskeletal reorganization. These results suggest that Cdc42Hs facilitates cytoskeletal reorganization and neurite outgrowth by localizing protein complexes via adaptor proteins such as IRS-58 to F-actin. 
 Tyrosine phosphorylation of ACK in response to temperature shift-down, hyperosmotic shock, and epidermal growth factor stimulation. The mammalian Cdc42 protein regulates various kinds of cellular responses, including formation of   filopodia  , polarization of T cells, and cell cycle progression. A non-receptor tyrosine kinase ACK, which specifically binds to the GTP-bound form of Cdc42, was isolated as a putative target of Cdc42. Here we show the induction of tyrosine phosphorylation of ACK in response to temperature shift-down to 25 degrees C, and hypertonic shock, as well as stimulation with epidermal growth factor (EGF) in human embryonic kidney (HEK) 293 cells. The increased tyrosine phosphorylation level upon temperature shift-down was sustained for at least 60 min, whereas reversion of the temperature to 37 degrees C caused rapid tyrosine dephosphorylation to the initial level. The responses to EGF and the high osmolarity were transient. Furthermore, we observed association of ACK with an adaptor protein Grb2, which may suggest the involvement of Grb2 in EGF receptor-mediated tyrosine phosphorylation of ACK. 
  [Mm]igration  Process  
 cell   migration   
  PubMed  
 Ack1 mediates Cdc42-dependent cell   migration   and signaling to p130Cas. We previously showed that activation of the small GTPase Cdc42 promotes breast cell   migration   on a collagen matrix. Here we further define the signaling pathways that drive this response and show that Cdc42-mediated   migration   relies on the adaptor molecule p130(Cas). Activated Cdc42 enhanced p130(Cas) phosphorylation and its binding to Crk. Cdc42-driven   migration   and p130(Cas) phosphorylation were dependent on the Cdc42 effector Ack1 (activated Cdc42-associated kinase). Ack1 formed a signaling complex that also included Cdc42, p130(Cas), and Crk, formation of which was regulated by collagen stimulation. The interaction between Ack1 and p130(Cas) occurred through their respective SH3 domains, while the substrate domain of p130(Cas) was the major site of Ack1-dependent phosphorylation. Signaling through this complex is functionally relevant, because treatment with either p130(Cas) or Ack1 siRNA blocked Cdc42-induced   migration  . These results suggest that Cdc42 exerts its effects on cell   migration   in part through its effector Ack1, which regulates p130(Cas) signaling. 
 Ack1 mediates Cdc42-dependent cell   migration   and signaling to p130Cas. We previously showed that activation of the small GTPase Cdc42 promotes breast cell   migration   on a collagen matrix. Here we further define the signaling pathways that drive this response and show that Cdc42-mediated   migration   relies on the adaptor molecule p130(Cas). Activated Cdc42 enhanced p130(Cas) phosphorylation and its binding to Crk. Cdc42-driven   migration   and p130(Cas) phosphorylation were dependent on the Cdc42 effector Ack1 (activated Cdc42-associated kinase). Ack1 formed a signaling complex that also included Cdc42, p130(Cas), and Crk, formation of which was regulated by collagen stimulation. The interaction between Ack1 and p130(Cas) occurred through their respective SH3 domains, while the substrate domain of p130(Cas) was the major site of Ack1-dependent phosphorylation. Signaling through this complex is functionally relevant, because treatment with either p130(Cas) or Ack1 siRNA blocked Cdc42-induced   migration  . These results suggest that Cdc42 exerts its effects on cell   migration   in part through its effector Ack1, which regulates p130(Cas) signaling. 
 TNK2 gene amplification is a novel predictor of a poor prognosis in patients with gastric cancer. We previously examined the amplification status of 10 kinase genes (PIK3CA, EPHB3, TNK2, PTK7, EGFR, MET, ERBB2, HCK, SRC, and AURKA) in gastric cancer (GC). This study aimed to determine the prognostic significance of these gene amplifications in GC.A survival analysis was performed for GC patients. Since TNK2 amplification was identified as a prognostic marker in the analysis, we also examined the functional effect of TNK2 overexpression on gastric cells.A Kaplan-Meier analysis showed that the prognosis of patients with GC exhibiting TNK2 or AURKA amplification was significantly poorer than the prognosis of patients with GC without TNK2 or AURKA amplification. A further multivariate analysis revealed that TNK2 amplification was an independent predictor of a poor survival outcome among patients with GC (hazard ratio, 3.668; 95% confidence interval, 1.513-7.968; P = 0.0056). TNK2-overexpressing GC cells showed an increase in cell   migration   and non-anchored cell growth. Finally, microarray and pathway analyses revealed the aberrant regulation of some cancer-related pathways in TNK2-overexpressing GC cells.These results suggested that TNK2 amplification is an independent predictor of a poor prognosis in patients with GC and leads to an increase in the malignant potential of GC cells. 
 ACK1 promotes hepatocellular carcinoma progression via downregulating WWOX and activating AKT signaling. Several studies have revealed that ACK1 is upregulated in various cancers and promotes tumor progression. However, the role and mechanism of ACK1 in hepatocellular carcinoma (HCC) remains unknown. In this study, the expression of ACK1 was assessed in several cell lines and 150 pairs of HCC and adjacent noncancerous liver tissues. The protein expression of p-ACK1 and WWOX were detected by immunohistochemistry to evaluate their correlation with ACK1. Flow cytometry, caspase 3/7 activity assay, BrdU cell proliferation assay, MTT assay and Transwell assay were used to detect apoptosis, proliferation, invasion and   migration   of HCC cells. The regulatory effect of ACK1 on WWOX, AKT, p-AKT, MMP2 and MMP9 in HCC cells was confirmed by immuno-blotting. We found that ACK1 was more highly expressed in HCC tissues than in non-HCC tissues, and over-expression of ACK1 was correlated with clinicopathological features of poor prognosis. Clinical analysis demonstrated that ACK1 is an independent prognostic marker for predicting overall survival and disease-free survival of HCC patients. Pearson's correlation coefficient analysis indicated that ACK1 was positively associated with p-ACK1 and was negatively associated with WWOX expression. In vitro studies showed that knockdown of ACK1 promoted HCC cell apoptosis and repressed HCC cells invasion,   migration   and proliferation. Furthermore, knockdown of ACK1 resulted in upregulation of WWOX and inactivation of AKT signaling. In this study, we also found that knockdown of ACK1 resulted in the downregulation of MMP2 and MMP9 in HCC. Our results indicate that ACK1 is an independent prognostic marker and promotes HCC progression via downregulating WWOX and activating AKT signaling. 
 Cancer-associated mutations activate the nonreceptor tyrosine kinase Ack1. Ack1 is a nonreceptor tyrosine kinase that participates in tumorigenesis, cell survival, and   migration  . Relatively little is known about the mechanisms that regulate Ack1 activity. Recently, four somatic missense mutations of Ack1 were identified in cancer tissue samples, but the effects on Ack1 activity, and function have not been described. These mutations occur in the N-terminal region, the C-lobe of the kinase domain, and the SH3 domain. Here, we show that the cancer-associated mutations increase Ack1 autophosphorylation in mammalian cells without affecting localization and increase Ack1 activity in immune complex kinase assays. The cancer-associated mutations potentiate the ability of Ack1 to promote proliferation and   migration  , suggesting that point mutation is a mechanism for Ack1 deregulation. We propose that the C-terminal Mig6 homology region (MHR) (residues 802-990) participates in inhibitory intramolecular interactions. The isolated kinase domain of Ack1 interacts directly with the MHR, and the cancer-associated E346K mutation prevents binding. Likewise, mutation of a key hydrophobic residue in the MHR (Phe(820)) prevents the MHR-kinase interaction, activates Ack1, and increases cell   migration  . Thus, the cancer-associated mutation E346K appears to destabilize an autoinhibited conformation of Ack1, leading to constitutively high Ack1 activity. 
 Involvement of Activated Cdc42 Kinase1 in Colitis and Colorectal Neoplasms. BACKGROUND Activated Cdc42 kinase1 (ACK1) is a non-receptor tyrosine kinase which is critical for cell survival, proliferation, and   migration  . Genomic amplification of ACK1 has been reported in multiple human cancers. We aimed to investigate ACK1 protein expression in colorectal mucosa with inflammation and neoplasm, and to evaluate its correlation with disease activity and severity. MATERIAL AND METHODS A total of 250 individuals who underwent total colonoscopy were collected randomly from January 2007 to May 2013 in Nanfang Hospital, Guangzhou, China. Colorectal mucosal biopsy specimens were obtained by endoscopy from 78 patients with ulcerative colitis (UC), 22 with Crohn's disease (CD), 20 with infectious colitis, 26 with non-IBD and noninfectious colitis, 16 with sporadic adenomas, 4 with dysplasia-associated lesions or masses, 10 with sporadic colorectal cancer (CRC), 4 with UC-related CRC, 10 with hyperplastic polyps, and 60 without colonic abnormalities. ACK1 protein levels were determined immunohistochemically. The correlations of ACK1 expression with disease activity and severity were also evaluated. RESULTS Significantly increased ACK1 expression was observed in epithelial cells of colorectal mucosa with inflammation and dysplasia compared to controls (P<0.05). ACK1 expression correlated with clinical activity in IBD (χ²=4.57, P=0.033 for UC; χ²=5.68, P=0.017 for CD), as well as grade of dysplasia in preneoplastic lesions (P<0.05). No significant differences in ACK1 expression were found between UC and CD, or between IBD and non-IBD conditions (P>0.05). CONCLUSIONS ACK1 protein is increased extensively in colitis and colorectal dysplasia. ACK1 overexpression may play a role in colorectal inflammation and neoplasms. 
 ACK1 promotes gastric cancer epithelial-mesenchymal transition and metastasis through AKT-POU2F1-ECD signalling. Amplification of the activated Cdc42-associated kinase 1 (ACK1) gene is frequent in gastric cancer (GC). However, little is known about the clinical roles and molecular mechanisms of ACK1 abnormalities in GC. Here, we found that the ACK1 protein level and ACK1 phosphorylation at Tyr 284 were frequently elevated in GC and associated with poor patient survival. Ectopic ACK1 expression in GC cells induced epithelial-mesenchymal transition (EMT) and promoted   migration   and invasion in vitro, and metastasis in vivo; the depletion of ACK1 induced the opposite effects. We utilized SILAC quantitative proteomics to discover that the level of the cell cycle-related protein ecdysoneless homologue (ECD) was markedly altered by ACK1. Overexpression of ECD promoted EMT,   migration  , and invasion in GC, similar to the effects of ACK1 overexpression. Silencing of ECD completely blocked the augmentation of ACK1 overexpression-induced EMT,   migration  , and invasion. Mechanistically, ACK1 phosphorylated AKT at Thr 308 and Ser 473 and activated the AKT pathway to up-regulate the transcription factor POU2F1, which directly bound to the promoter region of its novel target gene ECD and thus regulated ECD expression in GC cells. Furthermore, the phosphorylation levels of AKT at Thr 308 and Ser 473 and POU2F1 and ECD levels were positively associated with ACK1 levels in clinical GC specimens. Collectively, we have demonstrated that ACK1 promotes EMT,   migration  , and invasion by activating AKT-POU2F1-ECD signalling in GC cells. ACK1 may be employed as a new prognostic factor and therapeutic target for GC. 
 Bosutinib inhibits   migration   and invasion via ACK1 in KRAS mutant non-small cell lung cancer. The advent of effective targeted therapeutics has led to increasing emphasis on precise biomarkers for accurate patient stratification. Here, we describe the role of ACK1, a non-receptor tyrosine kinase in abrogating   migration   and invasion in KRAS mutant lung adenocarcinoma. Bosutinib, which inhibits ACK1 at 2.7 nM IC50, was found to inhibit cell   migration   and invasion but not viability in a panel of non-small cell lung cancer (NSCLC) cell lines. Knockdown of ACK1 abrogated bosutinib-induced inhibition of cell   migration   and invasion specifically in KRAS mutant cells. This finding was further confirmed in an in vivo zebrafish metastatic model. Tissue microarray data on 210 Singaporean lung adenocarcinomas indicate that cytoplasmic ACK1 was significantly over-expressed relative to paired adjacent non-tumor tissue. Interestingly, ACK1 expression in "normal" tissue adjacent to tumour, but not tumour, was independently associated with poor overall and relapse-free survival. In conclusion, inhibition of ACK1 with bosutinib attenuates   migration   and invasion in the context of KRAS mutant NSCLC and may fulfil a therapeutic niche through combinatorial treatment approaches. 
 Somatic mutation in the ACK1 ubiquitin association domain enhances oncogenic signaling through EGFR regulation in renal cancer derived cells. Activated Cdc42-associated Kinase, ACK1, is a non-receptor tyrosine kinase with numerous interacting partners, including Cdc42 and EGFR. Gene amplification and overexpression of ACK1 were found in many cancer types such as those of the lung and prostate. Previously, we identified both somatic- and germ line missense mutations in the ACK1 coding sequence, by surveying 261 cancer cell lines and 15 control tissues. Here, we verified and characterized the non-synonymous mutation, ACK-S985 N, located in the ubiquitin association domain of the protein. Both overexpression and silencing experiments in MCF7 and A498 cells, respectively, demonstrated a role of the ACK1 S985 N mutation in enhancing cell proliferation,   migration   and anchorage-independent growth as well as the epithelial-mesenchymal transition. Further, we showed that the ACK1 S985 N mutant is unable to bind ubiquitin, unlike the wild type kinase. This contributed to ACK1 protein stability and stabilized EGFR after EGF stimulation, thereby prolonging mitogenic signaling in cancer cells. In addition, the ACK1 S985 N-EGFR interaction is enhanced, but not the ubiquitination of the receptor. Intriguingly, silencing of ACK1 in A498 cells sensitized the renal carcinoma cells to gefitinib, against which they are otherwise resistant. The work demonstrates that other than gene amplification, a single somatic mutation in ACK1 can result in extended protein stability enabling the oncoprotein to exert its oncogenic function in tumor progression. It also provides a rationale to target ACK1 in combination with other chemotherapeutic drugs, such as EGFR inhibitors, to potentiate therapeutic action against resistant tumors. 
 TNK2 preserves epidermal growth factor receptor expression on the cell surface and enhances   migration   and invasion of human breast cancer cells. Amplification of the TNK2 gene in primary tumours correlates with poor prognosis. In accordance, TNK2 overexpression was shown to promote invasion of cancer cells--but the mechanism by which TNK2 mediates these effects is unresolved. TNK2 was suggested to regulate Cdc42-driven   migration   by activation of breast cancer antioestrogen resistance 1 (BCAR1); however, distinct from this effect is evidence for a role of TNK2 in the regulation of epidermal growth factor receptor (EGFR) endocytosis and degradation. In the present study we sought to investigate whether negative targeting of TNK2 by siRNA could be used to inhibit cancer cell invasion, to establish the contribution of its effect on the EGFR and to consequently attempt to resolve the issue of TNK2's mechanism of action.We used siRNA to knockdown expression of TNK2 and its proposed effector BCAR1 in order to analyse the effect of this knockdown on cancer cell behaviour in vitro. We examined morphological changes using phase-contrast microscopy and immunohistochemistry. Functional parameters examined included apoptosis, proliferation,   migration   and invasion. We also performed flow cytometry analysis to examine EGFR cell surface expression and carried out western blot to examine the total EGFR levels.We observed that targeting of TNK2 by siRNA in breast cancer cells resulted in distinct morphological changes characterised by a stellate appearance and an absence of protrusions at membrane edges. These changes were not recapitulated upon siRNA targeting of BCAR1. We thus hypothesised that a component of the effects induced by TNK2 may be independent of BCAR1. Consistent with the idea of an alternative mechanism for TNK2, we observed that TNK2 associates with activated EGFR in breast cancer cells in a TNK2-kinase-independent manner. Furthermore, we demonstrated that TNK2 functions to maintain EGFRs on the cell surface. We could demonstrate that the main functional effect of activating these surface EGFRs in breast cancer cells is stimulation of   migration  . In accordance, TNK2 silencing by siRNA led to a significant reduction in cell surface EGFR and to a concomitant decrease in the migratory and invasive capacity of breast cancer cells.Our data suggest that TNK2 can enhance   migration   and invasion of breast cancer cells via preservation of EGFR expression, notwithstanding its previously reported signalling via BCAR1, explaining its oncogenic behaviour in vitro and correlation with metastatic human breast cancer in vivo. 
 Cytoplasmic ACK1 interaction with multiple receptor tyrosine kinases is mediated by Grb2: an analysis of ACK1 effects on Axl signaling. ACK1 (activated Cdc42-associated kinase 1), a cytoplsmic tyrosine kinase, is implicated in metastatic behavior, cell spreading and   migration  , and epidermal growth factor receptor (EGFR) signaling. The function of ACK1 in the regulation of receptor tyrosine kinases requires a C-terminal region that demonstrates a significant homology to the EGFR binding domain of MIG6. In this study, we have identified additional receptor tyrosine kinases, including Axl, leukocyte tyrosine kinase, and anaplastic lymphoma kinase, that can bind to the ACK1/MIG6 homology region. Unlike the interaction between MIG6 and EGFR, our data suggest that these receptor tyrosine kinases require the adaptor protein Grb2 for efficient binding, which interacts with highly conserved proline-rich regions that are conserved between ACK1 and MIG6. We have focused on Axl and compared how ACK1/Axl differs from the ACK1/EGFR axis by investigating effects of knockdown of endogenous ACK1. Although EGFR activation promotes ACK1 turnover, Axl activation by GAS6 does not; interestingly, the reciprocal down-regulation of GAS6-stimulated Axl is blocked by removing ACK1. Thus, ACK1 functions in part to control Axl receptor levels. Silencing of ACK1 also leads to diminished ruffling and   migration   in DU145 and COS7 cells upon GAS6-Axl signaling. The ability of ACK1 to modulate Axl and perhaps anaplastic lymphoma kinase (altered in anaplastic large cell lymphomas) might explain why ACK1 can promote metastatic and transformed behavior in a number of cancers. 
 Activated cdc42-associated kinase is up-regulated in non-small-cell lung cancer and necessary for FGFR-mediated AKT activation. Activated cdc42-associated tyrosine kinase 1 (ACK1) has been reported to be implicated in non-small-cell lung cancer (NSCLC). However, the expression pattern and biological functions of ACK1 in the progression of NSCLC are not fully understood. In this study, it was found that the expression of ACK1 was significantly up-regulated in NSCLC samples compared to their adjacent normal tissues. Meanwhile, the expression of ACK1 was inversely correlated with the survival of NSCLC patients. Moreover, in the biological function studies, ACK1 was further validated to promote the growth,   migration  , and metastasis of NSCLC cells in vitro and in vivo. Mechanistically, ACK1 bind with FGFR1 and was essential for the phosphorylation of AKT induced by FGF. Our study demonstrated that ACK1 played an oncogenic role in the progression of NSCLC and ACK1 might be a promising target for the treatment of NSCLC. 
  GeneRIFs  
 ACK1 promotes EMT,   migration  , and invasion by activating AKT-POU2F1-ECD signalling in Gastric cancer cells. ACK1 may be employed as a new prognostic factor and therapeutic target for gastric cancer. 
 we describe the role of ACK1, a non-receptor tyrosine kinase in abrogating   migration   and invasion in KRAS mutant lung adenocarcinoma 
 TNK2 enhanced   migration   and invasion of breast cancer cells by preserving EGFR expression, inspite of its previously reported signalling via BCAR1, explaining its oncogenic behaviour in vitro and correlation with metastatic human breast cancer in vivo. 
  [Ee]xtracellular [Mm]atrix  PubMed  
 Metastatic properties and genomic amplification of the tyrosine kinase gene ACK1. Metastasis of primary tumors leads to a very poor prognosis for patients suffering from cancer. Although it is well established that not every tumor will eventually metastasize, it is less clear whether primary tumors acquire genetic alterations in a stochastic process at a late stage, which make them invasive, or whether genetic alterations acquired early in the process of tumor development drive primary tumor growth and determine whether this tumor is going to be metastatic. To address this issue, we tested genes identified in a large-scale comparative genomic hybridization analysis of primary tumor for their ability to confer metastatic properties on a cancer cell. We identified amplification of the ACK1 gene in primary tumors, which correlates with poor prognosis. We further show that overexpression of Ack1 in cancer cell lines can increase the invasive phenotype of these cells both in vitro and in vivo and leads to increased mortality in a mouse model of metastasis. Biochemical studies show that Ack1 is involved in   extracellular matrix  -induced integrin signaling, ultimately activating signaling processes like the activation of the small GTPase Rac. Taken together, this study supports a theory from Bernards and Weinberg [Bernards, R. & Weinberg, R. A. (2002) Nature 418, 823], which postulates that the tendency to metastasize is largely predetermined. 
  GeneRIFs  
 overexpression of Ack1 in cancer cell lines can increase the invasive phenotype of these cells; Ack1 is involved in   extracellular matrix  -induced integrin signaling, ultimately activating signaling processes like the activation of the small GTPase Rac 
  [Ii]nvasive  PubMed  
 TNK2 preserves epidermal growth factor receptor expression on the cell surface and enhances migration and invasion of human breast cancer cells. Amplification of the TNK2 gene in primary tumours correlates with poor prognosis. In accordance, TNK2 overexpression was shown to promote invasion of cancer cells--but the mechanism by which TNK2 mediates these effects is unresolved. TNK2 was suggested to regulate Cdc42-driven migration by activation of breast cancer antioestrogen resistance 1 (BCAR1); however, distinct from this effect is evidence for a role of TNK2 in the regulation of epidermal growth factor receptor (EGFR) endocytosis and degradation. In the present study we sought to investigate whether negative targeting of TNK2 by siRNA could be used to inhibit cancer cell invasion, to establish the contribution of its effect on the EGFR and to consequently attempt to resolve the issue of TNK2's mechanism of action.We used siRNA to knockdown expression of TNK2 and its proposed effector BCAR1 in order to analyse the effect of this knockdown on cancer cell behaviour in vitro. We examined morphological changes using phase-contrast microscopy and immunohistochemistry. Functional parameters examined included apoptosis, proliferation, migration and invasion. We also performed flow cytometry analysis to examine EGFR cell surface expression and carried out western blot to examine the total EGFR levels.We observed that targeting of TNK2 by siRNA in breast cancer cells resulted in distinct morphological changes characterised by a stellate appearance and an absence of protrusions at membrane edges. These changes were not recapitulated upon siRNA targeting of BCAR1. We thus hypothesised that a component of the effects induced by TNK2 may be independent of BCAR1. Consistent with the idea of an alternative mechanism for TNK2, we observed that TNK2 associates with activated EGFR in breast cancer cells in a TNK2-kinase-independent manner. Furthermore, we demonstrated that TNK2 functions to maintain EGFRs on the cell surface. We could demonstrate that the main functional effect of activating these surface EGFRs in breast cancer cells is stimulation of migration. In accordance, TNK2 silencing by siRNA led to a significant reduction in cell surface EGFR and to a concomitant decrease in the migratory and   invasive   capacity of breast cancer cells.Our data suggest that TNK2 can enhance migration and invasion of breast cancer cells via preservation of EGFR expression, notwithstanding its previously reported signalling via BCAR1, explaining its oncogenic behaviour in vitro and correlation with metastatic human breast cancer in vivo. 
 Metastatic properties and genomic amplification of the tyrosine kinase gene ACK1. Metastasis of primary tumors leads to a very poor prognosis for patients suffering from cancer. Although it is well established that not every tumor will eventually metastasize, it is less clear whether primary tumors acquire genetic alterations in a stochastic process at a late stage, which make them   invasive  , or whether genetic alterations acquired early in the process of tumor development drive primary tumor growth and determine whether this tumor is going to be metastatic. To address this issue, we tested genes identified in a large-scale comparative genomic hybridization analysis of primary tumor for their ability to confer metastatic properties on a cancer cell. We identified amplification of the ACK1 gene in primary tumors, which correlates with poor prognosis. We further show that overexpression of Ack1 in cancer cell lines can increase the   invasive   phenotype of these cells both in vitro and in vivo and leads to increased mortality in a mouse model of metastasis. Biochemical studies show that Ack1 is involved in extracellular matrix-induced integrin signaling, ultimately activating signaling processes like the activation of the small GTPase Rac. Taken together, this study supports a theory from Bernards and Weinberg [Bernards, R. & Weinberg, R. A. (2002) Nature 418, 823], which postulates that the tendency to metastasize is largely predetermined. 
  GeneRIFs  
 overexpression of Ack1 in cancer cell lines can increase the   invasive   phenotype of these cells; Ack1 is involved in extracellular matrix-induced integrin signaling, ultimately activating signaling processes like the activation of the small GTPase Rac 
  [Mm]etastasize  PubMed  
 Metastatic properties and genomic amplification of the tyrosine kinase gene ACK1. Metastasis of primary tumors leads to a very poor prognosis for patients suffering from cancer. Although it is well established that not every tumor will eventually   metastasize  , it is less clear whether primary tumors acquire genetic alterations in a stochastic process at a late stage, which make them invasive, or whether genetic alterations acquired early in the process of tumor development drive primary tumor growth and determine whether this tumor is going to be metastatic. To address this issue, we tested genes identified in a large-scale comparative genomic hybridization analysis of primary tumor for their ability to confer metastatic properties on a cancer cell. We identified amplification of the ACK1 gene in primary tumors, which correlates with poor prognosis. We further show that overexpression of Ack1 in cancer cell lines can increase the invasive phenotype of these cells both in vitro and in vivo and leads to increased mortality in a mouse model of metastasis. Biochemical studies show that Ack1 is involved in extracellular matrix-induced integrin signaling, ultimately activating signaling processes like the activation of the small GTPase Rac. Taken together, this study supports a theory from Bernards and Weinberg [Bernards, R. & Weinberg, R. A. (2002) Nature 418, 823], which postulates that the tendency to   metastasize   is largely predetermined. 
  [Mm]etastasis  PubMed  
 Ack1 overexpression promotes   metastasis   and indicates poor prognosis of hepatocellular carcinoma. Despite the substantial data supporting the oncogenic role of Ack1, the predictive value and biologic role of Ack1 in hepatocellular carcinoma (HCC)   metastasis   remains unknown. In this study, both correlations of Ack1 expression with prognosis of HCC, and the role of Ack1 in   metastasis   of HCC were investigated in vitro and in vivo. Our results showed that Ack1 was overexpressed in human HCC tissues and cell lines. High Ack1 expression was associated with HCC   metastasis   and determined as a significant and independent prognostic factor for HCC after liver resection. Ack1 promoted HCC invasion and   metastasis   in vitro and in vivo. Mechanistically, we confirmed that Ack1 enhanced invasion and   metastasis   of HCC via EMT by mediating AKT phosphorylation. In conclusion, our study shows Ack1 is a novel prognostic biomarker for HCC and promotes   metastasis   of HCC via EMT by activating AKT signaling. 
 Overexpression of activated Cdc42-associated kinase1 (Ack1) predicts tumor recurrence and poor survival in human hepatocellular carcinoma. Hepatocellular carcinoma (HCC) is one of the most common cancers in China. Recent research suggested that activated Cdc42-associated kinase 1 (Ack1) played an important role in facilitating tumorigenesis, tumor invasion and   metastasis  . However, the role of Ack1 in HCC is not clear. Herein, the expression level of Ack1 mRNA in 30 fresh HCC specimens (carcinoma, peri-carcinoma and distal-carcinoma tissues) was detected by reverse transcription-polymerase chain reaction (RT-PCR), while the expression of Ack1 protein in 18 fresh HCC specimens (carcinoma, peri-carcinoma and distal-carcinoma tissues) was analyzed by Western blotting. Immunohistochemical (IHC) staining was also employed to assess both the expression level and distribution of Ack1 protein in HCC tissues collected from 173 lesions, so as to study the correlations between Ack1 protein expression and other HCC-related clinicopathologic parameters. The results showed that both Ack1 mRNA and protein were significantly over-expressed in HCC tissues than that of either peri-carcinoma or distal-carcinoma tissues (P < 0.001, P = 0.012, respectively), while there was no significant difference between peri-carcinoma and distal-carcinoma tissues. Furthermore, the results of IHC indicated that the rates of Ack1 expressions in the patients with capsular invasion, hepatic vessel involvement and recurrence were higher than without above three conditions (P = 0.037, P = 0.036, P = 0.019, respectively), whereas the patients with overexpression of Ack1 protein had low survival rate (P = 0.007). Ack1 expression, tumor size and recurrence were independently related to survival (P = 0.014, P = 0.018, P < 0.001, respectively). Thus, the level of Ack1 is associated with tumor invasion potential, and the expression of Ack1 plays an important role as predictor of recurrence and poor outcome in HCC patients. 
 [Correlation study of the overexpression of activated Cdc42-associated kinase 1 and the stage and prognosis of esophageal squamous cell carcinoma]. To investigate the expression and relationship of activated Cdc42-associated kinase 1 (ACK1) and the clinical characteristics of esophageal squamous cell carcinoma (ESCC).The ACK1 expression in ESCC cell lines was detected by Western blot. Immunohistochemistry was performed to assay the expression level of ACK1 protein in tumor tissues and adjacent normal epithelium from 105 ESCC patients in tissue microarray and 45 patients in normal tissue slices. Semi-quantitative RT-PCR(reverse transcription-polymerase chain reaction)was performed to determine the expression level of ACK1 mRNA in 45 pairs of ESCC frozen tissues.The expression level of ACK1 protein was significantly up-regulated in 48.6% ESCC tissues as compared with the normal adjacent epithelium in tissue microarray. The overexpression of ACK1 was significantly correlated with the lymph node   metastasis   and TNM stage of ESCC patients. The results of normal tissue slices were consistent with those of tissue microarray. Furthermore the overexpression of ACK1 was associated with a poor survival of ESCC patients (P = 0.030). The elevated mRNA level of ACK1 in ESCC tissues was correlated with the lymph node   metastasis   and TNM stage of ESCC patients. And a significant correlation was observed between protein and mRNA level of ACK1 (P = 0.021).The up-regulated expressions of ACK1 protein and mRNA are correlated with the progression and prognosis of ESCC. 
 ACK1 promotes gastric cancer epithelial-mesenchymal transition and   metastasis   through AKT-POU2F1-ECD signalling. Amplification of the activated Cdc42-associated kinase 1 (ACK1) gene is frequent in gastric cancer (GC). However, little is known about the clinical roles and molecular mechanisms of ACK1 abnormalities in GC. Here, we found that the ACK1 protein level and ACK1 phosphorylation at Tyr 284 were frequently elevated in GC and associated with poor patient survival. Ectopic ACK1 expression in GC cells induced epithelial-mesenchymal transition (EMT) and promoted migration and invasion in vitro, and   metastasis   in vivo; the depletion of ACK1 induced the opposite effects. We utilized SILAC quantitative proteomics to discover that the level of the cell cycle-related protein ecdysoneless homologue (ECD) was markedly altered by ACK1. Overexpression of ECD promoted EMT, migration, and invasion in GC, similar to the effects of ACK1 overexpression. Silencing of ECD completely blocked the augmentation of ACK1 overexpression-induced EMT, migration, and invasion. Mechanistically, ACK1 phosphorylated AKT at Thr 308 and Ser 473 and activated the AKT pathway to up-regulate the transcription factor POU2F1, which directly bound to the promoter region of its novel target gene ECD and thus regulated ECD expression in GC cells. Furthermore, the phosphorylation levels of AKT at Thr 308 and Ser 473 and POU2F1 and ECD levels were positively associated with ACK1 levels in clinical GC specimens. Collectively, we have demonstrated that ACK1 promotes EMT, migration, and invasion by activating AKT-POU2F1-ECD signalling in GC cells. ACK1 may be employed as a new prognostic factor and therapeutic target for GC. 
 Metastatic properties and genomic amplification of the tyrosine kinase gene ACK1.   Metastasis   of primary tumors leads to a very poor prognosis for patients suffering from cancer. Although it is well established that not every tumor will eventually metastasize, it is less clear whether primary tumors acquire genetic alterations in a stochastic process at a late stage, which make them invasive, or whether genetic alterations acquired early in the process of tumor development drive primary tumor growth and determine whether this tumor is going to be metastatic. To address this issue, we tested genes identified in a large-scale comparative genomic hybridization analysis of primary tumor for their ability to confer metastatic properties on a cancer cell. We identified amplification of the ACK1 gene in primary tumors, which correlates with poor prognosis. We further show that overexpression of Ack1 in cancer cell lines can increase the invasive phenotype of these cells both in vitro and in vivo and leads to increased mortality in a mouse model of   metastasis  . Biochemical studies show that Ack1 is involved in extracellular matrix-induced integrin signaling, ultimately activating signaling processes like the activation of the small GTPase Rac. Taken together, this study supports a theory from Bernards and Weinberg [Bernards, R. & Weinberg, R. A. (2002) Nature 418, 823], which postulates that the tendency to metastasize is largely predetermined. 
 SIAH ubiquitin ligases target the nonreceptor tyrosine kinase ACK1 for ubiquitinylation and proteasomal degradation. Activated Cdc42-associated kinase 1 (ACK1) is a nonreceptor tyrosine kinase linked to cellular transformation. The aberrant regulation of ACK1 promotes tumor progression and   metastasis  . Therefore, ACK1 is regarded as a valid target in cancer therapy. Seven in absentia homolog (SIAH) ubiquitin ligases facilitate substrate ubiquitinylation that targets proteins to the proteasomal degradation pathway. Here we report that ACK1 and SIAH1 from Homo sapiens interact in a yeast two-hybrid screen. Protein-protein interaction studies and protein degradation analyses using deletion and point mutants of ACK1 verify that SIAH1 and the related SIAH2 interact with ACK1. The association between SIAHs and ACK1 depends on the integrity of a highly conserved SIAH-binding motif located in the far C-terminus of ACK1. Furthermore, we demonstrate that the interaction of ACK1 with SIAH1 and the induction of proteasomal degradation of ACK1 by SIAH1 are independent of ACK1's kinase activity. Chemical inhibitors blocking proteasomal activity corroborate that SIAH1 and SIAH2 destabilize the ACK1 protein by inducing its proteasomal turnover. This mechanism apparently differs from the lysosomal pathway targeting ACK1 after stimulation with the epidermal growth factor. Our data also show that ACK1, but not ACK1 mutants lacking the SIAH binding motif, has a discernable negative effect on SIAH levels. Additionally, knockdown approaches targeting the SIAH2 mRNA uncover specifically that the induction of SIAH2 expression, by hormonally-induced estrogen receptor (ER) activation, decreases the levels of ACK1 in luminal human breast cancer cells. Collectively, our data provide novel insights into the molecular mechanisms modulating ACK1 and they position SIAH ubiquitin ligases as negative regulators of ACK1 in transformed cells. 
 Activated cdc42-associated kinase is up-regulated in non-small-cell lung cancer and necessary for FGFR-mediated AKT activation. Activated cdc42-associated tyrosine kinase 1 (ACK1) has been reported to be implicated in non-small-cell lung cancer (NSCLC). However, the expression pattern and biological functions of ACK1 in the progression of NSCLC are not fully understood. In this study, it was found that the expression of ACK1 was significantly up-regulated in NSCLC samples compared to their adjacent normal tissues. Meanwhile, the expression of ACK1 was inversely correlated with the survival of NSCLC patients. Moreover, in the biological function studies, ACK1 was further validated to promote the growth, migration, and   metastasis   of NSCLC cells in vitro and in vivo. Mechanistically, ACK1 bind with FGFR1 and was essential for the phosphorylation of AKT induced by FGF. Our study demonstrated that ACK1 played an oncogenic role in the progression of NSCLC and ACK1 might be a promising target for the treatment of NSCLC. 
 Comparison of gene expression profiles between primary tumor and metastatic lesions in gastric cancer patients using laser microdissection and cDNA microarray. To study the differential gene expression profiles of target cells in primary gastric cancer and its metastatic lymph nodes using laser microdissection (LMD) in combination with cDNA microarray.Normal gastric tissue samples from 30 healthy individuals, 36 cancer tissue samples from primary gastric carcinoma and lymph node   metastasis   tissue samples from 58 patients during gastric cancer resection were obtained using LMD in combination with cDNA microarray independently. After P27-based amplification, aRNA from 36 of 58 patients (group 1) with lymph node   metastasis   and metastatic tissue specimens from the remaining 22 patients (group 2) were applied to cDNA microarray. Semiquantitative reverse transcriptase polymerase chain reaction (RT-PCR) and immunohistochemical assay verified the results of microarray in group 2 and further identified genes differentially expressed in the progression of gastric cancer.The expression of 10 genes was up-regulated while the expression of 15 genes was down-regulated in 22 gastric carcinoma samples compared with that of genes in the normal controls. The results were confirmed at the level of mRNA and protein, and suggested that four genes (OPCML, RNASE1, YES1 and ACK1) could play a key role in the tumorigenesis and   metastasis   of gastric cancer. The expression pattern of 3 genes (OPCML, RNASE1 and YES1) was similar to tumor suppressor genes. For example, the expression level of these genes was the highest in normal gastric epithelium, which was decreased in primary carcinoma, and further decreased in metastatic lymph nodes. On the contrary, the expression pattern of gene ACK1 was similar to that of oncogene. Four genes were further identified as differentially expressed genes in the majority of the cases in the progression of gastric cancer.LMD in combination with cDNA microarray provides a unique support foe the identification of early expression profiles of differential genes and the expression pattern of 3 genes (OPCML, RNASE1 and YES1) associated with the progression of gastric cancer. Further study is needed to reveal the molecular mechanism of lymph node   metastasis   in patients with gastric cancer. 
  GeneRIFs  
 Ack1 overexpression promotes   metastasis   in hepatocellular carcinoma. 
 The results were confirmed at the level of mRNA and protein, and suggested that four genes (OPCML, RNASE1, YES1 and ACK1) could play a key role in the tumorigenesis and   metastasis   of gastric cancer. 
  [Cc]ell [Ii]nvasion  PubMed  
 TNK2 preserves epidermal growth factor receptor expression on the cell surface and enhances migration and invasion of human breast cancer cells. Amplification of the TNK2 gene in primary tumours correlates with poor prognosis. In accordance, TNK2 overexpression was shown to promote invasion of cancer cells--but the mechanism by which TNK2 mediates these effects is unresolved. TNK2 was suggested to regulate Cdc42-driven migration by activation of breast cancer antioestrogen resistance 1 (BCAR1); however, distinct from this effect is evidence for a role of TNK2 in the regulation of epidermal growth factor receptor (EGFR) endocytosis and degradation. In the present study we sought to investigate whether negative targeting of TNK2 by siRNA could be used to inhibit cancer   cell invasion  , to establish the contribution of its effect on the EGFR and to consequently attempt to resolve the issue of TNK2's mechanism of action.We used siRNA to knockdown expression of TNK2 and its proposed effector BCAR1 in order to analyse the effect of this knockdown on cancer cell behaviour in vitro. We examined morphological changes using phase-contrast microscopy and immunohistochemistry. Functional parameters examined included apoptosis, proliferation, migration and invasion. We also performed flow cytometry analysis to examine EGFR cell surface expression and carried out western blot to examine the total EGFR levels.We observed that targeting of TNK2 by siRNA in breast cancer cells resulted in distinct morphological changes characterised by a stellate appearance and an absence of protrusions at membrane edges. These changes were not recapitulated upon siRNA targeting of BCAR1. We thus hypothesised that a component of the effects induced by TNK2 may be independent of BCAR1. Consistent with the idea of an alternative mechanism for TNK2, we observed that TNK2 associates with activated EGFR in breast cancer cells in a TNK2-kinase-independent manner. Furthermore, we demonstrated that TNK2 functions to maintain EGFRs on the cell surface. We could demonstrate that the main functional effect of activating these surface EGFRs in breast cancer cells is stimulation of migration. In accordance, TNK2 silencing by siRNA led to a significant reduction in cell surface EGFR and to a concomitant decrease in the migratory and invasive capacity of breast cancer cells.Our data suggest that TNK2 can enhance migration and invasion of breast cancer cells via preservation of EGFR expression, notwithstanding its previously reported signalling via BCAR1, explaining its oncogenic behaviour in vitro and correlation with metastatic human breast cancer in vivo. 
   
  
 XRCC5 
 
  NCBI Gene  
  GeneRIF  
  Pubmed  
 
 
  Search Term  Rank  Fields  
  [Mm]igration  6  PubMed(2); GeneRIFs(1)  
  [Ii]nvasive  10  PubMed(3)  
  [Mm]etastasize  11  PubMed(1)  
  [Mm]etastasis  12  PubMed(4); GeneRIFs(2)  
  [Cc]ell [Ii]nvasion  13  PubMed(1)  
 
 
 hide/show details for XRCC5 
  [Mm]igration  PubMed  
 Transport of the leaderless protein Ku on the cell surface of activated monocytes regulates their migratory abilities. Recent evidence shows that the DNA repair protein Ku is expressed on the surface of a subset of cells, where it contributes to adhesion and invasion processes, and also to viral or bacterial entry into target cells. Here, we show that Ku was expressed on the cell surface during activation of human monocytes and that its expression was independent of the conventional endoplasmic reticulum (ER)/Golgi secretory pathway. Ku inhibition, by blocking antibodies, decreases the   migration   of monocytes on fibronectin and laminin. On activation, nuclear Ku seems to move to the periphery of the cell and it shows a punctuate staining in the cytoplasm. The cytoplasmic distribution of Ku was shown to be unaltered by brefeldin A. Protease protection experiments show that Ku is contained within vesicles in activated monocytes. These data support a new role for Ku in the   migration   of monocytes into tissues, which depends on a tightly regulated pathway of intracellular redistribution. 
 Effect of Ku proteins on IRES-mediated translation. Ku is an abundant nuclear heterodimeric protein composed of 70 and 86 kDa subunits. As an activator of the catalytic subunit of DNA-PK (DNA-dependent protein kinase), Ku plays an important role in DNA repair and recombination. Ku is also involved in actions independent of DNA-PK, such as transcription regulation and telomere maintenance. Although Ku is localized in the cytoplasm under specific cellular conditions, no functions for Ku outside of the nucleus have as yet been reported. In addition to DNA binding, Ku binds specific RNA sequences with high affinity. However, no specific cellular mRNA targets for Ku have been identified.In a yeast three-hybrid system, Ku70 bound to an RNA bait that contained an IRES (internal ribosomal entry site) element. A single band with   migration   properties similar to those of Ku70 was immunoprecipitated with anti-Ku antibody, using UV cross-linked complexes formed by HeLa cell nuclear extracts and an IRES-containing RNA probe. IRES activity was reduced in Ku80(-/-) cells. Overexpression of Ku proteins stimulated IRES-dependent translation.The present study suggests that Ku binds IRES elements within RNA molecules, and that Ku plays a role in the modulation of IRES-mediated mRNA translation. 
  GeneRIFs  
 These data support a new role for Ku in the   migration   of monocytes into tissues, which depends on a tightly regulated pathway of intracellular redistribution. 
  [Ii]nvasive  PubMed  
 Genetic susceptibility to distinct bladder cancer subphenotypes. Clinical, pathologic, and molecular evidence indicate that bladder cancer is heterogeneous with pathologic/molecular features that define distinct subphenotypes with different prognoses. It is conceivable that specific patterns of genetic susceptibility are associated with particular subphenotypes.To examine evidence for the contribution of germline genetic variation to bladder cancer heterogeneity.The Spanish Bladder Cancer/EPICURO Study is a case-control study based in 18 hospitals located in five areas in Spain. Cases were patients with a newly diagnosed, histologically confirmed, urothelial cell carcinoma of the bladder from 1998 to 2001. Case diagnoses were reviewed and uniformly classified by pathologists following the World Health Organisation/International Society of Urological Pathology 1999 criteria. Controls were hospital-matched patients (n=1149).A total of 1526 candidate variants in 423 candidate genes were analysed. Three distinct subphenotypes were defined according to stage and grade: low-grade nonmuscle   invasive   (n=586), high-grade nonmuscle   invasive   (n=219), and muscle   invasive   (n=246). The association between each variant and subphenotype was assessed by polytomous risk models adjusting for potential confounders. Heterogeneity in genetic susceptibility among subphenotypes was also tested.Two established bladder cancer susceptibility genotypes, NAT2 slow-acetylation and GSTM1-null, exhibited similar associations among the subphenotypes, as did VEGF-rs25648, which was previously identified in our study. Other variants conferred risks for specific tumour subphenotypes such as PMS2-rs6463524 and CD4-rs3213427 (respective heterogeneity p values of 0.006 and 0.004), which were associated with muscle-  invasive   tumours (per-allele odds ratios [95% confidence interval] of 0.56 [0.41-0.77] and 0.71 [0.57-0.88], respectively) but not with non-muscle-  invasive   tumours. Heterogeneity p values were not robust in multiple testing according to their false-discovery rate.These exploratory analyses suggest that genetic susceptibility loci might be related to the molecular/pathologic diversity of bladder cancer. Validation through large-scale replication studies and the study of additional genes and single nucleotide polymorphisms are required. 
 Genetic susceptibility to distinct bladder cancer subphenotypes. Clinical, pathologic, and molecular evidence indicate that bladder cancer is heterogeneous with pathologic/molecular features that define distinct subphenotypes with different prognoses. It is conceivable that specific patterns of genetic susceptibility are associated with particular subphenotypes.To examine evidence for the contribution of germline genetic variation to bladder cancer heterogeneity.The Spanish Bladder Cancer/EPICURO Study is a case-control study based in 18 hospitals located in five areas in Spain. Cases were patients with a newly diagnosed, histologically confirmed, urothelial cell carcinoma of the bladder from 1998 to 2001. Case diagnoses were reviewed and uniformly classified by pathologists following the World Health Organisation/International Society of Urological Pathology 1999 criteria. Controls were hospital-matched patients (n=1149).A total of 1526 candidate variants in 423 candidate genes were analysed. Three distinct subphenotypes were defined according to stage and grade: low-grade nonmuscle   invasive   (n=586), high-grade nonmuscle   invasive   (n=219), and muscle   invasive   (n=246). The association between each variant and subphenotype was assessed by polytomous risk models adjusting for potential confounders. Heterogeneity in genetic susceptibility among subphenotypes was also tested.Two established bladder cancer susceptibility genotypes, NAT2 slow-acetylation and GSTM1-null, exhibited similar associations among the subphenotypes, as did VEGF-rs25648, which was previously identified in our study. Other variants conferred risks for specific tumour subphenotypes such as PMS2-rs6463524 and CD4-rs3213427 (respective heterogeneity p values of 0.006 and 0.004), which were associated with muscle-  invasive   tumours (per-allele odds ratios [95% confidence interval] of 0.56 [0.41-0.77] and 0.71 [0.57-0.88], respectively) but not with non-muscle-  invasive   tumours. Heterogeneity p values were not robust in multiple testing according to their false-discovery rate.These exploratory analyses suggest that genetic susceptibility loci might be related to the molecular/pathologic diversity of bladder cancer. Validation through large-scale replication studies and the study of additional genes and single nucleotide polymorphisms are required. 
 DNA-PK, the DNA-activated protein kinase, is differentially expressed in normal and malignant human tissues. DNA-PK is a nuclear, serine/threonine protein kinase required for repairing DNA double-strand breaks and for V(D)J recombination. To determine the distribution of DNA-PK in human tissues, we assayed paraffin-embedded sections of normal and cancerous tissues for DNA-PKcs and Ku80 by immunohistochemistry. We also assayed for Brca2, a human tumor suppressor gene that is implicated in the repair of DNA strand-breaks. Brca2 was strongly expressed in epithelial cells of the breast, endometrium, and thymus, in tingible body macrophages of follicular germinal centers of lymphoid tissue, and in reticuloendothelial cells in the spleen. DNA-PKcs and Ku80 expression was usually parallel, but both were expressed in a highly cell- and tissue-specific manner. The highest levels were observed in spermatogenic cells (but not in spermatozoa), and in neurons and glial cells of the central and autonomic nervous system. Neither protein was consistently expressed in liver nor in resting mammary epithelium, but lactating breast epithelium was strongly positive for DNA-PKcs and Ku80. In contrast to established human cell cultures, expression between cells in the same tissue was highly selective in the epidermis, exocrine pancreas, renal glomeruli, the red pulp of the spleen, and within cellular compartments of tonsils, lymph nodes, and thymus. Most cancerous tissues were consistently positive for DNA-PKcs and Ku80, except   invasive   carcinoma of the breast. DNA-PKcs, Ku80, and Ku70 mRNAs were expressed in all normal tissues with relatively little variation in levels. Our results suggest that the apparent absence of DNA-PKcs and Ku80 from some cells or tissues is a consequence of post-transcriptional mechanisms that regulate protein levels. 
  [Mm]etastasize  PubMed  
 DNA repair and replication proteins as prognostic markers in melanoma.   Elevated expression of DNA repair and replication genes has been reported in thick, non-fixed primary melanomas that subsequently went on t  o metastasi  ze, when compared to non-recurrent primary tumours. This increased expression could contribute to the extreme resistance shown by melanoma to DNA-damaging chemotherapeutics. We have investigated the hypothesis that levels of key DNA repair and replication proteins are prognostic biomarkers in melanoma.  We used a tissue microarray containing samples from all stages of melanomagenesis to investigate the hypothesis that levels of key DNA repair and replication proteins are prognostic biomarkers in a larger, more representative and readily available set of fixed primary melanomas. High expression of topoisomerase IIα (TOP2A), that relieves torsional stress during DNA replication, and XRCC5 (Ku80), required for DNA double-strand break repair, were associated with significantly worse survival.  Two (XRCC5 and TOP2A) of seven DNA repair and replication proteins studied were prognostic for melanoma. 
  [Mm]etastasis  PubMed  
 Ku86 is important for TrkA overexpression-induced breast cancer cell invasion. We have recently shown that breast tumors express high levels of TrkA compared with normal breast tissues, with TrkA overexpression enhancing breast cancer cell invasion in vitro and   metastasis   in animal models. In this study, we tried to identify molecules involved in TrkA overexpression-mediated biological effects in breast cancer cells.We used a proteomic-based approach to identify proteins involved in TrkA overexpression-stimulated invasion of MDA-MB-231 breast cancer cells. Proteins from control and TrkA overexpressing cells were separated using a cup-loading two-dimensional electrophoresis system before MALDI and LC-MS/MS mass spectrometry analysis.Among several putative regulated proteins, Ku86 was found increased in TrkA overexpressing cells. Moreover, Ku86 was co-immunoprecipitated with TrkA, suggesting the interaction of these two proteins in TrkA overexpressing cells. Interestingly, inhibition with small-interfering RNA and neutralizing antibodies showed that Ku86 was required for TrkA-stimulated cell invasion.These data allowed the identification of Ku86 as a new player involved in   metastasis   in breast cancer cells. Our findings suggest that TrkA and its down stream signaling pathways should be regarded as potential new targets for the development of future breast cancer therapy. 
 Prognostic significance of Ku80 in pT2N0M0 esophageal squamous cell carcinoma after Ivor-Lewis esophagectomy. Recent studies have shown that Ku80, a DNA repair protein, was involved in progression of malignant tumors. This study aimed to clarify the clinicopathological significance and prognostic value of Ku80 in pT2N0M0 esophageal squamous cell carcinoma (ESCC). We enrolled 217 patients with pT2N0M0 midthoracic ESCC who had undergone Ivor-Lewis esophagectomy. The expression profile of Ku80 was examined by immunohistochemistry. The results were correlated with the clinicopathological variables, overall survival (OS) and disease-free survival (DFS), in pT2N0M0 ESCC patients. The expression of Ku80 were higher in ESCC tissues than the corresponding health esophageal mucosa (P < 0.001). Clinically, the Ku80 expression levels were significantly related to tumor size (P = 0.018), differentiation degree (P = 0.010), and   tumor-nod  e-metastasis (TNM) stage (P = 0.001). Subsequent multivariate analysis demonstrated that tumor size, differentiation degree, TNM stage, and Ku80 expression were independent prognostic factors for the OS and the DFS of pT2N0M0 ESCC patients. Our data indicated that Ku80 expression level associates with key clinicopathological features and is an independent predictor of the OS and the DFS in pT2N0M0 ESCC patients. 
 Influence of Ku86 and XRCC4 expression in uterine cervical cancer on the response to preoperative radiotherapy. DNA double-strand breaks (DSB) are severe damages induced by ionizing radiation. Non-homologous end joining (NHEJ) is a major mechanism for repairing DSB. Immunohistochemical analysis of proteins involved in NHEJ, such as Ku86 and XRCC4 (X-ray repair cross-complementing protein 4) may be useful for predicting tumor radiosensitivity. We examined the relationship between expression of DSB-related proteins in biopsy specimens of uterine cervical cancer and the pathological effect of 40 Gy of preoperative radiotherapy. 119 patients with uterine cervical cancer were treated between 2000 and 2011. Pathological effects of preoperative radiotherapy were classified by examining hysterectomy specimens. Patients with complete response (pCR) had a significantly better overall 5-year survival rate than those without pCR (96.3 vs. 76.9 %, P = 0.02). The pCR rate was significantly higher in patients with low Ku86 and XRCC4 expression than in other patients (47.4 vs. 21.3 %, P = 0.04). Logistic regression analysis also demonstrated that low Ku86 and XRCC4 expression was a significant predictor of pCR (P = 0.03). Patients with high Ku86 and XRCC4 expression had a significantly lowe  r 5-year m  etastasis-free rate than others (79.3 vs. 93.5 %, P = 0.02). Proteins involved with NHEJ might have an influence on results of radiotherapy for uterine cervical cancer. 
 Immunohistochemical analysis of Ku70/86 expression of breast cancer tissues. DNA-dependent protein kinase (DNA-PK) has an important role in DNA double-strand break repair. We previously demonstrated the association of DNA-PK activity in peripheral blood lymphocytes (PBL) with incidence of chromosomal aberrations and risk of cancer. In this study, we examined the expression of Ku70 and Ku86 in breast cancer tissue and normal breast tissue with immunohistochemistry. We also measured the DNA-PK activity in PBL of the same patient. One hundred and ten breast cancer patients were included in this study. The expression of Ku70, and Ku86 in normal mammary epithelial cells and breast cancer cells obtained from surgical specimens was immunohistochemically examined. DNA-PK activity of PBL was measured by DNA-pull-down assay. The expression of Ku70 and that of Ku86 tended to parallel each other in normal and cancer tissues. There was also a relationship in the expression of Ku70 and Ku86 between cancer tissues and normal tissues in the same samples. Lower expression of Ku70 or Ku86 tended to be associated with higher malignant nuclear grade of cancer cells and higher frequency of axillary lymph node   metastasis  . The staining score of Ku70 or Ku86 of normal mammary epithelial cells or breast cancer cells had no significant relationship with DNA-PK activity of PBL. In conclusion, breast cancer cells inherited the characteristics of expression of Ku proteins from original mammary epithelial cells. The staining score of Ku70 or Ku86 of normal or cancer cells had no significant relationship with DNA-PK activity of PBL. This may be due to limitations in the assay sensitivity of immunohistochemistry. 
  GeneRIFs  
 uterine cervical cancer patients with high Ku86 and XRCC4 expression had a significantly lower 5-year   metastasis  -free rate than others. 
 data allowed the identification of Ku86 as a new player involved in   metastasis   in breast cancer cells. 
  [Cc]ell [Ii]nvasion  PubMed  
 Ku86 is important for TrkA overexpression-induced breast cancer   cell invasion  . We have recently shown that breast tumors express high levels of TrkA compared with normal breast tissues, with TrkA overexpression enhancing breast cancer   cell invasion   in vitro and metastasis in animal models. In this study, we tried to identify molecules involved in TrkA overexpression-mediated biological effects in breast cancer cells.We used a proteomic-based approach to identify proteins involved in TrkA overexpression-stimulated invasion of MDA-MB-231 breast cancer cells. Proteins from control and TrkA overexpressing cells were separated using a cup-loading two-dimensional electrophoresis system before MALDI and LC-MS/MS mass spectrometry analysis.Among several putative regulated proteins, Ku86 was found increased in TrkA overexpressing cells. Moreover, Ku86 was co-immunoprecipitated with TrkA, suggesting the interaction of these two proteins in TrkA overexpressing cells. Interestingly, inhibition with small-interfering RNA and neutralizing antibodies showed that Ku86 was required for TrkA-stimulated   cell invasion  .These data allowed the identification of Ku86 as a new player involved in metastasis in breast cancer cells. Our findings suggest that TrkA and its down stream signaling pathways should be regarded as potential new targets for the development of future breast cancer therapy. 
   
  
 MYPN 
 
  NCBI Gene  
  GeneRIF  
  Pubmed  
 
 
  Search Term  Rank  Fields  
  [Mm]igration  6  Process(1)  
  [Ii]nvasive  10  PubMed(1)  
 
 
 hide/show details for MYPN 
  [Mm]igration  Process  
 regulation of cell   migration   
  [Ii]nvasive  PubMed  
 The role of palladin in actin organization and cell motility. Palladin is a widely expressed protein found in stress fibers, focal adhesions, growth cones, Z-discs, and other actin-based subcellular structures. It belongs to a small gene family that includes the Z-disc proteins myopalladin and myotilin, all of which share similar Ig-like domains. Recent advances have shown that palladin shares with myotilin the ability to bind directly to F-actin, and to crosslink actin filaments into bundles, in vitro. Studies in a variety of cultured cells suggest that the actin-organizing activity of palladin plays a central role in promoting cell motility. Correlative evidence also supports this hypothesis, as palladin levels are typically up-regulated in cells that are actively migrating: in developing vertebrate embryos, in cells along a wound edge, and in metastatic cancer cells. Recently, a mutation in the human palladin gene was implicated in an unusually penetrant form of inherited pancreatic cancer, which has stimulated new ideas about the role of palladin in   invasive   cancer. 
   
  
 NEXN 
 
  NCBI Gene  
  GeneRIF  
  Pubmed  
 
 
  Search Term  Rank  Fields  
  [Mm]igration  6  Process(1); PubMed(2); Summary(1)  
  [Ii]nvasive  10  PubMed(1)  
 
 
 hide/show details for NEXN 
  [Mm]igration  Process  
 regulation of cell   migration   
  PubMed  
 Analysis of the myosin-II-responsive focal adhesion proteome reveals a role for β-Pix in negative regulation of focal adhesion maturation. Focal adhesions undergo myosin-II-mediated maturation wherein they grow and change composition to modulate integrin signalling for cell   migratio  n, growth and differentiation. To determine how focal adhesion composition is affected by myosin II activity, we performed proteomic analysis of isolated focal adhesions and compared protein abundance in focal adhesions from cells with and without myosin II inhibition. We identified 905 focal adhesion proteins, 459 of which changed in abundance with myosin II inhibition, defining the myosin-II-responsive focal adhesion proteome. The abundance of 73% of the proteins in the myosin-II-responsive focal adhesion proteome was enhanced by contractility, including proteins involved in Rho-mediated focal adhesion maturation and endocytosis- and calpain-dependent focal adhesion disassembly. During myosin II inhibition, 27% of proteins in the myosin-II-responsive focal adhesion proteome, including proteins involved in Rac-mediated lamellipodial protrusion, were enriched in focal adhesions, establishing that focal adhesion protein recruitment is also negatively regulated by contractility. We focused on the Rac guanine nucleotide exchange factor β-Pix, documenting its role in the negative regulation of focal adhesion maturation and the promotion of lamellipodial protrusion and focal adhesion turnover to drive cel  l migrati  on. 
 NELIN, a new F-actin associated protein, stimulates HeLa cell   migration   and adhesion. A new gene (GenBank Accession No. AF114264) was cloned from umbilical vein wall tissue by using RT-PCR. The gene shares high similarity to the gene encoding F-actin binding protein nexilin, so named as NELIN. A clone of 2737bp contains open reading frame of 1344bp extending from 412 to 1755. NELIN was expressed primarily in the heart and skeletal muscle among eight tested normal tissues. Immunofluorescence and immunoprecipitation demonstrated that NELIN product was associated with F-actin. Stable transfection of NELIN into HeLa cells increased the cell   migration   by 2.17-fold and the adhesion by 1.67-fold, respectively, compared to cells with the empty vector (P<0.05). The results support that NELIN product is an F-actin associated protein and mediates cell motility. 
  Summary  
 This gene encodes a filamentous actin-binding protein that may function in cell adhesion and   migration  . Mutations in this gene have been associated with dilated cardiomyopathy, also known as CMD1CC. Alternatively spliced transcript variants have been described.[provided by RefSeq, Feb 2010] 
  [Ii]nvasive  PubMed  
 Nexilin is a dynamic component of Listeria monocytogenes and enteropathogenic Escherichia coli actin-rich structures. The bacterial pathogens Listeria monocytogenes and enteropathogenic Escherichia coli (EPEC) generate motile actin-rich structures (comet tails and pedestals) as part of their infectious processes. Nexilin, an actin-associated protein and a component of focal adhesions, has been suggested to be involved in actin-based motility. To determine whether nexilin is commandeered during L. monocytogenes and EPEC infections, we infected cultured cells and found that nexilin is crucial for L. monocytogenes invasion as levels of internalized bacteria were significantly decreased in nexilin-targeted siRNA-treated cells. In addition, nexilin is a component of the machinery that drives the formation of L. monocytogenes comet tails and EPEC pedestals. Nexilin colocalizes with stationary bacteria and accumulates at the distal portion of comet tails and pedestals of motile bacteria. We also show that nexilin is crucial for efficient comet tail formation as cells pre-treated with nexilin siRNA generate malformed comet tails, whereas nexilin is dispensable during EPEC pedestal generation. These findings demonstrate that nexilin is required for efficient infection with   invasive   and adherent bacteria and is key to the actin-rich structures these microbes generate. 
   
  
 OSMR 
 
  NCBI Gene  
  GeneRIF  
  Pubmed  
 
 
  Search Term  Rank  Fields  
  [Mm]igration  6  PubMed(3)  
  [Ee]xtracellular [Mm]atrix  8  PubMed(1)  
  [Ii]nvasive  10  PubMed(5); GeneRIFs(1)  
 
 
 hide/show details for OSMR 
  [Mm]igration  PubMed  
 Oncostatin M receptor-beta signaling limits monocytic cell recruitment in acute inflammation. Although the IL-6-related cytokine oncostatin M (OSM) affects processes associated with disease progression, the specific function of OSM in the face of an inflammatory challenge remains unclear. In this report, a peritoneal model of acute inflammation was used to define the influence of OSM on chemokine-mediated leukocyte recruitment. When compared with wild-type and IL-6-deficient mice, peritoneal inflammation in oncostatin M receptor-beta-deficient (OSMR-KO) mice resulted in enhanced monocytic cell trafficking. In contrast to IL-6-deficient mice, OSMR-KO mice displayed no difference in neutrophil and lymphocyte   migration  . Subsequent in vitro studies using human peritoneal mesothelial cells and an in vivo appraisal of inflammatory chemokine expression after peritoneal inflammation identified OSM as a prominent regulator of CCL5 expression. Specifically, OSM inhibited IL-1beta-mediated NF-kappaB activity and CCL5 expression in human mesothelial cells. This was substantiated in vivo where peritoneal inflammation in OSMR-KO mice resulted in a temporal increase in both CCL5 secretion and NF-kappaB activation. These findings suggest that IL-6 and OSM individually affect the profile of leukocyte trafficking, and they point to a hitherto unidentified interplay between OSM signaling and the inflammatory activation of NF-kappaB. 
 Tissue transglutaminase mediates the pro-malignant effects of oncostatin M receptor over-expression in cervical squamous cell carcinoma. Oncostatin M receptor (OSMR) is commonly over-expressed in advanced cervical squamous cell carcinoma (SCC), producing a significantly worse clinical outcome. Cervical SCC cells that over-express OSMR show enhanced responsiveness to the major ligand OSM, which induces multiple pro-malignant effects, including increased cell   migration   and invasiveness. Here, we show that tissue transglutaminase (TGM2) is an important mediator of the ligand-dependent phenotypic effects of OSMR over-expression in SCC cells. TGM2 expression correlated with disease progression and with OSMR levels in clinical samples of cervical and oral SCC. TGM2 depletion in cervical SCC cells abrogated OSM-induced   migration   on fibronectin-coated surfaces and invasiveness through extracellular matrix, while ectopic expression of TGM2 increased cell motility and invasiveness. Confocal microscopy and co-immunoprecipitation assays showed that TGM2 interacted with integrin-α5β1 in the presence of fibronectin in cervical SCC cells, with OSM treatment strengthening the interaction. Importantly, integrin-α5β1 and fibronectin were also over-expressed in cervical and oral SCC, where levels correlated with those of OSMR and TGM2. This combined tissue and in vitro study demonstrates for the first time that stimulation of over-expressed OSMR in cervical SCC cells activates TGM2/integrin-α5β1 interactions and induces pro-malignant changes. We conclude that an OSMR/TGM2/integrin-α5β1/fibronectin pathway is of biological significance in cervical SCC and a candidate for therapeutic targeting. 
 Overexpression of the oncostatin M receptor in cervical squamous cell carcinoma cells is associated with a pro-angiogenic phenotype and increased cell motility and invasiveness. Oncostatin M receptor (OSMR) shows frequent copy number gain and overexpression in advanced cervical squamous cell carcinoma (SCC). We used cell-based in vitro assays, RNA interference, and integrative gene expression profiling to investigate the functional significance of this observation. CaSki and SW756 were selected as representative cervical SCC cells that overexpressed OSMR, and ME180 and MS751 as cells that did not. The STAT-dependent pro-angiogenic factors VEGF-A and ID1 were rapidly induced by OSM in CaSki/SW756 but not in ME180/MS751. However, rapid induction did occur in MS751 following forced OSMR overexpression, while depleting OSMR in CaSki abrogated VEGF-A expression. Conditioned medium from both CaSki and SW756 stimulated endothelial tube formation in vitro, effects that were inhibited by depleting OSMR in the SCC cells. For both CaSki and SW756,   migration   in a wound healing assay and invasion through Matrigel were stimulated by OSM and consistently inhibited by OSMR depletion. The phenotype was rescued by transfection with OSMR containing a silent mutation that provided specific siRNA resistance. Overall, there was a positive correlation between OSMR levels and invasiveness. We used gene expression profiling to identify genes induced by OSM in CaSki/SW756 but not in ME180/MS751. The most prominent gene ontology category groups for the differentially expressed genes were cell motility/invasion, angiogenesis, signal transduction, and apoptosis. We also profiled 23 cervical SCC samples, identifying genes that were differentially expressed in cases with OSMR overexpression versus those without. Integration of the datasets identified 15 genes that showed consistent differential expression in association with OSMR levels in vitro and in vivo. We conclude that OSMR overexpression in cervical SCC cells provides increased sensitivity to OSM, which induces pro-malignant changes. OSMR is a potential prognostic and therapeutic target in cervical SCC. The genes that mediate OSM:OSMR effects will be valuable indicators of the effectiveness of antibody blockade in pre-clinical systems. 
  [Ee]xtracellular [Mm]atrix  PubMed  
 Tissue transglutaminase mediates the pro-malignant effects of oncostatin M receptor over-expression in cervical squamous cell carcinoma. Oncostatin M receptor (OSMR) is commonly over-expressed in advanced cervical squamous cell carcinoma (SCC), producing a significantly worse clinical outcome. Cervical SCC cells that over-express OSMR show enhanced responsiveness to the major ligand OSM, which induces multiple pro-malignant effects, including increased cell migration and invasiveness. Here, we show that tissue transglutaminase (TGM2) is an important mediator of the ligand-dependent phenotypic effects of OSMR over-expression in SCC cells. TGM2 expression correlated with disease progression and with OSMR levels in clinical samples of cervical and oral SCC. TGM2 depletion in cervical SCC cells abrogated OSM-induced migration on fibronectin-coated surfaces and invasiveness through   extracellular matrix  , while ectopic expression of TGM2 increased cell motility and invasiveness. Confocal microscopy and co-immunoprecipitation assays showed that TGM2 interacted with integrin-α5β1 in the presence of fibronectin in cervical SCC cells, with OSM treatment strengthening the interaction. Importantly, integrin-α5β1 and fibronectin were also over-expressed in cervical and oral SCC, where levels correlated with those of OSMR and TGM2. This combined tissue and in vitro study demonstrates for the first time that stimulation of over-expressed OSMR in cervical SCC cells activates TGM2/integrin-α5β1 interactions and induces pro-malignant changes. We conclude that an OSMR/TGM2/integrin-α5β1/fibronectin pathway is of biological significance in cervical SCC and a candidate for therapeutic targeting. 
  [Ii]nvasive  PubMed  
 Evaluation of candidate stromal epithelial cross-talk genes identifies association between risk of serous ovarian cancer and TERT, a cancer susceptibility "hot-spot". We hypothesized that variants in genes expressed as a consequence of interactions between ovarian cancer cells and the host micro-environment could contribute to cancer susceptibility. We therefore used a two-stage approach to evaluate common single nucleotide polymorphisms (SNPs) in 173 genes involved in stromal epithelial interactions in the Ovarian Cancer Association Consortium (OCAC). In the discovery stage, cases with epithelial ovarian cancer (n=675) and controls (n=1,162) were genotyped at 1,536 SNPs using an Illumina GoldenGate assay. Based on Positive Predictive Value estimates, three SNPs-PODXL rs1013368, ITGA6 rs13027811, and MMP3 rs522616-were selected for replication using TaqMan genotyping in up to 3,059 serous   invasive   cases and 8,905 controls from 16 OCAC case-control studies. An additional 18 SNPs with Pper-allele<0.05 in the discovery stage were selected for replication in a subset of five OCAC studies (n=1,233 serous   invasive   cases; n=3,364 controls). The discovery stage associations in PODXL, ITGA6, and MMP3 were attenuated in the larger replication set (adj. Pper-allele>or=0.5). However genotypes at TERT rs7726159 were associated with ovarian cancer risk in the smaller, five-study replication study (Pper-allele=0.03). Combined analysis of the discovery and replication sets for this TERT SNP showed an increased risk of serous ovarian cancer among non-Hispanic whites [adj. ORper-allele 1.14 (1.04-1.24) p=0.003]. Our study adds to the growing evidence that, like the 8q24 locus, the telomerase reverse transcriptase locus at 5p15.33, is a general cancer susceptibility locus. 
 Evaluation of candidate stromal epithelial cross-talk genes identifies association between risk of serous ovarian cancer and TERT, a cancer susceptibility "hot-spot". We hypothesized that variants in genes expressed as a consequence of interactions between ovarian cancer cells and the host micro-environment could contribute to cancer susceptibility. We therefore used a two-stage approach to evaluate common single nucleotide polymorphisms (SNPs) in 173 genes involved in stromal epithelial interactions in the Ovarian Cancer Association Consortium (OCAC). In the discovery stage, cases with epithelial ovarian cancer (n=675) and controls (n=1,162) were genotyped at 1,536 SNPs using an Illumina GoldenGate assay. Based on Positive Predictive Value estimates, three SNPs-PODXL rs1013368, ITGA6 rs13027811, and MMP3 rs522616-were selected for replication using TaqMan genotyping in up to 3,059 serous   invasive   cases and 8,905 controls from 16 OCAC case-control studies. An additional 18 SNPs with Pper-allele<0.05 in the discovery stage were selected for replication in a subset of five OCAC studies (n=1,233 serous   invasive   cases; n=3,364 controls). The discovery stage associations in PODXL, ITGA6, and MMP3 were attenuated in the larger replication set (adj. Pper-allele>or=0.5). However genotypes at TERT rs7726159 were associated with ovarian cancer risk in the smaller, five-study replication study (Pper-allele=0.03). Combined analysis of the discovery and replication sets for this TERT SNP showed an increased risk of serous ovarian cancer among non-Hispanic whites [adj. ORper-allele 1.14 (1.04-1.24) p=0.003]. Our study adds to the growing evidence that, like the 8q24 locus, the telomerase reverse transcriptase locus at 5p15.33, is a general cancer susceptibility locus. 
 Tissue transglutaminase mediates the pro-malignant effects of oncostatin M receptor over-expression in cervical squamous cell carcinoma. Oncostatin M receptor (OSMR) is commonly over-expressed in advanced cervical squamous cell carcinoma (SCC), producing a significantly worse clinical outcome. Cervical SCC cells that over-express OSMR show enhanced responsiveness to the major ligand OSM, which induces multiple pro-malignant effects, including increased cell migration and   invasive  ness. Here, we show that tissue transglutaminase (TGM2) is an important mediator of the ligand-dependent phenotypic effects of OSMR over-expression in SCC cells. TGM2 expression correlated with disease progression and with OSMR levels in clinical samples of cervical and oral SCC. TGM2 depletion in cervical SCC cells abrogated OSM-induced migration on fibronectin-coated surfaces and   invasive  ness through extracellular matrix, while ectopic expression of TGM2 increased cell motility and   invasive  ness. Confocal microscopy and co-immunoprecipitation assays showed that TGM2 interacted with integrin-α5β1 in the presence of fibronectin in cervical SCC cells, with OSM treatment strengthening the interaction. Importantly, integrin-α5β1 and fibronectin were also over-expressed in cervical and oral SCC, where levels correlated with those of OSMR and TGM2. This combined tissue and in vitro study demonstrates for the first time that stimulation of over-expressed OSMR in cervical SCC cells activates TGM2/integrin-α5β1 interactions and induces pro-malignant changes. We conclude that an OSMR/TGM2/integrin-α5β1/fibronectin pathway is of biological significance in cervical SCC and a candidate for therapeutic targeting. 
 Overexpression of the oncostatin M receptor in cervical squamous cell carcinoma cells is associated with a pro-angiogenic phenotype and increased cell motility and   invasive  ness. Oncostatin M receptor (OSMR) shows frequent copy number gain and overexpression in advanced cervical squamous cell carcinoma (SCC). We used cell-based in vitro assays, RNA interference, and integrative gene expression profiling to investigate the functional significance of this observation. CaSki and SW756 were selected as representative cervical SCC cells that overexpressed OSMR, and ME180 and MS751 as cells that did not. The STAT-dependent pro-angiogenic factors VEGF-A and ID1 were rapidly induced by OSM in CaSki/SW756 but not in ME180/MS751. However, rapid induction did occur in MS751 following forced OSMR overexpression, while depleting OSMR in CaSki abrogated VEGF-A expression. Conditioned medium from both CaSki and SW756 stimulated endothelial tube formation in vitro, effects that were inhibited by depleting OSMR in the SCC cells. For both CaSki and SW756, migration in a wound healing assay and invasion through Matrigel were stimulated by OSM and consistently inhibited by OSMR depletion. The phenotype was rescued by transfection with OSMR containing a silent mutation that provided specific siRNA resistance. Overall, there was a positive correlation between OSMR levels and   invasive  ness. We used gene expression profiling to identify genes induced by OSM in CaSki/SW756 but not in ME180/MS751. The most prominent gene ontology category groups for the differentially expressed genes were cell motility/invasion, angiogenesis, signal transduction, and apoptosis. We also profiled 23 cervical SCC samples, identifying genes that were differentially expressed in cases with OSMR overexpression versus those without. Integration of the datasets identified 15 genes that showed consistent differential expression in association with OSMR levels in vitro and in vivo. We conclude that OSMR overexpression in cervical SCC cells provides increased sensitivity to OSM, which induces pro-malignant changes. OSMR is a potential prognostic and therapeutic target in cervical SCC. The genes that mediate OSM:OSMR effects will be valuable indicators of the effectiveness of antibody blockade in pre-clinical systems. 
 Methylation of OSMR gene is frequently observed in non-  invasive   colorectal cancer. Recently, it has been reported that oncostatin M receptor-β (OSMR) is frequently methylated in primary colon cancer tissues, but not in normal tissues. We examined the methylation status of the OSMR gene in primary carcinomas and the corresponding normal tissues derived from 56 patients with colorectal cancer.The methylation status of the OSMR gene was examined in primary carcinomas and corresponding normal tissues derived from 56 patients with colorectal cancer using quantitative methylation-specific PCR (qMSP), and the correlation between the methylation status and the clinicopathological findings was evaluated.Methylation of the OSMR gene was detected in 18 out of the 56 (32%) primary colon carcinomas. The clinicopathological data were then compared with the methylation results. A significant difference was observed in regard to the extent of tumour (p=0.0442). These results indicated that OSMR was more frequently methylated in non  -invasiv  e colorectal carcinomas.OSMR may act as a tumour suppressor in colorectal carcinoma and OSMR methylation may play an important role in non  -invasiv  e colorectal cancer. 
  GeneRIFs  
 Aberrant methylation of the OSMR gene is associated with non-  invasive   colorectal cancer. 
   
  
 ZNF335 
 
  NCBI Gene  
  GeneRIF  
  Pubmed  
 
 
  Search Term  Rank  Fields  
  [Ii]nvasive  10  PubMed(1)  
 
 
 hide/show details for ZNF335 
  [Ii]nvasive  PubMed  
 IMAC fractionation in combination with LC-MS reveals H2B and NIF-1 peptides as potential bladder cancer biomarkers. Improvement in bladder cancer (BC) management requires more effective diagnosis and prognosis of disease recurrence and progression. Urinary biomarkers attract special interest because of the non  invasive   means of urine collection. Proteomic analysis of urine entails the adoption of a fractionation methodology to reduce sample complexity. In this study, we applied immobilized metal affinity chromatography in combination with high-resolution LC-MS/MS for the discovery of native urinary peptides potentially associated with BC aggressiveness. This approach was employed toward urine samples from patients with   invasive   BC, non  invasive   BC, and benign urogenital diseases. A total of 1845 peptides were identified, corresponding to a total of 638 precursor proteins. Specific enrichment for proteins involved in nucleosome assembly and for zinc-finger transcription factors was observed. The differential expression of two candidate biomarkers, histone H2B and NIF-1 (zinc finger 335) in BC, was verified in independent sets of urine samples by ELISA and by immunohistochemical analysis of BC tissue. The results collectively support changes in the expression of both of these proteins with tumor progression, suggesting their potential role as markers for discriminating BC stages. In addition, the data indicate a possible involvement of NIF-1 in BC progression, likely as a suppressor and through interactions with Sox9 and HoxA1. 
   
  
 REST 
 
  NCBI Gene  
  GeneRIF  
  Pubmed  
 
 
  Search Term  Rank  Fields  
  [Mm]igration  6  PubMed(4); GeneRIFs(3)  
  ECM  7  PubMed(1)  
  [Ee]xtracellular [Mm]atrix  8  PubMed(2)  
  [Ii]nvasive  10  PubMed(2)  
  [Mm]etastasis  12  PubMed(3)  
 
 
 hide/show details for REST 
  [Mm]igration  PubMed  
 Neuron restrictive silencer factor NRSF/REST is a transcriptional repressor of neuropilin-1 and diminishes the ability of semaphorin 3A to inhibit keratinocyte   migration  . Neuropilin-1 (NRP1) is expressed by endothelial cells and neurons and serves as a receptor for both vascular endothelial growth factor (VEGF), an angiogenesis factor, and semaphorin 3A (Sema3A), a mediator of axonal guidance. We show here that NRP1 is also expressed in keratinocytes in vitro and in vivo. However, nothing has been reported about the regulation or function of keratinocyte NRP1. Using NRP1 promoter constructs in HaCaT cells, a keratinocyte cell line, we could demonstrate that a neuron restrictive silencer element (NRSE) was implicated in transcriptional repression of the NRP1 gene. Electrophoretic mobility shift assays demonstrated that the neuron restrictive silencer factor (NRSF) binds to NRSE. Overexpression of NRSF in HaCaT cells decreased NRP1 RNA and protein, whereas a dominant negative NRSF increased NRP1. Furthermore, the histone deacetylase inhibitor trichostatin A, an inhibitor of NRSF silencing activity, also increased NRP1 levels. NRP2 expression was not affected. Epidermal growth factor (EGF) and heparin-binding EGF-like growth factor (HB-EGF) strongly up-regulated NRP1 expression, concomitant with down-regulation of NRSF. Other keratinocyte mitogens such as keratinocyte growth factor (KGF) had no effect. To address function, HaCaT cells were exposed to two NRP1 ligands, VEGF165 and Sema3A. Neither had an effect on proliferation, whereas Sema3A, but not VEGF165, inhibited cell   migration  . Down-regulation of NRP1 by NRSF overexpression reduced Sema3A activity. It was concluded that NRSF is a transcription factor that silences NRP1 expression and thereby diminishes the Sema3A mediated inhibition of HaCaT keratinocyte   migration  . 
 Repressor element 1 silencing transcription factor (REST) controls radial   migration   and temporal neuronal specification during neocortical development. Neurogenesis requires mechanisms that coordinate early cell-fate decisions,   migration  , and terminal differentiation. Here, we show that the transcriptional repressor, repressor element 1 silencing transcription factor (REST), regulates radial   migration   and the timing of neural progenitor differentiation during neocortical development, and that the regulation is contingent upon differential REST levels. Specifically, a sustained presence of REST blocks   migration   and greatly delays--but does not prevent--neuronal differentiation, resulting in a subcortical band heterotopia-like phenotype, reminiscent of loss of doublecortin. We further show that doublecortin is a direct gene target of REST, and that its overexpression rescues, at least in part, the aberrant phenotype caused by persistent presence of REST. Our studies support the view that the targeted down-regulation of REST to low levels in neural progenitors, and its subsequent disappearance during neurogenesis, is critical for timing the spatiotemporal transition of neural progenitor cells to neurons. 
 RE-1-silencing transcription factor shows tumor-suppressor functions and negatively regulates the oncogenic TAC1 in breast cancer cells. Breast cancer remains the most prevalent cancer among women in the United States. Substance P, a peptide derived from the TAC1 gene, mediates oncogenic properties in breast and other cancers. TAC1 expression facilitates the entry of breast cancer cells into bone marrow. The transcriptional repressor element 1-silencing transcription factor (REST) has been implicated in both oncogenic and tumor-suppressor functions. REST binds to the 5' untranslated region of the TAC1 promoter and suppresses its expression. This study investigated a role for REST in TAC1 induction in breast cancer. Western blots and real-time PCR indicated that REST expression in breast cancer cells was inversely proportional to the cells' aggressiveness, for both cell lines and primary breast cancer cells. REST knockdown in low-metastatic T47D cells and nontumorigenic MCF12A cells resulted in increases in TAC1 induction, proliferation, and   migration  . These parameters were negatively affected by ectopic expression of REST in highly aggressive MDA-MB-231 cells. Together, these findings show a central role for REST in the oncogenic function of TAC1 and suggest a tumor-suppressor role for REST in breast cancer. 
 Type 1 Insulin-Like Growth Factor Receptor/Insulin Receptor Substrate 1 Signaling Confers Pathogenic Activity on Breast Tumor Cells Lacking REST. Loss of repressor element 1 silencing transcription factor (REST) occurs in 20% of breast cancers and correlates with a poor patient prognosis. However, the molecular basis for enhanced malignancy in tumors lacking REST (RESTless) is only partially understood. We used multiplatform array data from the Cancer Genome Atlas to identify consistent changes in key signaling pathways. Of the proteins screened in the reverse-phase protein array, we found that insulin receptor substrate 1 (IRS1) is the most highly upregulated protein in RESTless breast tumors. Analysis of breast tumor cell lines showed that REST directly represses IRS1, and cells lacking REST have increased levels of IRS1 mRNA and protein. We find that the upregulation of IRS1 function is both necessary and sufficient for enhanced signaling and growth in breast cancer cells lacking REST. IRS1 overexpression is sufficient to phenocopy the enhanced activation of the signaling hubs AKT and mitogen-activated protein kinase (MAPK) of MCF7 cells lacking REST. Loss of REST renders MCF7 and MDA-MB-231 breast tumor cells dependent on IRS1 activity for colony formation in soft agar. Inhibition of the type 1 insulin-like growth factor receptor (IGF1R) reduces the enhanced signaling, growth, and   migration   in breast tumor cells that occur upon REST loss. We show that loss of REST induces a pathogenic program that works through the IGF1R/IRS1 pathway. 
  GeneRIFs  
 Repressor element 1 silencing transcription factor (REST) controls radial   migration   and temporal neuronal specification during neocortical development. 
 REST knockdown in low-metastatic cells and nontumorigenic cells resulted in increases in TAC1 induction, proliferation, and   migration   
 Down-regulation of NRP1 by NRSF overexpression reduced Sema3A activity. It was concluded that NRSF is a transcription factor that silences NRP1 expression and thereby diminishes the Sema3A mediated inhibition of HaCaT keratinocyte   migration   
  ECM  PubMed  
 The small cell lung cancer-specific isoform of RE1-silencing transcription factor (REST) is regulated by neural-specific Ser/Arg repeat-related protein of 100 kDa (nSR100). Small cell lung cancer (SCLC) is a highly malignant form of cancer, which originates from primitive neuroendocrine cells in the lung. SCLC cells express several autocrine neurotransmitters/neuropeptides and their respective receptors. Expression of these neuronal markers is frequently regulated by RE1-silencing transcription factor (REST). In SCLC cells, an SCLC-specific isoform of REST (sREST) is highly expressed, whereas REST expression is undetectable, suggesting that the expression of sREST correlates with the pathogenesis of SCLC. Expression of sREST, which is derived through alternative splicing of REST, is abnormally regulated in SCLC cells, but the mechanism is unknown. Most recently, nSR100 (SRRM4) was described as an activator of REST alternative splicing. We now show that nSR100 is highly expressed in SCLC cells correlating with high sREST and low REST expression. Adhesion to the extracellular matrix (  ECM  ) is thought to enhance tumorigenicity and confer resistance to apoptosis. Interestingly, nSR100 expression is enhanced in cells grown with   ECM  . Overexpression of REST caused repression of sREST and nSR100, the latter containing RE1 element controlled by REST. Culturing the SCLC cell line NCI-N417 cells with   ECM   also upregulated RE1-containing gene, the voltage-gated calcium channel subunit. Inhibition of the PI3K/Akt/mTOR pathway by LY294002 induced nSR100 expression, whereas the specific MEK/ERK inhibitor U0126 inhibited nSR100 expression. Repressing nSR100 by siRNA effectively repressed sREST, and conversely increased REST in NCI-N417 cells. Taken together, this report clarifies the   ECM  -dependent signaling pathway that impacts nSR100 expression and its regulation of alternative splicing in SCLC.The splicing factor nSR100 may be novel SCLC-specific biomarker, as well as a therapeutic target. 
  [Ee]xtracellular [Mm]atrix  PubMed  
 Loss of the repressor REST in uterine fibroids promotes aberrant G protein-coupled receptor 10 expression and activates mammalian target of rapamycin pathway. Uterine fibroids (leiomyomas) are the most common tumors of the female reproductive tract, occurring in up to 77% of reproductive-aged women, yet molecular pathogenesis remains poorly understood. A role for atypically activated mammalian target of rapamycin (mTOR) pathway in the pathogenesis of uterine fibroids has been suggested in several studies. We identified that G protein-coupled receptor 10 [GPR10, a putative signaling protein upstream of the phosphoinositide 3-kinase-protein kinase B/AKT-mammalian target of rapamycin (PI3K/AKT-mTOR) pathway] is aberrantly expressed in uterine fibroids. The activation of GPR10 by its cognate ligand, prolactin releasing peptide, promotes PI3K-AKT-mTOR pathways and cell proliferation specifically in cultured primary leiomyoma cells. Additionally, we report that RE1 suppressing transcription factor/neuron-restrictive silencing factor (REST/NRSF), a known tumor suppressor, transcriptionally represses GPR10 in the normal myometrium, and that the loss of REST in fibroids permits GPR10 expression. Importantly, mice overexpressing human GPR10 in the myometrium develop myometrial hyperplasia with excessive   extracellular matrix   deposition, a hallmark of uterine fibroids. We demonstrate previously unrecognized roles for GPR10 and its upstream regulator REST in the pathogenesis of uterine fibroids. Importantly, we report a unique genetically modified mouse model for a gene that is misexpressed in uterine fibroids. 
 The small cell lung cancer-specific isoform of RE1-silencing transcription factor (REST) is regulated by neural-specific Ser/Arg repeat-related protein of 100 kDa (nSR100). Small cell lung cancer (SCLC) is a highly malignant form of cancer, which originates from primitive neuroendocrine cells in the lung. SCLC cells express several autocrine neurotransmitters/neuropeptides and their respective receptors. Expression of these neuronal markers is frequently regulated by RE1-silencing transcription factor (REST). In SCLC cells, an SCLC-specific isoform of REST (sREST) is highly expressed, whereas REST expression is undetectable, suggesting that the expression of sREST correlates with the pathogenesis of SCLC. Expression of sREST, which is derived through alternative splicing of REST, is abnormally regulated in SCLC cells, but the mechanism is unknown. Most recently, nSR100 (SRRM4) was described as an activator of REST alternative splicing. We now show that nSR100 is highly expressed in SCLC cells correlating with high sREST and low REST expression. Adhesion to the   extracellular matrix   (ECM) is thought to enhance tumorigenicity and confer resistance to apoptosis. Interestingly, nSR100 expression is enhanced in cells grown with ECM. Overexpression of REST caused repression of sREST and nSR100, the latter containing RE1 element controlled by REST. Culturing the SCLC cell line NCI-N417 cells with ECM also upregulated RE1-containing gene, the voltage-gated calcium channel subunit. Inhibition of the PI3K/Akt/mTOR pathway by LY294002 induced nSR100 expression, whereas the specific MEK/ERK inhibitor U0126 inhibited nSR100 expression. Repressing nSR100 by siRNA effectively repressed sREST, and conversely increased REST in NCI-N417 cells. Taken together, this report clarifies the ECM-dependent signaling pathway that impacts nSR100 expression and its regulation of alternative splicing in SCLC.The splicing factor nSR100 may be novel SCLC-specific biomarker, as well as a therapeutic target. 
  [Ii]nvasive  PubMed  
 Induction of the RNA regulator LIN28A is required for the growth and pathogenesis of RESTless breast tumors. The transcription factor RE1 silencing transcription factor (REST) is lost in approximately 20% of breast cancers. Although it is known that these RESTless tumors are highly aggressive and include all tumor subtypes, the underlying tumorigenic mechanisms remain unknown. In this study, we show that loss of REST results in upregulation of LIN28A, a known promoter of tumor development, in breast cancer cell lines and human breast tumors. We found that LIN28A was a direct transcriptional target of REST in cancer cells and that loss of REST resulted in increased LIN28A expression and enhanced tumor growth both in vitro and in vivo, effects that were dependent on heightened LIN28A expression. Tumors lacking REST expression were locally   invasive  , consistent with the increased lymph node involvement observed in human RESTless tumors. Clinically, human RESTless breast tumors also displayed significantly enhanced LIN28A expression when compared with non-RESTless tumors. Our findings therefore show a critical role for the REST-LIN28A axis in tumor aggression and suggest a causative relationship between REST loss and tumorigenicity in vivo. 
 REST regulates oncogenic properties of glioblastoma stem cells. Glioblastoma multiforme (GBM) tumors are the most common malignant primary brain tumors in adults. Although many GBM tumors are believed to be caused by self-renewing, glioblastoma-derived stem-like cells (GSCs), the mechanisms that regulate self-renewal and other oncogenic properties of GSCs are only now being unraveled. Here we showed that GSCs derived from GBM patient specimens express varying levels of the transcriptional repressor repressor element 1 silencing transcription factor (REST), suggesting heterogeneity across different GSC lines. Loss- and gain-of-function experiments indicated that REST maintains self-renewal of GSCs. High REST-expressing GSCs (HR-GSCs) produced tumors histopathologically distinct from those generated by low REST-expressing GSCs (LR-GSCs) in orthotopic mouse brain tumor models. Knockdown of REST in HR-GSCs resulted in increased survival in GSC-transplanted mice and produced tumors with higher apoptotic and lower   invasive   properties. Conversely, forced expression of exogenous REST in LR-GSCs produced decreased survival in mice and produced tumors with lower apoptotic and higher   invasive   properties, similar to HR-GSCs. Thus, based on our results, we propose that a novel function of REST is to maintain self-renewal and other oncogenic properties of GSCs and that REST can play a major role in mediating tumorigenicity in GBM. 
  [Mm]etastasis  PubMed  
 Demethylation of Repressor Element-1 Silencing Transcription (REST) Suppresses the Malignant Phenotype of Breast Cancer via MMP9. Breast cancer is the leading cause of cancer deaths in females all over the world, mainly resulting from   metastasis  . Previous studies have revealed that repressor element-1 (RE-1) silencing transcription (REST) acted as a tumor suppressor in breast cancer. However, the mechanism by which REST is regulated remains unknown, and its role in the   metastasis   in breast cancer cells remains unclear. In the present study, we showed that the expression of REST was lower in breast cancer samples than that of adjacent samples by immunohistochemical analysis, which may be due to hypermethylation of the REST promoter. Low REST levels are significantly associated with malignant progression in breast cancer patients. Additionally, we elucidated the functions of REST on proliferation and invasion in breast cancer cells. Lentivirus transfection was used to overexpress REST in human breast MDA-MB-231 cells. Then the biologic consequences of overexpressing REST in regard to cell proliferation, apoptosis, and invasion were determined. Furthermore, we also determined matrix metalloproteinase-9 (MMP9) as a target of REST. These results demonstrate that downregulation of REST, a tumor suppressor in breast cancer, is associated with hypermethylation. Induced REST expression is capable of attenuating invasion ability of breast cancer cells, which may be a novel strategy for metastatic breast cancer treatment. 
 Down-regulation of RE-1 silencing transcription factor (REST) in advanced prostate cancer by hypoxia-induced miR-106b~25. Clinically aggressive prostate cancer (PCa) is linked to androgen resistance,   metastasis  , and expression of neuroendocrine markers. To understand mechanism(s) of neuroendocrine differentiation (NED) of PCa epithelia, we compared neuronal differentiation occurring during embryogenesis, in primary cultures of neural crest (NC) cells, and NED in PCa cell lines (LNCaP and PC3). We demonstrate, hypoxia promotes neuronal and neuroendocrine differentiation of NC cells and PCa cells, respectively, by inducing the miR-106 b~25 cluster. In turn, miR-106b~25 comprised of miR-106b, miR-93 and miR-25, down-regulates the transcriptional repressor REST, which represses neuron-specific protein-coding and miRNA genes. In prostate tumors of high Gleason score (≥ 8), an inverse trend was observed between REST and miR-106b~25 induction. Employing miRNA PCR arrays, we identified miRNAs up-regulated by hypoxia in LNCaP cells and REST-knockdown in NC cells. Significantly, a subset of miRNAs (miR-9, miR-25, miR-30d and miR302b) is up-regulated in high Gleason score (≥ 8) PCa, suggesting a mechanism by which NED contributes to PCa malignancy. We propose that loss of REST and induction of this set of microRNAs can serve as potential novel clinical markers of advanced PCa. 
 The oncogenic STP axis promotes triple-negative breast cancer via degradation of the REST tumor suppressor. Defining the molecular networks that drive breast cancer has led to therapeutic interventions and improved patient survival. However, the aggressive triple-negative breast cancer subtype (TNBC) remains recalcitrant to targeted therapies because its molecular etiology is poorly defined. In this study, we used a forward genetic screen to discover an oncogenic network driving human TNBC. SCYL1, TEX14, and PLK1 ("STP axis") cooperatively trigger degradation of the REST tumor suppressor protein, a frequent event in human TNBC. The STP axis induces REST degradation by phosphorylating a conserved REST phospho-degron and bridging REST interaction with the ubiquitin-ligase βTRCP. Inhibition of the STP axis leads to increased REST protein levels and impairs TNBC transformation, tumor progression, and   metastasi  s. Expression of the STP axis correlates with low REST protein levels in human TNBCs and poor clinical outcome for TNBC patients. Our findings demonstrate that the STP-REST axis is a molecular driver of human TNBC. 
   
  
 CES1 
 
  NCBI Gene  
  GeneRIF  
  Pubmed  
 
 
  Search Term  Rank  Fields  
  [Mm]etastasis  12  PubMed(2)  
 
 
 hide/show details for CES1 
  [Mm]etastasis  PubMed  
 Microarray analysis identifies a death-from-cancer signature predicting therapy failure in patients with multiple types of cancer. Activation in transformed cells of normal stem cells' self-renewal pathways might contribute to the survival life cycle of cancer stem cells and promote tumor progression. The BMI-1 oncogene-driven gene expression pathway is essential for the self-renewal of hematopoietic and neural stem cells. We applied a mouse/human comparative translational genomics approach to identify an 11-gene signature that consistently displays a stem cell-resembling expression profile in distant metastatic lesions as revealed by the analysis of metastases and primary tumors from a transgenic mouse model of prostate cancer and cancer patients. To further validate these results, we examined the prognostic power of the 11-gene signature in several independent therapy-outcome sets of clinical samples obtained from 1,153 cancer patients diagnosed with 11 different types of cancer, including 5 epithelial malignancies (prostate, breast, lung, ovarian, and bladder cancers) and 5 nonepithelial malignancies (lymphoma, mesothelioma, medulloblastoma, glioma, and acute myeloid leukemia). Kaplan-Meier analysis demonstrated that a stem cell-like expression profile of the 11-gene signature in primary tumors is a consistent powerful predictor of a short interval to disease recurrence, distant   metastasis  , and death after therapy in cancer patients diagnosed with 11 distinct types of cancer. These data suggest the presence of a conserved BMI-1-driven pathway, which is similarly engaged in both normal stem cells and a highly malignant subset of human cancers diagnosed in a wide range of organs and uniformly exhibiting a marked propensity toward metastatic dissemination as well as a high probability of unfavorable therapy outcome. 
 Microarray analysis identifies a death-from-cancer signature predicting therapy failure in patients with multiple types of cancer. Activation in transformed cells of normal stem cells' self-renewal pathways might contribute to the survival life cycle of cancer stem cells and promote tumor progression. The BMI-1 oncogene-driven gene expression pathway is essential for the self-renewal of hematopoietic and neural stem cells. We applied a mouse/human comparative translational genomics approach to identify an 11-gene signature that consistently displays a stem cell-resembling expression profile in distant metastatic lesions as revealed by the analysis of metastases and primary tumors from a transgenic mouse model of prostate cancer and cancer patients. To further validate these results, we examined the prognostic power of the 11-gene signature in several independent therapy-outcome sets of clinical samples obtained from 1,153 cancer patients diagnosed with 11 different types of cancer, including 5 epithelial malignancies (prostate, breast, lung, ovarian, and bladder cancers) and 5 nonepithelial malignancies (lymphoma, mesothelioma, medulloblastoma, glioma, and acute myeloid leukemia). Kaplan-Meier analysis demonstrated that a stem cell-like expression profile of the 11-gene signature in primary tumors is a consistent powerful predictor of a short interval to disease recurrence, distant   metastasis  , and death after therapy in cancer patients diagnosed with 11 distinct types of cancer. These data suggest the presence of a conserved BMI-1-driven pathway, which is similarly engaged in both normal stem cells and a highly malignant subset of human cancers diagnosed in a wide range of organs and uniformly exhibiting a marked propensity toward metastatic dissemination as well as a high probability of unfavorable therapy outcome. 
   
  
 CES3 
 
  NCBI Gene  
  GeneRIF  
  Pubmed  
 
 
  Search Term  Rank  Fields  
  [Ee]xtracellular [Mm]atrix  8  PubMed(1)  
 
 
 hide/show details for CES3 
  [Ee]xtracellular [Mm]atrix  PubMed  
 Proteomic analysis of human parotid gland exosomes by multidimensional protein identification technology (MudPIT). Human ductal saliva contributes over a thousand unique proteins to whole oral fluids. The mechanism by which most of these proteins are secreted by salivary glands remains to be determined. The present study used a mass spectrometry-based, shotgun proteomics approach to explore the possibility that a subset of the proteins found in saliva are derived from exosomes, membrane-bound vesicles of endosomal origin within multivesicular endosomes. Using MudPIT (multidimensional protein identification technology) mass spectrometry, we catalogued 491 proteins in the exosome fraction of human parotid saliva. Many of these proteins were previously observed in ductal saliva from parotid glands (265 proteins). Furthermore, 72 of the proteins in parotid exosomes overlap with those previously identified as urinary exosome proteins, proteins which are also frequently associated with exosomes from other tissues and cell types. Gene Ontology (GO) and KEGG pathway analyses found that cytosolic proteins comprise the largest category of proteins in parotid exosomes (43%), involved in such processes as phosphatidylinositol signaling system, calcium signaling pathway, inositol metabolism, protein export, and signal transduction, among others; whereas the integral plasma membrane proteins and associated/peripheral plasma membrane proteins (26%) were associated with   extracellular matrix  -receptor interaction, epithelial cell signaling, T-cell and B-cell receptor signaling, cytokine receptor interaction, and antigen processing and presentation, among other biological functions. In addition, these putative saliva exosomal proteins were linked to specific diseases (e.g., neurodegenerative disorders, prion disease, cancers, type I and II diabetes). Consequently, parotid glands secrete exosomes that reflect the metabolic and functional status of the gland and may also carry informative protein markers useful in the diagnosis and treatment of systemic diseases. 
   
  
 RNASEL 
 
  NCBI Gene  
  GeneRIF  
  Pubmed  
 
 
  Search Term  Rank  Fields  
  [Mm]igration  6  PubMed(2); GeneRIFs(1)  
  [Ii]nvasive  10  PubMed(3)  
  [Mm]etastasis  12  PubMed(2); GeneRIFs(1)  
 
 
 hide/show details for RNASEL 
  [Mm]igration  PubMed  
 RNase L Suppresses Androgen Receptor Signaling, Cell   Migration   and Matrix Metalloproteinase Activity in Prostate Cancer Cells. The interferon antiviral pathways and prostate cancer genetics converge on a regulated endoribonuclease, RNase L. Positional cloning and linkage studies mapped Hereditary Prostate Cancer 1 (HPC1) to RNASEL. To date, there is no correlation of viral infections with prostate cancer, suggesting that RNase L may play additional roles in tumor suppression. Here, we demonstrate a role of RNase L as a suppressor of androgen receptor (AR) signaling, cell   migration   and matrix metalloproteinase activity. Using RNase L mutants, we show that its nucleolytic activity is dispensable for both AR signaling and   migration  . The most prevalent HPC1-associated mutations in RNase L, R462Q and E265X, enhance AR signaling and cell   migration  . RNase L negatively regulates cell   migration   and attachment on various extracellular matrices. We demonstrate that RNase L knockdown cells promote increased cell surface expression of integrin β1 which activates Focal Adhesion Kinase-Sarcoma (FAK-Src) pathway and Ras-related C3 botulinum toxin substrate 1-guanosine triphosphatase (Rac1-GTPase) activity to increase cell   migratio  n. Activity of matrix metalloproteinase (MMP)-2 and -9 is significantly increased in cells where RNase L levels are ablated. We show that mutations in RNase L found in HPC patients may promote prostate cancer by increasing expression of AR-responsive genes and cell motility and identify novel roles of RNase L as a prostate cancer susceptibility gene. 
 RNase L is a negative regulator of cell   migration  . RNase L is a regulated endoribonuclease that functions in the interferon antiviral response. Activation of RNase L by 2', 5'-oligoadenylates has been linked to apoptosis, autophagy and inflammation. Genetic studies have also suggested the possible involvement of the RNase L gene (RNASEL) on chromosome 1q25.3 in several types of cancer. Here we report that ablation of RNase L in human prostate cancer PC3 cells by CRISPR/Cas9 gene editing technology enhanced cell   migration   as determined both by transwell assays and scratch wound healing assays. In addition, RNase L knockdown by means of RNAi increased   migration   of PC3 and DU145 cells in response to either fibronectin or serum stimulation, as did homozygous disruption of the RNase L gene in mouse embryonic fibroblasts. Serum or fibronectin stimulation of focal adhesion kinase (FAK) autophosphorylation on tyrosine-397 was increased by either knockdown or ablation of RNase L. In contrast, a missense mutant RNase L (R667A) lacking catalytic activity failed to suppress cell   migration   in PC3 cells. However, a nuclease-inactive mutant mouse RNase L (W630A) was able to partially inhibit   migration   of mouse fibroblasts. Consistent with a role for the catalytic activity of RNase L, transfection of PC3 cells with the RNase L activator, 2', 5'-oligoadenylate, suppressed cell   migration  . RNase L knockdown in PC3 cells enhanced tumor growth and metastasis following implantation in the mouse prostate. Our results suggest that naturally occurring mutations in the RNase L gene might promote enhanced cell   migration   and metastasis. 
  GeneRIFs  
 Suggest that naturally occurring mutations in the RNase L gene might promote enhanced prostate cancer cell   migration   and metastasis. 
  [Ii]nvasive  PubMed  
 Genetic susceptibility to distinct bladder cancer subphenotypes. Clinical, pathologic, and molecular evidence indicate that bladder cancer is heterogeneous with pathologic/molecular features that define distinct subphenotypes with different prognoses. It is conceivable that specific patterns of genetic susceptibility are associated with particular subphenotypes.To examine evidence for the contribution of germline genetic variation to bladder cancer heterogeneity.The Spanish Bladder Cancer/EPICURO Study is a case-control study based in 18 hospitals located in five areas in Spain. Cases were patients with a newly diagnosed, histologically confirmed, urothelial cell carcinoma of the bladder from 1998 to 2001. Case diagnoses were reviewed and uniformly classified by pathologists following the World Health Organisation/International Society of Urological Pathology 1999 criteria. Controls were hospital-matched patients (n=1149).A total of 1526 candidate variants in 423 candidate genes were analysed. Three distinct subphenotypes were defined according to stage and grade: low-grade nonmuscle   invasive   (n=586), high-grade nonmuscle   invasive   (n=219), and muscle   invasive   (n=246). The association between each variant and subphenotype was assessed by polytomous risk models adjusting for potential confounders. Heterogeneity in genetic susceptibility among subphenotypes was also tested.Two established bladder cancer susceptibility genotypes, NAT2 slow-acetylation and GSTM1-null, exhibited similar associations among the subphenotypes, as did VEGF-rs25648, which was previously identified in our study. Other variants conferred risks for specific tumour subphenotypes such as PMS2-rs6463524 and CD4-rs3213427 (respective heterogeneity p values of 0.006 and 0.004), which were associated with muscle-  invasive   tumours (per-allele odds ratios [95% confidence interval] of 0.56 [0.41-0.77] and 0.71 [0.57-0.88], respectively) but not with non-muscle-  invasive   tumours. Heterogeneity p values were not robust in multiple testing according to their false-discovery rate.These exploratory analyses suggest that genetic susceptibility loci might be related to the molecular/pathologic diversity of bladder cancer. Validation through large-scale replication studies and the study of additional genes and single nucleotide polymorphisms are required. 
 Genetic susceptibility to distinct bladder cancer subphenotypes. Clinical, pathologic, and molecular evidence indicate that bladder cancer is heterogeneous with pathologic/molecular features that define distinct subphenotypes with different prognoses. It is conceivable that specific patterns of genetic susceptibility are associated with particular subphenotypes.To examine evidence for the contribution of germline genetic variation to bladder cancer heterogeneity.The Spanish Bladder Cancer/EPICURO Study is a case-control study based in 18 hospitals located in five areas in Spain. Cases were patients with a newly diagnosed, histologically confirmed, urothelial cell carcinoma of the bladder from 1998 to 2001. Case diagnoses were reviewed and uniformly classified by pathologists following the World Health Organisation/International Society of Urological Pathology 1999 criteria. Controls were hospital-matched patients (n=1149).A total of 1526 candidate variants in 423 candidate genes were analysed. Three distinct subphenotypes were defined according to stage and grade: low-grade nonmuscle   invasive   (n=586), high-grade nonmuscle   invasive   (n=219), and muscle   invasive   (n=246). The association between each variant and subphenotype was assessed by polytomous risk models adjusting for potential confounders. Heterogeneity in genetic susceptibility among subphenotypes was also tested.Two established bladder cancer susceptibility genotypes, NAT2 slow-acetylation and GSTM1-null, exhibited similar associations among the subphenotypes, as did VEGF-rs25648, which was previously identified in our study. Other variants conferred risks for specific tumour subphenotypes such as PMS2-rs6463524 and CD4-rs3213427 (respective heterogeneity p values of 0.006 and 0.004), which were associated with muscle-  invasive   tumours (per-allele odds ratios [95% confidence interval] of 0.56 [0.41-0.77] and 0.71 [0.57-0.88], respectively) but not with non-muscle-  invasive   tumours. Heterogeneity p values were not robust in multiple testing according to their false-discovery rate.These exploratory analyses suggest that genetic susceptibility loci might be related to the molecular/pathologic diversity of bladder cancer. Validation through large-scale replication studies and the study of additional genes and single nucleotide polymorphisms are required. 
 A genome-wide association study of brain lesion distribution in multiple sclerosis. Brain magnetic resonance imaging is widely used as a diagnostic and monitoring tool in multiple sclerosis and provides a non-  invasive  , sensitive and reproducible way to track the disease. Topological characteristics relating to the distribution and shape of lesions are recognized as important neuroradiological markers in the diagnosis of multiple sclerosis, although these have been much less well characterized quantitatively than have traditional measures such as T2 hyperintense or T1 hypointense lesion volumes. Here, we used voxel-level 3 T magnetic resonance imaging T1-weighted scans to reconstruct the 3D topology of lesions in 284 subjects with multiple sclerosis and tested whether this is a heritable phenotype. To this end, we extracted the genotypes from a published genome-wide association study on these same individuals and searched for genetic associations with lesion load, shape and topological distribution. Lesion probability maps were created to identify frequently affected areas and to assess the overall distribution of T1 lesions in the subject population as a whole. We then developed an original algorithm to cluster adjacent lesional voxels (cluxels) in each subject and tested whether cluxel topology was significantly associated with any single-nucleotide polymorphism in our data set. To focus on patterns of lesion distribution, we computed the first 10 principal components. Although principal component 1 correlated with lesion load, none of the remaining orthogonal components correlated with any other known variable. We then conducted genome-wide association studies on each of these and found 31 significant associations (false discovery rate <0.01) with principal component 8, which represents a mode of variation of lesion topology in the population. The majority of the loci can be linked to genes related to immune cell function and to myelin and neural growth; some (SYK, MYT1L, TRAPPC9, SLITKR6 and RIC3) have been previously associated with the distribution of white matter lesions in multiple sclerosis. Finally, we used a bioinformatics approach to identify a network of 48 interacting proteins showing genetic associations (P < 0.01) with cluxel topology in multiple sclerosis. This network also contains proteins expressed in immune cells and is enriched in molecules expressed in the central nervous system that contribute to neural development and regeneration. Our results show how quantitative traits derived from brain magnetic resonance images of patients with multiple sclerosis can be used as dependent variables in a genome-wide association study. With the widespread availability of powerful computing and the availability of genotyped populations, integration of imaging and genetic data sets is likely to become a mainstream tool for understanding the complex biological processes of multiple sclerosis and other brain disorders. 
  [Mm]etastasis  PubMed  
 Pathological aggressiveness of prostatic carcinomas related to RNASEL R462Q allelic variants. Allelic variations in the HPC1/RNASEL gene, especially the R462Q single nucleotide polymorphism, have been associated with increased susceptibility to prostate cancer. Prior studies have suggested that HPC1 or R462Q associated tumors present with more aggressive clinical features. We assessed a series of men undergoing radical prostatectomy for clinical and pathological measures of tumor aggressiveness according to the RNASEL R462Q genotype.A prospective analysis of 232 men treated for prostate cancer with radical prostatectomy was performed. Disease aggressiveness at diagnosis was assessed by age at disease onset, biopsy Gleason score, clinical T stage and pretreatment prostate specific antigen. Tumor aggressiveness was assessed pathologically by tumor volume, extraprostatic extension, seminal vesicle involvement and lymph node   metastasis  . Clinical and pathological characteristics were then correlated with RNASEL genotype.Of the 232 men studied 104 (45%) were homozygous WT, 101 (43%) were heterozygous and 27 (12%) were homozygous for the R462Q variant, mirroring the distribution in the general population. No significant differences were seen between genotypes in age at disease onset, pretreatment characteristics or pathological features, as assessed by surgical grade and pathological stage. Tumors homozygous for the R462Q variant were of smaller volume than other genotypes (p = 0.02).This prospective study suggests that prostate cancer in patients with the R462Q allelic variant of the HPC1/RNASEL gene is not associated with more aggressive clinical or pathological features in radical prostatectomy specimens. 
 RNase L is a negative regulator of cell migration. RNase L is a regulated endoribonuclease that functions in the interferon antiviral response. Activation of RNase L by 2', 5'-oligoadenylates has been linked to apoptosis, autophagy and inflammation. Genetic studies have also suggested the possible involvement of the RNase L gene (RNASEL) on chromosome 1q25.3 in several types of cancer. Here we report that ablation of RNase L in human prostate cancer PC3 cells by CRISPR/Cas9 gene editing technology enhanced cell migration as determined both by transwell assays and scratch wound healing assays. In addition, RNase L knockdown by means of RNAi increased migration of PC3 and DU145 cells in response to either fibronectin or serum stimulation, as did homozygous disruption of the RNase L gene in mouse embryonic fibroblasts. Serum or fibronectin stimulation of focal adhesion kinase (FAK) autophosphorylation on tyrosine-397 was increased by either knockdown or ablation of RNase L. In contrast, a missense mutant RNase L (R667A) lacking catalytic activity failed to suppress cell migration in PC3 cells. However, a nuclease-inactive mutant mouse RNase L (W630A) was able to partially inhibit migration of mouse fibroblasts. Consistent with a role for the catalytic activity of RNase L, transfection of PC3 cells with the RNase L activator, 2', 5'-oligoadenylate, suppressed cell migration. RNase L knockdown in PC3 cells enhanced tumor growth and   metastasis   following implantation in the mouse prostate. Our results suggest that naturally occurring mutations in the RNase L gene might promote enhanced cell migration and   metastasis  . 
  GeneRIFs  
 Suggest that naturally occurring mutations in the RNase L gene might promote enhanced prostate cancer cell migration and   metastasis  . 
   
  
 MAP2 
 
  NCBI Gene  
  GeneRIF  
  Pubmed  
 
 
  Search Term  Rank  Fields  
  [Mm]igration  6  PubMed(3)  
  ECM  7  PubMed(1); GeneRIFs(1)  
  [Ee]xtracellular [Mm]atrix  8  PubMed(1)  
  [Ii]nvasive  10  PubMed(3); GeneRIFs(2)  
  [Mm]etastasis  12  PubMed(2); GeneRIFs(1)  
  [Cc]ell [Ii]nvasion  13  PubMed(1)  
 
 
 hide/show details for MAP2 
  [Mm]igration  PubMed  
 Involvement of microtubule-associated protein 2 (MAP2) in oral cancer cell motility: a novel biological function of MAP2 in non-neuronal cells. Microtubule-associated protein 2 (MAP2) has been better known for its well-defined role primarily in neurite outgrowth during neuronal development. However, the biological functions of MAP2 in non-neuronal cells, such as epithelial cells, remain largely unknown. In the present study, we sought to investigate the cellular functions of MAP2 by separately establishing stable expression of two MAP2 isoforms, MAP2A and MAP2C, in oral squamous cell carcinoma, Ca9-22. Ectopic expression of MAP2A or MAP2C results in microtubule bundling predominantly at the cell periphery. Remarkably, overexpression of MAP2A but not MAP2C significantly promotes   migration   of Ca9-22 cells, whereas knockdown of MAP2A expression by specific siRNA oligos dramatically decreases cell   migration   of HaCaT, an immortalized keratinocyte cell line with abundant endogenous MAP2A. Furthermore, by immunohistochemical studies, MAP2A was shown to highly and selectively express in invasive oral cancer tissues, consistent with its motility-promoting cellular function revealed through in vitro assays. Thus, our findings have not only identified a novel role of MAP2 in non-neuronal cells, but also provided the first implication of MAP2 in malignant oral cancer tissues. 
 Increased expression of MAP2 inhibits melanoma cell proliferation, invasion and tumor growth in vitro and in vivo. Malignant melanoma (MM) is characterized by aggressive metastasis and high mortality rate. Microtubule-associated proteins 2 (MAP2) is expressed abundantly in majority of melanocytic nevi and primary melanomas, but absent in metastatic melanomas. To determine whether MAP2 correlates with tumor progression of MM, we investigated the effects of MAP2 inhibition on the biological behaviour of metastatic melanoma in vitro and in vivo. Our results demonstrated that adenovirus-mediated MAP2 induced apoptotic cell death and cell cycle arrest in metastatic human and mouse melanoma cell lines in vitro, and substantially inhibited the growth of melanomas in nude mice in vivo. In addition, intracellular expression of MAP2 was found to induce the morphologic alteration, suppress the   migration   and invasion and affect the assembly, stabilization and bundling of microtubules in melanoma cells. This is the first study that MAP2 expression significantly inhibits the growth of MM in vivo. Our results suggest that MAP2 may serve as a promising molecular target for therapy and chemoprevention of MM in humans. 
 Plectin and IFAP-300K are homologous proteins binding to microtubule-associated proteins 1 and 2 and to the 240-kilodalton subunit of spectrin. Structural and functional characteristics of plectin from intermediate filament preparations of rat glioma C6 cells were compared to those of the intermediate filament-associated protein of Mr = 300,000 (IFAP-300K) of baby hamster kidney cells (Yang, H.-S., Lieska, N., Goldman, A.E., and Goldman, R.D. (1985) J. Cell Biol. 100, 620-631). After radiolabeling and proteolytic digestion under varied conditions, both proteins yielded nearly identical peptide maps. Immunological cross-reactivity, co-  migration   on one- and two-dimensional high-resolution gels, chromatofocusing, and amino acid analysis demonstrated structural homology as well. In vivo labeling with 32Pi showed that plectin was the target for cAMP-independent protein kinases which phosphorylated 18-kDa domains at the end(s) of the molecule. Previously reported phosphorylation sites for cAMP-dependent and a newly identified site for Ca2+/calmodulin-dependent protein kinases were located on different domains. In solid-phase binding assays, plectin bound to vimentin, microtubule-associated proteins 1 and 2, the 240-kDa chain of brain fodrin, and alpha-spectrin from human erythrocytes. Similar characteristics were revealed for corresponding 300-kDa components of various other cell lines, supporting the concept that plectin is a general cytoskeletal cross-linking element, probably of multiple function. 
  ECM  PubMed  
 Immunocytochemical expression of microtubule-associated protein-2 (MAP-2) in small cell lung cancer cell lines with neuronal-like processes. Five of six human small cell lung cancer (SCLC) cell lines changed morphologically into cells with neuronal-like processes on the extracellular matrix of human lung adenocarcinoma cell line PC-9 cells (PC-9 /   ECM   substrate). The features of the neuronal-like processes of these SCLC cell lines were examined immunocytochemically using monoclonal antibodies against beta-chains of tubulin and microtubule-associated protein-2 (MAP-2), which is somatodendritic MAP of neurons. It was observed that beta-chains of tubulin and MAP-2 were expressed along the neuronal-like processes of SCLC cell lines. These findings suggest that the beta-chains of tubulin and MAP-2 are expressed functionally in SCLC cell lines in association with the development of dendrite-like processes on PC-9 /   ECM   substrate. 
  GeneRIFs  
 beta-chains of tubulin and MAP-2 are expressed functionally in SCLC cell lines in association with the development of dendrite-like processes on PC-9 /   ECM   substrate 
  [Ee]xtracellular [Mm]atrix  PubMed  
 Immunocytochemical expression of microtubule-associated protein-2 (MAP-2) in small cell lung cancer cell lines with neuronal-like processes. Five of six human small cell lung cancer (SCLC) cell lines changed morphologically into cells with neuronal-like processes on the   extracellular matrix   of human lung adenocarcinoma cell line PC-9 cells (PC-9 / ECM substrate). The features of the neuronal-like processes of these SCLC cell lines were examined immunocytochemically using monoclonal antibodies against beta-chains of tubulin and microtubule-associated protein-2 (MAP-2), which is somatodendritic MAP of neurons. It was observed that beta-chains of tubulin and MAP-2 were expressed along the neuronal-like processes of SCLC cell lines. These findings suggest that the beta-chains of tubulin and MAP-2 are expressed functionally in SCLC cell lines in association with the development of dendrite-like processes on PC-9 / ECM substrate. 
  [Ii]nvasive  PubMed  
 Involvement of microtubule-associated protein 2 (MAP2) in oral cancer cell motility: a novel biological function of MAP2 in non-neuronal cells. Microtubule-associated protein 2 (MAP2) has been better known for its well-defined role primarily in neurite outgrowth during neuronal development. However, the biological functions of MAP2 in non-neuronal cells, such as epithelial cells, remain largely unknown. In the present study, we sought to investigate the cellular functions of MAP2 by separately establishing stable expression of two MAP2 isoforms, MAP2A and MAP2C, in oral squamous cell carcinoma, Ca9-22. Ectopic expression of MAP2A or MAP2C results in microtubule bundling predominantly at the cell periphery. Remarkably, overexpression of MAP2A but not MAP2C significantly promotes migration of Ca9-22 cells, whereas knockdown of MAP2A expression by specific siRNA oligos dramatically decreases cell migration of HaCaT, an immortalized keratinocyte cell line with abundant endogenous MAP2A. Furthermore, by immunohistochemical studies, MAP2A was shown to highly and selectively express in   invasive   oral cancer tissues, consistent with its motility-promoting cellular function revealed through in vitro assays. Thus, our findings have not only identified a novel role of MAP2 in non-neuronal cells, but also provided the first implication of MAP2 in malignant oral cancer tissues. 
 Specific induction of the high-molecular-weight microtubule-associated protein 2 (hmw-MAP2) by betel quid extract in cultured oral keratinocytes: clinical implications in betel quid-associated oral squamous cell carcinoma (OSCC). Betel quid (BQ) chewing, a popular habit in numerous Asian countries including India and Taiwan, has a strong correlation with an increased risk of oral squamous cell carcinoma (OSCC). While substantial efforts have been made to test the cytotoxic, genotoxic and mutagenic effects of BQ extract and its components, the disease mechanisms underlying BQ-induced oral carcinogenesis remain obscure. Here, we show that a neuronal protein, microtubule-associated protein 2 (MAP2), was induced by BQ extract in cultured normal human oral keratinocytes (NHOKs). Subsequent analyses demonstrated that such induction was more eminent and consistent in the high-molecular-weight isoform of MAP2 (hmw-MAP2) than that in its low-molecular-weight counterpart (lmw-MAP2). Furthermore, we analyzed expression of hmw-MAP2 protein in 88 oral specimens consisting of clinicopathologically pre-malignant (leukoplakia) and malignant (OSCC) lesions, along with their adjacent normal mucosa. Immunohistochemistry revealed that, with the exposure to BQ, the hmw-MAP2 was over-expressed in 41.2% (7/17) of OSCC, 11.2% (1/9) of leukoplakia and none (0/19) of normal mucosa. In contrast, expression of the hmw-MAP2 was barely detected in BQ-free OSCC. These results suggest a significant correlation between expression of the hmw-MAP2 and BQ-associated progression of oral carcinogenesis (P=0.0046). Interestingly, the hmw-MAP2 was found to preferentially express in histopathologically less differentiated OSCC (P=0.014); the percentages of positive staining in poorly, moderately and well differentiated OSCC were 62.5, 21.4 and 7.1%, respectively. However, BQ chewing appeared to have marginal correlation with such propensity. Finally, we show that the majority of hmw-MAP2-positive poorly differentiated lesions were also histopathologically   invasive  . Taken together, these findings suggest the possibility that the hmw-MAP2 may be a diagnostic marker for BQ-chewing lesions and a potential therapeutic target. To our knowledge, this study has provided the first clinical implication that closely links a cytoskeletal protein to BQ-associated oral cancer. 
 Transcriptional upregulation of microtubule-associated protein 2 is involved in the protein kinase A-induced decrease in the   invasive  ness of glioma cells. Malignant glioma is the most lethal primary tumor of the central nervous system, with notable cell invasion causing significant recurrence. Suppression of glioma invasion is very important for improving clinical outcomes. Drugs that directly disrupt the cytoskeleton have been developed for this purpose; however, drug resistance and unsatisfactory selectivity have limited their clinical use. Previously, we reported that protein kinase A (PKA, also known as cyclic-AMP dependent protein kinase) activation induced the differentiation of glioma cells.We used several small molecular inhibitors and RNA interference, combined with wound healing assays, Matrigel transwell assay, and microscopic observation, to determine whether activation of the PKA pathway could inhibit the invasion of human glioma cells.Activation of PKA decreased the invasion of glioma cells. The mechanism operated via transcriptional upregulation of microtubule-associated protein 2 (MAP2), which was activated by the PKA pathway and led to ossification of microtubule dynamics via polymerization of tubulin. This resulted in morphological changes and a reduction in glioma cell invasion. Furthermore, chromosome immunoprecipitation and quantitative real-time polymerase chain reaction showed that signal transducer and activator of transcription 3 (STAT3) is involved in the transcriptional upregulation of MAP2.Our findings suggested that PKA may represent a potential target for anti-invasion glioma therapy and that the downstream modulators (eg, STAT3/MAP2) partially mediate the effects of PKA. 
  GeneRIFs  
 Transcriptional upregulation of microtubule-associated protein 2 is involved in the protein kinase A-induced decrease in the   invasive  ness of glioma cells. 
 Increased MAP2 expression in malignant melanoma cells significantly inhibits tumor cell growth and neoplasm   invasive  ness, and induces apoptosis and cell cycle arrest. 
  [Mm]etastasis  PubMed  
 Increased expression of MAP2 inhibits melanoma cell proliferation, invasion and tumor growth in vitro and in vivo. Malignant melanoma (MM) is characterized by aggressive   metastasis   and high mortality rate. Microtubule-associated proteins 2 (MAP2) is expressed abundantly in majority of melanocytic nevi and primary melanomas, but absent in metastatic melanomas. To determine whether MAP2 correlates with tumor progression of MM, we investigated the effects of MAP2 inhibition on the biological behaviour of metastatic melanoma in vitro and in vivo. Our results demonstrated that adenovirus-mediated MAP2 induced apoptotic cell death and cell cycle arrest in metastatic human and mouse melanoma cell lines in vitro, and substantially inhibited the growth of melanomas in nude mice in vivo. In addition, intracellular expression of MAP2 was found to induce the morphologic alteration, suppress the migration and invasion and affect the assembly, stabilization and bundling of microtubules in melanoma cells. This is the first study that MAP2 expression significantly inhibits the growth of MM in vivo. Our results suggest that MAP2 may serve as a promising molecular target for therapy and chemoprevention of MM in humans. 
 Expression of SNCG, MAP2, SDF-1 and CXCR4 in gastric adenocarcinoma and their clinical significance. The purpose of the study was to detect the expression of SNCG, MAP2, SDF-1 and CXCR4 in gastric adenocarcinoma, and to evaluate their roles in the carcinogenesis of gastric adenocarcinoma, development, invasion and   metastasis   as well as their clinical significance.The expression of SNCG, MAP2, SDF-1 and CXCR4 was detected by SP immunohistochemical method in 225 cases of gastric adenocarcinoma and 105 cases of nonneoplastic adjacent gastric tissue. The expression of SNCG, MAP2, SDF-1 and CXCR4 mRNA was also detected by RT-PCR method in 50 cases of gastric adenocarcinoma and 30 cases of nonneoplastic adjacent gastric tissue.The expression of SNCG, MAP2, SDF-1 and CXCR4 in the gastric adenocarcinoma was remarkably higher than those in the nonneoplastic adjacent gastric tissue (P < 0.01); The positive expression of SNCG and MAP2 was correlated with the depth of tumor invasion and the   metastasis   of lymph nodes (P < 0.05), and that of SDF-1 and CXCR4 was correlated with the   metastasis   of lymph nodes (P < 0.05).SNCG, MAP2, SDF-1 and CXCR4 may play an important role in the carcinogenesis, progression, invasion and   metastasis   of gastric adenocarcinoma. However, it still needs more exploration whether they can serve as promising therapeutic targets of gastric adenocarcinoma. 
  GeneRIFs  
 SNCG, MAP2, SDF-1 and CXCR4 may play an important role in the carcinogenesis, progression, invasion and   metastasis   of gastric adenocarcinoma. 
  [Cc]ell [Ii]nvasion  PubMed  
 Transcriptional upregulation of microtubule-associated protein 2 is involved in the protein kinase A-induced decrease in the invasiveness of glioma cells. Malignant glioma is the most lethal primary tumor of the central nervous system, with notable   cell invasion   causing significant recurrence. Suppression of glioma invasion is very important for improving clinical outcomes. Drugs that directly disrupt the cytoskeleton have been developed for this purpose; however, drug resistance and unsatisfactory selectivity have limited their clinical use. Previously, we reported that protein kinase A (PKA, also known as cyclic-AMP dependent protein kinase) activation induced the differentiation of glioma cells.We used several small molecular inhibitors and RNA interference, combined with wound healing assays, Matrigel transwell assay, and microscopic observation, to determine whether activation of the PKA pathway could inhibit the invasion of human glioma cells.Activation of PKA decreased the invasion of glioma cells. The mechanism operated via transcriptional upregulation of microtubule-associated protein 2 (MAP2), which was activated by the PKA pathway and led to ossification of microtubule dynamics via polymerization of tubulin. This resulted in morphological changes and a reduction in glioma   cell invasion  . Furthermore, chromosome immunoprecipitation and quantitative real-time polymerase chain reaction showed that signal transducer and activator of transcription 3 (STAT3) is involved in the transcriptional upregulation of MAP2.Our findings suggested that PKA may represent a potential target for anti-invasion glioma therapy and that the downstream modulators (eg, STAT3/MAP2) partially mediate the effects of PKA. 
   
  
 MAP6 
 
  NCBI Gene  
  GeneRIF  
  Pubmed  
 
 
  Search Term  Rank  Fields  
  [Mm]igration  6  PubMed(1)  
 
 
 hide/show details for MAP6 
  [Mm]igration  PubMed  
 Stable tubule only polypeptides (STOP) proteins co-aggregate with spheroid neurofilaments in amyotrophic lateral sclerosis. A major cytopathological hallmark of amyotrophic lateral sclerosis (ALS) is the presence of axonal spheroids containing abnormally accumulated neurofilaments. The mechanism of their formation, their contribution to the disease, and the possibility of other co-aggregated components are still enigmatic. Here we analyze the composition of such lesions with special reference to stable tubule only polypeptide (STOP), a protein responsible for microtubule cold stabilization. In normal human brain and spinal cord, the distribution of STOP proteins is uniform between the cytoplasm and neurites of neurons. However, all the neurofilament-rich spheroids present in the tissues of affected patients are intensely labeled with 3 different anti-STOP antibodies. Moreover, when neurofilaments and microtubules are isolated from spinal cord and brain, STOP proteins are systematically co-purified with neurofilaments. By SDS-PAGE analysis, no alteration of the   migration   profile of STOP proteins is observed in pathological samples. Other microtubular proteins, like tubulin or kinesin, are inconstantly present in spheroids, suggesting that a microtubule destabilizing process may be involved in the pathogenesis of ALS. These results indicate that the selective co-aggregation of neurofilament and STOP proteins represent a new cytopathological marker for spheroids. 
   
  
 AKAP12 
 
  NCBI Gene  
  GeneRIF  
  Pubmed  
 
 
  Search Term  Rank  Fields  
  [Ff]ilopodia  1  PubMed(2); GeneRIFs(1)  
  [Pp]odosome(s)?  5  PubMed(1); GeneRIFs(1)  
  [Mm]igration  6  PubMed(6); GeneRIFs(2)  
  ECM  7  PubMed(1)  
  [Ee]xtracellular [Mm]atrix  8  PubMed(1)  
  [Ii]nvasive  10  PubMed(3)  
  [Mm]etastasis  12  PubMed(7); GeneRIFs(2)  
  [Cc]ell [Ii]nvasion  13  PubMed(1)  
 
 
 hide/show details for AKAP12 
  [Ff]ilopodia  PubMed  
 Suppression of chemotaxis by SSeCKS via scaffolding of phosphoinositol phosphates and the recruitment of the Cdc42 GEF, Frabin, to the leading edge. Chemotaxis is controlled by interactions between receptors, Rho-family GTPases, phosphatidylinositol 3-kinases, and cytoskeleton remodeling proteins. We investigated how the metastasis suppressor, SSeCKS, attenuates chemotaxis. Chemotaxis activity inversely correlated with SSeCKS levels in mouse embryo fibroblasts (MEF), DU145 and MDA-MB-231 cancer cells. SSeCKS loss induced chemotactic velocity and linear directionality, correlating with replacement of leading edge lamellipodia with fascin-enriched   filopodia  -like extensions, the formation of thickened longitudinal F-actin stress fibers reaching to   filopodia  l tips, relative enrichments at the leading edge of phosphatidylinositol (3,4,5)P3 (PIP3), Akt, PKC-ζ, Cdc42-GTP and active Src (SrcpoY416), and a loss of Rac1. Leading edge lamellipodia and chemotaxis inhibition in SSeCKS-null MEF could be restored by full-length SSeCKS or SSeCKS deleted of its Src-binding domain (ΔSrc), but not by SSeCKS deleted of its three MARCKS (myristylated alanine-rich C kinase substrate) polybasic domains (ΔPBD), which bind PIP2 and PIP3. The enrichment of activated Cdc42 in SSeCKS-null leading ed  ge filopo  dia correlated with recruitment of the Cdc42-specific guanine nucleotide exchange factor, Frabin, likely recruited via multiple PIP2/3-binding domains. Frabin knockdown in SSeCKS-null MEF restores leading edge lamellipodia and chemotaxis inhibition. However, SSeCKS failed to co-immunoprecipitate with Rac1, Cdc42 or Frabin. Consistent with the notion that chemotaxis is controlled by SSeCKS-PIP (vs. -Src) scaffolding activity, constitutively-active phosphatidylinositol 3-kinase could override the ability of the Src inhibitor, SKI-606, to suppress chemotaxis a  nd filopo  dial enrichment of Frabin in SSeCKS-null MEF. Our data suggest a role for SSeCKS in controlling Rac1 vs. Cdc42-induced cellular dynamics at the leading chemotactic edge through the scaffolding of phospholipids and signal mediators, and through the reorganization of the actin cytoskeleton controlling directional movement. 
 Gravin, an autoantigen recognized by serum from myasthenia gravis patients, is a kinase scaffold protein. Subcellular targeting of protein kinases and phosphatases provides a mechanism for co-localizing these enzymes with their preferred substrates. A recently identified mammalian scaffold protein, AKAP79, controls the location of two broad-specificity kinases and a phosphatase.We have identified and characterized another mammalian scaffold protein which coordinates the location of protein kinase A and protein kinase C. We isolated a cDNA encoding a 250 kDa A-skinase anchoring protein (AKAP) called gravin, which was originally identified as a cytoplasmic antigen recognized by myasthenia gravis sera. Sequence homology to proteins that are known to bind protein kinase C suggests that gravin also binds this kinase. Studies of binding in vitro show that residues 1526-1780 of gravin bind the regulatory subunit (RII) of protein kinase A with high affinity, and residues 265-556 bind protein kinase C. Gravin expression in human erythroleukemia cells can be induced with phorbol ester, resulting in the detection of a 250 kDa RII- and PKC-binding protein. Immunolocalization experiments show that gravin is concentrated at the cell periphery and is enriched in   filopodia  . Gravin staining is coincident with an AKAP detected by an in situ RII-overlay assay, and a PKA-gravin complex can be isolated from human erythroleukemia cells.We present biochemical evidence that gravin forms part of a signaling scaffold, and propose that protein kinases A and C may participate in the coordination of signal transduction events in the   filopodia   of human erythroleukemia cells. 
  GeneRIFs  
 The enrichment of activated Cdc42 in SSeCKS-null leading edge   filopodia   correlated with recruitment of the Cdc42-specific guanine nucleotide exchange factor, Frabin. 
  [Pp]odosome(s)?  PubMed  
 SSeCKS/Gravin/AKAP12 inhibits cancer cell invasiveness and chemotaxis by suppressing a protein kinase C- Raf/MEK/ERK pathway. SSeCKS/Gravin/AKAP12 ("SSeCKS") encodes a cytoskeletal protein that regulates G(1) --> S progression by scaffolding cyclins, protein kinase C (PKC) and PKA. SSeCKS is down-regulated in many tumor types including prostate, and when re-expressed in MAT-LyLu (MLL) prostate cancer cells, SSeCKS selectively inhibits metastasis by suppressing neovascularization at distal sites, correlating with its ability to down-regulate proangiogenic genes including Vegfa. However, the forced re-expression of VEGF only rescues partial lung metastasis formation. Here, we show that SSeCKS potently inhibits chemotaxis and Matrigel invasion, motility parameters contributing to metastasis formation. SSeCKS suppressed serum-induced activation of the Raf/MEK/ERK pathway, resulting in down-regulation of matrix metalloproteinase-2 expression. In contrast, SSeCKS had no effect on serum-induced phosphorylation of the Src substrate, Shc, in agreement with our previous data that SSeCKS does not inhibit Src kinase activity in cells. Invasiveness and chemotaxis could be restored by the forced expression of constitutively active MEK1, MEK2, ERK1, or PKCalpha. SSeCKS suppressed phorbol ester-induced ERK1/2 activity only if it encoded its PKC binding domain (amino acids 553-900), suggesting that SSeCKS attenuates ERK activation through a direct scaffolding of conventional and/or novel PKC isozymes. Finally, control of MLL invasiveness by SSeCKS is influenced by the actin cytoskeleton: the ability of SSeCKS to inhibit   podosome   formation is unaffected by cytochalasin D or jasplakinolide, whereas its ability to inhibit MEK1/2 and ERK1/2 activation is nullified by jasplakinolide. Our findings suggest that SSeCKS suppresses metastatic motility by disengaging activated Src and then inhibiting the PKC-Raf/MEK/ERK pathways controlling matrix metalloproteinase-2 expression and   podosome   formation. 
  GeneRIFs  
 Findings suggest that SSeCKS suppresses metastatic motility by disengaging activated Src and then inhibiting the PKC-Raf/MEK/ERK pathways controlling matrix metalloproteinase-2 expression and   podosome   formation. 
  [Mm]igration  PubMed  
 Analysis of the myosin-II-responsive focal adhesion proteome reveals a role for β-Pix in negative regulation of focal adhesion maturation. Focal adhesions undergo myosin-II-mediated maturation wherein they grow and change composition to modulate integrin signalling for cell   migratio  n, growth and differentiation. To determine how focal adhesion composition is affected by myosin II activity, we performed proteomic analysis of isolated focal adhesions and compared protein abundance in focal adhesions from cells with and without myosin II inhibition. We identified 905 focal adhesion proteins, 459 of which changed in abundance with myosin II inhibition, defining the myosin-II-responsive focal adhesion proteome. The abundance of 73% of the proteins in the myosin-II-responsive focal adhesion proteome was enhanced by contractility, including proteins involved in Rho-mediated focal adhesion maturation and endocytosis- and calpain-dependent focal adhesion disassembly. During myosin II inhibition, 27% of proteins in the myosin-II-responsive focal adhesion proteome, including proteins involved in Rac-mediated lamellipodial protrusion, were enriched in focal adhesions, establishing that focal adhesion protein recruitment is also negatively regulated by contractility. We focused on the Rac guanine nucleotide exchange factor β-Pix, documenting its role in the negative regulation of focal adhesion maturation and the promotion of lamellipodial protrusion and focal adhesion turnover to drive cel  l migrati  on. 
 The angiogenesis suppressor gene AKAP12 is under the epigenetic control of HDAC7 in endothelial cells. Histone deacetylases (HDACs) are a family of 18 enzymes that deacetylate lysine residues of both histone and nonhistone proteins and to a large extent govern the process of angiogenesis. Previous studies have shown that specific inhibition of HDAC7 blocks angiogenesis both in vitro and in vivo. However, the underlying molecular mechanisms are not fully understood and hence preclude any meaningful development of suitable therapeutic modalities. The goal of the present study was to further the understanding of HDAC7 epigenetic control of angiogenesis in human endothelial cells using the proteomic approach. The underlying problem was approached through siRNA-mediated gene-expression silencing of HDAC7 in human umbilical vein endothelial cells (HUVECs). To this end, HUVEC proteins were extracted and proteomically analyzed. The emphasis was placed on up-regulated proteins, as these may represent potential direct epigenetic targets of HDAC7. Among several proteins, A-kinase anchor protein 12 (AKAP12) was the most reproducibly up-regulated protein following HDAC7 depletion. This overexpression of AKAP12 was responsible for the inhibition of   migration   and tube formation in HDAC7-depleted HUVEC. Mechanistically, H3 histones associated with AKAP12 promoter were acetylated following the removal of HDAC7, leading to an increase in its mRNA and protein levels. AKAP12 is responsible for protein kinase C mediated phosphorylation of signal transducer and activator of transcription 3 (STAT3). Phosphorylated STAT3 increasingly binds to the chromatin and AKAP12 promoter and is necessary for maintaining the elevated levels of AKAP12 following HDAC7 knockdown. We demonstrated for the first time that AKAP12 tumor/angiogenesis suppressor gene is an epigenetic target of HDAC7, whose elevated levels lead to a negative regulation of HUVEC   migration   and inhibit formation of tube-like structures. 
 Hypoxic induction of AKAP12 variant 2 shifts PKA-mediated protein phosphorylation to enhance   migration   and metastasis of melanoma cells. Scaffold proteins are critical hubs within cells that have the ability to modulate upstream signaling molecules and their downstream effectors to fine-tune biological responses. Although they can serve as focal points for association of signaling molecules and downstream pathways that regulate tumorigenesis, little is known about how the tumor microenvironment affects the expression and activity of scaffold proteins. This study demonstrates that hypoxia, a common element of solid tumors harboring low oxygen levels, regulates expression of a specific variant of the scaffold protein AKAP12 (A-kinase anchor protein 12), AKAP12v2, in metastatic melanoma. In turn, through a kinome-wide phosphoproteomic and MS study, we demonstrate that this scaffolding protein regulates a shift in protein kinase A (PKA)-mediated phosphorylation events under hypoxia, causing alterations in tumor cell invasion and   migration   in vitro, as well as metastasis in an in vivo orthotopic model of melanoma. Mechanistically, the shift in AKAP12-dependent PKA-mediated phosphorylations under hypoxia is due to changes in AKAP12 localization vs. structural differences between its two variants. Importantly, our work defines a mechanism through which a scaffold protein can be regulated by the tumor microenvironment and further explains how a tumor cell can coordinate many critical signaling pathways that are essential for tumor growth through one individual scaffolding protein. 
 Inhibition of endothelial cell   migration   through the down‑regulation of MMP-9 by A-kinase anchoring protein 12. Matrix metalloproteinases (MMPs) play an important role in the degradation of extracellular matrix (ECM) molecules. ECM degradation is associated with tumor metastasis and angiogenesis. Therefore, the regulation of MMPs is of potential benefit in the treatment of various diseases, including cancer. A-kinase anchoring protein 12 (AKAP12) has been identified as a potential tumor suppressor. However, the function of AKAP12 as a tumor suppressor is not well understood. Herein, to determine the relationship between AKAP12 and MMP-9 in cancer, we first investigated the expression of MMP-9 under normoxic and hypoxic conditions in human fibrosarcoma cells. The expression of MMP-9 was not detected under normoxic conditions.; however, it was markedly increased under hypoxia in HT1080 cells. The effect of AKAP12 on the expression of MMP-9 was subsequently investigated. Hypoxia-induced MMP-9 mRNA expression was significantly reduced by overexpression of AKAP12, as was MMP-9 protein expression. In addition, when the AKAP12 transfectant-conditioned media (CM) were transferred into human endothelial cells, cel  l migrati  on was significantly inhibited compared to the control group. Notably, the inhibition of AKAP12 expression by siRNA targeting AKAP12 resulted in an increase in the expression of active MMP-2 under normoxia, as well as of MMP-9. Endothelial cel  l migrati  on was also strongly increased by treatment with CM of siRNA against AKAP12, as compared to the control group. Taken together, the results indicate that AKAP12 is involved in the regulation of endothelial cel  l migrati  on through the inhibitory regulation of MMP-9 expression in tumor cells. 
 Adhesion-mediated cytoskeletal remodeling is controlled by the direct scaffolding of Src from FAK complexes to lipid rafts by SSeCKS/AKAP12. Metastatic cell   migration   and invasion are regulated by altered adhesion-mediated signaling to the actin-based cytoskeleton via activated Src-FAK complexes. Src-suppressed C-kinase substrate (SSeCKS, the rodent orthologue of human Gravin/AKAP12), whose expression is downregulated by oncogenic Src and in many human cancers, antagonizes oncogenic Src pathways including those driving neovascularization at metastatic sites, metastatic cell motility and invasiveness. This is likely manifested through its function as a scaffolder of F-actin and signaling proteins such as cyclins, calmodulin, protein kinase C and A. Here we show that in contrast to its ability to inhibit haptotaxis, SSeCKS increased prostate cancer cell adhesion to fibronectin and type I collagen in a FAK-dependent manner, correlating with a relative increase in FAK(poY397) levels. In contrast, SSeCKS suppressed adhesion-induced Src activation (Src(poY416)) and phosphorylation of FAK at Y925, a known Src substrate site. SSeCKS also induced increased cell spreading, cell flattening, integrin β1 clustering and formation of mature focal adhesion plaques. An in silico analysis identified a Src-binding domain on SSeCKS(aa 153-166) that is homologous to the Src-binding domain of caveolin-1, and this region is required for SSeCKS-Src interaction, for SSeCKS-enhanced Src activity and sequestration to lipid rafts and for SSeCKS-enhanced adhesion of MAT-LyLu and CWR22Rv1 prostate cancer cells. Our data suggest a model in which SSeCKS suppresses oncogenic motility by sequestering Src to caveolin-rich lipid rafts, thereby disengaging Src from FAK-associated adhesion and signaling complexes. 
 Re-expression of AKAP12 inhibits progression and metastasis potential of colorectal carcinoma in vivo and in vitro. AKAP12/Gravin (A kinase anchor protein 12) is one of the A-kinase scaffold proteins and a potential tumor suppressor gene in human primary cancers. Our recent study demonstrated the highly recurrent loss of AKAP12 in colorectal cancer and AKAP12 reexpression inhibited proliferation and anchorage-independent growth in colorectal cancer cells, implicating AKAP12 in colorectal cancer pathogenesis.To evaluate the effect of this gene on the progression and metastasis of colorectal cancer, we examined the impact of overexpressing AKAP12 in the AKAP12-negative human colorectal cancer cell line LoVo, the single clone (LoVo-AKAP12) compared to mock-transfected cells (LoVo-CON).pCMV6-AKAP12-mediated AKAP12 re-expression induced apoptosis (3% to 12.7%, p<0.01),   migration   (89.6±7.5 cells to 31.0±4.1 cells, p<0.01) and invasion (82.7±5.2 cells to 24.7±3.3 cells, p<0.01) of LoVo cells in vitro compared to control cells. Nude mice injected with LoVo-AKAP12 cells had both significantly reduced tumor volume (p<0.01) and increased apoptosis compared to mice given AKAP12-CON. The quantitative human-specific Alu PCR analysis showed overexpression of AKAP12 suppressed the number of intravasated cells in vivo (p<0.01).These results demonstrate that AKAP12 may play an important role in tumor growth suppression and the survival of human colorectal cancer. 
  GeneRIFs  
 AKAP12 variant 2 hypoxic induction shifts PKA-mediated protein phosphorylation to enhance   migration   and metastasis of melanoma cells 
 AKAP12 is involved in the regulation of endothelial cell   migration   through the inhibitory regulation of MMP-9 expression in tumor cells. 
  ECM  PubMed  
 Inhibition of endothelial cell migration through the down‑regulation of MMP-9 by A-kinase anchoring protein 12. Matrix metalloproteinases (MMPs) play an important role in the degradation of extracellular matrix   (E  CM) molecules  . E  CM degradation is associated with tumor metastasis and angiogenesis. Therefore, the regulation of MMPs is of potential benefit in the treatment of various diseases, including cancer. A-kinase anchoring protein 12 (AKAP12) has been identified as a potential tumor suppressor. However, the function of AKAP12 as a tumor suppressor is not well understood. Herein, to determine the relationship between AKAP12 and MMP-9 in cancer, we first investigated the expression of MMP-9 under normoxic and hypoxic conditions in human fibrosarcoma cells. The expression of MMP-9 was not detected under normoxic conditions.; however, it was markedly increased under hypoxia in HT1080 cells. The effect of AKAP12 on the expression of MMP-9 was subsequently investigated. Hypoxia-induced MMP-9 mRNA expression was significantly reduced by overexpression of AKAP12, as was MMP-9 protein expression. In addition, when the AKAP12 transfectant-conditioned media (CM) were transferred into human endothelial cells, cell migration was significantly inhibited compared to the control group. Notably, the inhibition of AKAP12 expression by siRNA targeting AKAP12 resulted in an increase in the expression of active MMP-2 under normoxia, as well as of MMP-9. Endothelial cell migration was also strongly increased by treatment with CM of siRNA against AKAP12, as compared to the control group. Taken together, the results indicate that AKAP12 is involved in the regulation of endothelial cell migration through the inhibitory regulation of MMP-9 expression in tumor cells. 
  [Ee]xtracellular [Mm]atrix  PubMed  
 Inhibition of endothelial cell migration through the down‑regulation of MMP-9 by A-kinase anchoring protein 12. Matrix metalloproteinases (MMPs) play an important role in the degradation o  f extracellular matr  ix (ECM) molecules. ECM degradation is associated with tumor metastasis and angiogenesis. Therefore, the regulation of MMPs is of potential benefit in the treatment of various diseases, including cancer. A-kinase anchoring protein 12 (AKAP12) has been identified as a potential tumor suppressor. However, the function of AKAP12 as a tumor suppressor is not well understood. Herein, to determine the relationship between AKAP12 and MMP-9 in cancer, we first investigated the expression of MMP-9 under normoxic and hypoxic conditions in human fibrosarcoma cells. The expression of MMP-9 was not detected under normoxic conditions.; however, it was markedly increased under hypoxia in HT1080 cells. The effect of AKAP12 on the expression of MMP-9 was subsequently investigated. Hypoxia-induced MMP-9 mRNA expression was significantly reduced by overexpression of AKAP12, as was MMP-9 protein expression. In addition, when the AKAP12 transfectant-conditioned media (CM) were transferred into human endothelial cells, cell migration was significantly inhibited compared to the control group. Notably, the inhibition of AKAP12 expression by siRNA targeting AKAP12 resulted in an increase in the expression of active MMP-2 under normoxia, as well as of MMP-9. Endothelial cell migration was also strongly increased by treatment with CM of siRNA against AKAP12, as compared to the control group. Taken together, the results indicate that AKAP12 is involved in the regulation of endothelial cell migration through the inhibitory regulation of MMP-9 expression in tumor cells. 
  [Ii]nvasive  PubMed  
 SERPINB5 and AKAP12 - expression and promoter methylation of metastasis suppressor genes in pancreatic ductal adenocarcinoma. Early metastasis and infiltration are survival limiting characteristics of pancreatic ductal adenocarcinoma (PDAC). Thus, PDAC is likely to harbor alterations in metastasis suppressor genes that may provide novel diagnostic and therapeutic opportunities. This study investigates a panel of metastasis suppressor genes in correlation to PDAC phenotype and examines promoter methylation for regulatory influence on metastasis suppressor gene expression and for its potential as a diagnostic tool.Metastatic and   invasive   potential of 16 PDAC cell lines were quantified in an orthotopic mouse model and mRNA expression of 11 metastasis suppressor genes determined by quantitative RT-PCR. Analysis for promoter methylation was performed using methylation specific PCR and bisulfite sequencing PCR. Protein expression was determined by Western blot.In general, higher metastasis suppressor gene mRNA expression was not consistent with less aggressive phenotypes of PDAC. Instead, mRNA overexpression of several metastasis suppressor genes was found in PDAC cell lines vs. normal pancreatic RNA. Of the investigated metastasis suppressor genes, only higher AKAP12 mRNA expression was correlated with decreased metastasis (P < 0.05) and invasion scores (P < 0.01) while higher SERPINB5 mRNA expression was correlated with increased metastasis scores (P < 0.05). Both genes' promoters showed methylation, but only increased SERPINB5 methylation was associated with loss of mRNA and protein expression (P < 0.05). SERPINB5 methylation was also directly correlated to decreased metastasis scores (P < 0.05).AKAP12 mRNA expression was correlated to attenuated   invasive   and metastatic potential and may be associated with less aggressive phenotypes of PDAC while no such evidence was obtained for the remaining metastasis suppressor genes. Increased SERPINB5 mRNA expression was correlated to increased metastasis and mRNA expression was regulated by methylation. Thus, SERPINB5 methylation was directly correlated to metastasis scores and may provide a diagnostic tool for PDAC. 
 Adhesion-mediated cytoskeletal remodeling is controlled by the direct scaffolding of Src from FAK complexes to lipid rafts by SSeCKS/AKAP12. Metastatic cell migration and invasion are regulated by altered adhesion-mediated signaling to the actin-based cytoskeleton via activated Src-FAK complexes. Src-suppressed C-kinase substrate (SSeCKS, the rodent orthologue of human Gravin/AKAP12), whose expression is downregulated by oncogenic Src and in many human cancers, antagonizes oncogenic Src pathways including those driving neovascularization at metastatic sites, metastatic cell motility and   invasive  ness. This is likely manifested through its function as a scaffolder of F-actin and signaling proteins such as cyclins, calmodulin, protein kinase C and A. Here we show that in contrast to its ability to inhibit haptotaxis, SSeCKS increased prostate cancer cell adhesion to fibronectin and type I collagen in a FAK-dependent manner, correlating with a relative increase in FAK(poY397) levels. In contrast, SSeCKS suppressed adhesion-induced Src activation (Src(poY416)) and phosphorylation of FAK at Y925, a known Src substrate site. SSeCKS also induced increased cell spreading, cell flattening, integrin β1 clustering and formation of mature focal adhesion plaques. An in silico analysis identified a Src-binding domain on SSeCKS(aa 153-166) that is homologous to the Src-binding domain of caveolin-1, and this region is required for SSeCKS-Src interaction, for SSeCKS-enhanced Src activity and sequestration to lipid rafts and for SSeCKS-enhanced adhesion of MAT-LyLu and CWR22Rv1 prostate cancer cells. Our data suggest a model in which SSeCKS suppresses oncogenic motility by sequestering Src to caveolin-rich lipid rafts, thereby disengaging Src from FAK-associated adhesion and signaling complexes. 
 SSeCKS/Gravin/AKAP12 inhibits cancer cell   invasive  ness and chemotaxis by suppressing a protein kinase C- Raf/MEK/ERK pathway. SSeCKS/Gravin/AKAP12 ("SSeCKS") encodes a cytoskeletal protein that regulates G(1) --> S progression by scaffolding cyclins, protein kinase C (PKC) and PKA. SSeCKS is down-regulated in many tumor types including prostate, and when re-expressed in MAT-LyLu (MLL) prostate cancer cells, SSeCKS selectively inhibits metastasis by suppressing neovascularization at distal sites, correlating with its ability to down-regulate proangiogenic genes including Vegfa. However, the forced re-expression of VEGF only rescues partial lung metastasis formation. Here, we show that SSeCKS potently inhibits chemotaxis and Matrigel invasion, motility parameters contributing to metastasis formation. SSeCKS suppressed serum-induced activation of the Raf/MEK/ERK pathway, resulting in down-regulation of matrix metalloproteinase-2 expression. In contrast, SSeCKS had no effect on serum-induced phosphorylation of the Src substrate, Shc, in agreement with our previous data that SSeCKS does not inhibit Src kinase activity in cells.   Invasive  ness and chemotaxis could be restored by the forced expression of constitutively active MEK1, MEK2, ERK1, or PKCalpha. SSeCKS suppressed phorbol ester-induced ERK1/2 activity only if it encoded its PKC binding domain (amino acids 553-900), suggesting that SSeCKS attenuates ERK activation through a direct scaffolding of conventional and/or novel PKC isozymes. Finally, control of MLL   invasive  ness by SSeCKS is influenced by the actin cytoskeleton: the ability of SSeCKS to inhibit podosome formation is unaffected by cytochalasin D or jasplakinolide, whereas its ability to inhibit MEK1/2 and ERK1/2 activation is nullified by jasplakinolide. Our findings suggest that SSeCKS suppresses metastatic motility by disengaging activated Src and then inhibiting the PKC-Raf/MEK/ERK pathways controlling matrix metalloproteinase-2 expression and podosome formation. 
  [Mm]etastasis  PubMed  
 Suppression of chemotaxis by SSeCKS via scaffolding of phosphoinositol phosphates and the recruitment of the Cdc42 GEF, Frabin, to the leading edge. Chemotaxis is controlled by interactions between receptors, Rho-family GTPases, phosphatidylinositol 3-kinases, and cytoskeleton remodeling proteins. We investigated how the   metastasis   suppressor, SSeCKS, attenuates chemotaxis. Chemotaxis activity inversely correlated with SSeCKS levels in mouse embryo fibroblasts (MEF), DU145 and MDA-MB-231 cancer cells. SSeCKS loss induced chemotactic velocity and linear directionality, correlating with replacement of leading edge lamellipodia with fascin-enriched filopodia-like extensions, the formation of thickened longitudinal F-actin stress fibers reaching to filopodial tips, relative enrichments at the leading edge of phosphatidylinositol (3,4,5)P3 (PIP3), Akt, PKC-ζ, Cdc42-GTP and active Src (SrcpoY416), and a loss of Rac1. Leading edge lamellipodia and chemotaxis inhibition in SSeCKS-null MEF could be restored by full-length SSeCKS or SSeCKS deleted of its Src-binding domain (ΔSrc), but not by SSeCKS deleted of its three MARCKS (myristylated alanine-rich C kinase substrate) polybasic domains (ΔPBD), which bind PIP2 and PIP3. The enrichment of activated Cdc42 in SSeCKS-null leading edge filopodia correlated with recruitment of the Cdc42-specific guanine nucleotide exchange factor, Frabin, likely recruited via multiple PIP2/3-binding domains. Frabin knockdown in SSeCKS-null MEF restores leading edge lamellipodia and chemotaxis inhibition. However, SSeCKS failed to co-immunoprecipitate with Rac1, Cdc42 or Frabin. Consistent with the notion that chemotaxis is controlled by SSeCKS-PIP (vs. -Src) scaffolding activity, constitutively-active phosphatidylinositol 3-kinase could override the ability of the Src inhibitor, SKI-606, to suppress chemotaxis and filopodial enrichment of Frabin in SSeCKS-null MEF. Our data suggest a role for SSeCKS in controlling Rac1 vs. Cdc42-induced cellular dynamics at the leading chemotactic edge through the scaffolding of phospholipids and signal mediators, and through the reorganization of the actin cytoskeleton controlling directional movement. 
 SERPINB5 and AKAP12 - expression and promoter methylation of   metastasis   suppressor genes in pancreatic ductal adenocarcinoma. Early   metastasis   and infiltration are survival limiting characteristics of pancreatic ductal adenocarcinoma (PDAC). Thus, PDAC is likely to harbor alterations in   metastasis   suppressor genes that may provide novel diagnostic and therapeutic opportunities. This study investigates a panel of   metastasis   suppressor genes in correlation to PDAC phenotype and examines promoter methylation for regulatory influence on   metastasis   suppressor gene expression and for its potential as a diagnostic tool.Metastatic and invasive potential of 16 PDAC cell lines were quantified in an orthotopic mouse model and mRNA expression of 11   metastasis   suppressor genes determined by quantitative RT-PCR. Analysis for promoter methylation was performed using methylation specific PCR and bisulfite sequencing PCR. Protein expression was determined by Western blot.In general, higher   metastasis   suppressor gene mRNA expression was not consistent with less aggressive phenotypes of PDAC. Instead, mRNA overexpression of several   metastasis   suppressor genes was found in PDAC cell lines vs. normal pancreatic RNA. Of the investigated   metastasis   suppressor genes, only higher AKAP12 mRNA expression was correlated with decreased   metastasis   (P < 0.05) and invasion scores (P < 0.01) while higher SERPINB5 mRNA expression was correlated with increased   metastasis   scores (P < 0.05). Both genes' promoters showed methylation, but only increased SERPINB5 methylation was associated with loss of mRNA and protein expression (P < 0.05). SERPINB5 methylation was also directly correlated to decreased   metastasis   scores (P < 0.05).AKAP12 mRNA expression was correlated to attenuated invasive and metastatic potential and may be associated with less aggressive phenotypes of PDAC while no such evidence was obtained for the remaining   metastasis   suppressor genes. Increased SERPINB5 mRNA expression was correlated to increased   metastasis   and mRNA expression was regulated by methylation. Thus, SERPINB5 methylation was directly correlated to   metastasis   scores and may provide a diagnostic tool for PDAC. 
 Hypoxic induction of AKAP12 variant 2 shifts PKA-mediated protein phosphorylation to enhance migration and   metastasis   of melanoma cells. Scaffold proteins are critical hubs within cells that have the ability to modulate upstream signaling molecules and their downstream effectors to fine-tune biological responses. Although they can serve as focal points for association of signaling molecules and downstream pathways that regulate tumorigenesis, little is known about how the tumor microenvironment affects the expression and activity of scaffold proteins. This study demonstrates that hypoxia, a common element of solid tumors harboring low oxygen levels, regulates expression of a specific variant of the scaffold protein AKAP12 (A-kinase anchor protein 12), AKAP12v2, in metastatic melanoma. In turn, through a kinome-wide phosphoproteomic and MS study, we demonstrate that this scaffolding protein regulates a shift in protein kinase A (PKA)-mediated phosphorylation events under hypoxia, causing alterations in tumor cell invasion and migration in vitro, as well as   metastasis   in an in vivo orthotopic model of melanoma. Mechanistically, the shift in AKAP12-dependent PKA-mediated phosphorylations under hypoxia is due to changes in AKAP12 localization vs. structural differences between its two variants. Importantly, our work defines a mechanism through which a scaffold protein can be regulated by the tumor microenvironment and further explains how a tumor cell can coordinate many critical signaling pathways that are essential for tumor growth through one individual scaffolding protein. 
 Control of protein kinase C activity, phorbol ester-induced cytoskeletal remodeling, and cell survival signals by the scaffolding protein SSeCKS/GRAVIN/AKAP12. The product of the SSeCKS/GRAVIN/AKAP12 gene ("SSeCKS") is a major protein kinase (PK) C substrate that exhibits tumor- and   metastasis  -suppressing activity likely through its ability to scaffold multiple signaling mediators such as PKC, PKA, cyclins, calmodulin, and Src. Although SSeCKS and PKCα bind phosphatidylserine, we demonstrate that phosphatidylserine-independent binding of PKC by SSeCKS is facilitated by two homologous SSeCKS motifs, EG(I/V)(T/S)XWXSFK(K/R)(M/L)VTP(K/R)K(K/R)X(K/R)XXXEXXXE(E/D) (amino acids 592-620 and 741-769). SSeCKS binding to PKCα decreased kinase activity and was dependent on the two PKC-binding motifs. SSeCKS scaffolding of PKC was increased in confluent cell cultures, correlating with significantly increased SSeCKS protein levels and decreased PKCα activity, suggesting a role for SSeCKS in suppressing PKC activation during contact inhibition. SSeCKS-null mouse embryo fibroblasts displayed increased relative basal and phorbol ester (phorbol 12-myristate 13-acetate)-induced PKC activity but were defective in phorbol 12-myristate 13-acetate-induced actin cytoskeletal reorganization and cell shape change; these responses could be rescued by the forced expression of full-length SSeCKS but not by an SSeCKS variant deleted of its PKC-binding domains. Finally, the PKC binding sites in SSeCKS were required to restore cell rounding and/or decreased apoptosis in phorbol ester-treated LNCaP, LNCaP-C4-2, and MAT-LyLu prostate cancer cells. Thus, PKC-mediated remodeling of the actin cytoskeleton is likely regulated by the ability of SSeCKS to control PKC signaling and activity through a direct scaffolding function. 
 Inhibition of endothelial cell migration through the down‑regulation of MMP-9 by A-kinase anchoring protein 12. Matrix metalloproteinases (MMPs) play an important role in the degradation of extracellular matrix (ECM) molecules. ECM degradation is associated with tumo  r metastas  is and angiogenesis. Therefore, the regulation of MMPs is of potential benefit in the treatment of various diseases, including cancer. A-kinase anchoring protein 12 (AKAP12) has been identified as a potential tumor suppressor. However, the function of AKAP12 as a tumor suppressor is not well understood. Herein, to determine the relationship between AKAP12 and MMP-9 in cancer, we first investigated the expression of MMP-9 under normoxic and hypoxic conditions in human fibrosarcoma cells. The expression of MMP-9 was not detected under normoxic conditions.; however, it was markedly increased under hypoxia in HT1080 cells. The effect of AKAP12 on the expression of MMP-9 was subsequently investigated. Hypoxia-induced MMP-9 mRNA expression was significantly reduced by overexpression of AKAP12, as was MMP-9 protein expression. In addition, when the AKAP12 transfectant-conditioned media (CM) were transferred into human endothelial cells, cell migration was significantly inhibited compared to the control group. Notably, the inhibition of AKAP12 expression by siRNA targeting AKAP12 resulted in an increase in the expression of active MMP-2 under normoxia, as well as of MMP-9. Endothelial cell migration was also strongly increased by treatment with CM of siRNA against AKAP12, as compared to the control group. Taken together, the results indicate that AKAP12 is involved in the regulation of endothelial cell migration through the inhibitory regulation of MMP-9 expression in tumor cells. 
 Re-expression of AKAP12 inhibits progression and   metastasis   potential of colorectal carcinoma in vivo and in vitro. AKAP12/Gravin (A kinase anchor protein 12) is one of the A-kinase scaffold proteins and a potential tumor suppressor gene in human primary cancers. Our recent study demonstrated the highly recurrent loss of AKAP12 in colorectal cancer and AKAP12 reexpression inhibited proliferation and anchorage-independent growth in colorectal cancer cells, implicating AKAP12 in colorectal cancer pathogenesis.To evaluate the effect of this gene on the progression and   metastasis   of colorectal cancer, we examined the impact of overexpressing AKAP12 in the AKAP12-negative human colorectal cancer cell line LoVo, the single clone (LoVo-AKAP12) compared to mock-transfected cells (LoVo-CON).pCMV6-AKAP12-mediated AKAP12 re-expression induced apoptosis (3% to 12.7%, p<0.01), migration (89.6±7.5 cells to 31.0±4.1 cells, p<0.01) and invasion (82.7±5.2 cells to 24.7±3.3 cells, p<0.01) of LoVo cells in vitro compared to control cells. Nude mice injected with LoVo-AKAP12 cells had both significantly reduced tumor volume (p<0.01) and increased apoptosis compared to mice given AKAP12-CON. The quantitative human-specific Alu PCR analysis showed overexpression of AKAP12 suppressed the number of intravasated cells in vivo (p<0.01).These results demonstrate that AKAP12 may play an important role in tumor growth suppression and the survival of human colorectal cancer. 
 SSeCKS/Gravin/AKAP12 inhibits cancer cell invasiveness and chemotaxis by suppressing a protein kinase C- Raf/MEK/ERK pathway. SSeCKS/Gravin/AKAP12 ("SSeCKS") encodes a cytoskeletal protein that regulates G(1) --> S progression by scaffolding cyclins, protein kinase C (PKC) and PKA. SSeCKS is down-regulated in many tumor types including prostate, and when re-expressed in MAT-LyLu (MLL) prostate cancer cells, SSeCKS selectively inhibits   metastasis   by suppressing neovascularization at distal sites, correlating with its ability to down-regulate proangiogenic genes including Vegfa. However, the forced re-expression of VEGF only rescues partial lung   metastasis   formation. Here, we show that SSeCKS potently inhibits chemotaxis and Matrigel invasion, motility parameters contributing to   metastasis   formation. SSeCKS suppressed serum-induced activation of the Raf/MEK/ERK pathway, resulting in down-regulation of matrix metalloproteinase-2 expression. In contrast, SSeCKS had no effect on serum-induced phosphorylation of the Src substrate, Shc, in agreement with our previous data that SSeCKS does not inhibit Src kinase activity in cells. Invasiveness and chemotaxis could be restored by the forced expression of constitutively active MEK1, MEK2, ERK1, or PKCalpha. SSeCKS suppressed phorbol ester-induced ERK1/2 activity only if it encoded its PKC binding domain (amino acids 553-900), suggesting that SSeCKS attenuates ERK activation through a direct scaffolding of conventional and/or novel PKC isozymes. Finally, control of MLL invasiveness by SSeCKS is influenced by the actin cytoskeleton: the ability of SSeCKS to inhibit podosome formation is unaffected by cytochalasin D or jasplakinolide, whereas its ability to inhibit MEK1/2 and ERK1/2 activation is nullified by jasplakinolide. Our findings suggest that SSeCKS suppresses metastatic motility by disengaging activated Src and then inhibiting the PKC-Raf/MEK/ERK pathways controlling matrix metalloproteinase-2 expression and podosome formation. 
  GeneRIFs  
 AKAP12 variant 2 hypoxic induction shifts PKA-mediated protein phosphorylation to enhance migration and   metastasis   of melanoma cells 
 SERPINB5 and AKAP12 may have a role in increased   metastasis   in pancreatic ductal adenocarcinoma 
  [Cc]ell [Ii]nvasion  PubMed  
 Hypoxic induction of AKAP12 variant 2 shifts PKA-mediated protein phosphorylation to enhance migration and metastasis of melanoma cells. Scaffold proteins are critical hubs within cells that have the ability to modulate upstream signaling molecules and their downstream effectors to fine-tune biological responses. Although they can serve as focal points for association of signaling molecules and downstream pathways that regulate tumorigenesis, little is known about how the tumor microenvironment affects the expression and activity of scaffold proteins. This study demonstrates that hypoxia, a common element of solid tumors harboring low oxygen levels, regulates expression of a specific variant of the scaffold protein AKAP12 (A-kinase anchor protein 12), AKAP12v2, in metastatic melanoma. In turn, through a kinome-wide phosphoproteomic and MS study, we demonstrate that this scaffolding protein regulates a shift in protein kinase A (PKA)-mediated phosphorylation events under hypoxia, causing alterations in tumor   cell invasion   and migration in vitro, as well as metastasis in an in vivo orthotopic model of melanoma. Mechanistically, the shift in AKAP12-dependent PKA-mediated phosphorylations under hypoxia is due to changes in AKAP12 localization vs. structural differences between its two variants. Importantly, our work defines a mechanism through which a scaffold protein can be regulated by the tumor microenvironment and further explains how a tumor cell can coordinate many critical signaling pathways that are essential for tumor growth through one individual scaffolding protein. 
   
  
 RTN4 
 
  NCBI Gene  
  GeneRIF  
  Pubmed  
 
 
  Search Term  Rank  Fields  
  [Ff]ilopodia  1  PubMed(1)  
  [Mm]igration  6  Process(2); PubMed(10); GeneRIFs(3)  
  [Ee]xtracellular [Mm]atrix  8  PubMed(2); GeneRIFs(1)  
  [Ii]nvasive  10  PubMed(1)  
  [Mm]etastasis  12  PubMed(2)  
 
 
 hide/show details for RTN4 
  [Ff]ilopodia  PubMed  
 Dendritic spine alterations in neocortical pyramidal neurons following postnatal neuronal Nogo-A knockdown. The myelin-associated protein Nogo-A is a well-known inhibitor of axonal regeneration and compensatory plasticity, yet functions of neuronal Nogo-A are not as clear. The present study examined the effects of decreased levels of neuronal Nogo-A on dendritic spines of developing neocortical neurons. Decreased Nogo-A levels in these neurons resulted in lowered spine density and an increase in   filopodia  l type protrusions. These results suggest a role for neuronal Nogo-A in maintaining a spine phenotype in neocortical pyramidal cells. 
  [Mm]igration  Process  
 cerebral cortex radial glia guided   migration   
 positive regulation of epithelial cell   migration   
  PubMed  
 Nogo-B regulates   migration   and contraction of airway smooth muscle cells by decreasing ARPC 2/3 and increasing MYL-9 expression. Abnormal proliferation, apoptosis,   migration   and contraction of airway smooth muscle (ASM) cells in airway remodeling in asthma are basically excessive repair responses to a network of inflammatory mediators such as PDGF, but the mechanisms of such responses remain unclear. Nogo-B, a member of the reticulum family 4(RTN4), is known to play a key role in arteriogenesis and tissue repair. Further studies are needed to elucidate the role of Nogo-B in airway smooth muscle abnormalities.A mouse model of chronic asthma was established by repeated OVA inhalation and subjected to Nogo-B expression analysis using immunohistochemistry and Western Blotting. Then, primary human bronchial smooth muscle cells (HBSMCs) were cultured in vitro and a siRNA interference was performed to knockdown the expression of Nogo-B in the cells. The effects of Nogo-B inhibition on PDGF-induced HBSMCs proliferation,   migration   and contraction were evaluated. Finally, a proteomic analysis was conducted to unveil the underlying mechanisms responsible for the function of Nogo-B.Total Nogo-B expression was approximately 3.08-fold lower in chronic asthmatic mice compared to naïve mice, which was obvious in the smooth muscle layer of the airways. Interference of Nogo-B expression by siRNA resulted nearly 96% reduction in mRNA in cultured HBSMCs. In addition, knockdown of Nogo-B using specific siRNA significantly decreased PDGF-induced   migratio  n of HBSMCs by 2.3-fold, and increased the cellular contraction by 16% compared to negative controls, but had limited effects on PDGF-induced proliferation. Furthermore, using proteomic analysis, we demonstrate that the expression of actin related protein 2/3 complex subunit 5 (ARPC 2/3) decreased and, myosin regulatory light chain 9 isoform a (MYL-9) increased after Nogo-B knockdown.These data define a novel role for Nogo-B in airway remodeling in chronic asthma. Endogenous Nogo-B, which may exert its effects through ARPC 2/3 and MYL-9, is necessary for the   migratio  n and contraction of airway smooth muscle cells. 
 Nogo-B regulates   migration   and contraction of airway smooth muscle cells by decreasing ARPC 2/3 and increasing MYL-9 expression. Abnormal proliferation, apoptosis,   migration   and contraction of airway smooth muscle (ASM) cells in airway remodeling in asthma are basically excessive repair responses to a network of inflammatory mediators such as PDGF, but the mechanisms of such responses remain unclear. Nogo-B, a member of the reticulum family 4(RTN4), is known to play a key role in arteriogenesis and tissue repair. Further studies are needed to elucidate the role of Nogo-B in airway smooth muscle abnormalities.A mouse model of chronic asthma was established by repeated OVA inhalation and subjected to Nogo-B expression analysis using immunohistochemistry and Western Blotting. Then, primary human bronchial smooth muscle cells (HBSMCs) were cultured in vitro and a siRNA interference was performed to knockdown the expression of Nogo-B in the cells. The effects of Nogo-B inhibition on PDGF-induced HBSMCs proliferation,   migration   and contraction were evaluated. Finally, a proteomic analysis was conducted to unveil the underlying mechanisms responsible for the function of Nogo-B.Total Nogo-B expression was approximately 3.08-fold lower in chronic asthmatic mice compared to naïve mice, which was obvious in the smooth muscle layer of the airways. Interference of Nogo-B expression by siRNA resulted nearly 96% reduction in mRNA in cultured HBSMCs. In addition, knockdown of Nogo-B using specific siRNA significantly decreased PDGF-induced   migratio  n of HBSMCs by 2.3-fold, and increased the cellular contraction by 16% compared to negative controls, but had limited effects on PDGF-induced proliferation. Furthermore, using proteomic analysis, we demonstrate that the expression of actin related protein 2/3 complex subunit 5 (ARPC 2/3) decreased and, myosin regulatory light chain 9 isoform a (MYL-9) increased after Nogo-B knockdown.These data define a novel role for Nogo-B in airway remodeling in chronic asthma. Endogenous Nogo-B, which may exert its effects through ARPC 2/3 and MYL-9, is necessary for the   migratio  n and contraction of airway smooth muscle cells. 
 Nogo-B promotes the epithelial-mesenchymal transition in HeLa cervical cancer cells via Fibulin-5. Cervical cancer is a common malignancy in women worldwide, and the occurrence of invasion and metastasis is the major cause for most cancer-related deaths. Epithelial-mesenchymal transition (EMT) has been implicated in the metastasis of primary tumors and provides molecular mechanisms for cervical cancer metastasis. We previously reported that Nogo-B mediates cell motility by binding Fibulin-5. Herein, we show that the increased expression of Nogo-B is correlated with the degree of cervical cancer metastasis. In HeLa cervical cancer cells, overexpression of Nogo-B induces the EMT and promotes cell   migration   and invasion, while inhibiting cell adhesion. Furthermore, we found that Nogo-B accumulates and co-localizes with Fibulin-5 in pseudopods, and the downstream effects of overexpression of Nogo-B on cell motility could be partially abolished by RNA interference against Fibulin-5. These results suggest that Nogo-B functions as an inducer of cervical cancer metastasis and that this effect is mediated, at least in part, through Fibulin-5. 
 The association between dilated cardiomyopathy and RTN4 3'UTR insertion/deletion polymorphisms. The Nogo isoforms A, B and C are members of the reticulon family of proteins. Nogo-B is found in most tissues, especially highly expressed in endothelial and smooth muscle cells of the vessel wall, and Nogo-B is a regulator of cell   migration   in vitro and vascular remodeling in vivo. The TATC and CAA 3'UTR insertion/deletion polymorphisms of the RTN4, the gene encoding Nogo isoforms A, B and C, have been linked to schizophrenia and depression, but data were inconsistent. However, it is unclear whether these polymorphisms are associated with dilated cardiomyopathy (DCM).A total of 159 DCM patients and 215 control subjects were recruited in this study. The RTN4 TATC and CAA insertion/deletion genotypes were determined using PCR-polyacrylamide gel electrophoresis.Frequencies of (TATC)(2) allele and (TATC)(2)/(TATC)(2) genotype were significantly different from that in healthy controls (P = 0.045, OR = 1.356, 95% CI = 1.006-1.827 and P = 0.021, OR = 2.094, 95% CI = 1.113-3.940, respectively). No significant differences in CAA insertion/deletion genotype and allele frequencies were observed between the DCM and controls.These data provide evidence that RTN4 allele (TATC)(2) and (TATC)(2)/(TATC)(2) genotype are associated with DCM. 
 RNT4 3'-UTR insertion/deletion polymorphisms are not associated with atrial septal defect in Chinese Han population: a brief communication. Atrial septal defect (ASD) is a common type of congenital heart disease, which is defined as any communication through atrial septum. Several studies have revealed that genetic factors may influence the susceptibility of ASD. Recent studies have shown that reticulon 4 (RTN4) gene might be involved in some processes relevant to heart development, such as regulation of cell   migration   and vascular remodeling. This study aimed to evaluate RTN4 gene polymorphisms of CAA and TATC insertion/deletion in relation to the risk of ASD in Chinese Han population. A total of 175 ASD patients and 308 unrelated healthy controls were successfully investigated. The polymorphisms of patients were determined by polymerase chain reaction-polyacrylamide gel electrophoresis. There was no significant difference in the allele frequencies of CAA and TATC insertion/deletion in RNT4 gene between ASD patients and controls. The same results were seen in their genotypes. The present study suggests that CAA and TATC insertion/deletion polymorphisms of RNT4 gene may not be a useful marker to predict the susceptibility of ASD in Chinese Han population. 
 The ER structural protein Rtn4A stabilizes and enhances signaling through the receptor tyrosine kinase ErbB3. ErbB3 and ErbB4 are receptor tyrosine kinases that are activated by the neuregulin (NRG) family of growth factors. These receptors govern various developmental processes, and their dysregulation contributes to several human disease states. The abundance of ErbB3 and ErbB4, and thus signaling through these receptors, is limited by the E3 ubiquitin ligase Nrdp1, which targets ErbB3 and ErbB4 for degradation. Reticulons are proteins that influence the morphology of the endoplasmic reticulum (ER) by promoting the formation of tubules, a response of cells to some stressors. We found that the ER structural protein reticulon 4A (Rtn4A, also known as Nogo-A) increased ErbB3 abundance and proliferative signaling by suppressing Nrdp1 function. Rtn4A interacted with Nrdp1 and stabilized ErbB3 in an Nrdp1-dependent manner. Rtn4A overexpression induced the redistribution of Nrdp1 from a cytosolic or perinuclear localization to ER tubules. Rtn4A knockdown in human breast tumor cells decreased ErbB3 abundance, NRG-stimulated signaling, and cellular proliferation and   migration  . Because proteins destined for the plasma membrane are primarily synthesized in the sheet portions of the ER, our observations suggest that Rtn4A counteracts the Nrdp1-mediated degradation of ErbB3 by sequestering the ubiquitin ligase into ER tubules. The involvement of a reticulon suggests a molecular link between ER structure and the sensitivity of cells to receptor tyrosine kinase-mediated survival signals at the cell surface. 
 A new role for Nogo as a regulator of vascular remodeling. Although Nogo-A has been identified in the central nervous system as an inhibitor of axonal regeneration, the peripheral roles of Nogo isoforms remain virtually unknown. Here, using a proteomic analysis to identify proteins enriched in caveolae and/or lipid rafts (CEM/LR), we show that Nogo-B is highly expressed in cultured endothelial and smooth muscle cells, as well as in intact blood vessels. The N terminus of Nogo-B promotes the   migration   of endothelial cells but inhibits the   migration   of vascular smooth muscle (VSM) cells, processes necessary for vascular remodeling. Vascular injury in Nogo-A/B-deficient mice promotes exaggerated neointimal proliferation, and adenoviral-mediated gene transfer of Nogo-B rescues the abnormal vascular expansion in those knockout mice. Our discovery that Nogo-B is a regulator of vascular homeostasis and remodeling broadens the functional scope of this family of proteins. 
 Nogo-B mediates HeLa cell adhesion and motility through binding of Fibulin-5. Nogo-B is a known regulator of neural functions and plays an important role in cell adhesion and   migration  . To our knowledge, the molecular mechanism behind its regulation of cell motility is still unknown. Here, we identified Fibulin-5, a secreted extracellular matrix protein, as a binding partner of Nogo-B. Using HeLa cells as a model, we found that Nogo-B and Fibulin-5 co-localize in the cytoplasm and plasma membrane. Furthermore, in HeLa cells that overexpress Nogo-B, cell   migration   and invasion was promoted by the elevated secretion of Fibulin-5. Thus, identification of the Nogo-B binding protein Fibulin-5 may contribute to uncover the pathway in which Nogo-B regulates tumor cell movement. 
 Endothelial reticulon-4B (Nogo-B) regulates ICAM-1-mediated leukocyte trans  migration   and acute inflammation. The reticulon (Rtn) family of proteins are localized primarily to the endoplasmic reticulum (ER) of most cells. The Rtn-4 family, (aka Nogo) consists of 3 splice variants of a common gene called Rtn-4A, Rtn-4B, and Rtn-4C. Recently, we identified the Rtn-4B (Nogo-B) protein in endothelial and smooth muscle cells of the vessel wall, and showed that Nogo-B is a regulator of cell   migration   in vitro and vascular remodeling and angiogenesis in vivo. However, the role of Nogo-B in inflammation is still largely unknown. In the present study, we use 2 models of inflammation to show that endothelial Nogo-B regulates leukocyte trans  migration   and intercellular adhesion molecule-1 (ICAM-1)-dependent signaling. Mice lacking Nogo-A/B have a marked reduction in neutrophil and monocyte recruitment to sites of inflammation, while Nogo-A/B(-/-) mice engrafted with wild-type (WT) bone marrow still exhibit impaired inflammation compared with WT mice engrafted with Nogo-A/B(-/-) bone marrow, arguing for a critical role of host Nogo in this response. Using human leukocytes and endothelial cells, we show mechanistically that the silencing of Nogo-B with small interfering RNA (siRNA) impairs the trans  migration   of neutrophils and reduces ICAM-1-stimulated phosphorylation of vascular endothelial-cell cadherin (VE-cadherin). Our results reveal a novel role of endothelial Nogo-B in basic immune functions and provide a key link in the molecular network governing endothelial-cell regulation of diapedesis. 
 Phosphorylation of Nogo receptors suppresses Nogo signaling, allowing neurite regeneration. The myelin-associated proteins Nogo-A, MAG, and OMgp transmit signals from oligodendrocytes into neurons through binding to Nogo receptors. Nogo signaling has critical roles in development and maintenance of the central nervous system (CNS). It can inhibit differentiation,   migration  , and neurite outgrowth of neurons, causing poor recovery of the adult CNS from damage. Here, I show that phosphorylation of Nogo receptors by casein kinase II (CK2) inhibits binding of the myelin-associated proteins. Brain-derived neurotrophic factor stimulates the phosphorylation, suppressing Nogo-dependent inhibition of neurite outgrowth from neuroblastoma-derived neural cells. Similarly, in rat adult neurons, extracellular CK2 treatment overcomes inhibition of neurite outgrowth by the myelin-associated proteins. These findings provide new strategies to control Nogo signaling and hence neuronal regeneration. 
  GeneRIFs  
 Neither   migration   speed, nor cell proliferation, or layer area sizes were influenced by Nogo-A deletion, hence suggesting another role for early postnatal Nogo-A expression in the premigratory zone of the external granule layer. 
 Endogenous Nogo-B, which may exert its effects through ARPC 2/3 and MYL-9, is necessary for the   migration   and contraction of airway smooth muscle cells. 
 Using leukocytes and endothelial cells, we show mechanistically that the silencing of Nogo-B with siRNA impairs the trans  migration   of neutrophils and reduces ICAM-1-stimulated phosphorylation of VE-cadherin. 
  [Ee]xtracellular [Mm]atrix  PubMed  
 The N-terminal domain of Nogo-A inhibits cell adhesion and axonal outgrowth by an integrin-specific mechanism. Myelin-derived Nogo-A protein limits axonal growth after CNS injury. One domain binds to the Nogo-66 receptor to inhibit axonal outgrowth, whereas a second domain, Amino-Nogo, inhibits axonal outgrowth and cell adhesion through unknown mechanisms. Here, we show that Amino-Nogo inhibition depends strictly on the composition of the   extracellular matrix  , suggesting that Amino-Nogo inhibits the function of certain integrins. Amino-Nogo inhibition can be partially overcome by antibodies that activate integrin beta1 or by the addition of Mn2+, an integrin activator. Furthermore, Amino-Nogo reduces focal adhesion kinase activation by fibronectin. Analysis of various cell lines reveals that alpha(v)beta3, alpha5, and alpha4 integrins are sensitive to Amino-Nogo, but alpha6 integrin is not. Both alpha(v) and alpha5 integrins have widespread expression in adult brain and are found in axonal growth cones. Thus, inhibition of integrin signaling by Amino-Nogo contributes to the failure of CNS axon regeneration. 
 Nogo-B mediates HeLa cell adhesion and motility through binding of Fibulin-5. Nogo-B is a known regulator of neural functions and plays an important role in cell adhesion and migration. To our knowledge, the molecular mechanism behind its regulation of cell motility is still unknown. Here, we identified Fibulin-5, a secreted   extracellular matrix   protein, as a binding partner of Nogo-B. Using HeLa cells as a model, we found that Nogo-B and Fibulin-5 co-localize in the cytoplasm and plasma membrane. Furthermore, in HeLa cells that overexpress Nogo-B, cell migration and invasion was promoted by the elevated secretion of Fibulin-5. Thus, identification of the Nogo-B binding protein Fibulin-5 may contribute to uncover the pathway in which Nogo-B regulates tumor cell movement. 
  GeneRIFs  
 Fibulin-5, a secreted   extracellular matrix   protein, was identified as a binding partner of Nogo-B. 
  [Ii]nvasive  PubMed  
 Expression of Nogo isoforms and Nogo-B receptor (NgBR) in non-small cell lung carcinomas. Nogo-B was recently shown to be involved in proliferation, apoptosis and   invasive  ness of cancer cells, whereas its specific receptor (NgBR) was found to be up-regulated in estrogen receptor-α positive breast cancer. No data are currently available concerning their expression in non-small cell lung carcinomas (NSCLC).Expression of Nogo isoforms and NgBR was studied in 191 NSCLC.Higher Nogo-A/B immunoreactivity was noted in cancer cells of squamous cell carcinomas (SQC) compared to adenocarcinomas (p<0.001). Stage II-IV tumors had the lowest Nogo-A/B expression (p<0.0001) compared to stage I cases. Nogo-A/B expression decreased with increasing SQC malignancy grade (p=0.026). Significant NgBR mRNA down-regulation was associated with larger primary tumor size (p=0.039), lymph node involvement (p=0.039) and advancement stage (p=0.0054). Low NgBR mRNA expression predicted poor patients outcome (p=0.029).The current data may point to the involvement of Nogo isoforms and NgBR in the pathogenesis of NSCLC. 
  [Mm]etastasis  PubMed  
 Down-Regulation of Nogo-B Expression as a Newly Identified Feature of Intrahepatic Cholangiocarcinoma. Nogo-B, located in the endoplasmic reticulum, is an isoform belonging to the reticulon protein family, which is expressed specifically in cholangiocytes and non-parenchymal cells in the liver. Nogo-B expression is down-regulated with the progression of liver fibrosis, but its distinct function in liver malignancies has not been fully clarified. We have hypothesized that Nogo-B expression may be altered in intrahepatic cholangiocarcinoma (ICC), a relatively rare type of primary liver cancer with highly malignant behavior. The present study aimed to investigate the relationship between Nogo-B expression, assessed by immunohistochemical staining, and clinicopathological factors and prognosis in 34 ICC patients. Positive expression was observed in 19 (56%) of 34 ICC specimens: 6 patients (18%) with positivity levels of 1+ (positive cells in 10-50% of cancer cells) and 13 patients (38%) with 2+ (positive cells over 50%). Importantly, the remaining 15 patients (44%) were categorized as negative expression (Nogo-B-positive cells, less than 10%). Conversely, the mass-forming type of ICC tended to express Nogo-B with the degree of 2+ positivity, compared to the periductal infiltration type (p = 0.064), and the mass-forming type showed a better 5-year survival rate (66% vs. 5%) after hepatectomy (p < 0.05). However, the degree of positivity was not associated with tumor relapse rate, disease-free and overall survival, although each of the periductal infiltration type, intrahepatic   metastasis  , larger tumor size, and lower microvessel counts was associated with lower survival rates. We propose that Nogo-B expression is down-regulated in ICC, the implication of which, however, remains to be investigated. 
 Nogo-B promotes the epithelial-mesenchymal transition in HeLa cervical cancer cells via Fibulin-5. Cervical cancer is a common malignancy in women worldwide, and the occurrence of invasion and   metastasis   is the major cause for most cancer-related deaths. Epithelial-mesenchymal transition (EMT) has been implicated in the   metastasis   of primary tumors and provides molecular mechanisms for cervical cancer   metastasis  . We previously reported that Nogo-B mediates cell motility by binding Fibulin-5. Herein, we show that the increased expression of Nogo-B is correlated with the degree of cervical cancer   metastasis  . In HeLa cervical cancer cells, overexpression of Nogo-B induces the EMT and promotes cell migration and invasion, while inhibiting cell adhesion. Furthermore, we found that Nogo-B accumulates and co-localizes with Fibulin-5 in pseudopods, and the downstream effects of overexpression of Nogo-B on cell motility could be partially abolished by RNA interference against Fibulin-5. These results suggest that Nogo-B functions as an inducer of cervical cancer   metastasis   and that this effect is mediated, at least in part, through Fibulin-5. 
   
  
 RTN3 
 
  NCBI Gene  
  GeneRIF  
  Pubmed  
 
 
  Search Term  Rank  Fields  
  [Mm]igration  6  PubMed(2); GeneRIFs(1)  
 
 
 hide/show details for RTN3 
  [Mm]igration  PubMed  
 RTN3 Regulates the Expression Level of Chemokine Receptor CXCR4 and is Required for   Migration   of Primordial Germ Cells. CXCR4 is a crucial chemokine receptor that plays key roles in primordial germ cell (PGC) homing. To further characterize the CXCR4-mediated   migration   of PGCs, we screened CXCR4-interacting proteins using yeast two-hybrid screening. We identified reticulon3 (RTN3), a member of the reticulon family, and considered an apoptotic signal transducer, as able to interact directly with CXCR4. Furthermore, we discovered that the mRNA and protein expression levels of CXCR4 could be regulated by RTN3. We also found that RTN3 altered CXCR4 translocation and localization. Moreover, increasing the signaling of either CXCR4b or RTN3 produced similar PGC mislocalization phenotypes in zebrafish. These results suggested that RTN3 modulates PGC   migration   through interaction with, and regulation of, CXCR4. 
 The potential role of RTN3 in monocyte recruitment and atherosclerosis. The recruitment of monocytes to arterial wall and their transformation into macrophages are generally accepted as important early events in the pathogenesis of atherosclerosis (AS). Our research group found Reticulon3 (RTN3), a member of the reticulon family, may be a candidate pathogenic element in the progress of AS. But it is virtually unknown in which process RTN3 may participate in and regulate the pathogenesis of AS. Here, we hypothesis that RTN3 may participate in the continuous process of circulating monocyte recruitment in AS including: (1) monocyte spreading and adhesion to luminal endothelium; (2) transendothelial   migration   and may also contribute to the conversion of monocyte to macrophage in subendothelium. 
  GeneRIFs  
 These results suggested that RTN3 modulates primordial germ cell   migration   through interaction with, and regulation of, CXCR4. 
   
  
 RRBP1 
 
  NCBI Gene  
  GeneRIF  
  Pubmed  
 
 
  Search Term  Rank  Fields  
  [Ee]xtracellular [Mm]atrix  8  PubMed(1)  
 
 
 hide/show details for RRBP1 
  [Ee]xtracellular [Mm]atrix  PubMed  
 Identification, characterization, and chromosomal localization of the human homolog (hES) of ES/130. The chicken   extracellular matrix   glycoprotein ES/ 130 is necessary for epithelial--mesenchymal transformation in the developing heart and is also expressed in noncardiac chicken tissues such as limb and notochord. We have identified hES, the human homolog of chicken ES/130. Fluorescence in situ hybridization analysis (FISH) localizes hES to human chromosome 20p11.2-p12. FISH analyses of individuals with 20p12 deletions and affected by Alagille syndrome exclude hES as a candidate gene for this disorder. Reverse transcriptase-polymerase chain reaction studies reveal that hES is expressed in both fetal and adult human tissues and that hES expression in the left ventricle is increased in the failing adult heart. Further studies will evaluate how hES mutations may relate to congenital human cardiac and skeletal anomalies as well as cardiac remodeling in the adult. 
   
  
 CADM2 
 
  NCBI Gene  
  GeneRIF  
  Pubmed  
 
 
  Search Term  Rank  Fields  
  ECM  7  PubMed(1)  
 
 
 hide/show details for CADM2 
  ECM  PubMed  
 Genetic analysis in a Dutch study sample identifies more ulcerative colitis susceptibility loci and shows their additive role in disease risk. Genetic susceptibility is known to make a major contribution to the pathogenesis of ulcerative colitis (UC). Recently, three studies, including a genome-wide association study (GWAS), reported novel UC risk loci.The top-20 single-nucleotide polymorphisms (SNPs) from the first UC-GWAS were genotyped, as part of the study's replication phase, in 561 UC cases and 728 controls from our Dutch UC study sample. We genotyped eight SNPs identified in two more studies, in these individuals, and replicated all significantly associated SNPs in an additional 894 UC cases and 1,174 controls from our Dutch UC study sample. A combined analysis for all patients (n=1,455) and controls (n=1,902) was performed. Dose-response models were constructed with the associated risk alleles.We found 12 SNPs tagging ten loci, including HLA-DRA, IL10, IL23R, JAK2, S100Z, ARPC2, and   ECM  1, to be associated with UC. We identified 10q26, flagged by the UC-GWAS but not confirmed in its replication phase, as a UC locus and found a trend toward association for GAS7. No association with disease localization or severity was found. The dose-response models show that individuals carrying 11 or more risk alleles have an odds ratio of 8.2 (confidence interval 3.0-22.8) for UC susceptibility.We confirmed the association of multiple loci with UC in the Dutch population and found evidence for association of 10q26 and a trend suggesting association for GAS7. Genetic models show that multiple risk loci in an individual increase the risk for developing UC. 
   
  
 CADM3 
 
  NCBI Gene  
  GeneRIF  
  Pubmed  
 
 
  Search Term  Rank  Fields  
  [Ff]ilopodia  1  PubMed(1)  
  [Mm]igration  6  PubMed(2); GeneRIFs(2)  
  [Ee]xtracellular [Mm]atrix  8  PubMed(1); GeneRIFs(1)  
  [Ii]nvasive  10  PubMed(2)  
  [Mm]etastasis  12  PubMed(2)  
 
 
 hide/show details for CADM3 
  [Ff]ilopodia  PubMed  
 HVEM and nectin-1 are the major mediators of herpes simplex virus 1 (HSV-1) entry into human conjunctival epithelium. The human conjunctiva is a natural target for herpes simplex virus (HSV)-1 infection. The goals of this study were to investigate the cellular and molecular mechanisms of HSV-1 entry into human conjunctival epithelial (HCjE) cells. Specific features of entry studied included the method of initial viral binding to cells, pH dependency, and expression and usage of specific HSV-1 entry receptors.To observe HSV-1 initial binding, live cell imaging was performed on HSV-1-infected HCjE cells. Reporter HSV-1 virions expressing beta-galactosidase were used to determine entry of wild-type HSV-1(KOS) and a mutant, HSV-1(KOS)Rid1, into HCjE cells. HSV-1 replication in HCjE cells was determined by plaque assays. Lysosomotropic agents were used to determine whether viral entry was pH dependent. Reverse transcription (RT)-PCR, flow cytometry, and immunohistochemistry were used to determine the expression of receptors. Receptor-specific siRNAs were used to define the role of individual entry receptors.HSV-1 virions attach to   filopodia   present on HCjE cells and use them to reach the cell body for entry. Cultured HCjE cells demonstrate susceptibility to HSV-1 entry and form plaques confirming viral replication. Blocking vesicular acidification significantly reduces entry, implicating a pH-dependent mode of entry. Multiple assays confirm the expression of entry receptors nectin-1, HVEM, and 3-O-sulfated heparan sulfate (3-OS HS) on the HCjE cell membrane. Knocking down of gD receptors by siRNAs interference implicates nectin-1 and HVEM as the major mediators of entry.HSV-1 entry into HCjE cells is a pH-dependent process that is aided by targeted virus travel on   filopodia  . HCjE cells express all three major entry receptors, with nectin-1 and HVEM playing the predominant role in mediating entry. 
  [Mm]igration  PubMed  
 Nectin-like molecule 1 inhibits the   migration   and invasion of U251 glioma cells by regulating the expression of an extracellular matrix protein osteopontin. To investigate the molecular mechanism of nectin-like molecule 1 (NECL1) inhibiting the   migration   and invasion of U251 glioma cells.We infected U251 glioma cells with adeno-nectin-like molecule 1 (Ad-NECL1) or empty adenovirus (Ad). Transwell and wound healing assays were performed to observe the   migration   of U251 cells incubated with the cell supernatant from Ad-NECL1 or Ad infected U251 cells. DNA microarray was applied to screen the gene expression profile after the restoration of NECL1 in U251 glioma cell lines. The differential expression of osteopontin (OPN), a gene related to   migration   and invasion, was further analyzed with semi-quantitative reverse transcription-polymerase chain reaction (RT-PCR), Western blot, and immunohistochemistry.The restoration of NECL1 inhibited   migration   of U251 cells significantly (P<0.05). Altogether 195 genes were found differentially expressed by microarray, in which 175 were up-regulated and 20 down-regulated, including 9 extracellular matrix proteins involved in the   migration   of cells. Both mRNA and protein expressions of OPN, the most markedly reduced extracellular matrix protein, were found decreased in U251 cells after restoration of NECL1. Immunohistochemical assay also detected an increase of OPN in glioma tissues, related with the progressing of malignant grade.A link might exist between NECL1 and the extracellular matrix protein OPN in inhibiting the   migration   and invasion of U251 glioma cells. 
 [Neural adhesion molecule NECL1 inhibits   migration  , invasion, and potentially induces differentiation of glioma cell]. To explore the influences of the restoration of neural adhesion molecule NECL1 on the morphology,   migration  , and invasion of NECL1-deficient glioma cell lines.Scratch and Transwell assays were used to observe the cell   migration   and invasion, the activities of extracellular metalloproteinases were measured, and the cell morphology was observed. Astrocytes marker glial fibrillary acidic protein was detected by Western blot after the restoration of NECL1 in glioma U251 cell line.In NECL1-deficient U251 glioma cell lines,   migration   and invasion were inhibited. The U251 cells was differentiated potentially to astrocytes, and glial fibrillary acidic protein was up-regulated after the restoration of the NECL1 expression.As a potential tumor repressor, the neural adhesion molecule NECL1 can inhibit the   migration   and invasion of glioma cell and induces its differentiation. 
  GeneRIFs  
 NECL1 can inhibit the   migration   and invasion of glioma cells and induce differentiation. 
 A link might exist between NECL1 and the extracellular matrix protein OPN in inhibiting the   migration   and invasion of U251 glioma ce 
  [Ee]xtracellular [Mm]atrix  PubMed  
 Nectin-like molecule 1 inhibits the migration and invasion of U251 glioma cells by regulating the expression of an   extracellular matrix   protein osteopontin. To investigate the molecular mechanism of nectin-like molecule 1 (NECL1) inhibiting the migration and invasion of U251 glioma cells.We infected U251 glioma cells with adeno-nectin-like molecule 1 (Ad-NECL1) or empty adenovirus (Ad). Transwell and wound healing assays were performed to observe the migration of U251 cells incubated with the cell supernatant from Ad-NECL1 or Ad infected U251 cells. DNA microarray was applied to screen the gene expression profile after the restoration of NECL1 in U251 glioma cell lines. The differential expression of osteopontin (OPN), a gene related to migration and invasion, was further analyzed with semi-quantitative reverse transcription-polymerase chain reaction (RT-PCR), Western blot, and immunohistochemistry.The restoration of NECL1 inhibited migration of U251 cells significantly (P<0.05). Altogether 195 genes were found differentially expressed by microarray, in which 175 were up-regulated and 20 down-regulated, including 9   extracellular matrix   proteins involved in the migration of cells. Both mRNA and protein expressions of OPN, the most markedly reduced   extracellular matrix   protein, were found decreased in U251 cells after restoration of NECL1. Immunohistochemical assay also detected an increase of OPN in glioma tissues, related with the progressing of malignant grade.A link might exist between NECL1 and the   extracellular matrix   protein OPN in inhibiting the migration and invasion of U251 glioma cells. 
  GeneRIFs  
 A link might exist between NECL1 and the   extracellular matrix   protein OPN in inhibiting the migration and invasion of U251 glioma ce 
  [Ii]nvasive  PubMed  
 The cell adhesion nectin-like molecules (Necl) 1 and 4 suppress the growth and tumorigenic ability of colon cancer cells. A key step in human colon cancer development includes the hyperactivation of Wnt/beta-catenin signaling and the induction of beta-catenin-TCF target genes that participate in colon cancer progression. Recent studies identified members of the immunoglobulin-like cell adhesion molecules (IgCAM) of the L1CAM family (L1 and Nr-CAM) as targets of beta-catenin-TCF signaling in colon cancer cells. L1 was detected at the   invasive   front of colon cancer tissue and confers metastasis when overexpressed in cells. In contrast to L1, we did not detect in colon cancer cells significant levels of another IgCAM family of molecules, the nectin-like (Necl) receptors Necl1 and Necl4, while Necl4 was previously found in the normal small intestine and colon tissues. We studied the properties of colon cancer cells in which Necl4 and Necl1 were expressed either alone, or in combination, and found that such cells display a wide range of properties associated with tumor suppression. Expression of both Necl1 and Necl4 was the most efficient in suppressing the tumorigenicity of colon cancer cells. This was associated with enhanced rates of apoptosis and change in several apoptosis-related markers. In contrast to its capacity to suppress tumorigenesis, Necl4 was unable to affect the highly malignant and metastatic capacities of colon cancer cells in which L1 was overexpressed. Our results suggest that various IgCAM receptor families play different roles in affecting the tumorigenic function of the same cells, and that Necl1 and Necl4 can fulfill a tumor suppressive role. 
 The cell adhesion nectin-like molecules (Necl) 1 and 4 suppress the growth and tumorigenic ability of colon cancer cells. A key step in human colon cancer development includes the hyperactivation of Wnt/beta-catenin signaling and the induction of beta-catenin-TCF target genes that participate in colon cancer progression. Recent studies identified members of the immunoglobulin-like cell adhesion molecules (IgCAM) of the L1CAM family (L1 and Nr-CAM) as targets of beta-catenin-TCF signaling in colon cancer cells. L1 was detected at the   invasive   front of colon cancer tissue and confers metastasis when overexpressed in cells. In contrast to L1, we did not detect in colon cancer cells significant levels of another IgCAM family of molecules, the nectin-like (Necl) receptors Necl1 and Necl4, while Necl4 was previously found in the normal small intestine and colon tissues. We studied the properties of colon cancer cells in which Necl4 and Necl1 were expressed either alone, or in combination, and found that such cells display a wide range of properties associated with tumor suppression. Expression of both Necl1 and Necl4 was the most efficient in suppressing the tumorigenicity of colon cancer cells. This was associated with enhanced rates of apoptosis and change in several apoptosis-related markers. In contrast to its capacity to suppress tumorigenesis, Necl4 was unable to affect the highly malignant and metastatic capacities of colon cancer cells in which L1 was overexpressed. Our results suggest that various IgCAM receptor families play different roles in affecting the tumorigenic function of the same cells, and that Necl1 and Necl4 can fulfill a tumor suppressive role. 
  [Mm]etastasis  PubMed  
 The cell adhesion nectin-like molecules (Necl) 1 and 4 suppress the growth and tumorigenic ability of colon cancer cells. A key step in human colon cancer development includes the hyperactivation of Wnt/beta-catenin signaling and the induction of beta-catenin-TCF target genes that participate in colon cancer progression. Recent studies identified members of the immunoglobulin-like cell adhesion molecules (IgCAM) of the L1CAM family (L1 and Nr-CAM) as targets of beta-catenin-TCF signaling in colon cancer cells. L1 was detected at the invasive front of colon cancer tissue and confers   metastasis   when overexpressed in cells. In contrast to L1, we did not detect in colon cancer cells significant levels of another IgCAM family of molecules, the nectin-like (Necl) receptors Necl1 and Necl4, while Necl4 was previously found in the normal small intestine and colon tissues. We studied the properties of colon cancer cells in which Necl4 and Necl1 were expressed either alone, or in combination, and found that such cells display a wide range of properties associated with tumor suppression. Expression of both Necl1 and Necl4 was the most efficient in suppressing the tumorigenicity of colon cancer cells. This was associated with enhanced rates of apoptosis and change in several apoptosis-related markers. In contrast to its capacity to suppress tumorigenesis, Necl4 was unable to affect the highly malignant and metastatic capacities of colon cancer cells in which L1 was overexpressed. Our results suggest that various IgCAM receptor families play different roles in affecting the tumorigenic function of the same cells, and that Necl1 and Necl4 can fulfill a tumor suppressive role. 
 The cell adhesion nectin-like molecules (Necl) 1 and 4 suppress the growth and tumorigenic ability of colon cancer cells. A key step in human colon cancer development includes the hyperactivation of Wnt/beta-catenin signaling and the induction of beta-catenin-TCF target genes that participate in colon cancer progression. Recent studies identified members of the immunoglobulin-like cell adhesion molecules (IgCAM) of the L1CAM family (L1 and Nr-CAM) as targets of beta-catenin-TCF signaling in colon cancer cells. L1 was detected at the invasive front of colon cancer tissue and confers   metastasis   when overexpressed in cells. In contrast to L1, we did not detect in colon cancer cells significant levels of another IgCAM family of molecules, the nectin-like (Necl) receptors Necl1 and Necl4, while Necl4 was previously found in the normal small intestine and colon tissues. We studied the properties of colon cancer cells in which Necl4 and Necl1 were expressed either alone, or in combination, and found that such cells display a wide range of properties associated with tumor suppression. Expression of both Necl1 and Necl4 was the most efficient in suppressing the tumorigenicity of colon cancer cells. This was associated with enhanced rates of apoptosis and change in several apoptosis-related markers. In contrast to its capacity to suppress tumorigenesis, Necl4 was unable to affect the highly malignant and metastatic capacities of colon cancer cells in which L1 was overexpressed. Our results suggest that various IgCAM receptor families play different roles in affecting the tumorigenic function of the same cells, and that Necl1 and Necl4 can fulfill a tumor suppressive role.
